# Integrated analysis of miR-15a-5p, miR-20a-5p, and miR-33b-3p identifies EGR2-associated biomarkers in multiple myeloma

**DOI:** 10.1186/s12885-026-15610-5

**Published:** 2026-02-12

**Authors:** Riham Abdel-Hamid Haroun, Nada M. Ismail, Samar S. Elshazly, Fatma F. Abdel Hamid, Reem Nabil

**Affiliations:** 1 Biochemistry Department, Faculty of Science, Ain Shams University, Cairo, Egypt; 2 Clinical Pathology Department, National Cancer Institute, Cairo University, Cairo, Egypt

**Keywords:** Multiple myeloma, MicroRNA, miR-15a, miR-20a, miR-33b, EGR2 protein

## Abstract

**Introduction:**

Recently miR-15a-5p, miR-20a-5p and miR-33b-3p were found to be abnormally expressed in multiple myeloma (MM) patients. However, the significance of these microRNAs in MM pathogenesis remains poorly understood. Therefore the aim of the current study was to make an integrated bioinformatics analysis of miR-15a-5p, miR-20a-5p and miR-33b-3p to identify the biological processes in MM pathogenesis.

**Methods:**

The *Steven K. Thompson* equation and a-priori power analysis was conducted to determine the minimum sample size required. Forty patients with newly diagnosed MM and staged by the International Staging System (ISS) and 20 healthy bone marrow donors were included in our study. The baseline bone marrow miR-15a-5p, miR-20a-5p and miR-33b-3p expression levels were evaluated by RTqPCR technique. Moreover, EGR2 expression level was evaluated by using immunohistochemistry. A bioinformatics analysis for miR-15a-5p, miR-20a-5p, miR-33b-3p and EGR2 was done by using miRNet and miRDB (for target genes prediction), GO analysis and KEGG analysis (for biological processes identification) and STRING database (for PPI network).

**Results:**

The target genes of miR-15a-5p, miR-20a-5p and miR-33b-3p were retrieved then GO analysis was done which showed that the pathway with many shared target genes was MAPK signaling pathway that involved in the cell cycle control. The baseline bone marrow miR-15a-5p, miR-20a-5p& miR-33b-3p expression levels were significantly decreased (0.29 ± 0.04, *p* < 0.001;0.42 ± 0.06, *p* < 0.001;0.51 ± 0.07, *p* = 0.02 fold-change relative to controls) in MM patients when compared to controls (1.04 ± 0.11,1.02 ± 0.07&1.05 ± 0.11;respectively) while EGR2 protein was significantly increased in MM patients when compared to controls. Results obtained from ROC curve revealed that miR-15a-5p + miR-20a-5p + miR-33b-3p panel (AUC = 0.98, 100%sensitivity, 85%specificity, *p* < 0.001) and EGR2 protein expression (AUC = 0.986, 97.5%sensitivity, 90%specificity, *p* < 0.001) were the best ones as diagnostic biomarkers could differentiate MM disease. By using Kaplan − Meier survival test, the mean (95%CI) for OS was 24.08 (21.58–29.88) months throughout the 32-month follow-up period for MM patients, our results indicated that patients with lower expression levels of miR-15a-5p, miR-20a-5p&miR-33b-3p and higher expression level of EGR2 protein had poorer prognosis and possessing a shorter OS. The logistic regression analysis indicates that miR-15a-5p, miR-20a-5p, and miR-33b-3p are risk factors for the development of MM (OR = 9.36,2.56,4.76; 95% CI 4.02–21.28,1.28–5.32,2.43–9.16; *p* < 0.001, *p* = 0.016, *p* < 0.001;respectively).

**Conclusion:**

Finally miR-15a-5p, miR-20a-5p, and miR-33b-3p may have roles in MM pathogenesis through cell cycle control.

**Supplementary Information:**

The online version contains supplementary material available at 10.1186/s12885-026-15610-5.

## Introduction

 Multiple myeloma (MM) is the second most prevalent hematologic malignancy, following non-Hodgkin lymphoma, and is distinguished by the presence of more than 10% plasma cells in the bone marrow [1, 2]. It induces multiple organ failure attributable to CRAB: *C*alcium increase, *R*enal dysfunction, *A*nemia, and *B*one disease [3]. Over the past 20 years, new medications have increased patient survival, but MM is still an incurable condition [4]. The therapeutic landscape for MM patients has significantly advanced in the past decade, emphasizing minimal residual disease and negative complete remission through methods like autologous bone marrow transplantation. However, novel treatments that directly target myeloma cells and their microenvironment are desperately needed [5]. Growing evidence reveals that microRNAs play critical roles in the development and progression of human malignancies, including MM [6–8]. MicroRNAs (miRNAs) are non-coding, single-stranded RNA transcripts that occur in both plants and mammals. They are generally 18-25nt in length. They serve a vital role in controlling gene expression by silencing or degrading mRNA. As a result, miRNAs play important roles in cell survival, proliferation, and differentiation [9, 10]. Since their discovery, microRNAs have held considerable promise for cancer detection, prognosis, and treatment [11]. The first proof of miRNA participation in MM pathogenesis was presented by Al Masri et al. [12] who discovered that MM patient samples and MM cell lines had considerably lower levels of miR-125b, miR-133a, miR-1, miR-124a, miR-15, and miR-16 than healthy cells [12].

The miR-15 is a potent cell cycle and survival regulator that is a member of the MIR15A/MIR16-1 cluster and is found on the 13q14 chromosome [13, 14]. It was observed that miR-15 expression may be decreased in MM patients, which could be linked to MM pathogenesis [15]. Moreover, miR-20a is a member of the miR-17-92 cluster and has been found to be substantially associated with cancer invasion, metastasis, proliferation, and chemotherapy resistance [16]. Also, miR-20a has been found to directly target early growth response 2 (EGR2), as the levels of EGR2 protein and mRNA were either downregulated or increased in response to the overexpression or knockdown of miR-20a, respectively. Furthermore, the dual-luciferase reporter gene tests indicated that EGR2 is a direct target of miR-20a [17]. Moreover, reduced expression of miR-33b is also associated with the development of cancer, and it has been shown that miR-33b functions as a tumor suppressor in cancer [18, 19]. The investigation of miR-15a-5p, miR-20a-5p, miR-33b-3p together is novel and may be particularly promising for MM through regulation a common pathway or disease hallmark. Therefore, the current study intended to investigate the molecular roles and biological processes involved in MM pathogenesis by using an integrated bioinformatics analysis of miR-15a-5p, miR-20a-5p, miR-33b-3p and EGR2.

## Patients and methods

### Study design and participants

The Steven K. Thompson Eq [20]. was used to determine the sample size:

$$n=\frac{N\times Z^2\times P\times\left(1-P\right)}{d^2\times\left(N-1\right)+Z^2\times P\times\left(1-P\right)}$$

Where n is the minimum required sample size, N is the total population size, Z is the Z-score corresponding to the desired confidence level (1.96 for a 95% confidence level), P is the expected proportion or probability (often set at 0.50 for maximum variability if the true proportion is unknown), d is the error proportion (0.05 for a 5% error proportion).

**Fig. 1:**
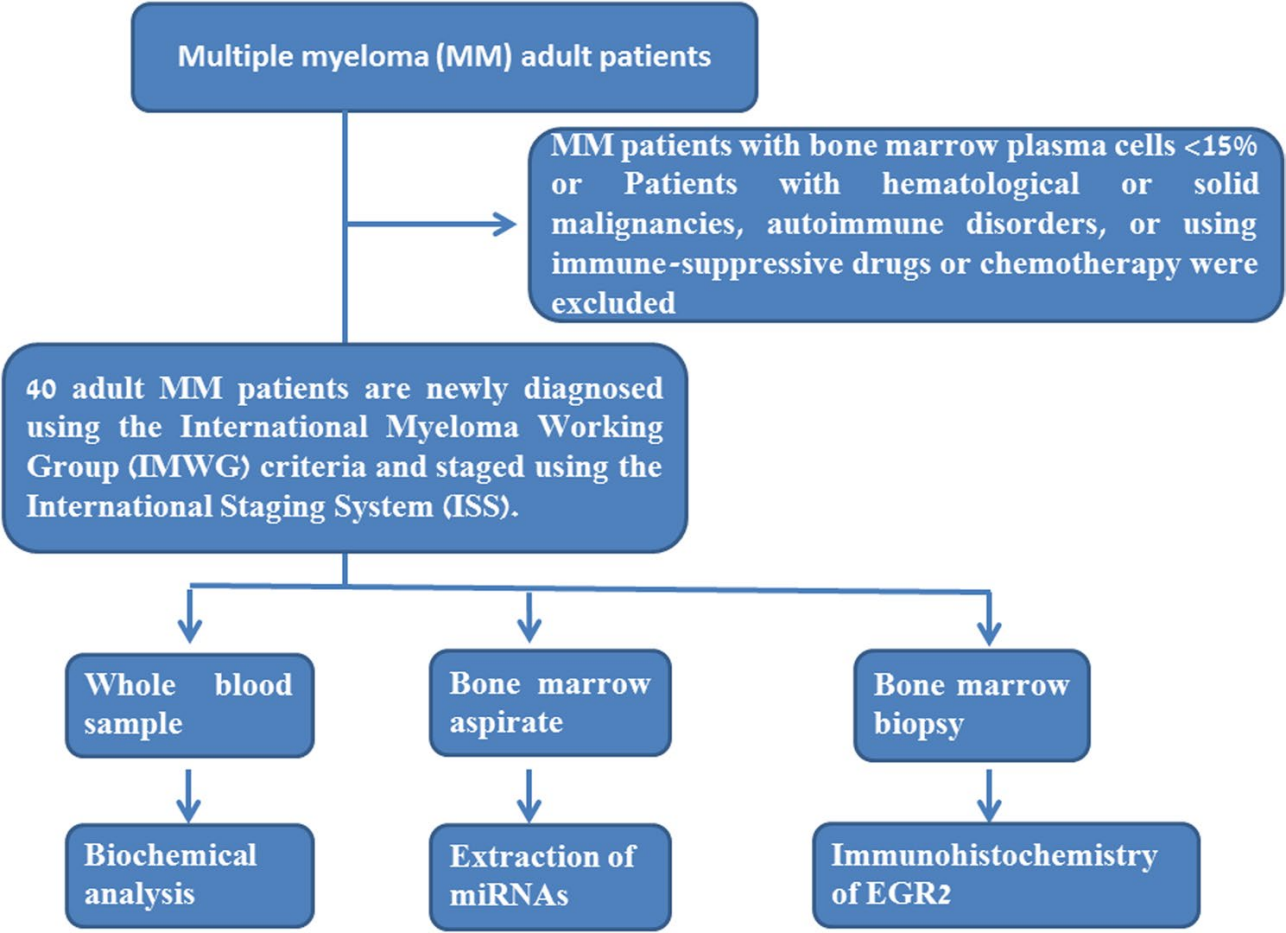
Patient recruitment flow chart: Newly diagnosed untreated MM adult patients without concurrent malignancy were included, while patients with hematological or solid malignancies, autoimmune disorders, or using immune-suppressive drugs or chemotherapy were excluded

Then a-priori power analysis was conducted before a study began to determine the minimum sample size required to have a reasonable chance of detecting a true effect of a specific size. This study included forty patients with newly diagnosed MM before initiating any treatment and twenty control participants who underwent bone marrow harvest for allogeneic transplantation and without hematological diseases i.e. healthy bone marrow donors were collected from the National Cancer Institute (NCI), Cairo University in Egypt. The patient recruitment flow chart was shown in Fig. 1. Patients with MM are diagnosed using the International Myeloma Working Group (IMWG) criteria [21] and staged using the International Staging System (ISS) [22]. The inclusion criteria were newly diagnosed untreated MM adult (age > 18 years) patients with no concomitant malignancy other than MM. In our research, we only included MM patients with marrow plasma cells ≥ 15% since, according to Gutiérrez et al. [23], patients with higher BM plasma cell infiltration were included in a genetic inquiry to detect genetic anomalies. Patients with hematological or solid malignancies, autoimmune disorders, or using immune-suppressive drugs or chemotherapy were excluded. The current study was approved by the Institutional Review Board of Cairo University’s National Cancer Institute in Cairo, Egypt (IRB00004025, Approval No. CP2405-303-024). Every patient gave their informed consent, and each procedure carried out in this study conformed with the declaration of Helsinki.

### Data collection & sample preparation

Complete patient information was acquired from the medical records, including demographic, clinical-pathological, and therapeutic approach information. Clinical data included age, gender, MM stage and the presence of a lytic bone lesion. Laboratory testing included a complete blood count (white blood cells, haemoglobin concentration, platelet count), kidney and liver function tests, serum calcium, β2-microglobin, protein electrophoresis, and immunofixation. All participants provided whole blood, bone marrow aspiration, bone marrow biopsy, and 24-hour urine samples. Prior to beginning any treatment, samples were taken from each participant under complete aseptic conditions. To extract miRNA, BM aspirate samples were collected into EDTA-treated tubes and centrifuged at 10,000 rpm for 10 min at room temperature. The pellet was then transferred directly to RNase-free tubes and kept at -80 °C until microRNA extraction. The BM biopsy specimen was fixed, decalcified, and embedded in paraffin for immunohistochemistry. While venous blood samples were centrifuged after coagulation, serum was extracted for chemical analysis.

### Bioinformatics analysis

The target genes of miR-15a-5p, miR-20a-5p, and miR-33b-3p were analyzed using miRNet and miRDB databases. We subsequently conducted Gene Ontology (GO) analysis on miR-15a-5p, miR-20a-5p, and miR-33b-3p target genes to identify the molecular functions and biological processes. Additionally, the signaling pathways for the putative enrichment of these target genes were succinctly examined using Kyoto Encyclopedia of Genes and Genomes (KEGG) enrichment analysis. Furthermore, the protein-protein interaction network of the EGR2 protein was retrieved from the STRING database. An interaction score of more than 0.7 was considered statistically significant.

### Determination of the baseline bone marrow miR-15a-5p, miR-20a-5p & miR-33b-3p expression levels by RT-qPCR

#### RNA extraction and cDNA Preparation

The MiRNeasy^®^ Mini Kit (Cat. No. 217004, Qiagen, Germany) was used to extract total RNA, including miRNA, from the BM aspirates of MM patients and control samples in accordance with the manufacturer’s instructions. A NanoDrop spectrophotometer (NanoDrop Maestrogen Inc., Taiwan) was used to assess the RNA samples in order to ascertain their purity and concentration. Only samples with an absorbance ratio between 1.8 and 2.0 at 260 nm to 280 nm were used. The TaqManTM MicroRNA Reverse Transcription Kit (Cat. No. 4366596, Thermo Fisher Scientific, Applied Biosystems, USA, California) was used to reverse transcribe the isolated RNA into cDNA in accordance with the manufacturer’s instructions. After that, it was kept at -20 °C until qPCR was done.

#### Quantitative real-time PCR (qPCR)

Real-time PCR quantification assays for the three different miRNAs examined in this work (miR-15a, miR-20a, and miR-33b) were carried out using Taqman Universal Master Mix II (Cat. No. 4440043, Thermo Fisher Scientific, Applied Biosystems, USA, California) in accordance with the manufacturer’s protocol. Thermo Fisher Scientific supplied the miRNA primers (miR-20a Cat. No. 4427975, ID 000580, miR-15a Cat. No. 4427975, ID 00389, miR-33b Cat. No. 4427975, ID 241007_mat), and the internal control used in this investigation was SnRNA U6 (Cat. No. 4427975, ID 001973). The reaction mixture was loaded into the real-time cycler (Applied Biosystems™ StepOne™ Real-Time PCR System). The thermal settings were as follows: an initial activation step at 95 °C for 15 min, followed by 40 cycles of 94 °C for 15 s, 55 °C for 30 s, and 70 °C for 30 s. The CT 2^(- ΔΔCT) equation was used to calculate relative miRNA expression, where ΔΔCt = [(Ct miR of interest - Ct SnRNA U6) MM patient] – [(Ct miR of interest - Ct SnRNA U6) control].

### EGR2 protein immunohistochemistry (IHC) assay

To evaluate the protein expression of EGR2 in bone marrow samples from MM patients and controls, IHC staining analysis was performed using the standard immunoperoxidase staining method. Simply, paraffin-embedded bone marrow slices were deparaffinized using of xylene for times, thereafter heated in sodium citrate (10 mM, pH 6), and then incubated at room temperature in 3% H_2_O_2_ for 20 min. Samples were incubated for 1 h at room temperature in a primary antibody (polyclonal rabbit IgG Anti-EGR2/Krox20 Antibody, Cat. No. YPA2386) diluted 1:100 in phosphate-buffered saline-2% bovine serum albumin (PBS-2%BSA), washed with PBS, and then incubated for 30 min at room temperature with an HRP-conjugated secondary antibody (EnVision™ FLEX /HRP (RTU), Dako, Cat. No. K8000, SM802) diluted 1:100 in PBS-2%BSA. The immunoreactivity score was calculated by combining staining intensity (0 was non-existent, 1 was weak, 2 was moderate, while 3 was strong) and staining proportion (0 was no staining, 1 was < 1% positive cells, 2 was 1–10% positive cells, 3 was 11–33% positive cells, 4 was 34–65% positive cells, while 5 was ≥ 66% positive cells). Only cases with moderate to strong staining in more than 10% of cells (proportion score > 2) were considered positive for the study.

### Statistical analysis

The IBM SPSS software (version 23.0; IBM Corp., Armonk, NY, USA) was used to edit, tabulate, and analyze the obtained data. For quantitative parametric data, the data was reported as mean ± standard error (SE), while for quantitative non-parametric data, the median and interquartile range were used. Qualitative data is presented using frequency and percentage. The chi square test and Fisher’s exact test will be used to examine qualitative data, while the Student’s t-test or Mann Whitney test will be used to analyze quantitative data. The area under the curve (AUC), sensitivity, and specificity of miR-15a-5p, miR-20a-5p and miR-33b-3p expression levels as diagnostic biomarkers for MM disease were determined using the Receiver Operating Characteristic curve (ROC curve). To assess the impact of miR-15a-5p, miR-20a-5p, and miR-33b-3p expression levels on patient outcomes, Kaplan-Meier survival tests with log-rank option and Cox regression analysis were used. Then the risk factors of MM were assessed using logistic regression analysis. Significance was determined when the *p*-value (2-sided only) was less than 0.05.

## Results

### Demographic and biochemical data of MM patients

The study comprised 40 adult patients with MM (23 males and 17 females; mean age 56.5 ± 1.35 years) and 20 control participants (12 men and 8 females; mean age 55.3 ± 2.36 years). Table 1 shows demographic and clinical information for both MM patients and controls. There was no significant difference in age or gender between the study’s controls and patients when they were matched in age and gender (*p*˃0.05). Table 1 shows a significant (*p* < 0.05) increase in serum urea, BM blood plasma cell percentage, AST, total proteins, albumin, calcium, and B2-microglobin, as well as a significant (*p* < 0.05) decrease in haemoglobin concentration and platelet count among MM patients compared to controls (Table 1).

**Table 1:** Clinicopathological characteristics of MM patients and controls

Group Variable	MM Patients (*n* = 40) (Mean ± SE)	Controls (*n* = 20) (Mean ± SE)	*p* Value
Age (yrs)	56.5 ± 1.3	55.3 ± 2.3	0.70
Gender (*n* (%))			
Male	23(57.5%)	12(60.0%)	0.58
Female	17(42.2%)	8(40.0%)	
HB (g/dL)	**9.5 ± 0.27**	**12.09 ± 0.31**	< 0.001**
WBCs (10^3/µl)	8.51 ± 0.81	5.90 ± 0.37	0.121
Platelet count (10^3/mm3)	**216.37 ± 15.35**	**289.7 ± 15.46**	0.026*
Urea (mg/dL)	50.74 ± 5.61	17.41 ± 1.16	0.005*
Creatinine (mg/dL)	2.02 ± 0.47	0.67 ± 0.10	0.166
ALT (U/L)	23.91 ± 3.76	11.50 ± 1.39	0.11
AST (U/L)	**24.53 ± 1.97**	**7.17 ± 1.13**	< 0.001**
Total proteins (g/dL)	**10.18 ± 0.34**	**6.93 ± 0.26**	< 0.001**
Albumin (g/dL)	**3.32 ± 0.09**	**4.20 ± 0.26**	< 0.001**
Calcium (mg/dL)	**10.57 ± 0.39**	**8.57 ± 0.97**	0.035*
B2-microglobin	**7.59 ± 0.79**	**1.90 ± 0.23**	0.002*
BM plasma cell %	**43.73 ± 7.041**	**1.5 ± 0.07**	< 0.001**
EGR2 intensity (*n* (%))		----------------	----------------
Weak	1 (2.5%)		
Moderate	30 (75.0%)		
Strong	9 (22.5%)		
ISS Staging (*n* (%))		----------------	----------------
I	8 (20.0%)		
II	19 (47.5%)		
III	13 (32.5%)		
Lytic bone lesion (*n* (%))		----------------	----------------
Absent	3 (7.5%)		
Present	37 (92.5%)		

*MM* Multiple myeloma, *HB* Hemoglobin, *WBCs* White Blood Cells, *ALT* Alanine transaminase, *AST* Aspartate aminotransferase, *BM* Bone marrow, *EGR2* Early growth response 2, *ISS* International Staging System

*Significant at *p*-value < 0.05, ** Highly significant at *p*-value < 0.001. Bold indicates statistical significance

### Analysis of miR-15a-5p, miR-20a-5p, and miR-33b-3p target genes

**Fig. 2:**
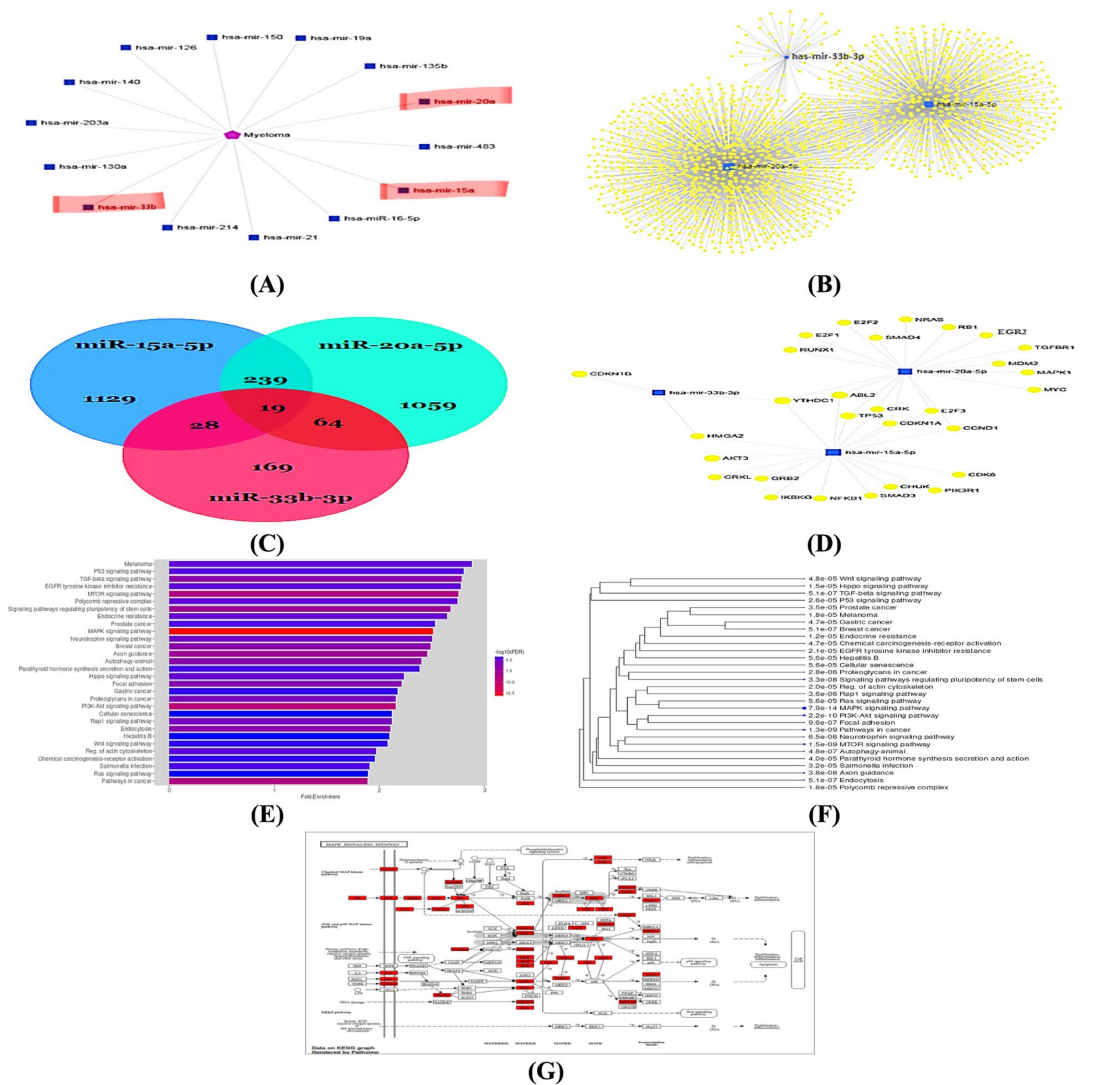
Bioinformatics analysis of miR-15a-5p, miR-20a-5p and miR-33b-3p: **A** MicroRNAs that play a role in MM pathogensis, **B** The miR-15a-5p, miR-20a-5p and miR-33b-3p-mRNA networks, **C** Venn diagram of miR-15a-5p, miR-20a-5p and miR-33b-3p, **D** The miR-15a-5p, miR-20a-5p and miR-33b-3p target genes that play a role in MM diseases, **E** GO functional enrichment analysis of miR-15a-5p, miR-20a-5p and miR-33b-3p all target genes. **F** A hierarchical clustering tree summarizes the correlation among significant pathways listed in miR-15a-5p, miR-20a-5p and miR-33b-3p target genes. Pathways with many shared genes are clustered together. Bigger dots indicate more significant *P*-values. **G** MAPK signaling pathway retreived from KEGG

Using miRNet and miRDB databases, we identified miR-15a-5p, miR-20a-5p, and miR-33b-3p as microRNAs involved in MM pathogenesis (Fig. 2A). Therefore, the target genes of miR-15a-5p, miR-20a-5p, and miR-33b-3p were retrieved. It was found that there were 1415 target genes for miR-15a-5p, 1381 target genes for miR-20a-5p, and 280 target genes for miR-33b-3p, as shown in supplementary Tables 1–3. It was discovered that there were 19 common target genes for the three microRNAs, while there were 239 target genes common between miR-15a-5p and miR-20a-5p, 28 target genes common between miR-15a-5p and miR-33b-3p, and 64 target genes common between 20a-5p and miR-33b-3p (Fig. 2B and C & supplementary Tables 4–7). The results of GO analysis are shown in Fig. 2E and F. The Biological process analysis showed that the target genes of miR-15a-5p, miR-20a-5p and miR-33b-3p were enriched in Wnt signaling pathway, TGF-β signaling pathway, p53 signaling pathway, Ras signaling pathway, MAPK signaling pathway, PI3K signaling pathway and pathways in cancer. Our results showed that the pathway with many shared genes is MAPK signaling pathway (Fig. 2E and F). The MAPK signaling pathway retreived from KEGG which was found to be envolved in the cell cycle (Fig. 2G).

### Expression levels of miR-15a-5p, miR-20a-5p and miR-33b-3p in the bone marrow of MM patients

**Fig. 3:**
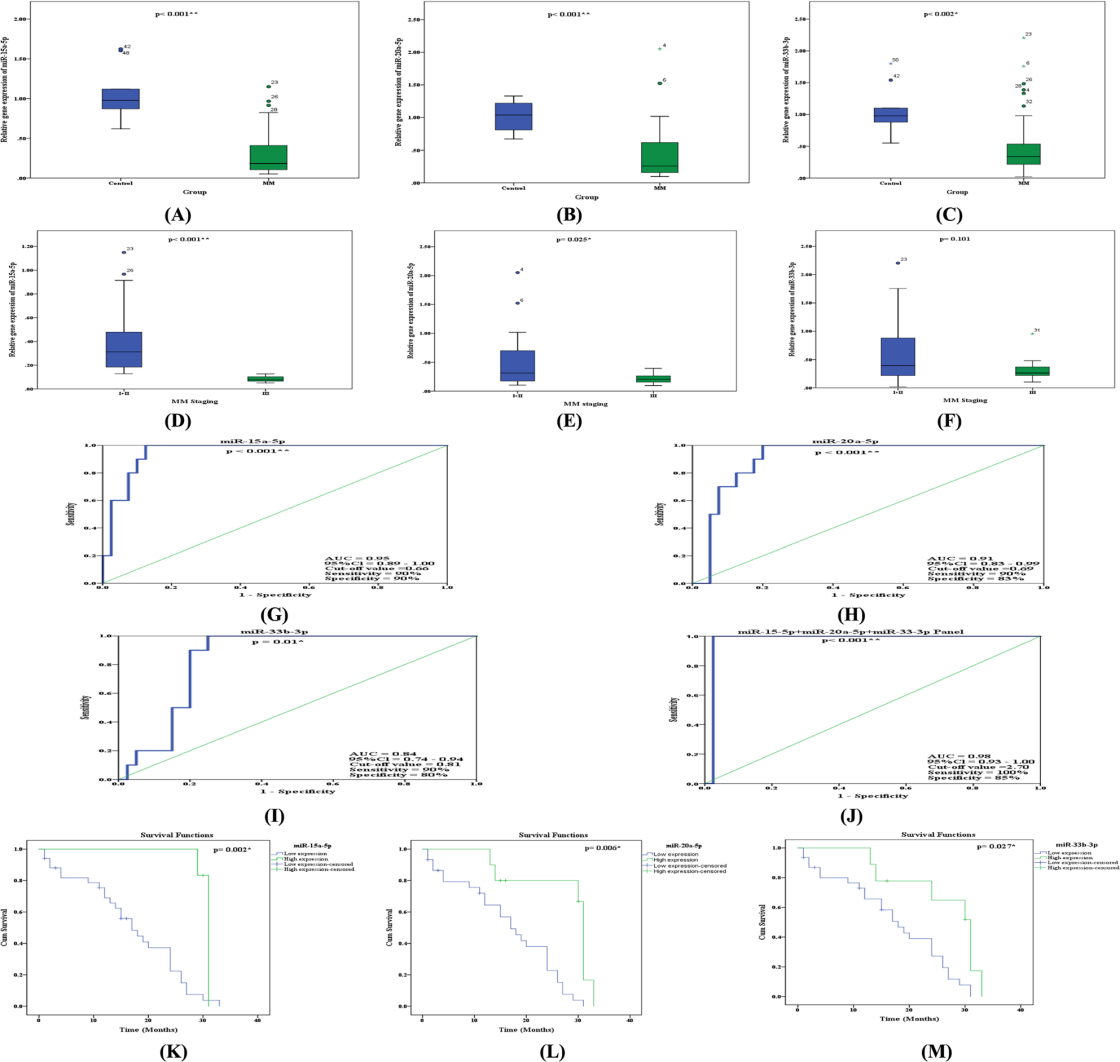
Expression levels of miR-15a-5p, miR-20a-5p and miR-33b-3p: **A-C** Relative gene expression of miR-15a-5p, miR-20a-5p and miR-33b-3p in MM patients and controls; respectively, **D-F** Relative gene expression of miR-15a-5p, miR-20a-5p and miR-33b-3p in MM staging; respectively, **G-J** ROC curve of miR-15a-5p, miR-20a-5p, miR-33b-3p and miR-15a-5p **+** miR-20a-5p **+** miR-33b-3p panel in MM patients versus controls; respectively, **K-M** Survival curve of miR-15a-5p, miR-20a-5p and miR-33b-3p in MM patients; respectively

The base line of bone marrow miR-15a-5p, miR-20a-5p, and miR-33b-3p expression levels in MM patients were significantly decreased (0.29 ± 0.04, *p* < 0.001; 0.42 ± 0.06, *p* < 0.001; 0.51 ± 0.07, *p* = 0.002 fold-change relative to controls) than controls (1.04 ± 0.11, 1.02 ± 0.07 and 1.05 ± 0.11 fold-change relative to controls; respectively), as illustrated in Fig. 3A and C. Additionally, our results revealed that the expression levels of miR-15a-5p and miR-20a-5p were significantly declined in stage III patients (0.08 ± 0.01, *p* < 0.001; 0.21 ± 0.02, *p* = 0.025 fold-change relative to controls) in comparison to stage I + II patients (0.39 ± 0.05, 0.52 ± 0.08 fold-change relative to controls; respectively) (Fig. 3D and E), but that miR-33b-3p was not significantly (*p* = 0.101) lower in stage III patients in comparison to stage I + II patients (Fig. 3F).

### ROC curves analysis of miR-15a-5p, miR-20a-5p and miR-33b-3p expression levels in the bone marrow of MM patients

The clinical use of miR-15a-5p, miR-20a-5p, and miR-33b-3p expression levels as indicators for the diagnosis of MM disease was examined using ROC curve analysis. Our findings showed that miR-15a-5p had 90% sensitivity and 90% specificity at a cut-off value of 0.66 and an AUC of 0.95 (95%CI = 0.897-1.00, *p* < 0.001) (Fig. 3G). The AUCs for miR-20a-5p and miR-33b-3p expression levels were 0.91 (95% CI = 0.829–0.991, *p* < 0.001), and 0.84 (95% CI = 0.736–0.949, *p* = 0.01), respectively. At cut-off values of 0.69 and 0.81, they showed 90% sensitivity and 90% specificity, respectively (Fig. 3H and I). Moreover, the miR-15a-5p + miR-20a-5p + miR-33b-3p panel ROC curve explored that to be more clinically impactful than single miRNA as it had AUC 0.98 (95% CI = 0.93–1.00, *p* < 0.001), 100% sensitivity and 85% specificity (Fig. 3J). By comparing the results from ROC curve analysis, it was discovered that the miR-15a-5p expression level and miR-15a-5p **+** miR-20a-5p **+** miR-33b-3p panel were the best diagnostic biomarkers to differentiate MM disease.

### Survival curve and Kaplan − Meier analysis of miR-15a-5p, miR-20a-5p and miR-33b-3p in the bone marrow of MM patients

The expression levels of miR-15a-5p, miR-20a-5p, and miR-33b-3p were examined for potential correlations with patient survival rates using Kaplan-Meier survival analysis with a log-rank test for OS. The mean (95% CI) for OS was 24.08 (21.58–29.88) months throughout the 32-month follow-up period for MM patients. Based on ROC curve data, patients were classified as having low (less than 0.66, 0.69 and 0.81 for miR-15a-5p, miR-20a-5p and miR-33b-3p; respectively) or high (larger than 0.66, 0.69 and 0.81 for miR-15a-5p, miR-20a-5p and miR-33b-3p; respectively) expression based on cut-off values obtained from ROC curves. According to our findings, patients who expressed less miR-15a-5p (*p* = 0.002), miR-20a-5p (*p* = 0.006), and miR-33b-3p (*p* = 0.027) had a worse prognosis and a shorter overall survival time; as shown in Fig. 3K and M.

### Logistic regression analysis

The risk factors of MM were assessed using logistic regression analysis. Table 2 indicates that miR-15a-5p, miR-20a-5p, and miR-33b-3p are risk factors for the development of MM (OR = 9.36, 2.56, 4.76; 95% CI 4.02–21.28, 1.28–5.32, 2.43–9.16; *p* < 0.001, 0.016, < 0.001; respectively); as shown in Table 2.

**Table 2:** Multivariate logistic regression analysis of of miR-15a-5p, miR-20a-5p and miR-33b-3p in MM patients

Factors	Multivariate analysis		
	OR	95% CI	*p*
Age (yrs)	1.23	0.46–3.05	0.42
Gender (*n*)	1.18	0.35–2.46	0.76
HB (g/dL)	1.06	0.39–2.65	0.63
WBCs (10^3/µl)	1.14	0.45–2.71	0.67
Platelet count (10^3/mm3)	1.01	0.65–1.27	0.84
Urea (mg/dL)	1.00	1.00–1.00	0.65
Creatinine (mg/dL)	0.63	0.42–1.02	0.07
ALT (U/L)	0.72	0.49–1.05	0.26
AST (U/L)	1.26	0.88–1.78	0.17
Total proteins (g/dL)	1.24	0.98–1.85	0.18
Albumin (g/dL)	0.61	0.31–1.12	0.22
Calcium (mg/dL)	1.17	0.91–1.52	0.62
B2-microglobin	1.05	0.62–1.73	0.79
BM plasma cell %	**1.93**	**0.98–3.32**	0.027*
EGR2 intensity (*n*)	0.83	0.12–1.33	0.38
ISS Staging (*n*)	1.17	0.55–2.38	0.47
Lytic bone lesion (*n*)	1.42	0.55–3.44	0.32
miR-15a-5p	**9.36**	**4.02–21.28**	< 0.001**
miR-20a-5p	**2.56**	**1.28–5.32**	0.016*
miR-33b-3p	**4.76**	**2.43–9.16**	< 0.001**

*MM* Multiple myeloma, *HB* Hemoglobin, *WBCs* White Blood Cells, *ALT* Alanine transaminase, *AST* Aspartate aminotransferase, *BM* Bone marrow, *EGR2* Early growth response 2, *ISS* International Staging System, *OR* Odds Ratio, *CI* Confidence interval

*Significant at *p*-value < 0.05, ** Highly significant at *p*-value < 0.001. Bold indicates statistical significance

### EGR2 protein immunohistochemistry (IHC) assay

**Fig. 4:**
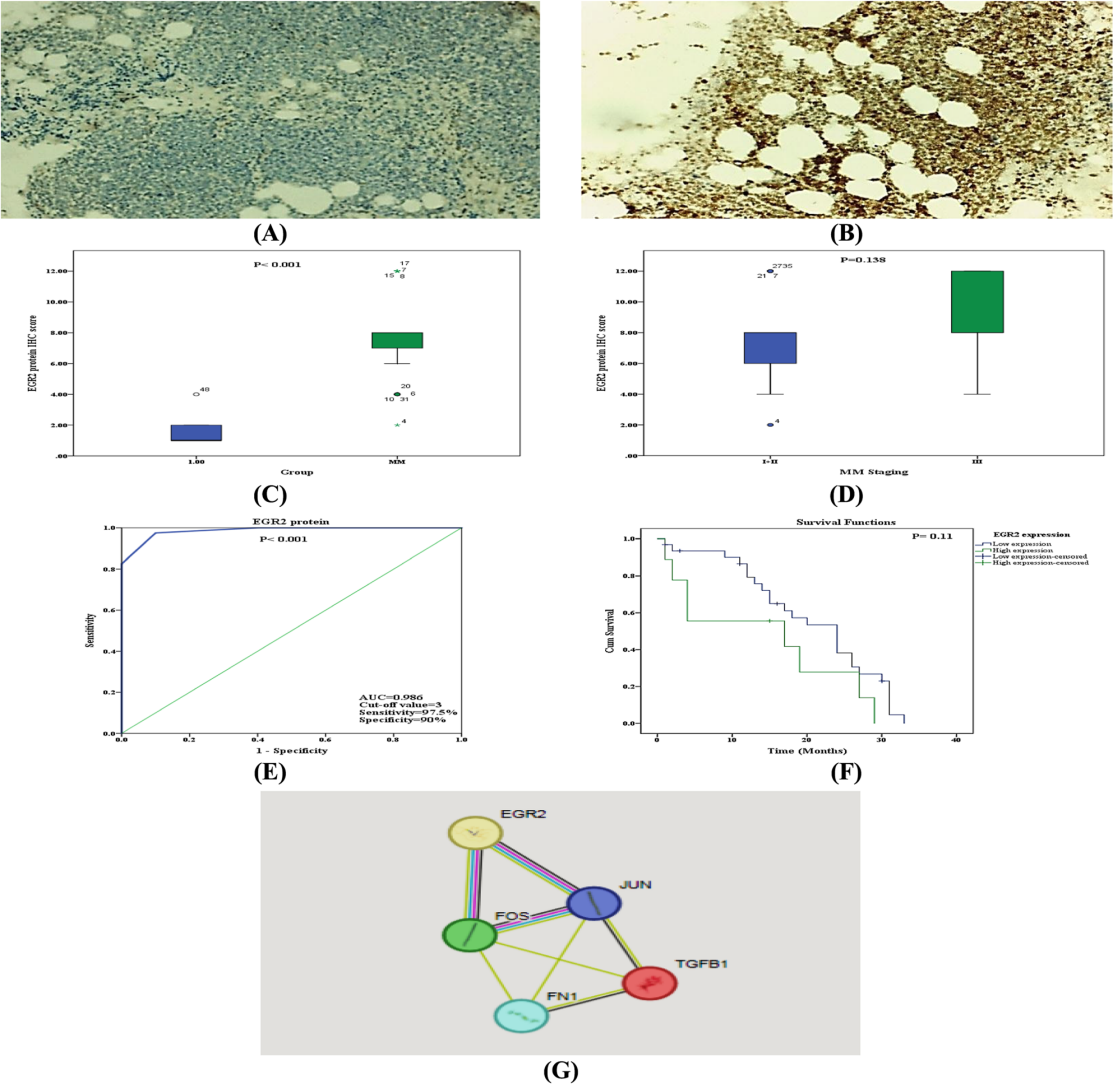
Expression level of EGR2 protein: **A **&** B** EGR2 protein IHC images in the bone marrow of control and MM patient; respectively, **C & D** EGR2 protein IHC score in MM patients versus controls and in MM staging; respectively, **E** ROC curve of EGR2 protein IHC score in MM patients versus controls; respectively, **F** Survival curve of EGR2 protein IHC score in MM patients, **G**The protein-protein interaction network of EGR2

By exploring the target genes of miR-15a-5p, miR-20a-5p, and miR-33b-3p that have a role in MM pathogenesis, it was found that EGR2 one of those genes (Fig. 2D). As consistent with our bioinformatics analysis (supplementary Table 2, highlighted part), the EGR2 protein is a putative target of miR-20a, therefore the expression of EGR2 protein was evaluated through immunohistochemistry. Our results showed that EGR2 protein expression in the bone marrow of MM patients was significantly upregulated in comparison to control (Fig. 4A and C), while it was not significantly upregulated in stage III patients in comparison to stage I + II patients (Fig. 4D). Furthermore, ROC curve analysis revealed that EGR2 expression was an excellent biomarker of AUC 0.986 (95%CI = 0.96-1.00), and at cut-off values 3 it had 97.5% sensitivity and 90% specificity (Fig. 4E). By using Kaplan-Meier survival analysis with a log-rank test for OS, patients were classified as having low (< 3) or high (> 3) expression based on ROC curve cut-off values. Although not statistically significant (*p* = 0.11), survival analysis provided additional evidence that higher EGR2 protein expression is a negative prognostic factor for MM patients (Fig. 4F). Also the protein-protein interaction (PPI) network of EGR2 protein was obtained using STRING database. Our results revealed that EGR2 connected with proteins TGFB1, JUN, FOS and FN1 which have roles in cancer proliferation and metastasis (Fig. 4G).

## Discussion

Numerous studies have found that miRNAs dysregulation contributes considerably to the development of malignant tumors [24]. Furthermore, it has been proven that miRNAs play a crucial role in MM pathogenesis by modulating the gene expression [25]. Our bioinformatics analysis identified miR-15a-5p, miR-20a-5p, and miR-33b-3p as microRNAs associated with MM. (Fig. 2A). Therefore, the current study aimed to conduct an integrated bioinformatics analysis of miR-15a-5p, miR-20a-5p, and miR-33b-3p to explore the molecular functions and biological processes in MM pathogenesis. Our GO function analysis of target genes of miR-15a-5p, miR-20a-5p, and miR-33b-3p revealed that the most significant pathway was MAPK signaling pathway (Fig. 2E and F), which play an important role in the cell cycle by retrieving the MAPK signaling pathway from KEGG (Fig. 2G). These findings showed that miR-15a-5p, miR-20a-5p, and miR-33b-3p may have roles in MM pathogensis through the cell cycle control. The MM pathogensis is complex and involves multiple aspects, including genetic anomalies [26], cytokine production [27], suppressive bone marrow microenvironment [28], and different signaling pathways [29]. Moreover, the MAPK signaling pathway is extensively researched and governs essential cellular functions, including differentiation, proliferation, apoptosis, and survival, in response to diverse micro-environmental stimuli in eukaryotes [30]. As consistent with our results, Lu et al. [31] reviewed that the MAPK signaling pathway is one from the patways that have crusial roles in MM pathogensis. But further studies are required to confirm the role of miR-15a-5p, miR-20a-5p, and miR-33b-3p in the development of MM through MAPK signaling pathway.

Regarding to miR-15a-5p and miR-33b-3p, our results revealed that their expression levels were significantly decreased in MM patients when compared to controls (Fig. 3A and C, respectively), while only miR-15a-5p expression level was significantly decreased in stage III patients when compared to stage I and II (Fig. 3D). Besides, both miR-15a-5p and miR-33b-3p were good diagnostic biomarkers could differentiate MM (Fig. 3G and I, respectively). Moreover, our results indicated that patients with lower expression levels of miR-15a-5p and miR-33b-3p had poorer prognosis and possessing shorter OS (Fig. 3K and M, respectively). It is well known that miR-15a is located on chromosome 13, a location that is frequently deleted in MM, and chromosome 13 deletion is typically associated with poor survival rates in MM patients [32, 33]. Also, Sun et al. [34] found that miR-15a was significantly downregulated in MM cell lines and primary MM tissues, which is consistent with our findings. Moreover, our findings were consistent with those of Zhang et al. [35], who reported that miR-15a-5p expression levels decreased in MM patients. MiR-33b is located in the 17p11.3 chromosomal region, specifically in the seventeenth intron of sterol regulatory element-binding protein-1 (SREBP). Practically, miR-33b was discovered to be downregulated in practically all forms of cancer. miR-33b inhibits protein-coding genes involved in DNA repair, proliferation, cell cycle, apoptosis, migration, and invasion [36]. Furthermore, our findings were agreed with Tian et al. [37] who revealed that miR-33b is a tumor suppressor that inhibited in MM cells. Additionally, Li et al. [38] discovered that patients with MM had clearly down-regulated miR-33b expression, and that patients with low expression of miR-33b had noticeably shorter OS.

For miR-20a-5p and its putative target gene EGR2, our results revealed that miR-20a-5p expression level was significantly decreased while EGR2 expression level was significantly increased in MM patients when compared to controls (Figs. 3B and 4A and B, respectively), while only miR-20a-5p expression level was significantly decreased in stage III patients when compared to stage I + II patients (Fig. 3E). Furthermore, both miR-20a-5p and EGR2 expression were good diagnostic biomarkers could differentiate MM (Figs. 3H and 4E, respectively). Moreover, our results revealed that patients with lower expression levels of miR-20a-5p and higher expression levels of EGR2 had poorer prognosis and possessing shorter OS (Figs. 3K and 4F, respectively). MiR-20a is a member of the miR-17-92 microRNA cluster, which is located on the 13q31.1 chromosome and is largely carcinogenic. It has been linked to several cancers as a tumor suppressor and a potential oncogene. However, its precise function in MM remains uncertain [39]. However, miR-20a functions as a tumor suppressor in other malignancies, such as hepatic and oral squamous cancer. These results imply that various cell types may have distinct miR-20a functions [17]. Our results were in agreement with Zhang et al. [35] who found both miR-15a-5p and miR-20a-5p expression levels decreased in MM patients. Also, Wang et al. [40] and Moura et al. [41] reported that miR-20a-5p expression level was decreased in MM patients. Our findings concurred with those of Xiao et al. [42], who discovered that EGR2 was significantly expressed in the tissues of bladder cancer which could not migrate, invade, or multiply when EGR2 was knocked down, while EGR2 overexpression was associated with a bad prognosis.

As diagnostic markers, our results revealed that miR-15a-5p expression level was the best miRNA used better than miR-20a-5p or miR-33b-3p could differentiate MM disease. As a prognostic tool, our results revealed that only miR-15a-5p and miR-20a-5p rather than miR-33b-3p and EGR2 protein were significantly increased in stage III MM patients in comparison to stage I + II patients (Figs. 3D and F and 4D), which indicated that miR-15a-5p and miR-20a-5p were good diagnostic and prognostic markers while miR-33b-3p and EGR2 protein were diagnostic rather than prognostic markers in MM patients. Our results were in agreement with Tavakoli et al. [15] and Dubaj et al. [43] who found that miR-15a-5p expression level was good diagnostic marker in MM patiens. Moreover, Li et al. [44] and Zhang et al. [45] reported that miR-15a-5p and miR-20a-5p are good prognostic markers could differentiate smouldering myeloma (SMM) and MM, respectively. But when we used miR-15a-5p + miR-20a-5p + miR-33b-3p panel as a diagnostic marker, it gave the best result with AUC 0.98, 100% sensitivity and 85% specificity (Fig. 3J). Till now there is no published data about miR-15a-5p + miR-20a-5p + miR-33b-3p panel.

This study had several limitations: (1) the retrospective design impeded comprehensive data collection from specific subjects; (2) the limited sample size reduced statistical power, and the study’s singular site may have influenced the outcomes; (3) the exclusively Egyptian cohort restricts ethnic diversity, necessitating cautious interpretation of the results; (4) the single-center design, unadjusted survival analysis, and the need for *in vitro/in vivo* functional validation of the miRNA-EGR2 axis.

## Conclusion

In summary, we found that the levels of miR-15a-5p, miR-20a-5p, and miR-33b-3p were decreased in MM patients versus controls and also served as indicators of low survival rates among MM patients. The combined diagnostic potential of the miR-15a-5p + miR-20a-5p + miR-33b-3p panel and EGR2, along with stage-specific drop in miR-15a/20a showed high diagnostic and prognostic accuracy and were associated with survival, suggesting utility for risk stratification. Our findings suggest that miR-15a-5p, miR-20a-5p, and miR-33b-3p may have roles in MM pathogenesis through MAPK signaling pathway and hence the cell cycle control.

## Supplementary Information

Supplementary Material 1.

Supplementary Material 2.

Supplementary Material 3.

Supplementary Material 4.

## Data Availability

The datasets used and/or analysed during the current study are available from the corresponding author on reasonable request.
