# Supplementary material for: Integrated analysis of miR-15a-5p, miR-20a-5p, and miR-33b-3p identifies EGR2-associated biomarkers in multiple myeloma
Source: BMC Cancer. 2026 Feb 12;26:298. doi: 10.1186/s12885-026-15610-5 (PMC12930744; doi:10.1186/s12885-026-15610-5)
Supplement: Supplementary file 1 — Supplementary Material 1. [file 12885_2026_15610_MOESM1_ESM.docx]

**Supplementary table (1):** Predicted targets for hsa-miR-15a-5p.

| **Target Detail** | **Target Rank** | **Target Score** | **miRNA Name** | **Gene Symbol** | **Gene Description** |
| --- | --- | --- | --- | --- | --- |
| [Details](https://mirdb.org/cgi-bin/target_detail.cgi?targetID=3253048) | 1 | 100 | hsa-miR-15a-5p | [PAPPA](http://www.ncbi.nlm.nih.gov/entrez/query.fcgi?db=gene&cmd=Retrieve&dopt=full_report&list_uids=5069) | pappalysin 1 |
| [Details](https://mirdb.org/cgi-bin/target_detail.cgi?targetID=3253354) | 2 | 100 | hsa-miR-15a-5p | [FASN](http://www.ncbi.nlm.nih.gov/entrez/query.fcgi?db=gene&cmd=Retrieve&dopt=full_report&list_uids=2194) | fatty acid synthase |
| [Details](https://mirdb.org/cgi-bin/target_detail.cgi?targetID=3253829) | 3 | 100 | hsa-miR-15a-5p | [UNC80](http://www.ncbi.nlm.nih.gov/entrez/query.fcgi?db=gene&cmd=Retrieve&dopt=full_report&list_uids=285175) | unc-80 homolog, NALCN channel complex subunit |
| [Details](https://mirdb.org/cgi-bin/target_detail.cgi?targetID=3253858) | 4 | 100 | hsa-miR-15a-5p | [FGF2](http://www.ncbi.nlm.nih.gov/entrez/query.fcgi?db=gene&cmd=Retrieve&dopt=full_report&list_uids=2247) | fibroblast growth factor 2 |
| [Details](https://mirdb.org/cgi-bin/target_detail.cgi?targetID=3253898) | 5 | 100 | hsa-miR-15a-5p | [TNRC6B](http://www.ncbi.nlm.nih.gov/entrez/query.fcgi?db=gene&cmd=Retrieve&dopt=full_report&list_uids=23112) | trinucleotide repeat containing 6B |
| [Details](https://mirdb.org/cgi-bin/target_detail.cgi?targetID=3253914) | 6 | 100 | hsa-miR-15a-5p | [PTPN4](http://www.ncbi.nlm.nih.gov/entrez/query.fcgi?db=gene&cmd=Retrieve&dopt=full_report&list_uids=5775) | protein tyrosine phosphatase, non-receptor type 4 |
| [Details](https://mirdb.org/cgi-bin/target_detail.cgi?targetID=3253945) | 7 | 100 | hsa-miR-15a-5p | [PHF19](http://www.ncbi.nlm.nih.gov/entrez/query.fcgi?db=gene&cmd=Retrieve&dopt=full_report&list_uids=26147) | PHD finger protein 19 |
| [Details](https://mirdb.org/cgi-bin/target_detail.cgi?targetID=3253959) | 8 | 100 | hsa-miR-15a-5p | [DESI1](http://www.ncbi.nlm.nih.gov/entrez/query.fcgi?db=gene&cmd=Retrieve&dopt=full_report&list_uids=27351) | desumoylating isopeptidase 1 |
| [Details](https://mirdb.org/cgi-bin/target_detail.cgi?targetID=3252779) | 9 | 99 | hsa-miR-15a-5p | [UBE2Q1](http://www.ncbi.nlm.nih.gov/entrez/query.fcgi?db=gene&cmd=Retrieve&dopt=full_report&list_uids=55585) | ubiquitin conjugating enzyme E2 Q1 |
| [Details](https://mirdb.org/cgi-bin/target_detail.cgi?targetID=3252820) | 10 | 99 | hsa-miR-15a-5p | [LSM11](http://www.ncbi.nlm.nih.gov/entrez/query.fcgi?db=gene&cmd=Retrieve&dopt=full_report&list_uids=134353) | LSM11, U7 small nuclear RNA associated |
| [Details](https://mirdb.org/cgi-bin/target_detail.cgi?targetID=3252827) | 11 | 99 | hsa-miR-15a-5p | [NECTIN1](http://www.ncbi.nlm.nih.gov/entrez/query.fcgi?db=gene&cmd=Retrieve&dopt=full_report&list_uids=5818) | nectin cell adhesion molecule 1 |
| [Details](https://mirdb.org/cgi-bin/target_detail.cgi?targetID=3252862) | 12 | 99 | hsa-miR-15a-5p | [GAREM1](http://www.ncbi.nlm.nih.gov/entrez/query.fcgi?db=gene&cmd=Retrieve&dopt=full_report&list_uids=64762) | GRB2 associated regulator of MAPK1 subtype 1 |
| [Details](https://mirdb.org/cgi-bin/target_detail.cgi?targetID=3252885) | 13 | 99 | hsa-miR-15a-5p | [ANKUB1](http://www.ncbi.nlm.nih.gov/entrez/query.fcgi?db=gene&cmd=Retrieve&dopt=full_report&list_uids=389161) | ankyrin repeat and ubiquitin domain containing 1 |
| [Details](https://mirdb.org/cgi-bin/target_detail.cgi?targetID=3252957) | 14 | 99 | hsa-miR-15a-5p | [FBXO21](http://www.ncbi.nlm.nih.gov/entrez/query.fcgi?db=gene&cmd=Retrieve&dopt=full_report&list_uids=23014) | F-box protein 21 |
| [Details](https://mirdb.org/cgi-bin/target_detail.cgi?targetID=3253009) | 15 | 99 | hsa-miR-15a-5p | [CCNE1](http://www.ncbi.nlm.nih.gov/entrez/query.fcgi?db=gene&cmd=Retrieve&dopt=full_report&list_uids=898) | cyclin E1 |
| [Details](https://mirdb.org/cgi-bin/target_detail.cgi?targetID=3253017) | 16 | 99 | hsa-miR-15a-5p | [ATG14](http://www.ncbi.nlm.nih.gov/entrez/query.fcgi?db=gene&cmd=Retrieve&dopt=full_report&list_uids=22863) | autophagy related 14 |
| [Details](https://mirdb.org/cgi-bin/target_detail.cgi?targetID=3253077) | 17 | 99 | hsa-miR-15a-5p | [LUZP1](http://www.ncbi.nlm.nih.gov/entrez/query.fcgi?db=gene&cmd=Retrieve&dopt=full_report&list_uids=7798) | leucine zipper protein 1 |
| [Details](https://mirdb.org/cgi-bin/target_detail.cgi?targetID=3253101) | 18 | 99 | hsa-miR-15a-5p | [SLC13A3](http://www.ncbi.nlm.nih.gov/entrez/query.fcgi?db=gene&cmd=Retrieve&dopt=full_report&list_uids=64849) | solute carrier family 13 member 3 |
| [Details](https://mirdb.org/cgi-bin/target_detail.cgi?targetID=3253132) | 19 | 99 | hsa-miR-15a-5p | [ARIH1](http://www.ncbi.nlm.nih.gov/entrez/query.fcgi?db=gene&cmd=Retrieve&dopt=full_report&list_uids=25820) | ariadne RBR E3 ubiquitin protein ligase 1 |
| [Details](https://mirdb.org/cgi-bin/target_detail.cgi?targetID=3253372) | 20 | 99 | hsa-miR-15a-5p | [MGAT4A](http://www.ncbi.nlm.nih.gov/entrez/query.fcgi?db=gene&cmd=Retrieve&dopt=full_report&list_uids=11320) | alpha-1,3-mannosyl-glycoprotein 4-beta-N-acetylglucosaminyltransferase A |
| [Details](https://mirdb.org/cgi-bin/target_detail.cgi?targetID=3253498) | 21 | 99 | hsa-miR-15a-5p | [EPHB2](http://www.ncbi.nlm.nih.gov/entrez/query.fcgi?db=gene&cmd=Retrieve&dopt=full_report&list_uids=2048) | EPH receptor B2 |
| [Details](https://mirdb.org/cgi-bin/target_detail.cgi?targetID=3253558) | 22 | 99 | hsa-miR-15a-5p | [BTRC](http://www.ncbi.nlm.nih.gov/entrez/query.fcgi?db=gene&cmd=Retrieve&dopt=full_report&list_uids=8945) | beta-transducin repeat containing E3 ubiquitin protein ligase |
| [Details](https://mirdb.org/cgi-bin/target_detail.cgi?targetID=3253612) | 23 | 99 | hsa-miR-15a-5p | [SPRYD3](http://www.ncbi.nlm.nih.gov/entrez/query.fcgi?db=gene&cmd=Retrieve&dopt=full_report&list_uids=84926) | SPRY domain containing 3 |
| [Details](https://mirdb.org/cgi-bin/target_detail.cgi?targetID=3253638) | 24 | 99 | hsa-miR-15a-5p | [ARL2](http://www.ncbi.nlm.nih.gov/entrez/query.fcgi?db=gene&cmd=Retrieve&dopt=full_report&list_uids=402) | ADP ribosylation factor like GTPase 2 |
| [Details](https://mirdb.org/cgi-bin/target_detail.cgi?targetID=3253641) | 25 | 99 | hsa-miR-15a-5p | [CASK](http://www.ncbi.nlm.nih.gov/entrez/query.fcgi?db=gene&cmd=Retrieve&dopt=full_report&list_uids=8573) | calcium/calmodulin dependent serine protein kinase |
| [Details](https://mirdb.org/cgi-bin/target_detail.cgi?targetID=3253656) | 26 | 99 | hsa-miR-15a-5p | [NUP50](http://www.ncbi.nlm.nih.gov/entrez/query.fcgi?db=gene&cmd=Retrieve&dopt=full_report&list_uids=10762) | nucleoporin 50 |
| [Details](https://mirdb.org/cgi-bin/target_detail.cgi?targetID=3253828) | 27 | 99 | hsa-miR-15a-5p | [DCLK1](http://www.ncbi.nlm.nih.gov/entrez/query.fcgi?db=gene&cmd=Retrieve&dopt=full_report&list_uids=9201) | doublecortin like kinase 1 |
| [Details](https://mirdb.org/cgi-bin/target_detail.cgi?targetID=3253999) | 28 | 99 | hsa-miR-15a-5p | [CYB561A3](http://www.ncbi.nlm.nih.gov/entrez/query.fcgi?db=gene&cmd=Retrieve&dopt=full_report&list_uids=220002) | cytochrome b561 family member A3 |
| [Details](https://mirdb.org/cgi-bin/target_detail.cgi?targetID=3254005) | 29 | 99 | hsa-miR-15a-5p | [ZBTB46](http://www.ncbi.nlm.nih.gov/entrez/query.fcgi?db=gene&cmd=Retrieve&dopt=full_report&list_uids=140685) | zinc finger and BTB domain containing 46 |
| [Details](https://mirdb.org/cgi-bin/target_detail.cgi?targetID=3254008) | 30 | 99 | hsa-miR-15a-5p | [FGF7](http://www.ncbi.nlm.nih.gov/entrez/query.fcgi?db=gene&cmd=Retrieve&dopt=full_report&list_uids=2252) | fibroblast growth factor 7 |
| [Details](https://mirdb.org/cgi-bin/target_detail.cgi?targetID=3252631) | 31 | 98 | hsa-miR-15a-5p | [RECK](http://www.ncbi.nlm.nih.gov/entrez/query.fcgi?db=gene&cmd=Retrieve&dopt=full_report&list_uids=8434) | reversion inducing cysteine rich protein with kazal motifs |
| [Details](https://mirdb.org/cgi-bin/target_detail.cgi?targetID=3252668) | 32 | 98 | hsa-miR-15a-5p | [PLAG1](http://www.ncbi.nlm.nih.gov/entrez/query.fcgi?db=gene&cmd=Retrieve&dopt=full_report&list_uids=5324) | PLAG1 zinc finger |
| [Details](https://mirdb.org/cgi-bin/target_detail.cgi?targetID=3252772) | 33 | 98 | hsa-miR-15a-5p | [AXIN2](http://www.ncbi.nlm.nih.gov/entrez/query.fcgi?db=gene&cmd=Retrieve&dopt=full_report&list_uids=8313) | axin 2 |
| [Details](https://mirdb.org/cgi-bin/target_detail.cgi?targetID=3252785) | 34 | 98 | hsa-miR-15a-5p | [GPR63](http://www.ncbi.nlm.nih.gov/entrez/query.fcgi?db=gene&cmd=Retrieve&dopt=full_report&list_uids=81491) | G protein-coupled receptor 63 |
| [Details](https://mirdb.org/cgi-bin/target_detail.cgi?targetID=3252822) | 35 | 98 | hsa-miR-15a-5p | [SYNJ1](http://www.ncbi.nlm.nih.gov/entrez/query.fcgi?db=gene&cmd=Retrieve&dopt=full_report&list_uids=8867) | synaptojanin 1 |
| [Details](https://mirdb.org/cgi-bin/target_detail.cgi?targetID=3252870) | 36 | 98 | hsa-miR-15a-5p | [ABL2](http://www.ncbi.nlm.nih.gov/entrez/query.fcgi?db=gene&cmd=Retrieve&dopt=full_report&list_uids=27) | ABL proto-oncogene 2, non-receptor tyrosine kinase |
| [Details](https://mirdb.org/cgi-bin/target_detail.cgi?targetID=3252920) | 37 | 98 | hsa-miR-15a-5p | [SCN8A](http://www.ncbi.nlm.nih.gov/entrez/query.fcgi?db=gene&cmd=Retrieve&dopt=full_report&list_uids=6334) | sodium voltage-gated channel alpha subunit 8 |
| [Details](https://mirdb.org/cgi-bin/target_detail.cgi?targetID=3252953) | 38 | 98 | hsa-miR-15a-5p | [CACNA1E](http://www.ncbi.nlm.nih.gov/entrez/query.fcgi?db=gene&cmd=Retrieve&dopt=full_report&list_uids=777) | calcium voltage-gated channel subunit alpha1 E |
| [Details](https://mirdb.org/cgi-bin/target_detail.cgi?targetID=3252990) | 39 | 98 | hsa-miR-15a-5p | [PISD](http://www.ncbi.nlm.nih.gov/entrez/query.fcgi?db=gene&cmd=Retrieve&dopt=full_report&list_uids=23761) | phosphatidylserine decarboxylase |
| [Details](https://mirdb.org/cgi-bin/target_detail.cgi?targetID=3253002) | 40 | 98 | hsa-miR-15a-5p | [KCNJ2](http://www.ncbi.nlm.nih.gov/entrez/query.fcgi?db=gene&cmd=Retrieve&dopt=full_report&list_uids=3759) | potassium voltage-gated channel subfamily J member 2 |
| [Details](https://mirdb.org/cgi-bin/target_detail.cgi?targetID=3253023) | 41 | 98 | hsa-miR-15a-5p | [C2orf42](http://www.ncbi.nlm.nih.gov/entrez/query.fcgi?db=gene&cmd=Retrieve&dopt=full_report&list_uids=54980) | chromosome 2 open reading frame 42 |
| [Details](https://mirdb.org/cgi-bin/target_detail.cgi?targetID=3253046) | 42 | 98 | hsa-miR-15a-5p | [UBE2V1](http://www.ncbi.nlm.nih.gov/entrez/query.fcgi?db=gene&cmd=Retrieve&dopt=full_report&list_uids=7335) | ubiquitin conjugating enzyme E2 V1 |
| [Details](https://mirdb.org/cgi-bin/target_detail.cgi?targetID=3253073) | 43 | 98 | hsa-miR-15a-5p | [KIF1B](http://www.ncbi.nlm.nih.gov/entrez/query.fcgi?db=gene&cmd=Retrieve&dopt=full_report&list_uids=23095) | kinesin family member 1B |
| [Details](https://mirdb.org/cgi-bin/target_detail.cgi?targetID=3253106) | 44 | 98 | hsa-miR-15a-5p | [SPRED1](http://www.ncbi.nlm.nih.gov/entrez/query.fcgi?db=gene&cmd=Retrieve&dopt=full_report&list_uids=161742) | sprouty related EVH1 domain containing 1 |
| [Details](https://mirdb.org/cgi-bin/target_detail.cgi?targetID=3253133) | 45 | 98 | hsa-miR-15a-5p | [SREK1](http://www.ncbi.nlm.nih.gov/entrez/query.fcgi?db=gene&cmd=Retrieve&dopt=full_report&list_uids=140890) | splicing regulatory glutamic acid and lysine rich protein 1 |
| [Details](https://mirdb.org/cgi-bin/target_detail.cgi?targetID=3253165) | 46 | 98 | hsa-miR-15a-5p | [TBL1XR1](http://www.ncbi.nlm.nih.gov/entrez/query.fcgi?db=gene&cmd=Retrieve&dopt=full_report&list_uids=79718) | transducin beta like 1 X-linked receptor 1 |
| [Details](https://mirdb.org/cgi-bin/target_detail.cgi?targetID=3253167) | 47 | 98 | hsa-miR-15a-5p | [MTMR3](http://www.ncbi.nlm.nih.gov/entrez/query.fcgi?db=gene&cmd=Retrieve&dopt=full_report&list_uids=8897) | myotubularin related protein 3 |
| [Details](https://mirdb.org/cgi-bin/target_detail.cgi?targetID=3253221) | 48 | 98 | hsa-miR-15a-5p | [TLK1](http://www.ncbi.nlm.nih.gov/entrez/query.fcgi?db=gene&cmd=Retrieve&dopt=full_report&list_uids=9874) | tousled like kinase 1 |
| [Details](https://mirdb.org/cgi-bin/target_detail.cgi?targetID=3253285) | 49 | 98 | hsa-miR-15a-5p | [SLC11A2](http://www.ncbi.nlm.nih.gov/entrez/query.fcgi?db=gene&cmd=Retrieve&dopt=full_report&list_uids=4891) | solute carrier family 11 member 2 |
| [Details](https://mirdb.org/cgi-bin/target_detail.cgi?targetID=3253288) | 50 | 98 | hsa-miR-15a-5p | [MOB3B](http://www.ncbi.nlm.nih.gov/entrez/query.fcgi?db=gene&cmd=Retrieve&dopt=full_report&list_uids=79817) | MOB kinase activator 3B |
| [Details](https://mirdb.org/cgi-bin/target_detail.cgi?targetID=3253290) | 51 | 98 | hsa-miR-15a-5p | [ZBTB44](http://www.ncbi.nlm.nih.gov/entrez/query.fcgi?db=gene&cmd=Retrieve&dopt=full_report&list_uids=29068) | zinc finger and BTB domain containing 44 |
| [Details](https://mirdb.org/cgi-bin/target_detail.cgi?targetID=3253521) | 52 | 98 | hsa-miR-15a-5p | [ANO3](http://www.ncbi.nlm.nih.gov/entrez/query.fcgi?db=gene&cmd=Retrieve&dopt=full_report&list_uids=63982) | anoctamin 3 |
| [Details](https://mirdb.org/cgi-bin/target_detail.cgi?targetID=3253545) | 53 | 98 | hsa-miR-15a-5p | [SLC9A6](http://www.ncbi.nlm.nih.gov/entrez/query.fcgi?db=gene&cmd=Retrieve&dopt=full_report&list_uids=10479) | solute carrier family 9 member A6 |
| [Details](https://mirdb.org/cgi-bin/target_detail.cgi?targetID=3253552) | 54 | 98 | hsa-miR-15a-5p | [APLN](http://www.ncbi.nlm.nih.gov/entrez/query.fcgi?db=gene&cmd=Retrieve&dopt=full_report&list_uids=8862) | apelin |
| [Details](https://mirdb.org/cgi-bin/target_detail.cgi?targetID=3253556) | 55 | 98 | hsa-miR-15a-5p | [AKT3](http://www.ncbi.nlm.nih.gov/entrez/query.fcgi?db=gene&cmd=Retrieve&dopt=full_report&list_uids=10000) | AKT serine/threonine kinase 3 |
| [Details](https://mirdb.org/cgi-bin/target_detail.cgi?targetID=3253559) | 56 | 98 | hsa-miR-15a-5p | [IPO7](http://www.ncbi.nlm.nih.gov/entrez/query.fcgi?db=gene&cmd=Retrieve&dopt=full_report&list_uids=10527) | importin 7 |
| [Details](https://mirdb.org/cgi-bin/target_detail.cgi?targetID=3253589) | 57 | 98 | hsa-miR-15a-5p | [RASGEF1B](http://www.ncbi.nlm.nih.gov/entrez/query.fcgi?db=gene&cmd=Retrieve&dopt=full_report&list_uids=153020) | RasGEF domain family member 1B |
| [Details](https://mirdb.org/cgi-bin/target_detail.cgi?targetID=3253608) | 58 | 98 | hsa-miR-15a-5p | [ATG9A](http://www.ncbi.nlm.nih.gov/entrez/query.fcgi?db=gene&cmd=Retrieve&dopt=full_report&list_uids=79065) | autophagy related 9A |
| [Details](https://mirdb.org/cgi-bin/target_detail.cgi?targetID=3253645) | 59 | 98 | hsa-miR-15a-5p | [CPEB2](http://www.ncbi.nlm.nih.gov/entrez/query.fcgi?db=gene&cmd=Retrieve&dopt=full_report&list_uids=132864) | cytoplasmic polyadenylation element binding protein 2 |
| [Details](https://mirdb.org/cgi-bin/target_detail.cgi?targetID=3253648) | 60 | 98 | hsa-miR-15a-5p | [AHCYL2](http://www.ncbi.nlm.nih.gov/entrez/query.fcgi?db=gene&cmd=Retrieve&dopt=full_report&list_uids=23382) | adenosylhomocysteinase like 2 |
| [Details](https://mirdb.org/cgi-bin/target_detail.cgi?targetID=3253710) | 61 | 98 | hsa-miR-15a-5p | [MYB](http://www.ncbi.nlm.nih.gov/entrez/query.fcgi?db=gene&cmd=Retrieve&dopt=full_report&list_uids=4602) | MYB proto-oncogene, transcription factor |
| [Details](https://mirdb.org/cgi-bin/target_detail.cgi?targetID=3253781) | 62 | 98 | hsa-miR-15a-5p | [CCND2](http://www.ncbi.nlm.nih.gov/entrez/query.fcgi?db=gene&cmd=Retrieve&dopt=full_report&list_uids=894) | cyclin D2 |
| [Details](https://mirdb.org/cgi-bin/target_detail.cgi?targetID=3253803) | 63 | 98 | hsa-miR-15a-5p | [KIF5C](http://www.ncbi.nlm.nih.gov/entrez/query.fcgi?db=gene&cmd=Retrieve&dopt=full_report&list_uids=3800) | kinesin family member 5C |
| [Details](https://mirdb.org/cgi-bin/target_detail.cgi?targetID=3253903) | 64 | 98 | hsa-miR-15a-5p | [UBN2](http://www.ncbi.nlm.nih.gov/entrez/query.fcgi?db=gene&cmd=Retrieve&dopt=full_report&list_uids=254048) | ubinuclein 2 |
| [Details](https://mirdb.org/cgi-bin/target_detail.cgi?targetID=3253931) | 65 | 98 | hsa-miR-15a-5p | [UBE4B](http://www.ncbi.nlm.nih.gov/entrez/query.fcgi?db=gene&cmd=Retrieve&dopt=full_report&list_uids=10277) | ubiquitination factor E4B |
| [Details](https://mirdb.org/cgi-bin/target_detail.cgi?targetID=3254031) | 66 | 98 | hsa-miR-15a-5p | [ARL3](http://www.ncbi.nlm.nih.gov/entrez/query.fcgi?db=gene&cmd=Retrieve&dopt=full_report&list_uids=403) | ADP ribosylation factor like GTPase 3 |
| [Details](https://mirdb.org/cgi-bin/target_detail.cgi?targetID=3252679) | 67 | 97 | hsa-miR-15a-5p | [MAMSTR](http://www.ncbi.nlm.nih.gov/entrez/query.fcgi?db=gene&cmd=Retrieve&dopt=full_report&list_uids=284358) | MEF2 activating motif and SAP domain containing transcriptional regulator |
| [Details](https://mirdb.org/cgi-bin/target_detail.cgi?targetID=3252784) | 68 | 97 | hsa-miR-15a-5p | [RNF144B](http://www.ncbi.nlm.nih.gov/entrez/query.fcgi?db=gene&cmd=Retrieve&dopt=full_report&list_uids=255488) | ring finger protein 144B |
| [Details](https://mirdb.org/cgi-bin/target_detail.cgi?targetID=3252877) | 69 | 97 | hsa-miR-15a-5p | [CDCA4](http://www.ncbi.nlm.nih.gov/entrez/query.fcgi?db=gene&cmd=Retrieve&dopt=full_report&list_uids=55038) | cell division cycle associated 4 |
| [Details](https://mirdb.org/cgi-bin/target_detail.cgi?targetID=3252915) | 70 | 97 | hsa-miR-15a-5p | [KDSR](http://www.ncbi.nlm.nih.gov/entrez/query.fcgi?db=gene&cmd=Retrieve&dopt=full_report&list_uids=2531) | 3-ketodihydrosphingosine reductase |
| [Details](https://mirdb.org/cgi-bin/target_detail.cgi?targetID=3252921) | 71 | 97 | hsa-miR-15a-5p | [KIF23](http://www.ncbi.nlm.nih.gov/entrez/query.fcgi?db=gene&cmd=Retrieve&dopt=full_report&list_uids=9493) | kinesin family member 23 |
| [Details](https://mirdb.org/cgi-bin/target_detail.cgi?targetID=3253068) | 72 | 97 | hsa-miR-15a-5p | [TFAP2A](http://www.ncbi.nlm.nih.gov/entrez/query.fcgi?db=gene&cmd=Retrieve&dopt=full_report&list_uids=7020) | transcription factor AP-2 alpha |
| [Details](https://mirdb.org/cgi-bin/target_detail.cgi?targetID=3253128) | 73 | 97 | hsa-miR-15a-5p | [PPM1E](http://www.ncbi.nlm.nih.gov/entrez/query.fcgi?db=gene&cmd=Retrieve&dopt=full_report&list_uids=22843) | protein phosphatase, Mg2+/Mn2+ dependent 1E |
| [Details](https://mirdb.org/cgi-bin/target_detail.cgi?targetID=3253196) | 74 | 97 | hsa-miR-15a-5p | [SHOC2](http://www.ncbi.nlm.nih.gov/entrez/query.fcgi?db=gene&cmd=Retrieve&dopt=full_report&list_uids=8036) | SHOC2, leucine rich repeat scaffold protein |
| [Details](https://mirdb.org/cgi-bin/target_detail.cgi?targetID=3253225) | 75 | 97 | hsa-miR-15a-5p | [HTR2A](http://www.ncbi.nlm.nih.gov/entrez/query.fcgi?db=gene&cmd=Retrieve&dopt=full_report&list_uids=3356) | 5-hydroxytryptamine receptor 2A |
| [Details](https://mirdb.org/cgi-bin/target_detail.cgi?targetID=3253295) | 76 | 97 | hsa-miR-15a-5p | [LURAP1L](http://www.ncbi.nlm.nih.gov/entrez/query.fcgi?db=gene&cmd=Retrieve&dopt=full_report&list_uids=286343) | leucine rich adaptor protein 1 like |
| [Details](https://mirdb.org/cgi-bin/target_detail.cgi?targetID=3253361) | 77 | 97 | hsa-miR-15a-5p | [DMPK](http://www.ncbi.nlm.nih.gov/entrez/query.fcgi?db=gene&cmd=Retrieve&dopt=full_report&list_uids=1760) | DM1 protein kinase |
| [Details](https://mirdb.org/cgi-bin/target_detail.cgi?targetID=3253380) | 78 | 97 | hsa-miR-15a-5p | [RAB11FIP2](http://www.ncbi.nlm.nih.gov/entrez/query.fcgi?db=gene&cmd=Retrieve&dopt=full_report&list_uids=22841) | RAB11 family interacting protein 2 |
| [Details](https://mirdb.org/cgi-bin/target_detail.cgi?targetID=3253446) | 79 | 97 | hsa-miR-15a-5p | [TMEM100](http://www.ncbi.nlm.nih.gov/entrez/query.fcgi?db=gene&cmd=Retrieve&dopt=full_report&list_uids=55273) | transmembrane protein 100 |
| [Details](https://mirdb.org/cgi-bin/target_detail.cgi?targetID=3253457) | 80 | 97 | hsa-miR-15a-5p | [RASSF8](http://www.ncbi.nlm.nih.gov/entrez/query.fcgi?db=gene&cmd=Retrieve&dopt=full_report&list_uids=11228) | Ras association domain family member 8 |
| [Details](https://mirdb.org/cgi-bin/target_detail.cgi?targetID=3253490) | 81 | 97 | hsa-miR-15a-5p | [FBXW7](http://www.ncbi.nlm.nih.gov/entrez/query.fcgi?db=gene&cmd=Retrieve&dopt=full_report&list_uids=55294) | F-box and WD repeat domain containing 7 |
| [Details](https://mirdb.org/cgi-bin/target_detail.cgi?targetID=3253555) | 82 | 97 | hsa-miR-15a-5p | [CCDC6](http://www.ncbi.nlm.nih.gov/entrez/query.fcgi?db=gene&cmd=Retrieve&dopt=full_report&list_uids=8030) | coiled-coil domain containing 6 |
| [Details](https://mirdb.org/cgi-bin/target_detail.cgi?targetID=3253601) | 83 | 97 | hsa-miR-15a-5p | [DNAJB4](http://www.ncbi.nlm.nih.gov/entrez/query.fcgi?db=gene&cmd=Retrieve&dopt=full_report&list_uids=11080) | DnaJ heat shock protein family (Hsp40) member B4 |
| [Details](https://mirdb.org/cgi-bin/target_detail.cgi?targetID=3253765) | 84 | 97 | hsa-miR-15a-5p | [GRM7](http://www.ncbi.nlm.nih.gov/entrez/query.fcgi?db=gene&cmd=Retrieve&dopt=full_report&list_uids=2917) | glutamate metabotropic receptor 7 |
| [Details](https://mirdb.org/cgi-bin/target_detail.cgi?targetID=3253866) | 85 | 97 | hsa-miR-15a-5p | [WEE1](http://www.ncbi.nlm.nih.gov/entrez/query.fcgi?db=gene&cmd=Retrieve&dopt=full_report&list_uids=7465) | WEE1 G2 checkpoint kinase |
| [Details](https://mirdb.org/cgi-bin/target_detail.cgi?targetID=3253877) | 86 | 97 | hsa-miR-15a-5p | [STOX2](http://www.ncbi.nlm.nih.gov/entrez/query.fcgi?db=gene&cmd=Retrieve&dopt=full_report&list_uids=56977) | storkhead box 2 |
| [Details](https://mirdb.org/cgi-bin/target_detail.cgi?targetID=3253927) | 87 | 97 | hsa-miR-15a-5p | [CYP26B1](http://www.ncbi.nlm.nih.gov/entrez/query.fcgi?db=gene&cmd=Retrieve&dopt=full_report&list_uids=56603) | cytochrome P450 family 26 subfamily B member 1 |
| [Details](https://mirdb.org/cgi-bin/target_detail.cgi?targetID=3252656) | 88 | 96 | hsa-miR-15a-5p | [N4BP1](http://www.ncbi.nlm.nih.gov/entrez/query.fcgi?db=gene&cmd=Retrieve&dopt=full_report&list_uids=9683) | NEDD4 binding protein 1 |
| [Details](https://mirdb.org/cgi-bin/target_detail.cgi?targetID=3252660) | 89 | 96 | hsa-miR-15a-5p | [PLPP1](http://www.ncbi.nlm.nih.gov/entrez/query.fcgi?db=gene&cmd=Retrieve&dopt=full_report&list_uids=8611) | phospholipid phosphatase 1 |
| [Details](https://mirdb.org/cgi-bin/target_detail.cgi?targetID=3252671) | 90 | 96 | hsa-miR-15a-5p | [ZNF691](http://www.ncbi.nlm.nih.gov/entrez/query.fcgi?db=gene&cmd=Retrieve&dopt=full_report&list_uids=51058) | zinc finger protein 691 |
| [Details](https://mirdb.org/cgi-bin/target_detail.cgi?targetID=3252674) | 91 | 96 | hsa-miR-15a-5p | [ZCCHC3](http://www.ncbi.nlm.nih.gov/entrez/query.fcgi?db=gene&cmd=Retrieve&dopt=full_report&list_uids=85364) | zinc finger CCHC-type containing 3 |
| [Details](https://mirdb.org/cgi-bin/target_detail.cgi?targetID=3252807) | 92 | 96 | hsa-miR-15a-5p | [ARMH4](http://www.ncbi.nlm.nih.gov/entrez/query.fcgi?db=gene&cmd=Retrieve&dopt=full_report&list_uids=145407) | armadillo-like helical domain containing 4 |
| [Details](https://mirdb.org/cgi-bin/target_detail.cgi?targetID=3252835) | 93 | 96 | hsa-miR-15a-5p | [SALL4](http://www.ncbi.nlm.nih.gov/entrez/query.fcgi?db=gene&cmd=Retrieve&dopt=full_report&list_uids=57167) | spalt like transcription factor 4 |
| [Details](https://mirdb.org/cgi-bin/target_detail.cgi?targetID=3252889) | 94 | 96 | hsa-miR-15a-5p | [TBPL1](http://www.ncbi.nlm.nih.gov/entrez/query.fcgi?db=gene&cmd=Retrieve&dopt=full_report&list_uids=9519) | TATA-box binding protein like 1 |
| [Details](https://mirdb.org/cgi-bin/target_detail.cgi?targetID=3252940) | 95 | 96 | hsa-miR-15a-5p | [CFAP45](http://www.ncbi.nlm.nih.gov/entrez/query.fcgi?db=gene&cmd=Retrieve&dopt=full_report&list_uids=25790) | cilia and flagella associated protein 45 |
| [Details](https://mirdb.org/cgi-bin/target_detail.cgi?targetID=3252950) | 96 | 96 | hsa-miR-15a-5p | [CNOT6L](http://www.ncbi.nlm.nih.gov/entrez/query.fcgi?db=gene&cmd=Retrieve&dopt=full_report&list_uids=246175) | CCR4-NOT transcription complex subunit 6 like |
| [Details](https://mirdb.org/cgi-bin/target_detail.cgi?targetID=3253013) | 97 | 96 | hsa-miR-15a-5p | [SEMA6D](http://www.ncbi.nlm.nih.gov/entrez/query.fcgi?db=gene&cmd=Retrieve&dopt=full_report&list_uids=80031) | semaphorin 6D |
| [Details](https://mirdb.org/cgi-bin/target_detail.cgi?targetID=3253035) | 98 | 96 | hsa-miR-15a-5p | [RFX3](http://www.ncbi.nlm.nih.gov/entrez/query.fcgi?db=gene&cmd=Retrieve&dopt=full_report&list_uids=5991) | regulatory factor X3 |
| [Details](https://mirdb.org/cgi-bin/target_detail.cgi?targetID=3253113) | 99 | 96 | hsa-miR-15a-5p | [PCMT1](http://www.ncbi.nlm.nih.gov/entrez/query.fcgi?db=gene&cmd=Retrieve&dopt=full_report&list_uids=5110) | protein-L-isoaspartate (D-aspartate) O-methyltransferase |
| [Details](https://mirdb.org/cgi-bin/target_detail.cgi?targetID=3253169) | 100 | 96 | hsa-miR-15a-5p | [EDA](http://www.ncbi.nlm.nih.gov/entrez/query.fcgi?db=gene&cmd=Retrieve&dopt=full_report&list_uids=1896) | ectodysplasin A |
| [Details](https://mirdb.org/cgi-bin/target_detail.cgi?targetID=3253241) | 101 | 96 | hsa-miR-15a-5p | [SEC24A](http://www.ncbi.nlm.nih.gov/entrez/query.fcgi?db=gene&cmd=Retrieve&dopt=full_report&list_uids=10802) | SEC24 homolog A, COPII coat complex component |
| [Details](https://mirdb.org/cgi-bin/target_detail.cgi?targetID=3253332) | 102 | 96 | hsa-miR-15a-5p | [KLHL2](http://www.ncbi.nlm.nih.gov/entrez/query.fcgi?db=gene&cmd=Retrieve&dopt=full_report&list_uids=11275) | kelch like family member 2 |
| [Details](https://mirdb.org/cgi-bin/target_detail.cgi?targetID=3253363) | 103 | 96 | hsa-miR-15a-5p | [PAFAH1B1](http://www.ncbi.nlm.nih.gov/entrez/query.fcgi?db=gene&cmd=Retrieve&dopt=full_report&list_uids=5048) | platelet activating factor acetylhydrolase 1b regulatory subunit 1 |
| [Details](https://mirdb.org/cgi-bin/target_detail.cgi?targetID=3253413) | 104 | 96 | hsa-miR-15a-5p | [PTPN3](http://www.ncbi.nlm.nih.gov/entrez/query.fcgi?db=gene&cmd=Retrieve&dopt=full_report&list_uids=5774) | protein tyrosine phosphatase, non-receptor type 3 |
| [Details](https://mirdb.org/cgi-bin/target_detail.cgi?targetID=3253509) | 105 | 96 | hsa-miR-15a-5p | [CASR](http://www.ncbi.nlm.nih.gov/entrez/query.fcgi?db=gene&cmd=Retrieve&dopt=full_report&list_uids=846) | calcium sensing receptor |
| [Details](https://mirdb.org/cgi-bin/target_detail.cgi?targetID=3253518) | 106 | 96 | hsa-miR-15a-5p | [MYLK](http://www.ncbi.nlm.nih.gov/entrez/query.fcgi?db=gene&cmd=Retrieve&dopt=full_report&list_uids=4638) | myosin light chain kinase |
| [Details](https://mirdb.org/cgi-bin/target_detail.cgi?targetID=3253700) | 107 | 96 | hsa-miR-15a-5p | [NAPG](http://www.ncbi.nlm.nih.gov/entrez/query.fcgi?db=gene&cmd=Retrieve&dopt=full_report&list_uids=8774) | NSF attachment protein gamma |
| [Details](https://mirdb.org/cgi-bin/target_detail.cgi?targetID=3253740) | 108 | 96 | hsa-miR-15a-5p | [ZNF367](http://www.ncbi.nlm.nih.gov/entrez/query.fcgi?db=gene&cmd=Retrieve&dopt=full_report&list_uids=195828) | zinc finger protein 367 |
| [Details](https://mirdb.org/cgi-bin/target_detail.cgi?targetID=3252739) | 109 | 95 | hsa-miR-15a-5p | [ZMAT3](http://www.ncbi.nlm.nih.gov/entrez/query.fcgi?db=gene&cmd=Retrieve&dopt=full_report&list_uids=64393) | zinc finger matrin-type 3 |
| [Details](https://mirdb.org/cgi-bin/target_detail.cgi?targetID=3252800) | 110 | 95 | hsa-miR-15a-5p | [UBFD1](http://www.ncbi.nlm.nih.gov/entrez/query.fcgi?db=gene&cmd=Retrieve&dopt=full_report&list_uids=56061) | ubiquitin family domain containing 1 |
| [Details](https://mirdb.org/cgi-bin/target_detail.cgi?targetID=3252821) | 111 | 95 | hsa-miR-15a-5p | [CHAC1](http://www.ncbi.nlm.nih.gov/entrez/query.fcgi?db=gene&cmd=Retrieve&dopt=full_report&list_uids=79094) | ChaC glutathione specific gamma-glutamylcyclotransferase 1 |
| [Details](https://mirdb.org/cgi-bin/target_detail.cgi?targetID=3252851) | 112 | 95 | hsa-miR-15a-5p | [HIPK2](http://www.ncbi.nlm.nih.gov/entrez/query.fcgi?db=gene&cmd=Retrieve&dopt=full_report&list_uids=28996) | homeodomain interacting protein kinase 2 |
| [Details](https://mirdb.org/cgi-bin/target_detail.cgi?targetID=3252857) | 113 | 95 | hsa-miR-15a-5p | [UBE4A](http://www.ncbi.nlm.nih.gov/entrez/query.fcgi?db=gene&cmd=Retrieve&dopt=full_report&list_uids=9354) | ubiquitination factor E4A |
| [Details](https://mirdb.org/cgi-bin/target_detail.cgi?targetID=3252898) | 114 | 95 | hsa-miR-15a-5p | [CEP55](http://www.ncbi.nlm.nih.gov/entrez/query.fcgi?db=gene&cmd=Retrieve&dopt=full_report&list_uids=55165) | centrosomal protein 55 |
| [Details](https://mirdb.org/cgi-bin/target_detail.cgi?targetID=3252904) | 115 | 95 | hsa-miR-15a-5p | [MKX](http://www.ncbi.nlm.nih.gov/entrez/query.fcgi?db=gene&cmd=Retrieve&dopt=full_report&list_uids=283078) | mohawk homeobox |
| [Details](https://mirdb.org/cgi-bin/target_detail.cgi?targetID=3253149) | 116 | 95 | hsa-miR-15a-5p | [CBX2](http://www.ncbi.nlm.nih.gov/entrez/query.fcgi?db=gene&cmd=Retrieve&dopt=full_report&list_uids=84733) | chromobox 2 |
| [Details](https://mirdb.org/cgi-bin/target_detail.cgi?targetID=3253156) | 117 | 95 | hsa-miR-15a-5p | [TNFSF13B](http://www.ncbi.nlm.nih.gov/entrez/query.fcgi?db=gene&cmd=Retrieve&dopt=full_report&list_uids=10673) | TNF superfamily member 13b |
| [Details](https://mirdb.org/cgi-bin/target_detail.cgi?targetID=3253243) | 118 | 95 | hsa-miR-15a-5p | [IPPK](http://www.ncbi.nlm.nih.gov/entrez/query.fcgi?db=gene&cmd=Retrieve&dopt=full_report&list_uids=64768) | inositol-pentakisphosphate 2-kinase |
| [Details](https://mirdb.org/cgi-bin/target_detail.cgi?targetID=3253388) | 119 | 95 | hsa-miR-15a-5p | [VEGFA](http://www.ncbi.nlm.nih.gov/entrez/query.fcgi?db=gene&cmd=Retrieve&dopt=full_report&list_uids=7422) | vascular endothelial growth factor A |
| [Details](https://mirdb.org/cgi-bin/target_detail.cgi?targetID=3253424) | 120 | 95 | hsa-miR-15a-5p | [SLC25A37](http://www.ncbi.nlm.nih.gov/entrez/query.fcgi?db=gene&cmd=Retrieve&dopt=full_report&list_uids=51312) | solute carrier family 25 member 37 |
| [Details](https://mirdb.org/cgi-bin/target_detail.cgi?targetID=3253445) | 121 | 95 | hsa-miR-15a-5p | [ZBTB34](http://www.ncbi.nlm.nih.gov/entrez/query.fcgi?db=gene&cmd=Retrieve&dopt=full_report&list_uids=403341) | zinc finger and BTB domain containing 34 |
| [Details](https://mirdb.org/cgi-bin/target_detail.cgi?targetID=3253497) | 122 | 95 | hsa-miR-15a-5p | [KIF5B](http://www.ncbi.nlm.nih.gov/entrez/query.fcgi?db=gene&cmd=Retrieve&dopt=full_report&list_uids=3799) | kinesin family member 5B |
| [Details](https://mirdb.org/cgi-bin/target_detail.cgi?targetID=3253531) | 123 | 95 | hsa-miR-15a-5p | [SETD3](http://www.ncbi.nlm.nih.gov/entrez/query.fcgi?db=gene&cmd=Retrieve&dopt=full_report&list_uids=84193) | SET domain containing 3, actin histidine methyltransferase |
| [Details](https://mirdb.org/cgi-bin/target_detail.cgi?targetID=3253570) | 124 | 95 | hsa-miR-15a-5p | [PAFAH1B2](http://www.ncbi.nlm.nih.gov/entrez/query.fcgi?db=gene&cmd=Retrieve&dopt=full_report&list_uids=5049) | platelet activating factor acetylhydrolase 1b catalytic subunit 2 |
| [Details](https://mirdb.org/cgi-bin/target_detail.cgi?targetID=3253580) | 125 | 95 | hsa-miR-15a-5p | [MAP2K1](http://www.ncbi.nlm.nih.gov/entrez/query.fcgi?db=gene&cmd=Retrieve&dopt=full_report&list_uids=5604) | mitogen-activated protein kinase kinase 1 |
| [Details](https://mirdb.org/cgi-bin/target_detail.cgi?targetID=3253685) | 126 | 95 | hsa-miR-15a-5p | [SMURF1](http://www.ncbi.nlm.nih.gov/entrez/query.fcgi?db=gene&cmd=Retrieve&dopt=full_report&list_uids=57154) | SMAD specific E3 ubiquitin protein ligase 1 |
| [Details](https://mirdb.org/cgi-bin/target_detail.cgi?targetID=3253722) | 127 | 95 | hsa-miR-15a-5p | [GABARAPL1](http://www.ncbi.nlm.nih.gov/entrez/query.fcgi?db=gene&cmd=Retrieve&dopt=full_report&list_uids=23710) | GABA type A receptor associated protein like 1 |
| [Details](https://mirdb.org/cgi-bin/target_detail.cgi?targetID=3253741) | 128 | 95 | hsa-miR-15a-5p | [USP25](http://www.ncbi.nlm.nih.gov/entrez/query.fcgi?db=gene&cmd=Retrieve&dopt=full_report&list_uids=29761) | ubiquitin specific peptidase 25 |
| [Details](https://mirdb.org/cgi-bin/target_detail.cgi?targetID=3253872) | 129 | 95 | hsa-miR-15a-5p | [STXBP3](http://www.ncbi.nlm.nih.gov/entrez/query.fcgi?db=gene&cmd=Retrieve&dopt=full_report&list_uids=6814) | syntaxin binding protein 3 |
| [Details](https://mirdb.org/cgi-bin/target_detail.cgi?targetID=3253893) | 130 | 95 | hsa-miR-15a-5p | [FGFR1](http://www.ncbi.nlm.nih.gov/entrez/query.fcgi?db=gene&cmd=Retrieve&dopt=full_report&list_uids=2260) | fibroblast growth factor receptor 1 |
| [Details](https://mirdb.org/cgi-bin/target_detail.cgi?targetID=3253919) | 131 | 95 | hsa-miR-15a-5p | [MYBL1](http://www.ncbi.nlm.nih.gov/entrez/query.fcgi?db=gene&cmd=Retrieve&dopt=full_report&list_uids=4603) | MYB proto-oncogene like 1 |
| [Details](https://mirdb.org/cgi-bin/target_detail.cgi?targetID=3253947) | 132 | 95 | hsa-miR-15a-5p | [GPATCH8](http://www.ncbi.nlm.nih.gov/entrez/query.fcgi?db=gene&cmd=Retrieve&dopt=full_report&list_uids=23131) | G-patch domain containing 8 |
| [Details](https://mirdb.org/cgi-bin/target_detail.cgi?targetID=3254000) | 133 | 95 | hsa-miR-15a-5p | [PLXNC1](http://www.ncbi.nlm.nih.gov/entrez/query.fcgi?db=gene&cmd=Retrieve&dopt=full_report&list_uids=10154) | plexin C1 |
| [Details](https://mirdb.org/cgi-bin/target_detail.cgi?targetID=3254030) | 134 | 95 | hsa-miR-15a-5p | [CBX4](http://www.ncbi.nlm.nih.gov/entrez/query.fcgi?db=gene&cmd=Retrieve&dopt=full_report&list_uids=8535) | chromobox 4 |
| [Details](https://mirdb.org/cgi-bin/target_detail.cgi?targetID=3252662) | 135 | 94 | hsa-miR-15a-5p | [JPH3](http://www.ncbi.nlm.nih.gov/entrez/query.fcgi?db=gene&cmd=Retrieve&dopt=full_report&list_uids=57338) | junctophilin 3 |
| [Details](https://mirdb.org/cgi-bin/target_detail.cgi?targetID=3252678) | 136 | 94 | hsa-miR-15a-5p | [USP42](http://www.ncbi.nlm.nih.gov/entrez/query.fcgi?db=gene&cmd=Retrieve&dopt=full_report&list_uids=84132) | ubiquitin specific peptidase 42 |
| [Details](https://mirdb.org/cgi-bin/target_detail.cgi?targetID=3252699) | 137 | 94 | hsa-miR-15a-5p | [STRADB](http://www.ncbi.nlm.nih.gov/entrez/query.fcgi?db=gene&cmd=Retrieve&dopt=full_report&list_uids=55437) | STE20 related adaptor beta |
| [Details](https://mirdb.org/cgi-bin/target_detail.cgi?targetID=3252717) | 138 | 94 | hsa-miR-15a-5p | [RNF217](http://www.ncbi.nlm.nih.gov/entrez/query.fcgi?db=gene&cmd=Retrieve&dopt=full_report&list_uids=154214) | ring finger protein 217 |
| [Details](https://mirdb.org/cgi-bin/target_detail.cgi?targetID=3252774) | 139 | 94 | hsa-miR-15a-5p | [LRRN3](http://www.ncbi.nlm.nih.gov/entrez/query.fcgi?db=gene&cmd=Retrieve&dopt=full_report&list_uids=54674) | leucine rich repeat neuronal 3 |
| [Details](https://mirdb.org/cgi-bin/target_detail.cgi?targetID=3252787) | 140 | 94 | hsa-miR-15a-5p | [STXBP5](http://www.ncbi.nlm.nih.gov/entrez/query.fcgi?db=gene&cmd=Retrieve&dopt=full_report&list_uids=134957) | syntaxin binding protein 5 |
| [Details](https://mirdb.org/cgi-bin/target_detail.cgi?targetID=3252803) | 141 | 94 | hsa-miR-15a-5p | [ZFHX4](http://www.ncbi.nlm.nih.gov/entrez/query.fcgi?db=gene&cmd=Retrieve&dopt=full_report&list_uids=79776) | zinc finger homeobox 4 |
| [Details](https://mirdb.org/cgi-bin/target_detail.cgi?targetID=3252876) | 142 | 94 | hsa-miR-15a-5p | [ZNF622](http://www.ncbi.nlm.nih.gov/entrez/query.fcgi?db=gene&cmd=Retrieve&dopt=full_report&list_uids=90441) | zinc finger protein 622 |
| [Details](https://mirdb.org/cgi-bin/target_detail.cgi?targetID=3252878) | 143 | 94 | hsa-miR-15a-5p | [PTPRR](http://www.ncbi.nlm.nih.gov/entrez/query.fcgi?db=gene&cmd=Retrieve&dopt=full_report&list_uids=5801) | protein tyrosine phosphatase, receptor type R |
| [Details](https://mirdb.org/cgi-bin/target_detail.cgi?targetID=3252963) | 144 | 94 | hsa-miR-15a-5p | [STK33](http://www.ncbi.nlm.nih.gov/entrez/query.fcgi?db=gene&cmd=Retrieve&dopt=full_report&list_uids=65975) | serine/threonine kinase 33 |
| [Details](https://mirdb.org/cgi-bin/target_detail.cgi?targetID=3252977) | 145 | 94 | hsa-miR-15a-5p | [ATXN2](http://www.ncbi.nlm.nih.gov/entrez/query.fcgi?db=gene&cmd=Retrieve&dopt=full_report&list_uids=6311) | ataxin 2 |
| [Details](https://mirdb.org/cgi-bin/target_detail.cgi?targetID=3253108) | 146 | 94 | hsa-miR-15a-5p | [OOEP](http://www.ncbi.nlm.nih.gov/entrez/query.fcgi?db=gene&cmd=Retrieve&dopt=full_report&list_uids=441161) | oocyte expressed protein |
| [Details](https://mirdb.org/cgi-bin/target_detail.cgi?targetID=3253234) | 147 | 94 | hsa-miR-15a-5p | [P3H2](http://www.ncbi.nlm.nih.gov/entrez/query.fcgi?db=gene&cmd=Retrieve&dopt=full_report&list_uids=55214) | prolyl 3-hydroxylase 2 |
| [Details](https://mirdb.org/cgi-bin/target_detail.cgi?targetID=3253255) | 148 | 94 | hsa-miR-15a-5p | [AGO4](http://www.ncbi.nlm.nih.gov/entrez/query.fcgi?db=gene&cmd=Retrieve&dopt=full_report&list_uids=192670) | argonaute RISC catalytic component 4 |
| [Details](https://mirdb.org/cgi-bin/target_detail.cgi?targetID=3253312) | 149 | 94 | hsa-miR-15a-5p | [ZBTB39](http://www.ncbi.nlm.nih.gov/entrez/query.fcgi?db=gene&cmd=Retrieve&dopt=full_report&list_uids=9880) | zinc finger and BTB domain containing 39 |
| [Details](https://mirdb.org/cgi-bin/target_detail.cgi?targetID=3253322) | 150 | 94 | hsa-miR-15a-5p | [SRPRA](http://www.ncbi.nlm.nih.gov/entrez/query.fcgi?db=gene&cmd=Retrieve&dopt=full_report&list_uids=6734) | SRP receptor subunit alpha |
| [Details](https://mirdb.org/cgi-bin/target_detail.cgi?targetID=3253416) | 151 | 94 | hsa-miR-15a-5p | [PLXNA4](http://www.ncbi.nlm.nih.gov/entrez/query.fcgi?db=gene&cmd=Retrieve&dopt=full_report&list_uids=91584) | plexin A4 |
| [Details](https://mirdb.org/cgi-bin/target_detail.cgi?targetID=3253455) | 152 | 94 | hsa-miR-15a-5p | [WNK3](http://www.ncbi.nlm.nih.gov/entrez/query.fcgi?db=gene&cmd=Retrieve&dopt=full_report&list_uids=65267) | WNK lysine deficient protein kinase 3 |
| [Details](https://mirdb.org/cgi-bin/target_detail.cgi?targetID=3253467) | 153 | 94 | hsa-miR-15a-5p | [PPP1R11](http://www.ncbi.nlm.nih.gov/entrez/query.fcgi?db=gene&cmd=Retrieve&dopt=full_report&list_uids=6992) | protein phosphatase 1 regulatory inhibitor subunit 11 |
| [Details](https://mirdb.org/cgi-bin/target_detail.cgi?targetID=3253513) | 154 | 94 | hsa-miR-15a-5p | [LATS1](http://www.ncbi.nlm.nih.gov/entrez/query.fcgi?db=gene&cmd=Retrieve&dopt=full_report&list_uids=9113) | large tumor suppressor kinase 1 |
| [Details](https://mirdb.org/cgi-bin/target_detail.cgi?targetID=3253516) | 155 | 94 | hsa-miR-15a-5p | [DDX3X](http://www.ncbi.nlm.nih.gov/entrez/query.fcgi?db=gene&cmd=Retrieve&dopt=full_report&list_uids=1654) | DEAD-box helicase 3 X-linked |
| [Details](https://mirdb.org/cgi-bin/target_detail.cgi?targetID=3253659) | 156 | 94 | hsa-miR-15a-5p | [LRIG2](http://www.ncbi.nlm.nih.gov/entrez/query.fcgi?db=gene&cmd=Retrieve&dopt=full_report&list_uids=9860) | leucine rich repeats and immunoglobulin like domains 2 |
| [Details](https://mirdb.org/cgi-bin/target_detail.cgi?targetID=3253684) | 157 | 94 | hsa-miR-15a-5p | [ARHGDIA](http://www.ncbi.nlm.nih.gov/entrez/query.fcgi?db=gene&cmd=Retrieve&dopt=full_report&list_uids=396) | Rho GDP dissociation inhibitor alpha |
| [Details](https://mirdb.org/cgi-bin/target_detail.cgi?targetID=3253770) | 158 | 94 | hsa-miR-15a-5p | [PPP2R1B](http://www.ncbi.nlm.nih.gov/entrez/query.fcgi?db=gene&cmd=Retrieve&dopt=full_report&list_uids=5519) | protein phosphatase 2 scaffold subunit Abeta |
| [Details](https://mirdb.org/cgi-bin/target_detail.cgi?targetID=3253815) | 159 | 94 | hsa-miR-15a-5p | [NAA25](http://www.ncbi.nlm.nih.gov/entrez/query.fcgi?db=gene&cmd=Retrieve&dopt=full_report&list_uids=80018) | N(alpha)-acetyltransferase 25, NatB auxiliary subunit |
| [Details](https://mirdb.org/cgi-bin/target_detail.cgi?targetID=3253904) | 160 | 94 | hsa-miR-15a-5p | [DCP1A](http://www.ncbi.nlm.nih.gov/entrez/query.fcgi?db=gene&cmd=Retrieve&dopt=full_report&list_uids=55802) | decapping mRNA 1A |
| [Details](https://mirdb.org/cgi-bin/target_detail.cgi?targetID=3253925) | 161 | 94 | hsa-miR-15a-5p | [CHEK1](http://www.ncbi.nlm.nih.gov/entrez/query.fcgi?db=gene&cmd=Retrieve&dopt=full_report&list_uids=1111) | checkpoint kinase 1 |
| [Details](https://mirdb.org/cgi-bin/target_detail.cgi?targetID=3253949) | 162 | 94 | hsa-miR-15a-5p | [OMG](http://www.ncbi.nlm.nih.gov/entrez/query.fcgi?db=gene&cmd=Retrieve&dopt=full_report&list_uids=4974) | oligodendrocyte myelin glycoprotein |
| [Details](https://mirdb.org/cgi-bin/target_detail.cgi?targetID=3254010) | 163 | 94 | hsa-miR-15a-5p | [GALNT13](http://www.ncbi.nlm.nih.gov/entrez/query.fcgi?db=gene&cmd=Retrieve&dopt=full_report&list_uids=114805) | polypeptide N-acetylgalactosaminyltransferase 13 |
| [Details](https://mirdb.org/cgi-bin/target_detail.cgi?targetID=3252633) | 164 | 93 | hsa-miR-15a-5p | [ACTR2](http://www.ncbi.nlm.nih.gov/entrez/query.fcgi?db=gene&cmd=Retrieve&dopt=full_report&list_uids=10097) | ARP2 actin related protein 2 homolog |
| [Details](https://mirdb.org/cgi-bin/target_detail.cgi?targetID=3252675) | 165 | 93 | hsa-miR-15a-5p | [GHR](http://www.ncbi.nlm.nih.gov/entrez/query.fcgi?db=gene&cmd=Retrieve&dopt=full_report&list_uids=2690) | growth hormone receptor |
| [Details](https://mirdb.org/cgi-bin/target_detail.cgi?targetID=3252726) | 166 | 93 | hsa-miR-15a-5p | [BTAF1](http://www.ncbi.nlm.nih.gov/entrez/query.fcgi?db=gene&cmd=Retrieve&dopt=full_report&list_uids=9044) | B-TFIID TATA-box binding protein associated factor 1 |
| [Details](https://mirdb.org/cgi-bin/target_detail.cgi?targetID=3252735) | 167 | 93 | hsa-miR-15a-5p | [AVL9](http://www.ncbi.nlm.nih.gov/entrez/query.fcgi?db=gene&cmd=Retrieve&dopt=full_report&list_uids=23080) | AVL9 cell migration associated |
| [Details](https://mirdb.org/cgi-bin/target_detail.cgi?targetID=3252759) | 168 | 93 | hsa-miR-15a-5p | [TRANK1](http://www.ncbi.nlm.nih.gov/entrez/query.fcgi?db=gene&cmd=Retrieve&dopt=full_report&list_uids=9881) | tetratricopeptide repeat and ankyrin repeat containing 1 |
| [Details](https://mirdb.org/cgi-bin/target_detail.cgi?targetID=3252769) | 169 | 93 | hsa-miR-15a-5p | [FBXL20](http://www.ncbi.nlm.nih.gov/entrez/query.fcgi?db=gene&cmd=Retrieve&dopt=full_report&list_uids=84961) | F-box and leucine rich repeat protein 20 |
| [Details](https://mirdb.org/cgi-bin/target_detail.cgi?targetID=3252799) | 170 | 93 | hsa-miR-15a-5p | [DIXDC1](http://www.ncbi.nlm.nih.gov/entrez/query.fcgi?db=gene&cmd=Retrieve&dopt=full_report&list_uids=85458) | DIX domain containing 1 |
| [Details](https://mirdb.org/cgi-bin/target_detail.cgi?targetID=3252847) | 171 | 93 | hsa-miR-15a-5p | [CCND1](http://www.ncbi.nlm.nih.gov/entrez/query.fcgi?db=gene&cmd=Retrieve&dopt=full_report&list_uids=595) | cyclin D1 |
| [Details](https://mirdb.org/cgi-bin/target_detail.cgi?targetID=3252880) | 172 | 93 | hsa-miR-15a-5p | [SEPT2](http://www.ncbi.nlm.nih.gov/entrez/query.fcgi?db=gene&cmd=Retrieve&dopt=full_report&list_uids=4735) | septin 2 |
| [Details](https://mirdb.org/cgi-bin/target_detail.cgi?targetID=3253005) | 173 | 93 | hsa-miR-15a-5p | [MFN2](http://www.ncbi.nlm.nih.gov/entrez/query.fcgi?db=gene&cmd=Retrieve&dopt=full_report&list_uids=9927) | mitofusin 2 |
| [Details](https://mirdb.org/cgi-bin/target_detail.cgi?targetID=3253016) | 174 | 93 | hsa-miR-15a-5p | [SIK1](http://www.ncbi.nlm.nih.gov/entrez/query.fcgi?db=gene&cmd=Retrieve&dopt=full_report&list_uids=150094) | salt inducible kinase 1 |
| [Details](https://mirdb.org/cgi-bin/target_detail.cgi?targetID=3253030) | 175 | 93 | hsa-miR-15a-5p | [E2F3](http://www.ncbi.nlm.nih.gov/entrez/query.fcgi?db=gene&cmd=Retrieve&dopt=full_report&list_uids=1871) | E2F transcription factor 3 |
| [Details](https://mirdb.org/cgi-bin/target_detail.cgi?targetID=3253052) | 176 | 93 | hsa-miR-15a-5p | [WNT3A](http://www.ncbi.nlm.nih.gov/entrez/query.fcgi?db=gene&cmd=Retrieve&dopt=full_report&list_uids=89780) | Wnt family member 3A |
| [Details](https://mirdb.org/cgi-bin/target_detail.cgi?targetID=3253097) | 177 | 93 | hsa-miR-15a-5p | [ZNRF2](http://www.ncbi.nlm.nih.gov/entrez/query.fcgi?db=gene&cmd=Retrieve&dopt=full_report&list_uids=223082) | zinc and ring finger 2 |
| [Details](https://mirdb.org/cgi-bin/target_detail.cgi?targetID=3253111) | 178 | 93 | hsa-miR-15a-5p | [SOCS6](http://www.ncbi.nlm.nih.gov/entrez/query.fcgi?db=gene&cmd=Retrieve&dopt=full_report&list_uids=9306) | suppressor of cytokine signaling 6 |
| [Details](https://mirdb.org/cgi-bin/target_detail.cgi?targetID=3253153) | 179 | 93 | hsa-miR-15a-5p | [HSPA4L](http://www.ncbi.nlm.nih.gov/entrez/query.fcgi?db=gene&cmd=Retrieve&dopt=full_report&list_uids=22824) | heat shock protein family A (Hsp70) member 4 like |
| [Details](https://mirdb.org/cgi-bin/target_detail.cgi?targetID=3253157) | 180 | 93 | hsa-miR-15a-5p | [SEL1L3](http://www.ncbi.nlm.nih.gov/entrez/query.fcgi?db=gene&cmd=Retrieve&dopt=full_report&list_uids=23231) | SEL1L family member 3 |
| [Details](https://mirdb.org/cgi-bin/target_detail.cgi?targetID=3253352) | 181 | 93 | hsa-miR-15a-5p | [FOXK1](http://www.ncbi.nlm.nih.gov/entrez/query.fcgi?db=gene&cmd=Retrieve&dopt=full_report&list_uids=221937) | forkhead box K1 |
| [Details](https://mirdb.org/cgi-bin/target_detail.cgi?targetID=3253402) | 182 | 93 | hsa-miR-15a-5p | [SYT3](http://www.ncbi.nlm.nih.gov/entrez/query.fcgi?db=gene&cmd=Retrieve&dopt=full_report&list_uids=84258) | synaptotagmin 3 |
| [Details](https://mirdb.org/cgi-bin/target_detail.cgi?targetID=3253423) | 183 | 93 | hsa-miR-15a-5p | [HSPG2](http://www.ncbi.nlm.nih.gov/entrez/query.fcgi?db=gene&cmd=Retrieve&dopt=full_report&list_uids=3339) | heparan sulfate proteoglycan 2 |
| [Details](https://mirdb.org/cgi-bin/target_detail.cgi?targetID=3253426) | 184 | 93 | hsa-miR-15a-5p | [CD2AP](http://www.ncbi.nlm.nih.gov/entrez/query.fcgi?db=gene&cmd=Retrieve&dopt=full_report&list_uids=23607) | CD2 associated protein |
| [Details](https://mirdb.org/cgi-bin/target_detail.cgi?targetID=3253483) | 185 | 93 | hsa-miR-15a-5p | [SUCO](http://www.ncbi.nlm.nih.gov/entrez/query.fcgi?db=gene&cmd=Retrieve&dopt=full_report&list_uids=51430) | SUN domain containing ossification factor |
| [Details](https://mirdb.org/cgi-bin/target_detail.cgi?targetID=3253499) | 186 | 93 | hsa-miR-15a-5p | [MYO5A](http://www.ncbi.nlm.nih.gov/entrez/query.fcgi?db=gene&cmd=Retrieve&dopt=full_report&list_uids=4644) | myosin VA |
| [Details](https://mirdb.org/cgi-bin/target_detail.cgi?targetID=3253623) | 187 | 93 | hsa-miR-15a-5p | [AMOTL1](http://www.ncbi.nlm.nih.gov/entrez/query.fcgi?db=gene&cmd=Retrieve&dopt=full_report&list_uids=154810) | angiomotin like 1 |
| [Details](https://mirdb.org/cgi-bin/target_detail.cgi?targetID=3253729) | 188 | 93 | hsa-miR-15a-5p | [KANK1](http://www.ncbi.nlm.nih.gov/entrez/query.fcgi?db=gene&cmd=Retrieve&dopt=full_report&list_uids=23189) | KN motif and ankyrin repeat domains 1 |
| [Details](https://mirdb.org/cgi-bin/target_detail.cgi?targetID=3253767) | 189 | 93 | hsa-miR-15a-5p | [RBPJ](http://www.ncbi.nlm.nih.gov/entrez/query.fcgi?db=gene&cmd=Retrieve&dopt=full_report&list_uids=3516) | recombination signal binding protein for immunoglobulin kappa J region |
| [Details](https://mirdb.org/cgi-bin/target_detail.cgi?targetID=3253856) | 190 | 93 | hsa-miR-15a-5p | [SYDE2](http://www.ncbi.nlm.nih.gov/entrez/query.fcgi?db=gene&cmd=Retrieve&dopt=full_report&list_uids=84144) | synapse defective Rho GTPase homolog 2 |
| [Details](https://mirdb.org/cgi-bin/target_detail.cgi?targetID=3253860) | 191 | 93 | hsa-miR-15a-5p | [TMEM245](http://www.ncbi.nlm.nih.gov/entrez/query.fcgi?db=gene&cmd=Retrieve&dopt=full_report&list_uids=23731) | transmembrane protein 245 |
| [Details](https://mirdb.org/cgi-bin/target_detail.cgi?targetID=3253888) | 192 | 93 | hsa-miR-15a-5p | [RPS6KA3](http://www.ncbi.nlm.nih.gov/entrez/query.fcgi?db=gene&cmd=Retrieve&dopt=full_report&list_uids=6197) | ribosomal protein S6 kinase A3 |
| [Details](https://mirdb.org/cgi-bin/target_detail.cgi?targetID=3254040) | 193 | 93 | hsa-miR-15a-5p | [VPS33B](http://www.ncbi.nlm.nih.gov/entrez/query.fcgi?db=gene&cmd=Retrieve&dopt=full_report&list_uids=26276) | VPS33B, late endosome and lysosome associated |
| [Details](https://mirdb.org/cgi-bin/target_detail.cgi?targetID=3254044) | 194 | 93 | hsa-miR-15a-5p | [KIF21A](http://www.ncbi.nlm.nih.gov/entrez/query.fcgi?db=gene&cmd=Retrieve&dopt=full_report&list_uids=55605) | kinesin family member 21A |
| [Details](https://mirdb.org/cgi-bin/target_detail.cgi?targetID=3252685) | 195 | 92 | hsa-miR-15a-5p | [ZMYM2](http://www.ncbi.nlm.nih.gov/entrez/query.fcgi?db=gene&cmd=Retrieve&dopt=full_report&list_uids=7750) | zinc finger MYM-type containing 2 |
| [Details](https://mirdb.org/cgi-bin/target_detail.cgi?targetID=3252687) | 196 | 92 | hsa-miR-15a-5p | [SUMO3](http://www.ncbi.nlm.nih.gov/entrez/query.fcgi?db=gene&cmd=Retrieve&dopt=full_report&list_uids=6612) | small ubiquitin-like modifier 3 |
| [Details](https://mirdb.org/cgi-bin/target_detail.cgi?targetID=3252691) | 197 | 92 | hsa-miR-15a-5p | [CACUL1](http://www.ncbi.nlm.nih.gov/entrez/query.fcgi?db=gene&cmd=Retrieve&dopt=full_report&list_uids=143384) | CDK2 associated cullin domain 1 |
| [Details](https://mirdb.org/cgi-bin/target_detail.cgi?targetID=3252750) | 198 | 92 | hsa-miR-15a-5p | [PIAS2](http://www.ncbi.nlm.nih.gov/entrez/query.fcgi?db=gene&cmd=Retrieve&dopt=full_report&list_uids=9063) | protein inhibitor of activated STAT 2 |
| [Details](https://mirdb.org/cgi-bin/target_detail.cgi?targetID=3252798) | 199 | 92 | hsa-miR-15a-5p | [ACVR2A](http://www.ncbi.nlm.nih.gov/entrez/query.fcgi?db=gene&cmd=Retrieve&dopt=full_report&list_uids=92) | activin A receptor type 2A |
| [Details](https://mirdb.org/cgi-bin/target_detail.cgi?targetID=3252855) | 200 | 92 | hsa-miR-15a-5p | [TBP](http://www.ncbi.nlm.nih.gov/entrez/query.fcgi?db=gene&cmd=Retrieve&dopt=full_report&list_uids=6908) | TATA-box binding protein |
| [Details](https://mirdb.org/cgi-bin/target_detail.cgi?targetID=3252968) | 201 | 92 | hsa-miR-15a-5p | [SLIT2](http://www.ncbi.nlm.nih.gov/entrez/query.fcgi?db=gene&cmd=Retrieve&dopt=full_report&list_uids=9353) | slit guidance ligand 2 |
| [Details](https://mirdb.org/cgi-bin/target_detail.cgi?targetID=3252974) | 202 | 92 | hsa-miR-15a-5p | [XPO7](http://www.ncbi.nlm.nih.gov/entrez/query.fcgi?db=gene&cmd=Retrieve&dopt=full_report&list_uids=23039) | exportin 7 |
| [Details](https://mirdb.org/cgi-bin/target_detail.cgi?targetID=3253049) | 203 | 92 | hsa-miR-15a-5p | [SMAD7](http://www.ncbi.nlm.nih.gov/entrez/query.fcgi?db=gene&cmd=Retrieve&dopt=full_report&list_uids=4092) | SMAD family member 7 |
| [Details](https://mirdb.org/cgi-bin/target_detail.cgi?targetID=3253069) | 204 | 92 | hsa-miR-15a-5p | [YTHDC1](http://www.ncbi.nlm.nih.gov/entrez/query.fcgi?db=gene&cmd=Retrieve&dopt=full_report&list_uids=91746) | YTH domain containing 1 |
| [Details](https://mirdb.org/cgi-bin/target_detail.cgi?targetID=3253082) | 205 | 92 | hsa-miR-15a-5p | [CHD2](http://www.ncbi.nlm.nih.gov/entrez/query.fcgi?db=gene&cmd=Retrieve&dopt=full_report&list_uids=1106) | chromodomain helicase DNA binding protein 2 |
| [Details](https://mirdb.org/cgi-bin/target_detail.cgi?targetID=3253141) | 206 | 92 | hsa-miR-15a-5p | [SLC12A2](http://www.ncbi.nlm.nih.gov/entrez/query.fcgi?db=gene&cmd=Retrieve&dopt=full_report&list_uids=6558) | solute carrier family 12 member 2 |
| [Details](https://mirdb.org/cgi-bin/target_detail.cgi?targetID=3253166) | 207 | 92 | hsa-miR-15a-5p | [COP1](http://www.ncbi.nlm.nih.gov/entrez/query.fcgi?db=gene&cmd=Retrieve&dopt=full_report&list_uids=64326) | COP1, E3 ubiquitin ligase |
| [Details](https://mirdb.org/cgi-bin/target_detail.cgi?targetID=3253188) | 208 | 92 | hsa-miR-15a-5p | [ADAMTS3](http://www.ncbi.nlm.nih.gov/entrez/query.fcgi?db=gene&cmd=Retrieve&dopt=full_report&list_uids=9508) | ADAM metallopeptidase with thrombospondin type 1 motif 3 |
| [Details](https://mirdb.org/cgi-bin/target_detail.cgi?targetID=3253309) | 209 | 92 | hsa-miR-15a-5p | [KCNK10](http://www.ncbi.nlm.nih.gov/entrez/query.fcgi?db=gene&cmd=Retrieve&dopt=full_report&list_uids=54207) | potassium two pore domain channel subfamily K member 10 |
| [Details](https://mirdb.org/cgi-bin/target_detail.cgi?targetID=3253319) | 210 | 92 | hsa-miR-15a-5p | [ASH1L](http://www.ncbi.nlm.nih.gov/entrez/query.fcgi?db=gene&cmd=Retrieve&dopt=full_report&list_uids=55870) | ASH1 like histone lysine methyltransferase |
| [Details](https://mirdb.org/cgi-bin/target_detail.cgi?targetID=3253344) | 211 | 92 | hsa-miR-15a-5p | [RAD23B](http://www.ncbi.nlm.nih.gov/entrez/query.fcgi?db=gene&cmd=Retrieve&dopt=full_report&list_uids=5887) | RAD23 homolog B, nucleotide excision repair protein |
| [Details](https://mirdb.org/cgi-bin/target_detail.cgi?targetID=3253345) | 212 | 92 | hsa-miR-15a-5p | [SIRT4](http://www.ncbi.nlm.nih.gov/entrez/query.fcgi?db=gene&cmd=Retrieve&dopt=full_report&list_uids=23409) | sirtuin 4 |
| [Details](https://mirdb.org/cgi-bin/target_detail.cgi?targetID=3253394) | 213 | 92 | hsa-miR-15a-5p | [TMEM178B](http://www.ncbi.nlm.nih.gov/entrez/query.fcgi?db=gene&cmd=Retrieve&dopt=full_report&list_uids=100507421) | transmembrane protein 178B |
| [Details](https://mirdb.org/cgi-bin/target_detail.cgi?targetID=3253397) | 214 | 92 | hsa-miR-15a-5p | [RNF10](http://www.ncbi.nlm.nih.gov/entrez/query.fcgi?db=gene&cmd=Retrieve&dopt=full_report&list_uids=9921) | ring finger protein 10 |
| [Details](https://mirdb.org/cgi-bin/target_detail.cgi?targetID=3253466) | 215 | 92 | hsa-miR-15a-5p | [IFT74](http://www.ncbi.nlm.nih.gov/entrez/query.fcgi?db=gene&cmd=Retrieve&dopt=full_report&list_uids=80173) | intraflagellar transport 74 |
| [Details](https://mirdb.org/cgi-bin/target_detail.cgi?targetID=3253536) | 216 | 92 | hsa-miR-15a-5p | [PTH](http://www.ncbi.nlm.nih.gov/entrez/query.fcgi?db=gene&cmd=Retrieve&dopt=full_report&list_uids=5741) | parathyroid hormone |
| [Details](https://mirdb.org/cgi-bin/target_detail.cgi?targetID=3253575) | 217 | 92 | hsa-miR-15a-5p | [G2E3](http://www.ncbi.nlm.nih.gov/entrez/query.fcgi?db=gene&cmd=Retrieve&dopt=full_report&list_uids=55632) | G2/M-phase specific E3 ubiquitin protein ligase |
| [Details](https://mirdb.org/cgi-bin/target_detail.cgi?targetID=3253593) | 218 | 92 | hsa-miR-15a-5p | [ATXN7L2](http://www.ncbi.nlm.nih.gov/entrez/query.fcgi?db=gene&cmd=Retrieve&dopt=full_report&list_uids=127002) | ataxin 7 like 2 |
| [Details](https://mirdb.org/cgi-bin/target_detail.cgi?targetID=3253597) | 219 | 92 | hsa-miR-15a-5p | [LITAF](http://www.ncbi.nlm.nih.gov/entrez/query.fcgi?db=gene&cmd=Retrieve&dopt=full_report&list_uids=9516) | lipopolysaccharide induced TNF factor |
| [Details](https://mirdb.org/cgi-bin/target_detail.cgi?targetID=3253640) | 220 | 92 | hsa-miR-15a-5p | [KRTAP11-1](http://www.ncbi.nlm.nih.gov/entrez/query.fcgi?db=gene&cmd=Retrieve&dopt=full_report&list_uids=337880) | keratin associated protein 11-1 |
| [Details](https://mirdb.org/cgi-bin/target_detail.cgi?targetID=3253655) | 221 | 92 | hsa-miR-15a-5p | [TRABD2B](http://www.ncbi.nlm.nih.gov/entrez/query.fcgi?db=gene&cmd=Retrieve&dopt=full_report&list_uids=388630) | TraB domain containing 2B |
| [Details](https://mirdb.org/cgi-bin/target_detail.cgi?targetID=3253665) | 222 | 92 | hsa-miR-15a-5p | [ZNF449](http://www.ncbi.nlm.nih.gov/entrez/query.fcgi?db=gene&cmd=Retrieve&dopt=full_report&list_uids=203523) | zinc finger protein 449 |
| [Details](https://mirdb.org/cgi-bin/target_detail.cgi?targetID=3253688) | 223 | 92 | hsa-miR-15a-5p | [RETREG2](http://www.ncbi.nlm.nih.gov/entrez/query.fcgi?db=gene&cmd=Retrieve&dopt=full_report&list_uids=79137) | reticulophagy regulator family member 2 |
| [Details](https://mirdb.org/cgi-bin/target_detail.cgi?targetID=3253733) | 224 | 92 | hsa-miR-15a-5p | [DLL1](http://www.ncbi.nlm.nih.gov/entrez/query.fcgi?db=gene&cmd=Retrieve&dopt=full_report&list_uids=28514) | delta like canonical Notch ligand 1 |
| [Details](https://mirdb.org/cgi-bin/target_detail.cgi?targetID=3253749) | 225 | 92 | hsa-miR-15a-5p | [NHLRC2](http://www.ncbi.nlm.nih.gov/entrez/query.fcgi?db=gene&cmd=Retrieve&dopt=full_report&list_uids=374354) | NHL repeat containing 2 |
| [Details](https://mirdb.org/cgi-bin/target_detail.cgi?targetID=3253753) | 226 | 92 | hsa-miR-15a-5p | [SLC20A2](http://www.ncbi.nlm.nih.gov/entrez/query.fcgi?db=gene&cmd=Retrieve&dopt=full_report&list_uids=6575) | solute carrier family 20 member 2 |
| [Details](https://mirdb.org/cgi-bin/target_detail.cgi?targetID=3253820) | 227 | 92 | hsa-miR-15a-5p | [LAMP3](http://www.ncbi.nlm.nih.gov/entrez/query.fcgi?db=gene&cmd=Retrieve&dopt=full_report&list_uids=27074) | lysosomal associated membrane protein 3 |
| [Details](https://mirdb.org/cgi-bin/target_detail.cgi?targetID=3253837) | 228 | 92 | hsa-miR-15a-5p | [ELL](http://www.ncbi.nlm.nih.gov/entrez/query.fcgi?db=gene&cmd=Retrieve&dopt=full_report&list_uids=8178) | elongation factor for RNA polymerase II |
| [Details](https://mirdb.org/cgi-bin/target_detail.cgi?targetID=3253845) | 229 | 92 | hsa-miR-15a-5p | [TMEM183A](http://www.ncbi.nlm.nih.gov/entrez/query.fcgi?db=gene&cmd=Retrieve&dopt=full_report&list_uids=92703) | transmembrane protein 183A |
| [Details](https://mirdb.org/cgi-bin/target_detail.cgi?targetID=3253883) | 230 | 92 | hsa-miR-15a-5p | [CDC25A](http://www.ncbi.nlm.nih.gov/entrez/query.fcgi?db=gene&cmd=Retrieve&dopt=full_report&list_uids=993) | cell division cycle 25A |
| [Details](https://mirdb.org/cgi-bin/target_detail.cgi?targetID=3253892) | 231 | 92 | hsa-miR-15a-5p | [MEOX2](http://www.ncbi.nlm.nih.gov/entrez/query.fcgi?db=gene&cmd=Retrieve&dopt=full_report&list_uids=4223) | mesenchyme homeobox 2 |
| [Details](https://mirdb.org/cgi-bin/target_detail.cgi?targetID=3253946) | 232 | 92 | hsa-miR-15a-5p | [CPEB3](http://www.ncbi.nlm.nih.gov/entrez/query.fcgi?db=gene&cmd=Retrieve&dopt=full_report&list_uids=22849) | cytoplasmic polyadenylation element binding protein 3 |
| [Details](https://mirdb.org/cgi-bin/target_detail.cgi?targetID=3253970) | 233 | 92 | hsa-miR-15a-5p | [MEX3C](http://www.ncbi.nlm.nih.gov/entrez/query.fcgi?db=gene&cmd=Retrieve&dopt=full_report&list_uids=51320) | mex-3 RNA binding family member C |
| [Details](https://mirdb.org/cgi-bin/target_detail.cgi?targetID=3253977) | 234 | 92 | hsa-miR-15a-5p | [PCDH17](http://www.ncbi.nlm.nih.gov/entrez/query.fcgi?db=gene&cmd=Retrieve&dopt=full_report&list_uids=27253) | protocadherin 17 |
| [Details](https://mirdb.org/cgi-bin/target_detail.cgi?targetID=3252704) | 235 | 91 | hsa-miR-15a-5p | [CDK5R1](http://www.ncbi.nlm.nih.gov/entrez/query.fcgi?db=gene&cmd=Retrieve&dopt=full_report&list_uids=8851) | cyclin dependent kinase 5 regulatory subunit 1 |
| [Details](https://mirdb.org/cgi-bin/target_detail.cgi?targetID=3252771) | 236 | 91 | hsa-miR-15a-5p | [ATXN7L1](http://www.ncbi.nlm.nih.gov/entrez/query.fcgi?db=gene&cmd=Retrieve&dopt=full_report&list_uids=222255) | ataxin 7 like 1 |
| [Details](https://mirdb.org/cgi-bin/target_detail.cgi?targetID=3253063) | 237 | 91 | hsa-miR-15a-5p | [SALL1](http://www.ncbi.nlm.nih.gov/entrez/query.fcgi?db=gene&cmd=Retrieve&dopt=full_report&list_uids=6299) | spalt like transcription factor 1 |
| [Details](https://mirdb.org/cgi-bin/target_detail.cgi?targetID=3253134) | 238 | 91 | hsa-miR-15a-5p | [CC2D1B](http://www.ncbi.nlm.nih.gov/entrez/query.fcgi?db=gene&cmd=Retrieve&dopt=full_report&list_uids=200014) | coiled-coil and C2 domain containing 1B |
| [Details](https://mirdb.org/cgi-bin/target_detail.cgi?targetID=3253142) | 239 | 91 | hsa-miR-15a-5p | [INSR](http://www.ncbi.nlm.nih.gov/entrez/query.fcgi?db=gene&cmd=Retrieve&dopt=full_report&list_uids=3643) | insulin receptor |
| [Details](https://mirdb.org/cgi-bin/target_detail.cgi?targetID=3253152) | 240 | 91 | hsa-miR-15a-5p | [POU2F1](http://www.ncbi.nlm.nih.gov/entrez/query.fcgi?db=gene&cmd=Retrieve&dopt=full_report&list_uids=5451) | POU class 2 homeobox 1 |
| [Details](https://mirdb.org/cgi-bin/target_detail.cgi?targetID=3253155) | 241 | 91 | hsa-miR-15a-5p | [TMCC1](http://www.ncbi.nlm.nih.gov/entrez/query.fcgi?db=gene&cmd=Retrieve&dopt=full_report&list_uids=23023) | transmembrane and coiled-coil domain family 1 |
| [Details](https://mirdb.org/cgi-bin/target_detail.cgi?targetID=3253181) | 242 | 91 | hsa-miR-15a-5p | [ZNRF3](http://www.ncbi.nlm.nih.gov/entrez/query.fcgi?db=gene&cmd=Retrieve&dopt=full_report&list_uids=84133) | zinc and ring finger 3 |
| [Details](https://mirdb.org/cgi-bin/target_detail.cgi?targetID=3253201) | 243 | 91 | hsa-miR-15a-5p | [CLOCK](http://www.ncbi.nlm.nih.gov/entrez/query.fcgi?db=gene&cmd=Retrieve&dopt=full_report&list_uids=9575) | clock circadian regulator |
| [Details](https://mirdb.org/cgi-bin/target_detail.cgi?targetID=3253204) | 244 | 91 | hsa-miR-15a-5p | [KRTAP4-6](http://www.ncbi.nlm.nih.gov/entrez/query.fcgi?db=gene&cmd=Retrieve&dopt=full_report&list_uids=81871) | keratin associated protein 4-6 |
| [Details](https://mirdb.org/cgi-bin/target_detail.cgi?targetID=3253215) | 245 | 91 | hsa-miR-15a-5p | [CDC42SE2](http://www.ncbi.nlm.nih.gov/entrez/query.fcgi?db=gene&cmd=Retrieve&dopt=full_report&list_uids=56990) | CDC42 small effector 2 |
| [Details](https://mirdb.org/cgi-bin/target_detail.cgi?targetID=3253314) | 246 | 91 | hsa-miR-15a-5p | [SIK1B](http://www.ncbi.nlm.nih.gov/entrez/query.fcgi?db=gene&cmd=Retrieve&dopt=full_report&list_uids=102724428) | salt inducible kinase 1B (putative) |
| [Details](https://mirdb.org/cgi-bin/target_detail.cgi?targetID=3253343) | 247 | 91 | hsa-miR-15a-5p | [ZFHX3](http://www.ncbi.nlm.nih.gov/entrez/query.fcgi?db=gene&cmd=Retrieve&dopt=full_report&list_uids=463) | zinc finger homeobox 3 |
| [Details](https://mirdb.org/cgi-bin/target_detail.cgi?targetID=3253429) | 248 | 91 | hsa-miR-15a-5p | [PPM1A](http://www.ncbi.nlm.nih.gov/entrez/query.fcgi?db=gene&cmd=Retrieve&dopt=full_report&list_uids=5494) | protein phosphatase, Mg2+/Mn2+ dependent 1A |
| [Details](https://mirdb.org/cgi-bin/target_detail.cgi?targetID=3253435) | 249 | 91 | hsa-miR-15a-5p | [CCNT1](http://www.ncbi.nlm.nih.gov/entrez/query.fcgi?db=gene&cmd=Retrieve&dopt=full_report&list_uids=904) | cyclin T1 |
| [Details](https://mirdb.org/cgi-bin/target_detail.cgi?targetID=3253456) | 250 | 91 | hsa-miR-15a-5p | [NOS1](http://www.ncbi.nlm.nih.gov/entrez/query.fcgi?db=gene&cmd=Retrieve&dopt=full_report&list_uids=4842) | nitric oxide synthase 1 |
| [Details](https://mirdb.org/cgi-bin/target_detail.cgi?targetID=3253468) | 251 | 91 | hsa-miR-15a-5p | [LRRK1](http://www.ncbi.nlm.nih.gov/entrez/query.fcgi?db=gene&cmd=Retrieve&dopt=full_report&list_uids=79705) | leucine rich repeat kinase 1 |
| [Details](https://mirdb.org/cgi-bin/target_detail.cgi?targetID=3253485) | 252 | 91 | hsa-miR-15a-5p | [RPS6KA6](http://www.ncbi.nlm.nih.gov/entrez/query.fcgi?db=gene&cmd=Retrieve&dopt=full_report&list_uids=27330) | ribosomal protein S6 kinase A6 |
| [Details](https://mirdb.org/cgi-bin/target_detail.cgi?targetID=3253505) | 253 | 91 | hsa-miR-15a-5p | [TLL1](http://www.ncbi.nlm.nih.gov/entrez/query.fcgi?db=gene&cmd=Retrieve&dopt=full_report&list_uids=7092) | tolloid like 1 |
| [Details](https://mirdb.org/cgi-bin/target_detail.cgi?targetID=3253522) | 254 | 91 | hsa-miR-15a-5p | [EZH1](http://www.ncbi.nlm.nih.gov/entrez/query.fcgi?db=gene&cmd=Retrieve&dopt=full_report&list_uids=2145) | enhancer of zeste 1 polycomb repressive complex 2 subunit |
| [Details](https://mirdb.org/cgi-bin/target_detail.cgi?targetID=3253583) | 255 | 91 | hsa-miR-15a-5p | [SPTLC1](http://www.ncbi.nlm.nih.gov/entrez/query.fcgi?db=gene&cmd=Retrieve&dopt=full_report&list_uids=10558) | serine palmitoyltransferase long chain base subunit 1 |
| [Details](https://mirdb.org/cgi-bin/target_detail.cgi?targetID=3253595) | 256 | 91 | hsa-miR-15a-5p | [GATAD2A](http://www.ncbi.nlm.nih.gov/entrez/query.fcgi?db=gene&cmd=Retrieve&dopt=full_report&list_uids=54815) | GATA zinc finger domain containing 2A |
| [Details](https://mirdb.org/cgi-bin/target_detail.cgi?targetID=3253619) | 257 | 91 | hsa-miR-15a-5p | [AMER1](http://www.ncbi.nlm.nih.gov/entrez/query.fcgi?db=gene&cmd=Retrieve&dopt=full_report&list_uids=139285) | APC membrane recruitment protein 1 |
| [Details](https://mirdb.org/cgi-bin/target_detail.cgi?targetID=3253670) | 258 | 91 | hsa-miR-15a-5p | [LARGE2](http://www.ncbi.nlm.nih.gov/entrez/query.fcgi?db=gene&cmd=Retrieve&dopt=full_report&list_uids=120071) | LARGE xylosyl- and glucuronyltransferase 2 |
| [Details](https://mirdb.org/cgi-bin/target_detail.cgi?targetID=3253785) | 259 | 91 | hsa-miR-15a-5p | [RARB](http://www.ncbi.nlm.nih.gov/entrez/query.fcgi?db=gene&cmd=Retrieve&dopt=full_report&list_uids=5915) | retinoic acid receptor beta |
| [Details](https://mirdb.org/cgi-bin/target_detail.cgi?targetID=3253804) | 260 | 91 | hsa-miR-15a-5p | [SLC6A11](http://www.ncbi.nlm.nih.gov/entrez/query.fcgi?db=gene&cmd=Retrieve&dopt=full_report&list_uids=6538) | solute carrier family 6 member 11 |
| [Details](https://mirdb.org/cgi-bin/target_detail.cgi?targetID=3253847) | 261 | 91 | hsa-miR-15a-5p | [ZC3H13](http://www.ncbi.nlm.nih.gov/entrez/query.fcgi?db=gene&cmd=Retrieve&dopt=full_report&list_uids=23091) | zinc finger CCCH-type containing 13 |
| [Details](https://mirdb.org/cgi-bin/target_detail.cgi?targetID=3253936) | 262 | 91 | hsa-miR-15a-5p | [CSDE1](http://www.ncbi.nlm.nih.gov/entrez/query.fcgi?db=gene&cmd=Retrieve&dopt=full_report&list_uids=7812) | cold shock domain containing E1 |
| [Details](https://mirdb.org/cgi-bin/target_detail.cgi?targetID=3252650) | 263 | 90 | hsa-miR-15a-5p | [WIPI2](http://www.ncbi.nlm.nih.gov/entrez/query.fcgi?db=gene&cmd=Retrieve&dopt=full_report&list_uids=26100) | WD repeat domain, phosphoinositide interacting 2 |
| [Details](https://mirdb.org/cgi-bin/target_detail.cgi?targetID=3252653) | 264 | 90 | hsa-miR-15a-5p | [TFCP2L1](http://www.ncbi.nlm.nih.gov/entrez/query.fcgi?db=gene&cmd=Retrieve&dopt=full_report&list_uids=29842) | transcription factor CP2 like 1 |
| [Details](https://mirdb.org/cgi-bin/target_detail.cgi?targetID=3252826) | 265 | 90 | hsa-miR-15a-5p | [TGFBR3](http://www.ncbi.nlm.nih.gov/entrez/query.fcgi?db=gene&cmd=Retrieve&dopt=full_report&list_uids=7049) | transforming growth factor beta receptor 3 |
| [Details](https://mirdb.org/cgi-bin/target_detail.cgi?targetID=3252928) | 266 | 90 | hsa-miR-15a-5p | [FAM133B](http://www.ncbi.nlm.nih.gov/entrez/query.fcgi?db=gene&cmd=Retrieve&dopt=full_report&list_uids=257415) | family with sequence similarity 133 member B |
| [Details](https://mirdb.org/cgi-bin/target_detail.cgi?targetID=3252931) | 267 | 90 | hsa-miR-15a-5p | [UBQLNL](http://www.ncbi.nlm.nih.gov/entrez/query.fcgi?db=gene&cmd=Retrieve&dopt=full_report&list_uids=143630) | ubiquilin like |
| [Details](https://mirdb.org/cgi-bin/target_detail.cgi?targetID=3252934) | 268 | 90 | hsa-miR-15a-5p | [TRIM66](http://www.ncbi.nlm.nih.gov/entrez/query.fcgi?db=gene&cmd=Retrieve&dopt=full_report&list_uids=9866) | tripartite motif containing 66 |
| [Details](https://mirdb.org/cgi-bin/target_detail.cgi?targetID=3252943) | 269 | 90 | hsa-miR-15a-5p | [ELMSAN1](http://www.ncbi.nlm.nih.gov/entrez/query.fcgi?db=gene&cmd=Retrieve&dopt=full_report&list_uids=91748) | ELM2 and Myb/SANT domain containing 1 |
| [Details](https://mirdb.org/cgi-bin/target_detail.cgi?targetID=3253041) | 270 | 90 | hsa-miR-15a-5p | [AK4](http://www.ncbi.nlm.nih.gov/entrez/query.fcgi?db=gene&cmd=Retrieve&dopt=full_report&list_uids=205) | adenylate kinase 4 |
| [Details](https://mirdb.org/cgi-bin/target_detail.cgi?targetID=3253054) | 271 | 90 | hsa-miR-15a-5p | [ABHD2](http://www.ncbi.nlm.nih.gov/entrez/query.fcgi?db=gene&cmd=Retrieve&dopt=full_report&list_uids=11057) | abhydrolase domain containing 2 |
| [Details](https://mirdb.org/cgi-bin/target_detail.cgi?targetID=3253103) | 272 | 90 | hsa-miR-15a-5p | [C1orf21](http://www.ncbi.nlm.nih.gov/entrez/query.fcgi?db=gene&cmd=Retrieve&dopt=full_report&list_uids=81563) | chromosome 1 open reading frame 21 |
| [Details](https://mirdb.org/cgi-bin/target_detail.cgi?targetID=3253214) | 273 | 90 | hsa-miR-15a-5p | [NRN1](http://www.ncbi.nlm.nih.gov/entrez/query.fcgi?db=gene&cmd=Retrieve&dopt=full_report&list_uids=51299) | neuritin 1 |
| [Details](https://mirdb.org/cgi-bin/target_detail.cgi?targetID=3253238) | 274 | 90 | hsa-miR-15a-5p | [ADGRL1](http://www.ncbi.nlm.nih.gov/entrez/query.fcgi?db=gene&cmd=Retrieve&dopt=full_report&list_uids=22859) | adhesion G protein-coupled receptor L1 |
| [Details](https://mirdb.org/cgi-bin/target_detail.cgi?targetID=3253306) | 275 | 90 | hsa-miR-15a-5p | [JARID2](http://www.ncbi.nlm.nih.gov/entrez/query.fcgi?db=gene&cmd=Retrieve&dopt=full_report&list_uids=3720) | jumonji and AT-rich interaction domain containing 2 |
| [Details](https://mirdb.org/cgi-bin/target_detail.cgi?targetID=3253340) | 276 | 90 | hsa-miR-15a-5p | [SYNRG](http://www.ncbi.nlm.nih.gov/entrez/query.fcgi?db=gene&cmd=Retrieve&dopt=full_report&list_uids=11276) | synergin gamma |
| [Details](https://mirdb.org/cgi-bin/target_detail.cgi?targetID=3253395) | 277 | 90 | hsa-miR-15a-5p | [USP31](http://www.ncbi.nlm.nih.gov/entrez/query.fcgi?db=gene&cmd=Retrieve&dopt=full_report&list_uids=57478) | ubiquitin specific peptidase 31 |
| [Details](https://mirdb.org/cgi-bin/target_detail.cgi?targetID=3253398) | 278 | 90 | hsa-miR-15a-5p | [CD47](http://www.ncbi.nlm.nih.gov/entrez/query.fcgi?db=gene&cmd=Retrieve&dopt=full_report&list_uids=961) | CD47 molecule |
| [Details](https://mirdb.org/cgi-bin/target_detail.cgi?targetID=3253399) | 279 | 90 | hsa-miR-15a-5p | [SEMA3A](http://www.ncbi.nlm.nih.gov/entrez/query.fcgi?db=gene&cmd=Retrieve&dopt=full_report&list_uids=10371) | semaphorin 3A |
| [Details](https://mirdb.org/cgi-bin/target_detail.cgi?targetID=3253436) | 280 | 90 | hsa-miR-15a-5p | [LRIG1](http://www.ncbi.nlm.nih.gov/entrez/query.fcgi?db=gene&cmd=Retrieve&dopt=full_report&list_uids=26018) | leucine rich repeats and immunoglobulin like domains 1 |
| [Details](https://mirdb.org/cgi-bin/target_detail.cgi?targetID=3253462) | 281 | 90 | hsa-miR-15a-5p | [TMC7](http://www.ncbi.nlm.nih.gov/entrez/query.fcgi?db=gene&cmd=Retrieve&dopt=full_report&list_uids=79905) | transmembrane channel like 7 |
| [Details](https://mirdb.org/cgi-bin/target_detail.cgi?targetID=3253642) | 282 | 90 | hsa-miR-15a-5p | [DENND1B](http://www.ncbi.nlm.nih.gov/entrez/query.fcgi?db=gene&cmd=Retrieve&dopt=full_report&list_uids=163486) | DENN domain containing 1B |
| [Details](https://mirdb.org/cgi-bin/target_detail.cgi?targetID=3253652) | 283 | 90 | hsa-miR-15a-5p | [RAB9B](http://www.ncbi.nlm.nih.gov/entrez/query.fcgi?db=gene&cmd=Retrieve&dopt=full_report&list_uids=51209) | RAB9B, member RAS oncogene family |
| [Details](https://mirdb.org/cgi-bin/target_detail.cgi?targetID=3253689) | 284 | 90 | hsa-miR-15a-5p | [AMOT](http://www.ncbi.nlm.nih.gov/entrez/query.fcgi?db=gene&cmd=Retrieve&dopt=full_report&list_uids=154796) | angiomotin |
| [Details](https://mirdb.org/cgi-bin/target_detail.cgi?targetID=3253762) | 285 | 90 | hsa-miR-15a-5p | [FERMT2](http://www.ncbi.nlm.nih.gov/entrez/query.fcgi?db=gene&cmd=Retrieve&dopt=full_report&list_uids=10979) | fermitin family member 2 |
| [Details](https://mirdb.org/cgi-bin/target_detail.cgi?targetID=3253763) | 286 | 90 | hsa-miR-15a-5p | [ILDR2](http://www.ncbi.nlm.nih.gov/entrez/query.fcgi?db=gene&cmd=Retrieve&dopt=full_report&list_uids=387597) | immunoglobulin like domain containing receptor 2 |
| [Details](https://mirdb.org/cgi-bin/target_detail.cgi?targetID=3253766) | 287 | 90 | hsa-miR-15a-5p | [CSRNP1](http://www.ncbi.nlm.nih.gov/entrez/query.fcgi?db=gene&cmd=Retrieve&dopt=full_report&list_uids=64651) | cysteine and serine rich nuclear protein 1 |
| [Details](https://mirdb.org/cgi-bin/target_detail.cgi?targetID=3253834) | 288 | 90 | hsa-miR-15a-5p | [NR2C2](http://www.ncbi.nlm.nih.gov/entrez/query.fcgi?db=gene&cmd=Retrieve&dopt=full_report&list_uids=7182) | nuclear receptor subfamily 2 group C member 2 |
| [Details](https://mirdb.org/cgi-bin/target_detail.cgi?targetID=3253870) | 289 | 90 | hsa-miR-15a-5p | [C12orf76](http://www.ncbi.nlm.nih.gov/entrez/query.fcgi?db=gene&cmd=Retrieve&dopt=full_report&list_uids=400073) | chromosome 12 open reading frame 76 |
| [Details](https://mirdb.org/cgi-bin/target_detail.cgi?targetID=3254028) | 290 | 90 | hsa-miR-15a-5p | [BAG4](http://www.ncbi.nlm.nih.gov/entrez/query.fcgi?db=gene&cmd=Retrieve&dopt=full_report&list_uids=9530) | BCL2 associated athanogene 4 |
| [Details](https://mirdb.org/cgi-bin/target_detail.cgi?targetID=3252689) | 291 | 89 | hsa-miR-15a-5p | [DYRK1B](http://www.ncbi.nlm.nih.gov/entrez/query.fcgi?db=gene&cmd=Retrieve&dopt=full_report&list_uids=9149) | dual specificity tyrosine phosphorylation regulated kinase 1B |
| [Details](https://mirdb.org/cgi-bin/target_detail.cgi?targetID=3252780) | 292 | 89 | hsa-miR-15a-5p | [ZSCAN31](http://www.ncbi.nlm.nih.gov/entrez/query.fcgi?db=gene&cmd=Retrieve&dopt=full_report&list_uids=64288) | zinc finger and SCAN domain containing 31 |
| [Details](https://mirdb.org/cgi-bin/target_detail.cgi?targetID=3252797) | 293 | 89 | hsa-miR-15a-5p | [PDZD8](http://www.ncbi.nlm.nih.gov/entrez/query.fcgi?db=gene&cmd=Retrieve&dopt=full_report&list_uids=118987) | PDZ domain containing 8 |
| [Details](https://mirdb.org/cgi-bin/target_detail.cgi?targetID=3252882) | 294 | 89 | hsa-miR-15a-5p | [ARFGAP2](http://www.ncbi.nlm.nih.gov/entrez/query.fcgi?db=gene&cmd=Retrieve&dopt=full_report&list_uids=84364) | ADP ribosylation factor GTPase activating protein 2 |
| [Details](https://mirdb.org/cgi-bin/target_detail.cgi?targetID=3252918) | 295 | 89 | hsa-miR-15a-5p | [CDK17](http://www.ncbi.nlm.nih.gov/entrez/query.fcgi?db=gene&cmd=Retrieve&dopt=full_report&list_uids=5128) | cyclin dependent kinase 17 |
| [Details](https://mirdb.org/cgi-bin/target_detail.cgi?targetID=3252925) | 296 | 89 | hsa-miR-15a-5p | [ROCK2](http://www.ncbi.nlm.nih.gov/entrez/query.fcgi?db=gene&cmd=Retrieve&dopt=full_report&list_uids=9475) | Rho associated coiled-coil containing protein kinase 2 |
| [Details](https://mirdb.org/cgi-bin/target_detail.cgi?targetID=3253036) | 297 | 89 | hsa-miR-15a-5p | [PNPLA6](http://www.ncbi.nlm.nih.gov/entrez/query.fcgi?db=gene&cmd=Retrieve&dopt=full_report&list_uids=10908) | patatin like phospholipase domain containing 6 |
| [Details](https://mirdb.org/cgi-bin/target_detail.cgi?targetID=3253062) | 298 | 89 | hsa-miR-15a-5p | [MED26](http://www.ncbi.nlm.nih.gov/entrez/query.fcgi?db=gene&cmd=Retrieve&dopt=full_report&list_uids=9441) | mediator complex subunit 26 |
| [Details](https://mirdb.org/cgi-bin/target_detail.cgi?targetID=3253130) | 299 | 89 | hsa-miR-15a-5p | [ATXN1L](http://www.ncbi.nlm.nih.gov/entrez/query.fcgi?db=gene&cmd=Retrieve&dopt=full_report&list_uids=342371) | ataxin 1 like |
| [Details](https://mirdb.org/cgi-bin/target_detail.cgi?targetID=3253203) | 300 | 89 | hsa-miR-15a-5p | [ISLR](http://www.ncbi.nlm.nih.gov/entrez/query.fcgi?db=gene&cmd=Retrieve&dopt=full_report&list_uids=3671) | immunoglobulin superfamily containing leucine rich repeat |
| [Details](https://mirdb.org/cgi-bin/target_detail.cgi?targetID=3253208) | 301 | 89 | hsa-miR-15a-5p | [PRDM4](http://www.ncbi.nlm.nih.gov/entrez/query.fcgi?db=gene&cmd=Retrieve&dopt=full_report&list_uids=11108) | PR/SET domain 4 |
| [Details](https://mirdb.org/cgi-bin/target_detail.cgi?targetID=3253342) | 302 | 89 | hsa-miR-15a-5p | [SKI](http://www.ncbi.nlm.nih.gov/entrez/query.fcgi?db=gene&cmd=Retrieve&dopt=full_report&list_uids=6497) | SKI proto-oncogene |
| [Details](https://mirdb.org/cgi-bin/target_detail.cgi?targetID=3253362) | 303 | 89 | hsa-miR-15a-5p | [MNT](http://www.ncbi.nlm.nih.gov/entrez/query.fcgi?db=gene&cmd=Retrieve&dopt=full_report&list_uids=4335) | MAX network transcriptional repressor |
| [Details](https://mirdb.org/cgi-bin/target_detail.cgi?targetID=3253366) | 304 | 89 | hsa-miR-15a-5p | [GSTCD](http://www.ncbi.nlm.nih.gov/entrez/query.fcgi?db=gene&cmd=Retrieve&dopt=full_report&list_uids=79807) | glutathione S-transferase C-terminal domain containing |
| [Details](https://mirdb.org/cgi-bin/target_detail.cgi?targetID=3253386) | 305 | 89 | hsa-miR-15a-5p | [CBX6](http://www.ncbi.nlm.nih.gov/entrez/query.fcgi?db=gene&cmd=Retrieve&dopt=full_report&list_uids=23466) | chromobox 6 |
| [Details](https://mirdb.org/cgi-bin/target_detail.cgi?targetID=3253404) | 306 | 89 | hsa-miR-15a-5p | [PAG1](http://www.ncbi.nlm.nih.gov/entrez/query.fcgi?db=gene&cmd=Retrieve&dopt=full_report&list_uids=55824) | phosphoprotein membrane anchor with glycosphingolipid microdomains 1 |
| [Details](https://mirdb.org/cgi-bin/target_detail.cgi?targetID=3253412) | 307 | 89 | hsa-miR-15a-5p | [TAB3](http://www.ncbi.nlm.nih.gov/entrez/query.fcgi?db=gene&cmd=Retrieve&dopt=full_report&list_uids=257397) | TGF-beta activated kinase 1 (MAP3K7) binding protein 3 |
| [Details](https://mirdb.org/cgi-bin/target_detail.cgi?targetID=3253431) | 308 | 89 | hsa-miR-15a-5p | [PARVA](http://www.ncbi.nlm.nih.gov/entrez/query.fcgi?db=gene&cmd=Retrieve&dopt=full_report&list_uids=55742) | parvin alpha |
| [Details](https://mirdb.org/cgi-bin/target_detail.cgi?targetID=3253433) | 309 | 89 | hsa-miR-15a-5p | [MKNK1](http://www.ncbi.nlm.nih.gov/entrez/query.fcgi?db=gene&cmd=Retrieve&dopt=full_report&list_uids=8569) | MAP kinase interacting serine/threonine kinase 1 |
| [Details](https://mirdb.org/cgi-bin/target_detail.cgi?targetID=3253449) | 310 | 89 | hsa-miR-15a-5p | [CMPK1](http://www.ncbi.nlm.nih.gov/entrez/query.fcgi?db=gene&cmd=Retrieve&dopt=full_report&list_uids=51727) | cytidine/uridine monophosphate kinase 1 |
| [Details](https://mirdb.org/cgi-bin/target_detail.cgi?targetID=3253515) | 311 | 89 | hsa-miR-15a-5p | [COL12A1](http://www.ncbi.nlm.nih.gov/entrez/query.fcgi?db=gene&cmd=Retrieve&dopt=full_report&list_uids=1303) | collagen type XII alpha 1 chain |
| [Details](https://mirdb.org/cgi-bin/target_detail.cgi?targetID=3253527) | 312 | 89 | hsa-miR-15a-5p | [SERBP1](http://www.ncbi.nlm.nih.gov/entrez/query.fcgi?db=gene&cmd=Retrieve&dopt=full_report&list_uids=26135) | SERPINE1 mRNA binding protein 1 |
| [Details](https://mirdb.org/cgi-bin/target_detail.cgi?targetID=3253539) | 313 | 89 | hsa-miR-15a-5p | [ETNK1](http://www.ncbi.nlm.nih.gov/entrez/query.fcgi?db=gene&cmd=Retrieve&dopt=full_report&list_uids=55500) | ethanolamine kinase 1 |
| [Details](https://mirdb.org/cgi-bin/target_detail.cgi?targetID=3253547) | 314 | 89 | hsa-miR-15a-5p | [ANKS1A](http://www.ncbi.nlm.nih.gov/entrez/query.fcgi?db=gene&cmd=Retrieve&dopt=full_report&list_uids=23294) | ankyrin repeat and sterile alpha motif domain containing 1A |
| [Details](https://mirdb.org/cgi-bin/target_detail.cgi?targetID=3253581) | 315 | 89 | hsa-miR-15a-5p | [HIGD1A](http://www.ncbi.nlm.nih.gov/entrez/query.fcgi?db=gene&cmd=Retrieve&dopt=full_report&list_uids=25994) | HIG1 hypoxia inducible domain family member 1A |
| [Details](https://mirdb.org/cgi-bin/target_detail.cgi?targetID=3253660) | 316 | 89 | hsa-miR-15a-5p | [BCL11B](http://www.ncbi.nlm.nih.gov/entrez/query.fcgi?db=gene&cmd=Retrieve&dopt=full_report&list_uids=64919) | BCL11B, BAF complex component |
| [Details](https://mirdb.org/cgi-bin/target_detail.cgi?targetID=3253691) | 317 | 89 | hsa-miR-15a-5p | [ARHGAP32](http://www.ncbi.nlm.nih.gov/entrez/query.fcgi?db=gene&cmd=Retrieve&dopt=full_report&list_uids=9743) | Rho GTPase activating protein 32 |
| [Details](https://mirdb.org/cgi-bin/target_detail.cgi?targetID=3253694) | 318 | 89 | hsa-miR-15a-5p | [TMEM189-UBE2V1](http://www.ncbi.nlm.nih.gov/entrez/query.fcgi?db=gene&cmd=Retrieve&dopt=full_report&list_uids=387522) | TMEM189-UBE2V1 readthrough |
| [Details](https://mirdb.org/cgi-bin/target_detail.cgi?targetID=3253717) | 319 | 89 | hsa-miR-15a-5p | [FAM91A1](http://www.ncbi.nlm.nih.gov/entrez/query.fcgi?db=gene&cmd=Retrieve&dopt=full_report&list_uids=157769) | family with sequence similarity 91 member A1 |
| [Details](https://mirdb.org/cgi-bin/target_detail.cgi?targetID=3253719) | 320 | 89 | hsa-miR-15a-5p | [SEMA5B](http://www.ncbi.nlm.nih.gov/entrez/query.fcgi?db=gene&cmd=Retrieve&dopt=full_report&list_uids=54437) | semaphorin 5B |
| [Details](https://mirdb.org/cgi-bin/target_detail.cgi?targetID=3253757) | 321 | 89 | hsa-miR-15a-5p | [SGK1](http://www.ncbi.nlm.nih.gov/entrez/query.fcgi?db=gene&cmd=Retrieve&dopt=full_report&list_uids=6446) | serum/glucocorticoid regulated kinase 1 |
| [Details](https://mirdb.org/cgi-bin/target_detail.cgi?targetID=3253769) | 322 | 89 | hsa-miR-15a-5p | [RS1](http://www.ncbi.nlm.nih.gov/entrez/query.fcgi?db=gene&cmd=Retrieve&dopt=full_report&list_uids=6247) | retinoschisin 1 |
| [Details](https://mirdb.org/cgi-bin/target_detail.cgi?targetID=3253824) | 323 | 89 | hsa-miR-15a-5p | [PIP4P2](http://www.ncbi.nlm.nih.gov/entrez/query.fcgi?db=gene&cmd=Retrieve&dopt=full_report&list_uids=55529) | phosphatidylinositol-4,5-bisphosphate 4-phosphatase 2 |
| [Details](https://mirdb.org/cgi-bin/target_detail.cgi?targetID=3253832) | 324 | 89 | hsa-miR-15a-5p | [CHPT1](http://www.ncbi.nlm.nih.gov/entrez/query.fcgi?db=gene&cmd=Retrieve&dopt=full_report&list_uids=56994) | choline phosphotransferase 1 |
| [Details](https://mirdb.org/cgi-bin/target_detail.cgi?targetID=3253981) | 325 | 89 | hsa-miR-15a-5p | [MYO5B](http://www.ncbi.nlm.nih.gov/entrez/query.fcgi?db=gene&cmd=Retrieve&dopt=full_report&list_uids=4645) | myosin VB |
| [Details](https://mirdb.org/cgi-bin/target_detail.cgi?targetID=3253984) | 326 | 89 | hsa-miR-15a-5p | [PCDH9](http://www.ncbi.nlm.nih.gov/entrez/query.fcgi?db=gene&cmd=Retrieve&dopt=full_report&list_uids=5101) | protocadherin 9 |
| [Details](https://mirdb.org/cgi-bin/target_detail.cgi?targetID=3254027) | 327 | 89 | hsa-miR-15a-5p | [CLCN4](http://www.ncbi.nlm.nih.gov/entrez/query.fcgi?db=gene&cmd=Retrieve&dopt=full_report&list_uids=1183) | chloride voltage-gated channel 4 |
| [Details](https://mirdb.org/cgi-bin/target_detail.cgi?targetID=3252645) | 328 | 88 | hsa-miR-15a-5p | [TCAIM](http://www.ncbi.nlm.nih.gov/entrez/query.fcgi?db=gene&cmd=Retrieve&dopt=full_report&list_uids=285343) | T cell activation inhibitor, mitochondrial |
| [Details](https://mirdb.org/cgi-bin/target_detail.cgi?targetID=3252652) | 329 | 88 | hsa-miR-15a-5p | [EPHA7](http://www.ncbi.nlm.nih.gov/entrez/query.fcgi?db=gene&cmd=Retrieve&dopt=full_report&list_uids=2045) | EPH receptor A7 |
| [Details](https://mirdb.org/cgi-bin/target_detail.cgi?targetID=3252680) | 330 | 88 | hsa-miR-15a-5p | [TMEM268](http://www.ncbi.nlm.nih.gov/entrez/query.fcgi?db=gene&cmd=Retrieve&dopt=full_report&list_uids=203197) | transmembrane protein 268 |
| [Details](https://mirdb.org/cgi-bin/target_detail.cgi?targetID=3252732) | 331 | 88 | hsa-miR-15a-5p | [CACNA2D1](http://www.ncbi.nlm.nih.gov/entrez/query.fcgi?db=gene&cmd=Retrieve&dopt=full_report&list_uids=781) | calcium voltage-gated channel auxiliary subunit alpha2delta 1 |
| [Details](https://mirdb.org/cgi-bin/target_detail.cgi?targetID=3252734) | 332 | 88 | hsa-miR-15a-5p | [RASEF](http://www.ncbi.nlm.nih.gov/entrez/query.fcgi?db=gene&cmd=Retrieve&dopt=full_report&list_uids=158158) | RAS and EF-hand domain containing |
| [Details](https://mirdb.org/cgi-bin/target_detail.cgi?targetID=3252745) | 333 | 88 | hsa-miR-15a-5p | [RREB1](http://www.ncbi.nlm.nih.gov/entrez/query.fcgi?db=gene&cmd=Retrieve&dopt=full_report&list_uids=6239) | ras responsive element binding protein 1 |
| [Details](https://mirdb.org/cgi-bin/target_detail.cgi?targetID=3252775) | 334 | 88 | hsa-miR-15a-5p | [CAPRIN1](http://www.ncbi.nlm.nih.gov/entrez/query.fcgi?db=gene&cmd=Retrieve&dopt=full_report&list_uids=4076) | cell cycle associated protein 1 |
| [Details](https://mirdb.org/cgi-bin/target_detail.cgi?targetID=3252843) | 335 | 88 | hsa-miR-15a-5p | [EGLN1](http://www.ncbi.nlm.nih.gov/entrez/query.fcgi?db=gene&cmd=Retrieve&dopt=full_report&list_uids=54583) | egl-9 family hypoxia inducible factor 1 |
| [Details](https://mirdb.org/cgi-bin/target_detail.cgi?targetID=3252899) | 336 | 88 | hsa-miR-15a-5p | [MAP3K13](http://www.ncbi.nlm.nih.gov/entrez/query.fcgi?db=gene&cmd=Retrieve&dopt=full_report&list_uids=9175) | mitogen-activated protein kinase kinase kinase 13 |
| [Details](https://mirdb.org/cgi-bin/target_detail.cgi?targetID=3252936) | 337 | 88 | hsa-miR-15a-5p | [PRRC2C](http://www.ncbi.nlm.nih.gov/entrez/query.fcgi?db=gene&cmd=Retrieve&dopt=full_report&list_uids=23215) | proline rich coiled-coil 2C |
| [Details](https://mirdb.org/cgi-bin/target_detail.cgi?targetID=3252964) | 338 | 88 | hsa-miR-15a-5p | [CEP85L](http://www.ncbi.nlm.nih.gov/entrez/query.fcgi?db=gene&cmd=Retrieve&dopt=full_report&list_uids=387119) | centrosomal protein 85 like |
| [Details](https://mirdb.org/cgi-bin/target_detail.cgi?targetID=3252967) | 339 | 88 | hsa-miR-15a-5p | [RBM6](http://www.ncbi.nlm.nih.gov/entrez/query.fcgi?db=gene&cmd=Retrieve&dopt=full_report&list_uids=10180) | RNA binding motif protein 6 |
| [Details](https://mirdb.org/cgi-bin/target_detail.cgi?targetID=3252986) | 340 | 88 | hsa-miR-15a-5p | [EXOC3L2](http://www.ncbi.nlm.nih.gov/entrez/query.fcgi?db=gene&cmd=Retrieve&dopt=full_report&list_uids=90332) | exocyst complex component 3 like 2 |
| [Details](https://mirdb.org/cgi-bin/target_detail.cgi?targetID=3253034) | 341 | 88 | hsa-miR-15a-5p | [CDC37L1](http://www.ncbi.nlm.nih.gov/entrez/query.fcgi?db=gene&cmd=Retrieve&dopt=full_report&list_uids=55664) | cell division cycle 37 like 1 |
| [Details](https://mirdb.org/cgi-bin/target_detail.cgi?targetID=3253067) | 342 | 88 | hsa-miR-15a-5p | [NRP2](http://www.ncbi.nlm.nih.gov/entrez/query.fcgi?db=gene&cmd=Retrieve&dopt=full_report&list_uids=8828) | neuropilin 2 |
| [Details](https://mirdb.org/cgi-bin/target_detail.cgi?targetID=3253088) | 343 | 88 | hsa-miR-15a-5p | [SCOC](http://www.ncbi.nlm.nih.gov/entrez/query.fcgi?db=gene&cmd=Retrieve&dopt=full_report&list_uids=60592) | short coiled-coil protein |
| [Details](https://mirdb.org/cgi-bin/target_detail.cgi?targetID=3253092) | 344 | 88 | hsa-miR-15a-5p | [HERC6](http://www.ncbi.nlm.nih.gov/entrez/query.fcgi?db=gene&cmd=Retrieve&dopt=full_report&list_uids=55008) | HECT and RLD domain containing E3 ubiquitin protein ligase family member 6 |
| [Details](https://mirdb.org/cgi-bin/target_detail.cgi?targetID=3253118) | 345 | 88 | hsa-miR-15a-5p | [GNAT1](http://www.ncbi.nlm.nih.gov/entrez/query.fcgi?db=gene&cmd=Retrieve&dopt=full_report&list_uids=2779) | G protein subunit alpha transducin 1 |
| [Details](https://mirdb.org/cgi-bin/target_detail.cgi?targetID=3253146) | 346 | 88 | hsa-miR-15a-5p | [DRD1](http://www.ncbi.nlm.nih.gov/entrez/query.fcgi?db=gene&cmd=Retrieve&dopt=full_report&list_uids=1812) | dopamine receptor D1 |
| [Details](https://mirdb.org/cgi-bin/target_detail.cgi?targetID=3253240) | 347 | 88 | hsa-miR-15a-5p | [CPD](http://www.ncbi.nlm.nih.gov/entrez/query.fcgi?db=gene&cmd=Retrieve&dopt=full_report&list_uids=1362) | carboxypeptidase D |
| [Details](https://mirdb.org/cgi-bin/target_detail.cgi?targetID=3253318) | 348 | 88 | hsa-miR-15a-5p | [CXCR5](http://www.ncbi.nlm.nih.gov/entrez/query.fcgi?db=gene&cmd=Retrieve&dopt=full_report&list_uids=643) | C-X-C motif chemokine receptor 5 |
| [Details](https://mirdb.org/cgi-bin/target_detail.cgi?targetID=3253321) | 349 | 88 | hsa-miR-15a-5p | [PDIA6](http://www.ncbi.nlm.nih.gov/entrez/query.fcgi?db=gene&cmd=Retrieve&dopt=full_report&list_uids=10130) | protein disulfide isomerase family A member 6 |
| [Details](https://mirdb.org/cgi-bin/target_detail.cgi?targetID=3253360) | 350 | 88 | hsa-miR-15a-5p | [RBBP6](http://www.ncbi.nlm.nih.gov/entrez/query.fcgi?db=gene&cmd=Retrieve&dopt=full_report&list_uids=5930) | RB binding protein 6, ubiquitin ligase |
| [Details](https://mirdb.org/cgi-bin/target_detail.cgi?targetID=3253376) | 351 | 88 | hsa-miR-15a-5p | [PIK3R1](http://www.ncbi.nlm.nih.gov/entrez/query.fcgi?db=gene&cmd=Retrieve&dopt=full_report&list_uids=5295) | phosphoinositide-3-kinase regulatory subunit 1 |
| [Details](https://mirdb.org/cgi-bin/target_detail.cgi?targetID=3253389) | 352 | 88 | hsa-miR-15a-5p | [USP15](http://www.ncbi.nlm.nih.gov/entrez/query.fcgi?db=gene&cmd=Retrieve&dopt=full_report&list_uids=9958) | ubiquitin specific peptidase 15 |
| [Details](https://mirdb.org/cgi-bin/target_detail.cgi?targetID=3253443) | 353 | 88 | hsa-miR-15a-5p | [ADRB2](http://www.ncbi.nlm.nih.gov/entrez/query.fcgi?db=gene&cmd=Retrieve&dopt=full_report&list_uids=154) | adrenoceptor beta 2 |
| [Details](https://mirdb.org/cgi-bin/target_detail.cgi?targetID=3253563) | 354 | 88 | hsa-miR-15a-5p | [WNT7A](http://www.ncbi.nlm.nih.gov/entrez/query.fcgi?db=gene&cmd=Retrieve&dopt=full_report&list_uids=7476) | Wnt family member 7A |
| [Details](https://mirdb.org/cgi-bin/target_detail.cgi?targetID=3253585) | 355 | 88 | hsa-miR-15a-5p | [SNRPB2](http://www.ncbi.nlm.nih.gov/entrez/query.fcgi?db=gene&cmd=Retrieve&dopt=full_report&list_uids=6629) | small nuclear ribonucleoprotein polypeptide B2 |
| [Details](https://mirdb.org/cgi-bin/target_detail.cgi?targetID=3253596) | 356 | 88 | hsa-miR-15a-5p | [QKI](http://www.ncbi.nlm.nih.gov/entrez/query.fcgi?db=gene&cmd=Retrieve&dopt=full_report&list_uids=9444) | QKI, KH domain containing RNA binding |
| [Details](https://mirdb.org/cgi-bin/target_detail.cgi?targetID=3253658) | 357 | 88 | hsa-miR-15a-5p | [DMTF1](http://www.ncbi.nlm.nih.gov/entrez/query.fcgi?db=gene&cmd=Retrieve&dopt=full_report&list_uids=9988) | cyclin D binding myb like transcription factor 1 |
| [Details](https://mirdb.org/cgi-bin/target_detail.cgi?targetID=3253743) | 358 | 88 | hsa-miR-15a-5p | [LRP6](http://www.ncbi.nlm.nih.gov/entrez/query.fcgi?db=gene&cmd=Retrieve&dopt=full_report&list_uids=4040) | LDL receptor related protein 6 |
| [Details](https://mirdb.org/cgi-bin/target_detail.cgi?targetID=3253792) | 359 | 88 | hsa-miR-15a-5p | [BZW1](http://www.ncbi.nlm.nih.gov/entrez/query.fcgi?db=gene&cmd=Retrieve&dopt=full_report&list_uids=9689) | basic leucine zipper and W2 domains 1 |
| [Details](https://mirdb.org/cgi-bin/target_detail.cgi?targetID=3253836) | 360 | 88 | hsa-miR-15a-5p | [FAM81A](http://www.ncbi.nlm.nih.gov/entrez/query.fcgi?db=gene&cmd=Retrieve&dopt=full_report&list_uids=145773) | family with sequence similarity 81 member A |
| [Details](https://mirdb.org/cgi-bin/target_detail.cgi?targetID=3253865) | 361 | 88 | hsa-miR-15a-5p | [SMIM13](http://www.ncbi.nlm.nih.gov/entrez/query.fcgi?db=gene&cmd=Retrieve&dopt=full_report&list_uids=221710) | small integral membrane protein 13 |
| [Details](https://mirdb.org/cgi-bin/target_detail.cgi?targetID=3253895) | 362 | 88 | hsa-miR-15a-5p | [GLS2](http://www.ncbi.nlm.nih.gov/entrez/query.fcgi?db=gene&cmd=Retrieve&dopt=full_report&list_uids=27165) | glutaminase 2 |
| [Details](https://mirdb.org/cgi-bin/target_detail.cgi?targetID=3253907) | 363 | 88 | hsa-miR-15a-5p | [MOB4](http://www.ncbi.nlm.nih.gov/entrez/query.fcgi?db=gene&cmd=Retrieve&dopt=full_report&list_uids=25843) | MOB family member 4, phocein |
| [Details](https://mirdb.org/cgi-bin/target_detail.cgi?targetID=3253910) | 364 | 88 | hsa-miR-15a-5p | [KCNG4](http://www.ncbi.nlm.nih.gov/entrez/query.fcgi?db=gene&cmd=Retrieve&dopt=full_report&list_uids=93107) | potassium voltage-gated channel modifier subfamily G member 4 |
| [Details](https://mirdb.org/cgi-bin/target_detail.cgi?targetID=3253916) | 365 | 88 | hsa-miR-15a-5p | [HMGA1](http://www.ncbi.nlm.nih.gov/entrez/query.fcgi?db=gene&cmd=Retrieve&dopt=full_report&list_uids=3159) | high mobility group AT-hook 1 |
| [Details](https://mirdb.org/cgi-bin/target_detail.cgi?targetID=3252630) | 366 | 87 | hsa-miR-15a-5p | [SPTBN2](http://www.ncbi.nlm.nih.gov/entrez/query.fcgi?db=gene&cmd=Retrieve&dopt=full_report&list_uids=6712) | spectrin beta, non-erythrocytic 2 |
| [Details](https://mirdb.org/cgi-bin/target_detail.cgi?targetID=3252746) | 367 | 87 | hsa-miR-15a-5p | [SLC36A1](http://www.ncbi.nlm.nih.gov/entrez/query.fcgi?db=gene&cmd=Retrieve&dopt=full_report&list_uids=206358) | solute carrier family 36 member 1 |
| [Details](https://mirdb.org/cgi-bin/target_detail.cgi?targetID=3252823) | 368 | 87 | hsa-miR-15a-5p | [TENM2](http://www.ncbi.nlm.nih.gov/entrez/query.fcgi?db=gene&cmd=Retrieve&dopt=full_report&list_uids=57451) | teneurin transmembrane protein 2 |
| [Details](https://mirdb.org/cgi-bin/target_detail.cgi?targetID=3252863) | 369 | 87 | hsa-miR-15a-5p | [PDE3B](http://www.ncbi.nlm.nih.gov/entrez/query.fcgi?db=gene&cmd=Retrieve&dopt=full_report&list_uids=5140) | phosphodiesterase 3B |
| [Details](https://mirdb.org/cgi-bin/target_detail.cgi?targetID=3252864) | 370 | 87 | hsa-miR-15a-5p | [HTR4](http://www.ncbi.nlm.nih.gov/entrez/query.fcgi?db=gene&cmd=Retrieve&dopt=full_report&list_uids=3360) | 5-hydroxytryptamine receptor 4 |
| [Details](https://mirdb.org/cgi-bin/target_detail.cgi?targetID=3252867) | 371 | 87 | hsa-miR-15a-5p | [ATXN7L3](http://www.ncbi.nlm.nih.gov/entrez/query.fcgi?db=gene&cmd=Retrieve&dopt=full_report&list_uids=56970) | ataxin 7 like 3 |
| [Details](https://mirdb.org/cgi-bin/target_detail.cgi?targetID=3252893) | 372 | 87 | hsa-miR-15a-5p | [IKBKB](http://www.ncbi.nlm.nih.gov/entrez/query.fcgi?db=gene&cmd=Retrieve&dopt=full_report&list_uids=3551) | inhibitor of nuclear factor kappa B kinase subunit beta |
| [Details](https://mirdb.org/cgi-bin/target_detail.cgi?targetID=3252933) | 373 | 87 | hsa-miR-15a-5p | [CHUK](http://www.ncbi.nlm.nih.gov/entrez/query.fcgi?db=gene&cmd=Retrieve&dopt=full_report&list_uids=1147) | conserved helix-loop-helix ubiquitous kinase |
| [Details](https://mirdb.org/cgi-bin/target_detail.cgi?targetID=3252955) | 374 | 87 | hsa-miR-15a-5p | [USP44](http://www.ncbi.nlm.nih.gov/entrez/query.fcgi?db=gene&cmd=Retrieve&dopt=full_report&list_uids=84101) | ubiquitin specific peptidase 44 |
| [Details](https://mirdb.org/cgi-bin/target_detail.cgi?targetID=3252981) | 375 | 87 | hsa-miR-15a-5p | [WWC1](http://www.ncbi.nlm.nih.gov/entrez/query.fcgi?db=gene&cmd=Retrieve&dopt=full_report&list_uids=23286) | WW and C2 domain containing 1 |
| [Details](https://mirdb.org/cgi-bin/target_detail.cgi?targetID=3253129) | 376 | 87 | hsa-miR-15a-5p | [SIPA1L2](http://www.ncbi.nlm.nih.gov/entrez/query.fcgi?db=gene&cmd=Retrieve&dopt=full_report&list_uids=57568) | signal induced proliferation associated 1 like 2 |
| [Details](https://mirdb.org/cgi-bin/target_detail.cgi?targetID=3253158) | 377 | 87 | hsa-miR-15a-5p | [MCU](http://www.ncbi.nlm.nih.gov/entrez/query.fcgi?db=gene&cmd=Retrieve&dopt=full_report&list_uids=90550) | mitochondrial calcium uniporter |
| [Details](https://mirdb.org/cgi-bin/target_detail.cgi?targetID=3253216) | 378 | 87 | hsa-miR-15a-5p | [SSR1](http://www.ncbi.nlm.nih.gov/entrez/query.fcgi?db=gene&cmd=Retrieve&dopt=full_report&list_uids=6745) | signal sequence receptor subunit 1 |
| [Details](https://mirdb.org/cgi-bin/target_detail.cgi?targetID=3253253) | 379 | 87 | hsa-miR-15a-5p | [CUX1](http://www.ncbi.nlm.nih.gov/entrez/query.fcgi?db=gene&cmd=Retrieve&dopt=full_report&list_uids=1523) | cut like homeobox 1 |
| [Details](https://mirdb.org/cgi-bin/target_detail.cgi?targetID=3253270) | 380 | 87 | hsa-miR-15a-5p | [RUNDC3B](http://www.ncbi.nlm.nih.gov/entrez/query.fcgi?db=gene&cmd=Retrieve&dopt=full_report&list_uids=154661) | RUN domain containing 3B |
| [Details](https://mirdb.org/cgi-bin/target_detail.cgi?targetID=3253276) | 381 | 87 | hsa-miR-15a-5p | [PEX13](http://www.ncbi.nlm.nih.gov/entrez/query.fcgi?db=gene&cmd=Retrieve&dopt=full_report&list_uids=5194) | peroxisomal biogenesis factor 13 |
| [Details](https://mirdb.org/cgi-bin/target_detail.cgi?targetID=3253283) | 382 | 87 | hsa-miR-15a-5p | [UNC5D](http://www.ncbi.nlm.nih.gov/entrez/query.fcgi?db=gene&cmd=Retrieve&dopt=full_report&list_uids=137970) | unc-5 netrin receptor D |
| [Details](https://mirdb.org/cgi-bin/target_detail.cgi?targetID=3253351) | 383 | 87 | hsa-miR-15a-5p | [HEPHL1](http://www.ncbi.nlm.nih.gov/entrez/query.fcgi?db=gene&cmd=Retrieve&dopt=full_report&list_uids=341208) | hephaestin like 1 |
| [Details](https://mirdb.org/cgi-bin/target_detail.cgi?targetID=3253357) | 384 | 87 | hsa-miR-15a-5p | [SLC4A4](http://www.ncbi.nlm.nih.gov/entrez/query.fcgi?db=gene&cmd=Retrieve&dopt=full_report&list_uids=8671) | solute carrier family 4 member 4 |
| [Details](https://mirdb.org/cgi-bin/target_detail.cgi?targetID=3253411) | 385 | 87 | hsa-miR-15a-5p | [HECTD1](http://www.ncbi.nlm.nih.gov/entrez/query.fcgi?db=gene&cmd=Retrieve&dopt=full_report&list_uids=25831) | HECT domain E3 ubiquitin protein ligase 1 |
| [Details](https://mirdb.org/cgi-bin/target_detail.cgi?targetID=3253428) | 386 | 87 | hsa-miR-15a-5p | [SNX16](http://www.ncbi.nlm.nih.gov/entrez/query.fcgi?db=gene&cmd=Retrieve&dopt=full_report&list_uids=64089) | sorting nexin 16 |
| [Details](https://mirdb.org/cgi-bin/target_detail.cgi?targetID=3253478) | 387 | 87 | hsa-miR-15a-5p | [VPS4A](http://www.ncbi.nlm.nih.gov/entrez/query.fcgi?db=gene&cmd=Retrieve&dopt=full_report&list_uids=27183) | vacuolar protein sorting 4 homolog A |
| [Details](https://mirdb.org/cgi-bin/target_detail.cgi?targetID=3253538) | 388 | 87 | hsa-miR-15a-5p | [IVNS1ABP](http://www.ncbi.nlm.nih.gov/entrez/query.fcgi?db=gene&cmd=Retrieve&dopt=full_report&list_uids=10625) | influenza virus NS1A binding protein |
| [Details](https://mirdb.org/cgi-bin/target_detail.cgi?targetID=3253611) | 389 | 87 | hsa-miR-15a-5p | [MASP1](http://www.ncbi.nlm.nih.gov/entrez/query.fcgi?db=gene&cmd=Retrieve&dopt=full_report&list_uids=5648) | mannan binding lectin serine peptidase 1 |
| [Details](https://mirdb.org/cgi-bin/target_detail.cgi?targetID=3253617) | 390 | 87 | hsa-miR-15a-5p | [NAV1](http://www.ncbi.nlm.nih.gov/entrez/query.fcgi?db=gene&cmd=Retrieve&dopt=full_report&list_uids=89796) | neuron navigator 1 |
| [Details](https://mirdb.org/cgi-bin/target_detail.cgi?targetID=3253695) | 391 | 87 | hsa-miR-15a-5p | [RET](http://www.ncbi.nlm.nih.gov/entrez/query.fcgi?db=gene&cmd=Retrieve&dopt=full_report&list_uids=5979) | ret proto-oncogene |
| [Details](https://mirdb.org/cgi-bin/target_detail.cgi?targetID=3253771) | 392 | 87 | hsa-miR-15a-5p | [MTFR1L](http://www.ncbi.nlm.nih.gov/entrez/query.fcgi?db=gene&cmd=Retrieve&dopt=full_report&list_uids=56181) | mitochondrial fission regulator 1 like |
| [Details](https://mirdb.org/cgi-bin/target_detail.cgi?targetID=3253839) | 393 | 87 | hsa-miR-15a-5p | [RELN](http://www.ncbi.nlm.nih.gov/entrez/query.fcgi?db=gene&cmd=Retrieve&dopt=full_report&list_uids=5649) | reelin |
| [Details](https://mirdb.org/cgi-bin/target_detail.cgi?targetID=3253987) | 394 | 87 | hsa-miR-15a-5p | [DDX3Y](http://www.ncbi.nlm.nih.gov/entrez/query.fcgi?db=gene&cmd=Retrieve&dopt=full_report&list_uids=8653) | DEAD-box helicase 3 Y-linked |
| [Details](https://mirdb.org/cgi-bin/target_detail.cgi?targetID=3254034) | 395 | 87 | hsa-miR-15a-5p | [DEPDC4](http://www.ncbi.nlm.nih.gov/entrez/query.fcgi?db=gene&cmd=Retrieve&dopt=full_report&list_uids=120863) | DEP domain containing 4 |
| [Details](https://mirdb.org/cgi-bin/target_detail.cgi?targetID=3252637) | 396 | 86 | hsa-miR-15a-5p | [ANXA11](http://www.ncbi.nlm.nih.gov/entrez/query.fcgi?db=gene&cmd=Retrieve&dopt=full_report&list_uids=311) | annexin A11 |
| [Details](https://mirdb.org/cgi-bin/target_detail.cgi?targetID=3252695) | 397 | 86 | hsa-miR-15a-5p | [CLUH](http://www.ncbi.nlm.nih.gov/entrez/query.fcgi?db=gene&cmd=Retrieve&dopt=full_report&list_uids=23277) | clustered mitochondria homolog |
| [Details](https://mirdb.org/cgi-bin/target_detail.cgi?targetID=3252801) | 398 | 86 | hsa-miR-15a-5p | [RFK](http://www.ncbi.nlm.nih.gov/entrez/query.fcgi?db=gene&cmd=Retrieve&dopt=full_report&list_uids=55312) | riboflavin kinase |
| [Details](https://mirdb.org/cgi-bin/target_detail.cgi?targetID=3252804) | 399 | 86 | hsa-miR-15a-5p | [KPNA3](http://www.ncbi.nlm.nih.gov/entrez/query.fcgi?db=gene&cmd=Retrieve&dopt=full_report&list_uids=3839) | karyopherin subunit alpha 3 |
| [Details](https://mirdb.org/cgi-bin/target_detail.cgi?targetID=3252832) | 400 | 86 | hsa-miR-15a-5p | [ST8SIA3](http://www.ncbi.nlm.nih.gov/entrez/query.fcgi?db=gene&cmd=Retrieve&dopt=full_report&list_uids=51046) | ST8 alpha-N-acetyl-neuraminide alpha-2,8-sialyltransferase 3 |
| [Details](https://mirdb.org/cgi-bin/target_detail.cgi?targetID=3252860) | 401 | 86 | hsa-miR-15a-5p | [TARBP2](http://www.ncbi.nlm.nih.gov/entrez/query.fcgi?db=gene&cmd=Retrieve&dopt=full_report&list_uids=6895) | TARBP2, RISC loading complex RNA binding subunit |
| [Details](https://mirdb.org/cgi-bin/target_detail.cgi?targetID=3252901) | 402 | 86 | hsa-miR-15a-5p | [USP3](http://www.ncbi.nlm.nih.gov/entrez/query.fcgi?db=gene&cmd=Retrieve&dopt=full_report&list_uids=9960) | ubiquitin specific peptidase 3 |
| [Details](https://mirdb.org/cgi-bin/target_detail.cgi?targetID=3252913) | 403 | 86 | hsa-miR-15a-5p | [GALNT7](http://www.ncbi.nlm.nih.gov/entrez/query.fcgi?db=gene&cmd=Retrieve&dopt=full_report&list_uids=51809) | polypeptide N-acetylgalactosaminyltransferase 7 |
| [Details](https://mirdb.org/cgi-bin/target_detail.cgi?targetID=3253075) | 404 | 86 | hsa-miR-15a-5p | [LGR5](http://www.ncbi.nlm.nih.gov/entrez/query.fcgi?db=gene&cmd=Retrieve&dopt=full_report&list_uids=8549) | leucine rich repeat containing G protein-coupled receptor 5 |
| [Details](https://mirdb.org/cgi-bin/target_detail.cgi?targetID=3253080) | 405 | 86 | hsa-miR-15a-5p | [MYEF2](http://www.ncbi.nlm.nih.gov/entrez/query.fcgi?db=gene&cmd=Retrieve&dopt=full_report&list_uids=50804) | myelin expression factor 2 |
| [Details](https://mirdb.org/cgi-bin/target_detail.cgi?targetID=3253180) | 406 | 86 | hsa-miR-15a-5p | [YRDC](http://www.ncbi.nlm.nih.gov/entrez/query.fcgi?db=gene&cmd=Retrieve&dopt=full_report&list_uids=79693) | yrdC N6-threonylcarbamoyltransferase domain containing |
| [Details](https://mirdb.org/cgi-bin/target_detail.cgi?targetID=3253232) | 407 | 86 | hsa-miR-15a-5p | [ARHGAP12](http://www.ncbi.nlm.nih.gov/entrez/query.fcgi?db=gene&cmd=Retrieve&dopt=full_report&list_uids=94134) | Rho GTPase activating protein 12 |
| [Details](https://mirdb.org/cgi-bin/target_detail.cgi?targetID=3253307) | 408 | 86 | hsa-miR-15a-5p | [SLC39A10](http://www.ncbi.nlm.nih.gov/entrez/query.fcgi?db=gene&cmd=Retrieve&dopt=full_report&list_uids=57181) | solute carrier family 39 member 10 |
| [Details](https://mirdb.org/cgi-bin/target_detail.cgi?targetID=3253327) | 409 | 86 | hsa-miR-15a-5p | [KIF3B](http://www.ncbi.nlm.nih.gov/entrez/query.fcgi?db=gene&cmd=Retrieve&dopt=full_report&list_uids=9371) | kinesin family member 3B |
| [Details](https://mirdb.org/cgi-bin/target_detail.cgi?targetID=3253330) | 410 | 86 | hsa-miR-15a-5p | [SYPL1](http://www.ncbi.nlm.nih.gov/entrez/query.fcgi?db=gene&cmd=Retrieve&dopt=full_report&list_uids=6856) | synaptophysin like 1 |
| [Details](https://mirdb.org/cgi-bin/target_detail.cgi?targetID=3253346) | 411 | 86 | hsa-miR-15a-5p | [OGT](http://www.ncbi.nlm.nih.gov/entrez/query.fcgi?db=gene&cmd=Retrieve&dopt=full_report&list_uids=8473) | O-linked N-acetylglucosamine (GlcNAc) transferase |
| [Details](https://mirdb.org/cgi-bin/target_detail.cgi?targetID=3253373) | 412 | 86 | hsa-miR-15a-5p | [UNC13A](http://www.ncbi.nlm.nih.gov/entrez/query.fcgi?db=gene&cmd=Retrieve&dopt=full_report&list_uids=23025) | unc-13 homolog A |
| [Details](https://mirdb.org/cgi-bin/target_detail.cgi?targetID=3253382) | 413 | 86 | hsa-miR-15a-5p | [YWHAH](http://www.ncbi.nlm.nih.gov/entrez/query.fcgi?db=gene&cmd=Retrieve&dopt=full_report&list_uids=7533) | tyrosine 3-monooxygenase/tryptophan 5-monooxygenase activation protein eta |
| [Details](https://mirdb.org/cgi-bin/target_detail.cgi?targetID=3253406) | 414 | 86 | hsa-miR-15a-5p | [SOBP](http://www.ncbi.nlm.nih.gov/entrez/query.fcgi?db=gene&cmd=Retrieve&dopt=full_report&list_uids=55084) | sine oculis binding protein homolog |
| [Details](https://mirdb.org/cgi-bin/target_detail.cgi?targetID=3253419) | 415 | 86 | hsa-miR-15a-5p | [SLC35G1](http://www.ncbi.nlm.nih.gov/entrez/query.fcgi?db=gene&cmd=Retrieve&dopt=full_report&list_uids=159371) | solute carrier family 35 member G1 |
| [Details](https://mirdb.org/cgi-bin/target_detail.cgi?targetID=3253434) | 416 | 86 | hsa-miR-15a-5p | [GFAP](http://www.ncbi.nlm.nih.gov/entrez/query.fcgi?db=gene&cmd=Retrieve&dopt=full_report&list_uids=2670) | glial fibrillary acidic protein |
| [Details](https://mirdb.org/cgi-bin/target_detail.cgi?targetID=3253439) | 417 | 86 | hsa-miR-15a-5p | [PIP4P1](http://www.ncbi.nlm.nih.gov/entrez/query.fcgi?db=gene&cmd=Retrieve&dopt=full_report&list_uids=90809) | phosphatidylinositol-4,5-bisphosphate 4-phosphatase 1 |
| [Details](https://mirdb.org/cgi-bin/target_detail.cgi?targetID=3253486) | 418 | 86 | hsa-miR-15a-5p | [SPAG7](http://www.ncbi.nlm.nih.gov/entrez/query.fcgi?db=gene&cmd=Retrieve&dopt=full_report&list_uids=9552) | sperm associated antigen 7 |
| [Details](https://mirdb.org/cgi-bin/target_detail.cgi?targetID=3253488) | 419 | 86 | hsa-miR-15a-5p | [IARS](http://www.ncbi.nlm.nih.gov/entrez/query.fcgi?db=gene&cmd=Retrieve&dopt=full_report&list_uids=3376) | isoleucyl-tRNA synthetase |
| [Details](https://mirdb.org/cgi-bin/target_detail.cgi?targetID=3253500) | 420 | 86 | hsa-miR-15a-5p | [CDK8](http://www.ncbi.nlm.nih.gov/entrez/query.fcgi?db=gene&cmd=Retrieve&dopt=full_report&list_uids=1024) | cyclin dependent kinase 8 |
| [Details](https://mirdb.org/cgi-bin/target_detail.cgi?targetID=3253507) | 421 | 86 | hsa-miR-15a-5p | [CYP2S1](http://www.ncbi.nlm.nih.gov/entrez/query.fcgi?db=gene&cmd=Retrieve&dopt=full_report&list_uids=29785) | cytochrome P450 family 2 subfamily S member 1 |
| [Details](https://mirdb.org/cgi-bin/target_detail.cgi?targetID=3253510) | 422 | 86 | hsa-miR-15a-5p | [TUBA4A](http://www.ncbi.nlm.nih.gov/entrez/query.fcgi?db=gene&cmd=Retrieve&dopt=full_report&list_uids=7277) | tubulin alpha 4a |
| [Details](https://mirdb.org/cgi-bin/target_detail.cgi?targetID=3253514) | 423 | 86 | hsa-miR-15a-5p | [RSPO3](http://www.ncbi.nlm.nih.gov/entrez/query.fcgi?db=gene&cmd=Retrieve&dopt=full_report&list_uids=84870) | R-spondin 3 |
| [Details](https://mirdb.org/cgi-bin/target_detail.cgi?targetID=3253571) | 424 | 86 | hsa-miR-15a-5p | [DPY19L4](http://www.ncbi.nlm.nih.gov/entrez/query.fcgi?db=gene&cmd=Retrieve&dopt=full_report&list_uids=286148) | dpy-19 like 4 |
| [Details](https://mirdb.org/cgi-bin/target_detail.cgi?targetID=3253639) | 425 | 86 | hsa-miR-15a-5p | [COPS7B](http://www.ncbi.nlm.nih.gov/entrez/query.fcgi?db=gene&cmd=Retrieve&dopt=full_report&list_uids=64708) | COP9 signalosome subunit 7B |
| [Details](https://mirdb.org/cgi-bin/target_detail.cgi?targetID=3253646) | 426 | 86 | hsa-miR-15a-5p | [NF1](http://www.ncbi.nlm.nih.gov/entrez/query.fcgi?db=gene&cmd=Retrieve&dopt=full_report&list_uids=4763) | neurofibromin 1 |
| [Details](https://mirdb.org/cgi-bin/target_detail.cgi?targetID=3253649) | 427 | 86 | hsa-miR-15a-5p | [BCL2L2](http://www.ncbi.nlm.nih.gov/entrez/query.fcgi?db=gene&cmd=Retrieve&dopt=full_report&list_uids=599) | BCL2 like 2 |
| [Details](https://mirdb.org/cgi-bin/target_detail.cgi?targetID=3253666) | 428 | 86 | hsa-miR-15a-5p | [KCNN4](http://www.ncbi.nlm.nih.gov/entrez/query.fcgi?db=gene&cmd=Retrieve&dopt=full_report&list_uids=3783) | potassium calcium-activated channel subfamily N member 4 |
| [Details](https://mirdb.org/cgi-bin/target_detail.cgi?targetID=3253739) | 429 | 86 | hsa-miR-15a-5p | [SESN1](http://www.ncbi.nlm.nih.gov/entrez/query.fcgi?db=gene&cmd=Retrieve&dopt=full_report&list_uids=27244) | sestrin 1 |
| [Details](https://mirdb.org/cgi-bin/target_detail.cgi?targetID=3253774) | 430 | 86 | hsa-miR-15a-5p | [SON](http://www.ncbi.nlm.nih.gov/entrez/query.fcgi?db=gene&cmd=Retrieve&dopt=full_report&list_uids=6651) | SON DNA binding protein |
| [Details](https://mirdb.org/cgi-bin/target_detail.cgi?targetID=3253835) | 431 | 86 | hsa-miR-15a-5p | [NOB1](http://www.ncbi.nlm.nih.gov/entrez/query.fcgi?db=gene&cmd=Retrieve&dopt=full_report&list_uids=28987) | NIN1 (RPN12) binding protein 1 homolog |
| [Details](https://mirdb.org/cgi-bin/target_detail.cgi?targetID=3253843) | 432 | 86 | hsa-miR-15a-5p | [WBP11](http://www.ncbi.nlm.nih.gov/entrez/query.fcgi?db=gene&cmd=Retrieve&dopt=full_report&list_uids=51729) | WW domain binding protein 11 |
| [Details](https://mirdb.org/cgi-bin/target_detail.cgi?targetID=3253868) | 433 | 86 | hsa-miR-15a-5p | [DENND2C](http://www.ncbi.nlm.nih.gov/entrez/query.fcgi?db=gene&cmd=Retrieve&dopt=full_report&list_uids=163259) | DENN domain containing 2C |
| [Details](https://mirdb.org/cgi-bin/target_detail.cgi?targetID=3253917) | 434 | 86 | hsa-miR-15a-5p | [NUFIP2](http://www.ncbi.nlm.nih.gov/entrez/query.fcgi?db=gene&cmd=Retrieve&dopt=full_report&list_uids=57532) | nuclear FMR1 interacting protein 2 |
| [Details](https://mirdb.org/cgi-bin/target_detail.cgi?targetID=3252841) | 435 | 85 | hsa-miR-15a-5p | [ARHGAP20](http://www.ncbi.nlm.nih.gov/entrez/query.fcgi?db=gene&cmd=Retrieve&dopt=full_report&list_uids=57569) | Rho GTPase activating protein 20 |
| [Details](https://mirdb.org/cgi-bin/target_detail.cgi?targetID=3252891) | 436 | 85 | hsa-miR-15a-5p | [RAB9A](http://www.ncbi.nlm.nih.gov/entrez/query.fcgi?db=gene&cmd=Retrieve&dopt=full_report&list_uids=9367) | RAB9A, member RAS oncogene family |
| [Details](https://mirdb.org/cgi-bin/target_detail.cgi?targetID=3252896) | 437 | 85 | hsa-miR-15a-5p | [BTG2](http://www.ncbi.nlm.nih.gov/entrez/query.fcgi?db=gene&cmd=Retrieve&dopt=full_report&list_uids=7832) | BTG anti-proliferation factor 2 |
| [Details](https://mirdb.org/cgi-bin/target_detail.cgi?targetID=3252923) | 438 | 85 | hsa-miR-15a-5p | [RBM12](http://www.ncbi.nlm.nih.gov/entrez/query.fcgi?db=gene&cmd=Retrieve&dopt=full_report&list_uids=10137) | RNA binding motif protein 12 |
| [Details](https://mirdb.org/cgi-bin/target_detail.cgi?targetID=3252966) | 439 | 85 | hsa-miR-15a-5p | [ZNHIT6](http://www.ncbi.nlm.nih.gov/entrez/query.fcgi?db=gene&cmd=Retrieve&dopt=full_report&list_uids=54680) | zinc finger HIT-type containing 6 |
| [Details](https://mirdb.org/cgi-bin/target_detail.cgi?targetID=3253026) | 440 | 85 | hsa-miR-15a-5p | [RICTOR](http://www.ncbi.nlm.nih.gov/entrez/query.fcgi?db=gene&cmd=Retrieve&dopt=full_report&list_uids=253260) | RPTOR independent companion of MTOR complex 2 |
| [Details](https://mirdb.org/cgi-bin/target_detail.cgi?targetID=3253070) | 441 | 85 | hsa-miR-15a-5p | [CD80](http://www.ncbi.nlm.nih.gov/entrez/query.fcgi?db=gene&cmd=Retrieve&dopt=full_report&list_uids=941) | CD80 molecule |
| [Details](https://mirdb.org/cgi-bin/target_detail.cgi?targetID=3253095) | 442 | 85 | hsa-miR-15a-5p | [EPC1](http://www.ncbi.nlm.nih.gov/entrez/query.fcgi?db=gene&cmd=Retrieve&dopt=full_report&list_uids=80314) | enhancer of polycomb homolog 1 |
| [Details](https://mirdb.org/cgi-bin/target_detail.cgi?targetID=3253109) | 443 | 85 | hsa-miR-15a-5p | [FLT3](http://www.ncbi.nlm.nih.gov/entrez/query.fcgi?db=gene&cmd=Retrieve&dopt=full_report&list_uids=2322) | fms related tyrosine kinase 3 |
| [Details](https://mirdb.org/cgi-bin/target_detail.cgi?targetID=3253185) | 444 | 85 | hsa-miR-15a-5p | [STXBP1](http://www.ncbi.nlm.nih.gov/entrez/query.fcgi?db=gene&cmd=Retrieve&dopt=full_report&list_uids=6812) | syntaxin binding protein 1 |
| [Details](https://mirdb.org/cgi-bin/target_detail.cgi?targetID=3253192) | 445 | 85 | hsa-miR-15a-5p | [PDK4](http://www.ncbi.nlm.nih.gov/entrez/query.fcgi?db=gene&cmd=Retrieve&dopt=full_report&list_uids=5166) | pyruvate dehydrogenase kinase 4 |
| [Details](https://mirdb.org/cgi-bin/target_detail.cgi?targetID=3253261) | 446 | 85 | hsa-miR-15a-5p | [FAM110C](http://www.ncbi.nlm.nih.gov/entrez/query.fcgi?db=gene&cmd=Retrieve&dopt=full_report&list_uids=642273) | family with sequence similarity 110 member C |
| [Details](https://mirdb.org/cgi-bin/target_detail.cgi?targetID=3253370) | 447 | 85 | hsa-miR-15a-5p | [LRP2](http://www.ncbi.nlm.nih.gov/entrez/query.fcgi?db=gene&cmd=Retrieve&dopt=full_report&list_uids=4036) | LDL receptor related protein 2 |
| [Details](https://mirdb.org/cgi-bin/target_detail.cgi?targetID=3253381) | 448 | 85 | hsa-miR-15a-5p | [ACOX1](http://www.ncbi.nlm.nih.gov/entrez/query.fcgi?db=gene&cmd=Retrieve&dopt=full_report&list_uids=51) | acyl-CoA oxidase 1 |
| [Details](https://mirdb.org/cgi-bin/target_detail.cgi?targetID=3253442) | 449 | 85 | hsa-miR-15a-5p | [GCC2](http://www.ncbi.nlm.nih.gov/entrez/query.fcgi?db=gene&cmd=Retrieve&dopt=full_report&list_uids=9648) | GRIP and coiled-coil domain containing 2 |
| [Details](https://mirdb.org/cgi-bin/target_detail.cgi?targetID=3253477) | 450 | 85 | hsa-miR-15a-5p | [MAN2A2](http://www.ncbi.nlm.nih.gov/entrez/query.fcgi?db=gene&cmd=Retrieve&dopt=full_report&list_uids=4122) | mannosidase alpha class 2A member 2 |
| [Details](https://mirdb.org/cgi-bin/target_detail.cgi?targetID=3253534) | 451 | 85 | hsa-miR-15a-5p | [GPN1](http://www.ncbi.nlm.nih.gov/entrez/query.fcgi?db=gene&cmd=Retrieve&dopt=full_report&list_uids=11321) | GPN-loop GTPase 1 |
| [Details](https://mirdb.org/cgi-bin/target_detail.cgi?targetID=3253540) | 452 | 85 | hsa-miR-15a-5p | [SSTR3](http://www.ncbi.nlm.nih.gov/entrez/query.fcgi?db=gene&cmd=Retrieve&dopt=full_report&list_uids=6753) | somatostatin receptor 3 |
| [Details](https://mirdb.org/cgi-bin/target_detail.cgi?targetID=3253543) | 453 | 85 | hsa-miR-15a-5p | [RUNX1T1](http://www.ncbi.nlm.nih.gov/entrez/query.fcgi?db=gene&cmd=Retrieve&dopt=full_report&list_uids=862) | RUNX1 translocation partner 1 |
| [Details](https://mirdb.org/cgi-bin/target_detail.cgi?targetID=3253562) | 454 | 85 | hsa-miR-15a-5p | [TRAM1](http://www.ncbi.nlm.nih.gov/entrez/query.fcgi?db=gene&cmd=Retrieve&dopt=full_report&list_uids=23471) | translocation associated membrane protein 1 |
| [Details](https://mirdb.org/cgi-bin/target_detail.cgi?targetID=3253626) | 455 | 85 | hsa-miR-15a-5p | [OTX1](http://www.ncbi.nlm.nih.gov/entrez/query.fcgi?db=gene&cmd=Retrieve&dopt=full_report&list_uids=5013) | orthodenticle homeobox 1 |
| [Details](https://mirdb.org/cgi-bin/target_detail.cgi?targetID=3253725) | 456 | 85 | hsa-miR-15a-5p | [RAB30](http://www.ncbi.nlm.nih.gov/entrez/query.fcgi?db=gene&cmd=Retrieve&dopt=full_report&list_uids=27314) | RAB30, member RAS oncogene family |
| [Details](https://mirdb.org/cgi-bin/target_detail.cgi?targetID=3253788) | 457 | 85 | hsa-miR-15a-5p | [PELI2](http://www.ncbi.nlm.nih.gov/entrez/query.fcgi?db=gene&cmd=Retrieve&dopt=full_report&list_uids=57161) | pellino E3 ubiquitin protein ligase family member 2 |
| [Details](https://mirdb.org/cgi-bin/target_detail.cgi?targetID=3253795) | 458 | 85 | hsa-miR-15a-5p | [TMEM199](http://www.ncbi.nlm.nih.gov/entrez/query.fcgi?db=gene&cmd=Retrieve&dopt=full_report&list_uids=147007) | transmembrane protein 199 |
| [Details](https://mirdb.org/cgi-bin/target_detail.cgi?targetID=3254011) | 459 | 85 | hsa-miR-15a-5p | [NFATC3](http://www.ncbi.nlm.nih.gov/entrez/query.fcgi?db=gene&cmd=Retrieve&dopt=full_report&list_uids=4775) | nuclear factor of activated T cells 3 |
| [Details](https://mirdb.org/cgi-bin/target_detail.cgi?targetID=3252640) | 460 | 84 | hsa-miR-15a-5p | [MAP3K9](http://www.ncbi.nlm.nih.gov/entrez/query.fcgi?db=gene&cmd=Retrieve&dopt=full_report&list_uids=4293) | mitogen-activated protein kinase kinase kinase 9 |
| [Details](https://mirdb.org/cgi-bin/target_detail.cgi?targetID=3252661) | 461 | 84 | hsa-miR-15a-5p | [ELAC1](http://www.ncbi.nlm.nih.gov/entrez/query.fcgi?db=gene&cmd=Retrieve&dopt=full_report&list_uids=55520) | elaC ribonuclease Z 1 |
| [Details](https://mirdb.org/cgi-bin/target_detail.cgi?targetID=3252703) | 462 | 84 | hsa-miR-15a-5p | [ANKRD46](http://www.ncbi.nlm.nih.gov/entrez/query.fcgi?db=gene&cmd=Retrieve&dopt=full_report&list_uids=157567) | ankyrin repeat domain 46 |
| [Details](https://mirdb.org/cgi-bin/target_detail.cgi?targetID=3252719) | 463 | 84 | hsa-miR-15a-5p | [MOV10](http://www.ncbi.nlm.nih.gov/entrez/query.fcgi?db=gene&cmd=Retrieve&dopt=full_report&list_uids=4343) | Mov10 RISC complex RNA helicase |
| [Details](https://mirdb.org/cgi-bin/target_detail.cgi?targetID=3252748) | 464 | 84 | hsa-miR-15a-5p | [CAPZA2](http://www.ncbi.nlm.nih.gov/entrez/query.fcgi?db=gene&cmd=Retrieve&dopt=full_report&list_uids=830) | capping actin protein of muscle Z-line subunit alpha 2 |
| [Details](https://mirdb.org/cgi-bin/target_detail.cgi?targetID=3252789) | 465 | 84 | hsa-miR-15a-5p | [CREBRF](http://www.ncbi.nlm.nih.gov/entrez/query.fcgi?db=gene&cmd=Retrieve&dopt=full_report&list_uids=153222) | CREB3 regulatory factor |
| [Details](https://mirdb.org/cgi-bin/target_detail.cgi?targetID=3252854) | 466 | 84 | hsa-miR-15a-5p | [RBM24](http://www.ncbi.nlm.nih.gov/entrez/query.fcgi?db=gene&cmd=Retrieve&dopt=full_report&list_uids=221662) | RNA binding motif protein 24 |
| [Details](https://mirdb.org/cgi-bin/target_detail.cgi?targetID=3252874) | 467 | 84 | hsa-miR-15a-5p | [PLEKHA1](http://www.ncbi.nlm.nih.gov/entrez/query.fcgi?db=gene&cmd=Retrieve&dopt=full_report&list_uids=59338) | pleckstrin homology domain containing A1 |
| [Details](https://mirdb.org/cgi-bin/target_detail.cgi?targetID=3252919) | 468 | 84 | hsa-miR-15a-5p | [PTPRD](http://www.ncbi.nlm.nih.gov/entrez/query.fcgi?db=gene&cmd=Retrieve&dopt=full_report&list_uids=5789) | protein tyrosine phosphatase, receptor type D |
| [Details](https://mirdb.org/cgi-bin/target_detail.cgi?targetID=3253093) | 469 | 84 | hsa-miR-15a-5p | [NSG1](http://www.ncbi.nlm.nih.gov/entrez/query.fcgi?db=gene&cmd=Retrieve&dopt=full_report&list_uids=27065) | neuronal vesicle trafficking associated 1 |
| [Details](https://mirdb.org/cgi-bin/target_detail.cgi?targetID=3253251) | 470 | 84 | hsa-miR-15a-5p | [NCS1](http://www.ncbi.nlm.nih.gov/entrez/query.fcgi?db=gene&cmd=Retrieve&dopt=full_report&list_uids=23413) | neuronal calcium sensor 1 |
| [Details](https://mirdb.org/cgi-bin/target_detail.cgi?targetID=3253256) | 471 | 84 | hsa-miR-15a-5p | [ACVR2B](http://www.ncbi.nlm.nih.gov/entrez/query.fcgi?db=gene&cmd=Retrieve&dopt=full_report&list_uids=93) | activin A receptor type 2B |
| [Details](https://mirdb.org/cgi-bin/target_detail.cgi?targetID=3253258) | 472 | 84 | hsa-miR-15a-5p | [SAV1](http://www.ncbi.nlm.nih.gov/entrez/query.fcgi?db=gene&cmd=Retrieve&dopt=full_report&list_uids=60485) | salvador family WW domain containing protein 1 |
| [Details](https://mirdb.org/cgi-bin/target_detail.cgi?targetID=3253292) | 473 | 84 | hsa-miR-15a-5p | [GGA3](http://www.ncbi.nlm.nih.gov/entrez/query.fcgi?db=gene&cmd=Retrieve&dopt=full_report&list_uids=23163) | golgi associated, gamma adaptin ear containing, ARF binding protein 3 |
| [Details](https://mirdb.org/cgi-bin/target_detail.cgi?targetID=3253355) | 474 | 84 | hsa-miR-15a-5p | [RIMKLB](http://www.ncbi.nlm.nih.gov/entrez/query.fcgi?db=gene&cmd=Retrieve&dopt=full_report&list_uids=57494) | ribosomal modification protein rimK like family member B |
| [Details](https://mirdb.org/cgi-bin/target_detail.cgi?targetID=3253464) | 475 | 84 | hsa-miR-15a-5p | [ZDHHC15](http://www.ncbi.nlm.nih.gov/entrez/query.fcgi?db=gene&cmd=Retrieve&dopt=full_report&list_uids=158866) | zinc finger DHHC-type containing 15 |
| [Details](https://mirdb.org/cgi-bin/target_detail.cgi?targetID=3253541) | 476 | 84 | hsa-miR-15a-5p | [ZCCHC2](http://www.ncbi.nlm.nih.gov/entrez/query.fcgi?db=gene&cmd=Retrieve&dopt=full_report&list_uids=54877) | zinc finger CCHC-type containing 2 |
| [Details](https://mirdb.org/cgi-bin/target_detail.cgi?targetID=3253606) | 477 | 84 | hsa-miR-15a-5p | [SEH1L](http://www.ncbi.nlm.nih.gov/entrez/query.fcgi?db=gene&cmd=Retrieve&dopt=full_report&list_uids=81929) | SEH1 like nucleoporin |
| [Details](https://mirdb.org/cgi-bin/target_detail.cgi?targetID=3253609) | 478 | 84 | hsa-miR-15a-5p | [SNTB2](http://www.ncbi.nlm.nih.gov/entrez/query.fcgi?db=gene&cmd=Retrieve&dopt=full_report&list_uids=6645) | syntrophin beta 2 |
| [Details](https://mirdb.org/cgi-bin/target_detail.cgi?targetID=3253709) | 479 | 84 | hsa-miR-15a-5p | [CCDC88C](http://www.ncbi.nlm.nih.gov/entrez/query.fcgi?db=gene&cmd=Retrieve&dopt=full_report&list_uids=440193) | coiled-coil domain containing 88C |
| [Details](https://mirdb.org/cgi-bin/target_detail.cgi?targetID=3253775) | 480 | 84 | hsa-miR-15a-5p | [DENND4A](http://www.ncbi.nlm.nih.gov/entrez/query.fcgi?db=gene&cmd=Retrieve&dopt=full_report&list_uids=10260) | DENN domain containing 4A |
| [Details](https://mirdb.org/cgi-bin/target_detail.cgi?targetID=3253801) | 481 | 84 | hsa-miR-15a-5p | [DYNC1LI2](http://www.ncbi.nlm.nih.gov/entrez/query.fcgi?db=gene&cmd=Retrieve&dopt=full_report&list_uids=1783) | dynein cytoplasmic 1 light intermediate chain 2 |
| [Details](https://mirdb.org/cgi-bin/target_detail.cgi?targetID=3253816) | 482 | 84 | hsa-miR-15a-5p | [ABCF3](http://www.ncbi.nlm.nih.gov/entrez/query.fcgi?db=gene&cmd=Retrieve&dopt=full_report&list_uids=55324) | ATP binding cassette subfamily F member 3 |
| [Details](https://mirdb.org/cgi-bin/target_detail.cgi?targetID=3253846) | 483 | 84 | hsa-miR-15a-5p | [EXT2](http://www.ncbi.nlm.nih.gov/entrez/query.fcgi?db=gene&cmd=Retrieve&dopt=full_report&list_uids=2132) | exostosin glycosyltransferase 2 |
| [Details](https://mirdb.org/cgi-bin/target_detail.cgi?targetID=3253875) | 484 | 84 | hsa-miR-15a-5p | [ZBTB20](http://www.ncbi.nlm.nih.gov/entrez/query.fcgi?db=gene&cmd=Retrieve&dopt=full_report&list_uids=26137) | zinc finger and BTB domain containing 20 |
| [Details](https://mirdb.org/cgi-bin/target_detail.cgi?targetID=3253928) | 485 | 84 | hsa-miR-15a-5p | [LAMC1](http://www.ncbi.nlm.nih.gov/entrez/query.fcgi?db=gene&cmd=Retrieve&dopt=full_report&list_uids=3915) | laminin subunit gamma 1 |
| [Details](https://mirdb.org/cgi-bin/target_detail.cgi?targetID=3253933) | 486 | 84 | hsa-miR-15a-5p | [ARMCX2](http://www.ncbi.nlm.nih.gov/entrez/query.fcgi?db=gene&cmd=Retrieve&dopt=full_report&list_uids=9823) | armadillo repeat containing X-linked 2 |
| [Details](https://mirdb.org/cgi-bin/target_detail.cgi?targetID=3253975) | 487 | 84 | hsa-miR-15a-5p | [PPP6R3](http://www.ncbi.nlm.nih.gov/entrez/query.fcgi?db=gene&cmd=Retrieve&dopt=full_report&list_uids=55291) | protein phosphatase 6 regulatory subunit 3 |
| [Details](https://mirdb.org/cgi-bin/target_detail.cgi?targetID=3253995) | 488 | 84 | hsa-miR-15a-5p | [HELZ](http://www.ncbi.nlm.nih.gov/entrez/query.fcgi?db=gene&cmd=Retrieve&dopt=full_report&list_uids=9931) | helicase with zinc finger |
| [Details](https://mirdb.org/cgi-bin/target_detail.cgi?targetID=3254019) | 489 | 84 | hsa-miR-15a-5p | [NSMF](http://www.ncbi.nlm.nih.gov/entrez/query.fcgi?db=gene&cmd=Retrieve&dopt=full_report&list_uids=26012) | NMDA receptor synaptonuclear signaling and neuronal migration factor |
| [Details](https://mirdb.org/cgi-bin/target_detail.cgi?targetID=3252635) | 490 | 83 | hsa-miR-15a-5p | [HMBOX1](http://www.ncbi.nlm.nih.gov/entrez/query.fcgi?db=gene&cmd=Retrieve&dopt=full_report&list_uids=79618) | homeobox containing 1 |
| [Details](https://mirdb.org/cgi-bin/target_detail.cgi?targetID=3252688) | 491 | 83 | hsa-miR-15a-5p | [KCTD8](http://www.ncbi.nlm.nih.gov/entrez/query.fcgi?db=gene&cmd=Retrieve&dopt=full_report&list_uids=386617) | potassium channel tetramerization domain containing 8 |
| [Details](https://mirdb.org/cgi-bin/target_detail.cgi?targetID=3252707) | 492 | 83 | hsa-miR-15a-5p | [ZNF548](http://www.ncbi.nlm.nih.gov/entrez/query.fcgi?db=gene&cmd=Retrieve&dopt=full_report&list_uids=147694) | zinc finger protein 548 |
| [Details](https://mirdb.org/cgi-bin/target_detail.cgi?targetID=3252753) | 493 | 83 | hsa-miR-15a-5p | [PPT2](http://www.ncbi.nlm.nih.gov/entrez/query.fcgi?db=gene&cmd=Retrieve&dopt=full_report&list_uids=9374) | palmitoyl-protein thioesterase 2 |
| [Details](https://mirdb.org/cgi-bin/target_detail.cgi?targetID=3252767) | 494 | 83 | hsa-miR-15a-5p | [ATF6](http://www.ncbi.nlm.nih.gov/entrez/query.fcgi?db=gene&cmd=Retrieve&dopt=full_report&list_uids=22926) | activating transcription factor 6 |
| [Details](https://mirdb.org/cgi-bin/target_detail.cgi?targetID=3252783) | 495 | 83 | hsa-miR-15a-5p | [CARM1](http://www.ncbi.nlm.nih.gov/entrez/query.fcgi?db=gene&cmd=Retrieve&dopt=full_report&list_uids=10498) | coactivator associated arginine methyltransferase 1 |
| [Details](https://mirdb.org/cgi-bin/target_detail.cgi?targetID=3252818) | 496 | 83 | hsa-miR-15a-5p | [IHH](http://www.ncbi.nlm.nih.gov/entrez/query.fcgi?db=gene&cmd=Retrieve&dopt=full_report&list_uids=3549) | Indian hedgehog signaling molecule |
| [Details](https://mirdb.org/cgi-bin/target_detail.cgi?targetID=3252824) | 497 | 83 | hsa-miR-15a-5p | [GOLGA1](http://www.ncbi.nlm.nih.gov/entrez/query.fcgi?db=gene&cmd=Retrieve&dopt=full_report&list_uids=2800) | golgin A1 |
| [Details](https://mirdb.org/cgi-bin/target_detail.cgi?targetID=3252902) | 498 | 83 | hsa-miR-15a-5p | [TSC22D2](http://www.ncbi.nlm.nih.gov/entrez/query.fcgi?db=gene&cmd=Retrieve&dopt=full_report&list_uids=9819) | TSC22 domain family member 2 |
| [Details](https://mirdb.org/cgi-bin/target_detail.cgi?targetID=3252908) | 499 | 83 | hsa-miR-15a-5p | [UTP25](http://www.ncbi.nlm.nih.gov/entrez/query.fcgi?db=gene&cmd=Retrieve&dopt=full_report&list_uids=27042) | UTP25, small subunit processor component |
| [Details](https://mirdb.org/cgi-bin/target_detail.cgi?targetID=3252958) | 500 | 83 | hsa-miR-15a-5p | [CLDN12](http://www.ncbi.nlm.nih.gov/entrez/query.fcgi?db=gene&cmd=Retrieve&dopt=full_report&list_uids=9069) | claudin 12 |
| [Details](https://mirdb.org/cgi-bin/target_detail.cgi?targetID=3252999) | 501 | 83 | hsa-miR-15a-5p | [AGO1](http://www.ncbi.nlm.nih.gov/entrez/query.fcgi?db=gene&cmd=Retrieve&dopt=full_report&list_uids=26523) | argonaute RISC catalytic component 1 |
| [Details](https://mirdb.org/cgi-bin/target_detail.cgi?targetID=3253000) | 502 | 83 | hsa-miR-15a-5p | [EYA1](http://www.ncbi.nlm.nih.gov/entrez/query.fcgi?db=gene&cmd=Retrieve&dopt=full_report&list_uids=2138) | EYA transcriptional coactivator and phosphatase 1 |
| [Details](https://mirdb.org/cgi-bin/target_detail.cgi?targetID=3253065) | 503 | 83 | hsa-miR-15a-5p | [ATXN7L3B](http://www.ncbi.nlm.nih.gov/entrez/query.fcgi?db=gene&cmd=Retrieve&dopt=full_report&list_uids=552889) | ataxin 7 like 3B |
| [Details](https://mirdb.org/cgi-bin/target_detail.cgi?targetID=3253197) | 504 | 83 | hsa-miR-15a-5p | [CHIC1](http://www.ncbi.nlm.nih.gov/entrez/query.fcgi?db=gene&cmd=Retrieve&dopt=full_report&list_uids=53344) | cysteine rich hydrophobic domain 1 |
| [Details](https://mirdb.org/cgi-bin/target_detail.cgi?targetID=3253207) | 505 | 83 | hsa-miR-15a-5p | [SLC2A14](http://www.ncbi.nlm.nih.gov/entrez/query.fcgi?db=gene&cmd=Retrieve&dopt=full_report&list_uids=144195) | solute carrier family 2 member 14 |
| [Details](https://mirdb.org/cgi-bin/target_detail.cgi?targetID=3253211) | 506 | 83 | hsa-miR-15a-5p | [BCL7A](http://www.ncbi.nlm.nih.gov/entrez/query.fcgi?db=gene&cmd=Retrieve&dopt=full_report&list_uids=605) | BCL7A, BAF complex component |
| [Details](https://mirdb.org/cgi-bin/target_detail.cgi?targetID=3253304) | 507 | 83 | hsa-miR-15a-5p | [AMMECR1](http://www.ncbi.nlm.nih.gov/entrez/query.fcgi?db=gene&cmd=Retrieve&dopt=full_report&list_uids=9949) | Alport syndrome, mental retardation, midface hypoplasia and elliptocytosis chromosomal region gene 1 |
| [Details](https://mirdb.org/cgi-bin/target_detail.cgi?targetID=3253551) | 508 | 83 | hsa-miR-15a-5p | [LYPLA2](http://www.ncbi.nlm.nih.gov/entrez/query.fcgi?db=gene&cmd=Retrieve&dopt=full_report&list_uids=11313) | lysophospholipase 2 |
| [Details](https://mirdb.org/cgi-bin/target_detail.cgi?targetID=3253621) | 509 | 83 | hsa-miR-15a-5p | [NRBP1](http://www.ncbi.nlm.nih.gov/entrez/query.fcgi?db=gene&cmd=Retrieve&dopt=full_report&list_uids=29959) | nuclear receptor binding protein 1 |
| [Details](https://mirdb.org/cgi-bin/target_detail.cgi?targetID=3253726) | 510 | 83 | hsa-miR-15a-5p | [CMC4](http://www.ncbi.nlm.nih.gov/entrez/query.fcgi?db=gene&cmd=Retrieve&dopt=full_report&list_uids=100272147) | C-X9-C motif containing 4 |
| [Details](https://mirdb.org/cgi-bin/target_detail.cgi?targetID=3253799) | 511 | 83 | hsa-miR-15a-5p | [AREL1](http://www.ncbi.nlm.nih.gov/entrez/query.fcgi?db=gene&cmd=Retrieve&dopt=full_report&list_uids=9870) | apoptosis resistant E3 ubiquitin protein ligase 1 |
| [Details](https://mirdb.org/cgi-bin/target_detail.cgi?targetID=3253902) | 512 | 83 | hsa-miR-15a-5p | [HOXA10](http://www.ncbi.nlm.nih.gov/entrez/query.fcgi?db=gene&cmd=Retrieve&dopt=full_report&list_uids=3206) | homeobox A10 |
| [Details](https://mirdb.org/cgi-bin/target_detail.cgi?targetID=3253906) | 513 | 83 | hsa-miR-15a-5p | [SOX6](http://www.ncbi.nlm.nih.gov/entrez/query.fcgi?db=gene&cmd=Retrieve&dopt=full_report&list_uids=55553) | SRY-box 6 |
| [Details](https://mirdb.org/cgi-bin/target_detail.cgi?targetID=3253921) | 514 | 83 | hsa-miR-15a-5p | [LDLRAD2](http://www.ncbi.nlm.nih.gov/entrez/query.fcgi?db=gene&cmd=Retrieve&dopt=full_report&list_uids=401944) | low density lipoprotein receptor class A domain containing 2 |
| [Details](https://mirdb.org/cgi-bin/target_detail.cgi?targetID=3254002) | 515 | 83 | hsa-miR-15a-5p | [VTI1B](http://www.ncbi.nlm.nih.gov/entrez/query.fcgi?db=gene&cmd=Retrieve&dopt=full_report&list_uids=10490) | vesicle transport through interaction with t-SNAREs 1B |
| [Details](https://mirdb.org/cgi-bin/target_detail.cgi?targetID=3252657) | 516 | 82 | hsa-miR-15a-5p | [FAM122B](http://www.ncbi.nlm.nih.gov/entrez/query.fcgi?db=gene&cmd=Retrieve&dopt=full_report&list_uids=159090) | family with sequence similarity 122B |
| [Details](https://mirdb.org/cgi-bin/target_detail.cgi?targetID=3252715) | 517 | 82 | hsa-miR-15a-5p | [CCDC83](http://www.ncbi.nlm.nih.gov/entrez/query.fcgi?db=gene&cmd=Retrieve&dopt=full_report&list_uids=220047) | coiled-coil domain containing 83 |
| [Details](https://mirdb.org/cgi-bin/target_detail.cgi?targetID=3252795) | 518 | 82 | hsa-miR-15a-5p | [COBLL1](http://www.ncbi.nlm.nih.gov/entrez/query.fcgi?db=gene&cmd=Retrieve&dopt=full_report&list_uids=22837) | cordon-bleu WH2 repeat protein like 1 |
| [Details](https://mirdb.org/cgi-bin/target_detail.cgi?targetID=3252811) | 519 | 82 | hsa-miR-15a-5p | [SALL3](http://www.ncbi.nlm.nih.gov/entrez/query.fcgi?db=gene&cmd=Retrieve&dopt=full_report&list_uids=27164) | spalt like transcription factor 3 |
| [Details](https://mirdb.org/cgi-bin/target_detail.cgi?targetID=3252838) | 520 | 82 | hsa-miR-15a-5p | [LOC100130451](http://www.ncbi.nlm.nih.gov/entrez/query.fcgi?db=gene&cmd=Retrieve&dopt=full_report&list_uids=100130451) | uncharacterized LOC100130451 |
| [Details](https://mirdb.org/cgi-bin/target_detail.cgi?targetID=3252886) | 521 | 82 | hsa-miR-15a-5p | [FAM135A](http://www.ncbi.nlm.nih.gov/entrez/query.fcgi?db=gene&cmd=Retrieve&dopt=full_report&list_uids=57579) | family with sequence similarity 135 member A |
| [Details](https://mirdb.org/cgi-bin/target_detail.cgi?targetID=3252907) | 522 | 82 | hsa-miR-15a-5p | [ADAMTS6](http://www.ncbi.nlm.nih.gov/entrez/query.fcgi?db=gene&cmd=Retrieve&dopt=full_report&list_uids=11174) | ADAM metallopeptidase with thrombospondin type 1 motif 6 |
| [Details](https://mirdb.org/cgi-bin/target_detail.cgi?targetID=3253051) | 523 | 82 | hsa-miR-15a-5p | [TMEM74B](http://www.ncbi.nlm.nih.gov/entrez/query.fcgi?db=gene&cmd=Retrieve&dopt=full_report&list_uids=55321) | transmembrane protein 74B |
| [Details](https://mirdb.org/cgi-bin/target_detail.cgi?targetID=3253072) | 524 | 82 | hsa-miR-15a-5p | [SYT4](http://www.ncbi.nlm.nih.gov/entrez/query.fcgi?db=gene&cmd=Retrieve&dopt=full_report&list_uids=6860) | synaptotagmin 4 |
| [Details](https://mirdb.org/cgi-bin/target_detail.cgi?targetID=3253227) | 525 | 82 | hsa-miR-15a-5p | [IGF1R](http://www.ncbi.nlm.nih.gov/entrez/query.fcgi?db=gene&cmd=Retrieve&dopt=full_report&list_uids=3480) | insulin like growth factor 1 receptor |
| [Details](https://mirdb.org/cgi-bin/target_detail.cgi?targetID=3253250) | 526 | 82 | hsa-miR-15a-5p | [PLRG1](http://www.ncbi.nlm.nih.gov/entrez/query.fcgi?db=gene&cmd=Retrieve&dopt=full_report&list_uids=5356) | pleiotropic regulator 1 |
| [Details](https://mirdb.org/cgi-bin/target_detail.cgi?targetID=3253316) | 527 | 82 | hsa-miR-15a-5p | [IGF2R](http://www.ncbi.nlm.nih.gov/entrez/query.fcgi?db=gene&cmd=Retrieve&dopt=full_report&list_uids=3482) | insulin like growth factor 2 receptor |
| [Details](https://mirdb.org/cgi-bin/target_detail.cgi?targetID=3253325) | 528 | 82 | hsa-miR-15a-5p | [ARPP19](http://www.ncbi.nlm.nih.gov/entrez/query.fcgi?db=gene&cmd=Retrieve&dopt=full_report&list_uids=10776) | cAMP regulated phosphoprotein 19 |
| [Details](https://mirdb.org/cgi-bin/target_detail.cgi?targetID=3253476) | 529 | 82 | hsa-miR-15a-5p | [MYRIP](http://www.ncbi.nlm.nih.gov/entrez/query.fcgi?db=gene&cmd=Retrieve&dopt=full_report&list_uids=25924) | myosin VIIA and Rab interacting protein |
| [Details](https://mirdb.org/cgi-bin/target_detail.cgi?targetID=3253542) | 530 | 82 | hsa-miR-15a-5p | [ACSL4](http://www.ncbi.nlm.nih.gov/entrez/query.fcgi?db=gene&cmd=Retrieve&dopt=full_report&list_uids=2182) | acyl-CoA synthetase long chain family member 4 |
| [Details](https://mirdb.org/cgi-bin/target_detail.cgi?targetID=3253576) | 531 | 82 | hsa-miR-15a-5p | [CDC27](http://www.ncbi.nlm.nih.gov/entrez/query.fcgi?db=gene&cmd=Retrieve&dopt=full_report&list_uids=996) | cell division cycle 27 |
| [Details](https://mirdb.org/cgi-bin/target_detail.cgi?targetID=3253586) | 532 | 82 | hsa-miR-15a-5p | [UROS](http://www.ncbi.nlm.nih.gov/entrez/query.fcgi?db=gene&cmd=Retrieve&dopt=full_report&list_uids=7390) | uroporphyrinogen III synthase |
| [Details](https://mirdb.org/cgi-bin/target_detail.cgi?targetID=3253615) | 533 | 82 | hsa-miR-15a-5p | [NOTCH2](http://www.ncbi.nlm.nih.gov/entrez/query.fcgi?db=gene&cmd=Retrieve&dopt=full_report&list_uids=4853) | notch 2 |
| [Details](https://mirdb.org/cgi-bin/target_detail.cgi?targetID=3253664) | 534 | 82 | hsa-miR-15a-5p | [TRIP11](http://www.ncbi.nlm.nih.gov/entrez/query.fcgi?db=gene&cmd=Retrieve&dopt=full_report&list_uids=9321) | thyroid hormone receptor interactor 11 |
| [Details](https://mirdb.org/cgi-bin/target_detail.cgi?targetID=3253668) | 535 | 82 | hsa-miR-15a-5p | [SLC2A3](http://www.ncbi.nlm.nih.gov/entrez/query.fcgi?db=gene&cmd=Retrieve&dopt=full_report&list_uids=6515) | solute carrier family 2 member 3 |
| [Details](https://mirdb.org/cgi-bin/target_detail.cgi?targetID=3253672) | 536 | 82 | hsa-miR-15a-5p | [WNT4](http://www.ncbi.nlm.nih.gov/entrez/query.fcgi?db=gene&cmd=Retrieve&dopt=full_report&list_uids=54361) | Wnt family member 4 |
| [Details](https://mirdb.org/cgi-bin/target_detail.cgi?targetID=3253699) | 537 | 82 | hsa-miR-15a-5p | [ZNF275](http://www.ncbi.nlm.nih.gov/entrez/query.fcgi?db=gene&cmd=Retrieve&dopt=full_report&list_uids=10838) | zinc finger protein 275 |
| [Details](https://mirdb.org/cgi-bin/target_detail.cgi?targetID=3253730) | 538 | 82 | hsa-miR-15a-5p | [RSBN1](http://www.ncbi.nlm.nih.gov/entrez/query.fcgi?db=gene&cmd=Retrieve&dopt=full_report&list_uids=54665) | round spermatid basic protein 1 |
| [Details](https://mirdb.org/cgi-bin/target_detail.cgi?targetID=3253798) | 539 | 82 | hsa-miR-15a-5p | [BMPR1A](http://www.ncbi.nlm.nih.gov/entrez/query.fcgi?db=gene&cmd=Retrieve&dopt=full_report&list_uids=657) | bone morphogenetic protein receptor type 1A |
| [Details](https://mirdb.org/cgi-bin/target_detail.cgi?targetID=3253852) | 540 | 82 | hsa-miR-15a-5p | [CDHR1](http://www.ncbi.nlm.nih.gov/entrez/query.fcgi?db=gene&cmd=Retrieve&dopt=full_report&list_uids=92211) | cadherin related family member 1 |
| [Details](https://mirdb.org/cgi-bin/target_detail.cgi?targetID=3253913) | 541 | 82 | hsa-miR-15a-5p | [IL7R](http://www.ncbi.nlm.nih.gov/entrez/query.fcgi?db=gene&cmd=Retrieve&dopt=full_report&list_uids=3575) | interleukin 7 receptor |
| [Details](https://mirdb.org/cgi-bin/target_detail.cgi?targetID=3253924) | 542 | 82 | hsa-miR-15a-5p | [FAM89A](http://www.ncbi.nlm.nih.gov/entrez/query.fcgi?db=gene&cmd=Retrieve&dopt=full_report&list_uids=375061) | family with sequence similarity 89 member A |
| [Details](https://mirdb.org/cgi-bin/target_detail.cgi?targetID=3253989) | 543 | 82 | hsa-miR-15a-5p | [RTN4](http://www.ncbi.nlm.nih.gov/entrez/query.fcgi?db=gene&cmd=Retrieve&dopt=full_report&list_uids=57142) | reticulon 4 |
| [Details](https://mirdb.org/cgi-bin/target_detail.cgi?targetID=3254022) | 544 | 82 | hsa-miR-15a-5p | [ST7L](http://www.ncbi.nlm.nih.gov/entrez/query.fcgi?db=gene&cmd=Retrieve&dopt=full_report&list_uids=54879) | suppression of tumorigenicity 7 like |
| [Details](https://mirdb.org/cgi-bin/target_detail.cgi?targetID=3254024) | 545 | 82 | hsa-miR-15a-5p | [MAP7](http://www.ncbi.nlm.nih.gov/entrez/query.fcgi?db=gene&cmd=Retrieve&dopt=full_report&list_uids=9053) | microtubule associated protein 7 |
| [Details](https://mirdb.org/cgi-bin/target_detail.cgi?targetID=3254036) | 546 | 82 | hsa-miR-15a-5p | [SMURF2](http://www.ncbi.nlm.nih.gov/entrez/query.fcgi?db=gene&cmd=Retrieve&dopt=full_report&list_uids=64750) | SMAD specific E3 ubiquitin protein ligase 2 |
| [Details](https://mirdb.org/cgi-bin/target_detail.cgi?targetID=3252667) | 547 | 81 | hsa-miR-15a-5p | [TGIF2](http://www.ncbi.nlm.nih.gov/entrez/query.fcgi?db=gene&cmd=Retrieve&dopt=full_report&list_uids=60436) | TGFB induced factor homeobox 2 |
| [Details](https://mirdb.org/cgi-bin/target_detail.cgi?targetID=3252730) | 548 | 81 | hsa-miR-15a-5p | [RAD50](http://www.ncbi.nlm.nih.gov/entrez/query.fcgi?db=gene&cmd=Retrieve&dopt=full_report&list_uids=10111) | RAD50 double strand break repair protein |
| [Details](https://mirdb.org/cgi-bin/target_detail.cgi?targetID=3252831) | 549 | 81 | hsa-miR-15a-5p | [ELMOD1](http://www.ncbi.nlm.nih.gov/entrez/query.fcgi?db=gene&cmd=Retrieve&dopt=full_report&list_uids=55531) | ELMO domain containing 1 |
| [Details](https://mirdb.org/cgi-bin/target_detail.cgi?targetID=3252839) | 550 | 81 | hsa-miR-15a-5p | [MBNL2](http://www.ncbi.nlm.nih.gov/entrez/query.fcgi?db=gene&cmd=Retrieve&dopt=full_report&list_uids=10150) | muscleblind like splicing regulator 2 |
| [Details](https://mirdb.org/cgi-bin/target_detail.cgi?targetID=3252858) | 551 | 81 | hsa-miR-15a-5p | [FGF9](http://www.ncbi.nlm.nih.gov/entrez/query.fcgi?db=gene&cmd=Retrieve&dopt=full_report&list_uids=2254) | fibroblast growth factor 9 |
| [Details](https://mirdb.org/cgi-bin/target_detail.cgi?targetID=3252868) | 552 | 81 | hsa-miR-15a-5p | [CD3E](http://www.ncbi.nlm.nih.gov/entrez/query.fcgi?db=gene&cmd=Retrieve&dopt=full_report&list_uids=916) | CD3e molecule |
| [Details](https://mirdb.org/cgi-bin/target_detail.cgi?targetID=3252881) | 553 | 81 | hsa-miR-15a-5p | [MIB1](http://www.ncbi.nlm.nih.gov/entrez/query.fcgi?db=gene&cmd=Retrieve&dopt=full_report&list_uids=57534) | mindbomb E3 ubiquitin protein ligase 1 |
| [Details](https://mirdb.org/cgi-bin/target_detail.cgi?targetID=3252894) | 554 | 81 | hsa-miR-15a-5p | [C1QL3](http://www.ncbi.nlm.nih.gov/entrez/query.fcgi?db=gene&cmd=Retrieve&dopt=full_report&list_uids=389941) | complement C1q like 3 |
| [Details](https://mirdb.org/cgi-bin/target_detail.cgi?targetID=3252947) | 555 | 81 | hsa-miR-15a-5p | [DLEU7](http://www.ncbi.nlm.nih.gov/entrez/query.fcgi?db=gene&cmd=Retrieve&dopt=full_report&list_uids=220107) | deleted in lymphocytic leukemia 7 |
| [Details](https://mirdb.org/cgi-bin/target_detail.cgi?targetID=3253025) | 556 | 81 | hsa-miR-15a-5p | [TMEM135](http://www.ncbi.nlm.nih.gov/entrez/query.fcgi?db=gene&cmd=Retrieve&dopt=full_report&list_uids=65084) | transmembrane protein 135 |
| [Details](https://mirdb.org/cgi-bin/target_detail.cgi?targetID=3253139) | 557 | 81 | hsa-miR-15a-5p | [ISM2](http://www.ncbi.nlm.nih.gov/entrez/query.fcgi?db=gene&cmd=Retrieve&dopt=full_report&list_uids=145501) | isthmin 2 |
| [Details](https://mirdb.org/cgi-bin/target_detail.cgi?targetID=3253164) | 558 | 81 | hsa-miR-15a-5p | [ATG13](http://www.ncbi.nlm.nih.gov/entrez/query.fcgi?db=gene&cmd=Retrieve&dopt=full_report&list_uids=9776) | autophagy related 13 |
| [Details](https://mirdb.org/cgi-bin/target_detail.cgi?targetID=3253470) | 559 | 81 | hsa-miR-15a-5p | [RGMA](http://www.ncbi.nlm.nih.gov/entrez/query.fcgi?db=gene&cmd=Retrieve&dopt=full_report&list_uids=56963) | repulsive guidance molecule BMP co-receptor a |
| [Details](https://mirdb.org/cgi-bin/target_detail.cgi?targetID=3253491) | 560 | 81 | hsa-miR-15a-5p | [EPB41L4B](http://www.ncbi.nlm.nih.gov/entrez/query.fcgi?db=gene&cmd=Retrieve&dopt=full_report&list_uids=54566) | erythrocyte membrane protein band 4.1 like 4B |
| [Details](https://mirdb.org/cgi-bin/target_detail.cgi?targetID=3253530) | 561 | 81 | hsa-miR-15a-5p | [SH3GL2](http://www.ncbi.nlm.nih.gov/entrez/query.fcgi?db=gene&cmd=Retrieve&dopt=full_report&list_uids=6456) | SH3 domain containing GRB2 like 2, endophilin A1 |
| [Details](https://mirdb.org/cgi-bin/target_detail.cgi?targetID=3253565) | 562 | 81 | hsa-miR-15a-5p | [ANLN](http://www.ncbi.nlm.nih.gov/entrez/query.fcgi?db=gene&cmd=Retrieve&dopt=full_report&list_uids=54443) | anillin actin binding protein |
| [Details](https://mirdb.org/cgi-bin/target_detail.cgi?targetID=3253624) | 563 | 81 | hsa-miR-15a-5p | [SUZ12](http://www.ncbi.nlm.nih.gov/entrez/query.fcgi?db=gene&cmd=Retrieve&dopt=full_report&list_uids=23512) | SUZ12, polycomb repressive complex 2 subunit |
| [Details](https://mirdb.org/cgi-bin/target_detail.cgi?targetID=3253713) | 564 | 81 | hsa-miR-15a-5p | [FNTA](http://www.ncbi.nlm.nih.gov/entrez/query.fcgi?db=gene&cmd=Retrieve&dopt=full_report&list_uids=2339) | farnesyltransferase, CAAX box, alpha |
| [Details](https://mirdb.org/cgi-bin/target_detail.cgi?targetID=3253723) | 565 | 81 | hsa-miR-15a-5p | [G0S2](http://www.ncbi.nlm.nih.gov/entrez/query.fcgi?db=gene&cmd=Retrieve&dopt=full_report&list_uids=50486) | G0/G1 switch 2 |
| [Details](https://mirdb.org/cgi-bin/target_detail.cgi?targetID=3253817) | 566 | 81 | hsa-miR-15a-5p | [HECTD4](http://www.ncbi.nlm.nih.gov/entrez/query.fcgi?db=gene&cmd=Retrieve&dopt=full_report&list_uids=283450) | HECT domain E3 ubiquitin protein ligase 4 |
| [Details](https://mirdb.org/cgi-bin/target_detail.cgi?targetID=3253841) | 567 | 81 | hsa-miR-15a-5p | [TMEM154](http://www.ncbi.nlm.nih.gov/entrez/query.fcgi?db=gene&cmd=Retrieve&dopt=full_report&list_uids=201799) | transmembrane protein 154 |
| [Details](https://mirdb.org/cgi-bin/target_detail.cgi?targetID=3253854) | 568 | 81 | hsa-miR-15a-5p | [RIF1](http://www.ncbi.nlm.nih.gov/entrez/query.fcgi?db=gene&cmd=Retrieve&dopt=full_report&list_uids=55183) | replication timing regulatory factor 1 |
| [Details](https://mirdb.org/cgi-bin/target_detail.cgi?targetID=3253899) | 569 | 81 | hsa-miR-15a-5p | [SESTD1](http://www.ncbi.nlm.nih.gov/entrez/query.fcgi?db=gene&cmd=Retrieve&dopt=full_report&list_uids=91404) | SEC14 and spectrin domain containing 1 |
| [Details](https://mirdb.org/cgi-bin/target_detail.cgi?targetID=3253979) | 570 | 81 | hsa-miR-15a-5p | [RORA](http://www.ncbi.nlm.nih.gov/entrez/query.fcgi?db=gene&cmd=Retrieve&dopt=full_report&list_uids=6095) | RAR related orphan receptor A |
| [Details](https://mirdb.org/cgi-bin/target_detail.cgi?targetID=3253988) | 571 | 81 | hsa-miR-15a-5p | [CCNJL](http://www.ncbi.nlm.nih.gov/entrez/query.fcgi?db=gene&cmd=Retrieve&dopt=full_report&list_uids=79616) | cyclin J like |
| [Details](https://mirdb.org/cgi-bin/target_detail.cgi?targetID=3254012) | 572 | 81 | hsa-miR-15a-5p | [SEMA3D](http://www.ncbi.nlm.nih.gov/entrez/query.fcgi?db=gene&cmd=Retrieve&dopt=full_report&list_uids=223117) | semaphorin 3D |
| [Details](https://mirdb.org/cgi-bin/target_detail.cgi?targetID=3252747) | 573 | 80 | hsa-miR-15a-5p | [ELL2](http://www.ncbi.nlm.nih.gov/entrez/query.fcgi?db=gene&cmd=Retrieve&dopt=full_report&list_uids=22936) | elongation factor for RNA polymerase II 2 |
| [Details](https://mirdb.org/cgi-bin/target_detail.cgi?targetID=3252887) | 574 | 80 | hsa-miR-15a-5p | [ZC2HC1A](http://www.ncbi.nlm.nih.gov/entrez/query.fcgi?db=gene&cmd=Retrieve&dopt=full_report&list_uids=51101) | zinc finger C2HC-type containing 1A |
| [Details](https://mirdb.org/cgi-bin/target_detail.cgi?targetID=3252890) | 575 | 80 | hsa-miR-15a-5p | [PRKAR2A](http://www.ncbi.nlm.nih.gov/entrez/query.fcgi?db=gene&cmd=Retrieve&dopt=full_report&list_uids=5576) | protein kinase cAMP-dependent type II regulatory subunit alpha |
| [Details](https://mirdb.org/cgi-bin/target_detail.cgi?targetID=3252944) | 576 | 80 | hsa-miR-15a-5p | [MYT1L](http://www.ncbi.nlm.nih.gov/entrez/query.fcgi?db=gene&cmd=Retrieve&dopt=full_report&list_uids=23040) | myelin transcription factor 1 like |
| [Details](https://mirdb.org/cgi-bin/target_detail.cgi?targetID=3252961) | 577 | 80 | hsa-miR-15a-5p | [KIF5A](http://www.ncbi.nlm.nih.gov/entrez/query.fcgi?db=gene&cmd=Retrieve&dopt=full_report&list_uids=3798) | kinesin family member 5A |
| [Details](https://mirdb.org/cgi-bin/target_detail.cgi?targetID=3253014) | 578 | 80 | hsa-miR-15a-5p | [SAMD10](http://www.ncbi.nlm.nih.gov/entrez/query.fcgi?db=gene&cmd=Retrieve&dopt=full_report&list_uids=140700) | sterile alpha motif domain containing 10 |
| [Details](https://mirdb.org/cgi-bin/target_detail.cgi?targetID=3253053) | 579 | 80 | hsa-miR-15a-5p | [POLR3F](http://www.ncbi.nlm.nih.gov/entrez/query.fcgi?db=gene&cmd=Retrieve&dopt=full_report&list_uids=10621) | RNA polymerase III subunit F |
| [Details](https://mirdb.org/cgi-bin/target_detail.cgi?targetID=3253107) | 580 | 80 | hsa-miR-15a-5p | [UBR3](http://www.ncbi.nlm.nih.gov/entrez/query.fcgi?db=gene&cmd=Retrieve&dopt=full_report&list_uids=130507) | ubiquitin protein ligase E3 component n-recognin 3 |
| [Details](https://mirdb.org/cgi-bin/target_detail.cgi?targetID=3253116) | 581 | 80 | hsa-miR-15a-5p | [TTC14](http://www.ncbi.nlm.nih.gov/entrez/query.fcgi?db=gene&cmd=Retrieve&dopt=full_report&list_uids=151613) | tetratricopeptide repeat domain 14 |
| [Details](https://mirdb.org/cgi-bin/target_detail.cgi?targetID=3253265) | 582 | 80 | hsa-miR-15a-5p | [SVIP](http://www.ncbi.nlm.nih.gov/entrez/query.fcgi?db=gene&cmd=Retrieve&dopt=full_report&list_uids=258010) | small VCP interacting protein |
| [Details](https://mirdb.org/cgi-bin/target_detail.cgi?targetID=3253287) | 583 | 80 | hsa-miR-15a-5p | [PRDM11](http://www.ncbi.nlm.nih.gov/entrez/query.fcgi?db=gene&cmd=Retrieve&dopt=full_report&list_uids=56981) | PR/SET domain 11 |
| [Details](https://mirdb.org/cgi-bin/target_detail.cgi?targetID=3253379) | 584 | 80 | hsa-miR-15a-5p | [CNTNAP1](http://www.ncbi.nlm.nih.gov/entrez/query.fcgi?db=gene&cmd=Retrieve&dopt=full_report&list_uids=8506) | contactin associated protein 1 |
| [Details](https://mirdb.org/cgi-bin/target_detail.cgi?targetID=3253400) | 585 | 80 | hsa-miR-15a-5p | [CPSF7](http://www.ncbi.nlm.nih.gov/entrez/query.fcgi?db=gene&cmd=Retrieve&dopt=full_report&list_uids=79869) | cleavage and polyadenylation specific factor 7 |
| [Details](https://mirdb.org/cgi-bin/target_detail.cgi?targetID=3253587) | 586 | 80 | hsa-miR-15a-5p | [PTCH1](http://www.ncbi.nlm.nih.gov/entrez/query.fcgi?db=gene&cmd=Retrieve&dopt=full_report&list_uids=5727) | patched 1 |
| [Details](https://mirdb.org/cgi-bin/target_detail.cgi?targetID=3253607) | 587 | 80 | hsa-miR-15a-5p | [EML6](http://www.ncbi.nlm.nih.gov/entrez/query.fcgi?db=gene&cmd=Retrieve&dopt=full_report&list_uids=400954) | EMAP like 6 |
| [Details](https://mirdb.org/cgi-bin/target_detail.cgi?targetID=3253614) | 588 | 80 | hsa-miR-15a-5p | [PID1](http://www.ncbi.nlm.nih.gov/entrez/query.fcgi?db=gene&cmd=Retrieve&dopt=full_report&list_uids=55022) | phosphotyrosine interaction domain containing 1 |
| [Details](https://mirdb.org/cgi-bin/target_detail.cgi?targetID=3253674) | 589 | 80 | hsa-miR-15a-5p | [DNAJA2](http://www.ncbi.nlm.nih.gov/entrez/query.fcgi?db=gene&cmd=Retrieve&dopt=full_report&list_uids=10294) | DnaJ heat shock protein family (Hsp40) member A2 |
| [Details](https://mirdb.org/cgi-bin/target_detail.cgi?targetID=3253794) | 590 | 80 | hsa-miR-15a-5p | [SIAH1](http://www.ncbi.nlm.nih.gov/entrez/query.fcgi?db=gene&cmd=Retrieve&dopt=full_report&list_uids=6477) | siah E3 ubiquitin protein ligase 1 |
| [Details](https://mirdb.org/cgi-bin/target_detail.cgi?targetID=3253800) | 591 | 80 | hsa-miR-15a-5p | [LCOR](http://www.ncbi.nlm.nih.gov/entrez/query.fcgi?db=gene&cmd=Retrieve&dopt=full_report&list_uids=84458) | ligand dependent nuclear receptor corepressor |
| [Details](https://mirdb.org/cgi-bin/target_detail.cgi?targetID=3253952) | 592 | 80 | hsa-miR-15a-5p | [OTUD4](http://www.ncbi.nlm.nih.gov/entrez/query.fcgi?db=gene&cmd=Retrieve&dopt=full_report&list_uids=54726) | OTU deubiquitinase 4 |
| [Details](https://mirdb.org/cgi-bin/target_detail.cgi?targetID=3253955) | 593 | 80 | hsa-miR-15a-5p | [ENAH](http://www.ncbi.nlm.nih.gov/entrez/query.fcgi?db=gene&cmd=Retrieve&dopt=full_report&list_uids=55740) | ENAH, actin regulator |
| [Details](https://mirdb.org/cgi-bin/target_detail.cgi?targetID=3254035) | 594 | 80 | hsa-miR-15a-5p | [PHACTR2](http://www.ncbi.nlm.nih.gov/entrez/query.fcgi?db=gene&cmd=Retrieve&dopt=full_report&list_uids=9749) | phosphatase and actin regulator 2 |
| [Details](https://mirdb.org/cgi-bin/target_detail.cgi?targetID=3252658) | 595 | 79 | hsa-miR-15a-5p | [SLC35B2](http://www.ncbi.nlm.nih.gov/entrez/query.fcgi?db=gene&cmd=Retrieve&dopt=full_report&list_uids=347734) | solute carrier family 35 member B2 |
| [Details](https://mirdb.org/cgi-bin/target_detail.cgi?targetID=3252706) | 596 | 79 | hsa-miR-15a-5p | [KBTBD2](http://www.ncbi.nlm.nih.gov/entrez/query.fcgi?db=gene&cmd=Retrieve&dopt=full_report&list_uids=25948) | kelch repeat and BTB domain containing 2 |
| [Details](https://mirdb.org/cgi-bin/target_detail.cgi?targetID=3252711) | 597 | 79 | hsa-miR-15a-5p | [BCAP29](http://www.ncbi.nlm.nih.gov/entrez/query.fcgi?db=gene&cmd=Retrieve&dopt=full_report&list_uids=55973) | B cell receptor associated protein 29 |
| [Details](https://mirdb.org/cgi-bin/target_detail.cgi?targetID=3252736) | 598 | 79 | hsa-miR-15a-5p | [EIF3A](http://www.ncbi.nlm.nih.gov/entrez/query.fcgi?db=gene&cmd=Retrieve&dopt=full_report&list_uids=8661) | eukaryotic translation initiation factor 3 subunit A |
| [Details](https://mirdb.org/cgi-bin/target_detail.cgi?targetID=3252825) | 599 | 79 | hsa-miR-15a-5p | [HSPE1-MOB4](http://www.ncbi.nlm.nih.gov/entrez/query.fcgi?db=gene&cmd=Retrieve&dopt=full_report&list_uids=100529241) | HSPE1-MOB4 readthrough |
| [Details](https://mirdb.org/cgi-bin/target_detail.cgi?targetID=3252942) | 600 | 79 | hsa-miR-15a-5p | [MDN1](http://www.ncbi.nlm.nih.gov/entrez/query.fcgi?db=gene&cmd=Retrieve&dopt=full_report&list_uids=23195) | midasin AAA ATPase 1 |
| [Details](https://mirdb.org/cgi-bin/target_detail.cgi?targetID=3253084) | 601 | 79 | hsa-miR-15a-5p | [PSKH1](http://www.ncbi.nlm.nih.gov/entrez/query.fcgi?db=gene&cmd=Retrieve&dopt=full_report&list_uids=5681) | protein serine kinase H1 |
| [Details](https://mirdb.org/cgi-bin/target_detail.cgi?targetID=3253085) | 602 | 79 | hsa-miR-15a-5p | [LHX3](http://www.ncbi.nlm.nih.gov/entrez/query.fcgi?db=gene&cmd=Retrieve&dopt=full_report&list_uids=8022) | LIM homeobox 3 |
| [Details](https://mirdb.org/cgi-bin/target_detail.cgi?targetID=3253105) | 603 | 79 | hsa-miR-15a-5p | [SLC15A4](http://www.ncbi.nlm.nih.gov/entrez/query.fcgi?db=gene&cmd=Retrieve&dopt=full_report&list_uids=121260) | solute carrier family 15 member 4 |
| [Details](https://mirdb.org/cgi-bin/target_detail.cgi?targetID=3253177) | 604 | 79 | hsa-miR-15a-5p | [USP9X](http://www.ncbi.nlm.nih.gov/entrez/query.fcgi?db=gene&cmd=Retrieve&dopt=full_report&list_uids=8239) | ubiquitin specific peptidase 9 X-linked |
| [Details](https://mirdb.org/cgi-bin/target_detail.cgi?targetID=3253296) | 605 | 79 | hsa-miR-15a-5p | [NISCH](http://www.ncbi.nlm.nih.gov/entrez/query.fcgi?db=gene&cmd=Retrieve&dopt=full_report&list_uids=11188) | nischarin |
| [Details](https://mirdb.org/cgi-bin/target_detail.cgi?targetID=3253390) | 606 | 79 | hsa-miR-15a-5p | [ERC2](http://www.ncbi.nlm.nih.gov/entrez/query.fcgi?db=gene&cmd=Retrieve&dopt=full_report&list_uids=26059) | ELKS/RAB6-interacting/CAST family member 2 |
| [Details](https://mirdb.org/cgi-bin/target_detail.cgi?targetID=3253407) | 607 | 79 | hsa-miR-15a-5p | [PPIF](http://www.ncbi.nlm.nih.gov/entrez/query.fcgi?db=gene&cmd=Retrieve&dopt=full_report&list_uids=10105) | peptidylprolyl isomerase F |
| [Details](https://mirdb.org/cgi-bin/target_detail.cgi?targetID=3253447) | 608 | 79 | hsa-miR-15a-5p | [RAF1](http://www.ncbi.nlm.nih.gov/entrez/query.fcgi?db=gene&cmd=Retrieve&dopt=full_report&list_uids=5894) | Raf-1 proto-oncogene, serine/threonine kinase |
| [Details](https://mirdb.org/cgi-bin/target_detail.cgi?targetID=3253568) | 609 | 79 | hsa-miR-15a-5p | [SUSD6](http://www.ncbi.nlm.nih.gov/entrez/query.fcgi?db=gene&cmd=Retrieve&dopt=full_report&list_uids=9766) | sushi domain containing 6 |
| [Details](https://mirdb.org/cgi-bin/target_detail.cgi?targetID=3253667) | 610 | 79 | hsa-miR-15a-5p | [BCL2](http://www.ncbi.nlm.nih.gov/entrez/query.fcgi?db=gene&cmd=Retrieve&dopt=full_report&list_uids=596) | BCL2, apoptosis regulator |
| [Details](https://mirdb.org/cgi-bin/target_detail.cgi?targetID=3253823) | 611 | 79 | hsa-miR-15a-5p | [YAP1](http://www.ncbi.nlm.nih.gov/entrez/query.fcgi?db=gene&cmd=Retrieve&dopt=full_report&list_uids=10413) | Yes associated protein 1 |
| [Details](https://mirdb.org/cgi-bin/target_detail.cgi?targetID=3253961) | 612 | 79 | hsa-miR-15a-5p | [GNAI3](http://www.ncbi.nlm.nih.gov/entrez/query.fcgi?db=gene&cmd=Retrieve&dopt=full_report&list_uids=2773) | G protein subunit alpha i3 |
| [Details](https://mirdb.org/cgi-bin/target_detail.cgi?targetID=3253976) | 613 | 79 | hsa-miR-15a-5p | [ATP7A](http://www.ncbi.nlm.nih.gov/entrez/query.fcgi?db=gene&cmd=Retrieve&dopt=full_report&list_uids=538) | ATPase copper transporting alpha |
| [Details](https://mirdb.org/cgi-bin/target_detail.cgi?targetID=3253978) | 614 | 79 | hsa-miR-15a-5p | [KLHL18](http://www.ncbi.nlm.nih.gov/entrez/query.fcgi?db=gene&cmd=Retrieve&dopt=full_report&list_uids=23276) | kelch like family member 18 |
| [Details](https://mirdb.org/cgi-bin/target_detail.cgi?targetID=3252632) | 615 | 78 | hsa-miR-15a-5p | [ATP13A3](http://www.ncbi.nlm.nih.gov/entrez/query.fcgi?db=gene&cmd=Retrieve&dopt=full_report&list_uids=79572) | ATPase 13A3 |
| [Details](https://mirdb.org/cgi-bin/target_detail.cgi?targetID=3252659) | 616 | 78 | hsa-miR-15a-5p | [LOC390877](http://www.ncbi.nlm.nih.gov/entrez/query.fcgi?db=gene&cmd=Retrieve&dopt=full_report&list_uids=390877) | adenylate kinase isoenzyme 1-like |
| [Details](https://mirdb.org/cgi-bin/target_detail.cgi?targetID=3252666) | 617 | 78 | hsa-miR-15a-5p | [HMGA2](http://www.ncbi.nlm.nih.gov/entrez/query.fcgi?db=gene&cmd=Retrieve&dopt=full_report&list_uids=8091) | high mobility group AT-hook 2 |
| [Details](https://mirdb.org/cgi-bin/target_detail.cgi?targetID=3252757) | 618 | 78 | hsa-miR-15a-5p | [PLXNA2](http://www.ncbi.nlm.nih.gov/entrez/query.fcgi?db=gene&cmd=Retrieve&dopt=full_report&list_uids=5362) | plexin A2 |
| [Details](https://mirdb.org/cgi-bin/target_detail.cgi?targetID=3252773) | 619 | 78 | hsa-miR-15a-5p | [SLC1A2](http://www.ncbi.nlm.nih.gov/entrez/query.fcgi?db=gene&cmd=Retrieve&dopt=full_report&list_uids=6506) | solute carrier family 1 member 2 |
| [Details](https://mirdb.org/cgi-bin/target_detail.cgi?targetID=3252903) | 620 | 78 | hsa-miR-15a-5p | [ADAMTSL3](http://www.ncbi.nlm.nih.gov/entrez/query.fcgi?db=gene&cmd=Retrieve&dopt=full_report&list_uids=57188) | ADAMTS like 3 |
| [Details](https://mirdb.org/cgi-bin/target_detail.cgi?targetID=3252917) | 621 | 78 | hsa-miR-15a-5p | [MAPRE1](http://www.ncbi.nlm.nih.gov/entrez/query.fcgi?db=gene&cmd=Retrieve&dopt=full_report&list_uids=22919) | microtubule associated protein RP/EB family member 1 |
| [Details](https://mirdb.org/cgi-bin/target_detail.cgi?targetID=3252949) | 622 | 78 | hsa-miR-15a-5p | [C2orf72](http://www.ncbi.nlm.nih.gov/entrez/query.fcgi?db=gene&cmd=Retrieve&dopt=full_report&list_uids=257407) | chromosome 2 open reading frame 72 |
| [Details](https://mirdb.org/cgi-bin/target_detail.cgi?targetID=3252996) | 623 | 78 | hsa-miR-15a-5p | [RAB10](http://www.ncbi.nlm.nih.gov/entrez/query.fcgi?db=gene&cmd=Retrieve&dopt=full_report&list_uids=10890) | RAB10, member RAS oncogene family |
| [Details](https://mirdb.org/cgi-bin/target_detail.cgi?targetID=3253022) | 624 | 78 | hsa-miR-15a-5p | [GNAQ](http://www.ncbi.nlm.nih.gov/entrez/query.fcgi?db=gene&cmd=Retrieve&dopt=full_report&list_uids=2776) | G protein subunit alpha q |
| [Details](https://mirdb.org/cgi-bin/target_detail.cgi?targetID=3253033) | 625 | 78 | hsa-miR-15a-5p | [ENTPD1](http://www.ncbi.nlm.nih.gov/entrez/query.fcgi?db=gene&cmd=Retrieve&dopt=full_report&list_uids=953) | ectonucleoside triphosphate diphosphohydrolase 1 |
| [Details](https://mirdb.org/cgi-bin/target_detail.cgi?targetID=3253058) | 626 | 78 | hsa-miR-15a-5p | [FAM122A](http://www.ncbi.nlm.nih.gov/entrez/query.fcgi?db=gene&cmd=Retrieve&dopt=full_report&list_uids=116224) | family with sequence similarity 122A |
| [Details](https://mirdb.org/cgi-bin/target_detail.cgi?targetID=3253094) | 627 | 78 | hsa-miR-15a-5p | [SLC13A1](http://www.ncbi.nlm.nih.gov/entrez/query.fcgi?db=gene&cmd=Retrieve&dopt=full_report&list_uids=6561) | solute carrier family 13 member 1 |
| [Details](https://mirdb.org/cgi-bin/target_detail.cgi?targetID=3253190) | 628 | 78 | hsa-miR-15a-5p | [TMEM121B](http://www.ncbi.nlm.nih.gov/entrez/query.fcgi?db=gene&cmd=Retrieve&dopt=full_report&list_uids=27439) | transmembrane protein 121B |
| [Details](https://mirdb.org/cgi-bin/target_detail.cgi?targetID=3253220) | 629 | 78 | hsa-miR-15a-5p | [MIGA1](http://www.ncbi.nlm.nih.gov/entrez/query.fcgi?db=gene&cmd=Retrieve&dopt=full_report&list_uids=374986) | mitoguardin 1 |
| [Details](https://mirdb.org/cgi-bin/target_detail.cgi?targetID=3253229) | 630 | 78 | hsa-miR-15a-5p | [RTF1](http://www.ncbi.nlm.nih.gov/entrez/query.fcgi?db=gene&cmd=Retrieve&dopt=full_report&list_uids=23168) | RTF1 homolog, Paf1/RNA polymerase II complex component |
| [Details](https://mirdb.org/cgi-bin/target_detail.cgi?targetID=3253233) | 631 | 78 | hsa-miR-15a-5p | [SCAI](http://www.ncbi.nlm.nih.gov/entrez/query.fcgi?db=gene&cmd=Retrieve&dopt=full_report&list_uids=286205) | suppressor of cancer cell invasion |
| [Details](https://mirdb.org/cgi-bin/target_detail.cgi?targetID=3253315) | 632 | 78 | hsa-miR-15a-5p | [CBFA2T3](http://www.ncbi.nlm.nih.gov/entrez/query.fcgi?db=gene&cmd=Retrieve&dopt=full_report&list_uids=863) | CBFA2/RUNX1 translocation partner 3 |
| [Details](https://mirdb.org/cgi-bin/target_detail.cgi?targetID=3253331) | 633 | 78 | hsa-miR-15a-5p | [POM121C](http://www.ncbi.nlm.nih.gov/entrez/query.fcgi?db=gene&cmd=Retrieve&dopt=full_report&list_uids=100101267) | POM121 transmembrane nucleoporin C |
| [Details](https://mirdb.org/cgi-bin/target_detail.cgi?targetID=3253335) | 634 | 78 | hsa-miR-15a-5p | [UHMK1](http://www.ncbi.nlm.nih.gov/entrez/query.fcgi?db=gene&cmd=Retrieve&dopt=full_report&list_uids=127933) | U2AF homology motif kinase 1 |
| [Details](https://mirdb.org/cgi-bin/target_detail.cgi?targetID=3253359) | 635 | 78 | hsa-miR-15a-5p | [OSCP1](http://www.ncbi.nlm.nih.gov/entrez/query.fcgi?db=gene&cmd=Retrieve&dopt=full_report&list_uids=127700) | organic solute carrier partner 1 |
| [Details](https://mirdb.org/cgi-bin/target_detail.cgi?targetID=3253396) | 636 | 78 | hsa-miR-15a-5p | [CAMSAP1](http://www.ncbi.nlm.nih.gov/entrez/query.fcgi?db=gene&cmd=Retrieve&dopt=full_report&list_uids=157922) | calmodulin regulated spectrin associated protein 1 |
| [Details](https://mirdb.org/cgi-bin/target_detail.cgi?targetID=3253450) | 637 | 78 | hsa-miR-15a-5p | [KIF1C](http://www.ncbi.nlm.nih.gov/entrez/query.fcgi?db=gene&cmd=Retrieve&dopt=full_report&list_uids=10749) | kinesin family member 1C |
| [Details](https://mirdb.org/cgi-bin/target_detail.cgi?targetID=3253480) | 638 | 78 | hsa-miR-15a-5p | [NEDD9](http://www.ncbi.nlm.nih.gov/entrez/query.fcgi?db=gene&cmd=Retrieve&dopt=full_report&list_uids=4739) | neural precursor cell expressed, developmentally down-regulated 9 |
| [Details](https://mirdb.org/cgi-bin/target_detail.cgi?targetID=3253519) | 639 | 78 | hsa-miR-15a-5p | [RAB40AL](http://www.ncbi.nlm.nih.gov/entrez/query.fcgi?db=gene&cmd=Retrieve&dopt=full_report&list_uids=282808) | RAB40A like |
| [Details](https://mirdb.org/cgi-bin/target_detail.cgi?targetID=3253533) | 640 | 78 | hsa-miR-15a-5p | [PTAR1](http://www.ncbi.nlm.nih.gov/entrez/query.fcgi?db=gene&cmd=Retrieve&dopt=full_report&list_uids=375743) | protein prenyltransferase alpha subunit repeat containing 1 |
| [Details](https://mirdb.org/cgi-bin/target_detail.cgi?targetID=3253598) | 641 | 78 | hsa-miR-15a-5p | [NUAK2](http://www.ncbi.nlm.nih.gov/entrez/query.fcgi?db=gene&cmd=Retrieve&dopt=full_report&list_uids=81788) | NUAK family kinase 2 |
| [Details](https://mirdb.org/cgi-bin/target_detail.cgi?targetID=3253603) | 642 | 78 | hsa-miR-15a-5p | [RIC1](http://www.ncbi.nlm.nih.gov/entrez/query.fcgi?db=gene&cmd=Retrieve&dopt=full_report&list_uids=57589) | RIC1 homolog, RAB6A GEF complex partner 1 |
| [Details](https://mirdb.org/cgi-bin/target_detail.cgi?targetID=3253618) | 643 | 78 | hsa-miR-15a-5p | [MMD](http://www.ncbi.nlm.nih.gov/entrez/query.fcgi?db=gene&cmd=Retrieve&dopt=full_report&list_uids=23531) | monocyte to macrophage differentiation associated |
| [Details](https://mirdb.org/cgi-bin/target_detail.cgi?targetID=3253633) | 644 | 78 | hsa-miR-15a-5p | [SRPK1](http://www.ncbi.nlm.nih.gov/entrez/query.fcgi?db=gene&cmd=Retrieve&dopt=full_report&list_uids=6732) | SRSF protein kinase 1 |
| [Details](https://mirdb.org/cgi-bin/target_detail.cgi?targetID=3253657) | 645 | 78 | hsa-miR-15a-5p | [CCDC85C](http://www.ncbi.nlm.nih.gov/entrez/query.fcgi?db=gene&cmd=Retrieve&dopt=full_report&list_uids=317762) | coiled-coil domain containing 85C |
| [Details](https://mirdb.org/cgi-bin/target_detail.cgi?targetID=3253831) | 646 | 78 | hsa-miR-15a-5p | [ZSWIM3](http://www.ncbi.nlm.nih.gov/entrez/query.fcgi?db=gene&cmd=Retrieve&dopt=full_report&list_uids=140831) | zinc finger SWIM-type containing 3 |
| [Details](https://mirdb.org/cgi-bin/target_detail.cgi?targetID=3253840) | 647 | 78 | hsa-miR-15a-5p | [PDIK1L](http://www.ncbi.nlm.nih.gov/entrez/query.fcgi?db=gene&cmd=Retrieve&dopt=full_report&list_uids=149420) | PDLIM1 interacting kinase 1 like |
| [Details](https://mirdb.org/cgi-bin/target_detail.cgi?targetID=3253886) | 648 | 78 | hsa-miR-15a-5p | [PLS1](http://www.ncbi.nlm.nih.gov/entrez/query.fcgi?db=gene&cmd=Retrieve&dopt=full_report&list_uids=5357) | plastin 1 |
| [Details](https://mirdb.org/cgi-bin/target_detail.cgi?targetID=3253890) | 649 | 78 | hsa-miR-15a-5p | [CREBL2](http://www.ncbi.nlm.nih.gov/entrez/query.fcgi?db=gene&cmd=Retrieve&dopt=full_report&list_uids=1389) | cAMP responsive element binding protein like 2 |
| [Details](https://mirdb.org/cgi-bin/target_detail.cgi?targetID=3252646) | 650 | 77 | hsa-miR-15a-5p | [RAPH1](http://www.ncbi.nlm.nih.gov/entrez/query.fcgi?db=gene&cmd=Retrieve&dopt=full_report&list_uids=65059) | Ras association (RalGDS/AF-6) and pleckstrin homology domains 1 |
| [Details](https://mirdb.org/cgi-bin/target_detail.cgi?targetID=3252681) | 651 | 77 | hsa-miR-15a-5p | [GLRX](http://www.ncbi.nlm.nih.gov/entrez/query.fcgi?db=gene&cmd=Retrieve&dopt=full_report&list_uids=2745) | glutaredoxin |
| [Details](https://mirdb.org/cgi-bin/target_detail.cgi?targetID=3252716) | 652 | 77 | hsa-miR-15a-5p | [PTPRJ](http://www.ncbi.nlm.nih.gov/entrez/query.fcgi?db=gene&cmd=Retrieve&dopt=full_report&list_uids=5795) | protein tyrosine phosphatase, receptor type J |
| [Details](https://mirdb.org/cgi-bin/target_detail.cgi?targetID=3252790) | 653 | 77 | hsa-miR-15a-5p | [TRMT9B](http://www.ncbi.nlm.nih.gov/entrez/query.fcgi?db=gene&cmd=Retrieve&dopt=full_report&list_uids=57604) | tRNA methyltransferase 9B (putative) |
| [Details](https://mirdb.org/cgi-bin/target_detail.cgi?targetID=3252816) | 654 | 77 | hsa-miR-15a-5p | [SLC6A4](http://www.ncbi.nlm.nih.gov/entrez/query.fcgi?db=gene&cmd=Retrieve&dopt=full_report&list_uids=6532) | solute carrier family 6 member 4 |
| [Details](https://mirdb.org/cgi-bin/target_detail.cgi?targetID=3252861) | 655 | 77 | hsa-miR-15a-5p | [CALU](http://www.ncbi.nlm.nih.gov/entrez/query.fcgi?db=gene&cmd=Retrieve&dopt=full_report&list_uids=813) | calumenin |
| [Details](https://mirdb.org/cgi-bin/target_detail.cgi?targetID=3252895) | 656 | 77 | hsa-miR-15a-5p | [SLC39A9](http://www.ncbi.nlm.nih.gov/entrez/query.fcgi?db=gene&cmd=Retrieve&dopt=full_report&list_uids=55334) | solute carrier family 39 member 9 |
| [Details](https://mirdb.org/cgi-bin/target_detail.cgi?targetID=3252948) | 657 | 77 | hsa-miR-15a-5p | [CCND3](http://www.ncbi.nlm.nih.gov/entrez/query.fcgi?db=gene&cmd=Retrieve&dopt=full_report&list_uids=896) | cyclin D3 |
| [Details](https://mirdb.org/cgi-bin/target_detail.cgi?targetID=3252972) | 658 | 77 | hsa-miR-15a-5p | [TOX3](http://www.ncbi.nlm.nih.gov/entrez/query.fcgi?db=gene&cmd=Retrieve&dopt=full_report&list_uids=27324) | TOX high mobility group box family member 3 |
| [Details](https://mirdb.org/cgi-bin/target_detail.cgi?targetID=3253064) | 659 | 77 | hsa-miR-15a-5p | [CLDN2](http://www.ncbi.nlm.nih.gov/entrez/query.fcgi?db=gene&cmd=Retrieve&dopt=full_report&list_uids=9075) | claudin 2 |
| [Details](https://mirdb.org/cgi-bin/target_detail.cgi?targetID=3253100) | 660 | 77 | hsa-miR-15a-5p | [DHRS7](http://www.ncbi.nlm.nih.gov/entrez/query.fcgi?db=gene&cmd=Retrieve&dopt=full_report&list_uids=51635) | dehydrogenase/reductase 7 |
| [Details](https://mirdb.org/cgi-bin/target_detail.cgi?targetID=3253104) | 661 | 77 | hsa-miR-15a-5p | [ESRRA](http://www.ncbi.nlm.nih.gov/entrez/query.fcgi?db=gene&cmd=Retrieve&dopt=full_report&list_uids=2101) | estrogen related receptor alpha |
| [Details](https://mirdb.org/cgi-bin/target_detail.cgi?targetID=3253137) | 662 | 77 | hsa-miR-15a-5p | [TFAP2D](http://www.ncbi.nlm.nih.gov/entrez/query.fcgi?db=gene&cmd=Retrieve&dopt=full_report&list_uids=83741) | transcription factor AP-2 delta |
| [Details](https://mirdb.org/cgi-bin/target_detail.cgi?targetID=3253219) | 663 | 77 | hsa-miR-15a-5p | [USP12](http://www.ncbi.nlm.nih.gov/entrez/query.fcgi?db=gene&cmd=Retrieve&dopt=full_report&list_uids=219333) | ubiquitin specific peptidase 12 |
| [Details](https://mirdb.org/cgi-bin/target_detail.cgi?targetID=3253247) | 664 | 77 | hsa-miR-15a-5p | [FAT4](http://www.ncbi.nlm.nih.gov/entrez/query.fcgi?db=gene&cmd=Retrieve&dopt=full_report&list_uids=79633) | FAT atypical cadherin 4 |
| [Details](https://mirdb.org/cgi-bin/target_detail.cgi?targetID=3253465) | 665 | 77 | hsa-miR-15a-5p | [SNRK](http://www.ncbi.nlm.nih.gov/entrez/query.fcgi?db=gene&cmd=Retrieve&dopt=full_report&list_uids=54861) | SNF related kinase |
| [Details](https://mirdb.org/cgi-bin/target_detail.cgi?targetID=3253577) | 666 | 77 | hsa-miR-15a-5p | [IRAK2](http://www.ncbi.nlm.nih.gov/entrez/query.fcgi?db=gene&cmd=Retrieve&dopt=full_report&list_uids=3656) | interleukin 1 receptor associated kinase 2 |
| [Details](https://mirdb.org/cgi-bin/target_detail.cgi?targetID=3253698) | 667 | 77 | hsa-miR-15a-5p | [CARD10](http://www.ncbi.nlm.nih.gov/entrez/query.fcgi?db=gene&cmd=Retrieve&dopt=full_report&list_uids=29775) | caspase recruitment domain family member 10 |
| [Details](https://mirdb.org/cgi-bin/target_detail.cgi?targetID=3253702) | 668 | 77 | hsa-miR-15a-5p | [BOLA3](http://www.ncbi.nlm.nih.gov/entrez/query.fcgi?db=gene&cmd=Retrieve&dopt=full_report&list_uids=388962) | bolA family member 3 |
| [Details](https://mirdb.org/cgi-bin/target_detail.cgi?targetID=3253718) | 669 | 77 | hsa-miR-15a-5p | [KCNQ5](http://www.ncbi.nlm.nih.gov/entrez/query.fcgi?db=gene&cmd=Retrieve&dopt=full_report&list_uids=56479) | potassium voltage-gated channel subfamily Q member 5 |
| [Details](https://mirdb.org/cgi-bin/target_detail.cgi?targetID=3253873) | 670 | 77 | hsa-miR-15a-5p | [MLYCD](http://www.ncbi.nlm.nih.gov/entrez/query.fcgi?db=gene&cmd=Retrieve&dopt=full_report&list_uids=23417) | malonyl-CoA decarboxylase |
| [Details](https://mirdb.org/cgi-bin/target_detail.cgi?targetID=3252819) | 671 | 76 | hsa-miR-15a-5p | [ZNF705E](http://www.ncbi.nlm.nih.gov/entrez/query.fcgi?db=gene&cmd=Retrieve&dopt=full_report&list_uids=100131539) | zinc finger protein 705E |
| [Details](https://mirdb.org/cgi-bin/target_detail.cgi?targetID=3252836) | 672 | 76 | hsa-miR-15a-5p | [C16orf72](http://www.ncbi.nlm.nih.gov/entrez/query.fcgi?db=gene&cmd=Retrieve&dopt=full_report&list_uids=29035) | chromosome 16 open reading frame 72 |
| [Details](https://mirdb.org/cgi-bin/target_detail.cgi?targetID=3252922) | 673 | 76 | hsa-miR-15a-5p | [FKBP1A](http://www.ncbi.nlm.nih.gov/entrez/query.fcgi?db=gene&cmd=Retrieve&dopt=full_report&list_uids=2280) | FKBP prolyl isomerase 1A |
| [Details](https://mirdb.org/cgi-bin/target_detail.cgi?targetID=3252952) | 674 | 76 | hsa-miR-15a-5p | [KCNU1](http://www.ncbi.nlm.nih.gov/entrez/query.fcgi?db=gene&cmd=Retrieve&dopt=full_report&list_uids=157855) | potassium calcium-activated channel subfamily U member 1 |
| [Details](https://mirdb.org/cgi-bin/target_detail.cgi?targetID=3252970) | 675 | 76 | hsa-miR-15a-5p | [IST1](http://www.ncbi.nlm.nih.gov/entrez/query.fcgi?db=gene&cmd=Retrieve&dopt=full_report&list_uids=9798) | IST1, ESCRT-III associated factor |
| [Details](https://mirdb.org/cgi-bin/target_detail.cgi?targetID=3252976) | 676 | 76 | hsa-miR-15a-5p | [PDCD1](http://www.ncbi.nlm.nih.gov/entrez/query.fcgi?db=gene&cmd=Retrieve&dopt=full_report&list_uids=5133) | programmed cell death 1 |
| [Details](https://mirdb.org/cgi-bin/target_detail.cgi?targetID=3252985) | 677 | 76 | hsa-miR-15a-5p | [YOD1](http://www.ncbi.nlm.nih.gov/entrez/query.fcgi?db=gene&cmd=Retrieve&dopt=full_report&list_uids=55432) | YOD1 deubiquitinase |
| [Details](https://mirdb.org/cgi-bin/target_detail.cgi?targetID=3252988) | 678 | 76 | hsa-miR-15a-5p | [DNAJC16](http://www.ncbi.nlm.nih.gov/entrez/query.fcgi?db=gene&cmd=Retrieve&dopt=full_report&list_uids=23341) | DnaJ heat shock protein family (Hsp40) member C16 |
| [Details](https://mirdb.org/cgi-bin/target_detail.cgi?targetID=3253099) | 679 | 76 | hsa-miR-15a-5p | [ZNF117](http://www.ncbi.nlm.nih.gov/entrez/query.fcgi?db=gene&cmd=Retrieve&dopt=full_report&list_uids=51351) | zinc finger protein 117 |
| [Details](https://mirdb.org/cgi-bin/target_detail.cgi?targetID=3253124) | 680 | 76 | hsa-miR-15a-5p | [PSAT1](http://www.ncbi.nlm.nih.gov/entrez/query.fcgi?db=gene&cmd=Retrieve&dopt=full_report&list_uids=29968) | phosphoserine aminotransferase 1 |
| [Details](https://mirdb.org/cgi-bin/target_detail.cgi?targetID=3253172) | 681 | 76 | hsa-miR-15a-5p | [PPFIA2](http://www.ncbi.nlm.nih.gov/entrez/query.fcgi?db=gene&cmd=Retrieve&dopt=full_report&list_uids=8499) | PTPRF interacting protein alpha 2 |
| [Details](https://mirdb.org/cgi-bin/target_detail.cgi?targetID=3253246) | 682 | 76 | hsa-miR-15a-5p | [KLHDC8B](http://www.ncbi.nlm.nih.gov/entrez/query.fcgi?db=gene&cmd=Retrieve&dopt=full_report&list_uids=200942) | kelch domain containing 8B |
| [Details](https://mirdb.org/cgi-bin/target_detail.cgi?targetID=3253279) | 683 | 76 | hsa-miR-15a-5p | [NLRX1](http://www.ncbi.nlm.nih.gov/entrez/query.fcgi?db=gene&cmd=Retrieve&dopt=full_report&list_uids=79671) | NLR family member X1 |
| [Details](https://mirdb.org/cgi-bin/target_detail.cgi?targetID=3253356) | 684 | 76 | hsa-miR-15a-5p | [KPNA1](http://www.ncbi.nlm.nih.gov/entrez/query.fcgi?db=gene&cmd=Retrieve&dopt=full_report&list_uids=3836) | karyopherin subunit alpha 1 |
| [Details](https://mirdb.org/cgi-bin/target_detail.cgi?targetID=3253458) | 685 | 76 | hsa-miR-15a-5p | [E2F7](http://www.ncbi.nlm.nih.gov/entrez/query.fcgi?db=gene&cmd=Retrieve&dopt=full_report&list_uids=144455) | E2F transcription factor 7 |
| [Details](https://mirdb.org/cgi-bin/target_detail.cgi?targetID=3253475) | 686 | 76 | hsa-miR-15a-5p | [GOLT1B](http://www.ncbi.nlm.nih.gov/entrez/query.fcgi?db=gene&cmd=Retrieve&dopt=full_report&list_uids=51026) | golgi transport 1B |
| [Details](https://mirdb.org/cgi-bin/target_detail.cgi?targetID=3253579) | 687 | 76 | hsa-miR-15a-5p | [NF2](http://www.ncbi.nlm.nih.gov/entrez/query.fcgi?db=gene&cmd=Retrieve&dopt=full_report&list_uids=4771) | neurofibromin 2 |
| [Details](https://mirdb.org/cgi-bin/target_detail.cgi?targetID=3253630) | 688 | 76 | hsa-miR-15a-5p | [CCNT2](http://www.ncbi.nlm.nih.gov/entrez/query.fcgi?db=gene&cmd=Retrieve&dopt=full_report&list_uids=905) | cyclin T2 |
| [Details](https://mirdb.org/cgi-bin/target_detail.cgi?targetID=3253764) | 689 | 76 | hsa-miR-15a-5p | [IL10RA](http://www.ncbi.nlm.nih.gov/entrez/query.fcgi?db=gene&cmd=Retrieve&dopt=full_report&list_uids=3587) | interleukin 10 receptor subunit alpha |
| [Details](https://mirdb.org/cgi-bin/target_detail.cgi?targetID=3253806) | 690 | 76 | hsa-miR-15a-5p | [MINDY2](http://www.ncbi.nlm.nih.gov/entrez/query.fcgi?db=gene&cmd=Retrieve&dopt=full_report&list_uids=54629) | MINDY lysine 48 deubiquitinase 2 |
| [Details](https://mirdb.org/cgi-bin/target_detail.cgi?targetID=3253844) | 691 | 76 | hsa-miR-15a-5p | [DLL4](http://www.ncbi.nlm.nih.gov/entrez/query.fcgi?db=gene&cmd=Retrieve&dopt=full_report&list_uids=54567) | delta like canonical Notch ligand 4 |
| [Details](https://mirdb.org/cgi-bin/target_detail.cgi?targetID=3253853) | 692 | 76 | hsa-miR-15a-5p | [HPSE2](http://www.ncbi.nlm.nih.gov/entrez/query.fcgi?db=gene&cmd=Retrieve&dopt=full_report&list_uids=60495) | heparanase 2 (inactive) |
| [Details](https://mirdb.org/cgi-bin/target_detail.cgi?targetID=3253930) | 693 | 76 | hsa-miR-15a-5p | [ASNSD1](http://www.ncbi.nlm.nih.gov/entrez/query.fcgi?db=gene&cmd=Retrieve&dopt=full_report&list_uids=54529) | asparagine synthetase domain containing 1 |
| [Details](https://mirdb.org/cgi-bin/target_detail.cgi?targetID=3253964) | 694 | 76 | hsa-miR-15a-5p | [RTN3](http://www.ncbi.nlm.nih.gov/entrez/query.fcgi?db=gene&cmd=Retrieve&dopt=full_report&list_uids=10313) | reticulon 3 |
| [Details](https://mirdb.org/cgi-bin/target_detail.cgi?targetID=3254032) | 695 | 76 | hsa-miR-15a-5p | [MXD3](http://www.ncbi.nlm.nih.gov/entrez/query.fcgi?db=gene&cmd=Retrieve&dopt=full_report&list_uids=83463) | MAX dimerization protein 3 |
| [Details](https://mirdb.org/cgi-bin/target_detail.cgi?targetID=3252713) | 696 | 75 | hsa-miR-15a-5p | [CLCN3](http://www.ncbi.nlm.nih.gov/entrez/query.fcgi?db=gene&cmd=Retrieve&dopt=full_report&list_uids=1182) | chloride voltage-gated channel 3 |
| [Details](https://mirdb.org/cgi-bin/target_detail.cgi?targetID=3252809) | 697 | 75 | hsa-miR-15a-5p | [LY9](http://www.ncbi.nlm.nih.gov/entrez/query.fcgi?db=gene&cmd=Retrieve&dopt=full_report&list_uids=4063) | lymphocyte antigen 9 |
| [Details](https://mirdb.org/cgi-bin/target_detail.cgi?targetID=3252869) | 698 | 75 | hsa-miR-15a-5p | [RNF43](http://www.ncbi.nlm.nih.gov/entrez/query.fcgi?db=gene&cmd=Retrieve&dopt=full_report&list_uids=54894) | ring finger protein 43 |
| [Details](https://mirdb.org/cgi-bin/target_detail.cgi?targetID=3252897) | 699 | 75 | hsa-miR-15a-5p | [YWHAQ](http://www.ncbi.nlm.nih.gov/entrez/query.fcgi?db=gene&cmd=Retrieve&dopt=full_report&list_uids=10971) | tyrosine 3-monooxygenase/tryptophan 5-monooxygenase activation protein theta |
| [Details](https://mirdb.org/cgi-bin/target_detail.cgi?targetID=3252900) | 700 | 75 | hsa-miR-15a-5p | [ABTB2](http://www.ncbi.nlm.nih.gov/entrez/query.fcgi?db=gene&cmd=Retrieve&dopt=full_report&list_uids=25841) | ankyrin repeat and BTB domain containing 2 |
| [Details](https://mirdb.org/cgi-bin/target_detail.cgi?targetID=3253008) | 701 | 75 | hsa-miR-15a-5p | [NAA15](http://www.ncbi.nlm.nih.gov/entrez/query.fcgi?db=gene&cmd=Retrieve&dopt=full_report&list_uids=80155) | N(alpha)-acetyltransferase 15, NatA auxiliary subunit |
| [Details](https://mirdb.org/cgi-bin/target_detail.cgi?targetID=3253071) | 702 | 75 | hsa-miR-15a-5p | [FGF18](http://www.ncbi.nlm.nih.gov/entrez/query.fcgi?db=gene&cmd=Retrieve&dopt=full_report&list_uids=8817) | fibroblast growth factor 18 |
| [Details](https://mirdb.org/cgi-bin/target_detail.cgi?targetID=3253074) | 703 | 75 | hsa-miR-15a-5p | [TLR1](http://www.ncbi.nlm.nih.gov/entrez/query.fcgi?db=gene&cmd=Retrieve&dopt=full_report&list_uids=7096) | toll like receptor 1 |
| [Details](https://mirdb.org/cgi-bin/target_detail.cgi?targetID=3253176) | 704 | 75 | hsa-miR-15a-5p | [SPRTN](http://www.ncbi.nlm.nih.gov/entrez/query.fcgi?db=gene&cmd=Retrieve&dopt=full_report&list_uids=83932) | SprT-like N-terminal domain |
| [Details](https://mirdb.org/cgi-bin/target_detail.cgi?targetID=3253179) | 705 | 75 | hsa-miR-15a-5p | [LATS2](http://www.ncbi.nlm.nih.gov/entrez/query.fcgi?db=gene&cmd=Retrieve&dopt=full_report&list_uids=26524) | large tumor suppressor kinase 2 |
| [Details](https://mirdb.org/cgi-bin/target_detail.cgi?targetID=3253266) | 706 | 75 | hsa-miR-15a-5p | [TSPYL2](http://www.ncbi.nlm.nih.gov/entrez/query.fcgi?db=gene&cmd=Retrieve&dopt=full_report&list_uids=64061) | TSPY like 2 |
| [Details](https://mirdb.org/cgi-bin/target_detail.cgi?targetID=3253474) | 707 | 75 | hsa-miR-15a-5p | [NUCKS1](http://www.ncbi.nlm.nih.gov/entrez/query.fcgi?db=gene&cmd=Retrieve&dopt=full_report&list_uids=64710) | nuclear casein kinase and cyclin dependent kinase substrate 1 |
| [Details](https://mirdb.org/cgi-bin/target_detail.cgi?targetID=3253501) | 708 | 75 | hsa-miR-15a-5p | [CRIM1](http://www.ncbi.nlm.nih.gov/entrez/query.fcgi?db=gene&cmd=Retrieve&dopt=full_report&list_uids=51232) | cysteine rich transmembrane BMP regulator 1 |
| [Details](https://mirdb.org/cgi-bin/target_detail.cgi?targetID=3253512) | 709 | 75 | hsa-miR-15a-5p | [ELAC2](http://www.ncbi.nlm.nih.gov/entrez/query.fcgi?db=gene&cmd=Retrieve&dopt=full_report&list_uids=60528) | elaC ribonuclease Z 2 |
| [Details](https://mirdb.org/cgi-bin/target_detail.cgi?targetID=3253549) | 710 | 75 | hsa-miR-15a-5p | [PRTG](http://www.ncbi.nlm.nih.gov/entrez/query.fcgi?db=gene&cmd=Retrieve&dopt=full_report&list_uids=283659) | protogenin |
| [Details](https://mirdb.org/cgi-bin/target_detail.cgi?targetID=3253663) | 711 | 75 | hsa-miR-15a-5p | [SCUBE3](http://www.ncbi.nlm.nih.gov/entrez/query.fcgi?db=gene&cmd=Retrieve&dopt=full_report&list_uids=222663) | signal peptide, CUB domain and EGF like domain containing 3 |
| [Details](https://mirdb.org/cgi-bin/target_detail.cgi?targetID=3253745) | 712 | 75 | hsa-miR-15a-5p | [DCAF8](http://www.ncbi.nlm.nih.gov/entrez/query.fcgi?db=gene&cmd=Retrieve&dopt=full_report&list_uids=50717) | DDB1 and CUL4 associated factor 8 |
| [Details](https://mirdb.org/cgi-bin/target_detail.cgi?targetID=3253869) | 713 | 75 | hsa-miR-15a-5p | [ALDH1A3](http://www.ncbi.nlm.nih.gov/entrez/query.fcgi?db=gene&cmd=Retrieve&dopt=full_report&list_uids=220) | aldehyde dehydrogenase 1 family member A3 |
| [Details](https://mirdb.org/cgi-bin/target_detail.cgi?targetID=3253889) | 714 | 75 | hsa-miR-15a-5p | [WDTC1](http://www.ncbi.nlm.nih.gov/entrez/query.fcgi?db=gene&cmd=Retrieve&dopt=full_report&list_uids=23038) | WD and tetratricopeptide repeats 1 |
| [Details](https://mirdb.org/cgi-bin/target_detail.cgi?targetID=3253900) | 715 | 75 | hsa-miR-15a-5p | [DEF8](http://www.ncbi.nlm.nih.gov/entrez/query.fcgi?db=gene&cmd=Retrieve&dopt=full_report&list_uids=54849) | differentially expressed in FDCP 8 homolog |
| [Details](https://mirdb.org/cgi-bin/target_detail.cgi?targetID=3253905) | 716 | 75 | hsa-miR-15a-5p | [WWP1](http://www.ncbi.nlm.nih.gov/entrez/query.fcgi?db=gene&cmd=Retrieve&dopt=full_report&list_uids=11059) | WW domain containing E3 ubiquitin protein ligase 1 |
| [Details](https://mirdb.org/cgi-bin/target_detail.cgi?targetID=3253950) | 717 | 75 | hsa-miR-15a-5p | [TRIM35](http://www.ncbi.nlm.nih.gov/entrez/query.fcgi?db=gene&cmd=Retrieve&dopt=full_report&list_uids=23087) | tripartite motif containing 35 |
| [Details](https://mirdb.org/cgi-bin/target_detail.cgi?targetID=3253962) | 718 | 75 | hsa-miR-15a-5p | [ENTPD7](http://www.ncbi.nlm.nih.gov/entrez/query.fcgi?db=gene&cmd=Retrieve&dopt=full_report&list_uids=57089) | ectonucleoside triphosphate diphosphohydrolase 7 |
| [Details](https://mirdb.org/cgi-bin/target_detail.cgi?targetID=3252709) | 719 | 74 | hsa-miR-15a-5p | [ZDHHC23](http://www.ncbi.nlm.nih.gov/entrez/query.fcgi?db=gene&cmd=Retrieve&dopt=full_report&list_uids=254887) | zinc finger DHHC-type containing 23 |
| [Details](https://mirdb.org/cgi-bin/target_detail.cgi?targetID=3252760) | 720 | 74 | hsa-miR-15a-5p | [MTMR4](http://www.ncbi.nlm.nih.gov/entrez/query.fcgi?db=gene&cmd=Retrieve&dopt=full_report&list_uids=9110) | myotubularin related protein 4 |
| [Details](https://mirdb.org/cgi-bin/target_detail.cgi?targetID=3252791) | 721 | 74 | hsa-miR-15a-5p | [ATP1B4](http://www.ncbi.nlm.nih.gov/entrez/query.fcgi?db=gene&cmd=Retrieve&dopt=full_report&list_uids=23439) | ATPase Na+/K+ transporting family member beta 4 |
| [Details](https://mirdb.org/cgi-bin/target_detail.cgi?targetID=3252973) | 722 | 74 | hsa-miR-15a-5p | [LRRFIP2](http://www.ncbi.nlm.nih.gov/entrez/query.fcgi?db=gene&cmd=Retrieve&dopt=full_report&list_uids=9209) | LRR binding FLII interacting protein 2 |
| [Details](https://mirdb.org/cgi-bin/target_detail.cgi?targetID=3253044) | 723 | 74 | hsa-miR-15a-5p | [ACSS2](http://www.ncbi.nlm.nih.gov/entrez/query.fcgi?db=gene&cmd=Retrieve&dopt=full_report&list_uids=55902) | acyl-CoA synthetase short chain family member 2 |
| [Details](https://mirdb.org/cgi-bin/target_detail.cgi?targetID=3253191) | 724 | 74 | hsa-miR-15a-5p | [XDH](http://www.ncbi.nlm.nih.gov/entrez/query.fcgi?db=gene&cmd=Retrieve&dopt=full_report&list_uids=7498) | xanthine dehydrogenase |
| [Details](https://mirdb.org/cgi-bin/target_detail.cgi?targetID=3253222) | 725 | 74 | hsa-miR-15a-5p | [DOLPP1](http://www.ncbi.nlm.nih.gov/entrez/query.fcgi?db=gene&cmd=Retrieve&dopt=full_report&list_uids=57171) | dolichyldiphosphatase 1 |
| [Details](https://mirdb.org/cgi-bin/target_detail.cgi?targetID=3253320) | 726 | 74 | hsa-miR-15a-5p | [DEPDC5](http://www.ncbi.nlm.nih.gov/entrez/query.fcgi?db=gene&cmd=Retrieve&dopt=full_report&list_uids=9681) | DEP domain containing 5 |
| [Details](https://mirdb.org/cgi-bin/target_detail.cgi?targetID=3253338) | 727 | 74 | hsa-miR-15a-5p | [FAM160B1](http://www.ncbi.nlm.nih.gov/entrez/query.fcgi?db=gene&cmd=Retrieve&dopt=full_report&list_uids=57700) | family with sequence similarity 160 member B1 |
| [Details](https://mirdb.org/cgi-bin/target_detail.cgi?targetID=3253375) | 728 | 74 | hsa-miR-15a-5p | [TACC1](http://www.ncbi.nlm.nih.gov/entrez/query.fcgi?db=gene&cmd=Retrieve&dopt=full_report&list_uids=6867) | transforming acidic coiled-coil containing protein 1 |
| [Details](https://mirdb.org/cgi-bin/target_detail.cgi?targetID=3253415) | 729 | 74 | hsa-miR-15a-5p | [TTC1](http://www.ncbi.nlm.nih.gov/entrez/query.fcgi?db=gene&cmd=Retrieve&dopt=full_report&list_uids=7265) | tetratricopeptide repeat domain 1 |
| [Details](https://mirdb.org/cgi-bin/target_detail.cgi?targetID=3253421) | 730 | 74 | hsa-miR-15a-5p | [ZBTB33](http://www.ncbi.nlm.nih.gov/entrez/query.fcgi?db=gene&cmd=Retrieve&dopt=full_report&list_uids=10009) | zinc finger and BTB domain containing 33 |
| [Details](https://mirdb.org/cgi-bin/target_detail.cgi?targetID=3253422) | 731 | 74 | hsa-miR-15a-5p | [PNISR](http://www.ncbi.nlm.nih.gov/entrez/query.fcgi?db=gene&cmd=Retrieve&dopt=full_report&list_uids=25957) | PNN interacting serine and arginine rich protein |
| [Details](https://mirdb.org/cgi-bin/target_detail.cgi?targetID=3253452) | 732 | 74 | hsa-miR-15a-5p | [ZNF704](http://www.ncbi.nlm.nih.gov/entrez/query.fcgi?db=gene&cmd=Retrieve&dopt=full_report&list_uids=619279) | zinc finger protein 704 |
| [Details](https://mirdb.org/cgi-bin/target_detail.cgi?targetID=3253459) | 733 | 74 | hsa-miR-15a-5p | [NEBL](http://www.ncbi.nlm.nih.gov/entrez/query.fcgi?db=gene&cmd=Retrieve&dopt=full_report&list_uids=10529) | nebulette |
| [Details](https://mirdb.org/cgi-bin/target_detail.cgi?targetID=3253508) | 734 | 74 | hsa-miR-15a-5p | [ETFRF1](http://www.ncbi.nlm.nih.gov/entrez/query.fcgi?db=gene&cmd=Retrieve&dopt=full_report&list_uids=144363) | electron transfer flavoprotein regulatory factor 1 |
| [Details](https://mirdb.org/cgi-bin/target_detail.cgi?targetID=3253517) | 735 | 74 | hsa-miR-15a-5p | [PWWP2B](http://www.ncbi.nlm.nih.gov/entrez/query.fcgi?db=gene&cmd=Retrieve&dopt=full_report&list_uids=170394) | PWWP domain containing 2B |
| [Details](https://mirdb.org/cgi-bin/target_detail.cgi?targetID=3253584) | 736 | 74 | hsa-miR-15a-5p | [SOS2](http://www.ncbi.nlm.nih.gov/entrez/query.fcgi?db=gene&cmd=Retrieve&dopt=full_report&list_uids=6655) | SOS Ras/Rho guanine nucleotide exchange factor 2 |
| [Details](https://mirdb.org/cgi-bin/target_detail.cgi?targetID=3253711) | 737 | 74 | hsa-miR-15a-5p | [SRSF11](http://www.ncbi.nlm.nih.gov/entrez/query.fcgi?db=gene&cmd=Retrieve&dopt=full_report&list_uids=9295) | serine and arginine rich splicing factor 11 |
| [Details](https://mirdb.org/cgi-bin/target_detail.cgi?targetID=3253821) | 738 | 74 | hsa-miR-15a-5p | [TFPI2](http://www.ncbi.nlm.nih.gov/entrez/query.fcgi?db=gene&cmd=Retrieve&dopt=full_report&list_uids=7980) | tissue factor pathway inhibitor 2 |
| [Details](https://mirdb.org/cgi-bin/target_detail.cgi?targetID=3253838) | 739 | 74 | hsa-miR-15a-5p | [CREG1](http://www.ncbi.nlm.nih.gov/entrez/query.fcgi?db=gene&cmd=Retrieve&dopt=full_report&list_uids=8804) | cellular repressor of E1A stimulated genes 1 |
| [Details](https://mirdb.org/cgi-bin/target_detail.cgi?targetID=3253879) | 740 | 74 | hsa-miR-15a-5p | [EN2](http://www.ncbi.nlm.nih.gov/entrez/query.fcgi?db=gene&cmd=Retrieve&dopt=full_report&list_uids=2020) | engrailed homeobox 2 |
| [Details](https://mirdb.org/cgi-bin/target_detail.cgi?targetID=3253934) | 741 | 74 | hsa-miR-15a-5p | [ATG4B](http://www.ncbi.nlm.nih.gov/entrez/query.fcgi?db=gene&cmd=Retrieve&dopt=full_report&list_uids=23192) | autophagy related 4B cysteine peptidase |
| [Details](https://mirdb.org/cgi-bin/target_detail.cgi?targetID=3253974) | 742 | 74 | hsa-miR-15a-5p | [ADSS](http://www.ncbi.nlm.nih.gov/entrez/query.fcgi?db=gene&cmd=Retrieve&dopt=full_report&list_uids=159) | adenylosuccinate synthase |
| [Details](https://mirdb.org/cgi-bin/target_detail.cgi?targetID=3253993) | 743 | 74 | hsa-miR-15a-5p | [TCIM](http://www.ncbi.nlm.nih.gov/entrez/query.fcgi?db=gene&cmd=Retrieve&dopt=full_report&list_uids=56892) | transcriptional and immune response regulator |
| [Details](https://mirdb.org/cgi-bin/target_detail.cgi?targetID=3254001) | 744 | 74 | hsa-miR-15a-5p | [DNAJC24](http://www.ncbi.nlm.nih.gov/entrez/query.fcgi?db=gene&cmd=Retrieve&dopt=full_report&list_uids=120526) | DnaJ heat shock protein family (Hsp40) member C24 |
| [Details](https://mirdb.org/cgi-bin/target_detail.cgi?targetID=3254042) | 745 | 74 | hsa-miR-15a-5p | [MARCH4](http://www.ncbi.nlm.nih.gov/entrez/query.fcgi?db=gene&cmd=Retrieve&dopt=full_report&list_uids=57574) | membrane associated ring-CH-type finger 4 |
| [Details](https://mirdb.org/cgi-bin/target_detail.cgi?targetID=3252649) | 746 | 73 | hsa-miR-15a-5p | [ZNF705A](http://www.ncbi.nlm.nih.gov/entrez/query.fcgi?db=gene&cmd=Retrieve&dopt=full_report&list_uids=440077) | zinc finger protein 705A |
| [Details](https://mirdb.org/cgi-bin/target_detail.cgi?targetID=3252654) | 747 | 73 | hsa-miR-15a-5p | [PPM1D](http://www.ncbi.nlm.nih.gov/entrez/query.fcgi?db=gene&cmd=Retrieve&dopt=full_report&list_uids=8493) | protein phosphatase, Mg2+/Mn2+ dependent 1D |
| [Details](https://mirdb.org/cgi-bin/target_detail.cgi?targetID=3252737) | 748 | 73 | hsa-miR-15a-5p | [PPP2R5C](http://www.ncbi.nlm.nih.gov/entrez/query.fcgi?db=gene&cmd=Retrieve&dopt=full_report&list_uids=5527) | protein phosphatase 2 regulatory subunit B'gamma |
| [Details](https://mirdb.org/cgi-bin/target_detail.cgi?targetID=3252837) | 749 | 73 | hsa-miR-15a-5p | [IP6K1](http://www.ncbi.nlm.nih.gov/entrez/query.fcgi?db=gene&cmd=Retrieve&dopt=full_report&list_uids=9807) | inositol hexakisphosphate kinase 1 |
| [Details](https://mirdb.org/cgi-bin/target_detail.cgi?targetID=3252871) | 750 | 73 | hsa-miR-15a-5p | [PHKA1](http://www.ncbi.nlm.nih.gov/entrez/query.fcgi?db=gene&cmd=Retrieve&dopt=full_report&list_uids=5255) | phosphorylase kinase regulatory subunit alpha 1 |
| [Details](https://mirdb.org/cgi-bin/target_detail.cgi?targetID=3252935) | 751 | 73 | hsa-miR-15a-5p | [MMS19](http://www.ncbi.nlm.nih.gov/entrez/query.fcgi?db=gene&cmd=Retrieve&dopt=full_report&list_uids=64210) | MMS19 homolog, cytosolic iron-sulfur assembly component |
| [Details](https://mirdb.org/cgi-bin/target_detail.cgi?targetID=3253055) | 752 | 73 | hsa-miR-15a-5p | [CASZ1](http://www.ncbi.nlm.nih.gov/entrez/query.fcgi?db=gene&cmd=Retrieve&dopt=full_report&list_uids=54897) | castor zinc finger 1 |
| [Details](https://mirdb.org/cgi-bin/target_detail.cgi?targetID=3253115) | 753 | 73 | hsa-miR-15a-5p | [KL](http://www.ncbi.nlm.nih.gov/entrez/query.fcgi?db=gene&cmd=Retrieve&dopt=full_report&list_uids=9365) | klotho |
| [Details](https://mirdb.org/cgi-bin/target_detail.cgi?targetID=3253160) | 754 | 73 | hsa-miR-15a-5p | [EMC4](http://www.ncbi.nlm.nih.gov/entrez/query.fcgi?db=gene&cmd=Retrieve&dopt=full_report&list_uids=51234) | ER membrane protein complex subunit 4 |
| [Details](https://mirdb.org/cgi-bin/target_detail.cgi?targetID=3253205) | 755 | 73 | hsa-miR-15a-5p | [ZNF264](http://www.ncbi.nlm.nih.gov/entrez/query.fcgi?db=gene&cmd=Retrieve&dopt=full_report&list_uids=9422) | zinc finger protein 264 |
| [Details](https://mirdb.org/cgi-bin/target_detail.cgi?targetID=3253409) | 756 | 73 | hsa-miR-15a-5p | [ANKRD33B](http://www.ncbi.nlm.nih.gov/entrez/query.fcgi?db=gene&cmd=Retrieve&dopt=full_report&list_uids=651746) | ankyrin repeat domain 33B |
| [Details](https://mirdb.org/cgi-bin/target_detail.cgi?targetID=3253567) | 757 | 73 | hsa-miR-15a-5p | [RAB11FIP1](http://www.ncbi.nlm.nih.gov/entrez/query.fcgi?db=gene&cmd=Retrieve&dopt=full_report&list_uids=80223) | RAB11 family interacting protein 1 |
| [Details](https://mirdb.org/cgi-bin/target_detail.cgi?targetID=3253706) | 758 | 73 | hsa-miR-15a-5p | [HPCAL4](http://www.ncbi.nlm.nih.gov/entrez/query.fcgi?db=gene&cmd=Retrieve&dopt=full_report&list_uids=51440) | hippocalcin like 4 |
| [Details](https://mirdb.org/cgi-bin/target_detail.cgi?targetID=3253747) | 759 | 73 | hsa-miR-15a-5p | [ESPN](http://www.ncbi.nlm.nih.gov/entrez/query.fcgi?db=gene&cmd=Retrieve&dopt=full_report&list_uids=83715) | espin |
| [Details](https://mirdb.org/cgi-bin/target_detail.cgi?targetID=3253796) | 760 | 73 | hsa-miR-15a-5p | [SATB2](http://www.ncbi.nlm.nih.gov/entrez/query.fcgi?db=gene&cmd=Retrieve&dopt=full_report&list_uids=23314) | SATB homeobox 2 |
| [Details](https://mirdb.org/cgi-bin/target_detail.cgi?targetID=3253811) | 761 | 73 | hsa-miR-15a-5p | [TMEM221](http://www.ncbi.nlm.nih.gov/entrez/query.fcgi?db=gene&cmd=Retrieve&dopt=full_report&list_uids=100130519) | transmembrane protein 221 |
| [Details](https://mirdb.org/cgi-bin/target_detail.cgi?targetID=3253876) | 762 | 73 | hsa-miR-15a-5p | [NCBP3](http://www.ncbi.nlm.nih.gov/entrez/query.fcgi?db=gene&cmd=Retrieve&dopt=full_report&list_uids=55421) | nuclear cap binding subunit 3 |
| [Details](https://mirdb.org/cgi-bin/target_detail.cgi?targetID=3253881) | 763 | 73 | hsa-miR-15a-5p | [MAPK9](http://www.ncbi.nlm.nih.gov/entrez/query.fcgi?db=gene&cmd=Retrieve&dopt=full_report&list_uids=5601) | mitogen-activated protein kinase 9 |
| [Details](https://mirdb.org/cgi-bin/target_detail.cgi?targetID=3253990) | 764 | 73 | hsa-miR-15a-5p | [OSCAR](http://www.ncbi.nlm.nih.gov/entrez/query.fcgi?db=gene&cmd=Retrieve&dopt=full_report&list_uids=126014) | osteoclast associated, immunoglobulin-like receptor |
| [Details](https://mirdb.org/cgi-bin/target_detail.cgi?targetID=3254015) | 765 | 73 | hsa-miR-15a-5p | [INSYN2](http://www.ncbi.nlm.nih.gov/entrez/query.fcgi?db=gene&cmd=Retrieve&dopt=full_report&list_uids=642938) | inhibitory synaptic factor 2A |
| [Details](https://mirdb.org/cgi-bin/target_detail.cgi?targetID=3252638) | 766 | 72 | hsa-miR-15a-5p | [RBM20](http://www.ncbi.nlm.nih.gov/entrez/query.fcgi?db=gene&cmd=Retrieve&dopt=full_report&list_uids=282996) | RNA binding motif protein 20 |
| [Details](https://mirdb.org/cgi-bin/target_detail.cgi?targetID=3252692) | 767 | 72 | hsa-miR-15a-5p | [ZDHHC16](http://www.ncbi.nlm.nih.gov/entrez/query.fcgi?db=gene&cmd=Retrieve&dopt=full_report&list_uids=84287) | zinc finger DHHC-type containing 16 |
| [Details](https://mirdb.org/cgi-bin/target_detail.cgi?targetID=3252733) | 768 | 72 | hsa-miR-15a-5p | [RNF41](http://www.ncbi.nlm.nih.gov/entrez/query.fcgi?db=gene&cmd=Retrieve&dopt=full_report&list_uids=10193) | ring finger protein 41 |
| [Details](https://mirdb.org/cgi-bin/target_detail.cgi?targetID=3252740) | 769 | 72 | hsa-miR-15a-5p | [SLC35A4](http://www.ncbi.nlm.nih.gov/entrez/query.fcgi?db=gene&cmd=Retrieve&dopt=full_report&list_uids=113829) | solute carrier family 35 member A4 |
| [Details](https://mirdb.org/cgi-bin/target_detail.cgi?targetID=3252743) | 770 | 72 | hsa-miR-15a-5p | [DENND6A](http://www.ncbi.nlm.nih.gov/entrez/query.fcgi?db=gene&cmd=Retrieve&dopt=full_report&list_uids=201627) | DENN domain containing 6A |
| [Details](https://mirdb.org/cgi-bin/target_detail.cgi?targetID=3252763) | 771 | 72 | hsa-miR-15a-5p | [PANK1](http://www.ncbi.nlm.nih.gov/entrez/query.fcgi?db=gene&cmd=Retrieve&dopt=full_report&list_uids=53354) | pantothenate kinase 1 |
| [Details](https://mirdb.org/cgi-bin/target_detail.cgi?targetID=3252929) | 772 | 72 | hsa-miR-15a-5p | [AFF4](http://www.ncbi.nlm.nih.gov/entrez/query.fcgi?db=gene&cmd=Retrieve&dopt=full_report&list_uids=27125) | AF4/FMR2 family member 4 |
| [Details](https://mirdb.org/cgi-bin/target_detail.cgi?targetID=3253003) | 773 | 72 | hsa-miR-15a-5p | [ESRRG](http://www.ncbi.nlm.nih.gov/entrez/query.fcgi?db=gene&cmd=Retrieve&dopt=full_report&list_uids=2104) | estrogen related receptor gamma |
| [Details](https://mirdb.org/cgi-bin/target_detail.cgi?targetID=3253127) | 774 | 72 | hsa-miR-15a-5p | [ADAMTS5](http://www.ncbi.nlm.nih.gov/entrez/query.fcgi?db=gene&cmd=Retrieve&dopt=full_report&list_uids=11096) | ADAM metallopeptidase with thrombospondin type 1 motif 5 |
| [Details](https://mirdb.org/cgi-bin/target_detail.cgi?targetID=3253151) | 775 | 72 | hsa-miR-15a-5p | [ABCB5](http://www.ncbi.nlm.nih.gov/entrez/query.fcgi?db=gene&cmd=Retrieve&dopt=full_report&list_uids=340273) | ATP binding cassette subfamily B member 5 |
| [Details](https://mirdb.org/cgi-bin/target_detail.cgi?targetID=3253224) | 776 | 72 | hsa-miR-15a-5p | [PIEZO1](http://www.ncbi.nlm.nih.gov/entrez/query.fcgi?db=gene&cmd=Retrieve&dopt=full_report&list_uids=9780) | piezo type mechanosensitive ion channel component 1 |
| [Details](https://mirdb.org/cgi-bin/target_detail.cgi?targetID=3253242) | 777 | 72 | hsa-miR-15a-5p | [ARHGAP5](http://www.ncbi.nlm.nih.gov/entrez/query.fcgi?db=gene&cmd=Retrieve&dopt=full_report&list_uids=394) | Rho GTPase activating protein 5 |
| [Details](https://mirdb.org/cgi-bin/target_detail.cgi?targetID=3253284) | 778 | 72 | hsa-miR-15a-5p | [TMEM43](http://www.ncbi.nlm.nih.gov/entrez/query.fcgi?db=gene&cmd=Retrieve&dopt=full_report&list_uids=79188) | transmembrane protein 43 |
| [Details](https://mirdb.org/cgi-bin/target_detail.cgi?targetID=3253310) | 779 | 72 | hsa-miR-15a-5p | [SLC25A22](http://www.ncbi.nlm.nih.gov/entrez/query.fcgi?db=gene&cmd=Retrieve&dopt=full_report&list_uids=79751) | solute carrier family 25 member 22 |
| [Details](https://mirdb.org/cgi-bin/target_detail.cgi?targetID=3253448) | 780 | 72 | hsa-miR-15a-5p | [TMEM255A](http://www.ncbi.nlm.nih.gov/entrez/query.fcgi?db=gene&cmd=Retrieve&dopt=full_report&list_uids=55026) | transmembrane protein 255A |
| [Details](https://mirdb.org/cgi-bin/target_detail.cgi?targetID=3253594) | 781 | 72 | hsa-miR-15a-5p | [ZNF705D](http://www.ncbi.nlm.nih.gov/entrez/query.fcgi?db=gene&cmd=Retrieve&dopt=full_report&list_uids=728957) | zinc finger protein 705D |
| [Details](https://mirdb.org/cgi-bin/target_detail.cgi?targetID=3253692) | 782 | 72 | hsa-miR-15a-5p | [CUL2](http://www.ncbi.nlm.nih.gov/entrez/query.fcgi?db=gene&cmd=Retrieve&dopt=full_report&list_uids=8453) | cullin 2 |
| [Details](https://mirdb.org/cgi-bin/target_detail.cgi?targetID=3253750) | 783 | 72 | hsa-miR-15a-5p | [RNF125](http://www.ncbi.nlm.nih.gov/entrez/query.fcgi?db=gene&cmd=Retrieve&dopt=full_report&list_uids=54941) | ring finger protein 125 |
| [Details](https://mirdb.org/cgi-bin/target_detail.cgi?targetID=3253754) | 784 | 72 | hsa-miR-15a-5p | [FZD6](http://www.ncbi.nlm.nih.gov/entrez/query.fcgi?db=gene&cmd=Retrieve&dopt=full_report&list_uids=8323) | frizzled class receptor 6 |
| [Details](https://mirdb.org/cgi-bin/target_detail.cgi?targetID=3253786) | 785 | 72 | hsa-miR-15a-5p | [MTHFR](http://www.ncbi.nlm.nih.gov/entrez/query.fcgi?db=gene&cmd=Retrieve&dopt=full_report&list_uids=4524) | methylenetetrahydrofolate reductase |
| [Details](https://mirdb.org/cgi-bin/target_detail.cgi?targetID=3253830) | 786 | 72 | hsa-miR-15a-5p | [SLC30A8](http://www.ncbi.nlm.nih.gov/entrez/query.fcgi?db=gene&cmd=Retrieve&dopt=full_report&list_uids=169026) | solute carrier family 30 member 8 |
| [Details](https://mirdb.org/cgi-bin/target_detail.cgi?targetID=3253859) | 787 | 72 | hsa-miR-15a-5p | [NPAS3](http://www.ncbi.nlm.nih.gov/entrez/query.fcgi?db=gene&cmd=Retrieve&dopt=full_report&list_uids=64067) | neuronal PAS domain protein 3 |
| [Details](https://mirdb.org/cgi-bin/target_detail.cgi?targetID=3253920) | 788 | 72 | hsa-miR-15a-5p | [ABCC5](http://www.ncbi.nlm.nih.gov/entrez/query.fcgi?db=gene&cmd=Retrieve&dopt=full_report&list_uids=10057) | ATP binding cassette subfamily C member 5 |
| [Details](https://mirdb.org/cgi-bin/target_detail.cgi?targetID=3253992) | 789 | 72 | hsa-miR-15a-5p | [MAP3K7](http://www.ncbi.nlm.nih.gov/entrez/query.fcgi?db=gene&cmd=Retrieve&dopt=full_report&list_uids=6885) | mitogen-activated protein kinase kinase kinase 7 |
| [Details](https://mirdb.org/cgi-bin/target_detail.cgi?targetID=3253994) | 790 | 72 | hsa-miR-15a-5p | [PFKFB4](http://www.ncbi.nlm.nih.gov/entrez/query.fcgi?db=gene&cmd=Retrieve&dopt=full_report&list_uids=5210) | 6-phosphofructo-2-kinase/fructose-2,6-biphosphatase 4 |
| [Details](https://mirdb.org/cgi-bin/target_detail.cgi?targetID=3254009) | 791 | 72 | hsa-miR-15a-5p | [GLYATL3](http://www.ncbi.nlm.nih.gov/entrez/query.fcgi?db=gene&cmd=Retrieve&dopt=full_report&list_uids=389396) | glycine-N-acyltransferase like 3 |
| [Details](https://mirdb.org/cgi-bin/target_detail.cgi?targetID=3252700) | 792 | 71 | hsa-miR-15a-5p | [PDK3](http://www.ncbi.nlm.nih.gov/entrez/query.fcgi?db=gene&cmd=Retrieve&dopt=full_report&list_uids=5165) | pyruvate dehydrogenase kinase 3 |
| [Details](https://mirdb.org/cgi-bin/target_detail.cgi?targetID=3252742) | 793 | 71 | hsa-miR-15a-5p | [PRKG1](http://www.ncbi.nlm.nih.gov/entrez/query.fcgi?db=gene&cmd=Retrieve&dopt=full_report&list_uids=5592) | protein kinase cGMP-dependent 1 |
| [Details](https://mirdb.org/cgi-bin/target_detail.cgi?targetID=3252756) | 794 | 71 | hsa-miR-15a-5p | [MFAP5](http://www.ncbi.nlm.nih.gov/entrez/query.fcgi?db=gene&cmd=Retrieve&dopt=full_report&list_uids=8076) | microfibril associated protein 5 |
| [Details](https://mirdb.org/cgi-bin/target_detail.cgi?targetID=3252764) | 795 | 71 | hsa-miR-15a-5p | [EXTL3](http://www.ncbi.nlm.nih.gov/entrez/query.fcgi?db=gene&cmd=Retrieve&dopt=full_report&list_uids=2137) | exostosin like glycosyltransferase 3 |
| [Details](https://mirdb.org/cgi-bin/target_detail.cgi?targetID=3252852) | 796 | 71 | hsa-miR-15a-5p | [PAM](http://www.ncbi.nlm.nih.gov/entrez/query.fcgi?db=gene&cmd=Retrieve&dopt=full_report&list_uids=5066) | peptidylglycine alpha-amidating monooxygenase |
| [Details](https://mirdb.org/cgi-bin/target_detail.cgi?targetID=3252853) | 797 | 71 | hsa-miR-15a-5p | [NOL4L](http://www.ncbi.nlm.nih.gov/entrez/query.fcgi?db=gene&cmd=Retrieve&dopt=full_report&list_uids=140688) | nucleolar protein 4 like |
| [Details](https://mirdb.org/cgi-bin/target_detail.cgi?targetID=3252954) | 798 | 71 | hsa-miR-15a-5p | [FURIN](http://www.ncbi.nlm.nih.gov/entrez/query.fcgi?db=gene&cmd=Retrieve&dopt=full_report&list_uids=5045) | furin, paired basic amino acid cleaving enzyme |
| [Details](https://mirdb.org/cgi-bin/target_detail.cgi?targetID=3252998) | 799 | 71 | hsa-miR-15a-5p | [TSPAN5](http://www.ncbi.nlm.nih.gov/entrez/query.fcgi?db=gene&cmd=Retrieve&dopt=full_report&list_uids=10098) | tetraspanin 5 |
| [Details](https://mirdb.org/cgi-bin/target_detail.cgi?targetID=3253301) | 800 | 71 | hsa-miR-15a-5p | [CAAP1](http://www.ncbi.nlm.nih.gov/entrez/query.fcgi?db=gene&cmd=Retrieve&dopt=full_report&list_uids=79886) | caspase activity and apoptosis inhibitor 1 |
| [Details](https://mirdb.org/cgi-bin/target_detail.cgi?targetID=3253313) | 801 | 71 | hsa-miR-15a-5p | [TVP23C](http://www.ncbi.nlm.nih.gov/entrez/query.fcgi?db=gene&cmd=Retrieve&dopt=full_report&list_uids=201158) | trans-golgi network vesicle protein 23 homolog C |
| [Details](https://mirdb.org/cgi-bin/target_detail.cgi?targetID=3253391) | 802 | 71 | hsa-miR-15a-5p | [RNF183](http://www.ncbi.nlm.nih.gov/entrez/query.fcgi?db=gene&cmd=Retrieve&dopt=full_report&list_uids=138065) | ring finger protein 183 |
| [Details](https://mirdb.org/cgi-bin/target_detail.cgi?targetID=3253418) | 803 | 71 | hsa-miR-15a-5p | [ZNF423](http://www.ncbi.nlm.nih.gov/entrez/query.fcgi?db=gene&cmd=Retrieve&dopt=full_report&list_uids=23090) | zinc finger protein 423 |
| [Details](https://mirdb.org/cgi-bin/target_detail.cgi?targetID=3253481) | 804 | 71 | hsa-miR-15a-5p | [FAT3](http://www.ncbi.nlm.nih.gov/entrez/query.fcgi?db=gene&cmd=Retrieve&dopt=full_report&list_uids=120114) | FAT atypical cadherin 3 |
| [Details](https://mirdb.org/cgi-bin/target_detail.cgi?targetID=3253506) | 805 | 71 | hsa-miR-15a-5p | [LYNX1](http://www.ncbi.nlm.nih.gov/entrez/query.fcgi?db=gene&cmd=Retrieve&dopt=full_report&list_uids=66004) | Ly6/neurotoxin 1 |
| [Details](https://mirdb.org/cgi-bin/target_detail.cgi?targetID=3253564) | 806 | 71 | hsa-miR-15a-5p | [COL4A3BP](http://www.ncbi.nlm.nih.gov/entrez/query.fcgi?db=gene&cmd=Retrieve&dopt=full_report&list_uids=10087) | collagen type IV alpha 3 binding protein |
| [Details](https://mirdb.org/cgi-bin/target_detail.cgi?targetID=3253573) | 807 | 71 | hsa-miR-15a-5p | [WSB1](http://www.ncbi.nlm.nih.gov/entrez/query.fcgi?db=gene&cmd=Retrieve&dopt=full_report&list_uids=26118) | WD repeat and SOCS box containing 1 |
| [Details](https://mirdb.org/cgi-bin/target_detail.cgi?targetID=3253578) | 808 | 71 | hsa-miR-15a-5p | [NTRK2](http://www.ncbi.nlm.nih.gov/entrez/query.fcgi?db=gene&cmd=Retrieve&dopt=full_report&list_uids=4915) | neurotrophic receptor tyrosine kinase 2 |
| [Details](https://mirdb.org/cgi-bin/target_detail.cgi?targetID=3253631) | 809 | 71 | hsa-miR-15a-5p | [MAP3K21](http://www.ncbi.nlm.nih.gov/entrez/query.fcgi?db=gene&cmd=Retrieve&dopt=full_report&list_uids=84451) | mitogen-activated protein kinase kinase kinase 21 |
| [Details](https://mirdb.org/cgi-bin/target_detail.cgi?targetID=3253676) | 810 | 71 | hsa-miR-15a-5p | [PARD6B](http://www.ncbi.nlm.nih.gov/entrez/query.fcgi?db=gene&cmd=Retrieve&dopt=full_report&list_uids=84612) | par-6 family cell polarity regulator beta |
| [Details](https://mirdb.org/cgi-bin/target_detail.cgi?targetID=3253712) | 811 | 71 | hsa-miR-15a-5p | [IRS1](http://www.ncbi.nlm.nih.gov/entrez/query.fcgi?db=gene&cmd=Retrieve&dopt=full_report&list_uids=3667) | insulin receptor substrate 1 |
| [Details](https://mirdb.org/cgi-bin/target_detail.cgi?targetID=3253894) | 812 | 71 | hsa-miR-15a-5p | [SEC61A1](http://www.ncbi.nlm.nih.gov/entrez/query.fcgi?db=gene&cmd=Retrieve&dopt=full_report&list_uids=29927) | Sec61 translocon alpha 1 subunit |
| [Details](https://mirdb.org/cgi-bin/target_detail.cgi?targetID=3254043) | 813 | 71 | hsa-miR-15a-5p | [PAQR3](http://www.ncbi.nlm.nih.gov/entrez/query.fcgi?db=gene&cmd=Retrieve&dopt=full_report&list_uids=152559) | progestin and adipoQ receptor family member 3 |
| [Details](https://mirdb.org/cgi-bin/target_detail.cgi?targetID=3252669) | 814 | 70 | hsa-miR-15a-5p | [DCAF7](http://www.ncbi.nlm.nih.gov/entrez/query.fcgi?db=gene&cmd=Retrieve&dopt=full_report&list_uids=10238) | DDB1 and CUL4 associated factor 7 |
| [Details](https://mirdb.org/cgi-bin/target_detail.cgi?targetID=3252693) | 815 | 70 | hsa-miR-15a-5p | [PARP12](http://www.ncbi.nlm.nih.gov/entrez/query.fcgi?db=gene&cmd=Retrieve&dopt=full_report&list_uids=64761) | poly(ADP-ribose) polymerase family member 12 |
| [Details](https://mirdb.org/cgi-bin/target_detail.cgi?targetID=3252738) | 816 | 70 | hsa-miR-15a-5p | [SPRY4](http://www.ncbi.nlm.nih.gov/entrez/query.fcgi?db=gene&cmd=Retrieve&dopt=full_report&list_uids=81848) | sprouty RTK signaling antagonist 4 |
| [Details](https://mirdb.org/cgi-bin/target_detail.cgi?targetID=3252755) | 817 | 70 | hsa-miR-15a-5p | [CDV3](http://www.ncbi.nlm.nih.gov/entrez/query.fcgi?db=gene&cmd=Retrieve&dopt=full_report&list_uids=55573) | CDV3 homolog |
| [Details](https://mirdb.org/cgi-bin/target_detail.cgi?targetID=3252765) | 818 | 70 | hsa-miR-15a-5p | [CHORDC1](http://www.ncbi.nlm.nih.gov/entrez/query.fcgi?db=gene&cmd=Retrieve&dopt=full_report&list_uids=26973) | cysteine and histidine rich domain containing 1 |
| [Details](https://mirdb.org/cgi-bin/target_detail.cgi?targetID=3252951) | 819 | 70 | hsa-miR-15a-5p | [PACRG](http://www.ncbi.nlm.nih.gov/entrez/query.fcgi?db=gene&cmd=Retrieve&dopt=full_report&list_uids=135138) | parkin coregulated |
| [Details](https://mirdb.org/cgi-bin/target_detail.cgi?targetID=3252978) | 820 | 70 | hsa-miR-15a-5p | [TXNDC17](http://www.ncbi.nlm.nih.gov/entrez/query.fcgi?db=gene&cmd=Retrieve&dopt=full_report&list_uids=84817) | thioredoxin domain containing 17 |
| [Details](https://mirdb.org/cgi-bin/target_detail.cgi?targetID=3253091) | 821 | 70 | hsa-miR-15a-5p | [SLC24A3](http://www.ncbi.nlm.nih.gov/entrez/query.fcgi?db=gene&cmd=Retrieve&dopt=full_report&list_uids=57419) | solute carrier family 24 member 3 |
| [Details](https://mirdb.org/cgi-bin/target_detail.cgi?targetID=3253121) | 822 | 70 | hsa-miR-15a-5p | [DRAM1](http://www.ncbi.nlm.nih.gov/entrez/query.fcgi?db=gene&cmd=Retrieve&dopt=full_report&list_uids=55332) | DNA damage regulated autophagy modulator 1 |
| [Details](https://mirdb.org/cgi-bin/target_detail.cgi?targetID=3253226) | 823 | 70 | hsa-miR-15a-5p | [FNDC3B](http://www.ncbi.nlm.nih.gov/entrez/query.fcgi?db=gene&cmd=Retrieve&dopt=full_report&list_uids=64778) | fibronectin type III domain containing 3B |
| [Details](https://mirdb.org/cgi-bin/target_detail.cgi?targetID=3253268) | 824 | 70 | hsa-miR-15a-5p | [CARNMT1](http://www.ncbi.nlm.nih.gov/entrez/query.fcgi?db=gene&cmd=Retrieve&dopt=full_report&list_uids=138199) | carnosine N-methyltransferase 1 |
| [Details](https://mirdb.org/cgi-bin/target_detail.cgi?targetID=3253277) | 825 | 70 | hsa-miR-15a-5p | [LIPE](http://www.ncbi.nlm.nih.gov/entrez/query.fcgi?db=gene&cmd=Retrieve&dopt=full_report&list_uids=3991) | lipase E, hormone sensitive type |
| [Details](https://mirdb.org/cgi-bin/target_detail.cgi?targetID=3253336) | 826 | 70 | hsa-miR-15a-5p | [RUBCNL](http://www.ncbi.nlm.nih.gov/entrez/query.fcgi?db=gene&cmd=Retrieve&dopt=full_report&list_uids=80183) | rubicon like autophagy enhancer |
| [Details](https://mirdb.org/cgi-bin/target_detail.cgi?targetID=3253537) | 827 | 70 | hsa-miR-15a-5p | [MAPK8](http://www.ncbi.nlm.nih.gov/entrez/query.fcgi?db=gene&cmd=Retrieve&dopt=full_report&list_uids=5599) | mitogen-activated protein kinase 8 |
| [Details](https://mirdb.org/cgi-bin/target_detail.cgi?targetID=3253572) | 828 | 70 | hsa-miR-15a-5p | [SINHCAF](http://www.ncbi.nlm.nih.gov/entrez/query.fcgi?db=gene&cmd=Retrieve&dopt=full_report&list_uids=58516) | SIN3-HDAC complex associated factor |
| [Details](https://mirdb.org/cgi-bin/target_detail.cgi?targetID=3253600) | 829 | 70 | hsa-miR-15a-5p | [C5orf63](http://www.ncbi.nlm.nih.gov/entrez/query.fcgi?db=gene&cmd=Retrieve&dopt=full_report&list_uids=401207) | chromosome 5 open reading frame 63 |
| [Details](https://mirdb.org/cgi-bin/target_detail.cgi?targetID=3253622) | 830 | 70 | hsa-miR-15a-5p | [ELK4](http://www.ncbi.nlm.nih.gov/entrez/query.fcgi?db=gene&cmd=Retrieve&dopt=full_report&list_uids=2005) | ELK4, ETS transcription factor |
| [Details](https://mirdb.org/cgi-bin/target_detail.cgi?targetID=3253661) | 831 | 70 | hsa-miR-15a-5p | [PANK2](http://www.ncbi.nlm.nih.gov/entrez/query.fcgi?db=gene&cmd=Retrieve&dopt=full_report&list_uids=80025) | pantothenate kinase 2 |
| [Details](https://mirdb.org/cgi-bin/target_detail.cgi?targetID=3253690) | 832 | 70 | hsa-miR-15a-5p | [VSX1](http://www.ncbi.nlm.nih.gov/entrez/query.fcgi?db=gene&cmd=Retrieve&dopt=full_report&list_uids=30813) | visual system homeobox 1 |
| [Details](https://mirdb.org/cgi-bin/target_detail.cgi?targetID=3253705) | 833 | 70 | hsa-miR-15a-5p | [FAM189B](http://www.ncbi.nlm.nih.gov/entrez/query.fcgi?db=gene&cmd=Retrieve&dopt=full_report&list_uids=10712) | family with sequence similarity 189 member B |
| [Details](https://mirdb.org/cgi-bin/target_detail.cgi?targetID=3253738) | 834 | 70 | hsa-miR-15a-5p | [ANK2](http://www.ncbi.nlm.nih.gov/entrez/query.fcgi?db=gene&cmd=Retrieve&dopt=full_report&list_uids=287) | ankyrin 2 |
| [Details](https://mirdb.org/cgi-bin/target_detail.cgi?targetID=3253802) | 835 | 70 | hsa-miR-15a-5p | [PLSCR4](http://www.ncbi.nlm.nih.gov/entrez/query.fcgi?db=gene&cmd=Retrieve&dopt=full_report&list_uids=57088) | phospholipid scramblase 4 |
| [Details](https://mirdb.org/cgi-bin/target_detail.cgi?targetID=3253848) | 836 | 70 | hsa-miR-15a-5p | [TPD52L3](http://www.ncbi.nlm.nih.gov/entrez/query.fcgi?db=gene&cmd=Retrieve&dopt=full_report&list_uids=89882) | TPD52 like 3 |
| [Details](https://mirdb.org/cgi-bin/target_detail.cgi?targetID=3253863) | 837 | 70 | hsa-miR-15a-5p | [HAS2](http://www.ncbi.nlm.nih.gov/entrez/query.fcgi?db=gene&cmd=Retrieve&dopt=full_report&list_uids=3037) | hyaluronan synthase 2 |
| [Details](https://mirdb.org/cgi-bin/target_detail.cgi?targetID=3253887) | 838 | 70 | hsa-miR-15a-5p | [LRP1B](http://www.ncbi.nlm.nih.gov/entrez/query.fcgi?db=gene&cmd=Retrieve&dopt=full_report&list_uids=53353) | LDL receptor related protein 1B |
| [Details](https://mirdb.org/cgi-bin/target_detail.cgi?targetID=3253901) | 839 | 70 | hsa-miR-15a-5p | [POM121](http://www.ncbi.nlm.nih.gov/entrez/query.fcgi?db=gene&cmd=Retrieve&dopt=full_report&list_uids=9883) | POM121 transmembrane nucleoporin |
| [Details](https://mirdb.org/cgi-bin/target_detail.cgi?targetID=3253967) | 840 | 70 | hsa-miR-15a-5p | [ADAMTSL1](http://www.ncbi.nlm.nih.gov/entrez/query.fcgi?db=gene&cmd=Retrieve&dopt=full_report&list_uids=92949) | ADAMTS like 1 |
| [Details](https://mirdb.org/cgi-bin/target_detail.cgi?targetID=3253972) | 841 | 70 | hsa-miR-15a-5p | [SCAF11](http://www.ncbi.nlm.nih.gov/entrez/query.fcgi?db=gene&cmd=Retrieve&dopt=full_report&list_uids=9169) | SR-related CTD associated factor 11 |
| [Details](https://mirdb.org/cgi-bin/target_detail.cgi?targetID=3252639) | 842 | 69 | hsa-miR-15a-5p | [FRY](http://www.ncbi.nlm.nih.gov/entrez/query.fcgi?db=gene&cmd=Retrieve&dopt=full_report&list_uids=10129) | FRY microtubule binding protein |
| [Details](https://mirdb.org/cgi-bin/target_detail.cgi?targetID=3252651) | 843 | 69 | hsa-miR-15a-5p | [PDLIM5](http://www.ncbi.nlm.nih.gov/entrez/query.fcgi?db=gene&cmd=Retrieve&dopt=full_report&list_uids=10611) | PDZ and LIM domain 5 |
| [Details](https://mirdb.org/cgi-bin/target_detail.cgi?targetID=3252677) | 844 | 69 | hsa-miR-15a-5p | [RAPGEFL1](http://www.ncbi.nlm.nih.gov/entrez/query.fcgi?db=gene&cmd=Retrieve&dopt=full_report&list_uids=51195) | Rap guanine nucleotide exchange factor like 1 |
| [Details](https://mirdb.org/cgi-bin/target_detail.cgi?targetID=3252697) | 845 | 69 | hsa-miR-15a-5p | [BTLA](http://www.ncbi.nlm.nih.gov/entrez/query.fcgi?db=gene&cmd=Retrieve&dopt=full_report&list_uids=151888) | B and T lymphocyte associated |
| [Details](https://mirdb.org/cgi-bin/target_detail.cgi?targetID=3252808) | 846 | 69 | hsa-miR-15a-5p | [PEX12](http://www.ncbi.nlm.nih.gov/entrez/query.fcgi?db=gene&cmd=Retrieve&dopt=full_report&list_uids=5193) | peroxisomal biogenesis factor 12 |
| [Details](https://mirdb.org/cgi-bin/target_detail.cgi?targetID=3252817) | 847 | 69 | hsa-miR-15a-5p | [ADCY5](http://www.ncbi.nlm.nih.gov/entrez/query.fcgi?db=gene&cmd=Retrieve&dopt=full_report&list_uids=111) | adenylate cyclase 5 |
| [Details](https://mirdb.org/cgi-bin/target_detail.cgi?targetID=3252834) | 848 | 69 | hsa-miR-15a-5p | [SPSB4](http://www.ncbi.nlm.nih.gov/entrez/query.fcgi?db=gene&cmd=Retrieve&dopt=full_report&list_uids=92369) | splA/ryanodine receptor domain and SOCS box containing 4 |
| [Details](https://mirdb.org/cgi-bin/target_detail.cgi?targetID=3252846) | 849 | 69 | hsa-miR-15a-5p | [APP](http://www.ncbi.nlm.nih.gov/entrez/query.fcgi?db=gene&cmd=Retrieve&dopt=full_report&list_uids=351) | amyloid beta precursor protein |
| [Details](https://mirdb.org/cgi-bin/target_detail.cgi?targetID=3252849) | 850 | 69 | hsa-miR-15a-5p | [CPSF6](http://www.ncbi.nlm.nih.gov/entrez/query.fcgi?db=gene&cmd=Retrieve&dopt=full_report&list_uids=11052) | cleavage and polyadenylation specific factor 6 |
| [Details](https://mirdb.org/cgi-bin/target_detail.cgi?targetID=3252969) | 851 | 69 | hsa-miR-15a-5p | [GRK3](http://www.ncbi.nlm.nih.gov/entrez/query.fcgi?db=gene&cmd=Retrieve&dopt=full_report&list_uids=157) | G protein-coupled receptor kinase 3 |
| [Details](https://mirdb.org/cgi-bin/target_detail.cgi?targetID=3253031) | 852 | 69 | hsa-miR-15a-5p | [RAB40B](http://www.ncbi.nlm.nih.gov/entrez/query.fcgi?db=gene&cmd=Retrieve&dopt=full_report&list_uids=10966) | RAB40B, member RAS oncogene family |
| [Details](https://mirdb.org/cgi-bin/target_detail.cgi?targetID=3253056) | 853 | 69 | hsa-miR-15a-5p | [USP49](http://www.ncbi.nlm.nih.gov/entrez/query.fcgi?db=gene&cmd=Retrieve&dopt=full_report&list_uids=25862) | ubiquitin specific peptidase 49 |
| [Details](https://mirdb.org/cgi-bin/target_detail.cgi?targetID=3253076) | 854 | 69 | hsa-miR-15a-5p | [PLBD2](http://www.ncbi.nlm.nih.gov/entrez/query.fcgi?db=gene&cmd=Retrieve&dopt=full_report&list_uids=196463) | phospholipase B domain containing 2 |
| [Details](https://mirdb.org/cgi-bin/target_detail.cgi?targetID=3253125) | 855 | 69 | hsa-miR-15a-5p | [ZFYVE1](http://www.ncbi.nlm.nih.gov/entrez/query.fcgi?db=gene&cmd=Retrieve&dopt=full_report&list_uids=53349) | zinc finger FYVE-type containing 1 |
| [Details](https://mirdb.org/cgi-bin/target_detail.cgi?targetID=3253189) | 856 | 69 | hsa-miR-15a-5p | [FRMPD1](http://www.ncbi.nlm.nih.gov/entrez/query.fcgi?db=gene&cmd=Retrieve&dopt=full_report&list_uids=22844) | FERM and PDZ domain containing 1 |
| [Details](https://mirdb.org/cgi-bin/target_detail.cgi?targetID=3253264) | 857 | 69 | hsa-miR-15a-5p | [UNC5B](http://www.ncbi.nlm.nih.gov/entrez/query.fcgi?db=gene&cmd=Retrieve&dopt=full_report&list_uids=219699) | unc-5 netrin receptor B |
| [Details](https://mirdb.org/cgi-bin/target_detail.cgi?targetID=3253358) | 858 | 69 | hsa-miR-15a-5p | [WAPL](http://www.ncbi.nlm.nih.gov/entrez/query.fcgi?db=gene&cmd=Retrieve&dopt=full_report&list_uids=23063) | WAPL cohesin release factor |
| [Details](https://mirdb.org/cgi-bin/target_detail.cgi?targetID=3253482) | 859 | 69 | hsa-miR-15a-5p | [OCRL](http://www.ncbi.nlm.nih.gov/entrez/query.fcgi?db=gene&cmd=Retrieve&dopt=full_report&list_uids=4952) | OCRL, inositol polyphosphate-5-phosphatase |
| [Details](https://mirdb.org/cgi-bin/target_detail.cgi?targetID=3253511) | 860 | 69 | hsa-miR-15a-5p | [HSPA8](http://www.ncbi.nlm.nih.gov/entrez/query.fcgi?db=gene&cmd=Retrieve&dopt=full_report&list_uids=3312) | heat shock protein family A (Hsp70) member 8 |
| [Details](https://mirdb.org/cgi-bin/target_detail.cgi?targetID=3253616) | 861 | 69 | hsa-miR-15a-5p | [DYNC1I1](http://www.ncbi.nlm.nih.gov/entrez/query.fcgi?db=gene&cmd=Retrieve&dopt=full_report&list_uids=1780) | dynein cytoplasmic 1 intermediate chain 1 |
| [Details](https://mirdb.org/cgi-bin/target_detail.cgi?targetID=3253634) | 862 | 69 | hsa-miR-15a-5p | [OTUD5](http://www.ncbi.nlm.nih.gov/entrez/query.fcgi?db=gene&cmd=Retrieve&dopt=full_report&list_uids=55593) | OTU deubiquitinase 5 |
| [Details](https://mirdb.org/cgi-bin/target_detail.cgi?targetID=3253704) | 863 | 69 | hsa-miR-15a-5p | [CLSPN](http://www.ncbi.nlm.nih.gov/entrez/query.fcgi?db=gene&cmd=Retrieve&dopt=full_report&list_uids=63967) | claspin |
| [Details](https://mirdb.org/cgi-bin/target_detail.cgi?targetID=3253707) | 864 | 69 | hsa-miR-15a-5p | [RIMS3](http://www.ncbi.nlm.nih.gov/entrez/query.fcgi?db=gene&cmd=Retrieve&dopt=full_report&list_uids=9783) | regulating synaptic membrane exocytosis 3 |
| [Details](https://mirdb.org/cgi-bin/target_detail.cgi?targetID=3253732) | 865 | 69 | hsa-miR-15a-5p | [CCNJ](http://www.ncbi.nlm.nih.gov/entrez/query.fcgi?db=gene&cmd=Retrieve&dopt=full_report&list_uids=54619) | cyclin J |
| [Details](https://mirdb.org/cgi-bin/target_detail.cgi?targetID=3253768) | 866 | 69 | hsa-miR-15a-5p | [EFCAB5](http://www.ncbi.nlm.nih.gov/entrez/query.fcgi?db=gene&cmd=Retrieve&dopt=full_report&list_uids=374786) | EF-hand calcium binding domain 5 |
| [Details](https://mirdb.org/cgi-bin/target_detail.cgi?targetID=3253827) | 867 | 69 | hsa-miR-15a-5p | [COPS7A](http://www.ncbi.nlm.nih.gov/entrez/query.fcgi?db=gene&cmd=Retrieve&dopt=full_report&list_uids=50813) | COP9 signalosome subunit 7A |
| [Details](https://mirdb.org/cgi-bin/target_detail.cgi?targetID=3253960) | 868 | 69 | hsa-miR-15a-5p | [CACNA2D4](http://www.ncbi.nlm.nih.gov/entrez/query.fcgi?db=gene&cmd=Retrieve&dopt=full_report&list_uids=93589) | calcium voltage-gated channel auxiliary subunit alpha2delta 4 |
| [Details](https://mirdb.org/cgi-bin/target_detail.cgi?targetID=3253980) | 869 | 69 | hsa-miR-15a-5p | [KMT2A](http://www.ncbi.nlm.nih.gov/entrez/query.fcgi?db=gene&cmd=Retrieve&dopt=full_report&list_uids=4297) | lysine methyltransferase 2A |
| [Details](https://mirdb.org/cgi-bin/target_detail.cgi?targetID=3253983) | 870 | 69 | hsa-miR-15a-5p | [CDC23](http://www.ncbi.nlm.nih.gov/entrez/query.fcgi?db=gene&cmd=Retrieve&dopt=full_report&list_uids=8697) | cell division cycle 23 |
| [Details](https://mirdb.org/cgi-bin/target_detail.cgi?targetID=3254016) | 871 | 69 | hsa-miR-15a-5p | [ERLIN2](http://www.ncbi.nlm.nih.gov/entrez/query.fcgi?db=gene&cmd=Retrieve&dopt=full_report&list_uids=11160) | ER lipid raft associated 2 |
| [Details](https://mirdb.org/cgi-bin/target_detail.cgi?targetID=3254037) | 872 | 69 | hsa-miR-15a-5p | [NAPEPLD](http://www.ncbi.nlm.nih.gov/entrez/query.fcgi?db=gene&cmd=Retrieve&dopt=full_report&list_uids=222236) | N-acyl phosphatidylethanolamine phospholipase D |
| [Details](https://mirdb.org/cgi-bin/target_detail.cgi?targetID=3252665) | 873 | 68 | hsa-miR-15a-5p | [LRRC27](http://www.ncbi.nlm.nih.gov/entrez/query.fcgi?db=gene&cmd=Retrieve&dopt=full_report&list_uids=80313) | leucine rich repeat containing 27 |
| [Details](https://mirdb.org/cgi-bin/target_detail.cgi?targetID=3252683) | 874 | 68 | hsa-miR-15a-5p | [METTL8](http://www.ncbi.nlm.nih.gov/entrez/query.fcgi?db=gene&cmd=Retrieve&dopt=full_report&list_uids=79828) | methyltransferase like 8 |
| [Details](https://mirdb.org/cgi-bin/target_detail.cgi?targetID=3252758) | 875 | 68 | hsa-miR-15a-5p | [SLITRK1](http://www.ncbi.nlm.nih.gov/entrez/query.fcgi?db=gene&cmd=Retrieve&dopt=full_report&list_uids=114798) | SLIT and NTRK like family member 1 |
| [Details](https://mirdb.org/cgi-bin/target_detail.cgi?targetID=3252813) | 876 | 68 | hsa-miR-15a-5p | [TPM2](http://www.ncbi.nlm.nih.gov/entrez/query.fcgi?db=gene&cmd=Retrieve&dopt=full_report&list_uids=7169) | tropomyosin 2 |
| [Details](https://mirdb.org/cgi-bin/target_detail.cgi?targetID=3252815) | 877 | 68 | hsa-miR-15a-5p | [TNFAIP8L3](http://www.ncbi.nlm.nih.gov/entrez/query.fcgi?db=gene&cmd=Retrieve&dopt=full_report&list_uids=388121) | TNF alpha induced protein 8 like 3 |
| [Details](https://mirdb.org/cgi-bin/target_detail.cgi?targetID=3253042) | 878 | 68 | hsa-miR-15a-5p | [KLHL26](http://www.ncbi.nlm.nih.gov/entrez/query.fcgi?db=gene&cmd=Retrieve&dopt=full_report&list_uids=55295) | kelch like family member 26 |
| [Details](https://mirdb.org/cgi-bin/target_detail.cgi?targetID=3253170) | 879 | 68 | hsa-miR-15a-5p | [BORCS6](http://www.ncbi.nlm.nih.gov/entrez/query.fcgi?db=gene&cmd=Retrieve&dopt=full_report&list_uids=54785) | BLOC-1 related complex subunit 6 |
| [Details](https://mirdb.org/cgi-bin/target_detail.cgi?targetID=3253244) | 880 | 68 | hsa-miR-15a-5p | [DLGAP1](http://www.ncbi.nlm.nih.gov/entrez/query.fcgi?db=gene&cmd=Retrieve&dopt=full_report&list_uids=9229) | DLG associated protein 1 |
| [Details](https://mirdb.org/cgi-bin/target_detail.cgi?targetID=3253248) | 881 | 68 | hsa-miR-15a-5p | [MIPOL1](http://www.ncbi.nlm.nih.gov/entrez/query.fcgi?db=gene&cmd=Retrieve&dopt=full_report&list_uids=145282) | mirror-image polydactyly 1 |
| [Details](https://mirdb.org/cgi-bin/target_detail.cgi?targetID=3253280) | 882 | 68 | hsa-miR-15a-5p | [DACH1](http://www.ncbi.nlm.nih.gov/entrez/query.fcgi?db=gene&cmd=Retrieve&dopt=full_report&list_uids=1602) | dachshund family transcription factor 1 |
| [Details](https://mirdb.org/cgi-bin/target_detail.cgi?targetID=3253317) | 883 | 68 | hsa-miR-15a-5p | [KLF7](http://www.ncbi.nlm.nih.gov/entrez/query.fcgi?db=gene&cmd=Retrieve&dopt=full_report&list_uids=8609) | Kruppel like factor 7 |
| [Details](https://mirdb.org/cgi-bin/target_detail.cgi?targetID=3253334) | 884 | 68 | hsa-miR-15a-5p | [RAB4B](http://www.ncbi.nlm.nih.gov/entrez/query.fcgi?db=gene&cmd=Retrieve&dopt=full_report&list_uids=53916) | RAB4B, member RAS oncogene family |
| [Details](https://mirdb.org/cgi-bin/target_detail.cgi?targetID=3253337) | 885 | 68 | hsa-miR-15a-5p | [ZBTB10](http://www.ncbi.nlm.nih.gov/entrez/query.fcgi?db=gene&cmd=Retrieve&dopt=full_report&list_uids=65986) | zinc finger and BTB domain containing 10 |
| [Details](https://mirdb.org/cgi-bin/target_detail.cgi?targetID=3253502) | 886 | 68 | hsa-miR-15a-5p | [NDP](http://www.ncbi.nlm.nih.gov/entrez/query.fcgi?db=gene&cmd=Retrieve&dopt=full_report&list_uids=4693) | NDP, norrin cystine knot growth factor |
| [Details](https://mirdb.org/cgi-bin/target_detail.cgi?targetID=3253526) | 887 | 68 | hsa-miR-15a-5p | [LRRC7](http://www.ncbi.nlm.nih.gov/entrez/query.fcgi?db=gene&cmd=Retrieve&dopt=full_report&list_uids=57554) | leucine rich repeat containing 7 |
| [Details](https://mirdb.org/cgi-bin/target_detail.cgi?targetID=3253560) | 888 | 68 | hsa-miR-15a-5p | [KCNAB1](http://www.ncbi.nlm.nih.gov/entrez/query.fcgi?db=gene&cmd=Retrieve&dopt=full_report&list_uids=7881) | potassium voltage-gated channel subfamily A member regulatory beta subunit 1 |
| [Details](https://mirdb.org/cgi-bin/target_detail.cgi?targetID=3253650) | 889 | 68 | hsa-miR-15a-5p | [SRPRB](http://www.ncbi.nlm.nih.gov/entrez/query.fcgi?db=gene&cmd=Retrieve&dopt=full_report&list_uids=58477) | SRP receptor subunit beta |
| [Details](https://mirdb.org/cgi-bin/target_detail.cgi?targetID=3253654) | 890 | 68 | hsa-miR-15a-5p | [LDLRAD4](http://www.ncbi.nlm.nih.gov/entrez/query.fcgi?db=gene&cmd=Retrieve&dopt=full_report&list_uids=753) | low density lipoprotein receptor class A domain containing 4 |
| [Details](https://mirdb.org/cgi-bin/target_detail.cgi?targetID=3253673) | 891 | 68 | hsa-miR-15a-5p | [PLPP3](http://www.ncbi.nlm.nih.gov/entrez/query.fcgi?db=gene&cmd=Retrieve&dopt=full_report&list_uids=8613) | phospholipid phosphatase 3 |
| [Details](https://mirdb.org/cgi-bin/target_detail.cgi?targetID=3253680) | 892 | 68 | hsa-miR-15a-5p | [ZBTB43](http://www.ncbi.nlm.nih.gov/entrez/query.fcgi?db=gene&cmd=Retrieve&dopt=full_report&list_uids=23099) | zinc finger and BTB domain containing 43 |
| [Details](https://mirdb.org/cgi-bin/target_detail.cgi?targetID=3253752) | 893 | 68 | hsa-miR-15a-5p | [SGCA](http://www.ncbi.nlm.nih.gov/entrez/query.fcgi?db=gene&cmd=Retrieve&dopt=full_report&list_uids=6442) | sarcoglycan alpha |
| [Details](https://mirdb.org/cgi-bin/target_detail.cgi?targetID=3253779) | 894 | 68 | hsa-miR-15a-5p | [CDC42EP2](http://www.ncbi.nlm.nih.gov/entrez/query.fcgi?db=gene&cmd=Retrieve&dopt=full_report&list_uids=10435) | CDC42 effector protein 2 |
| [Details](https://mirdb.org/cgi-bin/target_detail.cgi?targetID=3253797) | 895 | 68 | hsa-miR-15a-5p | [PHF20](http://www.ncbi.nlm.nih.gov/entrez/query.fcgi?db=gene&cmd=Retrieve&dopt=full_report&list_uids=51230) | PHD finger protein 20 |
| [Details](https://mirdb.org/cgi-bin/target_detail.cgi?targetID=3253884) | 896 | 68 | hsa-miR-15a-5p | [BDNF](http://www.ncbi.nlm.nih.gov/entrez/query.fcgi?db=gene&cmd=Retrieve&dopt=full_report&list_uids=627) | brain derived neurotrophic factor |
| [Details](https://mirdb.org/cgi-bin/target_detail.cgi?targetID=3253896) | 897 | 68 | hsa-miR-15a-5p | [RBMS1](http://www.ncbi.nlm.nih.gov/entrez/query.fcgi?db=gene&cmd=Retrieve&dopt=full_report&list_uids=5937) | RNA binding motif single stranded interacting protein 1 |
| [Details](https://mirdb.org/cgi-bin/target_detail.cgi?targetID=3253997) | 898 | 68 | hsa-miR-15a-5p | [NAT8L](http://www.ncbi.nlm.nih.gov/entrez/query.fcgi?db=gene&cmd=Retrieve&dopt=full_report&list_uids=339983) | N-acetyltransferase 8 like |
| [Details](https://mirdb.org/cgi-bin/target_detail.cgi?targetID=3254038) | 899 | 68 | hsa-miR-15a-5p | [HSPA1B](http://www.ncbi.nlm.nih.gov/entrez/query.fcgi?db=gene&cmd=Retrieve&dopt=full_report&list_uids=3304) | heat shock protein family A (Hsp70) member 1B |
| [Details](https://mirdb.org/cgi-bin/target_detail.cgi?targetID=3252634) | 900 | 67 | hsa-miR-15a-5p | [PPM1K](http://www.ncbi.nlm.nih.gov/entrez/query.fcgi?db=gene&cmd=Retrieve&dopt=full_report&list_uids=152926) | protein phosphatase, Mg2+/Mn2+ dependent 1K |
| [Details](https://mirdb.org/cgi-bin/target_detail.cgi?targetID=3252672) | 901 | 67 | hsa-miR-15a-5p | [FCHSD2](http://www.ncbi.nlm.nih.gov/entrez/query.fcgi?db=gene&cmd=Retrieve&dopt=full_report&list_uids=9873) | FCH and double SH3 domains 2 |
| [Details](https://mirdb.org/cgi-bin/target_detail.cgi?targetID=3252673) | 902 | 67 | hsa-miR-15a-5p | [PCBP4](http://www.ncbi.nlm.nih.gov/entrez/query.fcgi?db=gene&cmd=Retrieve&dopt=full_report&list_uids=57060) | poly(rC) binding protein 4 |
| [Details](https://mirdb.org/cgi-bin/target_detail.cgi?targetID=3252712) | 903 | 67 | hsa-miR-15a-5p | [SENP5](http://www.ncbi.nlm.nih.gov/entrez/query.fcgi?db=gene&cmd=Retrieve&dopt=full_report&list_uids=205564) | SUMO specific peptidase 5 |
| [Details](https://mirdb.org/cgi-bin/target_detail.cgi?targetID=3252725) | 904 | 67 | hsa-miR-15a-5p | [PCDHA8](http://www.ncbi.nlm.nih.gov/entrez/query.fcgi?db=gene&cmd=Retrieve&dopt=full_report&list_uids=56140) | protocadherin alpha 8 |
| [Details](https://mirdb.org/cgi-bin/target_detail.cgi?targetID=3252728) | 905 | 67 | hsa-miR-15a-5p | [PCDHAC1](http://www.ncbi.nlm.nih.gov/entrez/query.fcgi?db=gene&cmd=Retrieve&dopt=full_report&list_uids=56135) | protocadherin alpha subfamily C, 1 |
| [Details](https://mirdb.org/cgi-bin/target_detail.cgi?targetID=3252792) | 906 | 67 | hsa-miR-15a-5p | [DCUN1D1](http://www.ncbi.nlm.nih.gov/entrez/query.fcgi?db=gene&cmd=Retrieve&dopt=full_report&list_uids=54165) | defective in cullin neddylation 1 domain containing 1 |
| [Details](https://mirdb.org/cgi-bin/target_detail.cgi?targetID=3252845) | 907 | 67 | hsa-miR-15a-5p | [ZNF267](http://www.ncbi.nlm.nih.gov/entrez/query.fcgi?db=gene&cmd=Retrieve&dopt=full_report&list_uids=10308) | zinc finger protein 267 |
| [Details](https://mirdb.org/cgi-bin/target_detail.cgi?targetID=3252859) | 908 | 67 | hsa-miR-15a-5p | [SNIP1](http://www.ncbi.nlm.nih.gov/entrez/query.fcgi?db=gene&cmd=Retrieve&dopt=full_report&list_uids=79753) | Smad nuclear interacting protein 1 |
| [Details](https://mirdb.org/cgi-bin/target_detail.cgi?targetID=3252932) | 909 | 67 | hsa-miR-15a-5p | [PCDHAC2](http://www.ncbi.nlm.nih.gov/entrez/query.fcgi?db=gene&cmd=Retrieve&dopt=full_report&list_uids=56134) | protocadherin alpha subfamily C, 2 |
| [Details](https://mirdb.org/cgi-bin/target_detail.cgi?targetID=3252938) | 910 | 67 | hsa-miR-15a-5p | [PCDHA13](http://www.ncbi.nlm.nih.gov/entrez/query.fcgi?db=gene&cmd=Retrieve&dopt=full_report&list_uids=56136) | protocadherin alpha 13 |
| [Details](https://mirdb.org/cgi-bin/target_detail.cgi?targetID=3252941) | 911 | 67 | hsa-miR-15a-5p | [GRB10](http://www.ncbi.nlm.nih.gov/entrez/query.fcgi?db=gene&cmd=Retrieve&dopt=full_report&list_uids=2887) | growth factor receptor bound protein 10 |
| [Details](https://mirdb.org/cgi-bin/target_detail.cgi?targetID=3252994) | 912 | 67 | hsa-miR-15a-5p | [RGS8](http://www.ncbi.nlm.nih.gov/entrez/query.fcgi?db=gene&cmd=Retrieve&dopt=full_report&list_uids=85397) | regulator of G protein signaling 8 |
| [Details](https://mirdb.org/cgi-bin/target_detail.cgi?targetID=3253089) | 913 | 67 | hsa-miR-15a-5p | [ZNF559-ZNF177](http://www.ncbi.nlm.nih.gov/entrez/query.fcgi?db=gene&cmd=Retrieve&dopt=full_report&list_uids=100529215) | ZNF559-ZNF177 readthrough |
| [Details](https://mirdb.org/cgi-bin/target_detail.cgi?targetID=3253120) | 914 | 67 | hsa-miR-15a-5p | [ZNF177](http://www.ncbi.nlm.nih.gov/entrez/query.fcgi?db=gene&cmd=Retrieve&dopt=full_report&list_uids=7730) | zinc finger protein 177 |
| [Details](https://mirdb.org/cgi-bin/target_detail.cgi?targetID=3253131) | 915 | 67 | hsa-miR-15a-5p | [CNNM2](http://www.ncbi.nlm.nih.gov/entrez/query.fcgi?db=gene&cmd=Retrieve&dopt=full_report&list_uids=54805) | cyclin and CBS domain divalent metal cation transport mediator 2 |
| [Details](https://mirdb.org/cgi-bin/target_detail.cgi?targetID=3253150) | 916 | 67 | hsa-miR-15a-5p | [PCDHA10](http://www.ncbi.nlm.nih.gov/entrez/query.fcgi?db=gene&cmd=Retrieve&dopt=full_report&list_uids=56139) | protocadherin alpha 10 |
| [Details](https://mirdb.org/cgi-bin/target_detail.cgi?targetID=3253162) | 917 | 67 | hsa-miR-15a-5p | [PCDHA5](http://www.ncbi.nlm.nih.gov/entrez/query.fcgi?db=gene&cmd=Retrieve&dopt=full_report&list_uids=56143) | protocadherin alpha 5 |
| [Details](https://mirdb.org/cgi-bin/target_detail.cgi?targetID=3253187) | 918 | 67 | hsa-miR-15a-5p | [TASP1](http://www.ncbi.nlm.nih.gov/entrez/query.fcgi?db=gene&cmd=Retrieve&dopt=full_report&list_uids=55617) | taspase 1 |
| [Details](https://mirdb.org/cgi-bin/target_detail.cgi?targetID=3253202) | 919 | 67 | hsa-miR-15a-5p | [GEN1](http://www.ncbi.nlm.nih.gov/entrez/query.fcgi?db=gene&cmd=Retrieve&dopt=full_report&list_uids=348654) | GEN1, Holliday junction 5' flap endonuclease |
| [Details](https://mirdb.org/cgi-bin/target_detail.cgi?targetID=3253263) | 920 | 67 | hsa-miR-15a-5p | [IPO9](http://www.ncbi.nlm.nih.gov/entrez/query.fcgi?db=gene&cmd=Retrieve&dopt=full_report&list_uids=55705) | importin 9 |
| [Details](https://mirdb.org/cgi-bin/target_detail.cgi?targetID=3253271) | 921 | 67 | hsa-miR-15a-5p | [PCDHA6](http://www.ncbi.nlm.nih.gov/entrez/query.fcgi?db=gene&cmd=Retrieve&dopt=full_report&list_uids=56142) | protocadherin alpha 6 |
| [Details](https://mirdb.org/cgi-bin/target_detail.cgi?targetID=3253275) | 922 | 67 | hsa-miR-15a-5p | [COL24A1](http://www.ncbi.nlm.nih.gov/entrez/query.fcgi?db=gene&cmd=Retrieve&dopt=full_report&list_uids=255631) | collagen type XXIV alpha 1 chain |
| [Details](https://mirdb.org/cgi-bin/target_detail.cgi?targetID=3253282) | 923 | 67 | hsa-miR-15a-5p | [CRKL](http://www.ncbi.nlm.nih.gov/entrez/query.fcgi?db=gene&cmd=Retrieve&dopt=full_report&list_uids=1399) | CRK like proto-oncogene, adaptor protein |
| [Details](https://mirdb.org/cgi-bin/target_detail.cgi?targetID=3253323) | 924 | 67 | hsa-miR-15a-5p | [PCDHA11](http://www.ncbi.nlm.nih.gov/entrez/query.fcgi?db=gene&cmd=Retrieve&dopt=full_report&list_uids=56138) | protocadherin alpha 11 |
| [Details](https://mirdb.org/cgi-bin/target_detail.cgi?targetID=3253329) | 925 | 67 | hsa-miR-15a-5p | [YIF1B](http://www.ncbi.nlm.nih.gov/entrez/query.fcgi?db=gene&cmd=Retrieve&dopt=full_report&list_uids=90522) | Yip1 interacting factor homolog B, membrane trafficking protein |
| [Details](https://mirdb.org/cgi-bin/target_detail.cgi?targetID=3253347) | 926 | 67 | hsa-miR-15a-5p | [TTC25](http://www.ncbi.nlm.nih.gov/entrez/query.fcgi?db=gene&cmd=Retrieve&dopt=full_report&list_uids=83538) | tetratricopeptide repeat domain 25 |
| [Details](https://mirdb.org/cgi-bin/target_detail.cgi?targetID=3253374) | 927 | 67 | hsa-miR-15a-5p | [PCDHA3](http://www.ncbi.nlm.nih.gov/entrez/query.fcgi?db=gene&cmd=Retrieve&dopt=full_report&list_uids=56145) | protocadherin alpha 3 |
| [Details](https://mirdb.org/cgi-bin/target_detail.cgi?targetID=3253430) | 928 | 67 | hsa-miR-15a-5p | [KY](http://www.ncbi.nlm.nih.gov/entrez/query.fcgi?db=gene&cmd=Retrieve&dopt=full_report&list_uids=339855) | kyphoscoliosis peptidase |
| [Details](https://mirdb.org/cgi-bin/target_detail.cgi?targetID=3253662) | 929 | 67 | hsa-miR-15a-5p | [PCDHA4](http://www.ncbi.nlm.nih.gov/entrez/query.fcgi?db=gene&cmd=Retrieve&dopt=full_report&list_uids=56144) | protocadherin alpha 4 |
| [Details](https://mirdb.org/cgi-bin/target_detail.cgi?targetID=3253677) | 930 | 67 | hsa-miR-15a-5p | [LYPLAL1](http://www.ncbi.nlm.nih.gov/entrez/query.fcgi?db=gene&cmd=Retrieve&dopt=full_report&list_uids=127018) | lysophospholipase like 1 |
| [Details](https://mirdb.org/cgi-bin/target_detail.cgi?targetID=3253687) | 931 | 67 | hsa-miR-15a-5p | [CDS2](http://www.ncbi.nlm.nih.gov/entrez/query.fcgi?db=gene&cmd=Retrieve&dopt=full_report&list_uids=8760) | CDP-diacylglycerol synthase 2 |
| [Details](https://mirdb.org/cgi-bin/target_detail.cgi?targetID=3253737) | 932 | 67 | hsa-miR-15a-5p | [PCDHA1](http://www.ncbi.nlm.nih.gov/entrez/query.fcgi?db=gene&cmd=Retrieve&dopt=full_report&list_uids=56147) | protocadherin alpha 1 |
| [Details](https://mirdb.org/cgi-bin/target_detail.cgi?targetID=3253833) | 933 | 67 | hsa-miR-15a-5p | [NFS1](http://www.ncbi.nlm.nih.gov/entrez/query.fcgi?db=gene&cmd=Retrieve&dopt=full_report&list_uids=9054) | NFS1, cysteine desulfurase |
| [Details](https://mirdb.org/cgi-bin/target_detail.cgi?targetID=3253862) | 934 | 67 | hsa-miR-15a-5p | [PCDHA7](http://www.ncbi.nlm.nih.gov/entrez/query.fcgi?db=gene&cmd=Retrieve&dopt=full_report&list_uids=56141) | protocadherin alpha 7 |
| [Details](https://mirdb.org/cgi-bin/target_detail.cgi?targetID=3253882) | 935 | 67 | hsa-miR-15a-5p | [PCDHA2](http://www.ncbi.nlm.nih.gov/entrez/query.fcgi?db=gene&cmd=Retrieve&dopt=full_report&list_uids=56146) | protocadherin alpha 2 |
| [Details](https://mirdb.org/cgi-bin/target_detail.cgi?targetID=3253911) | 936 | 67 | hsa-miR-15a-5p | [ODAPH](http://www.ncbi.nlm.nih.gov/entrez/query.fcgi?db=gene&cmd=Retrieve&dopt=full_report&list_uids=152816) | odontogenesis associated phosphoprotein |
| [Details](https://mirdb.org/cgi-bin/target_detail.cgi?targetID=3253923) | 937 | 67 | hsa-miR-15a-5p | [AJUBA](http://www.ncbi.nlm.nih.gov/entrez/query.fcgi?db=gene&cmd=Retrieve&dopt=full_report&list_uids=84962) | ajuba LIM protein |
| [Details](https://mirdb.org/cgi-bin/target_detail.cgi?targetID=3253953) | 938 | 67 | hsa-miR-15a-5p | [PCDHA12](http://www.ncbi.nlm.nih.gov/entrez/query.fcgi?db=gene&cmd=Retrieve&dopt=full_report&list_uids=56137) | protocadherin alpha 12 |
| [Details](https://mirdb.org/cgi-bin/target_detail.cgi?targetID=3252641) | 939 | 66 | hsa-miR-15a-5p | [MED14OS](http://www.ncbi.nlm.nih.gov/entrez/query.fcgi?db=gene&cmd=Retrieve&dopt=full_report&list_uids=100873985) | MED14 opposite strand |
| [Details](https://mirdb.org/cgi-bin/target_detail.cgi?targetID=3252644) | 940 | 66 | hsa-miR-15a-5p | [GATAD2B](http://www.ncbi.nlm.nih.gov/entrez/query.fcgi?db=gene&cmd=Retrieve&dopt=full_report&list_uids=57459) | GATA zinc finger domain containing 2B |
| [Details](https://mirdb.org/cgi-bin/target_detail.cgi?targetID=3252714) | 941 | 66 | hsa-miR-15a-5p | [SLC38A4](http://www.ncbi.nlm.nih.gov/entrez/query.fcgi?db=gene&cmd=Retrieve&dopt=full_report&list_uids=55089) | solute carrier family 38 member 4 |
| [Details](https://mirdb.org/cgi-bin/target_detail.cgi?targetID=3252744) | 942 | 66 | hsa-miR-15a-5p | [C8orf58](http://www.ncbi.nlm.nih.gov/entrez/query.fcgi?db=gene&cmd=Retrieve&dopt=full_report&list_uids=541565) | chromosome 8 open reading frame 58 |
| [Details](https://mirdb.org/cgi-bin/target_detail.cgi?targetID=3252906) | 943 | 66 | hsa-miR-15a-5p | [SIDT2](http://www.ncbi.nlm.nih.gov/entrez/query.fcgi?db=gene&cmd=Retrieve&dopt=full_report&list_uids=51092) | SID1 transmembrane family member 2 |
| [Details](https://mirdb.org/cgi-bin/target_detail.cgi?targetID=3252914) | 944 | 66 | hsa-miR-15a-5p | [SKIL](http://www.ncbi.nlm.nih.gov/entrez/query.fcgi?db=gene&cmd=Retrieve&dopt=full_report&list_uids=6498) | SKI like proto-oncogene |
| [Details](https://mirdb.org/cgi-bin/target_detail.cgi?targetID=3253066) | 945 | 66 | hsa-miR-15a-5p | [ASB1](http://www.ncbi.nlm.nih.gov/entrez/query.fcgi?db=gene&cmd=Retrieve&dopt=full_report&list_uids=51665) | ankyrin repeat and SOCS box containing 1 |
| [Details](https://mirdb.org/cgi-bin/target_detail.cgi?targetID=3253145) | 946 | 66 | hsa-miR-15a-5p | [TBCK](http://www.ncbi.nlm.nih.gov/entrez/query.fcgi?db=gene&cmd=Retrieve&dopt=full_report&list_uids=93627) | TBC1 domain containing kinase |
| [Details](https://mirdb.org/cgi-bin/target_detail.cgi?targetID=3253218) | 947 | 66 | hsa-miR-15a-5p | [PRR13](http://www.ncbi.nlm.nih.gov/entrez/query.fcgi?db=gene&cmd=Retrieve&dopt=full_report&list_uids=54458) | proline rich 13 |
| [Details](https://mirdb.org/cgi-bin/target_detail.cgi?targetID=3253302) | 948 | 66 | hsa-miR-15a-5p | [ATP5MC3](http://www.ncbi.nlm.nih.gov/entrez/query.fcgi?db=gene&cmd=Retrieve&dopt=full_report&list_uids=518) | ATP synthase membrane subunit c locus 3 |
| [Details](https://mirdb.org/cgi-bin/target_detail.cgi?targetID=3253348) | 949 | 66 | hsa-miR-15a-5p | [FCRL2](http://www.ncbi.nlm.nih.gov/entrez/query.fcgi?db=gene&cmd=Retrieve&dopt=full_report&list_uids=79368) | Fc receptor like 2 |
| [Details](https://mirdb.org/cgi-bin/target_detail.cgi?targetID=3253349) | 950 | 66 | hsa-miR-15a-5p | [RAB23](http://www.ncbi.nlm.nih.gov/entrez/query.fcgi?db=gene&cmd=Retrieve&dopt=full_report&list_uids=51715) | RAB23, member RAS oncogene family |
| [Details](https://mirdb.org/cgi-bin/target_detail.cgi?targetID=3253378) | 951 | 66 | hsa-miR-15a-5p | [CXorf40B](http://www.ncbi.nlm.nih.gov/entrez/query.fcgi?db=gene&cmd=Retrieve&dopt=full_report&list_uids=541578) | chromosome X open reading frame 40B |
| [Details](https://mirdb.org/cgi-bin/target_detail.cgi?targetID=3253417) | 952 | 66 | hsa-miR-15a-5p | [UBE2J1](http://www.ncbi.nlm.nih.gov/entrez/query.fcgi?db=gene&cmd=Retrieve&dopt=full_report&list_uids=51465) | ubiquitin conjugating enzyme E2 J1 |
| [Details](https://mirdb.org/cgi-bin/target_detail.cgi?targetID=3253520) | 953 | 66 | hsa-miR-15a-5p | [PDPR](http://www.ncbi.nlm.nih.gov/entrez/query.fcgi?db=gene&cmd=Retrieve&dopt=full_report&list_uids=55066) | pyruvate dehydrogenase phosphatase regulatory subunit |
| [Details](https://mirdb.org/cgi-bin/target_detail.cgi?targetID=3253675) | 954 | 66 | hsa-miR-15a-5p | [ABCG4](http://www.ncbi.nlm.nih.gov/entrez/query.fcgi?db=gene&cmd=Retrieve&dopt=full_report&list_uids=64137) | ATP binding cassette subfamily G member 4 |
| [Details](https://mirdb.org/cgi-bin/target_detail.cgi?targetID=3253715) | 955 | 66 | hsa-miR-15a-5p | [HOXC8](http://www.ncbi.nlm.nih.gov/entrez/query.fcgi?db=gene&cmd=Retrieve&dopt=full_report&list_uids=3224) | homeobox C8 |
| [Details](https://mirdb.org/cgi-bin/target_detail.cgi?targetID=3253813) | 956 | 66 | hsa-miR-15a-5p | [SEC14L4](http://www.ncbi.nlm.nih.gov/entrez/query.fcgi?db=gene&cmd=Retrieve&dopt=full_report&list_uids=284904) | SEC14 like lipid binding 4 |
| [Details](https://mirdb.org/cgi-bin/target_detail.cgi?targetID=3253819) | 957 | 66 | hsa-miR-15a-5p | [RAB35](http://www.ncbi.nlm.nih.gov/entrez/query.fcgi?db=gene&cmd=Retrieve&dopt=full_report&list_uids=11021) | RAB35, member RAS oncogene family |
| [Details](https://mirdb.org/cgi-bin/target_detail.cgi?targetID=3253851) | 958 | 66 | hsa-miR-15a-5p | [CD40](http://www.ncbi.nlm.nih.gov/entrez/query.fcgi?db=gene&cmd=Retrieve&dopt=full_report&list_uids=958) | CD40 molecule |
| [Details](https://mirdb.org/cgi-bin/target_detail.cgi?targetID=3253880) | 959 | 66 | hsa-miR-15a-5p | [FAM189A1](http://www.ncbi.nlm.nih.gov/entrez/query.fcgi?db=gene&cmd=Retrieve&dopt=full_report&list_uids=23359) | family with sequence similarity 189 member A1 |
| [Details](https://mirdb.org/cgi-bin/target_detail.cgi?targetID=3253926) | 960 | 66 | hsa-miR-15a-5p | [PSMA5](http://www.ncbi.nlm.nih.gov/entrez/query.fcgi?db=gene&cmd=Retrieve&dopt=full_report&list_uids=5686) | proteasome subunit alpha 5 |
| [Details](https://mirdb.org/cgi-bin/target_detail.cgi?targetID=3253942) | 961 | 66 | hsa-miR-15a-5p | [CMTM4](http://www.ncbi.nlm.nih.gov/entrez/query.fcgi?db=gene&cmd=Retrieve&dopt=full_report&list_uids=146223) | CKLF like MARVEL transmembrane domain containing 4 |
| [Details](https://mirdb.org/cgi-bin/target_detail.cgi?targetID=3254013) | 962 | 66 | hsa-miR-15a-5p | [UBE3C](http://www.ncbi.nlm.nih.gov/entrez/query.fcgi?db=gene&cmd=Retrieve&dopt=full_report&list_uids=9690) | ubiquitin protein ligase E3C |
| [Details](https://mirdb.org/cgi-bin/target_detail.cgi?targetID=3252647) | 963 | 65 | hsa-miR-15a-5p | [TARBP1](http://www.ncbi.nlm.nih.gov/entrez/query.fcgi?db=gene&cmd=Retrieve&dopt=full_report&list_uids=6894) | TAR (HIV-1) RNA binding protein 1 |
| [Details](https://mirdb.org/cgi-bin/target_detail.cgi?targetID=3252670) | 964 | 65 | hsa-miR-15a-5p | [ESRP1](http://www.ncbi.nlm.nih.gov/entrez/query.fcgi?db=gene&cmd=Retrieve&dopt=full_report&list_uids=54845) | epithelial splicing regulatory protein 1 |
| [Details](https://mirdb.org/cgi-bin/target_detail.cgi?targetID=3252686) | 965 | 65 | hsa-miR-15a-5p | [RYBP](http://www.ncbi.nlm.nih.gov/entrez/query.fcgi?db=gene&cmd=Retrieve&dopt=full_report&list_uids=23429) | RING1 and YY1 binding protein |
| [Details](https://mirdb.org/cgi-bin/target_detail.cgi?targetID=3252776) | 966 | 65 | hsa-miR-15a-5p | [UBAC1](http://www.ncbi.nlm.nih.gov/entrez/query.fcgi?db=gene&cmd=Retrieve&dopt=full_report&list_uids=10422) | UBA domain containing 1 |
| [Details](https://mirdb.org/cgi-bin/target_detail.cgi?targetID=3252829) | 967 | 65 | hsa-miR-15a-5p | [ODF2L](http://www.ncbi.nlm.nih.gov/entrez/query.fcgi?db=gene&cmd=Retrieve&dopt=full_report&list_uids=57489) | outer dense fiber of sperm tails 2 like |
| [Details](https://mirdb.org/cgi-bin/target_detail.cgi?targetID=3252842) | 968 | 65 | hsa-miR-15a-5p | [CD28](http://www.ncbi.nlm.nih.gov/entrez/query.fcgi?db=gene&cmd=Retrieve&dopt=full_report&list_uids=940) | CD28 molecule |
| [Details](https://mirdb.org/cgi-bin/target_detail.cgi?targetID=3252883) | 969 | 65 | hsa-miR-15a-5p | [ZBTB7A](http://www.ncbi.nlm.nih.gov/entrez/query.fcgi?db=gene&cmd=Retrieve&dopt=full_report&list_uids=51341) | zinc finger and BTB domain containing 7A |
| [Details](https://mirdb.org/cgi-bin/target_detail.cgi?targetID=3252956) | 970 | 65 | hsa-miR-15a-5p | [GNA12](http://www.ncbi.nlm.nih.gov/entrez/query.fcgi?db=gene&cmd=Retrieve&dopt=full_report&list_uids=2768) | G protein subunit alpha 12 |
| [Details](https://mirdb.org/cgi-bin/target_detail.cgi?targetID=3252971) | 971 | 65 | hsa-miR-15a-5p | [FOSL1](http://www.ncbi.nlm.nih.gov/entrez/query.fcgi?db=gene&cmd=Retrieve&dopt=full_report&list_uids=8061) | FOS like 1, AP-1 transcription factor subunit |
| [Details](https://mirdb.org/cgi-bin/target_detail.cgi?targetID=3252983) | 972 | 65 | hsa-miR-15a-5p | [TSC1](http://www.ncbi.nlm.nih.gov/entrez/query.fcgi?db=gene&cmd=Retrieve&dopt=full_report&list_uids=7248) | TSC complex subunit 1 |
| [Details](https://mirdb.org/cgi-bin/target_detail.cgi?targetID=3253038) | 973 | 65 | hsa-miR-15a-5p | [ANKRD13B](http://www.ncbi.nlm.nih.gov/entrez/query.fcgi?db=gene&cmd=Retrieve&dopt=full_report&list_uids=124930) | ankyrin repeat domain 13B |
| [Details](https://mirdb.org/cgi-bin/target_detail.cgi?targetID=3253081) | 974 | 65 | hsa-miR-15a-5p | [AP2B1](http://www.ncbi.nlm.nih.gov/entrez/query.fcgi?db=gene&cmd=Retrieve&dopt=full_report&list_uids=163) | adaptor related protein complex 2 subunit beta 1 |
| [Details](https://mirdb.org/cgi-bin/target_detail.cgi?targetID=3253144) | 975 | 65 | hsa-miR-15a-5p | [AQP11](http://www.ncbi.nlm.nih.gov/entrez/query.fcgi?db=gene&cmd=Retrieve&dopt=full_report&list_uids=282679) | aquaporin 11 |
| [Details](https://mirdb.org/cgi-bin/target_detail.cgi?targetID=3253148) | 976 | 65 | hsa-miR-15a-5p | [RDH13](http://www.ncbi.nlm.nih.gov/entrez/query.fcgi?db=gene&cmd=Retrieve&dopt=full_report&list_uids=112724) | retinol dehydrogenase 13 |
| [Details](https://mirdb.org/cgi-bin/target_detail.cgi?targetID=3253193) | 977 | 65 | hsa-miR-15a-5p | [ASTN1](http://www.ncbi.nlm.nih.gov/entrez/query.fcgi?db=gene&cmd=Retrieve&dopt=full_report&list_uids=460) | astrotactin 1 |
| [Details](https://mirdb.org/cgi-bin/target_detail.cgi?targetID=3253305) | 978 | 65 | hsa-miR-15a-5p | [RNF138](http://www.ncbi.nlm.nih.gov/entrez/query.fcgi?db=gene&cmd=Retrieve&dopt=full_report&list_uids=51444) | ring finger protein 138 |
| [Details](https://mirdb.org/cgi-bin/target_detail.cgi?targetID=3253369) | 979 | 65 | hsa-miR-15a-5p | [BACE1](http://www.ncbi.nlm.nih.gov/entrez/query.fcgi?db=gene&cmd=Retrieve&dopt=full_report&list_uids=23621) | beta-secretase 1 |
| [Details](https://mirdb.org/cgi-bin/target_detail.cgi?targetID=3253383) | 980 | 65 | hsa-miR-15a-5p | [ZER1](http://www.ncbi.nlm.nih.gov/entrez/query.fcgi?db=gene&cmd=Retrieve&dopt=full_report&list_uids=10444) | zyg-11 related cell cycle regulator |
| [Details](https://mirdb.org/cgi-bin/target_detail.cgi?targetID=3253384) | 981 | 65 | hsa-miR-15a-5p | [TMEM143](http://www.ncbi.nlm.nih.gov/entrez/query.fcgi?db=gene&cmd=Retrieve&dopt=full_report&list_uids=55260) | transmembrane protein 143 |
| [Details](https://mirdb.org/cgi-bin/target_detail.cgi?targetID=3253401) | 982 | 65 | hsa-miR-15a-5p | [BMX](http://www.ncbi.nlm.nih.gov/entrez/query.fcgi?db=gene&cmd=Retrieve&dopt=full_report&list_uids=660) | BMX non-receptor tyrosine kinase |
| [Details](https://mirdb.org/cgi-bin/target_detail.cgi?targetID=3253403) | 983 | 65 | hsa-miR-15a-5p | [RHPN2](http://www.ncbi.nlm.nih.gov/entrez/query.fcgi?db=gene&cmd=Retrieve&dopt=full_report&list_uids=85415) | rhophilin Rho GTPase binding protein 2 |
| [Details](https://mirdb.org/cgi-bin/target_detail.cgi?targetID=3253408) | 984 | 65 | hsa-miR-15a-5p | [TEX19](http://www.ncbi.nlm.nih.gov/entrez/query.fcgi?db=gene&cmd=Retrieve&dopt=full_report&list_uids=400629) | testis expressed 19 |
| [Details](https://mirdb.org/cgi-bin/target_detail.cgi?targetID=3253471) | 985 | 65 | hsa-miR-15a-5p | [CYB561D1](http://www.ncbi.nlm.nih.gov/entrez/query.fcgi?db=gene&cmd=Retrieve&dopt=full_report&list_uids=284613) | cytochrome b561 family member D1 |
| [Details](https://mirdb.org/cgi-bin/target_detail.cgi?targetID=3253472) | 986 | 65 | hsa-miR-15a-5p | [PPIL1](http://www.ncbi.nlm.nih.gov/entrez/query.fcgi?db=gene&cmd=Retrieve&dopt=full_report&list_uids=51645) | peptidylprolyl isomerase like 1 |
| [Details](https://mirdb.org/cgi-bin/target_detail.cgi?targetID=3253532) | 987 | 65 | hsa-miR-15a-5p | [ITPR1](http://www.ncbi.nlm.nih.gov/entrez/query.fcgi?db=gene&cmd=Retrieve&dopt=full_report&list_uids=3708) | inositol 1,4,5-trisphosphate receptor type 1 |
| [Details](https://mirdb.org/cgi-bin/target_detail.cgi?targetID=3253629) | 988 | 65 | hsa-miR-15a-5p | [TAOK1](http://www.ncbi.nlm.nih.gov/entrez/query.fcgi?db=gene&cmd=Retrieve&dopt=full_report&list_uids=57551) | TAO kinase 1 |
| [Details](https://mirdb.org/cgi-bin/target_detail.cgi?targetID=3253751) | 989 | 65 | hsa-miR-15a-5p | [NHSL1](http://www.ncbi.nlm.nih.gov/entrez/query.fcgi?db=gene&cmd=Retrieve&dopt=full_report&list_uids=57224) | NHS like 1 |
| [Details](https://mirdb.org/cgi-bin/target_detail.cgi?targetID=3253759) | 990 | 65 | hsa-miR-15a-5p | [CCDC18](http://www.ncbi.nlm.nih.gov/entrez/query.fcgi?db=gene&cmd=Retrieve&dopt=full_report&list_uids=343099) | coiled-coil domain containing 18 |
| [Details](https://mirdb.org/cgi-bin/target_detail.cgi?targetID=3253818) | 991 | 65 | hsa-miR-15a-5p | [LGI2](http://www.ncbi.nlm.nih.gov/entrez/query.fcgi?db=gene&cmd=Retrieve&dopt=full_report&list_uids=55203) | leucine rich repeat LGI family member 2 |
| [Details](https://mirdb.org/cgi-bin/target_detail.cgi?targetID=3252684) | 992 | 64 | hsa-miR-15a-5p | [KCNIP1](http://www.ncbi.nlm.nih.gov/entrez/query.fcgi?db=gene&cmd=Retrieve&dopt=full_report&list_uids=30820) | potassium voltage-gated channel interacting protein 1 |
| [Details](https://mirdb.org/cgi-bin/target_detail.cgi?targetID=3252768) | 993 | 64 | hsa-miR-15a-5p | [VWA8](http://www.ncbi.nlm.nih.gov/entrez/query.fcgi?db=gene&cmd=Retrieve&dopt=full_report&list_uids=23078) | von Willebrand factor A domain containing 8 |
| [Details](https://mirdb.org/cgi-bin/target_detail.cgi?targetID=3252830) | 994 | 64 | hsa-miR-15a-5p | [SMPD1](http://www.ncbi.nlm.nih.gov/entrez/query.fcgi?db=gene&cmd=Retrieve&dopt=full_report&list_uids=6609) | sphingomyelin phosphodiesterase 1 |
| [Details](https://mirdb.org/cgi-bin/target_detail.cgi?targetID=3252987) | 995 | 64 | hsa-miR-15a-5p | [PLEKHA5](http://www.ncbi.nlm.nih.gov/entrez/query.fcgi?db=gene&cmd=Retrieve&dopt=full_report&list_uids=54477) | pleckstrin homology domain containing A5 |
| [Details](https://mirdb.org/cgi-bin/target_detail.cgi?targetID=3252992) | 996 | 64 | hsa-miR-15a-5p | [SLITRK6](http://www.ncbi.nlm.nih.gov/entrez/query.fcgi?db=gene&cmd=Retrieve&dopt=full_report&list_uids=84189) | SLIT and NTRK like family member 6 |
| [Details](https://mirdb.org/cgi-bin/target_detail.cgi?targetID=3253006) | 997 | 64 | hsa-miR-15a-5p | [OTUB2](http://www.ncbi.nlm.nih.gov/entrez/query.fcgi?db=gene&cmd=Retrieve&dopt=full_report&list_uids=78990) | OTU deubiquitinase, ubiquitin aldehyde binding 2 |
| [Details](https://mirdb.org/cgi-bin/target_detail.cgi?targetID=3253011) | 998 | 64 | hsa-miR-15a-5p | [RBSN](http://www.ncbi.nlm.nih.gov/entrez/query.fcgi?db=gene&cmd=Retrieve&dopt=full_report&list_uids=64145) | rabenosyn, RAB effector |
| [Details](https://mirdb.org/cgi-bin/target_detail.cgi?targetID=3253029) | 999 | 64 | hsa-miR-15a-5p | [PHIP](http://www.ncbi.nlm.nih.gov/entrez/query.fcgi?db=gene&cmd=Retrieve&dopt=full_report&list_uids=55023) | pleckstrin homology domain interacting protein |
| [Details](https://mirdb.org/cgi-bin/target_detail.cgi?targetID=3253043) | 1000 | 64 | hsa-miR-15a-5p | [MAP2K3](http://www.ncbi.nlm.nih.gov/entrez/query.fcgi?db=gene&cmd=Retrieve&dopt=full_report&list_uids=5606) | mitogen-activated protein kinase kinase 3 |
| [Details](https://mirdb.org/cgi-bin/target_detail.cgi?targetID=3253045) | 1001 | 64 | hsa-miR-15a-5p | [EIF4E](http://www.ncbi.nlm.nih.gov/entrez/query.fcgi?db=gene&cmd=Retrieve&dopt=full_report&list_uids=1977) | eukaryotic translation initiation factor 4E |
| [Details](https://mirdb.org/cgi-bin/target_detail.cgi?targetID=3253057) | 1002 | 64 | hsa-miR-15a-5p | [LAMTOR4](http://www.ncbi.nlm.nih.gov/entrez/query.fcgi?db=gene&cmd=Retrieve&dopt=full_report&list_uids=389541) | late endosomal/lysosomal adaptor, MAPK and MTOR activator 4 |
| [Details](https://mirdb.org/cgi-bin/target_detail.cgi?targetID=3253174) | 1003 | 64 | hsa-miR-15a-5p | [AP1S2](http://www.ncbi.nlm.nih.gov/entrez/query.fcgi?db=gene&cmd=Retrieve&dopt=full_report&list_uids=8905) | adaptor related protein complex 1 subunit sigma 2 |
| [Details](https://mirdb.org/cgi-bin/target_detail.cgi?targetID=3253273) | 1004 | 64 | hsa-miR-15a-5p | [ORC4](http://www.ncbi.nlm.nih.gov/entrez/query.fcgi?db=gene&cmd=Retrieve&dopt=full_report&list_uids=5000) | origin recognition complex subunit 4 |
| [Details](https://mirdb.org/cgi-bin/target_detail.cgi?targetID=3253291) | 1005 | 64 | hsa-miR-15a-5p | [CYB561](http://www.ncbi.nlm.nih.gov/entrez/query.fcgi?db=gene&cmd=Retrieve&dopt=full_report&list_uids=1534) | cytochrome b561 |
| [Details](https://mirdb.org/cgi-bin/target_detail.cgi?targetID=3253297) | 1006 | 64 | hsa-miR-15a-5p | [HARS2](http://www.ncbi.nlm.nih.gov/entrez/query.fcgi?db=gene&cmd=Retrieve&dopt=full_report&list_uids=23438) | histidyl-tRNA synthetase 2, mitochondrial |
| [Details](https://mirdb.org/cgi-bin/target_detail.cgi?targetID=3253339) | 1007 | 64 | hsa-miR-15a-5p | [DPP8](http://www.ncbi.nlm.nih.gov/entrez/query.fcgi?db=gene&cmd=Retrieve&dopt=full_report&list_uids=54878) | dipeptidyl peptidase 8 |
| [Details](https://mirdb.org/cgi-bin/target_detail.cgi?targetID=3253479) | 1008 | 64 | hsa-miR-15a-5p | [PPP1R2](http://www.ncbi.nlm.nih.gov/entrez/query.fcgi?db=gene&cmd=Retrieve&dopt=full_report&list_uids=5504) | protein phosphatase 1 regulatory inhibitor subunit 2 |
| [Details](https://mirdb.org/cgi-bin/target_detail.cgi?targetID=3253503) | 1009 | 64 | hsa-miR-15a-5p | [RAP2C](http://www.ncbi.nlm.nih.gov/entrez/query.fcgi?db=gene&cmd=Retrieve&dopt=full_report&list_uids=57826) | RAP2C, member of RAS oncogene family |
| [Details](https://mirdb.org/cgi-bin/target_detail.cgi?targetID=3253504) | 1010 | 64 | hsa-miR-15a-5p | [ACACB](http://www.ncbi.nlm.nih.gov/entrez/query.fcgi?db=gene&cmd=Retrieve&dopt=full_report&list_uids=32) | acetyl-CoA carboxylase beta |
| [Details](https://mirdb.org/cgi-bin/target_detail.cgi?targetID=3253529) | 1011 | 64 | hsa-miR-15a-5p | [CRACR2B](http://www.ncbi.nlm.nih.gov/entrez/query.fcgi?db=gene&cmd=Retrieve&dopt=full_report&list_uids=283229) | calcium release activated channel regulator 2B |
| [Details](https://mirdb.org/cgi-bin/target_detail.cgi?targetID=3253671) | 1012 | 64 | hsa-miR-15a-5p | [GLCE](http://www.ncbi.nlm.nih.gov/entrez/query.fcgi?db=gene&cmd=Retrieve&dopt=full_report&list_uids=26035) | glucuronic acid epimerase |
| [Details](https://mirdb.org/cgi-bin/target_detail.cgi?targetID=3253805) | 1013 | 64 | hsa-miR-15a-5p | [ELAVL4](http://www.ncbi.nlm.nih.gov/entrez/query.fcgi?db=gene&cmd=Retrieve&dopt=full_report&list_uids=1996) | ELAV like RNA binding protein 4 |
| [Details](https://mirdb.org/cgi-bin/target_detail.cgi?targetID=3253842) | 1014 | 64 | hsa-miR-15a-5p | [KLHL15](http://www.ncbi.nlm.nih.gov/entrez/query.fcgi?db=gene&cmd=Retrieve&dopt=full_report&list_uids=80311) | kelch like family member 15 |
| [Details](https://mirdb.org/cgi-bin/target_detail.cgi?targetID=3253951) | 1015 | 64 | hsa-miR-15a-5p | [TMPRSS15](http://www.ncbi.nlm.nih.gov/entrez/query.fcgi?db=gene&cmd=Retrieve&dopt=full_report&list_uids=5651) | transmembrane serine protease 15 |
| [Details](https://mirdb.org/cgi-bin/target_detail.cgi?targetID=3254039) | 1016 | 64 | hsa-miR-15a-5p | [GRB2](http://www.ncbi.nlm.nih.gov/entrez/query.fcgi?db=gene&cmd=Retrieve&dopt=full_report&list_uids=2885) | growth factor receptor bound protein 2 |
| [Details](https://mirdb.org/cgi-bin/target_detail.cgi?targetID=3252694) | 1017 | 63 | hsa-miR-15a-5p | [PSMD7](http://www.ncbi.nlm.nih.gov/entrez/query.fcgi?db=gene&cmd=Retrieve&dopt=full_report&list_uids=5713) | proteasome 26S subunit, non-ATPase 7 |
| [Details](https://mirdb.org/cgi-bin/target_detail.cgi?targetID=3252778) | 1018 | 63 | hsa-miR-15a-5p | [KCNC2](http://www.ncbi.nlm.nih.gov/entrez/query.fcgi?db=gene&cmd=Retrieve&dopt=full_report&list_uids=3747) | potassium voltage-gated channel subfamily C member 2 |
| [Details](https://mirdb.org/cgi-bin/target_detail.cgi?targetID=3252926) | 1019 | 63 | hsa-miR-15a-5p | [SLC4A8](http://www.ncbi.nlm.nih.gov/entrez/query.fcgi?db=gene&cmd=Retrieve&dopt=full_report&list_uids=9498) | solute carrier family 4 member 8 |
| [Details](https://mirdb.org/cgi-bin/target_detail.cgi?targetID=3252993) | 1020 | 63 | hsa-miR-15a-5p | [FRYL](http://www.ncbi.nlm.nih.gov/entrez/query.fcgi?db=gene&cmd=Retrieve&dopt=full_report&list_uids=285527) | FRY like transcription coactivator |
| [Details](https://mirdb.org/cgi-bin/target_detail.cgi?targetID=3252997) | 1021 | 63 | hsa-miR-15a-5p | [GABRA1](http://www.ncbi.nlm.nih.gov/entrez/query.fcgi?db=gene&cmd=Retrieve&dopt=full_report&list_uids=2554) | gamma-aminobutyric acid type A receptor alpha1 subunit |
| [Details](https://mirdb.org/cgi-bin/target_detail.cgi?targetID=3253007) | 1022 | 63 | hsa-miR-15a-5p | [CCNYL1](http://www.ncbi.nlm.nih.gov/entrez/query.fcgi?db=gene&cmd=Retrieve&dopt=full_report&list_uids=151195) | cyclin Y like 1 |
| [Details](https://mirdb.org/cgi-bin/target_detail.cgi?targetID=3253136) | 1023 | 63 | hsa-miR-15a-5p | [CLU](http://www.ncbi.nlm.nih.gov/entrez/query.fcgi?db=gene&cmd=Retrieve&dopt=full_report&list_uids=1191) | clusterin |
| [Details](https://mirdb.org/cgi-bin/target_detail.cgi?targetID=3253210) | 1024 | 63 | hsa-miR-15a-5p | [ATP4B](http://www.ncbi.nlm.nih.gov/entrez/query.fcgi?db=gene&cmd=Retrieve&dopt=full_report&list_uids=496) | ATPase H+/K+ transporting subunit beta |
| [Details](https://mirdb.org/cgi-bin/target_detail.cgi?targetID=3253293) | 1025 | 63 | hsa-miR-15a-5p | [CCDC28A](http://www.ncbi.nlm.nih.gov/entrez/query.fcgi?db=gene&cmd=Retrieve&dopt=full_report&list_uids=25901) | coiled-coil domain containing 28A |
| [Details](https://mirdb.org/cgi-bin/target_detail.cgi?targetID=3253440) | 1026 | 63 | hsa-miR-15a-5p | [B4GALT1](http://www.ncbi.nlm.nih.gov/entrez/query.fcgi?db=gene&cmd=Retrieve&dopt=full_report&list_uids=2683) | beta-1,4-galactosyltransferase 1 |
| [Details](https://mirdb.org/cgi-bin/target_detail.cgi?targetID=3253460) | 1027 | 63 | hsa-miR-15a-5p | [TREM1](http://www.ncbi.nlm.nih.gov/entrez/query.fcgi?db=gene&cmd=Retrieve&dopt=full_report&list_uids=54210) | triggering receptor expressed on myeloid cells 1 |
| [Details](https://mirdb.org/cgi-bin/target_detail.cgi?targetID=3253484) | 1028 | 63 | hsa-miR-15a-5p | [VSIR](http://www.ncbi.nlm.nih.gov/entrez/query.fcgi?db=gene&cmd=Retrieve&dopt=full_report&list_uids=64115) | V-set immunoregulatory receptor |
| [Details](https://mirdb.org/cgi-bin/target_detail.cgi?targetID=3253487) | 1029 | 63 | hsa-miR-15a-5p | [BTN1A1](http://www.ncbi.nlm.nih.gov/entrez/query.fcgi?db=gene&cmd=Retrieve&dopt=full_report&list_uids=696) | butyrophilin subfamily 1 member A1 |
| [Details](https://mirdb.org/cgi-bin/target_detail.cgi?targetID=3253525) | 1030 | 63 | hsa-miR-15a-5p | [ADORA2A](http://www.ncbi.nlm.nih.gov/entrez/query.fcgi?db=gene&cmd=Retrieve&dopt=full_report&list_uids=135) | adenosine A2a receptor |
| [Details](https://mirdb.org/cgi-bin/target_detail.cgi?targetID=3253592) | 1031 | 63 | hsa-miR-15a-5p | [ZNF436](http://www.ncbi.nlm.nih.gov/entrez/query.fcgi?db=gene&cmd=Retrieve&dopt=full_report&list_uids=80818) | zinc finger protein 436 |
| [Details](https://mirdb.org/cgi-bin/target_detail.cgi?targetID=3253605) | 1032 | 63 | hsa-miR-15a-5p | [UMOD](http://www.ncbi.nlm.nih.gov/entrez/query.fcgi?db=gene&cmd=Retrieve&dopt=full_report&list_uids=7369) | uromodulin |
| [Details](https://mirdb.org/cgi-bin/target_detail.cgi?targetID=3253736) | 1033 | 63 | hsa-miR-15a-5p | [C8orf86](http://www.ncbi.nlm.nih.gov/entrez/query.fcgi?db=gene&cmd=Retrieve&dopt=full_report&list_uids=389649) | chromosome 8 open reading frame 86 |
| [Details](https://mirdb.org/cgi-bin/target_detail.cgi?targetID=3253780) | 1034 | 63 | hsa-miR-15a-5p | [TATDN3](http://www.ncbi.nlm.nih.gov/entrez/query.fcgi?db=gene&cmd=Retrieve&dopt=full_report&list_uids=128387) | TatD DNase domain containing 3 |
| [Details](https://mirdb.org/cgi-bin/target_detail.cgi?targetID=3253807) | 1035 | 63 | hsa-miR-15a-5p | [TBC1D9](http://www.ncbi.nlm.nih.gov/entrez/query.fcgi?db=gene&cmd=Retrieve&dopt=full_report&list_uids=23158) | TBC1 domain family member 9 |
| [Details](https://mirdb.org/cgi-bin/target_detail.cgi?targetID=3253908) | 1036 | 63 | hsa-miR-15a-5p | [CCDC179](http://www.ncbi.nlm.nih.gov/entrez/query.fcgi?db=gene&cmd=Retrieve&dopt=full_report&list_uids=100500938) | coiled-coil domain containing 179 |
| [Details](https://mirdb.org/cgi-bin/target_detail.cgi?targetID=3253932) | 1037 | 63 | hsa-miR-15a-5p | [MIEF2](http://www.ncbi.nlm.nih.gov/entrez/query.fcgi?db=gene&cmd=Retrieve&dopt=full_report&list_uids=125170) | mitochondrial elongation factor 2 |
| [Details](https://mirdb.org/cgi-bin/target_detail.cgi?targetID=3253937) | 1038 | 63 | hsa-miR-15a-5p | [BRK1](http://www.ncbi.nlm.nih.gov/entrez/query.fcgi?db=gene&cmd=Retrieve&dopt=full_report&list_uids=55845) | BRICK1, SCAR/WAVE actin nucleating complex subunit |
| [Details](https://mirdb.org/cgi-bin/target_detail.cgi?targetID=3253938) | 1039 | 63 | hsa-miR-15a-5p | [TUBA1A](http://www.ncbi.nlm.nih.gov/entrez/query.fcgi?db=gene&cmd=Retrieve&dopt=full_report&list_uids=7846) | tubulin alpha 1a |
| [Details](https://mirdb.org/cgi-bin/target_detail.cgi?targetID=3253957) | 1040 | 63 | hsa-miR-15a-5p | [ELMO2](http://www.ncbi.nlm.nih.gov/entrez/query.fcgi?db=gene&cmd=Retrieve&dopt=full_report&list_uids=63916) | engulfment and cell motility 2 |
| [Details](https://mirdb.org/cgi-bin/target_detail.cgi?targetID=3253965) | 1041 | 63 | hsa-miR-15a-5p | [PTPN14](http://www.ncbi.nlm.nih.gov/entrez/query.fcgi?db=gene&cmd=Retrieve&dopt=full_report&list_uids=5784) | protein tyrosine phosphatase, non-receptor type 14 |
| [Details](https://mirdb.org/cgi-bin/target_detail.cgi?targetID=3252676) | 1042 | 62 | hsa-miR-15a-5p | [FSTL4](http://www.ncbi.nlm.nih.gov/entrez/query.fcgi?db=gene&cmd=Retrieve&dopt=full_report&list_uids=23105) | follistatin like 4 |
| [Details](https://mirdb.org/cgi-bin/target_detail.cgi?targetID=3252710) | 1043 | 62 | hsa-miR-15a-5p | [PTCD3](http://www.ncbi.nlm.nih.gov/entrez/query.fcgi?db=gene&cmd=Retrieve&dopt=full_report&list_uids=55037) | pentatricopeptide repeat domain 3 |
| [Details](https://mirdb.org/cgi-bin/target_detail.cgi?targetID=3252731) | 1044 | 62 | hsa-miR-15a-5p | [HIPK3](http://www.ncbi.nlm.nih.gov/entrez/query.fcgi?db=gene&cmd=Retrieve&dopt=full_report&list_uids=10114) | homeodomain interacting protein kinase 3 |
| [Details](https://mirdb.org/cgi-bin/target_detail.cgi?targetID=3252805) | 1045 | 62 | hsa-miR-15a-5p | [WASL](http://www.ncbi.nlm.nih.gov/entrez/query.fcgi?db=gene&cmd=Retrieve&dopt=full_report&list_uids=8976) | Wiskott-Aldrich syndrome like |
| [Details](https://mirdb.org/cgi-bin/target_detail.cgi?targetID=3252806) | 1046 | 62 | hsa-miR-15a-5p | [NFRKB](http://www.ncbi.nlm.nih.gov/entrez/query.fcgi?db=gene&cmd=Retrieve&dopt=full_report&list_uids=4798) | nuclear factor related to kappaB binding protein |
| [Details](https://mirdb.org/cgi-bin/target_detail.cgi?targetID=3252865) | 1047 | 62 | hsa-miR-15a-5p | [FCHSD1](http://www.ncbi.nlm.nih.gov/entrez/query.fcgi?db=gene&cmd=Retrieve&dopt=full_report&list_uids=89848) | FCH and double SH3 domains 1 |
| [Details](https://mirdb.org/cgi-bin/target_detail.cgi?targetID=3252960) | 1048 | 62 | hsa-miR-15a-5p | [PGM2L1](http://www.ncbi.nlm.nih.gov/entrez/query.fcgi?db=gene&cmd=Retrieve&dopt=full_report&list_uids=283209) | phosphoglucomutase 2 like 1 |
| [Details](https://mirdb.org/cgi-bin/target_detail.cgi?targetID=3253018) | 1049 | 62 | hsa-miR-15a-5p | [ARRDC4](http://www.ncbi.nlm.nih.gov/entrez/query.fcgi?db=gene&cmd=Retrieve&dopt=full_report&list_uids=91947) | arrestin domain containing 4 |
| [Details](https://mirdb.org/cgi-bin/target_detail.cgi?targetID=3253027) | 1050 | 62 | hsa-miR-15a-5p | [ITGA10](http://www.ncbi.nlm.nih.gov/entrez/query.fcgi?db=gene&cmd=Retrieve&dopt=full_report&list_uids=8515) | integrin subunit alpha 10 |
| [Details](https://mirdb.org/cgi-bin/target_detail.cgi?targetID=3253114) | 1051 | 62 | hsa-miR-15a-5p | [MYLK4](http://www.ncbi.nlm.nih.gov/entrez/query.fcgi?db=gene&cmd=Retrieve&dopt=full_report&list_uids=340156) | myosin light chain kinase family member 4 |
| [Details](https://mirdb.org/cgi-bin/target_detail.cgi?targetID=3253143) | 1052 | 62 | hsa-miR-15a-5p | [PLAGL1](http://www.ncbi.nlm.nih.gov/entrez/query.fcgi?db=gene&cmd=Retrieve&dopt=full_report&list_uids=5325) | PLAG1 like zinc finger 1 |
| [Details](https://mirdb.org/cgi-bin/target_detail.cgi?targetID=3253178) | 1053 | 62 | hsa-miR-15a-5p | [SSRP1](http://www.ncbi.nlm.nih.gov/entrez/query.fcgi?db=gene&cmd=Retrieve&dopt=full_report&list_uids=6749) | structure specific recognition protein 1 |
| [Details](https://mirdb.org/cgi-bin/target_detail.cgi?targetID=3253249) | 1054 | 62 | hsa-miR-15a-5p | [RAD9A](http://www.ncbi.nlm.nih.gov/entrez/query.fcgi?db=gene&cmd=Retrieve&dopt=full_report&list_uids=5883) | RAD9 checkpoint clamp component A |
| [Details](https://mirdb.org/cgi-bin/target_detail.cgi?targetID=3253377) | 1055 | 62 | hsa-miR-15a-5p | [RAB3IP](http://www.ncbi.nlm.nih.gov/entrez/query.fcgi?db=gene&cmd=Retrieve&dopt=full_report&list_uids=117177) | RAB3A interacting protein |
| [Details](https://mirdb.org/cgi-bin/target_detail.cgi?targetID=3253387) | 1056 | 62 | hsa-miR-15a-5p | [NFE2L1](http://www.ncbi.nlm.nih.gov/entrez/query.fcgi?db=gene&cmd=Retrieve&dopt=full_report&list_uids=4779) | nuclear factor, erythroid 2 like 1 |
| [Details](https://mirdb.org/cgi-bin/target_detail.cgi?targetID=3253550) | 1057 | 62 | hsa-miR-15a-5p | [GABRP](http://www.ncbi.nlm.nih.gov/entrez/query.fcgi?db=gene&cmd=Retrieve&dopt=full_report&list_uids=2568) | gamma-aminobutyric acid type A receptor pi subunit |
| [Details](https://mirdb.org/cgi-bin/target_detail.cgi?targetID=3253569) | 1058 | 62 | hsa-miR-15a-5p | [UBE2B](http://www.ncbi.nlm.nih.gov/entrez/query.fcgi?db=gene&cmd=Retrieve&dopt=full_report&list_uids=7320) | ubiquitin conjugating enzyme E2 B |
| [Details](https://mirdb.org/cgi-bin/target_detail.cgi?targetID=3253669) | 1059 | 62 | hsa-miR-15a-5p | [SH2D2A](http://www.ncbi.nlm.nih.gov/entrez/query.fcgi?db=gene&cmd=Retrieve&dopt=full_report&list_uids=9047) | SH2 domain containing 2A |
| [Details](https://mirdb.org/cgi-bin/target_detail.cgi?targetID=3253693) | 1060 | 62 | hsa-miR-15a-5p | [CDC14A](http://www.ncbi.nlm.nih.gov/entrez/query.fcgi?db=gene&cmd=Retrieve&dopt=full_report&list_uids=8556) | cell division cycle 14A |
| [Details](https://mirdb.org/cgi-bin/target_detail.cgi?targetID=3253735) | 1061 | 62 | hsa-miR-15a-5p | [THRB](http://www.ncbi.nlm.nih.gov/entrez/query.fcgi?db=gene&cmd=Retrieve&dopt=full_report&list_uids=7068) | thyroid hormone receptor beta |
| [Details](https://mirdb.org/cgi-bin/target_detail.cgi?targetID=3253861) | 1062 | 62 | hsa-miR-15a-5p | [GLUD1](http://www.ncbi.nlm.nih.gov/entrez/query.fcgi?db=gene&cmd=Retrieve&dopt=full_report&list_uids=2746) | glutamate dehydrogenase 1 |
| [Details](https://mirdb.org/cgi-bin/target_detail.cgi?targetID=3253885) | 1063 | 62 | hsa-miR-15a-5p | [IRF2BP2](http://www.ncbi.nlm.nih.gov/entrez/query.fcgi?db=gene&cmd=Retrieve&dopt=full_report&list_uids=359948) | interferon regulatory factor 2 binding protein 2 |
| [Details](https://mirdb.org/cgi-bin/target_detail.cgi?targetID=3253986) | 1064 | 62 | hsa-miR-15a-5p | [AP5B1](http://www.ncbi.nlm.nih.gov/entrez/query.fcgi?db=gene&cmd=Retrieve&dopt=full_report&list_uids=91056) | adaptor related protein complex 5 subunit beta 1 |
| [Details](https://mirdb.org/cgi-bin/target_detail.cgi?targetID=3254025) | 1065 | 62 | hsa-miR-15a-5p | [SYDE1](http://www.ncbi.nlm.nih.gov/entrez/query.fcgi?db=gene&cmd=Retrieve&dopt=full_report&list_uids=85360) | synapse defective Rho GTPase homolog 1 |
| [Details](https://mirdb.org/cgi-bin/target_detail.cgi?targetID=3252828) | 1066 | 61 | hsa-miR-15a-5p | [USP14](http://www.ncbi.nlm.nih.gov/entrez/query.fcgi?db=gene&cmd=Retrieve&dopt=full_report&list_uids=9097) | ubiquitin specific peptidase 14 |
| [Details](https://mirdb.org/cgi-bin/target_detail.cgi?targetID=3252892) | 1067 | 61 | hsa-miR-15a-5p | [PLEKHH1](http://www.ncbi.nlm.nih.gov/entrez/query.fcgi?db=gene&cmd=Retrieve&dopt=full_report&list_uids=57475) | pleckstrin homology, MyTH4 and FERM domain containing H1 |
| [Details](https://mirdb.org/cgi-bin/target_detail.cgi?targetID=3252924) | 1068 | 61 | hsa-miR-15a-5p | [FBXO33](http://www.ncbi.nlm.nih.gov/entrez/query.fcgi?db=gene&cmd=Retrieve&dopt=full_report&list_uids=254170) | F-box protein 33 |
| [Details](https://mirdb.org/cgi-bin/target_detail.cgi?targetID=3252962) | 1069 | 61 | hsa-miR-15a-5p | [SYNDIG1](http://www.ncbi.nlm.nih.gov/entrez/query.fcgi?db=gene&cmd=Retrieve&dopt=full_report&list_uids=79953) | synapse differentiation inducing 1 |
| [Details](https://mirdb.org/cgi-bin/target_detail.cgi?targetID=3253175) | 1070 | 61 | hsa-miR-15a-5p | [PTPRM](http://www.ncbi.nlm.nih.gov/entrez/query.fcgi?db=gene&cmd=Retrieve&dopt=full_report&list_uids=5797) | protein tyrosine phosphatase, receptor type M |
| [Details](https://mirdb.org/cgi-bin/target_detail.cgi?targetID=3253194) | 1071 | 61 | hsa-miR-15a-5p | [NECAP1](http://www.ncbi.nlm.nih.gov/entrez/query.fcgi?db=gene&cmd=Retrieve&dopt=full_report&list_uids=25977) | NECAP endocytosis associated 1 |
| [Details](https://mirdb.org/cgi-bin/target_detail.cgi?targetID=3253281) | 1072 | 61 | hsa-miR-15a-5p | [TBC1D7](http://www.ncbi.nlm.nih.gov/entrez/query.fcgi?db=gene&cmd=Retrieve&dopt=full_report&list_uids=51256) | TBC1 domain family member 7 |
| [Details](https://mirdb.org/cgi-bin/target_detail.cgi?targetID=3253299) | 1073 | 61 | hsa-miR-15a-5p | [DTNA](http://www.ncbi.nlm.nih.gov/entrez/query.fcgi?db=gene&cmd=Retrieve&dopt=full_report&list_uids=1837) | dystrobrevin alpha |
| [Details](https://mirdb.org/cgi-bin/target_detail.cgi?targetID=3253333) | 1074 | 61 | hsa-miR-15a-5p | [NACC2](http://www.ncbi.nlm.nih.gov/entrez/query.fcgi?db=gene&cmd=Retrieve&dopt=full_report&list_uids=138151) | NACC family member 2 |
| [Details](https://mirdb.org/cgi-bin/target_detail.cgi?targetID=3253405) | 1075 | 61 | hsa-miR-15a-5p | [ZNF362](http://www.ncbi.nlm.nih.gov/entrez/query.fcgi?db=gene&cmd=Retrieve&dopt=full_report&list_uids=149076) | zinc finger protein 362 |
| [Details](https://mirdb.org/cgi-bin/target_detail.cgi?targetID=3253444) | 1076 | 61 | hsa-miR-15a-5p | [EPHA1](http://www.ncbi.nlm.nih.gov/entrez/query.fcgi?db=gene&cmd=Retrieve&dopt=full_report&list_uids=2041) | EPH receptor A1 |
| [Details](https://mirdb.org/cgi-bin/target_detail.cgi?targetID=3253451) | 1077 | 61 | hsa-miR-15a-5p | [CCDC149](http://www.ncbi.nlm.nih.gov/entrez/query.fcgi?db=gene&cmd=Retrieve&dopt=full_report&list_uids=91050) | coiled-coil domain containing 149 |
| [Details](https://mirdb.org/cgi-bin/target_detail.cgi?targetID=3253528) | 1078 | 61 | hsa-miR-15a-5p | [LHFPL4](http://www.ncbi.nlm.nih.gov/entrez/query.fcgi?db=gene&cmd=Retrieve&dopt=full_report&list_uids=375323) | LHFPL tetraspan subfamily member 4 |
| [Details](https://mirdb.org/cgi-bin/target_detail.cgi?targetID=3253582) | 1079 | 61 | hsa-miR-15a-5p | [C15orf40](http://www.ncbi.nlm.nih.gov/entrez/query.fcgi?db=gene&cmd=Retrieve&dopt=full_report&list_uids=123207) | chromosome 15 open reading frame 40 |
| [Details](https://mirdb.org/cgi-bin/target_detail.cgi?targetID=3253682) | 1080 | 61 | hsa-miR-15a-5p | [PPIL4](http://www.ncbi.nlm.nih.gov/entrez/query.fcgi?db=gene&cmd=Retrieve&dopt=full_report&list_uids=85313) | peptidylprolyl isomerase like 4 |
| [Details](https://mirdb.org/cgi-bin/target_detail.cgi?targetID=3253724) | 1081 | 61 | hsa-miR-15a-5p | [RPS6KB1](http://www.ncbi.nlm.nih.gov/entrez/query.fcgi?db=gene&cmd=Retrieve&dopt=full_report&list_uids=6198) | ribosomal protein S6 kinase B1 |
| [Details](https://mirdb.org/cgi-bin/target_detail.cgi?targetID=3253731) | 1082 | 61 | hsa-miR-15a-5p | [PPM1H](http://www.ncbi.nlm.nih.gov/entrez/query.fcgi?db=gene&cmd=Retrieve&dopt=full_report&list_uids=57460) | protein phosphatase, Mg2+/Mn2+ dependent 1H |
| [Details](https://mirdb.org/cgi-bin/target_detail.cgi?targetID=3253761) | 1083 | 61 | hsa-miR-15a-5p | [GRPR](http://www.ncbi.nlm.nih.gov/entrez/query.fcgi?db=gene&cmd=Retrieve&dopt=full_report&list_uids=2925) | gastrin releasing peptide receptor |
| [Details](https://mirdb.org/cgi-bin/target_detail.cgi?targetID=3253777) | 1084 | 61 | hsa-miR-15a-5p | [MAP3K4](http://www.ncbi.nlm.nih.gov/entrez/query.fcgi?db=gene&cmd=Retrieve&dopt=full_report&list_uids=4216) | mitogen-activated protein kinase kinase kinase 4 |
| [Details](https://mirdb.org/cgi-bin/target_detail.cgi?targetID=3253812) | 1085 | 61 | hsa-miR-15a-5p | [VEGFD](http://www.ncbi.nlm.nih.gov/entrez/query.fcgi?db=gene&cmd=Retrieve&dopt=full_report&list_uids=2277) | vascular endothelial growth factor D |
| [Details](https://mirdb.org/cgi-bin/target_detail.cgi?targetID=3253891) | 1086 | 61 | hsa-miR-15a-5p | [ANKIB1](http://www.ncbi.nlm.nih.gov/entrez/query.fcgi?db=gene&cmd=Retrieve&dopt=full_report&list_uids=54467) | ankyrin repeat and IBR domain containing 1 |
| [Details](https://mirdb.org/cgi-bin/target_detail.cgi?targetID=3254006) | 1087 | 61 | hsa-miR-15a-5p | [TXNRD2](http://www.ncbi.nlm.nih.gov/entrez/query.fcgi?db=gene&cmd=Retrieve&dopt=full_report&list_uids=10587) | thioredoxin reductase 2 |
| [Details](https://mirdb.org/cgi-bin/target_detail.cgi?targetID=3252698) | 1088 | 60 | hsa-miR-15a-5p | [SNX29](http://www.ncbi.nlm.nih.gov/entrez/query.fcgi?db=gene&cmd=Retrieve&dopt=full_report&list_uids=92017) | sorting nexin 29 |
| [Details](https://mirdb.org/cgi-bin/target_detail.cgi?targetID=3252766) | 1089 | 60 | hsa-miR-15a-5p | [SLC4A7](http://www.ncbi.nlm.nih.gov/entrez/query.fcgi?db=gene&cmd=Retrieve&dopt=full_report&list_uids=9497) | solute carrier family 4 member 7 |
| [Details](https://mirdb.org/cgi-bin/target_detail.cgi?targetID=3252770) | 1090 | 60 | hsa-miR-15a-5p | [GRM1](http://www.ncbi.nlm.nih.gov/entrez/query.fcgi?db=gene&cmd=Retrieve&dopt=full_report&list_uids=2911) | glutamate metabotropic receptor 1 |
| [Details](https://mirdb.org/cgi-bin/target_detail.cgi?targetID=3252786) | 1091 | 60 | hsa-miR-15a-5p | [BVES](http://www.ncbi.nlm.nih.gov/entrez/query.fcgi?db=gene&cmd=Retrieve&dopt=full_report&list_uids=11149) | blood vessel epicardial substance |
| [Details](https://mirdb.org/cgi-bin/target_detail.cgi?targetID=3252788) | 1092 | 60 | hsa-miR-15a-5p | [CSMD1](http://www.ncbi.nlm.nih.gov/entrez/query.fcgi?db=gene&cmd=Retrieve&dopt=full_report&list_uids=64478) | CUB and Sushi multiple domains 1 |
| [Details](https://mirdb.org/cgi-bin/target_detail.cgi?targetID=3252844) | 1093 | 60 | hsa-miR-15a-5p | [ESYT3](http://www.ncbi.nlm.nih.gov/entrez/query.fcgi?db=gene&cmd=Retrieve&dopt=full_report&list_uids=83850) | extended synaptotagmin 3 |
| [Details](https://mirdb.org/cgi-bin/target_detail.cgi?targetID=3252905) | 1094 | 60 | hsa-miR-15a-5p | [SIDT1](http://www.ncbi.nlm.nih.gov/entrez/query.fcgi?db=gene&cmd=Retrieve&dopt=full_report&list_uids=54847) | SID1 transmembrane family member 1 |
| [Details](https://mirdb.org/cgi-bin/target_detail.cgi?targetID=3253037) | 1095 | 60 | hsa-miR-15a-5p | [ISOC1](http://www.ncbi.nlm.nih.gov/entrez/query.fcgi?db=gene&cmd=Retrieve&dopt=full_report&list_uids=51015) | isochorismatase domain containing 1 |
| [Details](https://mirdb.org/cgi-bin/target_detail.cgi?targetID=3253039) | 1096 | 60 | hsa-miR-15a-5p | [SAMD4A](http://www.ncbi.nlm.nih.gov/entrez/query.fcgi?db=gene&cmd=Retrieve&dopt=full_report&list_uids=23034) | sterile alpha motif domain containing 4A |
| [Details](https://mirdb.org/cgi-bin/target_detail.cgi?targetID=3253183) | 1097 | 60 | hsa-miR-15a-5p | [C11orf24](http://www.ncbi.nlm.nih.gov/entrez/query.fcgi?db=gene&cmd=Retrieve&dopt=full_report&list_uids=53838) | chromosome 11 open reading frame 24 |
| [Details](https://mirdb.org/cgi-bin/target_detail.cgi?targetID=3253195) | 1098 | 60 | hsa-miR-15a-5p | [ASB7](http://www.ncbi.nlm.nih.gov/entrez/query.fcgi?db=gene&cmd=Retrieve&dopt=full_report&list_uids=140460) | ankyrin repeat and SOCS box containing 7 |
| [Details](https://mirdb.org/cgi-bin/target_detail.cgi?targetID=3253231) | 1099 | 60 | hsa-miR-15a-5p | [SARM1](http://www.ncbi.nlm.nih.gov/entrez/query.fcgi?db=gene&cmd=Retrieve&dopt=full_report&list_uids=23098) | sterile alpha and TIR motif containing 1 |
| [Details](https://mirdb.org/cgi-bin/target_detail.cgi?targetID=3253252) | 1100 | 60 | hsa-miR-15a-5p | [DEPTOR](http://www.ncbi.nlm.nih.gov/entrez/query.fcgi?db=gene&cmd=Retrieve&dopt=full_report&list_uids=64798) | DEP domain containing MTOR interacting protein |
| [Details](https://mirdb.org/cgi-bin/target_detail.cgi?targetID=3253269) | 1101 | 60 | hsa-miR-15a-5p | [SLC16A6](http://www.ncbi.nlm.nih.gov/entrez/query.fcgi?db=gene&cmd=Retrieve&dopt=full_report&list_uids=9120) | solute carrier family 16 member 6 |
| [Details](https://mirdb.org/cgi-bin/target_detail.cgi?targetID=3253300) | 1102 | 60 | hsa-miR-15a-5p | [KCNMA1](http://www.ncbi.nlm.nih.gov/entrez/query.fcgi?db=gene&cmd=Retrieve&dopt=full_report&list_uids=3778) | potassium calcium-activated channel subfamily M alpha 1 |
| [Details](https://mirdb.org/cgi-bin/target_detail.cgi?targetID=3253367) | 1103 | 60 | hsa-miR-15a-5p | [NRARP](http://www.ncbi.nlm.nih.gov/entrez/query.fcgi?db=gene&cmd=Retrieve&dopt=full_report&list_uids=441478) | NOTCH regulated ankyrin repeat protein |
| [Details](https://mirdb.org/cgi-bin/target_detail.cgi?targetID=3253437) | 1104 | 60 | hsa-miR-15a-5p | [DSEL](http://www.ncbi.nlm.nih.gov/entrez/query.fcgi?db=gene&cmd=Retrieve&dopt=full_report&list_uids=92126) | dermatan sulfate epimerase like |
| [Details](https://mirdb.org/cgi-bin/target_detail.cgi?targetID=3253492) | 1105 | 60 | hsa-miR-15a-5p | [CADM1](http://www.ncbi.nlm.nih.gov/entrez/query.fcgi?db=gene&cmd=Retrieve&dopt=full_report&list_uids=23705) | cell adhesion molecule 1 |
| [Details](https://mirdb.org/cgi-bin/target_detail.cgi?targetID=3253647) | 1106 | 60 | hsa-miR-15a-5p | [CACNB4](http://www.ncbi.nlm.nih.gov/entrez/query.fcgi?db=gene&cmd=Retrieve&dopt=full_report&list_uids=785) | calcium voltage-gated channel auxiliary subunit beta 4 |
| [Details](https://mirdb.org/cgi-bin/target_detail.cgi?targetID=3253791) | 1107 | 60 | hsa-miR-15a-5p | [DCN](http://www.ncbi.nlm.nih.gov/entrez/query.fcgi?db=gene&cmd=Retrieve&dopt=full_report&list_uids=1634) | decorin |
| [Details](https://mirdb.org/cgi-bin/target_detail.cgi?targetID=3253850) | 1108 | 60 | hsa-miR-15a-5p | [VCL](http://www.ncbi.nlm.nih.gov/entrez/query.fcgi?db=gene&cmd=Retrieve&dopt=full_report&list_uids=7414) | vinculin |
| [Details](https://mirdb.org/cgi-bin/target_detail.cgi?targetID=3253857) | 1109 | 60 | hsa-miR-15a-5p | [RBMS2](http://www.ncbi.nlm.nih.gov/entrez/query.fcgi?db=gene&cmd=Retrieve&dopt=full_report&list_uids=5939) | RNA binding motif single stranded interacting protein 2 |
| [Details](https://mirdb.org/cgi-bin/target_detail.cgi?targetID=3253922) | 1110 | 60 | hsa-miR-15a-5p | [MEAK7](http://www.ncbi.nlm.nih.gov/entrez/query.fcgi?db=gene&cmd=Retrieve&dopt=full_report&list_uids=57707) | MTOR associated protein, eak-7 homolog |
| [Details](https://mirdb.org/cgi-bin/target_detail.cgi?targetID=3253935) | 1111 | 60 | hsa-miR-15a-5p | [ACTR1A](http://www.ncbi.nlm.nih.gov/entrez/query.fcgi?db=gene&cmd=Retrieve&dopt=full_report&list_uids=10121) | ARP1 actin related protein 1 homolog A |
| [Details](https://mirdb.org/cgi-bin/target_detail.cgi?targetID=3254004) | 1112 | 60 | hsa-miR-15a-5p | [DDI1](http://www.ncbi.nlm.nih.gov/entrez/query.fcgi?db=gene&cmd=Retrieve&dopt=full_report&list_uids=414301) | DNA damage inducible 1 homolog 1 |
| [Details](https://mirdb.org/cgi-bin/target_detail.cgi?targetID=3252648) | 1113 | 59 | hsa-miR-15a-5p | [TRIM37](http://www.ncbi.nlm.nih.gov/entrez/query.fcgi?db=gene&cmd=Retrieve&dopt=full_report&list_uids=4591) | tripartite motif containing 37 |
| [Details](https://mirdb.org/cgi-bin/target_detail.cgi?targetID=3252879) | 1114 | 59 | hsa-miR-15a-5p | [CXorf40A](http://www.ncbi.nlm.nih.gov/entrez/query.fcgi?db=gene&cmd=Retrieve&dopt=full_report&list_uids=91966) | chromosome X open reading frame 40A |
| [Details](https://mirdb.org/cgi-bin/target_detail.cgi?targetID=3252910) | 1115 | 59 | hsa-miR-15a-5p | [RNF111](http://www.ncbi.nlm.nih.gov/entrez/query.fcgi?db=gene&cmd=Retrieve&dopt=full_report&list_uids=54778) | ring finger protein 111 |
| [Details](https://mirdb.org/cgi-bin/target_detail.cgi?targetID=3253050) | 1116 | 59 | hsa-miR-15a-5p | [HS3ST5](http://www.ncbi.nlm.nih.gov/entrez/query.fcgi?db=gene&cmd=Retrieve&dopt=full_report&list_uids=222537) | heparan sulfate-glucosamine 3-sulfotransferase 5 |
| [Details](https://mirdb.org/cgi-bin/target_detail.cgi?targetID=3253239) | 1117 | 59 | hsa-miR-15a-5p | [SCN2A](http://www.ncbi.nlm.nih.gov/entrez/query.fcgi?db=gene&cmd=Retrieve&dopt=full_report&list_uids=6326) | sodium voltage-gated channel alpha subunit 2 |
| [Details](https://mirdb.org/cgi-bin/target_detail.cgi?targetID=3253410) | 1118 | 59 | hsa-miR-15a-5p | [ZBTB2](http://www.ncbi.nlm.nih.gov/entrez/query.fcgi?db=gene&cmd=Retrieve&dopt=full_report&list_uids=57621) | zinc finger and BTB domain containing 2 |
| [Details](https://mirdb.org/cgi-bin/target_detail.cgi?targetID=3253463) | 1119 | 59 | hsa-miR-15a-5p | [ZC3H11A](http://www.ncbi.nlm.nih.gov/entrez/query.fcgi?db=gene&cmd=Retrieve&dopt=full_report&list_uids=9877) | zinc finger CCCH-type containing 11A |
| [Details](https://mirdb.org/cgi-bin/target_detail.cgi?targetID=3253496) | 1120 | 59 | hsa-miR-15a-5p | [XPR1](http://www.ncbi.nlm.nih.gov/entrez/query.fcgi?db=gene&cmd=Retrieve&dopt=full_report&list_uids=9213) | xenotropic and polytropic retrovirus receptor 1 |
| [Details](https://mirdb.org/cgi-bin/target_detail.cgi?targetID=3253523) | 1121 | 59 | hsa-miR-15a-5p | [MTAP](http://www.ncbi.nlm.nih.gov/entrez/query.fcgi?db=gene&cmd=Retrieve&dopt=full_report&list_uids=4507) | methylthioadenosine phosphorylase |
| [Details](https://mirdb.org/cgi-bin/target_detail.cgi?targetID=3253524) | 1122 | 59 | hsa-miR-15a-5p | [BACE2](http://www.ncbi.nlm.nih.gov/entrez/query.fcgi?db=gene&cmd=Retrieve&dopt=full_report&list_uids=25825) | beta-secretase 2 |
| [Details](https://mirdb.org/cgi-bin/target_detail.cgi?targetID=3253588) | 1123 | 59 | hsa-miR-15a-5p | [RAB40A](http://www.ncbi.nlm.nih.gov/entrez/query.fcgi?db=gene&cmd=Retrieve&dopt=full_report&list_uids=142684) | RAB40A, member RAS oncogene family |
| [Details](https://mirdb.org/cgi-bin/target_detail.cgi?targetID=3253591) | 1124 | 59 | hsa-miR-15a-5p | [MAFK](http://www.ncbi.nlm.nih.gov/entrez/query.fcgi?db=gene&cmd=Retrieve&dopt=full_report&list_uids=7975) | MAF bZIP transcription factor K |
| [Details](https://mirdb.org/cgi-bin/target_detail.cgi?targetID=3253599) | 1125 | 59 | hsa-miR-15a-5p | [CWC15](http://www.ncbi.nlm.nih.gov/entrez/query.fcgi?db=gene&cmd=Retrieve&dopt=full_report&list_uids=51503) | CWC15 spliceosome associated protein homolog |
| [Details](https://mirdb.org/cgi-bin/target_detail.cgi?targetID=3253683) | 1126 | 59 | hsa-miR-15a-5p | [BACH2](http://www.ncbi.nlm.nih.gov/entrez/query.fcgi?db=gene&cmd=Retrieve&dopt=full_report&list_uids=60468) | BTB domain and CNC homolog 2 |
| [Details](https://mirdb.org/cgi-bin/target_detail.cgi?targetID=3253686) | 1127 | 59 | hsa-miR-15a-5p | [TNIK](http://www.ncbi.nlm.nih.gov/entrez/query.fcgi?db=gene&cmd=Retrieve&dopt=full_report&list_uids=23043) | TRAF2 and NCK interacting kinase |
| [Details](https://mirdb.org/cgi-bin/target_detail.cgi?targetID=3253703) | 1128 | 59 | hsa-miR-15a-5p | [SSU72](http://www.ncbi.nlm.nih.gov/entrez/query.fcgi?db=gene&cmd=Retrieve&dopt=full_report&list_uids=29101) | SSU72 homolog, RNA polymerase II CTD phosphatase |
| [Details](https://mirdb.org/cgi-bin/target_detail.cgi?targetID=3253728) | 1129 | 59 | hsa-miR-15a-5p | [SGPL1](http://www.ncbi.nlm.nih.gov/entrez/query.fcgi?db=gene&cmd=Retrieve&dopt=full_report&list_uids=8879) | sphingosine-1-phosphate lyase 1 |
| [Details](https://mirdb.org/cgi-bin/target_detail.cgi?targetID=3253758) | 1130 | 59 | hsa-miR-15a-5p | [VSTM2A](http://www.ncbi.nlm.nih.gov/entrez/query.fcgi?db=gene&cmd=Retrieve&dopt=full_report&list_uids=222008) | V-set and transmembrane domain containing 2A |
| [Details](https://mirdb.org/cgi-bin/target_detail.cgi?targetID=3253760) | 1131 | 59 | hsa-miR-15a-5p | [TLE4](http://www.ncbi.nlm.nih.gov/entrez/query.fcgi?db=gene&cmd=Retrieve&dopt=full_report&list_uids=7091) | TLE family member 4, transcriptional corepressor |
| [Details](https://mirdb.org/cgi-bin/target_detail.cgi?targetID=3253783) | 1132 | 59 | hsa-miR-15a-5p | [FDFT1](http://www.ncbi.nlm.nih.gov/entrez/query.fcgi?db=gene&cmd=Retrieve&dopt=full_report&list_uids=2222) | farnesyl-diphosphate farnesyltransferase 1 |
| [Details](https://mirdb.org/cgi-bin/target_detail.cgi?targetID=3253793) | 1133 | 59 | hsa-miR-15a-5p | [RANBP3](http://www.ncbi.nlm.nih.gov/entrez/query.fcgi?db=gene&cmd=Retrieve&dopt=full_report&list_uids=8498) | RAN binding protein 3 |
| [Details](https://mirdb.org/cgi-bin/target_detail.cgi?targetID=3253864) | 1134 | 59 | hsa-miR-15a-5p | [EYA4](http://www.ncbi.nlm.nih.gov/entrez/query.fcgi?db=gene&cmd=Retrieve&dopt=full_report&list_uids=2070) | EYA transcriptional coactivator and phosphatase 4 |
| [Details](https://mirdb.org/cgi-bin/target_detail.cgi?targetID=3253867) | 1135 | 59 | hsa-miR-15a-5p | [VAMP1](http://www.ncbi.nlm.nih.gov/entrez/query.fcgi?db=gene&cmd=Retrieve&dopt=full_report&list_uids=6843) | vesicle associated membrane protein 1 |
| [Details](https://mirdb.org/cgi-bin/target_detail.cgi?targetID=3253871) | 1136 | 59 | hsa-miR-15a-5p | [COMT](http://www.ncbi.nlm.nih.gov/entrez/query.fcgi?db=gene&cmd=Retrieve&dopt=full_report&list_uids=1312) | catechol-O-methyltransferase |
| [Details](https://mirdb.org/cgi-bin/target_detail.cgi?targetID=3253912) | 1137 | 59 | hsa-miR-15a-5p | [STX1A](http://www.ncbi.nlm.nih.gov/entrez/query.fcgi?db=gene&cmd=Retrieve&dopt=full_report&list_uids=6804) | syntaxin 1A |
| [Details](https://mirdb.org/cgi-bin/target_detail.cgi?targetID=3253941) | 1138 | 59 | hsa-miR-15a-5p | [TTC39A](http://www.ncbi.nlm.nih.gov/entrez/query.fcgi?db=gene&cmd=Retrieve&dopt=full_report&list_uids=22996) | tetratricopeptide repeat domain 39A |
| [Details](https://mirdb.org/cgi-bin/target_detail.cgi?targetID=3253948) | 1139 | 59 | hsa-miR-15a-5p | [MICAL2](http://www.ncbi.nlm.nih.gov/entrez/query.fcgi?db=gene&cmd=Retrieve&dopt=full_report&list_uids=9645) | microtubule associated monooxygenase, calponin and LIM domain containing 2 |
| [Details](https://mirdb.org/cgi-bin/target_detail.cgi?targetID=3254018) | 1140 | 59 | hsa-miR-15a-5p | [LHPP](http://www.ncbi.nlm.nih.gov/entrez/query.fcgi?db=gene&cmd=Retrieve&dopt=full_report&list_uids=64077) | phospholysine phosphohistidine inorganic pyrophosphate phosphatase |
| [Details](https://mirdb.org/cgi-bin/target_detail.cgi?targetID=3254020) | 1141 | 59 | hsa-miR-15a-5p | [PITPNA](http://www.ncbi.nlm.nih.gov/entrez/query.fcgi?db=gene&cmd=Retrieve&dopt=full_report&list_uids=5306) | phosphatidylinositol transfer protein alpha |
| [Details](https://mirdb.org/cgi-bin/target_detail.cgi?targetID=3254023) | 1142 | 59 | hsa-miR-15a-5p | [PLEKHB2](http://www.ncbi.nlm.nih.gov/entrez/query.fcgi?db=gene&cmd=Retrieve&dopt=full_report&list_uids=55041) | pleckstrin homology domain containing B2 |
| [Details](https://mirdb.org/cgi-bin/target_detail.cgi?targetID=3252701) | 1143 | 58 | hsa-miR-15a-5p | [PKDCC](http://www.ncbi.nlm.nih.gov/entrez/query.fcgi?db=gene&cmd=Retrieve&dopt=full_report&list_uids=91461) | protein kinase domain containing, cytoplasmic |
| [Details](https://mirdb.org/cgi-bin/target_detail.cgi?targetID=3252720) | 1144 | 58 | hsa-miR-15a-5p | [PDAP1](http://www.ncbi.nlm.nih.gov/entrez/query.fcgi?db=gene&cmd=Retrieve&dopt=full_report&list_uids=11333) | PDGFA associated protein 1 |
| [Details](https://mirdb.org/cgi-bin/target_detail.cgi?targetID=3252781) | 1145 | 58 | hsa-miR-15a-5p | [CDC42](http://www.ncbi.nlm.nih.gov/entrez/query.fcgi?db=gene&cmd=Retrieve&dopt=full_report&list_uids=998) | cell division cycle 42 |
| [Details](https://mirdb.org/cgi-bin/target_detail.cgi?targetID=3252802) | 1146 | 58 | hsa-miR-15a-5p | [RELT](http://www.ncbi.nlm.nih.gov/entrez/query.fcgi?db=gene&cmd=Retrieve&dopt=full_report&list_uids=84957) | RELT, TNF receptor |
| [Details](https://mirdb.org/cgi-bin/target_detail.cgi?targetID=3252927) | 1147 | 58 | hsa-miR-15a-5p | [PIP4K2C](http://www.ncbi.nlm.nih.gov/entrez/query.fcgi?db=gene&cmd=Retrieve&dopt=full_report&list_uids=79837) | phosphatidylinositol-5-phosphate 4-kinase type 2 gamma |
| [Details](https://mirdb.org/cgi-bin/target_detail.cgi?targetID=3252946) | 1148 | 58 | hsa-miR-15a-5p | [ELP1](http://www.ncbi.nlm.nih.gov/entrez/query.fcgi?db=gene&cmd=Retrieve&dopt=full_report&list_uids=8518) | elongator complex protein 1 |
| [Details](https://mirdb.org/cgi-bin/target_detail.cgi?targetID=3252979) | 1149 | 58 | hsa-miR-15a-5p | [AATK](http://www.ncbi.nlm.nih.gov/entrez/query.fcgi?db=gene&cmd=Retrieve&dopt=full_report&list_uids=9625) | apoptosis associated tyrosine kinase |
| [Details](https://mirdb.org/cgi-bin/target_detail.cgi?targetID=3252989) | 1150 | 58 | hsa-miR-15a-5p | [SLC9B2](http://www.ncbi.nlm.nih.gov/entrez/query.fcgi?db=gene&cmd=Retrieve&dopt=full_report&list_uids=133308) | solute carrier family 9 member B2 |
| [Details](https://mirdb.org/cgi-bin/target_detail.cgi?targetID=3253001) | 1151 | 58 | hsa-miR-15a-5p | [RAB3B](http://www.ncbi.nlm.nih.gov/entrez/query.fcgi?db=gene&cmd=Retrieve&dopt=full_report&list_uids=5865) | RAB3B, member RAS oncogene family |
| [Details](https://mirdb.org/cgi-bin/target_detail.cgi?targetID=3253040) | 1152 | 58 | hsa-miR-15a-5p | [TTPAL](http://www.ncbi.nlm.nih.gov/entrez/query.fcgi?db=gene&cmd=Retrieve&dopt=full_report&list_uids=79183) | alpha tocopherol transfer protein like |
| [Details](https://mirdb.org/cgi-bin/target_detail.cgi?targetID=3253086) | 1153 | 58 | hsa-miR-15a-5p | [NMD3](http://www.ncbi.nlm.nih.gov/entrez/query.fcgi?db=gene&cmd=Retrieve&dopt=full_report&list_uids=51068) | NMD3 ribosome export adaptor |
| [Details](https://mirdb.org/cgi-bin/target_detail.cgi?targetID=3253182) | 1154 | 58 | hsa-miR-15a-5p | [MYO1C](http://www.ncbi.nlm.nih.gov/entrez/query.fcgi?db=gene&cmd=Retrieve&dopt=full_report&list_uids=4641) | myosin IC |
| [Details](https://mirdb.org/cgi-bin/target_detail.cgi?targetID=3253368) | 1155 | 58 | hsa-miR-15a-5p | [THUMPD1](http://www.ncbi.nlm.nih.gov/entrez/query.fcgi?db=gene&cmd=Retrieve&dopt=full_report&list_uids=55623) | THUMP domain containing 1 |
| [Details](https://mirdb.org/cgi-bin/target_detail.cgi?targetID=3253414) | 1156 | 58 | hsa-miR-15a-5p | [CXCR3](http://www.ncbi.nlm.nih.gov/entrez/query.fcgi?db=gene&cmd=Retrieve&dopt=full_report&list_uids=2833) | C-X-C motif chemokine receptor 3 |
| [Details](https://mirdb.org/cgi-bin/target_detail.cgi?targetID=3253425) | 1157 | 58 | hsa-miR-15a-5p | [CNIH2](http://www.ncbi.nlm.nih.gov/entrez/query.fcgi?db=gene&cmd=Retrieve&dopt=full_report&list_uids=254263) | cornichon family AMPA receptor auxiliary protein 2 |
| [Details](https://mirdb.org/cgi-bin/target_detail.cgi?targetID=3253427) | 1158 | 58 | hsa-miR-15a-5p | [PCDHA9](http://www.ncbi.nlm.nih.gov/entrez/query.fcgi?db=gene&cmd=Retrieve&dopt=full_report&list_uids=9752) | protocadherin alpha 9 |
| [Details](https://mirdb.org/cgi-bin/target_detail.cgi?targetID=3253535) | 1159 | 58 | hsa-miR-15a-5p | [PLPP6](http://www.ncbi.nlm.nih.gov/entrez/query.fcgi?db=gene&cmd=Retrieve&dopt=full_report&list_uids=403313) | phospholipid phosphatase 6 |
| [Details](https://mirdb.org/cgi-bin/target_detail.cgi?targetID=3253566) | 1160 | 58 | hsa-miR-15a-5p | [PAFAH2](http://www.ncbi.nlm.nih.gov/entrez/query.fcgi?db=gene&cmd=Retrieve&dopt=full_report&list_uids=5051) | platelet activating factor acetylhydrolase 2 |
| [Details](https://mirdb.org/cgi-bin/target_detail.cgi?targetID=3253613) | 1161 | 58 | hsa-miR-15a-5p | [EFNB2](http://www.ncbi.nlm.nih.gov/entrez/query.fcgi?db=gene&cmd=Retrieve&dopt=full_report&list_uids=1948) | ephrin B2 |
| [Details](https://mirdb.org/cgi-bin/target_detail.cgi?targetID=3253628) | 1162 | 58 | hsa-miR-15a-5p | [BPIFA1](http://www.ncbi.nlm.nih.gov/entrez/query.fcgi?db=gene&cmd=Retrieve&dopt=full_report&list_uids=51297) | BPI fold containing family A member 1 |
| [Details](https://mirdb.org/cgi-bin/target_detail.cgi?targetID=3253651) | 1163 | 58 | hsa-miR-15a-5p | [LHFPL5](http://www.ncbi.nlm.nih.gov/entrez/query.fcgi?db=gene&cmd=Retrieve&dopt=full_report&list_uids=222662) | LHFPL tetraspan subfamily member 5 |
| [Details](https://mirdb.org/cgi-bin/target_detail.cgi?targetID=3253701) | 1164 | 58 | hsa-miR-15a-5p | [RNMT](http://www.ncbi.nlm.nih.gov/entrez/query.fcgi?db=gene&cmd=Retrieve&dopt=full_report&list_uids=8731) | RNA guanine-7 methyltransferase |
| [Details](https://mirdb.org/cgi-bin/target_detail.cgi?targetID=3253756) | 1165 | 58 | hsa-miR-15a-5p | [M6PR](http://www.ncbi.nlm.nih.gov/entrez/query.fcgi?db=gene&cmd=Retrieve&dopt=full_report&list_uids=4074) | mannose-6-phosphate receptor, cation dependent |
| [Details](https://mirdb.org/cgi-bin/target_detail.cgi?targetID=3253790) | 1166 | 58 | hsa-miR-15a-5p | [SVEP1](http://www.ncbi.nlm.nih.gov/entrez/query.fcgi?db=gene&cmd=Retrieve&dopt=full_report&list_uids=79987) | sushi, von Willebrand factor type A, EGF and pentraxin domain containing 1 |
| [Details](https://mirdb.org/cgi-bin/target_detail.cgi?targetID=3253825) | 1167 | 58 | hsa-miR-15a-5p | [C6orf222](http://www.ncbi.nlm.nih.gov/entrez/query.fcgi?db=gene&cmd=Retrieve&dopt=full_report&list_uids=389384) | chromosome 6 open reading frame 222 |
| [Details](https://mirdb.org/cgi-bin/target_detail.cgi?targetID=3253929) | 1168 | 58 | hsa-miR-15a-5p | [NIM1K](http://www.ncbi.nlm.nih.gov/entrez/query.fcgi?db=gene&cmd=Retrieve&dopt=full_report&list_uids=167359) | NIM1 serine/threonine protein kinase |
| [Details](https://mirdb.org/cgi-bin/target_detail.cgi?targetID=3253944) | 1169 | 58 | hsa-miR-15a-5p | [MACC1](http://www.ncbi.nlm.nih.gov/entrez/query.fcgi?db=gene&cmd=Retrieve&dopt=full_report&list_uids=346389) | MET transcriptional regulator MACC1 |
| [Details](https://mirdb.org/cgi-bin/target_detail.cgi?targetID=3253954) | 1170 | 58 | hsa-miR-15a-5p | [GHSR](http://www.ncbi.nlm.nih.gov/entrez/query.fcgi?db=gene&cmd=Retrieve&dopt=full_report&list_uids=2693) | growth hormone secretagogue receptor |
| [Details](https://mirdb.org/cgi-bin/target_detail.cgi?targetID=3253966) | 1171 | 58 | hsa-miR-15a-5p | [IGSF23](http://www.ncbi.nlm.nih.gov/entrez/query.fcgi?db=gene&cmd=Retrieve&dopt=full_report&list_uids=147710) | immunoglobulin superfamily member 23 |
| [Details](https://mirdb.org/cgi-bin/target_detail.cgi?targetID=3253982) | 1172 | 58 | hsa-miR-15a-5p | [CAPN6](http://www.ncbi.nlm.nih.gov/entrez/query.fcgi?db=gene&cmd=Retrieve&dopt=full_report&list_uids=827) | calpain 6 |
| [Details](https://mirdb.org/cgi-bin/target_detail.cgi?targetID=3253985) | 1173 | 58 | hsa-miR-15a-5p | [KCTD1](http://www.ncbi.nlm.nih.gov/entrez/query.fcgi?db=gene&cmd=Retrieve&dopt=full_report&list_uids=284252) | potassium channel tetramerization domain containing 1 |
| [Details](https://mirdb.org/cgi-bin/target_detail.cgi?targetID=3254029) | 1174 | 58 | hsa-miR-15a-5p | [TAF5](http://www.ncbi.nlm.nih.gov/entrez/query.fcgi?db=gene&cmd=Retrieve&dopt=full_report&list_uids=6877) | TATA-box binding protein associated factor 5 |
| [Details](https://mirdb.org/cgi-bin/target_detail.cgi?targetID=3254041) | 1175 | 58 | hsa-miR-15a-5p | [MYADM](http://www.ncbi.nlm.nih.gov/entrez/query.fcgi?db=gene&cmd=Retrieve&dopt=full_report&list_uids=91663) | myeloid associated differentiation marker |
| [Details](https://mirdb.org/cgi-bin/target_detail.cgi?targetID=3252636) | 1176 | 57 | hsa-miR-15a-5p | [LCP1](http://www.ncbi.nlm.nih.gov/entrez/query.fcgi?db=gene&cmd=Retrieve&dopt=full_report&list_uids=3936) | lymphocyte cytosolic protein 1 |
| [Details](https://mirdb.org/cgi-bin/target_detail.cgi?targetID=3252702) | 1177 | 57 | hsa-miR-15a-5p | [CYB5B](http://www.ncbi.nlm.nih.gov/entrez/query.fcgi?db=gene&cmd=Retrieve&dopt=full_report&list_uids=80777) | cytochrome b5 type B |
| [Details](https://mirdb.org/cgi-bin/target_detail.cgi?targetID=3252718) | 1178 | 57 | hsa-miR-15a-5p | [ANOS1](http://www.ncbi.nlm.nih.gov/entrez/query.fcgi?db=gene&cmd=Retrieve&dopt=full_report&list_uids=3730) | anosmin 1 |
| [Details](https://mirdb.org/cgi-bin/target_detail.cgi?targetID=3252724) | 1179 | 57 | hsa-miR-15a-5p | [ZDHHC21](http://www.ncbi.nlm.nih.gov/entrez/query.fcgi?db=gene&cmd=Retrieve&dopt=full_report&list_uids=340481) | zinc finger DHHC-type containing 21 |
| [Details](https://mirdb.org/cgi-bin/target_detail.cgi?targetID=3252777) | 1180 | 57 | hsa-miR-15a-5p | [UBL3](http://www.ncbi.nlm.nih.gov/entrez/query.fcgi?db=gene&cmd=Retrieve&dopt=full_report&list_uids=5412) | ubiquitin like 3 |
| [Details](https://mirdb.org/cgi-bin/target_detail.cgi?targetID=3252909) | 1181 | 57 | hsa-miR-15a-5p | [PIAS1](http://www.ncbi.nlm.nih.gov/entrez/query.fcgi?db=gene&cmd=Retrieve&dopt=full_report&list_uids=8554) | protein inhibitor of activated STAT 1 |
| [Details](https://mirdb.org/cgi-bin/target_detail.cgi?targetID=3252912) | 1182 | 57 | hsa-miR-15a-5p | [TMEM68](http://www.ncbi.nlm.nih.gov/entrez/query.fcgi?db=gene&cmd=Retrieve&dopt=full_report&list_uids=137695) | transmembrane protein 68 |
| [Details](https://mirdb.org/cgi-bin/target_detail.cgi?targetID=3252930) | 1183 | 57 | hsa-miR-15a-5p | [GRAMD2B](http://www.ncbi.nlm.nih.gov/entrez/query.fcgi?db=gene&cmd=Retrieve&dopt=full_report&list_uids=65983) | GRAM domain containing 2B |
| [Details](https://mirdb.org/cgi-bin/target_detail.cgi?targetID=3252939) | 1184 | 57 | hsa-miR-15a-5p | [TBC1D16](http://www.ncbi.nlm.nih.gov/entrez/query.fcgi?db=gene&cmd=Retrieve&dopt=full_report&list_uids=125058) | TBC1 domain family member 16 |
| [Details](https://mirdb.org/cgi-bin/target_detail.cgi?targetID=3252982) | 1185 | 57 | hsa-miR-15a-5p | [PIK3C2A](http://www.ncbi.nlm.nih.gov/entrez/query.fcgi?db=gene&cmd=Retrieve&dopt=full_report&list_uids=5286) | phosphatidylinositol-4-phosphate 3-kinase catalytic subunit type 2 alpha |
| [Details](https://mirdb.org/cgi-bin/target_detail.cgi?targetID=3253004) | 1186 | 57 | hsa-miR-15a-5p | [ZNF662](http://www.ncbi.nlm.nih.gov/entrez/query.fcgi?db=gene&cmd=Retrieve&dopt=full_report&list_uids=389114) | zinc finger protein 662 |
| [Details](https://mirdb.org/cgi-bin/target_detail.cgi?targetID=3253012) | 1187 | 57 | hsa-miR-15a-5p | [PDCD4](http://www.ncbi.nlm.nih.gov/entrez/query.fcgi?db=gene&cmd=Retrieve&dopt=full_report&list_uids=27250) | programmed cell death 4 |
| [Details](https://mirdb.org/cgi-bin/target_detail.cgi?targetID=3253015) | 1188 | 57 | hsa-miR-15a-5p | [USP38](http://www.ncbi.nlm.nih.gov/entrez/query.fcgi?db=gene&cmd=Retrieve&dopt=full_report&list_uids=84640) | ubiquitin specific peptidase 38 |
| [Details](https://mirdb.org/cgi-bin/target_detail.cgi?targetID=3253020) | 1189 | 57 | hsa-miR-15a-5p | [DEDD](http://www.ncbi.nlm.nih.gov/entrez/query.fcgi?db=gene&cmd=Retrieve&dopt=full_report&list_uids=9191) | death effector domain containing |
| [Details](https://mirdb.org/cgi-bin/target_detail.cgi?targetID=3253117) | 1190 | 57 | hsa-miR-15a-5p | [MAMLD1](http://www.ncbi.nlm.nih.gov/entrez/query.fcgi?db=gene&cmd=Retrieve&dopt=full_report&list_uids=10046) | mastermind like domain containing 1 |
| [Details](https://mirdb.org/cgi-bin/target_detail.cgi?targetID=3253135) | 1191 | 57 | hsa-miR-15a-5p | [CAB39](http://www.ncbi.nlm.nih.gov/entrez/query.fcgi?db=gene&cmd=Retrieve&dopt=full_report&list_uids=51719) | calcium binding protein 39 |
| [Details](https://mirdb.org/cgi-bin/target_detail.cgi?targetID=3253163) | 1192 | 57 | hsa-miR-15a-5p | [BCL9L](http://www.ncbi.nlm.nih.gov/entrez/query.fcgi?db=gene&cmd=Retrieve&dopt=full_report&list_uids=283149) | BCL9 like |
| [Details](https://mirdb.org/cgi-bin/target_detail.cgi?targetID=3253200) | 1193 | 57 | hsa-miR-15a-5p | [ZNF697](http://www.ncbi.nlm.nih.gov/entrez/query.fcgi?db=gene&cmd=Retrieve&dopt=full_report&list_uids=90874) | zinc finger protein 697 |
| [Details](https://mirdb.org/cgi-bin/target_detail.cgi?targetID=3253212) | 1194 | 57 | hsa-miR-15a-5p | [GLT1D1](http://www.ncbi.nlm.nih.gov/entrez/query.fcgi?db=gene&cmd=Retrieve&dopt=full_report&list_uids=144423) | glycosyltransferase 1 domain containing 1 |
| [Details](https://mirdb.org/cgi-bin/target_detail.cgi?targetID=3253237) | 1195 | 57 | hsa-miR-15a-5p | [SLC22A17](http://www.ncbi.nlm.nih.gov/entrez/query.fcgi?db=gene&cmd=Retrieve&dopt=full_report&list_uids=51310) | solute carrier family 22 member 17 |
| [Details](https://mirdb.org/cgi-bin/target_detail.cgi?targetID=3253245) | 1196 | 57 | hsa-miR-15a-5p | [GSG1](http://www.ncbi.nlm.nih.gov/entrez/query.fcgi?db=gene&cmd=Retrieve&dopt=full_report&list_uids=83445) | germ cell associated 1 |
| [Details](https://mirdb.org/cgi-bin/target_detail.cgi?targetID=3253254) | 1197 | 57 | hsa-miR-15a-5p | [RNF213](http://www.ncbi.nlm.nih.gov/entrez/query.fcgi?db=gene&cmd=Retrieve&dopt=full_report&list_uids=57674) | ring finger protein 213 |
| [Details](https://mirdb.org/cgi-bin/target_detail.cgi?targetID=3253324) | 1198 | 57 | hsa-miR-15a-5p | [AP5Z1](http://www.ncbi.nlm.nih.gov/entrez/query.fcgi?db=gene&cmd=Retrieve&dopt=full_report&list_uids=9907) | adaptor related protein complex 5 subunit zeta 1 |
| [Details](https://mirdb.org/cgi-bin/target_detail.cgi?targetID=3253326) | 1199 | 57 | hsa-miR-15a-5p | [SNCG](http://www.ncbi.nlm.nih.gov/entrez/query.fcgi?db=gene&cmd=Retrieve&dopt=full_report&list_uids=6623) | synuclein gamma |
| [Details](https://mirdb.org/cgi-bin/target_detail.cgi?targetID=3253328) | 1200 | 57 | hsa-miR-15a-5p | [IL17RE](http://www.ncbi.nlm.nih.gov/entrez/query.fcgi?db=gene&cmd=Retrieve&dopt=full_report&list_uids=132014) | interleukin 17 receptor E |
| [Details](https://mirdb.org/cgi-bin/target_detail.cgi?targetID=3253350) | 1201 | 57 | hsa-miR-15a-5p | [SLC45A2](http://www.ncbi.nlm.nih.gov/entrez/query.fcgi?db=gene&cmd=Retrieve&dopt=full_report&list_uids=51151) | solute carrier family 45 member 2 |
| [Details](https://mirdb.org/cgi-bin/target_detail.cgi?targetID=3253469) | 1202 | 57 | hsa-miR-15a-5p | [PURA](http://www.ncbi.nlm.nih.gov/entrez/query.fcgi?db=gene&cmd=Retrieve&dopt=full_report&list_uids=5813) | purine rich element binding protein A |
| [Details](https://mirdb.org/cgi-bin/target_detail.cgi?targetID=3253678) | 1203 | 57 | hsa-miR-15a-5p | [PPP2R1A](http://www.ncbi.nlm.nih.gov/entrez/query.fcgi?db=gene&cmd=Retrieve&dopt=full_report&list_uids=5518) | protein phosphatase 2 scaffold subunit Aalpha |
| [Details](https://mirdb.org/cgi-bin/target_detail.cgi?targetID=3253744) | 1204 | 57 | hsa-miR-15a-5p | [CHD6](http://www.ncbi.nlm.nih.gov/entrez/query.fcgi?db=gene&cmd=Retrieve&dopt=full_report&list_uids=84181) | chromodomain helicase DNA binding protein 6 |
| [Details](https://mirdb.org/cgi-bin/target_detail.cgi?targetID=3253746) | 1205 | 57 | hsa-miR-15a-5p | [ARMC8](http://www.ncbi.nlm.nih.gov/entrez/query.fcgi?db=gene&cmd=Retrieve&dopt=full_report&list_uids=25852) | armadillo repeat containing 8 |
| [Details](https://mirdb.org/cgi-bin/target_detail.cgi?targetID=3253748) | 1206 | 57 | hsa-miR-15a-5p | [CABLES2](http://www.ncbi.nlm.nih.gov/entrez/query.fcgi?db=gene&cmd=Retrieve&dopt=full_report&list_uids=81928) | Cdk5 and Abl enzyme substrate 2 |
| [Details](https://mirdb.org/cgi-bin/target_detail.cgi?targetID=3253778) | 1207 | 57 | hsa-miR-15a-5p | [BTF3](http://www.ncbi.nlm.nih.gov/entrez/query.fcgi?db=gene&cmd=Retrieve&dopt=full_report&list_uids=689) | basic transcription factor 3 |
| [Details](https://mirdb.org/cgi-bin/target_detail.cgi?targetID=3253810) | 1208 | 57 | hsa-miR-15a-5p | [ALOX12](http://www.ncbi.nlm.nih.gov/entrez/query.fcgi?db=gene&cmd=Retrieve&dopt=full_report&list_uids=239) | arachidonate 12-lipoxygenase, 12S type |
| [Details](https://mirdb.org/cgi-bin/target_detail.cgi?targetID=3253822) | 1209 | 57 | hsa-miR-15a-5p | [RASSF5](http://www.ncbi.nlm.nih.gov/entrez/query.fcgi?db=gene&cmd=Retrieve&dopt=full_report&list_uids=83593) | Ras association domain family member 5 |
| [Details](https://mirdb.org/cgi-bin/target_detail.cgi?targetID=3253849) | 1210 | 57 | hsa-miR-15a-5p | [PPP6C](http://www.ncbi.nlm.nih.gov/entrez/query.fcgi?db=gene&cmd=Retrieve&dopt=full_report&list_uids=5537) | protein phosphatase 6 catalytic subunit |
| [Details](https://mirdb.org/cgi-bin/target_detail.cgi?targetID=3254007) | 1211 | 57 | hsa-miR-15a-5p | [TMEM138](http://www.ncbi.nlm.nih.gov/entrez/query.fcgi?db=gene&cmd=Retrieve&dopt=full_report&list_uids=51524) | transmembrane protein 138 |
| [Details](https://mirdb.org/cgi-bin/target_detail.cgi?targetID=3252682) | 1212 | 56 | hsa-miR-15a-5p | [LOC100996842](http://www.ncbi.nlm.nih.gov/entrez/query.fcgi?db=gene&cmd=Retrieve&dopt=full_report&list_uids=100996842) | uncharacterized LOC100996842 |
| [Details](https://mirdb.org/cgi-bin/target_detail.cgi?targetID=3252722) | 1213 | 56 | hsa-miR-15a-5p | [PAGR1](http://www.ncbi.nlm.nih.gov/entrez/query.fcgi?db=gene&cmd=Retrieve&dopt=full_report&list_uids=79447) | PAXIP1 associated glutamate rich protein 1 |
| [Details](https://mirdb.org/cgi-bin/target_detail.cgi?targetID=3252754) | 1214 | 56 | hsa-miR-15a-5p | [TRAF6](http://www.ncbi.nlm.nih.gov/entrez/query.fcgi?db=gene&cmd=Retrieve&dopt=full_report&list_uids=7189) | TNF receptor associated factor 6 |
| [Details](https://mirdb.org/cgi-bin/target_detail.cgi?targetID=3252762) | 1215 | 56 | hsa-miR-15a-5p | [PTGFR](http://www.ncbi.nlm.nih.gov/entrez/query.fcgi?db=gene&cmd=Retrieve&dopt=full_report&list_uids=5737) | prostaglandin F receptor |
| [Details](https://mirdb.org/cgi-bin/target_detail.cgi?targetID=3252782) | 1216 | 56 | hsa-miR-15a-5p | [CNNM3](http://www.ncbi.nlm.nih.gov/entrez/query.fcgi?db=gene&cmd=Retrieve&dopt=full_report&list_uids=26505) | cyclin and CBS domain divalent metal cation transport mediator 3 |
| [Details](https://mirdb.org/cgi-bin/target_detail.cgi?targetID=3252875) | 1217 | 56 | hsa-miR-15a-5p | [MFAP3L](http://www.ncbi.nlm.nih.gov/entrez/query.fcgi?db=gene&cmd=Retrieve&dopt=full_report&list_uids=9848) | microfibril associated protein 3 like |
| [Details](https://mirdb.org/cgi-bin/target_detail.cgi?targetID=3253019) | 1218 | 56 | hsa-miR-15a-5p | [MLLT6](http://www.ncbi.nlm.nih.gov/entrez/query.fcgi?db=gene&cmd=Retrieve&dopt=full_report&list_uids=4302) | MLLT6, PHD finger containing |
| [Details](https://mirdb.org/cgi-bin/target_detail.cgi?targetID=3253032) | 1219 | 56 | hsa-miR-15a-5p | [AK9](http://www.ncbi.nlm.nih.gov/entrez/query.fcgi?db=gene&cmd=Retrieve&dopt=full_report&list_uids=221264) | adenylate kinase 9 |
| [Details](https://mirdb.org/cgi-bin/target_detail.cgi?targetID=3253060) | 1220 | 56 | hsa-miR-15a-5p | [DYNLT3](http://www.ncbi.nlm.nih.gov/entrez/query.fcgi?db=gene&cmd=Retrieve&dopt=full_report&list_uids=6990) | dynein light chain Tctex-type 3 |
| [Details](https://mirdb.org/cgi-bin/target_detail.cgi?targetID=3253079) | 1221 | 56 | hsa-miR-15a-5p | [VAMP8](http://www.ncbi.nlm.nih.gov/entrez/query.fcgi?db=gene&cmd=Retrieve&dopt=full_report&list_uids=8673) | vesicle associated membrane protein 8 |
| [Details](https://mirdb.org/cgi-bin/target_detail.cgi?targetID=3253083) | 1222 | 56 | hsa-miR-15a-5p | [SRP72](http://www.ncbi.nlm.nih.gov/entrez/query.fcgi?db=gene&cmd=Retrieve&dopt=full_report&list_uids=6731) | signal recognition particle 72 |
| [Details](https://mirdb.org/cgi-bin/target_detail.cgi?targetID=3253096) | 1223 | 56 | hsa-miR-15a-5p | [DPH5](http://www.ncbi.nlm.nih.gov/entrez/query.fcgi?db=gene&cmd=Retrieve&dopt=full_report&list_uids=51611) | diphthamide biosynthesis 5 |
| [Details](https://mirdb.org/cgi-bin/target_detail.cgi?targetID=3253119) | 1224 | 56 | hsa-miR-15a-5p | [PURG](http://www.ncbi.nlm.nih.gov/entrez/query.fcgi?db=gene&cmd=Retrieve&dopt=full_report&list_uids=29942) | purine rich element binding protein G |
| [Details](https://mirdb.org/cgi-bin/target_detail.cgi?targetID=3253154) | 1225 | 56 | hsa-miR-15a-5p | [TMEM33](http://www.ncbi.nlm.nih.gov/entrez/query.fcgi?db=gene&cmd=Retrieve&dopt=full_report&list_uids=55161) | transmembrane protein 33 |
| [Details](https://mirdb.org/cgi-bin/target_detail.cgi?targetID=3253206) | 1226 | 56 | hsa-miR-15a-5p | [SCN4B](http://www.ncbi.nlm.nih.gov/entrez/query.fcgi?db=gene&cmd=Retrieve&dopt=full_report&list_uids=6330) | sodium voltage-gated channel beta subunit 4 |
| [Details](https://mirdb.org/cgi-bin/target_detail.cgi?targetID=3253230) | 1227 | 56 | hsa-miR-15a-5p | [GOLGA4](http://www.ncbi.nlm.nih.gov/entrez/query.fcgi?db=gene&cmd=Retrieve&dopt=full_report&list_uids=2803) | golgin A4 |
| [Details](https://mirdb.org/cgi-bin/target_detail.cgi?targetID=3253441) | 1228 | 56 | hsa-miR-15a-5p | [BHLHE41](http://www.ncbi.nlm.nih.gov/entrez/query.fcgi?db=gene&cmd=Retrieve&dopt=full_report&list_uids=79365) | basic helix-loop-helix family member e41 |
| [Details](https://mirdb.org/cgi-bin/target_detail.cgi?targetID=3253489) | 1229 | 56 | hsa-miR-15a-5p | [PRR15L](http://www.ncbi.nlm.nih.gov/entrez/query.fcgi?db=gene&cmd=Retrieve&dopt=full_report&list_uids=79170) | proline rich 15 like |
| [Details](https://mirdb.org/cgi-bin/target_detail.cgi?targetID=3253544) | 1230 | 56 | hsa-miR-15a-5p | [PEX5](http://www.ncbi.nlm.nih.gov/entrez/query.fcgi?db=gene&cmd=Retrieve&dopt=full_report&list_uids=5830) | peroxisomal biogenesis factor 5 |
| [Details](https://mirdb.org/cgi-bin/target_detail.cgi?targetID=3253557) | 1231 | 56 | hsa-miR-15a-5p | [PSME3](http://www.ncbi.nlm.nih.gov/entrez/query.fcgi?db=gene&cmd=Retrieve&dopt=full_report&list_uids=10197) | proteasome activator subunit 3 |
| [Details](https://mirdb.org/cgi-bin/target_detail.cgi?targetID=3253625) | 1232 | 56 | hsa-miR-15a-5p | [CAST](http://www.ncbi.nlm.nih.gov/entrez/query.fcgi?db=gene&cmd=Retrieve&dopt=full_report&list_uids=831) | calpastatin |
| [Details](https://mirdb.org/cgi-bin/target_detail.cgi?targetID=3253696) | 1233 | 56 | hsa-miR-15a-5p | [SELENOS](http://www.ncbi.nlm.nih.gov/entrez/query.fcgi?db=gene&cmd=Retrieve&dopt=full_report&list_uids=55829) | selenoprotein S |
| [Details](https://mirdb.org/cgi-bin/target_detail.cgi?targetID=3253784) | 1234 | 56 | hsa-miR-15a-5p | [KRTAP4-4](http://www.ncbi.nlm.nih.gov/entrez/query.fcgi?db=gene&cmd=Retrieve&dopt=full_report&list_uids=84616) | keratin associated protein 4-4 |
| [Details](https://mirdb.org/cgi-bin/target_detail.cgi?targetID=3253789) | 1235 | 56 | hsa-miR-15a-5p | [USP32](http://www.ncbi.nlm.nih.gov/entrez/query.fcgi?db=gene&cmd=Retrieve&dopt=full_report&list_uids=84669) | ubiquitin specific peptidase 32 |
| [Details](https://mirdb.org/cgi-bin/target_detail.cgi?targetID=3253809) | 1236 | 56 | hsa-miR-15a-5p | [SH3BP2](http://www.ncbi.nlm.nih.gov/entrez/query.fcgi?db=gene&cmd=Retrieve&dopt=full_report&list_uids=6452) | SH3 domain binding protein 2 |
| [Details](https://mirdb.org/cgi-bin/target_detail.cgi?targetID=3253855) | 1237 | 56 | hsa-miR-15a-5p | [FAM174B](http://www.ncbi.nlm.nih.gov/entrez/query.fcgi?db=gene&cmd=Retrieve&dopt=full_report&list_uids=400451) | family with sequence similarity 174 member B |
| [Details](https://mirdb.org/cgi-bin/target_detail.cgi?targetID=3253968) | 1238 | 56 | hsa-miR-15a-5p | [CCDC81](http://www.ncbi.nlm.nih.gov/entrez/query.fcgi?db=gene&cmd=Retrieve&dopt=full_report&list_uids=60494) | coiled-coil domain containing 81 |
| [Details](https://mirdb.org/cgi-bin/target_detail.cgi?targetID=3253991) | 1239 | 56 | hsa-miR-15a-5p | [A4GNT](http://www.ncbi.nlm.nih.gov/entrez/query.fcgi?db=gene&cmd=Retrieve&dopt=full_report&list_uids=51146) | alpha-1,4-N-acetylglucosaminyltransferase |
| [Details](https://mirdb.org/cgi-bin/target_detail.cgi?targetID=3253996) | 1240 | 56 | hsa-miR-15a-5p | [ADGRD1](http://www.ncbi.nlm.nih.gov/entrez/query.fcgi?db=gene&cmd=Retrieve&dopt=full_report&list_uids=283383) | adhesion G protein-coupled receptor D1 |
| [Details](https://mirdb.org/cgi-bin/target_detail.cgi?targetID=3254014) | 1241 | 56 | hsa-miR-15a-5p | [ABI2](http://www.ncbi.nlm.nih.gov/entrez/query.fcgi?db=gene&cmd=Retrieve&dopt=full_report&list_uids=10152) | abl interactor 2 |
| [Details](https://mirdb.org/cgi-bin/target_detail.cgi?targetID=3252664) | 1242 | 55 | hsa-miR-15a-5p | [HOXA3](http://www.ncbi.nlm.nih.gov/entrez/query.fcgi?db=gene&cmd=Retrieve&dopt=full_report&list_uids=3200) | homeobox A3 |
| [Details](https://mirdb.org/cgi-bin/target_detail.cgi?targetID=3252690) | 1243 | 55 | hsa-miR-15a-5p | [NXPH1](http://www.ncbi.nlm.nih.gov/entrez/query.fcgi?db=gene&cmd=Retrieve&dopt=full_report&list_uids=30010) | neurexophilin 1 |
| [Details](https://mirdb.org/cgi-bin/target_detail.cgi?targetID=3252696) | 1244 | 55 | hsa-miR-15a-5p | [VAPB](http://www.ncbi.nlm.nih.gov/entrez/query.fcgi?db=gene&cmd=Retrieve&dopt=full_report&list_uids=9217) | VAMP associated protein B and C |
| [Details](https://mirdb.org/cgi-bin/target_detail.cgi?targetID=3252749) | 1245 | 55 | hsa-miR-15a-5p | [NSMCE3](http://www.ncbi.nlm.nih.gov/entrez/query.fcgi?db=gene&cmd=Retrieve&dopt=full_report&list_uids=56160) | NSE3 homolog, SMC5-SMC6 complex component |
| [Details](https://mirdb.org/cgi-bin/target_detail.cgi?targetID=3252752) | 1246 | 55 | hsa-miR-15a-5p | [SOX5](http://www.ncbi.nlm.nih.gov/entrez/query.fcgi?db=gene&cmd=Retrieve&dopt=full_report&list_uids=6660) | SRY-box 5 |
| [Details](https://mirdb.org/cgi-bin/target_detail.cgi?targetID=3252850) | 1247 | 55 | hsa-miR-15a-5p | [GCC1](http://www.ncbi.nlm.nih.gov/entrez/query.fcgi?db=gene&cmd=Retrieve&dopt=full_report&list_uids=79571) | GRIP and coiled-coil domain containing 1 |
| [Details](https://mirdb.org/cgi-bin/target_detail.cgi?targetID=3252965) | 1248 | 55 | hsa-miR-15a-5p | [TFEC](http://www.ncbi.nlm.nih.gov/entrez/query.fcgi?db=gene&cmd=Retrieve&dopt=full_report&list_uids=22797) | transcription factor EC |
| [Details](https://mirdb.org/cgi-bin/target_detail.cgi?targetID=3252980) | 1249 | 55 | hsa-miR-15a-5p | [SELENOI](http://www.ncbi.nlm.nih.gov/entrez/query.fcgi?db=gene&cmd=Retrieve&dopt=full_report&list_uids=85465) | selenoprotein I |
| [Details](https://mirdb.org/cgi-bin/target_detail.cgi?targetID=3253028) | 1250 | 55 | hsa-miR-15a-5p | [CPNE1](http://www.ncbi.nlm.nih.gov/entrez/query.fcgi?db=gene&cmd=Retrieve&dopt=full_report&list_uids=8904) | copine 1 |
| [Details](https://mirdb.org/cgi-bin/target_detail.cgi?targetID=3253047) | 1251 | 55 | hsa-miR-15a-5p | [AHNAK2](http://www.ncbi.nlm.nih.gov/entrez/query.fcgi?db=gene&cmd=Retrieve&dopt=full_report&list_uids=113146) | AHNAK nucleoprotein 2 |
| [Details](https://mirdb.org/cgi-bin/target_detail.cgi?targetID=3253061) | 1252 | 55 | hsa-miR-15a-5p | [CTDSPL](http://www.ncbi.nlm.nih.gov/entrez/query.fcgi?db=gene&cmd=Retrieve&dopt=full_report&list_uids=10217) | CTD small phosphatase like |
| [Details](https://mirdb.org/cgi-bin/target_detail.cgi?targetID=3253087) | 1253 | 55 | hsa-miR-15a-5p | [DNAJC5](http://www.ncbi.nlm.nih.gov/entrez/query.fcgi?db=gene&cmd=Retrieve&dopt=full_report&list_uids=80331) | DnaJ heat shock protein family (Hsp40) member C5 |
| [Details](https://mirdb.org/cgi-bin/target_detail.cgi?targetID=3253147) | 1254 | 55 | hsa-miR-15a-5p | [REEP1](http://www.ncbi.nlm.nih.gov/entrez/query.fcgi?db=gene&cmd=Retrieve&dopt=full_report&list_uids=65055) | receptor accessory protein 1 |
| [Details](https://mirdb.org/cgi-bin/target_detail.cgi?targetID=3253171) | 1255 | 55 | hsa-miR-15a-5p | [CA8](http://www.ncbi.nlm.nih.gov/entrez/query.fcgi?db=gene&cmd=Retrieve&dopt=full_report&list_uids=767) | carbonic anhydrase 8 |
| [Details](https://mirdb.org/cgi-bin/target_detail.cgi?targetID=3253173) | 1256 | 55 | hsa-miR-15a-5p | [CDK6](http://www.ncbi.nlm.nih.gov/entrez/query.fcgi?db=gene&cmd=Retrieve&dopt=full_report&list_uids=1021) | cyclin dependent kinase 6 |
| [Details](https://mirdb.org/cgi-bin/target_detail.cgi?targetID=3253199) | 1257 | 55 | hsa-miR-15a-5p | [ESS2](http://www.ncbi.nlm.nih.gov/entrez/query.fcgi?db=gene&cmd=Retrieve&dopt=full_report&list_uids=8220) | ess-2 splicing factor homolog |
| [Details](https://mirdb.org/cgi-bin/target_detail.cgi?targetID=3253286) | 1258 | 55 | hsa-miR-15a-5p | [SYT10](http://www.ncbi.nlm.nih.gov/entrez/query.fcgi?db=gene&cmd=Retrieve&dopt=full_report&list_uids=341359) | synaptotagmin 10 |
| [Details](https://mirdb.org/cgi-bin/target_detail.cgi?targetID=3253341) | 1259 | 55 | hsa-miR-15a-5p | [FBXO22](http://www.ncbi.nlm.nih.gov/entrez/query.fcgi?db=gene&cmd=Retrieve&dopt=full_report&list_uids=26263) | F-box protein 22 |
| [Details](https://mirdb.org/cgi-bin/target_detail.cgi?targetID=3253364) | 1260 | 55 | hsa-miR-15a-5p | [SEPT11](http://www.ncbi.nlm.nih.gov/entrez/query.fcgi?db=gene&cmd=Retrieve&dopt=full_report&list_uids=55752) | septin 11 |
| [Details](https://mirdb.org/cgi-bin/target_detail.cgi?targetID=3253385) | 1261 | 55 | hsa-miR-15a-5p | [AEBP2](http://www.ncbi.nlm.nih.gov/entrez/query.fcgi?db=gene&cmd=Retrieve&dopt=full_report&list_uids=121536) | AE binding protein 2 |
| [Details](https://mirdb.org/cgi-bin/target_detail.cgi?targetID=3253494) | 1262 | 55 | hsa-miR-15a-5p | [KLC4](http://www.ncbi.nlm.nih.gov/entrez/query.fcgi?db=gene&cmd=Retrieve&dopt=full_report&list_uids=89953) | kinesin light chain 4 |
| [Details](https://mirdb.org/cgi-bin/target_detail.cgi?targetID=3253495) | 1263 | 55 | hsa-miR-15a-5p | [SOGA1](http://www.ncbi.nlm.nih.gov/entrez/query.fcgi?db=gene&cmd=Retrieve&dopt=full_report&list_uids=140710) | suppressor of glucose, autophagy associated 1 |
| [Details](https://mirdb.org/cgi-bin/target_detail.cgi?targetID=3253548) | 1264 | 55 | hsa-miR-15a-5p | [TRIM36](http://www.ncbi.nlm.nih.gov/entrez/query.fcgi?db=gene&cmd=Retrieve&dopt=full_report&list_uids=55521) | tripartite motif containing 36 |
| [Details](https://mirdb.org/cgi-bin/target_detail.cgi?targetID=3253590) | 1265 | 55 | hsa-miR-15a-5p | [SMIM21](http://www.ncbi.nlm.nih.gov/entrez/query.fcgi?db=gene&cmd=Retrieve&dopt=full_report&list_uids=284274) | small integral membrane protein 21 |
| [Details](https://mirdb.org/cgi-bin/target_detail.cgi?targetID=3253681) | 1266 | 55 | hsa-miR-15a-5p | [TPM3](http://www.ncbi.nlm.nih.gov/entrez/query.fcgi?db=gene&cmd=Retrieve&dopt=full_report&list_uids=7170) | tropomyosin 3 |
| [Details](https://mirdb.org/cgi-bin/target_detail.cgi?targetID=3253720) | 1267 | 55 | hsa-miR-15a-5p | [RPRD1B](http://www.ncbi.nlm.nih.gov/entrez/query.fcgi?db=gene&cmd=Retrieve&dopt=full_report&list_uids=58490) | regulation of nuclear pre-mRNA domain containing 1B |
| [Details](https://mirdb.org/cgi-bin/target_detail.cgi?targetID=3253721) | 1268 | 55 | hsa-miR-15a-5p | [SZRD1](http://www.ncbi.nlm.nih.gov/entrez/query.fcgi?db=gene&cmd=Retrieve&dopt=full_report&list_uids=26099) | SUZ RNA binding domain containing 1 |
| [Details](https://mirdb.org/cgi-bin/target_detail.cgi?targetID=3253755) | 1269 | 55 | hsa-miR-15a-5p | [PARM1](http://www.ncbi.nlm.nih.gov/entrez/query.fcgi?db=gene&cmd=Retrieve&dopt=full_report&list_uids=25849) | prostate androgen-regulated mucin-like protein 1 |
| [Details](https://mirdb.org/cgi-bin/target_detail.cgi?targetID=3253773) | 1270 | 55 | hsa-miR-15a-5p | [ZBTB9](http://www.ncbi.nlm.nih.gov/entrez/query.fcgi?db=gene&cmd=Retrieve&dopt=full_report&list_uids=221504) | zinc finger and BTB domain containing 9 |
| [Details](https://mirdb.org/cgi-bin/target_detail.cgi?targetID=3253776) | 1271 | 55 | hsa-miR-15a-5p | [CLCN5](http://www.ncbi.nlm.nih.gov/entrez/query.fcgi?db=gene&cmd=Retrieve&dopt=full_report&list_uids=1184) | chloride voltage-gated channel 5 |
| [Details](https://mirdb.org/cgi-bin/target_detail.cgi?targetID=3253897) | 1272 | 55 | hsa-miR-15a-5p | [LRCH1](http://www.ncbi.nlm.nih.gov/entrez/query.fcgi?db=gene&cmd=Retrieve&dopt=full_report&list_uids=23143) | leucine rich repeats and calponin homology domain containing 1 |
| [Details](https://mirdb.org/cgi-bin/target_detail.cgi?targetID=3253939) | 1273 | 55 | hsa-miR-15a-5p | [THSD4](http://www.ncbi.nlm.nih.gov/entrez/query.fcgi?db=gene&cmd=Retrieve&dopt=full_report&list_uids=79875) | thrombospondin type 1 domain containing 4 |
| [Details](https://mirdb.org/cgi-bin/target_detail.cgi?targetID=3253940) | 1274 | 55 | hsa-miR-15a-5p | [C5orf64](http://www.ncbi.nlm.nih.gov/entrez/query.fcgi?db=gene&cmd=Retrieve&dopt=full_report&list_uids=285668) | chromosome 5 open reading frame 64 |
| [Details](https://mirdb.org/cgi-bin/target_detail.cgi?targetID=3253943) | 1275 | 55 | hsa-miR-15a-5p | [TIMM10B](http://www.ncbi.nlm.nih.gov/entrez/query.fcgi?db=gene&cmd=Retrieve&dopt=full_report&list_uids=26515) | translocase of inner mitochondrial membrane 10B |
| [Details](https://mirdb.org/cgi-bin/target_detail.cgi?targetID=3254021) | 1276 | 55 | hsa-miR-15a-5p | [HTR1B](http://www.ncbi.nlm.nih.gov/entrez/query.fcgi?db=gene&cmd=Retrieve&dopt=full_report&list_uids=3351) | 5-hydroxytryptamine receptor 1B |
| [Details](https://mirdb.org/cgi-bin/target_detail.cgi?targetID=3252721) | 1277 | 54 | hsa-miR-15a-5p | [CHRNB2](http://www.ncbi.nlm.nih.gov/entrez/query.fcgi?db=gene&cmd=Retrieve&dopt=full_report&list_uids=1141) | cholinergic receptor nicotinic beta 2 subunit |
| [Details](https://mirdb.org/cgi-bin/target_detail.cgi?targetID=3252727) | 1278 | 54 | hsa-miR-15a-5p | [ARHGEF12](http://www.ncbi.nlm.nih.gov/entrez/query.fcgi?db=gene&cmd=Retrieve&dopt=full_report&list_uids=23365) | Rho guanine nucleotide exchange factor 12 |
| [Details](https://mirdb.org/cgi-bin/target_detail.cgi?targetID=3252741) | 1279 | 54 | hsa-miR-15a-5p | [GDI2](http://www.ncbi.nlm.nih.gov/entrez/query.fcgi?db=gene&cmd=Retrieve&dopt=full_report&list_uids=2665) | GDP dissociation inhibitor 2 |
| [Details](https://mirdb.org/cgi-bin/target_detail.cgi?targetID=3252761) | 1280 | 54 | hsa-miR-15a-5p | [PRR11](http://www.ncbi.nlm.nih.gov/entrez/query.fcgi?db=gene&cmd=Retrieve&dopt=full_report&list_uids=55771) | proline rich 11 |
| [Details](https://mirdb.org/cgi-bin/target_detail.cgi?targetID=3252793) | 1281 | 54 | hsa-miR-15a-5p | [LRP10](http://www.ncbi.nlm.nih.gov/entrez/query.fcgi?db=gene&cmd=Retrieve&dopt=full_report&list_uids=26020) | LDL receptor related protein 10 |
| [Details](https://mirdb.org/cgi-bin/target_detail.cgi?targetID=3253021) | 1282 | 54 | hsa-miR-15a-5p | [AMMECR1L](http://www.ncbi.nlm.nih.gov/entrez/query.fcgi?db=gene&cmd=Retrieve&dopt=full_report&list_uids=83607) | AMMECR1 like |
| [Details](https://mirdb.org/cgi-bin/target_detail.cgi?targetID=3253078) | 1283 | 54 | hsa-miR-15a-5p | [ADAM10](http://www.ncbi.nlm.nih.gov/entrez/query.fcgi?db=gene&cmd=Retrieve&dopt=full_report&list_uids=102) | ADAM metallopeptidase domain 10 |
| [Details](https://mirdb.org/cgi-bin/target_detail.cgi?targetID=3253090) | 1284 | 54 | hsa-miR-15a-5p | [PRKAA1](http://www.ncbi.nlm.nih.gov/entrez/query.fcgi?db=gene&cmd=Retrieve&dopt=full_report&list_uids=5562) | protein kinase AMP-activated catalytic subunit alpha 1 |
| [Details](https://mirdb.org/cgi-bin/target_detail.cgi?targetID=3253102) | 1285 | 54 | hsa-miR-15a-5p | [SPEN](http://www.ncbi.nlm.nih.gov/entrez/query.fcgi?db=gene&cmd=Retrieve&dopt=full_report&list_uids=23013) | spen family transcriptional repressor |
| [Details](https://mirdb.org/cgi-bin/target_detail.cgi?targetID=3253122) | 1286 | 54 | hsa-miR-15a-5p | [GABRE](http://www.ncbi.nlm.nih.gov/entrez/query.fcgi?db=gene&cmd=Retrieve&dopt=full_report&list_uids=2564) | gamma-aminobutyric acid type A receptor epsilon subunit |
| [Details](https://mirdb.org/cgi-bin/target_detail.cgi?targetID=3253123) | 1287 | 54 | hsa-miR-15a-5p | [B3GNT2](http://www.ncbi.nlm.nih.gov/entrez/query.fcgi?db=gene&cmd=Retrieve&dopt=full_report&list_uids=10678) | UDP-GlcNAc:betaGal beta-1,3-N-acetylglucosaminyltransferase 2 |
| [Details](https://mirdb.org/cgi-bin/target_detail.cgi?targetID=3253138) | 1288 | 54 | hsa-miR-15a-5p | [GORASP1](http://www.ncbi.nlm.nih.gov/entrez/query.fcgi?db=gene&cmd=Retrieve&dopt=full_report&list_uids=64689) | golgi reassembly stacking protein 1 |
| [Details](https://mirdb.org/cgi-bin/target_detail.cgi?targetID=3253159) | 1289 | 54 | hsa-miR-15a-5p | [FSD1](http://www.ncbi.nlm.nih.gov/entrez/query.fcgi?db=gene&cmd=Retrieve&dopt=full_report&list_uids=79187) | fibronectin type III and SPRY domain containing 1 |
| [Details](https://mirdb.org/cgi-bin/target_detail.cgi?targetID=3253161) | 1290 | 54 | hsa-miR-15a-5p | [ADAP1](http://www.ncbi.nlm.nih.gov/entrez/query.fcgi?db=gene&cmd=Retrieve&dopt=full_report&list_uids=11033) | ArfGAP with dual PH domains 1 |
| [Details](https://mirdb.org/cgi-bin/target_detail.cgi?targetID=3253168) | 1291 | 54 | hsa-miR-15a-5p | [SWAP70](http://www.ncbi.nlm.nih.gov/entrez/query.fcgi?db=gene&cmd=Retrieve&dopt=full_report&list_uids=23075) | switching B cell complex subunit SWAP70 |
| [Details](https://mirdb.org/cgi-bin/target_detail.cgi?targetID=3253223) | 1292 | 54 | hsa-miR-15a-5p | [TECPR2](http://www.ncbi.nlm.nih.gov/entrez/query.fcgi?db=gene&cmd=Retrieve&dopt=full_report&list_uids=9895) | tectonin beta-propeller repeat containing 2 |
| [Details](https://mirdb.org/cgi-bin/target_detail.cgi?targetID=3253228) | 1293 | 54 | hsa-miR-15a-5p | [FGFR4](http://www.ncbi.nlm.nih.gov/entrez/query.fcgi?db=gene&cmd=Retrieve&dopt=full_report&list_uids=2264) | fibroblast growth factor receptor 4 |
| [Details](https://mirdb.org/cgi-bin/target_detail.cgi?targetID=3253236) | 1294 | 54 | hsa-miR-15a-5p | [DENR](http://www.ncbi.nlm.nih.gov/entrez/query.fcgi?db=gene&cmd=Retrieve&dopt=full_report&list_uids=8562) | density regulated re-initiation and release factor |
| [Details](https://mirdb.org/cgi-bin/target_detail.cgi?targetID=3253298) | 1295 | 54 | hsa-miR-15a-5p | [MBTPS2](http://www.ncbi.nlm.nih.gov/entrez/query.fcgi?db=gene&cmd=Retrieve&dopt=full_report&list_uids=51360) | membrane bound transcription factor peptidase, site 2 |
| [Details](https://mirdb.org/cgi-bin/target_detail.cgi?targetID=3253365) | 1296 | 54 | hsa-miR-15a-5p | [MYLK3](http://www.ncbi.nlm.nih.gov/entrez/query.fcgi?db=gene&cmd=Retrieve&dopt=full_report&list_uids=91807) | myosin light chain kinase 3 |
| [Details](https://mirdb.org/cgi-bin/target_detail.cgi?targetID=3253371) | 1297 | 54 | hsa-miR-15a-5p | [NEXMIF](http://www.ncbi.nlm.nih.gov/entrez/query.fcgi?db=gene&cmd=Retrieve&dopt=full_report&list_uids=340533) | neurite extension and migration factor |
| [Details](https://mirdb.org/cgi-bin/target_detail.cgi?targetID=3253432) | 1298 | 54 | hsa-miR-15a-5p | [ZDHHC14](http://www.ncbi.nlm.nih.gov/entrez/query.fcgi?db=gene&cmd=Retrieve&dopt=full_report&list_uids=79683) | zinc finger DHHC-type containing 14 |
| [Details](https://mirdb.org/cgi-bin/target_detail.cgi?targetID=3253461) | 1299 | 54 | hsa-miR-15a-5p | [FLCN](http://www.ncbi.nlm.nih.gov/entrez/query.fcgi?db=gene&cmd=Retrieve&dopt=full_report&list_uids=201163) | folliculin |
| [Details](https://mirdb.org/cgi-bin/target_detail.cgi?targetID=3253493) | 1300 | 54 | hsa-miR-15a-5p | [ANP32E](http://www.ncbi.nlm.nih.gov/entrez/query.fcgi?db=gene&cmd=Retrieve&dopt=full_report&list_uids=81611) | acidic nuclear phosphoprotein 32 family member E |
| [Details](https://mirdb.org/cgi-bin/target_detail.cgi?targetID=3253574) | 1301 | 54 | hsa-miR-15a-5p | [ZNF699](http://www.ncbi.nlm.nih.gov/entrez/query.fcgi?db=gene&cmd=Retrieve&dopt=full_report&list_uids=374879) | zinc finger protein 699 |
| [Details](https://mirdb.org/cgi-bin/target_detail.cgi?targetID=3253610) | 1302 | 54 | hsa-miR-15a-5p | [SLC9A8](http://www.ncbi.nlm.nih.gov/entrez/query.fcgi?db=gene&cmd=Retrieve&dopt=full_report&list_uids=23315) | solute carrier family 9 member A8 |
| [Details](https://mirdb.org/cgi-bin/target_detail.cgi?targetID=3253772) | 1303 | 54 | hsa-miR-15a-5p | [FRS2](http://www.ncbi.nlm.nih.gov/entrez/query.fcgi?db=gene&cmd=Retrieve&dopt=full_report&list_uids=10818) | fibroblast growth factor receptor substrate 2 |
| [Details](https://mirdb.org/cgi-bin/target_detail.cgi?targetID=3253874) | 1304 | 54 | hsa-miR-15a-5p | [GNPDA2](http://www.ncbi.nlm.nih.gov/entrez/query.fcgi?db=gene&cmd=Retrieve&dopt=full_report&list_uids=132789) | glucosamine-6-phosphate deaminase 2 |
| [Details](https://mirdb.org/cgi-bin/target_detail.cgi?targetID=3253878) | 1305 | 54 | hsa-miR-15a-5p | [MRPS2](http://www.ncbi.nlm.nih.gov/entrez/query.fcgi?db=gene&cmd=Retrieve&dopt=full_report&list_uids=51116) | mitochondrial ribosomal protein S2 |
| [Details](https://mirdb.org/cgi-bin/target_detail.cgi?targetID=3253958) | 1306 | 54 | hsa-miR-15a-5p | [MTMR11](http://www.ncbi.nlm.nih.gov/entrez/query.fcgi?db=gene&cmd=Retrieve&dopt=full_report&list_uids=10903) | myotubularin related protein 11 |
| [Details](https://mirdb.org/cgi-bin/target_detail.cgi?targetID=3252643) | 1307 | 53 | hsa-miR-15a-5p | [CNTN3](http://www.ncbi.nlm.nih.gov/entrez/query.fcgi?db=gene&cmd=Retrieve&dopt=full_report&list_uids=5067) | contactin 3 |
| [Details](https://mirdb.org/cgi-bin/target_detail.cgi?targetID=3252705) | 1308 | 53 | hsa-miR-15a-5p | [TTC38](http://www.ncbi.nlm.nih.gov/entrez/query.fcgi?db=gene&cmd=Retrieve&dopt=full_report&list_uids=55020) | tetratricopeptide repeat domain 38 |
| [Details](https://mirdb.org/cgi-bin/target_detail.cgi?targetID=3252812) | 1309 | 53 | hsa-miR-15a-5p | [AP1S3](http://www.ncbi.nlm.nih.gov/entrez/query.fcgi?db=gene&cmd=Retrieve&dopt=full_report&list_uids=130340) | adaptor related protein complex 1 subunit sigma 3 |
| [Details](https://mirdb.org/cgi-bin/target_detail.cgi?targetID=3252848) | 1310 | 53 | hsa-miR-15a-5p | [SBNO1](http://www.ncbi.nlm.nih.gov/entrez/query.fcgi?db=gene&cmd=Retrieve&dopt=full_report&list_uids=55206) | strawberry notch homolog 1 |
| [Details](https://mirdb.org/cgi-bin/target_detail.cgi?targetID=3252866) | 1311 | 53 | hsa-miR-15a-5p | [DCAF10](http://www.ncbi.nlm.nih.gov/entrez/query.fcgi?db=gene&cmd=Retrieve&dopt=full_report&list_uids=79269) | DDB1 and CUL4 associated factor 10 |
| [Details](https://mirdb.org/cgi-bin/target_detail.cgi?targetID=3252937) | 1312 | 53 | hsa-miR-15a-5p | [EPB41L1](http://www.ncbi.nlm.nih.gov/entrez/query.fcgi?db=gene&cmd=Retrieve&dopt=full_report&list_uids=2036) | erythrocyte membrane protein band 4.1 like 1 |
| [Details](https://mirdb.org/cgi-bin/target_detail.cgi?targetID=3253024) | 1313 | 53 | hsa-miR-15a-5p | [DAZAP2](http://www.ncbi.nlm.nih.gov/entrez/query.fcgi?db=gene&cmd=Retrieve&dopt=full_report&list_uids=9802) | DAZ associated protein 2 |
| [Details](https://mirdb.org/cgi-bin/target_detail.cgi?targetID=3253126) | 1314 | 53 | hsa-miR-15a-5p | [ZNF609](http://www.ncbi.nlm.nih.gov/entrez/query.fcgi?db=gene&cmd=Retrieve&dopt=full_report&list_uids=23060) | zinc finger protein 609 |
| [Details](https://mirdb.org/cgi-bin/target_detail.cgi?targetID=3253184) | 1315 | 53 | hsa-miR-15a-5p | [C11orf53](http://www.ncbi.nlm.nih.gov/entrez/query.fcgi?db=gene&cmd=Retrieve&dopt=full_report&list_uids=341032) | chromosome 11 open reading frame 53 |
| [Details](https://mirdb.org/cgi-bin/target_detail.cgi?targetID=3253198) | 1316 | 53 | hsa-miR-15a-5p | [DNAJA1](http://www.ncbi.nlm.nih.gov/entrez/query.fcgi?db=gene&cmd=Retrieve&dopt=full_report&list_uids=3301) | DnaJ heat shock protein family (Hsp40) member A1 |
| [Details](https://mirdb.org/cgi-bin/target_detail.cgi?targetID=3253217) | 1317 | 53 | hsa-miR-15a-5p | [LMAN2L](http://www.ncbi.nlm.nih.gov/entrez/query.fcgi?db=gene&cmd=Retrieve&dopt=full_report&list_uids=81562) | lectin, mannose binding 2 like |
| [Details](https://mirdb.org/cgi-bin/target_detail.cgi?targetID=3253257) | 1318 | 53 | hsa-miR-15a-5p | [MCFD2](http://www.ncbi.nlm.nih.gov/entrez/query.fcgi?db=gene&cmd=Retrieve&dopt=full_report&list_uids=90411) | multiple coagulation factor deficiency 2 |
| [Details](https://mirdb.org/cgi-bin/target_detail.cgi?targetID=3253260) | 1319 | 53 | hsa-miR-15a-5p | [ZNF81](http://www.ncbi.nlm.nih.gov/entrez/query.fcgi?db=gene&cmd=Retrieve&dopt=full_report&list_uids=347344) | zinc finger protein 81 |
| [Details](https://mirdb.org/cgi-bin/target_detail.cgi?targetID=3253262) | 1320 | 53 | hsa-miR-15a-5p | [KCNJ11](http://www.ncbi.nlm.nih.gov/entrez/query.fcgi?db=gene&cmd=Retrieve&dopt=full_report&list_uids=3767) | potassium voltage-gated channel subfamily J member 11 |
| [Details](https://mirdb.org/cgi-bin/target_detail.cgi?targetID=3253278) | 1321 | 53 | hsa-miR-15a-5p | [MOCS3](http://www.ncbi.nlm.nih.gov/entrez/query.fcgi?db=gene&cmd=Retrieve&dopt=full_report&list_uids=27304) | molybdenum cofactor synthesis 3 |
| [Details](https://mirdb.org/cgi-bin/target_detail.cgi?targetID=3253303) | 1322 | 53 | hsa-miR-15a-5p | [SLC38A7](http://www.ncbi.nlm.nih.gov/entrez/query.fcgi?db=gene&cmd=Retrieve&dopt=full_report&list_uids=55238) | solute carrier family 38 member 7 |
| [Details](https://mirdb.org/cgi-bin/target_detail.cgi?targetID=3253393) | 1323 | 53 | hsa-miR-15a-5p | [PIM1](http://www.ncbi.nlm.nih.gov/entrez/query.fcgi?db=gene&cmd=Retrieve&dopt=full_report&list_uids=5292) | Pim-1 proto-oncogene, serine/threonine kinase |
| [Details](https://mirdb.org/cgi-bin/target_detail.cgi?targetID=3253553) | 1324 | 53 | hsa-miR-15a-5p | [NYNRIN](http://www.ncbi.nlm.nih.gov/entrez/query.fcgi?db=gene&cmd=Retrieve&dopt=full_report&list_uids=57523) | NYN domain and retroviral integrase containing |
| [Details](https://mirdb.org/cgi-bin/target_detail.cgi?targetID=3253734) | 1325 | 53 | hsa-miR-15a-5p | [ANKRD11](http://www.ncbi.nlm.nih.gov/entrez/query.fcgi?db=gene&cmd=Retrieve&dopt=full_report&list_uids=29123) | ankyrin repeat domain 11 |
| [Details](https://mirdb.org/cgi-bin/target_detail.cgi?targetID=3253742) | 1326 | 53 | hsa-miR-15a-5p | [KLC1](http://www.ncbi.nlm.nih.gov/entrez/query.fcgi?db=gene&cmd=Retrieve&dopt=full_report&list_uids=3831) | kinesin light chain 1 |
| [Details](https://mirdb.org/cgi-bin/target_detail.cgi?targetID=3253826) | 1327 | 53 | hsa-miR-15a-5p | [BRWD1](http://www.ncbi.nlm.nih.gov/entrez/query.fcgi?db=gene&cmd=Retrieve&dopt=full_report&list_uids=54014) | bromodomain and WD repeat domain containing 1 |
| [Details](https://mirdb.org/cgi-bin/target_detail.cgi?targetID=3253963) | 1328 | 53 | hsa-miR-15a-5p | [TXN2](http://www.ncbi.nlm.nih.gov/entrez/query.fcgi?db=gene&cmd=Retrieve&dopt=full_report&list_uids=25828) | thioredoxin 2 |
| [Details](https://mirdb.org/cgi-bin/target_detail.cgi?targetID=3253971) | 1329 | 53 | hsa-miR-15a-5p | [ZC3H12C](http://www.ncbi.nlm.nih.gov/entrez/query.fcgi?db=gene&cmd=Retrieve&dopt=full_report&list_uids=85463) | zinc finger CCCH-type containing 12C |
| [Details](https://mirdb.org/cgi-bin/target_detail.cgi?targetID=3253998) | 1330 | 53 | hsa-miR-15a-5p | [ZNF497](http://www.ncbi.nlm.nih.gov/entrez/query.fcgi?db=gene&cmd=Retrieve&dopt=full_report&list_uids=162968) | zinc finger protein 497 |
| [Details](https://mirdb.org/cgi-bin/target_detail.cgi?targetID=3254003) | 1331 | 53 | hsa-miR-15a-5p | [WNT2B](http://www.ncbi.nlm.nih.gov/entrez/query.fcgi?db=gene&cmd=Retrieve&dopt=full_report&list_uids=7482) | Wnt family member 2B |
| [Details](https://mirdb.org/cgi-bin/target_detail.cgi?targetID=3252642) | 1332 | 52 | hsa-miR-15a-5p | [VTCN1](http://www.ncbi.nlm.nih.gov/entrez/query.fcgi?db=gene&cmd=Retrieve&dopt=full_report&list_uids=79679) | V-set domain containing T cell activation inhibitor 1 |
| [Details](https://mirdb.org/cgi-bin/target_detail.cgi?targetID=3252663) | 1333 | 52 | hsa-miR-15a-5p | [IDH3A](http://www.ncbi.nlm.nih.gov/entrez/query.fcgi?db=gene&cmd=Retrieve&dopt=full_report&list_uids=3419) | isocitrate dehydrogenase 3 (NAD(+)) alpha |
| [Details](https://mirdb.org/cgi-bin/target_detail.cgi?targetID=3252723) | 1334 | 52 | hsa-miR-15a-5p | [TUBGCP2](http://www.ncbi.nlm.nih.gov/entrez/query.fcgi?db=gene&cmd=Retrieve&dopt=full_report&list_uids=10844) | tubulin gamma complex associated protein 2 |
| [Details](https://mirdb.org/cgi-bin/target_detail.cgi?targetID=3252751) | 1335 | 52 | hsa-miR-15a-5p | [EIF2B2](http://www.ncbi.nlm.nih.gov/entrez/query.fcgi?db=gene&cmd=Retrieve&dopt=full_report&list_uids=8892) | eukaryotic translation initiation factor 2B subunit beta |
| [Details](https://mirdb.org/cgi-bin/target_detail.cgi?targetID=3252796) | 1336 | 52 | hsa-miR-15a-5p | [MRAS](http://www.ncbi.nlm.nih.gov/entrez/query.fcgi?db=gene&cmd=Retrieve&dopt=full_report&list_uids=22808) | muscle RAS oncogene homolog |
| [Details](https://mirdb.org/cgi-bin/target_detail.cgi?targetID=3252810) | 1337 | 52 | hsa-miR-15a-5p | [MYCL](http://www.ncbi.nlm.nih.gov/entrez/query.fcgi?db=gene&cmd=Retrieve&dopt=full_report&list_uids=4610) | MYCL proto-oncogene, bHLH transcription factor |
| [Details](https://mirdb.org/cgi-bin/target_detail.cgi?targetID=3252840) | 1338 | 52 | hsa-miR-15a-5p | [ONECUT2](http://www.ncbi.nlm.nih.gov/entrez/query.fcgi?db=gene&cmd=Retrieve&dopt=full_report&list_uids=9480) | one cut homeobox 2 |
| [Details](https://mirdb.org/cgi-bin/target_detail.cgi?targetID=3252856) | 1339 | 52 | hsa-miR-15a-5p | [C1orf226](http://www.ncbi.nlm.nih.gov/entrez/query.fcgi?db=gene&cmd=Retrieve&dopt=full_report&list_uids=400793) | chromosome 1 open reading frame 226 |
| [Details](https://mirdb.org/cgi-bin/target_detail.cgi?targetID=3252873) | 1340 | 52 | hsa-miR-15a-5p | [GDPD1](http://www.ncbi.nlm.nih.gov/entrez/query.fcgi?db=gene&cmd=Retrieve&dopt=full_report&list_uids=284161) | glycerophosphodiester phosphodiesterase domain containing 1 |
| [Details](https://mirdb.org/cgi-bin/target_detail.cgi?targetID=3252916) | 1341 | 52 | hsa-miR-15a-5p | [NKD1](http://www.ncbi.nlm.nih.gov/entrez/query.fcgi?db=gene&cmd=Retrieve&dopt=full_report&list_uids=85407) | NKD1, WNT signaling pathway inhibitor |
| [Details](https://mirdb.org/cgi-bin/target_detail.cgi?targetID=3252995) | 1342 | 52 | hsa-miR-15a-5p | [SELENOO](http://www.ncbi.nlm.nih.gov/entrez/query.fcgi?db=gene&cmd=Retrieve&dopt=full_report&list_uids=83642) | selenoprotein O |
| [Details](https://mirdb.org/cgi-bin/target_detail.cgi?targetID=3253098) | 1343 | 52 | hsa-miR-15a-5p | [UVSSA](http://www.ncbi.nlm.nih.gov/entrez/query.fcgi?db=gene&cmd=Retrieve&dopt=full_report&list_uids=57654) | UV stimulated scaffold protein A |
| [Details](https://mirdb.org/cgi-bin/target_detail.cgi?targetID=3253110) | 1344 | 52 | hsa-miR-15a-5p | [SPEG](http://www.ncbi.nlm.nih.gov/entrez/query.fcgi?db=gene&cmd=Retrieve&dopt=full_report&list_uids=10290) | striated muscle enriched protein kinase |
| [Details](https://mirdb.org/cgi-bin/target_detail.cgi?targetID=3253140) | 1345 | 52 | hsa-miR-15a-5p | [PHF21A](http://www.ncbi.nlm.nih.gov/entrez/query.fcgi?db=gene&cmd=Retrieve&dopt=full_report&list_uids=51317) | PHD finger protein 21A |
| [Details](https://mirdb.org/cgi-bin/target_detail.cgi?targetID=3253235) | 1346 | 52 | hsa-miR-15a-5p | [SLC7A2](http://www.ncbi.nlm.nih.gov/entrez/query.fcgi?db=gene&cmd=Retrieve&dopt=full_report&list_uids=6542) | solute carrier family 7 member 2 |
| [Details](https://mirdb.org/cgi-bin/target_detail.cgi?targetID=3253272) | 1347 | 52 | hsa-miR-15a-5p | [FBXO10](http://www.ncbi.nlm.nih.gov/entrez/query.fcgi?db=gene&cmd=Retrieve&dopt=full_report&list_uids=26267) | F-box protein 10 |
| [Details](https://mirdb.org/cgi-bin/target_detail.cgi?targetID=3253274) | 1348 | 52 | hsa-miR-15a-5p | [UBAP1](http://www.ncbi.nlm.nih.gov/entrez/query.fcgi?db=gene&cmd=Retrieve&dopt=full_report&list_uids=51271) | ubiquitin associated protein 1 |
| [Details](https://mirdb.org/cgi-bin/target_detail.cgi?targetID=3253311) | 1349 | 52 | hsa-miR-15a-5p | [XIRP2](http://www.ncbi.nlm.nih.gov/entrez/query.fcgi?db=gene&cmd=Retrieve&dopt=full_report&list_uids=129446) | xin actin binding repeat containing 2 |
| [Details](https://mirdb.org/cgi-bin/target_detail.cgi?targetID=3253353) | 1350 | 52 | hsa-miR-15a-5p | [PYM1](http://www.ncbi.nlm.nih.gov/entrez/query.fcgi?db=gene&cmd=Retrieve&dopt=full_report&list_uids=84305) | PYM homolog 1, exon junction complex associated factor |
| [Details](https://mirdb.org/cgi-bin/target_detail.cgi?targetID=3253454) | 1351 | 52 | hsa-miR-15a-5p | [TRAF3](http://www.ncbi.nlm.nih.gov/entrez/query.fcgi?db=gene&cmd=Retrieve&dopt=full_report&list_uids=7187) | TNF receptor associated factor 3 |
| [Details](https://mirdb.org/cgi-bin/target_detail.cgi?targetID=3253602) | 1352 | 52 | hsa-miR-15a-5p | [EIF1AX](http://www.ncbi.nlm.nih.gov/entrez/query.fcgi?db=gene&cmd=Retrieve&dopt=full_report&list_uids=1964) | eukaryotic translation initiation factor 1A X-linked |
| [Details](https://mirdb.org/cgi-bin/target_detail.cgi?targetID=3253632) | 1353 | 52 | hsa-miR-15a-5p | [PDCD11](http://www.ncbi.nlm.nih.gov/entrez/query.fcgi?db=gene&cmd=Retrieve&dopt=full_report&list_uids=22984) | programmed cell death 11 |
| [Details](https://mirdb.org/cgi-bin/target_detail.cgi?targetID=3253643) | 1354 | 52 | hsa-miR-15a-5p | [GCNT3](http://www.ncbi.nlm.nih.gov/entrez/query.fcgi?db=gene&cmd=Retrieve&dopt=full_report&list_uids=9245) | glucosaminyl (N-acetyl) transferase 3, mucin type |
| [Details](https://mirdb.org/cgi-bin/target_detail.cgi?targetID=3253644) | 1355 | 52 | hsa-miR-15a-5p | [TUFT1](http://www.ncbi.nlm.nih.gov/entrez/query.fcgi?db=gene&cmd=Retrieve&dopt=full_report&list_uids=7286) | tuftelin 1 |
| [Details](https://mirdb.org/cgi-bin/target_detail.cgi?targetID=3253653) | 1356 | 52 | hsa-miR-15a-5p | [ITPRIPL2](http://www.ncbi.nlm.nih.gov/entrez/query.fcgi?db=gene&cmd=Retrieve&dopt=full_report&list_uids=162073) | ITPRIP like 2 |
| [Details](https://mirdb.org/cgi-bin/target_detail.cgi?targetID=3253679) | 1357 | 52 | hsa-miR-15a-5p | [RLIM](http://www.ncbi.nlm.nih.gov/entrez/query.fcgi?db=gene&cmd=Retrieve&dopt=full_report&list_uids=51132) | ring finger protein, LIM domain interacting |
| [Details](https://mirdb.org/cgi-bin/target_detail.cgi?targetID=3253697) | 1358 | 52 | hsa-miR-15a-5p | [HIRA](http://www.ncbi.nlm.nih.gov/entrez/query.fcgi?db=gene&cmd=Retrieve&dopt=full_report&list_uids=7290) | histone cell cycle regulator |
| [Details](https://mirdb.org/cgi-bin/target_detail.cgi?targetID=3253727) | 1359 | 52 | hsa-miR-15a-5p | [PSMF1](http://www.ncbi.nlm.nih.gov/entrez/query.fcgi?db=gene&cmd=Retrieve&dopt=full_report&list_uids=9491) | proteasome inhibitor subunit 1 |
| [Details](https://mirdb.org/cgi-bin/target_detail.cgi?targetID=3253808) | 1360 | 52 | hsa-miR-15a-5p | [TPRG1L](http://www.ncbi.nlm.nih.gov/entrez/query.fcgi?db=gene&cmd=Retrieve&dopt=full_report&list_uids=127262) | tumor protein p63 regulated 1 like |
| [Details](https://mirdb.org/cgi-bin/target_detail.cgi?targetID=3253909) | 1361 | 52 | hsa-miR-15a-5p | [UBE2K](http://www.ncbi.nlm.nih.gov/entrez/query.fcgi?db=gene&cmd=Retrieve&dopt=full_report&list_uids=3093) | ubiquitin conjugating enzyme E2 K |
| [Details](https://mirdb.org/cgi-bin/target_detail.cgi?targetID=3253973) | 1362 | 52 | hsa-miR-15a-5p | [CNN1](http://www.ncbi.nlm.nih.gov/entrez/query.fcgi?db=gene&cmd=Retrieve&dopt=full_report&list_uids=1264) | calponin 1 |
| [Details](https://mirdb.org/cgi-bin/target_detail.cgi?targetID=3252655) | 1363 | 51 | hsa-miR-15a-5p | [ABHD13](http://www.ncbi.nlm.nih.gov/entrez/query.fcgi?db=gene&cmd=Retrieve&dopt=full_report&list_uids=84945) | abhydrolase domain containing 13 |
| [Details](https://mirdb.org/cgi-bin/target_detail.cgi?targetID=3252729) | 1364 | 51 | hsa-miR-15a-5p | [LRRTM2](http://www.ncbi.nlm.nih.gov/entrez/query.fcgi?db=gene&cmd=Retrieve&dopt=full_report&list_uids=26045) | leucine rich repeat transmembrane neuronal 2 |
| [Details](https://mirdb.org/cgi-bin/target_detail.cgi?targetID=3252794) | 1365 | 51 | hsa-miR-15a-5p | [ADNP2](http://www.ncbi.nlm.nih.gov/entrez/query.fcgi?db=gene&cmd=Retrieve&dopt=full_report&list_uids=22850) | ADNP homeobox 2 |
| [Details](https://mirdb.org/cgi-bin/target_detail.cgi?targetID=3252814) | 1366 | 51 | hsa-miR-15a-5p | [PEAK1](http://www.ncbi.nlm.nih.gov/entrez/query.fcgi?db=gene&cmd=Retrieve&dopt=full_report&list_uids=79834) | pseudopodium enriched atypical kinase 1 |
| [Details](https://mirdb.org/cgi-bin/target_detail.cgi?targetID=3252872) | 1367 | 51 | hsa-miR-15a-5p | [CHD9](http://www.ncbi.nlm.nih.gov/entrez/query.fcgi?db=gene&cmd=Retrieve&dopt=full_report&list_uids=80205) | chromodomain helicase DNA binding protein 9 |
| [Details](https://mirdb.org/cgi-bin/target_detail.cgi?targetID=3252884) | 1368 | 51 | hsa-miR-15a-5p | [LYZL4](http://www.ncbi.nlm.nih.gov/entrez/query.fcgi?db=gene&cmd=Retrieve&dopt=full_report&list_uids=131375) | lysozyme like 4 |
| [Details](https://mirdb.org/cgi-bin/target_detail.cgi?targetID=3252959) | 1369 | 51 | hsa-miR-15a-5p | [DICER1](http://www.ncbi.nlm.nih.gov/entrez/query.fcgi?db=gene&cmd=Retrieve&dopt=full_report&list_uids=23405) | dicer 1, ribonuclease III |
| [Details](https://mirdb.org/cgi-bin/target_detail.cgi?targetID=3252975) | 1370 | 51 | hsa-miR-15a-5p | [XPO4](http://www.ncbi.nlm.nih.gov/entrez/query.fcgi?db=gene&cmd=Retrieve&dopt=full_report&list_uids=64328) | exportin 4 |
| [Details](https://mirdb.org/cgi-bin/target_detail.cgi?targetID=3253059) | 1371 | 51 | hsa-miR-15a-5p | [FAM161A](http://www.ncbi.nlm.nih.gov/entrez/query.fcgi?db=gene&cmd=Retrieve&dopt=full_report&list_uids=84140) | FAM161A, centrosomal protein |
| [Details](https://mirdb.org/cgi-bin/target_detail.cgi?targetID=3253112) | 1372 | 51 | hsa-miR-15a-5p | [PIGB](http://www.ncbi.nlm.nih.gov/entrez/query.fcgi?db=gene&cmd=Retrieve&dopt=full_report&list_uids=9488) | phosphatidylinositol glycan anchor biosynthesis class B |
| [Details](https://mirdb.org/cgi-bin/target_detail.cgi?targetID=3253259) | 1373 | 51 | hsa-miR-15a-5p | [RGPD6](http://www.ncbi.nlm.nih.gov/entrez/query.fcgi?db=gene&cmd=Retrieve&dopt=full_report&list_uids=729540) | RANBP2-like and GRIP domain containing 6 |
| [Details](https://mirdb.org/cgi-bin/target_detail.cgi?targetID=3253267) | 1374 | 51 | hsa-miR-15a-5p | [TP53INP1](http://www.ncbi.nlm.nih.gov/entrez/query.fcgi?db=gene&cmd=Retrieve&dopt=full_report&list_uids=94241) | tumor protein p53 inducible nuclear protein 1 |
| [Details](https://mirdb.org/cgi-bin/target_detail.cgi?targetID=3253289) | 1375 | 51 | hsa-miR-15a-5p | [IFFO2](http://www.ncbi.nlm.nih.gov/entrez/query.fcgi?db=gene&cmd=Retrieve&dopt=full_report&list_uids=126917) | intermediate filament family orphan 2 |
| [Details](https://mirdb.org/cgi-bin/target_detail.cgi?targetID=3253294) | 1376 | 51 | hsa-miR-15a-5p | [PTEN](http://www.ncbi.nlm.nih.gov/entrez/query.fcgi?db=gene&cmd=Retrieve&dopt=full_report&list_uids=5728) | phosphatase and tensin homolog |
| [Details](https://mirdb.org/cgi-bin/target_detail.cgi?targetID=3253453) | 1377 | 51 | hsa-miR-15a-5p | [WDR47](http://www.ncbi.nlm.nih.gov/entrez/query.fcgi?db=gene&cmd=Retrieve&dopt=full_report&list_uids=22911) | WD repeat domain 47 |
| [Details](https://mirdb.org/cgi-bin/target_detail.cgi?targetID=3253604) | 1378 | 51 | hsa-miR-15a-5p | [SMYD5](http://www.ncbi.nlm.nih.gov/entrez/query.fcgi?db=gene&cmd=Retrieve&dopt=full_report&list_uids=10322) | SMYD family member 5 |
| [Details](https://mirdb.org/cgi-bin/target_detail.cgi?targetID=3253620) | 1379 | 51 | hsa-miR-15a-5p | [C14orf180](http://www.ncbi.nlm.nih.gov/entrez/query.fcgi?db=gene&cmd=Retrieve&dopt=full_report&list_uids=400258) | chromosome 14 open reading frame 180 |
| [Details](https://mirdb.org/cgi-bin/target_detail.cgi?targetID=3253627) | 1380 | 51 | hsa-miR-15a-5p | [CHFR](http://www.ncbi.nlm.nih.gov/entrez/query.fcgi?db=gene&cmd=Retrieve&dopt=full_report&list_uids=55743) | checkpoint with forkhead and ring finger domains |
| [Details](https://mirdb.org/cgi-bin/target_detail.cgi?targetID=3253635) | 1381 | 51 | hsa-miR-15a-5p | [MCRIP1](http://www.ncbi.nlm.nih.gov/entrez/query.fcgi?db=gene&cmd=Retrieve&dopt=full_report&list_uids=348262) | MAPK regulated corepressor interacting protein 1 |
| [Details](https://mirdb.org/cgi-bin/target_detail.cgi?targetID=3253636) | 1382 | 51 | hsa-miR-15a-5p | [C11orf68](http://www.ncbi.nlm.nih.gov/entrez/query.fcgi?db=gene&cmd=Retrieve&dopt=full_report&list_uids=83638) | chromosome 11 open reading frame 68 |
| [Details](https://mirdb.org/cgi-bin/target_detail.cgi?targetID=3253708) | 1383 | 51 | hsa-miR-15a-5p | [FAM84B](http://www.ncbi.nlm.nih.gov/entrez/query.fcgi?db=gene&cmd=Retrieve&dopt=full_report&list_uids=157638) | family with sequence similarity 84 member B |
| [Details](https://mirdb.org/cgi-bin/target_detail.cgi?targetID=3253714) | 1384 | 51 | hsa-miR-15a-5p | [KARS](http://www.ncbi.nlm.nih.gov/entrez/query.fcgi?db=gene&cmd=Retrieve&dopt=full_report&list_uids=3735) | lysyl-tRNA synthetase |
| [Details](https://mirdb.org/cgi-bin/target_detail.cgi?targetID=3253782) | 1385 | 51 | hsa-miR-15a-5p | [ANKRD34A](http://www.ncbi.nlm.nih.gov/entrez/query.fcgi?db=gene&cmd=Retrieve&dopt=full_report&list_uids=284615) | ankyrin repeat domain 34A |
| [Details](https://mirdb.org/cgi-bin/target_detail.cgi?targetID=3253787) | 1386 | 51 | hsa-miR-15a-5p | [NSD2](http://www.ncbi.nlm.nih.gov/entrez/query.fcgi?db=gene&cmd=Retrieve&dopt=full_report&list_uids=7468) | nuclear receptor binding SET domain protein 2 |
| [Details](https://mirdb.org/cgi-bin/target_detail.cgi?targetID=3253915) | 1387 | 51 | hsa-miR-15a-5p | [ZNF224](http://www.ncbi.nlm.nih.gov/entrez/query.fcgi?db=gene&cmd=Retrieve&dopt=full_report&list_uids=7767) | zinc finger protein 224 |
| [Details](https://mirdb.org/cgi-bin/target_detail.cgi?targetID=3253918) | 1388 | 51 | hsa-miR-15a-5p | [PXMP4](http://www.ncbi.nlm.nih.gov/entrez/query.fcgi?db=gene&cmd=Retrieve&dopt=full_report&list_uids=11264) | peroxisomal membrane protein 4 |
| [Details](https://mirdb.org/cgi-bin/target_detail.cgi?targetID=3254017) | 1389 | 51 | hsa-miR-15a-5p | [DUSP9](http://www.ncbi.nlm.nih.gov/entrez/query.fcgi?db=gene&cmd=Retrieve&dopt=full_report&list_uids=1852) | dual specificity phosphatase 9 |
| [Details](https://mirdb.org/cgi-bin/target_detail.cgi?targetID=3254026) | 1390 | 51 | hsa-miR-15a-5p | [GTPBP1](http://www.ncbi.nlm.nih.gov/entrez/query.fcgi?db=gene&cmd=Retrieve&dopt=full_report&list_uids=9567) | GTP binding protein 1 |
| [Details](https://mirdb.org/cgi-bin/target_detail.cgi?targetID=3252708) | 1391 | 50 | hsa-miR-15a-5p | [SEC22C](http://www.ncbi.nlm.nih.gov/entrez/query.fcgi?db=gene&cmd=Retrieve&dopt=full_report&list_uids=9117) | SEC22 homolog C, vesicle trafficking protein |
| [Details](https://mirdb.org/cgi-bin/target_detail.cgi?targetID=3252833) | 1392 | 50 | hsa-miR-15a-5p | [ALDH6A1](http://www.ncbi.nlm.nih.gov/entrez/query.fcgi?db=gene&cmd=Retrieve&dopt=full_report&list_uids=4329) | aldehyde dehydrogenase 6 family member A1 |
| [Details](https://mirdb.org/cgi-bin/target_detail.cgi?targetID=3252888) | 1393 | 50 | hsa-miR-15a-5p | [CSF1](http://www.ncbi.nlm.nih.gov/entrez/query.fcgi?db=gene&cmd=Retrieve&dopt=full_report&list_uids=1435) | colony stimulating factor 1 |
| [Details](https://mirdb.org/cgi-bin/target_detail.cgi?targetID=3252911) | 1394 | 50 | hsa-miR-15a-5p | [CLASP1](http://www.ncbi.nlm.nih.gov/entrez/query.fcgi?db=gene&cmd=Retrieve&dopt=full_report&list_uids=23332) | cytoplasmic linker associated protein 1 |
| [Details](https://mirdb.org/cgi-bin/target_detail.cgi?targetID=3252945) | 1395 | 50 | hsa-miR-15a-5p | [ANKRD13D](http://www.ncbi.nlm.nih.gov/entrez/query.fcgi?db=gene&cmd=Retrieve&dopt=full_report&list_uids=338692) | ankyrin repeat domain 13D |
| [Details](https://mirdb.org/cgi-bin/target_detail.cgi?targetID=3252984) | 1396 | 50 | hsa-miR-15a-5p | [RRAGA](http://www.ncbi.nlm.nih.gov/entrez/query.fcgi?db=gene&cmd=Retrieve&dopt=full_report&list_uids=10670) | Ras related GTP binding A |
| [Details](https://mirdb.org/cgi-bin/target_detail.cgi?targetID=3252991) | 1397 | 50 | hsa-miR-15a-5p | [KHNYN](http://www.ncbi.nlm.nih.gov/entrez/query.fcgi?db=gene&cmd=Retrieve&dopt=full_report&list_uids=23351) | KH and NYN domain containing |
| [Details](https://mirdb.org/cgi-bin/target_detail.cgi?targetID=3253010) | 1398 | 50 | hsa-miR-15a-5p | [PMM1](http://www.ncbi.nlm.nih.gov/entrez/query.fcgi?db=gene&cmd=Retrieve&dopt=full_report&list_uids=5372) | phosphomannomutase 1 |
| [Details](https://mirdb.org/cgi-bin/target_detail.cgi?targetID=3253186) | 1399 | 50 | hsa-miR-15a-5p | [SENP2](http://www.ncbi.nlm.nih.gov/entrez/query.fcgi?db=gene&cmd=Retrieve&dopt=full_report&list_uids=59343) | SUMO specific peptidase 2 |
| [Details](https://mirdb.org/cgi-bin/target_detail.cgi?targetID=3253209) | 1400 | 50 | hsa-miR-15a-5p | [HAPLN4](http://www.ncbi.nlm.nih.gov/entrez/query.fcgi?db=gene&cmd=Retrieve&dopt=full_report&list_uids=404037) | hyaluronan and proteoglycan link protein 4 |
| [Details](https://mirdb.org/cgi-bin/target_detail.cgi?targetID=3253213) | 1401 | 50 | hsa-miR-15a-5p | [MMP19](http://www.ncbi.nlm.nih.gov/entrez/query.fcgi?db=gene&cmd=Retrieve&dopt=full_report&list_uids=4327) | matrix metallopeptidase 19 |
| [Details](https://mirdb.org/cgi-bin/target_detail.cgi?targetID=3253308) | 1402 | 50 | hsa-miR-15a-5p | [DYRK2](http://www.ncbi.nlm.nih.gov/entrez/query.fcgi?db=gene&cmd=Retrieve&dopt=full_report&list_uids=8445) | dual specificity tyrosine phosphorylation regulated kinase 2 |
| [Details](https://mirdb.org/cgi-bin/target_detail.cgi?targetID=3253392) | 1403 | 50 | hsa-miR-15a-5p | [TK2](http://www.ncbi.nlm.nih.gov/entrez/query.fcgi?db=gene&cmd=Retrieve&dopt=full_report&list_uids=7084) | thymidine kinase 2, mitochondrial |
| [Details](https://mirdb.org/cgi-bin/target_detail.cgi?targetID=3253420) | 1404 | 50 | hsa-miR-15a-5p | [PCNX1](http://www.ncbi.nlm.nih.gov/entrez/query.fcgi?db=gene&cmd=Retrieve&dopt=full_report&list_uids=22990) | pecanex 1 |
| [Details](https://mirdb.org/cgi-bin/target_detail.cgi?targetID=3253438) | 1405 | 50 | hsa-miR-15a-5p | [SNRPA1](http://www.ncbi.nlm.nih.gov/entrez/query.fcgi?db=gene&cmd=Retrieve&dopt=full_report&list_uids=6627) | small nuclear ribonucleoprotein polypeptide A' |
| [Details](https://mirdb.org/cgi-bin/target_detail.cgi?targetID=3253473) | 1406 | 50 | hsa-miR-15a-5p | [TRPC1](http://www.ncbi.nlm.nih.gov/entrez/query.fcgi?db=gene&cmd=Retrieve&dopt=full_report&list_uids=7220) | transient receptor potential cation channel subfamily C member 1 |
| [Details](https://mirdb.org/cgi-bin/target_detail.cgi?targetID=3253546) | 1407 | 50 | hsa-miR-15a-5p | [TM7SF3](http://www.ncbi.nlm.nih.gov/entrez/query.fcgi?db=gene&cmd=Retrieve&dopt=full_report&list_uids=51768) | transmembrane 7 superfamily member 3 |
| [Details](https://mirdb.org/cgi-bin/target_detail.cgi?targetID=3253554) | 1408 | 50 | hsa-miR-15a-5p | [CASC1](http://www.ncbi.nlm.nih.gov/entrez/query.fcgi?db=gene&cmd=Retrieve&dopt=full_report&list_uids=55259) | cancer susceptibility 1 |
| [Details](https://mirdb.org/cgi-bin/target_detail.cgi?targetID=3253561) | 1409 | 50 | hsa-miR-15a-5p | [RGS5](http://www.ncbi.nlm.nih.gov/entrez/query.fcgi?db=gene&cmd=Retrieve&dopt=full_report&list_uids=8490) | regulator of G protein signaling 5 |
| [Details](https://mirdb.org/cgi-bin/target_detail.cgi?targetID=3253637) | 1410 | 50 | hsa-miR-15a-5p | [OTUD6A](http://www.ncbi.nlm.nih.gov/entrez/query.fcgi?db=gene&cmd=Retrieve&dopt=full_report&list_uids=139562) | OTU deubiquitinase 6A |
| [Details](https://mirdb.org/cgi-bin/target_detail.cgi?targetID=3253716) | 1411 | 50 | hsa-miR-15a-5p | [TBC1D13](http://www.ncbi.nlm.nih.gov/entrez/query.fcgi?db=gene&cmd=Retrieve&dopt=full_report&list_uids=54662) | TBC1 domain family member 13 |
| [Details](https://mirdb.org/cgi-bin/target_detail.cgi?targetID=3253814) | 1412 | 50 | hsa-miR-15a-5p | [ZHX1](http://www.ncbi.nlm.nih.gov/entrez/query.fcgi?db=gene&cmd=Retrieve&dopt=full_report&list_uids=11244) | zinc fingers and homeoboxes 1 |
| [Details](https://mirdb.org/cgi-bin/target_detail.cgi?targetID=3253956) | 1413 | 50 | hsa-miR-15a-5p | [CENPN](http://www.ncbi.nlm.nih.gov/entrez/query.fcgi?db=gene&cmd=Retrieve&dopt=full_report&list_uids=55839) | centromere protein N |
| [Details](https://mirdb.org/cgi-bin/target_detail.cgi?targetID=3253969) | 1414 | 50 | hsa-miR-15a-5p | [OGFOD3](http://www.ncbi.nlm.nih.gov/entrez/query.fcgi?db=gene&cmd=Retrieve&dopt=full_report&list_uids=79701) | 2-oxoglutarate and iron dependent oxygenase domain containing 3 |
| [Details](https://mirdb.org/cgi-bin/target_detail.cgi?targetID=3254033) | 1415 | 50 | hsa-miR-15a-5p | [TEAD1](http://www.ncbi.nlm.nih.gov/entrez/query.fcgi?db=gene&cmd=Retrieve&dopt=full_report&list_uids=7003) | TEA domain transcription factor 1 |
