# Supplementary material for: Integrated analysis of miR-15a-5p, miR-20a-5p, and miR-33b-3p identifies EGR2-associated biomarkers in multiple myeloma
Source: BMC Cancer. 2026 Feb 12;26:298. doi: 10.1186/s12885-026-15610-5 (PMC12930744; doi:10.1186/s12885-026-15610-5)
Supplement: Supplementary file 2 — Supplementary Material 2. [file 12885_2026_15610_MOESM2_ESM.docx]

**Supplementary table (2):** Predicted targets for hsa-miR-20a-5p.

| **Target Detail** | **Target Rank** | **Target Score** | **miRNA Name** | **Gene Symbol** | **Gene Description** |
| --- | --- | --- | --- | --- | --- |
| [Details](https://mirdb.org/cgi-bin/target_detail.cgi?targetID=2854536) | 1 | 100 | hsa-miR-20a-5p | [ANKRD52](http://www.ncbi.nlm.nih.gov/entrez/query.fcgi?db=gene&cmd=Retrieve&dopt=full_report&list_uids=283373) | ankyrin repeat domain 52 |
| [Details](https://mirdb.org/cgi-bin/target_detail.cgi?targetID=2854768) | 2 | 100 | hsa-miR-20a-5p | [ZFYVE26](http://www.ncbi.nlm.nih.gov/entrez/query.fcgi?db=gene&cmd=Retrieve&dopt=full_report&list_uids=23503) | zinc finger FYVE-type containing 26 |
| [Details](https://mirdb.org/cgi-bin/target_detail.cgi?targetID=2854791) | 3 | 100 | hsa-miR-20a-5p | [ENPP5](http://www.ncbi.nlm.nih.gov/entrez/query.fcgi?db=gene&cmd=Retrieve&dopt=full_report&list_uids=59084) | ectonucleotide pyrophosphatase/phosphodiesterase 5 (putative) |
| [Details](https://mirdb.org/cgi-bin/target_detail.cgi?targetID=2854904) | 4 | 100 | hsa-miR-20a-5p | [GPR137C](http://www.ncbi.nlm.nih.gov/entrez/query.fcgi?db=gene&cmd=Retrieve&dopt=full_report&list_uids=283554) | G protein-coupled receptor 137C |
| [Details](https://mirdb.org/cgi-bin/target_detail.cgi?targetID=2854919) | 5 | 100 | hsa-miR-20a-5p | [FYCO1](http://www.ncbi.nlm.nih.gov/entrez/query.fcgi?db=gene&cmd=Retrieve&dopt=full_report&list_uids=79443) | FYVE and coiled-coil domain containing 1 |
| [Details](https://mirdb.org/cgi-bin/target_detail.cgi?targetID=2855244) | 6 | 100 | hsa-miR-20a-5p | [DYNC1LI2](http://www.ncbi.nlm.nih.gov/entrez/query.fcgi?db=gene&cmd=Retrieve&dopt=full_report&list_uids=1783) | dynein cytoplasmic 1 light intermediate chain 2 |
| [Details](https://mirdb.org/cgi-bin/target_detail.cgi?targetID=2855362) | 7 | 100 | hsa-miR-20a-5p | [ZNFX1](http://www.ncbi.nlm.nih.gov/entrez/query.fcgi?db=gene&cmd=Retrieve&dopt=full_report&list_uids=57169) | zinc finger NFX1-type containing 1 |
| [Details](https://mirdb.org/cgi-bin/target_detail.cgi?targetID=2855383) | 8 | 100 | hsa-miR-20a-5p | [PKD2](http://www.ncbi.nlm.nih.gov/entrez/query.fcgi?db=gene&cmd=Retrieve&dopt=full_report&list_uids=5311) | polycystin 2, transient receptor potential cation channel |
| [Details](https://mirdb.org/cgi-bin/target_detail.cgi?targetID=2855456) | 9 | 100 | hsa-miR-20a-5p | [NAPEPLD](http://www.ncbi.nlm.nih.gov/entrez/query.fcgi?db=gene&cmd=Retrieve&dopt=full_report&list_uids=222236) | N-acyl phosphatidylethanolamine phospholipase D |
| [Details](https://mirdb.org/cgi-bin/target_detail.cgi?targetID=2854080) | 10 | 99 | hsa-miR-20a-5p | [NPAT](http://www.ncbi.nlm.nih.gov/entrez/query.fcgi?db=gene&cmd=Retrieve&dopt=full_report&list_uids=4863) | nuclear protein, coactivator of histone transcription |
| [Details](https://mirdb.org/cgi-bin/target_detail.cgi?targetID=2854094) | 11 | 99 | hsa-miR-20a-5p | [ARID4B](http://www.ncbi.nlm.nih.gov/entrez/query.fcgi?db=gene&cmd=Retrieve&dopt=full_report&list_uids=51742) | AT-rich interaction domain 4B |
| [Details](https://mirdb.org/cgi-bin/target_detail.cgi?targetID=2854124) | 12 | 99 | hsa-miR-20a-5p | [MED12L](http://www.ncbi.nlm.nih.gov/entrez/query.fcgi?db=gene&cmd=Retrieve&dopt=full_report&list_uids=116931) | mediator complex subunit 12 like |
| [Details](https://mirdb.org/cgi-bin/target_detail.cgi?targetID=2854142) | 13 | 99 | hsa-miR-20a-5p | [TBC1D20](http://www.ncbi.nlm.nih.gov/entrez/query.fcgi?db=gene&cmd=Retrieve&dopt=full_report&list_uids=128637) | TBC1 domain family member 20 |
| [Details](https://mirdb.org/cgi-bin/target_detail.cgi?targetID=2854183) | 14 | 99 | hsa-miR-20a-5p | [ZNF800](http://www.ncbi.nlm.nih.gov/entrez/query.fcgi?db=gene&cmd=Retrieve&dopt=full_report&list_uids=168850) | zinc finger protein 800 |
| [Details](https://mirdb.org/cgi-bin/target_detail.cgi?targetID=2854280) | 15 | 99 | hsa-miR-20a-5p | [NPAS2](http://www.ncbi.nlm.nih.gov/entrez/query.fcgi?db=gene&cmd=Retrieve&dopt=full_report&list_uids=4862) | neuronal PAS domain protein 2 |
| [Details](https://mirdb.org/cgi-bin/target_detail.cgi?targetID=2854331) | 16 | 99 | hsa-miR-20a-5p | [FAM45A](http://www.ncbi.nlm.nih.gov/entrez/query.fcgi?db=gene&cmd=Retrieve&dopt=full_report&list_uids=404636) | family with sequence similarity 45 member A |
| [Details](https://mirdb.org/cgi-bin/target_detail.cgi?targetID=2854398) | 17 | 99 | hsa-miR-20a-5p | [SAR1B](http://www.ncbi.nlm.nih.gov/entrez/query.fcgi?db=gene&cmd=Retrieve&dopt=full_report&list_uids=51128) | secretion associated Ras related GTPase 1B |
| [Details](https://mirdb.org/cgi-bin/target_detail.cgi?targetID=2854414) | 18 | 99 | hsa-miR-20a-5p | [BRMS1L](http://www.ncbi.nlm.nih.gov/entrez/query.fcgi?db=gene&cmd=Retrieve&dopt=full_report&list_uids=84312) | BRMS1 like transcriptional repressor |
| [Details](https://mirdb.org/cgi-bin/target_detail.cgi?targetID=2854442) | 19 | 99 | hsa-miR-20a-5p | [GPR6](http://www.ncbi.nlm.nih.gov/entrez/query.fcgi?db=gene&cmd=Retrieve&dopt=full_report&list_uids=2830) | G protein-coupled receptor 6 |
| [Details](https://mirdb.org/cgi-bin/target_detail.cgi?targetID=2854472) | 20 | 99 | hsa-miR-20a-5p | [ITGB8](http://www.ncbi.nlm.nih.gov/entrez/query.fcgi?db=gene&cmd=Retrieve&dopt=full_report&list_uids=3696) | integrin subunit beta 8 |
| [Details](https://mirdb.org/cgi-bin/target_detail.cgi?targetID=2854538) | 21 | 99 | hsa-miR-20a-5p | [VLDLR](http://www.ncbi.nlm.nih.gov/entrez/query.fcgi?db=gene&cmd=Retrieve&dopt=full_report&list_uids=7436) | very low density lipoprotein receptor |
| [Details](https://mirdb.org/cgi-bin/target_detail.cgi?targetID=2854549) | 22 | 99 | hsa-miR-20a-5p | [TXNIP](http://www.ncbi.nlm.nih.gov/entrez/query.fcgi?db=gene&cmd=Retrieve&dopt=full_report&list_uids=10628) | thioredoxin interacting protein |
| [Details](https://mirdb.org/cgi-bin/target_detail.cgi?targetID=2854569) | 23 | 99 | hsa-miR-20a-5p | [KCNB1](http://www.ncbi.nlm.nih.gov/entrez/query.fcgi?db=gene&cmd=Retrieve&dopt=full_report&list_uids=3745) | potassium voltage-gated channel subfamily B member 1 |
| [Details](https://mirdb.org/cgi-bin/target_detail.cgi?targetID=2854638) | 24 | 99 | hsa-miR-20a-5p | [CLOCK](http://www.ncbi.nlm.nih.gov/entrez/query.fcgi?db=gene&cmd=Retrieve&dopt=full_report&list_uids=9575) | clock circadian regulator |
| [Details](https://mirdb.org/cgi-bin/target_detail.cgi?targetID=2854655) | 25 | 99 | hsa-miR-20a-5p | [RUFY2](http://www.ncbi.nlm.nih.gov/entrez/query.fcgi?db=gene&cmd=Retrieve&dopt=full_report&list_uids=55680) | RUN and FYVE domain containing 2 |
| [Details](https://mirdb.org/cgi-bin/target_detail.cgi?targetID=2854667) | 26 | 99 | hsa-miR-20a-5p | [ARHGAP12](http://www.ncbi.nlm.nih.gov/entrez/query.fcgi?db=gene&cmd=Retrieve&dopt=full_report&list_uids=94134) | Rho GTPase activating protein 12 |
| [Details](https://mirdb.org/cgi-bin/target_detail.cgi?targetID=2854732) | 27 | 99 | hsa-miR-20a-5p | [KCNK10](http://www.ncbi.nlm.nih.gov/entrez/query.fcgi?db=gene&cmd=Retrieve&dopt=full_report&list_uids=54207) | potassium two pore domain channel subfamily K member 10 |
| [Details](https://mirdb.org/cgi-bin/target_detail.cgi?targetID=2854735) | 28 | 99 | hsa-miR-20a-5p | [STK17B](http://www.ncbi.nlm.nih.gov/entrez/query.fcgi?db=gene&cmd=Retrieve&dopt=full_report&list_uids=9262) | serine/threonine kinase 17b |
| [Details](https://mirdb.org/cgi-bin/target_detail.cgi?targetID=2854741) | 29 | 99 | hsa-miR-20a-5p | [PDCD1LG2](http://www.ncbi.nlm.nih.gov/entrez/query.fcgi?db=gene&cmd=Retrieve&dopt=full_report&list_uids=80380) | programmed cell death 1 ligand 2 |
| [Details](https://mirdb.org/cgi-bin/target_detail.cgi?targetID=2854778) | 30 | 99 | hsa-miR-20a-5p | [RAB22A](http://www.ncbi.nlm.nih.gov/entrez/query.fcgi?db=gene&cmd=Retrieve&dopt=full_report&list_uids=57403) | RAB22A, member RAS oncogene family |
| [Details](https://mirdb.org/cgi-bin/target_detail.cgi?targetID=2854784) | 31 | 99 | hsa-miR-20a-5p | [USP46](http://www.ncbi.nlm.nih.gov/entrez/query.fcgi?db=gene&cmd=Retrieve&dopt=full_report&list_uids=64854) | ubiquitin specific peptidase 46 |
| [Details](https://mirdb.org/cgi-bin/target_detail.cgi?targetID=2854851) | 32 | 99 | hsa-miR-20a-5p | [C2CD2](http://www.ncbi.nlm.nih.gov/entrez/query.fcgi?db=gene&cmd=Retrieve&dopt=full_report&list_uids=25966) | C2 calcium dependent domain containing 2 |
| [Details](https://mirdb.org/cgi-bin/target_detail.cgi?targetID=2854901) | 33 | 99 | hsa-miR-20a-5p | [SLC40A1](http://www.ncbi.nlm.nih.gov/entrez/query.fcgi?db=gene&cmd=Retrieve&dopt=full_report&list_uids=30061) | solute carrier family 40 member 1 |
| [Details](https://mirdb.org/cgi-bin/target_detail.cgi?targetID=2854967) | 34 | 99 | hsa-miR-20a-5p | [EZH1](http://www.ncbi.nlm.nih.gov/entrez/query.fcgi?db=gene&cmd=Retrieve&dopt=full_report&list_uids=2145) | enhancer of zeste 1 polycomb repressive complex 2 subunit |
| [Details](https://mirdb.org/cgi-bin/target_detail.cgi?targetID=2855099) | 35 | 99 | hsa-miR-20a-5p | [ITPRIPL2](http://www.ncbi.nlm.nih.gov/entrez/query.fcgi?db=gene&cmd=Retrieve&dopt=full_report&list_uids=162073) | ITPRIP like 2 |
| [Details](https://mirdb.org/cgi-bin/target_detail.cgi?targetID=2855144) | 36 | 99 | hsa-miR-20a-5p | [REEP3](http://www.ncbi.nlm.nih.gov/entrez/query.fcgi?db=gene&cmd=Retrieve&dopt=full_report&list_uids=221035) | receptor accessory protein 3 |
| [Details](https://mirdb.org/cgi-bin/target_detail.cgi?targetID=2855183) | 37 | 99 | hsa-miR-20a-5p | [RRAGD](http://www.ncbi.nlm.nih.gov/entrez/query.fcgi?db=gene&cmd=Retrieve&dopt=full_report&list_uids=58528) | Ras related GTP binding D |
| [Details](https://mirdb.org/cgi-bin/target_detail.cgi?targetID=2855254) | 38 | 99 | hsa-miR-20a-5p | [TBC1D9](http://www.ncbi.nlm.nih.gov/entrez/query.fcgi?db=gene&cmd=Retrieve&dopt=full_report&list_uids=23158) | TBC1 domain family member 9 |
| [Details](https://mirdb.org/cgi-bin/target_detail.cgi?targetID=2855316) | 39 | 99 | hsa-miR-20a-5p | [PLEKHA3](http://www.ncbi.nlm.nih.gov/entrez/query.fcgi?db=gene&cmd=Retrieve&dopt=full_report&list_uids=65977) | pleckstrin homology domain containing A3 |
| [Details](https://mirdb.org/cgi-bin/target_detail.cgi?targetID=2855347) | 40 | 99 | hsa-miR-20a-5p | [CFL2](http://www.ncbi.nlm.nih.gov/entrez/query.fcgi?db=gene&cmd=Retrieve&dopt=full_report&list_uids=1073) | cofilin 2 |
| [Details](https://mirdb.org/cgi-bin/target_detail.cgi?targetID=2855348) | 41 | 99 | hsa-miR-20a-5p | [PTPN4](http://www.ncbi.nlm.nih.gov/entrez/query.fcgi?db=gene&cmd=Retrieve&dopt=full_report&list_uids=5775) | protein tyrosine phosphatase, non-receptor type 4 |
| [Details](https://mirdb.org/cgi-bin/target_detail.cgi?targetID=2855350) | 42 | 99 | hsa-miR-20a-5p | [AAK1](http://www.ncbi.nlm.nih.gov/entrez/query.fcgi?db=gene&cmd=Retrieve&dopt=full_report&list_uids=22848) | AP2 associated kinase 1 |
| [Details](https://mirdb.org/cgi-bin/target_detail.cgi?targetID=2855379) | 43 | 99 | hsa-miR-20a-5p | [SACS](http://www.ncbi.nlm.nih.gov/entrez/query.fcgi?db=gene&cmd=Retrieve&dopt=full_report&list_uids=26278) | sacsin molecular chaperone |
| [Details](https://mirdb.org/cgi-bin/target_detail.cgi?targetID=2855409) | 44 | 99 | hsa-miR-20a-5p | [ZNF827](http://www.ncbi.nlm.nih.gov/entrez/query.fcgi?db=gene&cmd=Retrieve&dopt=full_report&list_uids=152485) | zinc finger protein 827 |
| [Details](https://mirdb.org/cgi-bin/target_detail.cgi?targetID=2855427) | 45 | 99 | hsa-miR-20a-5p | [MAP3K2](http://www.ncbi.nlm.nih.gov/entrez/query.fcgi?db=gene&cmd=Retrieve&dopt=full_report&list_uids=10746) | mitogen-activated protein kinase kinase kinase 2 |
| [Details](https://mirdb.org/cgi-bin/target_detail.cgi?targetID=2854108) | 46 | 98 | hsa-miR-20a-5p | [FBXL5](http://www.ncbi.nlm.nih.gov/entrez/query.fcgi?db=gene&cmd=Retrieve&dopt=full_report&list_uids=26234) | F-box and leucine rich repeat protein 5 |
| [Details](https://mirdb.org/cgi-bin/target_detail.cgi?targetID=2854277) | 47 | 98 | hsa-miR-20a-5p | [NCOA3](http://www.ncbi.nlm.nih.gov/entrez/query.fcgi?db=gene&cmd=Retrieve&dopt=full_report&list_uids=8202) | nuclear receptor coactivator 3 |
| [Details](https://mirdb.org/cgi-bin/target_detail.cgi?targetID=2854308) | 48 | 98 | hsa-miR-20a-5p | [EPHA4](http://www.ncbi.nlm.nih.gov/entrez/query.fcgi?db=gene&cmd=Retrieve&dopt=full_report&list_uids=2043) | EPH receptor A4 |
| [Details](https://mirdb.org/cgi-bin/target_detail.cgi?targetID=2854349) | 49 | 98 | hsa-miR-20a-5p | [EPHA5](http://www.ncbi.nlm.nih.gov/entrez/query.fcgi?db=gene&cmd=Retrieve&dopt=full_report&list_uids=2044) | EPH receptor A5 |
| [Details](https://mirdb.org/cgi-bin/target_detail.cgi?targetID=2854431) | 50 | 98 | hsa-miR-20a-5p | [FRMD6](http://www.ncbi.nlm.nih.gov/entrez/query.fcgi?db=gene&cmd=Retrieve&dopt=full_report&list_uids=122786) | FERM domain containing 6 |
| [Details](https://mirdb.org/cgi-bin/target_detail.cgi?targetID=2854518) | 51 | 98 | hsa-miR-20a-5p | [E2F1](http://www.ncbi.nlm.nih.gov/entrez/query.fcgi?db=gene&cmd=Retrieve&dopt=full_report&list_uids=1869) | E2F transcription factor 1 |
| [Details](https://mirdb.org/cgi-bin/target_detail.cgi?targetID=2854523) | 52 | 98 | hsa-miR-20a-5p | [VANGL1](http://www.ncbi.nlm.nih.gov/entrez/query.fcgi?db=gene&cmd=Retrieve&dopt=full_report&list_uids=81839) | VANGL planar cell polarity protein 1 |
| [Details](https://mirdb.org/cgi-bin/target_detail.cgi?targetID=2854560) | 53 | 98 | hsa-miR-20a-5p | [RPS6KA5](http://www.ncbi.nlm.nih.gov/entrez/query.fcgi?db=gene&cmd=Retrieve&dopt=full_report&list_uids=9252) | ribosomal protein S6 kinase A5 |
| [Details](https://mirdb.org/cgi-bin/target_detail.cgi?targetID=2854629) | 54 | 98 | hsa-miR-20a-5p | [CAMTA1](http://www.ncbi.nlm.nih.gov/entrez/query.fcgi?db=gene&cmd=Retrieve&dopt=full_report&list_uids=23261) | calmodulin binding transcription activator 1 |
| [Details](https://mirdb.org/cgi-bin/target_detail.cgi?targetID=2854669) | 55 | 98 | hsa-miR-20a-5p | [AKTIP](http://www.ncbi.nlm.nih.gov/entrez/query.fcgi?db=gene&cmd=Retrieve&dopt=full_report&list_uids=64400) | AKT interacting protein |
| [Details](https://mirdb.org/cgi-bin/target_detail.cgi?targetID=2854750) | 56 | 98 | hsa-miR-20a-5p | [FCHO2](http://www.ncbi.nlm.nih.gov/entrez/query.fcgi?db=gene&cmd=Retrieve&dopt=full_report&list_uids=115548) | FCH domain only 2 |
| [Details](https://mirdb.org/cgi-bin/target_detail.cgi?targetID=2854801) | 57 | 98 | hsa-miR-20a-5p | [NFAT5](http://www.ncbi.nlm.nih.gov/entrez/query.fcgi?db=gene&cmd=Retrieve&dopt=full_report&list_uids=10725) | nuclear factor of activated T cells 5 |
| [Details](https://mirdb.org/cgi-bin/target_detail.cgi?targetID=2854877) | 58 | 98 | hsa-miR-20a-5p | [ZBTB4](http://www.ncbi.nlm.nih.gov/entrez/query.fcgi?db=gene&cmd=Retrieve&dopt=full_report&list_uids=57659) | zinc finger and BTB domain containing 4 |
| [Details](https://mirdb.org/cgi-bin/target_detail.cgi?targetID=2854898) | 59 | 98 | hsa-miR-20a-5p | [RNF128](http://www.ncbi.nlm.nih.gov/entrez/query.fcgi?db=gene&cmd=Retrieve&dopt=full_report&list_uids=79589) | ring finger protein 128, E3 ubiquitin protein ligase |
| [Details](https://mirdb.org/cgi-bin/target_detail.cgi?targetID=2854939) | 60 | 98 | hsa-miR-20a-5p | [SAMD12](http://www.ncbi.nlm.nih.gov/entrez/query.fcgi?db=gene&cmd=Retrieve&dopt=full_report&list_uids=401474) | sterile alpha motif domain containing 12 |
| [Details](https://mirdb.org/cgi-bin/target_detail.cgi?targetID=2854960) | 61 | 98 | hsa-miR-20a-5p | [HSPA8](http://www.ncbi.nlm.nih.gov/entrez/query.fcgi?db=gene&cmd=Retrieve&dopt=full_report&list_uids=3312) | heat shock protein family A (Hsp70) member 8 |
| [Details](https://mirdb.org/cgi-bin/target_detail.cgi?targetID=2854998) | 62 | 98 | hsa-miR-20a-5p | [RGL1](http://www.ncbi.nlm.nih.gov/entrez/query.fcgi?db=gene&cmd=Retrieve&dopt=full_report&list_uids=23179) | ral guanine nucleotide dissociation stimulator like 1 |
| [Details](https://mirdb.org/cgi-bin/target_detail.cgi?targetID=2855081) | 63 | 98 | hsa-miR-20a-5p | [MKRN1](http://www.ncbi.nlm.nih.gov/entrez/query.fcgi?db=gene&cmd=Retrieve&dopt=full_report&list_uids=23608) | makorin ring finger protein 1 |
| [Details](https://mirdb.org/cgi-bin/target_detail.cgi?targetID=2855105) | 64 | 98 | hsa-miR-20a-5p | [EMSY](http://www.ncbi.nlm.nih.gov/entrez/query.fcgi?db=gene&cmd=Retrieve&dopt=full_report&list_uids=56946) | EMSY, BRCA2 interacting transcriptional repressor |
| [Details](https://mirdb.org/cgi-bin/target_detail.cgi?targetID=2855177) | 65 | 98 | hsa-miR-20a-5p | [ZNF367](http://www.ncbi.nlm.nih.gov/entrez/query.fcgi?db=gene&cmd=Retrieve&dopt=full_report&list_uids=195828) | zinc finger protein 367 |
| [Details](https://mirdb.org/cgi-bin/target_detail.cgi?targetID=2855318) | 66 | 98 | hsa-miR-20a-5p | [ZNF512B](http://www.ncbi.nlm.nih.gov/entrez/query.fcgi?db=gene&cmd=Retrieve&dopt=full_report&list_uids=57473) | zinc finger protein 512B |
| [Details](https://mirdb.org/cgi-bin/target_detail.cgi?targetID=2855322) | 67 | 98 | hsa-miR-20a-5p | [ANKIB1](http://www.ncbi.nlm.nih.gov/entrez/query.fcgi?db=gene&cmd=Retrieve&dopt=full_report&list_uids=54467) | ankyrin repeat and IBR domain containing 1 |
| [Details](https://mirdb.org/cgi-bin/target_detail.cgi?targetID=2855326) | 68 | 98 | hsa-miR-20a-5p | [REST](http://www.ncbi.nlm.nih.gov/entrez/query.fcgi?db=gene&cmd=Retrieve&dopt=full_report&list_uids=5978) | RE1 silencing transcription factor |
| [Details](https://mirdb.org/cgi-bin/target_detail.cgi?targetID=2855345) | 69 | 98 | hsa-miR-20a-5p | [KLHL28](http://www.ncbi.nlm.nih.gov/entrez/query.fcgi?db=gene&cmd=Retrieve&dopt=full_report&list_uids=54813) | kelch like family member 28 |
| [Details](https://mirdb.org/cgi-bin/target_detail.cgi?targetID=2855459) | 70 | 98 | hsa-miR-20a-5p | [KIAA0513](http://www.ncbi.nlm.nih.gov/entrez/query.fcgi?db=gene&cmd=Retrieve&dopt=full_report&list_uids=9764) | KIAA0513 |
| [Details](https://mirdb.org/cgi-bin/target_detail.cgi?targetID=2854085) | 71 | 97 | hsa-miR-20a-5p | [VASH2](http://www.ncbi.nlm.nih.gov/entrez/query.fcgi?db=gene&cmd=Retrieve&dopt=full_report&list_uids=79805) | vasohibin 2 |
| [Details](https://mirdb.org/cgi-bin/target_detail.cgi?targetID=2854226) | 72 | 97 | hsa-miR-20a-5p | [GPR63](http://www.ncbi.nlm.nih.gov/entrez/query.fcgi?db=gene&cmd=Retrieve&dopt=full_report&list_uids=81491) | G protein-coupled receptor 63 |
| [Details](https://mirdb.org/cgi-bin/target_detail.cgi?targetID=2854281) | 73 | 97 | hsa-miR-20a-5p | [MFSD8](http://www.ncbi.nlm.nih.gov/entrez/query.fcgi?db=gene&cmd=Retrieve&dopt=full_report&list_uids=256471) | major facilitator superfamily domain containing 8 |
| [Details](https://mirdb.org/cgi-bin/target_detail.cgi?targetID=2854283) | 74 | 97 | hsa-miR-20a-5p | [CCND1](http://www.ncbi.nlm.nih.gov/entrez/query.fcgi?db=gene&cmd=Retrieve&dopt=full_report&list_uids=595) | cyclin D1 |
| [Details](https://mirdb.org/cgi-bin/target_detail.cgi?targetID=2854344) | 75 | 97 | hsa-miR-20a-5p | [USP3](http://www.ncbi.nlm.nih.gov/entrez/query.fcgi?db=gene&cmd=Retrieve&dopt=full_report&list_uids=9960) | ubiquitin specific peptidase 3 |
| [Details](https://mirdb.org/cgi-bin/target_detail.cgi?targetID=2854380) | 76 | 97 | hsa-miR-20a-5p | [TRIP10](http://www.ncbi.nlm.nih.gov/entrez/query.fcgi?db=gene&cmd=Retrieve&dopt=full_report&list_uids=9322) | thyroid hormone receptor interactor 10 |
| [Details](https://mirdb.org/cgi-bin/target_detail.cgi?targetID=2854391) | 77 | 97 | hsa-miR-20a-5p | [MYT1L](http://www.ncbi.nlm.nih.gov/entrez/query.fcgi?db=gene&cmd=Retrieve&dopt=full_report&list_uids=23040) | myelin transcription factor 1 like |
| [Details](https://mirdb.org/cgi-bin/target_detail.cgi?targetID=2854503) | 78 | 97 | hsa-miR-20a-5p | [BNIP2](http://www.ncbi.nlm.nih.gov/entrez/query.fcgi?db=gene&cmd=Retrieve&dopt=full_report&list_uids=663) | BCL2 interacting protein 2 |
| [Details](https://mirdb.org/cgi-bin/target_detail.cgi?targetID=2854522) | 79 | 97 | hsa-miR-20a-5p | [GXYLT1](http://www.ncbi.nlm.nih.gov/entrez/query.fcgi?db=gene&cmd=Retrieve&dopt=full_report&list_uids=283464) | glucoside xylosyltransferase 1 |
| [Details](https://mirdb.org/cgi-bin/target_detail.cgi?targetID=2854548) | 80 | 97 | hsa-miR-20a-5p | [ZFYVE9](http://www.ncbi.nlm.nih.gov/entrez/query.fcgi?db=gene&cmd=Retrieve&dopt=full_report&list_uids=9372) | zinc finger FYVE-type containing 9 |
| [Details](https://mirdb.org/cgi-bin/target_detail.cgi?targetID=2854573) | 81 | 97 | hsa-miR-20a-5p | [ATXN1L](http://www.ncbi.nlm.nih.gov/entrez/query.fcgi?db=gene&cmd=Retrieve&dopt=full_report&list_uids=342371) | ataxin 1 like |
| [Details](https://mirdb.org/cgi-bin/target_detail.cgi?targetID=2854648) | 82 | 97 | hsa-miR-20a-5p | [ATG16L1](http://www.ncbi.nlm.nih.gov/entrez/query.fcgi?db=gene&cmd=Retrieve&dopt=full_report&list_uids=55054) | autophagy related 16 like 1 |
| [Details](https://mirdb.org/cgi-bin/target_detail.cgi?targetID=2854697) | 83 | 97 | hsa-miR-20a-5p | [ZXDA](http://www.ncbi.nlm.nih.gov/entrez/query.fcgi?db=gene&cmd=Retrieve&dopt=full_report&list_uids=7789) | zinc finger X-linked duplicated A |
| [Details](https://mirdb.org/cgi-bin/target_detail.cgi?targetID=2854753) | 84 | 97 | hsa-miR-20a-5p | [FGD4](http://www.ncbi.nlm.nih.gov/entrez/query.fcgi?db=gene&cmd=Retrieve&dopt=full_report&list_uids=121512) | FYVE, RhoGEF and PH domain containing 4 |
| [Details](https://mirdb.org/cgi-bin/target_detail.cgi?targetID=2854807) | 85 | 97 | hsa-miR-20a-5p | [ITGA4](http://www.ncbi.nlm.nih.gov/entrez/query.fcgi?db=gene&cmd=Retrieve&dopt=full_report&list_uids=3676) | integrin subunit alpha 4 |
| [Details](https://mirdb.org/cgi-bin/target_detail.cgi?targetID=2854812) | 86 | 97 | hsa-miR-20a-5p | [FAM129A](http://www.ncbi.nlm.nih.gov/entrez/query.fcgi?db=gene&cmd=Retrieve&dopt=full_report&list_uids=116496) | family with sequence similarity 129 member A |
| [Details](https://mirdb.org/cgi-bin/target_detail.cgi?targetID=2854869) | 87 | 97 | hsa-miR-20a-5p | [LRIG1](http://www.ncbi.nlm.nih.gov/entrez/query.fcgi?db=gene&cmd=Retrieve&dopt=full_report&list_uids=26018) | leucine rich repeats and immunoglobulin like domains 1 |
| [Details](https://mirdb.org/cgi-bin/target_detail.cgi?targetID=2854994) | 88 | 97 | hsa-miR-20a-5p | [EIF5A2](http://www.ncbi.nlm.nih.gov/entrez/query.fcgi?db=gene&cmd=Retrieve&dopt=full_report&list_uids=56648) | eukaryotic translation initiation factor 5A2 |
| [Details](https://mirdb.org/cgi-bin/target_detail.cgi?targetID=2855058) | 89 | 97 | hsa-miR-20a-5p | [RASGRF2](http://www.ncbi.nlm.nih.gov/entrez/query.fcgi?db=gene&cmd=Retrieve&dopt=full_report&list_uids=5924) | Ras protein specific guanine nucleotide releasing factor 2 |
| [Details](https://mirdb.org/cgi-bin/target_detail.cgi?targetID=2855074) | 90 | 97 | hsa-miR-20a-5p | [ELK4](http://www.ncbi.nlm.nih.gov/entrez/query.fcgi?db=gene&cmd=Retrieve&dopt=full_report&list_uids=2005) | ELK4, ETS transcription factor |
| [Details](https://mirdb.org/cgi-bin/target_detail.cgi?targetID=2855098) | 91 | 97 | hsa-miR-20a-5p | [SLC24A2](http://www.ncbi.nlm.nih.gov/entrez/query.fcgi?db=gene&cmd=Retrieve&dopt=full_report&list_uids=25769) | solute carrier family 24 member 2 |
| [Details](https://mirdb.org/cgi-bin/target_detail.cgi?targetID=2855106) | 92 | 97 | hsa-miR-20a-5p | [BCL11B](http://www.ncbi.nlm.nih.gov/entrez/query.fcgi?db=gene&cmd=Retrieve&dopt=full_report&list_uids=64919) | BCL11B, BAF complex component |
| [Details](https://mirdb.org/cgi-bin/target_detail.cgi?targetID=2855140) | 93 | 97 | hsa-miR-20a-5p | [FOXJ3](http://www.ncbi.nlm.nih.gov/entrez/query.fcgi?db=gene&cmd=Retrieve&dopt=full_report&list_uids=22887) | forkhead box J3 |
| [Details](https://mirdb.org/cgi-bin/target_detail.cgi?targetID=2855155) | 94 | 97 | hsa-miR-20a-5p | [NABP1](http://www.ncbi.nlm.nih.gov/entrez/query.fcgi?db=gene&cmd=Retrieve&dopt=full_report&list_uids=64859) | nucleic acid binding protein 1 |
| [Details](https://mirdb.org/cgi-bin/target_detail.cgi?targetID=2855217) | 95 | 97 | hsa-miR-20a-5p | [FRS2](http://www.ncbi.nlm.nih.gov/entrez/query.fcgi?db=gene&cmd=Retrieve&dopt=full_report&list_uids=10818) | fibroblast growth factor receptor substrate 2 |
| [Details](https://mirdb.org/cgi-bin/target_detail.cgi?targetID=2855239) | 96 | 97 | hsa-miR-20a-5p | [EFCAB14](http://www.ncbi.nlm.nih.gov/entrez/query.fcgi?db=gene&cmd=Retrieve&dopt=full_report&list_uids=9813) | EF-hand calcium binding domain 14 |
| [Details](https://mirdb.org/cgi-bin/target_detail.cgi?targetID=2855270) | 97 | 97 | hsa-miR-20a-5p | [NR2C2](http://www.ncbi.nlm.nih.gov/entrez/query.fcgi?db=gene&cmd=Retrieve&dopt=full_report&list_uids=7182) | nuclear receptor subfamily 2 group C member 2 |
| [Details](https://mirdb.org/cgi-bin/target_detail.cgi?targetID=2855281) | 98 | 97 | hsa-miR-20a-5p | [GLIS3](http://www.ncbi.nlm.nih.gov/entrez/query.fcgi?db=gene&cmd=Retrieve&dopt=full_report&list_uids=169792) | GLIS family zinc finger 3 |
| [Details](https://mirdb.org/cgi-bin/target_detail.cgi?targetID=2855289) | 99 | 97 | hsa-miR-20a-5p | [KMT2B](http://www.ncbi.nlm.nih.gov/entrez/query.fcgi?db=gene&cmd=Retrieve&dopt=full_report&list_uids=9757) | lysine methyltransferase 2B |
| [Details](https://mirdb.org/cgi-bin/target_detail.cgi?targetID=2855370) | 100 | 97 | hsa-miR-20a-5p | [FSD1L](http://www.ncbi.nlm.nih.gov/entrez/query.fcgi?db=gene&cmd=Retrieve&dopt=full_report&list_uids=83856) | fibronectin type III and SPRY domain containing 1 like |
| [Details](https://mirdb.org/cgi-bin/target_detail.cgi?targetID=2854206) | 101 | 96 | hsa-miR-20a-5p | [RBL2](http://www.ncbi.nlm.nih.gov/entrez/query.fcgi?db=gene&cmd=Retrieve&dopt=full_report&list_uids=5934) | RB transcriptional corepressor like 2 |
| [Details](https://mirdb.org/cgi-bin/target_detail.cgi?targetID=2854223) | 102 | 96 | hsa-miR-20a-5p | [ZBTB41](http://www.ncbi.nlm.nih.gov/entrez/query.fcgi?db=gene&cmd=Retrieve&dopt=full_report&list_uids=360023) | zinc finger and BTB domain containing 41 |
| [Details](https://mirdb.org/cgi-bin/target_detail.cgi?targetID=2854224) | 103 | 96 | hsa-miR-20a-5p | [U2SURP](http://www.ncbi.nlm.nih.gov/entrez/query.fcgi?db=gene&cmd=Retrieve&dopt=full_report&list_uids=23350) | U2 snRNP associated SURP domain containing |
| [Details](https://mirdb.org/cgi-bin/target_detail.cgi?targetID=2854227) | 104 | 96 | hsa-miR-20a-5p | [STXBP5](http://www.ncbi.nlm.nih.gov/entrez/query.fcgi?db=gene&cmd=Retrieve&dopt=full_report&list_uids=134957) | syntaxin binding protein 5 |
| [Details](https://mirdb.org/cgi-bin/target_detail.cgi?targetID=2854242) | 105 | 96 | hsa-miR-20a-5p | [UBXN2A](http://www.ncbi.nlm.nih.gov/entrez/query.fcgi?db=gene&cmd=Retrieve&dopt=full_report&list_uids=165324) | UBX domain protein 2A |
| [Details](https://mirdb.org/cgi-bin/target_detail.cgi?targetID=2854252) | 106 | 96 | hsa-miR-20a-5p | [FAM117B](http://www.ncbi.nlm.nih.gov/entrez/query.fcgi?db=gene&cmd=Retrieve&dopt=full_report&list_uids=150864) | family with sequence similarity 117 member B |
| [Details](https://mirdb.org/cgi-bin/target_detail.cgi?targetID=2854260) | 107 | 96 | hsa-miR-20a-5p | [AGTPBP1](http://www.ncbi.nlm.nih.gov/entrez/query.fcgi?db=gene&cmd=Retrieve&dopt=full_report&list_uids=23287) | ATP/GTP binding protein 1 |
| [Details](https://mirdb.org/cgi-bin/target_detail.cgi?targetID=2854298) | 108 | 96 | hsa-miR-20a-5p | [FAM126B](http://www.ncbi.nlm.nih.gov/entrez/query.fcgi?db=gene&cmd=Retrieve&dopt=full_report&list_uids=285172) | family with sequence similarity 126 member B |
| [Details](https://mirdb.org/cgi-bin/target_detail.cgi?targetID=2854322) | 109 | 96 | hsa-miR-20a-5p | [ZBTB7A](http://www.ncbi.nlm.nih.gov/entrez/query.fcgi?db=gene&cmd=Retrieve&dopt=full_report&list_uids=51341) | zinc finger and BTB domain containing 7A |
| [Details](https://mirdb.org/cgi-bin/target_detail.cgi?targetID=2854323) | 110 | 96 | hsa-miR-20a-5p | [CMTR2](http://www.ncbi.nlm.nih.gov/entrez/query.fcgi?db=gene&cmd=Retrieve&dopt=full_report&list_uids=55783) | cap methyltransferase 2 |
| [Details](https://mirdb.org/cgi-bin/target_detail.cgi?targetID=2854355) | 111 | 96 | hsa-miR-20a-5p | [TNFRSF21](http://www.ncbi.nlm.nih.gov/entrez/query.fcgi?db=gene&cmd=Retrieve&dopt=full_report&list_uids=27242) | TNF receptor superfamily member 21 |
| [Details](https://mirdb.org/cgi-bin/target_detail.cgi?targetID=2854370) | 112 | 96 | hsa-miR-20a-5p | [TGFBR2](http://www.ncbi.nlm.nih.gov/entrez/query.fcgi?db=gene&cmd=Retrieve&dopt=full_report&list_uids=7048) | transforming growth factor beta receptor 2 |
| [Details](https://mirdb.org/cgi-bin/target_detail.cgi?targetID=2854440) | 113 | 96 | hsa-miR-20a-5p | [SEMA7A](http://www.ncbi.nlm.nih.gov/entrez/query.fcgi?db=gene&cmd=Retrieve&dopt=full_report&list_uids=8482) | semaphorin 7A (John Milton Hagen blood group) |
| [Details](https://mirdb.org/cgi-bin/target_detail.cgi?targetID=2854471) | 114 | 96 | hsa-miR-20a-5p | [ZFP91](http://www.ncbi.nlm.nih.gov/entrez/query.fcgi?db=gene&cmd=Retrieve&dopt=full_report&list_uids=80829) | ZFP91 zinc finger protein |
| [Details](https://mirdb.org/cgi-bin/target_detail.cgi?targetID=2854492) | 115 | 96 | hsa-miR-20a-5p | [HS3ST5](http://www.ncbi.nlm.nih.gov/entrez/query.fcgi?db=gene&cmd=Retrieve&dopt=full_report&list_uids=222537) | heparan sulfate-glucosamine 3-sulfotransferase 5 |
| [Details](https://mirdb.org/cgi-bin/target_detail.cgi?targetID=2854545) | 116 | 96 | hsa-miR-20a-5p | [SPRED1](http://www.ncbi.nlm.nih.gov/entrez/query.fcgi?db=gene&cmd=Retrieve&dopt=full_report&list_uids=161742) | sprouty related EVH1 domain containing 1 |
| [Details](https://mirdb.org/cgi-bin/target_detail.cgi?targetID=2854711) | 117 | 96 | hsa-miR-20a-5p | [SERTAD2](http://www.ncbi.nlm.nih.gov/entrez/query.fcgi?db=gene&cmd=Retrieve&dopt=full_report&list_uids=9792) | SERTA domain containing 2 |
| [Details](https://mirdb.org/cgi-bin/target_detail.cgi?targetID=2854725) | 118 | 96 | hsa-miR-20a-5p | [MAP3K14](http://www.ncbi.nlm.nih.gov/entrez/query.fcgi?db=gene&cmd=Retrieve&dopt=full_report&list_uids=9020) | mitogen-activated protein kinase kinase kinase 14 |
| [Details](https://mirdb.org/cgi-bin/target_detail.cgi?targetID=2854762) | 119 | 96 | hsa-miR-20a-5p | [PRRG1](http://www.ncbi.nlm.nih.gov/entrez/query.fcgi?db=gene&cmd=Retrieve&dopt=full_report&list_uids=5638) | proline rich and Gla domain 1 |
| [Details](https://mirdb.org/cgi-bin/target_detail.cgi?targetID=2854766) | 120 | 96 | hsa-miR-20a-5p | [ATAD2](http://www.ncbi.nlm.nih.gov/entrez/query.fcgi?db=gene&cmd=Retrieve&dopt=full_report&list_uids=29028) | ATPase family, AAA domain containing 2 |
| [Details](https://mirdb.org/cgi-bin/target_detail.cgi?targetID=2854809) | 121 | 96 | hsa-miR-20a-5p | [PHC3](http://www.ncbi.nlm.nih.gov/entrez/query.fcgi?db=gene&cmd=Retrieve&dopt=full_report&list_uids=80012) | polyhomeotic homolog 3 |
| [Details](https://mirdb.org/cgi-bin/target_detail.cgi?targetID=2854826) | 122 | 96 | hsa-miR-20a-5p | [DPYSL5](http://www.ncbi.nlm.nih.gov/entrez/query.fcgi?db=gene&cmd=Retrieve&dopt=full_report&list_uids=56896) | dihydropyrimidinase like 5 |
| [Details](https://mirdb.org/cgi-bin/target_detail.cgi?targetID=2854827) | 123 | 96 | hsa-miR-20a-5p | [USP31](http://www.ncbi.nlm.nih.gov/entrez/query.fcgi?db=gene&cmd=Retrieve&dopt=full_report&list_uids=57478) | ubiquitin specific peptidase 31 |
| [Details](https://mirdb.org/cgi-bin/target_detail.cgi?targetID=2854831) | 124 | 96 | hsa-miR-20a-5p | [ZNF652](http://www.ncbi.nlm.nih.gov/entrez/query.fcgi?db=gene&cmd=Retrieve&dopt=full_report&list_uids=22834) | zinc finger protein 652 |
| [Details](https://mirdb.org/cgi-bin/target_detail.cgi?targetID=2854873) | 125 | 96 | hsa-miR-20a-5p | [PEX5L](http://www.ncbi.nlm.nih.gov/entrez/query.fcgi?db=gene&cmd=Retrieve&dopt=full_report&list_uids=51555) | peroxisomal biogenesis factor 5 like |
| [Details](https://mirdb.org/cgi-bin/target_detail.cgi?targetID=2854891) | 126 | 96 | hsa-miR-20a-5p | [ZNF704](http://www.ncbi.nlm.nih.gov/entrez/query.fcgi?db=gene&cmd=Retrieve&dopt=full_report&list_uids=619279) | zinc finger protein 704 |
| [Details](https://mirdb.org/cgi-bin/target_detail.cgi?targetID=2854908) | 127 | 96 | hsa-miR-20a-5p | [FGD5](http://www.ncbi.nlm.nih.gov/entrez/query.fcgi?db=gene&cmd=Retrieve&dopt=full_report&list_uids=152273) | FYVE, RhoGEF and PH domain containing 5 |
| [Details](https://mirdb.org/cgi-bin/target_detail.cgi?targetID=2854921) | 128 | 96 | hsa-miR-20a-5p | [LAPTM4A](http://www.ncbi.nlm.nih.gov/entrez/query.fcgi?db=gene&cmd=Retrieve&dopt=full_report&list_uids=9741) | lysosomal protein transmembrane 4 alpha |
| [Details](https://mirdb.org/cgi-bin/target_detail.cgi?targetID=2854984) | 129 | 96 | hsa-miR-20a-5p | [TRIM36](http://www.ncbi.nlm.nih.gov/entrez/query.fcgi?db=gene&cmd=Retrieve&dopt=full_report&list_uids=55521) | tripartite motif containing 36 |
| [Details](https://mirdb.org/cgi-bin/target_detail.cgi?targetID=2854999) | 130 | 96 | hsa-miR-20a-5p | [CHRM2](http://www.ncbi.nlm.nih.gov/entrez/query.fcgi?db=gene&cmd=Retrieve&dopt=full_report&list_uids=1129) | cholinergic receptor muscarinic 2 |
| [Details](https://mirdb.org/cgi-bin/target_detail.cgi?targetID=2855034) | 131 | 96 | hsa-miR-20a-5p | [EGLN3](http://www.ncbi.nlm.nih.gov/entrez/query.fcgi?db=gene&cmd=Retrieve&dopt=full_report&list_uids=112399) | egl-9 family hypoxia inducible factor 3 |
| [Details](https://mirdb.org/cgi-bin/target_detail.cgi?targetID=2855071) | 132 | 96 | hsa-miR-20a-5p | [PURB](http://www.ncbi.nlm.nih.gov/entrez/query.fcgi?db=gene&cmd=Retrieve&dopt=full_report&list_uids=5814) | purine rich element binding protein B |
| [Details](https://mirdb.org/cgi-bin/target_detail.cgi?targetID=2855123) | 133 | 96 | hsa-miR-20a-5p | [JPT1](http://www.ncbi.nlm.nih.gov/entrez/query.fcgi?db=gene&cmd=Retrieve&dopt=full_report&list_uids=51155) | Jupiter microtubule associated homolog 1 |
| [Details](https://mirdb.org/cgi-bin/target_detail.cgi?targetID=2855132) | 134 | 96 | hsa-miR-20a-5p | [VSX1](http://www.ncbi.nlm.nih.gov/entrez/query.fcgi?db=gene&cmd=Retrieve&dopt=full_report&list_uids=30813) | visual system homeobox 1 |
| [Details](https://mirdb.org/cgi-bin/target_detail.cgi?targetID=2855168) | 135 | 96 | hsa-miR-20a-5p | [TET3](http://www.ncbi.nlm.nih.gov/entrez/query.fcgi?db=gene&cmd=Retrieve&dopt=full_report&list_uids=200424) | tet methylcytosine dioxygenase 3 |
| [Details](https://mirdb.org/cgi-bin/target_detail.cgi?targetID=2855226) | 136 | 96 | hsa-miR-20a-5p | [CNOT4](http://www.ncbi.nlm.nih.gov/entrez/query.fcgi?db=gene&cmd=Retrieve&dopt=full_report&list_uids=4850) | CCR4-NOT transcription complex subunit 4 |
| [Details](https://mirdb.org/cgi-bin/target_detail.cgi?targetID=2855251) | 137 | 96 | hsa-miR-20a-5p | [FJX1](http://www.ncbi.nlm.nih.gov/entrez/query.fcgi?db=gene&cmd=Retrieve&dopt=full_report&list_uids=24147) | four-jointed box kinase 1 |
| [Details](https://mirdb.org/cgi-bin/target_detail.cgi?targetID=2855363) | 138 | 96 | hsa-miR-20a-5p | [ANKRD29](http://www.ncbi.nlm.nih.gov/entrez/query.fcgi?db=gene&cmd=Retrieve&dopt=full_report&list_uids=147463) | ankyrin repeat domain 29 |
| [Details](https://mirdb.org/cgi-bin/target_detail.cgi?targetID=2855396) | 139 | 96 | hsa-miR-20a-5p | [ZC3H12C](http://www.ncbi.nlm.nih.gov/entrez/query.fcgi?db=gene&cmd=Retrieve&dopt=full_report&list_uids=85463) | zinc finger CCCH-type containing 12C |
| [Details](https://mirdb.org/cgi-bin/target_detail.cgi?targetID=2855429) | 140 | 96 | hsa-miR-20a-5p | [ARID4A](http://www.ncbi.nlm.nih.gov/entrez/query.fcgi?db=gene&cmd=Retrieve&dopt=full_report&list_uids=5926) | AT-rich interaction domain 4A |
| [Details](https://mirdb.org/cgi-bin/target_detail.cgi?targetID=2854088) | 141 | 95 | hsa-miR-20a-5p | [KAT2B](http://www.ncbi.nlm.nih.gov/entrez/query.fcgi?db=gene&cmd=Retrieve&dopt=full_report&list_uids=8850) | lysine acetyltransferase 2B |
| [Details](https://mirdb.org/cgi-bin/target_detail.cgi?targetID=2854100) | 142 | 95 | hsa-miR-20a-5p | [EPHA7](http://www.ncbi.nlm.nih.gov/entrez/query.fcgi?db=gene&cmd=Retrieve&dopt=full_report&list_uids=2045) | EPH receptor A7 |
| [Details](https://mirdb.org/cgi-bin/target_detail.cgi?targetID=2854235) | 143 | 95 | hsa-miR-20a-5p | [MASTL](http://www.ncbi.nlm.nih.gov/entrez/query.fcgi?db=gene&cmd=Retrieve&dopt=full_report&list_uids=84930) | microtubule associated serine/threonine kinase like |
| [Details](https://mirdb.org/cgi-bin/target_detail.cgi?targetID=2854240) | 144 | 95 | hsa-miR-20a-5p | [NCKAP5](http://www.ncbi.nlm.nih.gov/entrez/query.fcgi?db=gene&cmd=Retrieve&dopt=full_report&list_uids=344148) | NCK associated protein 5 |
| [Details](https://mirdb.org/cgi-bin/target_detail.cgi?targetID=2854254) | 145 | 95 | hsa-miR-20a-5p | [SALL3](http://www.ncbi.nlm.nih.gov/entrez/query.fcgi?db=gene&cmd=Retrieve&dopt=full_report&list_uids=27164) | spalt like transcription factor 3 |
| [Details](https://mirdb.org/cgi-bin/target_detail.cgi?targetID=2854311) | 146 | 95 | hsa-miR-20a-5p | [LAMA3](http://www.ncbi.nlm.nih.gov/entrez/query.fcgi?db=gene&cmd=Retrieve&dopt=full_report&list_uids=3909) | laminin subunit alpha 3 |
| [Details](https://mirdb.org/cgi-bin/target_detail.cgi?targetID=2854342) | 147 | 95 | hsa-miR-20a-5p | [CEP97](http://www.ncbi.nlm.nih.gov/entrez/query.fcgi?db=gene&cmd=Retrieve&dopt=full_report&list_uids=79598) | centrosomal protein 97 |
| [Details](https://mirdb.org/cgi-bin/target_detail.cgi?targetID=2854361) | 148 | 95 | hsa-miR-20a-5p | [B3GALT2](http://www.ncbi.nlm.nih.gov/entrez/query.fcgi?db=gene&cmd=Retrieve&dopt=full_report&list_uids=8707) | beta-1,3-galactosyltransferase 2 |
| [Details](https://mirdb.org/cgi-bin/target_detail.cgi?targetID=2854400) | 149 | 95 | hsa-miR-20a-5p | [PGM2L1](http://www.ncbi.nlm.nih.gov/entrez/query.fcgi?db=gene&cmd=Retrieve&dopt=full_report&list_uids=283209) | phosphoglucomutase 2 like 1 |
| [Details](https://mirdb.org/cgi-bin/target_detail.cgi?targetID=2854405) | 150 | 95 | hsa-miR-20a-5p | [SLC17A7](http://www.ncbi.nlm.nih.gov/entrez/query.fcgi?db=gene&cmd=Retrieve&dopt=full_report&list_uids=57030) | solute carrier family 17 member 7 |
| [Details](https://mirdb.org/cgi-bin/target_detail.cgi?targetID=2854470) | 151 | 95 | hsa-miR-20a-5p | [DUSP2](http://www.ncbi.nlm.nih.gov/entrez/query.fcgi?db=gene&cmd=Retrieve&dopt=full_report&list_uids=1844) | dual specificity phosphatase 2 |
| [Details](https://mirdb.org/cgi-bin/target_detail.cgi?targetID=2854504) | 152 | 95 | hsa-miR-20a-5p | [SALL1](http://www.ncbi.nlm.nih.gov/entrez/query.fcgi?db=gene&cmd=Retrieve&dopt=full_report&list_uids=6299) | spalt like transcription factor 1 |
| [Details](https://mirdb.org/cgi-bin/target_detail.cgi?targetID=2854512) | 153 | 95 | hsa-miR-20a-5p | [RASD1](http://www.ncbi.nlm.nih.gov/entrez/query.fcgi?db=gene&cmd=Retrieve&dopt=full_report&list_uids=51655) | ras related dexamethasone induced 1 |
| [Details](https://mirdb.org/cgi-bin/target_detail.cgi?targetID=2854537) | 154 | 95 | hsa-miR-20a-5p | [LPGAT1](http://www.ncbi.nlm.nih.gov/entrez/query.fcgi?db=gene&cmd=Retrieve&dopt=full_report&list_uids=9926) | lysophosphatidylglycerol acyltransferase 1 |
| [Details](https://mirdb.org/cgi-bin/target_detail.cgi?targetID=2854571) | 155 | 95 | hsa-miR-20a-5p | [F3](http://www.ncbi.nlm.nih.gov/entrez/query.fcgi?db=gene&cmd=Retrieve&dopt=full_report&list_uids=2152) | coagulation factor III, tissue factor |
| [Details](https://mirdb.org/cgi-bin/target_detail.cgi?targetID=2854580) | 156 | 95 | hsa-miR-20a-5p | [SPOPL](http://www.ncbi.nlm.nih.gov/entrez/query.fcgi?db=gene&cmd=Retrieve&dopt=full_report&list_uids=339745) | speckle type BTB/POZ protein like |
| [Details](https://mirdb.org/cgi-bin/target_detail.cgi?targetID=2854588) | 157 | 95 | hsa-miR-20a-5p | [E2F5](http://www.ncbi.nlm.nih.gov/entrez/query.fcgi?db=gene&cmd=Retrieve&dopt=full_report&list_uids=1875) | E2F transcription factor 5 |
| [Details](https://mirdb.org/cgi-bin/target_detail.cgi?targetID=2854606) | 158 | 95 | hsa-miR-20a-5p | [RAB11FIP5](http://www.ncbi.nlm.nih.gov/entrez/query.fcgi?db=gene&cmd=Retrieve&dopt=full_report&list_uids=26056) | RAB11 family interacting protein 5 |
| [Details](https://mirdb.org/cgi-bin/target_detail.cgi?targetID=2854628) | 159 | 95 | hsa-miR-20a-5p | [PFKP](http://www.ncbi.nlm.nih.gov/entrez/query.fcgi?db=gene&cmd=Retrieve&dopt=full_report&list_uids=5214) | phosphofructokinase, platelet |
| [Details](https://mirdb.org/cgi-bin/target_detail.cgi?targetID=2854663) | 160 | 95 | hsa-miR-20a-5p | [IRF9](http://www.ncbi.nlm.nih.gov/entrez/query.fcgi?db=gene&cmd=Retrieve&dopt=full_report&list_uids=10379) | interferon regulatory factor 9 |
| [Details](https://mirdb.org/cgi-bin/target_detail.cgi?targetID=2854839) | 161 | 95 | hsa-miR-20a-5p | [ANKRD33B](http://www.ncbi.nlm.nih.gov/entrez/query.fcgi?db=gene&cmd=Retrieve&dopt=full_report&list_uids=651746) | ankyrin repeat domain 33B |
| [Details](https://mirdb.org/cgi-bin/target_detail.cgi?targetID=2854952) | 162 | 95 | hsa-miR-20a-5p | [PTHLH](http://www.ncbi.nlm.nih.gov/entrez/query.fcgi?db=gene&cmd=Retrieve&dopt=full_report&list_uids=5744) | parathyroid hormone like hormone |
| [Details](https://mirdb.org/cgi-bin/target_detail.cgi?targetID=2855016) | 163 | 95 | hsa-miR-20a-5p | [RASL11B](http://www.ncbi.nlm.nih.gov/entrez/query.fcgi?db=gene&cmd=Retrieve&dopt=full_report&list_uids=65997) | RAS like family 11 member B |
| [Details](https://mirdb.org/cgi-bin/target_detail.cgi?targetID=2855101) | 164 | 95 | hsa-miR-20a-5p | [UEVLD](http://www.ncbi.nlm.nih.gov/entrez/query.fcgi?db=gene&cmd=Retrieve&dopt=full_report&list_uids=55293) | UEV and lactate/malate dehyrogenase domains |
| [Details](https://mirdb.org/cgi-bin/target_detail.cgi?targetID=2855110) | 165 | 95 | hsa-miR-20a-5p | [TRIP11](http://www.ncbi.nlm.nih.gov/entrez/query.fcgi?db=gene&cmd=Retrieve&dopt=full_report&list_uids=9321) | thyroid hormone receptor interactor 11 |
| [Details](https://mirdb.org/cgi-bin/target_detail.cgi?targetID=2855112) | 166 | 95 | hsa-miR-20a-5p | [MKNK2](http://www.ncbi.nlm.nih.gov/entrez/query.fcgi?db=gene&cmd=Retrieve&dopt=full_report&list_uids=2872) | MAP kinase interacting serine/threonine kinase 2 |
| [Details](https://mirdb.org/cgi-bin/target_detail.cgi?targetID=2855188) | 167 | 95 | hsa-miR-20a-5p | [ARMC8](http://www.ncbi.nlm.nih.gov/entrez/query.fcgi?db=gene&cmd=Retrieve&dopt=full_report&list_uids=25852) | armadillo repeat containing 8 |
| [Details](https://mirdb.org/cgi-bin/target_detail.cgi?targetID=2855278) | 168 | 95 | hsa-miR-20a-5p | [PPP6C](http://www.ncbi.nlm.nih.gov/entrez/query.fcgi?db=gene&cmd=Retrieve&dopt=full_report&list_uids=5537) | protein phosphatase 6 catalytic subunit |
| [Details](https://mirdb.org/cgi-bin/target_detail.cgi?targetID=2855294) | 169 | 95 | hsa-miR-20a-5p | [HAS2](http://www.ncbi.nlm.nih.gov/entrez/query.fcgi?db=gene&cmd=Retrieve&dopt=full_report&list_uids=3037) | hyaluronan synthase 2 |
| [Details](https://mirdb.org/cgi-bin/target_detail.cgi?targetID=2855303) | 170 | 95 | hsa-miR-20a-5p | [LIMK1](http://www.ncbi.nlm.nih.gov/entrez/query.fcgi?db=gene&cmd=Retrieve&dopt=full_report&list_uids=3984) | LIM domain kinase 1 |
| [Details](https://mirdb.org/cgi-bin/target_detail.cgi?targetID=2855336) | 171 | 95 | hsa-miR-20a-5p | [SGMS1](http://www.ncbi.nlm.nih.gov/entrez/query.fcgi?db=gene&cmd=Retrieve&dopt=full_report&list_uids=259230) | sphingomyelin synthase 1 |
| [Details](https://mirdb.org/cgi-bin/target_detail.cgi?targetID=2855408) | 172 | 95 | hsa-miR-20a-5p | [RORA](http://www.ncbi.nlm.nih.gov/entrez/query.fcgi?db=gene&cmd=Retrieve&dopt=full_report&list_uids=6095) | RAR related orphan receptor A |
| [Details](https://mirdb.org/cgi-bin/target_detail.cgi?targetID=2855438) | 173 | 95 | hsa-miR-20a-5p | [NAGK](http://www.ncbi.nlm.nih.gov/entrez/query.fcgi?db=gene&cmd=Retrieve&dopt=full_report&list_uids=55577) | N-acetylglucosamine kinase |
| [Details](https://mirdb.org/cgi-bin/target_detail.cgi?targetID=2854082) | 174 | 94 | hsa-miR-20a-5p | [LIMA1](http://www.ncbi.nlm.nih.gov/entrez/query.fcgi?db=gene&cmd=Retrieve&dopt=full_report&list_uids=51474) | LIM domain and actin binding 1 |
| [Details](https://mirdb.org/cgi-bin/target_detail.cgi?targetID=2854119) | 175 | 94 | hsa-miR-20a-5p | [PPP1R3B](http://www.ncbi.nlm.nih.gov/entrez/query.fcgi?db=gene&cmd=Retrieve&dopt=full_report&list_uids=79660) | protein phosphatase 1 regulatory subunit 3B |
| [Details](https://mirdb.org/cgi-bin/target_detail.cgi?targetID=2854126) | 176 | 94 | hsa-miR-20a-5p | [RORC](http://www.ncbi.nlm.nih.gov/entrez/query.fcgi?db=gene&cmd=Retrieve&dopt=full_report&list_uids=6097) | RAR related orphan receptor C |
| [Details](https://mirdb.org/cgi-bin/target_detail.cgi?targetID=2854156) | 177 | 94 | hsa-miR-20a-5p | [BTBD10](http://www.ncbi.nlm.nih.gov/entrez/query.fcgi?db=gene&cmd=Retrieve&dopt=full_report&list_uids=84280) | BTB domain containing 10 |
| [Details](https://mirdb.org/cgi-bin/target_detail.cgi?targetID=2854167) | 178 | 94 | hsa-miR-20a-5p | [PAK5](http://www.ncbi.nlm.nih.gov/entrez/query.fcgi?db=gene&cmd=Retrieve&dopt=full_report&list_uids=57144) | p21 (RAC1) activated kinase 5 |
| [Details](https://mirdb.org/cgi-bin/target_detail.cgi?targetID=2854213) | 179 | 94 | hsa-miR-20a-5p | [TMEM127](http://www.ncbi.nlm.nih.gov/entrez/query.fcgi?db=gene&cmd=Retrieve&dopt=full_report&list_uids=55654) | transmembrane protein 127 |
| [Details](https://mirdb.org/cgi-bin/target_detail.cgi?targetID=2854234) | 180 | 94 | hsa-miR-20a-5p | [SLC46A3](http://www.ncbi.nlm.nih.gov/entrez/query.fcgi?db=gene&cmd=Retrieve&dopt=full_report&list_uids=283537) | solute carrier family 46 member 3 |
| [Details](https://mirdb.org/cgi-bin/target_detail.cgi?targetID=2854251) | 181 | 94 | hsa-miR-20a-5p | [ABCA1](http://www.ncbi.nlm.nih.gov/entrez/query.fcgi?db=gene&cmd=Retrieve&dopt=full_report&list_uids=19) | ATP binding cassette subfamily A member 1 |
| [Details](https://mirdb.org/cgi-bin/target_detail.cgi?targetID=2854296) | 182 | 94 | hsa-miR-20a-5p | [DDHD1](http://www.ncbi.nlm.nih.gov/entrez/query.fcgi?db=gene&cmd=Retrieve&dopt=full_report&list_uids=80821) | DDHD domain containing 1 |
| [Details](https://mirdb.org/cgi-bin/target_detail.cgi?targetID=2854299) | 183 | 94 | hsa-miR-20a-5p | [PDE3B](http://www.ncbi.nlm.nih.gov/entrez/query.fcgi?db=gene&cmd=Retrieve&dopt=full_report&list_uids=5140) | phosphodiesterase 3B |
| [Details](https://mirdb.org/cgi-bin/target_detail.cgi?targetID=2854338) | 184 | 94 | hsa-miR-20a-5p | [TM2D2](http://www.ncbi.nlm.nih.gov/entrez/query.fcgi?db=gene&cmd=Retrieve&dopt=full_report&list_uids=83877) | TM2 domain containing 2 |
| [Details](https://mirdb.org/cgi-bin/target_detail.cgi?targetID=2854365) | 185 | 94 | hsa-miR-20a-5p | [PTPRD](http://www.ncbi.nlm.nih.gov/entrez/query.fcgi?db=gene&cmd=Retrieve&dopt=full_report&list_uids=5789) | protein tyrosine phosphatase, receptor type D |
| [Details](https://mirdb.org/cgi-bin/target_detail.cgi?targetID=2854366) | 186 | 94 | hsa-miR-20a-5p | [ANKRD17](http://www.ncbi.nlm.nih.gov/entrez/query.fcgi?db=gene&cmd=Retrieve&dopt=full_report&list_uids=26057) | ankyrin repeat domain 17 |
| [Details](https://mirdb.org/cgi-bin/target_detail.cgi?targetID=2854432) | 187 | 94 | hsa-miR-20a-5p | [AGO1](http://www.ncbi.nlm.nih.gov/entrez/query.fcgi?db=gene&cmd=Retrieve&dopt=full_report&list_uids=26523) | argonaute RISC catalytic component 1 |
| [Details](https://mirdb.org/cgi-bin/target_detail.cgi?targetID=2854491) | 188 | 94 | hsa-miR-20a-5p | [UBE2Q2](http://www.ncbi.nlm.nih.gov/entrez/query.fcgi?db=gene&cmd=Retrieve&dopt=full_report&list_uids=92912) | ubiquitin conjugating enzyme E2 Q2 |
| [Details](https://mirdb.org/cgi-bin/target_detail.cgi?targetID=2854530) | 189 | 94 | hsa-miR-20a-5p | [ANKH](http://www.ncbi.nlm.nih.gov/entrez/query.fcgi?db=gene&cmd=Retrieve&dopt=full_report&list_uids=56172) | ANKH inorganic pyrophosphate transport regulator |
| [Details](https://mirdb.org/cgi-bin/target_detail.cgi?targetID=2854562) | 190 | 94 | hsa-miR-20a-5p | [HBP1](http://www.ncbi.nlm.nih.gov/entrez/query.fcgi?db=gene&cmd=Retrieve&dopt=full_report&list_uids=26959) | HMG-box transcription factor 1 |
| [Details](https://mirdb.org/cgi-bin/target_detail.cgi?targetID=2854651) | 191 | 94 | hsa-miR-20a-5p | [XRN1](http://www.ncbi.nlm.nih.gov/entrez/query.fcgi?db=gene&cmd=Retrieve&dopt=full_report&list_uids=54464) | 5'-3' exoribonuclease 1 |
| [Details](https://mirdb.org/cgi-bin/target_detail.cgi?targetID=2854739) | 192 | 94 | hsa-miR-20a-5p | [CAPRIN2](http://www.ncbi.nlm.nih.gov/entrez/query.fcgi?db=gene&cmd=Retrieve&dopt=full_report&list_uids=65981) | caprin family member 2 |
| [Details](https://mirdb.org/cgi-bin/target_detail.cgi?targetID=2854756) | 193 | 94 | hsa-miR-20a-5p | [KIF3B](http://www.ncbi.nlm.nih.gov/entrez/query.fcgi?db=gene&cmd=Retrieve&dopt=full_report&list_uids=9371) | kinesin family member 3B |
| [Details](https://mirdb.org/cgi-bin/target_detail.cgi?targetID=2854761) | 194 | 94 | hsa-miR-20a-5p | [FAT2](http://www.ncbi.nlm.nih.gov/entrez/query.fcgi?db=gene&cmd=Retrieve&dopt=full_report&list_uids=2196) | FAT atypical cadherin 2 |
| [Details](https://mirdb.org/cgi-bin/target_detail.cgi?targetID=2854765) | 195 | 94 | hsa-miR-20a-5p | [KLHL2](http://www.ncbi.nlm.nih.gov/entrez/query.fcgi?db=gene&cmd=Retrieve&dopt=full_report&list_uids=11275) | kelch like family member 2 |
| [Details](https://mirdb.org/cgi-bin/target_detail.cgi?targetID=2854815) | 196 | 94 | hsa-miR-20a-5p | [BTG3](http://www.ncbi.nlm.nih.gov/entrez/query.fcgi?db=gene&cmd=Retrieve&dopt=full_report&list_uids=10950) | BTG anti-proliferation factor 3 |
| [Details](https://mirdb.org/cgi-bin/target_detail.cgi?targetID=2854846) | 197 | 94 | hsa-miR-20a-5p | [PTPN3](http://www.ncbi.nlm.nih.gov/entrez/query.fcgi?db=gene&cmd=Retrieve&dopt=full_report&list_uids=5774) | protein tyrosine phosphatase, non-receptor type 3 |
| [Details](https://mirdb.org/cgi-bin/target_detail.cgi?targetID=2854852) | 198 | 94 | hsa-miR-20a-5p | [BBX](http://www.ncbi.nlm.nih.gov/entrez/query.fcgi?db=gene&cmd=Retrieve&dopt=full_report&list_uids=56987) | BBX, HMG-box containing |
| [Details](https://mirdb.org/cgi-bin/target_detail.cgi?targetID=2854857) | 199 | 94 | hsa-miR-20a-5p | [SSH1](http://www.ncbi.nlm.nih.gov/entrez/query.fcgi?db=gene&cmd=Retrieve&dopt=full_report&list_uids=54434) | slingshot protein phosphatase 1 |
| [Details](https://mirdb.org/cgi-bin/target_detail.cgi?targetID=2854866) | 200 | 94 | hsa-miR-20a-5p | [FBXO48](http://www.ncbi.nlm.nih.gov/entrez/query.fcgi?db=gene&cmd=Retrieve&dopt=full_report&list_uids=554251) | F-box protein 48 |
| [Details](https://mirdb.org/cgi-bin/target_detail.cgi?targetID=2854902) | 201 | 94 | hsa-miR-20a-5p | [SOS1](http://www.ncbi.nlm.nih.gov/entrez/query.fcgi?db=gene&cmd=Retrieve&dopt=full_report&list_uids=6654) | SOS Ras/Rac guanine nucleotide exchange factor 1 |
| [Details](https://mirdb.org/cgi-bin/target_detail.cgi?targetID=2854995) | 202 | 94 | hsa-miR-20a-5p | [HEG1](http://www.ncbi.nlm.nih.gov/entrez/query.fcgi?db=gene&cmd=Retrieve&dopt=full_report&list_uids=57493) | heart development protein with EGF like domains 1 |
| [Details](https://mirdb.org/cgi-bin/target_detail.cgi?targetID=2855004) | 203 | 94 | hsa-miR-20a-5p | [RAB5B](http://www.ncbi.nlm.nih.gov/entrez/query.fcgi?db=gene&cmd=Retrieve&dopt=full_report&list_uids=5869) | RAB5B, member RAS oncogene family |
| [Details](https://mirdb.org/cgi-bin/target_detail.cgi?targetID=2855017) | 204 | 94 | hsa-miR-20a-5p | [NKIRAS1](http://www.ncbi.nlm.nih.gov/entrez/query.fcgi?db=gene&cmd=Retrieve&dopt=full_report&list_uids=28512) | NFKB inhibitor interacting Ras like 1 |
| [Details](https://mirdb.org/cgi-bin/target_detail.cgi?targetID=2855055) | 205 | 94 | hsa-miR-20a-5p | [SNTB2](http://www.ncbi.nlm.nih.gov/entrez/query.fcgi?db=gene&cmd=Retrieve&dopt=full_report&list_uids=6645) | syntrophin beta 2 |
| [Details](https://mirdb.org/cgi-bin/target_detail.cgi?targetID=2855080) | 206 | 94 | hsa-miR-20a-5p | [NIN](http://www.ncbi.nlm.nih.gov/entrez/query.fcgi?db=gene&cmd=Retrieve&dopt=full_report&list_uids=51199) | ninein |
| [Details](https://mirdb.org/cgi-bin/target_detail.cgi?targetID=2855113) | 207 | 94 | hsa-miR-20a-5p | [FAM219B](http://www.ncbi.nlm.nih.gov/entrez/query.fcgi?db=gene&cmd=Retrieve&dopt=full_report&list_uids=57184) | family with sequence similarity 219 member B |
| [Details](https://mirdb.org/cgi-bin/target_detail.cgi?targetID=2855163) | 208 | 94 | hsa-miR-20a-5p | [LDLRAP1](http://www.ncbi.nlm.nih.gov/entrez/query.fcgi?db=gene&cmd=Retrieve&dopt=full_report&list_uids=26119) | low density lipoprotein receptor adaptor protein 1 |
| [Details](https://mirdb.org/cgi-bin/target_detail.cgi?targetID=2855189) | 209 | 94 | hsa-miR-20a-5p | [HAUS8](http://www.ncbi.nlm.nih.gov/entrez/query.fcgi?db=gene&cmd=Retrieve&dopt=full_report&list_uids=93323) | HAUS augmin like complex subunit 8 |
| [Details](https://mirdb.org/cgi-bin/target_detail.cgi?targetID=2855220) | 210 | 94 | hsa-miR-20a-5p | [PCDH15](http://www.ncbi.nlm.nih.gov/entrez/query.fcgi?db=gene&cmd=Retrieve&dopt=full_report&list_uids=65217) | protocadherin related 15 |
| [Details](https://mirdb.org/cgi-bin/target_detail.cgi?targetID=2855266) | 211 | 94 | hsa-miR-20a-5p | [BRWD1](http://www.ncbi.nlm.nih.gov/entrez/query.fcgi?db=gene&cmd=Retrieve&dopt=full_report&list_uids=54014) | bromodomain and WD repeat domain containing 1 |
| [Details](https://mirdb.org/cgi-bin/target_detail.cgi?targetID=2855300) | 212 | 94 | hsa-miR-20a-5p | [TOPORS](http://www.ncbi.nlm.nih.gov/entrez/query.fcgi?db=gene&cmd=Retrieve&dopt=full_report&list_uids=10210) | TOP1 binding arginine/serine rich protein |
| [Details](https://mirdb.org/cgi-bin/target_detail.cgi?targetID=2855301) | 213 | 94 | hsa-miR-20a-5p | [GPR137B](http://www.ncbi.nlm.nih.gov/entrez/query.fcgi?db=gene&cmd=Retrieve&dopt=full_report&list_uids=7107) | G protein-coupled receptor 137B |
| [Details](https://mirdb.org/cgi-bin/target_detail.cgi?targetID=2855306) | 214 | 94 | hsa-miR-20a-5p | [ZBTB20](http://www.ncbi.nlm.nih.gov/entrez/query.fcgi?db=gene&cmd=Retrieve&dopt=full_report&list_uids=26137) | zinc finger and BTB domain containing 20 |
| [Details](https://mirdb.org/cgi-bin/target_detail.cgi?targetID=2855308) | 215 | 94 | hsa-miR-20a-5p | [GAB1](http://www.ncbi.nlm.nih.gov/entrez/query.fcgi?db=gene&cmd=Retrieve&dopt=full_report&list_uids=2549) | GRB2 associated binding protein 1 |
| [Details](https://mirdb.org/cgi-bin/target_detail.cgi?targetID=2855382) | 216 | 94 | hsa-miR-20a-5p | [CLIP4](http://www.ncbi.nlm.nih.gov/entrez/query.fcgi?db=gene&cmd=Retrieve&dopt=full_report&list_uids=79745) | CAP-Gly domain containing linker protein family member 4 |
| [Details](https://mirdb.org/cgi-bin/target_detail.cgi?targetID=2855392) | 217 | 94 | hsa-miR-20a-5p | [DNAJC27](http://www.ncbi.nlm.nih.gov/entrez/query.fcgi?db=gene&cmd=Retrieve&dopt=full_report&list_uids=51277) | DnaJ heat shock protein family (Hsp40) member C27 |
| [Details](https://mirdb.org/cgi-bin/target_detail.cgi?targetID=2855397) | 218 | 94 | hsa-miR-20a-5p | [ADARB1](http://www.ncbi.nlm.nih.gov/entrez/query.fcgi?db=gene&cmd=Retrieve&dopt=full_report&list_uids=104) | adenosine deaminase, RNA specific B1 |
| [Details](https://mirdb.org/cgi-bin/target_detail.cgi?targetID=2855412) | 219 | 94 | hsa-miR-20a-5p | [KMT2A](http://www.ncbi.nlm.nih.gov/entrez/query.fcgi?db=gene&cmd=Retrieve&dopt=full_report&list_uids=4297) | lysine methyltransferase 2A |
| [Details](https://mirdb.org/cgi-bin/target_detail.cgi?targetID=2855423) | 220 | 94 | hsa-miR-20a-5p | [FZD3](http://www.ncbi.nlm.nih.gov/entrez/query.fcgi?db=gene&cmd=Retrieve&dopt=full_report&list_uids=7976) | frizzled class receptor 3 |
| [Details](https://mirdb.org/cgi-bin/target_detail.cgi?targetID=2854113) | 221 | 93 | hsa-miR-20a-5p | [SEMA4B](http://www.ncbi.nlm.nih.gov/entrez/query.fcgi?db=gene&cmd=Retrieve&dopt=full_report&list_uids=10509) | semaphorin 4B |
| [Details](https://mirdb.org/cgi-bin/target_detail.cgi?targetID=2854143) | 222 | 93 | hsa-miR-20a-5p | [ADAM9](http://www.ncbi.nlm.nih.gov/entrez/query.fcgi?db=gene&cmd=Retrieve&dopt=full_report&list_uids=8754) | ADAM metallopeptidase domain 9 |
| [Details](https://mirdb.org/cgi-bin/target_detail.cgi?targetID=2854166) | 223 | 93 | hsa-miR-20a-5p | [BMPR2](http://www.ncbi.nlm.nih.gov/entrez/query.fcgi?db=gene&cmd=Retrieve&dopt=full_report&list_uids=659) | bone morphogenetic protein receptor type 2 |
| [Details](https://mirdb.org/cgi-bin/target_detail.cgi?targetID=2854286) | 224 | 93 | hsa-miR-20a-5p | [CCNG2](http://www.ncbi.nlm.nih.gov/entrez/query.fcgi?db=gene&cmd=Retrieve&dopt=full_report&list_uids=901) | cyclin G2 |
| [Details](https://mirdb.org/cgi-bin/target_detail.cgi?targetID=2854294) | 225 | 93 | hsa-miR-20a-5p | [TAOK3](http://www.ncbi.nlm.nih.gov/entrez/query.fcgi?db=gene&cmd=Retrieve&dopt=full_report&list_uids=51347) | TAO kinase 3 |
| [Details](https://mirdb.org/cgi-bin/target_detail.cgi?targetID=2854307) | 226 | 93 | hsa-miR-20a-5p | [PPP1R15B](http://www.ncbi.nlm.nih.gov/entrez/query.fcgi?db=gene&cmd=Retrieve&dopt=full_report&list_uids=84919) | protein phosphatase 1 regulatory subunit 15B |
| [Details](https://mirdb.org/cgi-bin/target_detail.cgi?targetID=2854369) | 227 | 93 | hsa-miR-20a-5p | [NEDD4L](http://www.ncbi.nlm.nih.gov/entrez/query.fcgi?db=gene&cmd=Retrieve&dopt=full_report&list_uids=23327) | neural precursor cell expressed, developmentally down-regulated 4-like, E3 ubiquitin protein ligase |
| [Details](https://mirdb.org/cgi-bin/target_detail.cgi?targetID=2854377) | 228 | 93 | hsa-miR-20a-5p | [IQSEC2](http://www.ncbi.nlm.nih.gov/entrez/query.fcgi?db=gene&cmd=Retrieve&dopt=full_report&list_uids=23096) | IQ motif and Sec7 domain 2 |
| [Details](https://mirdb.org/cgi-bin/target_detail.cgi?targetID=2854389) | 229 | 93 | hsa-miR-20a-5p | [DENND5B](http://www.ncbi.nlm.nih.gov/entrez/query.fcgi?db=gene&cmd=Retrieve&dopt=full_report&list_uids=160518) | DENN domain containing 5B |
| [Details](https://mirdb.org/cgi-bin/target_detail.cgi?targetID=2854413) | 230 | 93 | hsa-miR-20a-5p | [SCN1A](http://www.ncbi.nlm.nih.gov/entrez/query.fcgi?db=gene&cmd=Retrieve&dopt=full_report&list_uids=6323) | sodium voltage-gated channel alpha subunit 1 |
| [Details](https://mirdb.org/cgi-bin/target_detail.cgi?targetID=2854418) | 231 | 93 | hsa-miR-20a-5p | [YOD1](http://www.ncbi.nlm.nih.gov/entrez/query.fcgi?db=gene&cmd=Retrieve&dopt=full_report&list_uids=55432) | YOD1 deubiquitinase |
| [Details](https://mirdb.org/cgi-bin/target_detail.cgi?targetID=2854466) | 232 | 93 | hsa-miR-20a-5p | [ATL3](http://www.ncbi.nlm.nih.gov/entrez/query.fcgi?db=gene&cmd=Retrieve&dopt=full_report&list_uids=25923) | atlastin GTPase 3 |
| [Details](https://mirdb.org/cgi-bin/target_detail.cgi?targetID=2854506) | 233 | 93 | hsa-miR-20a-5p | [CD69](http://www.ncbi.nlm.nih.gov/entrez/query.fcgi?db=gene&cmd=Retrieve&dopt=full_report&list_uids=969) | CD69 molecule |
| [Details](https://mirdb.org/cgi-bin/target_detail.cgi?targetID=2854532) | 234 | 93 | hsa-miR-20a-5p | [ULK1](http://www.ncbi.nlm.nih.gov/entrez/query.fcgi?db=gene&cmd=Retrieve&dopt=full_report&list_uids=8408) | unc-51 like autophagy activating kinase 1 |
| [Details](https://mirdb.org/cgi-bin/target_detail.cgi?targetID=2854587) | 235 | 93 | hsa-miR-20a-5p | [DRD1](http://www.ncbi.nlm.nih.gov/entrez/query.fcgi?db=gene&cmd=Retrieve&dopt=full_report&list_uids=1812) | dopamine receptor D1 |
| [Details](https://mirdb.org/cgi-bin/target_detail.cgi?targetID=2854709) | 236 | 93 | hsa-miR-20a-5p | [ANO6](http://www.ncbi.nlm.nih.gov/entrez/query.fcgi?db=gene&cmd=Retrieve&dopt=full_report&list_uids=196527) | anoctamin 6 |
| [Details](https://mirdb.org/cgi-bin/target_detail.cgi?targetID=2854775) | 237 | 93 | hsa-miR-20a-5p | [BNC2](http://www.ncbi.nlm.nih.gov/entrez/query.fcgi?db=gene&cmd=Retrieve&dopt=full_report&list_uids=54796) | basonuclin 2 |
| [Details](https://mirdb.org/cgi-bin/target_detail.cgi?targetID=2854859) | 238 | 93 | hsa-miR-20a-5p | [CROT](http://www.ncbi.nlm.nih.gov/entrez/query.fcgi?db=gene&cmd=Retrieve&dopt=full_report&list_uids=54677) | carnitine O-octanoyltransferase |
| [Details](https://mirdb.org/cgi-bin/target_detail.cgi?targetID=2854909) | 239 | 93 | hsa-miR-20a-5p | [ERAP1](http://www.ncbi.nlm.nih.gov/entrez/query.fcgi?db=gene&cmd=Retrieve&dopt=full_report&list_uids=51752) | endoplasmic reticulum aminopeptidase 1 |
| [Details](https://mirdb.org/cgi-bin/target_detail.cgi?targetID=2854949) | 240 | 93 | hsa-miR-20a-5p | [RAP2C](http://www.ncbi.nlm.nih.gov/entrez/query.fcgi?db=gene&cmd=Retrieve&dopt=full_report&list_uids=57826) | RAP2C, member of RAS oncogene family |
| [Details](https://mirdb.org/cgi-bin/target_detail.cgi?targetID=2855010) | 241 | 93 | hsa-miR-20a-5p | [SUSD6](http://www.ncbi.nlm.nih.gov/entrez/query.fcgi?db=gene&cmd=Retrieve&dopt=full_report&list_uids=9766) | sushi domain containing 6 |
| [Details](https://mirdb.org/cgi-bin/target_detail.cgi?targetID=2855061) | 242 | 93 | hsa-miR-20a-5p | [PRDM6](http://www.ncbi.nlm.nih.gov/entrez/query.fcgi?db=gene&cmd=Retrieve&dopt=full_report&list_uids=93166) | PR/SET domain 6 |
| [Details](https://mirdb.org/cgi-bin/target_detail.cgi?targetID=2855135) | 243 | 93 | hsa-miR-20a-5p | [ARHGAP26](http://www.ncbi.nlm.nih.gov/entrez/query.fcgi?db=gene&cmd=Retrieve&dopt=full_report&list_uids=23092) | Rho GTPase activating protein 26 |
| [Details](https://mirdb.org/cgi-bin/target_detail.cgi?targetID=2855138) | 244 | 93 | hsa-miR-20a-5p | [NRIP3](http://www.ncbi.nlm.nih.gov/entrez/query.fcgi?db=gene&cmd=Retrieve&dopt=full_report&list_uids=56675) | nuclear receptor interacting protein 3 |
| [Details](https://mirdb.org/cgi-bin/target_detail.cgi?targetID=2855150) | 245 | 93 | hsa-miR-20a-5p | [SLAIN2](http://www.ncbi.nlm.nih.gov/entrez/query.fcgi?db=gene&cmd=Retrieve&dopt=full_report&list_uids=57606) | SLAIN motif family member 2 |
| [Details](https://mirdb.org/cgi-bin/target_detail.cgi?targetID=2855272) | 246 | 93 | hsa-miR-20a-5p | [ELK3](http://www.ncbi.nlm.nih.gov/entrez/query.fcgi?db=gene&cmd=Retrieve&dopt=full_report&list_uids=2004) | ELK3, ETS transcription factor |
| [Details](https://mirdb.org/cgi-bin/target_detail.cgi?targetID=2855292) | 247 | 93 | hsa-miR-20a-5p | [BEST3](http://www.ncbi.nlm.nih.gov/entrez/query.fcgi?db=gene&cmd=Retrieve&dopt=full_report&list_uids=144453) | bestrophin 3 |
| [Details](https://mirdb.org/cgi-bin/target_detail.cgi?targetID=2855312) | 248 | 93 | hsa-miR-20a-5p | [FAM189A1](http://www.ncbi.nlm.nih.gov/entrez/query.fcgi?db=gene&cmd=Retrieve&dopt=full_report&list_uids=23359) | family with sequence similarity 189 member A1 |
| [Details](https://mirdb.org/cgi-bin/target_detail.cgi?targetID=2855341) | 249 | 93 | hsa-miR-20a-5p | [CALD1](http://www.ncbi.nlm.nih.gov/entrez/query.fcgi?db=gene&cmd=Retrieve&dopt=full_report&list_uids=800) | caldesmon 1 |
| [Details](https://mirdb.org/cgi-bin/target_detail.cgi?targetID=2855430) | 250 | 93 | hsa-miR-20a-5p | [STRIP2](http://www.ncbi.nlm.nih.gov/entrez/query.fcgi?db=gene&cmd=Retrieve&dopt=full_report&list_uids=57464) | striatin interacting protein 2 |
| [Details](https://mirdb.org/cgi-bin/target_detail.cgi?targetID=2855441) | 251 | 93 | hsa-miR-20a-5p | [SLITRK3](http://www.ncbi.nlm.nih.gov/entrez/query.fcgi?db=gene&cmd=Retrieve&dopt=full_report&list_uids=22865) | SLIT and NTRK like family member 3 |
| [Details](https://mirdb.org/cgi-bin/target_detail.cgi?targetID=2854138) | 252 | 92 | hsa-miR-20a-5p | [NTN4](http://www.ncbi.nlm.nih.gov/entrez/query.fcgi?db=gene&cmd=Retrieve&dopt=full_report&list_uids=59277) | netrin 4 |
| [Details](https://mirdb.org/cgi-bin/target_detail.cgi?targetID=2854155) | 253 | 92 | hsa-miR-20a-5p | [TMEM168](http://www.ncbi.nlm.nih.gov/entrez/query.fcgi?db=gene&cmd=Retrieve&dopt=full_report&list_uids=64418) | transmembrane protein 168 |
| [Details](https://mirdb.org/cgi-bin/target_detail.cgi?targetID=2854179) | 254 | 92 | hsa-miR-20a-5p | [L3MBTL3](http://www.ncbi.nlm.nih.gov/entrez/query.fcgi?db=gene&cmd=Retrieve&dopt=full_report&list_uids=84456) | L3MBTL3, histone methyl-lysine binding protein |
| [Details](https://mirdb.org/cgi-bin/target_detail.cgi?targetID=2854313) | 255 | 92 | hsa-miR-20a-5p | [ETV1](http://www.ncbi.nlm.nih.gov/entrez/query.fcgi?db=gene&cmd=Retrieve&dopt=full_report&list_uids=2115) | ETS variant 1 |
| [Details](https://mirdb.org/cgi-bin/target_detail.cgi?targetID=2854407) | 256 | 92 | hsa-miR-20a-5p | [RRM2](http://www.ncbi.nlm.nih.gov/entrez/query.fcgi?db=gene&cmd=Retrieve&dopt=full_report&list_uids=6241) | ribonucleotide reductase regulatory subunit M2 |
| [Details](https://mirdb.org/cgi-bin/target_detail.cgi?targetID=2854419) | 257 | 92 | hsa-miR-20a-5p | [DNAJC16](http://www.ncbi.nlm.nih.gov/entrez/query.fcgi?db=gene&cmd=Retrieve&dopt=full_report&list_uids=23341) | DnaJ heat shock protein family (Hsp40) member C16 |
| [Details](https://mirdb.org/cgi-bin/target_detail.cgi?targetID=2854428) | 258 | 92 | hsa-miR-20a-5p | [TRPV6](http://www.ncbi.nlm.nih.gov/entrez/query.fcgi?db=gene&cmd=Retrieve&dopt=full_report&list_uids=55503) | transient receptor potential cation channel subfamily V member 6 |
| [Details](https://mirdb.org/cgi-bin/target_detail.cgi?targetID=2854439) | 259 | 92 | hsa-miR-20a-5p | [MAPRE3](http://www.ncbi.nlm.nih.gov/entrez/query.fcgi?db=gene&cmd=Retrieve&dopt=full_report&list_uids=22924) | microtubule associated protein RP/EB family member 3 |
| [Details](https://mirdb.org/cgi-bin/target_detail.cgi?targetID=2854484) | 260 | 92 | hsa-miR-20a-5p | [EGR2](http://www.ncbi.nlm.nih.gov/entrez/query.fcgi?db=gene&cmd=Retrieve&dopt=full_report&list_uids=1959) | early growth response 2 |
| [Details](https://mirdb.org/cgi-bin/target_detail.cgi?targetID=2854499) | 261 | 92 | hsa-miR-20a-5p | [TMEM64](http://www.ncbi.nlm.nih.gov/entrez/query.fcgi?db=gene&cmd=Retrieve&dopt=full_report&list_uids=169200) | transmembrane protein 64 |
| [Details](https://mirdb.org/cgi-bin/target_detail.cgi?targetID=2854525) | 262 | 92 | hsa-miR-20a-5p | [AP2B1](http://www.ncbi.nlm.nih.gov/entrez/query.fcgi?db=gene&cmd=Retrieve&dopt=full_report&list_uids=163) | adaptor related protein complex 2 subunit beta 1 |
| [Details](https://mirdb.org/cgi-bin/target_detail.cgi?targetID=2854576) | 263 | 92 | hsa-miR-20a-5p | [OSM](http://www.ncbi.nlm.nih.gov/entrez/query.fcgi?db=gene&cmd=Retrieve&dopt=full_report&list_uids=5008) | oncostatin M |
| [Details](https://mirdb.org/cgi-bin/target_detail.cgi?targetID=2854579) | 264 | 92 | hsa-miR-20a-5p | [ISM2](http://www.ncbi.nlm.nih.gov/entrez/query.fcgi?db=gene&cmd=Retrieve&dopt=full_report&list_uids=145501) | isthmin 2 |
| [Details](https://mirdb.org/cgi-bin/target_detail.cgi?targetID=2854596) | 265 | 92 | hsa-miR-20a-5p | [HPS5](http://www.ncbi.nlm.nih.gov/entrez/query.fcgi?db=gene&cmd=Retrieve&dopt=full_report&list_uids=11234) | HPS5, biogenesis of lysosomal organelles complex 2 subunit 2 |
| [Details](https://mirdb.org/cgi-bin/target_detail.cgi?targetID=2854611) | 266 | 92 | hsa-miR-20a-5p | [RUNX3](http://www.ncbi.nlm.nih.gov/entrez/query.fcgi?db=gene&cmd=Retrieve&dopt=full_report&list_uids=864) | runt related transcription factor 3 |
| [Details](https://mirdb.org/cgi-bin/target_detail.cgi?targetID=2854621) | 267 | 92 | hsa-miR-20a-5p | [IGSF10](http://www.ncbi.nlm.nih.gov/entrez/query.fcgi?db=gene&cmd=Retrieve&dopt=full_report&list_uids=285313) | immunoglobulin superfamily member 10 |
| [Details](https://mirdb.org/cgi-bin/target_detail.cgi?targetID=2854657) | 268 | 92 | hsa-miR-20a-5p | [RACGAP1](http://www.ncbi.nlm.nih.gov/entrez/query.fcgi?db=gene&cmd=Retrieve&dopt=full_report&list_uids=29127) | Rac GTPase activating protein 1 |
| [Details](https://mirdb.org/cgi-bin/target_detail.cgi?targetID=2854664) | 269 | 92 | hsa-miR-20a-5p | [URI1](http://www.ncbi.nlm.nih.gov/entrez/query.fcgi?db=gene&cmd=Retrieve&dopt=full_report&list_uids=8725) | URI1, prefoldin like chaperone |
| [Details](https://mirdb.org/cgi-bin/target_detail.cgi?targetID=2854803) | 270 | 92 | hsa-miR-20a-5p | [FAM102A](http://www.ncbi.nlm.nih.gov/entrez/query.fcgi?db=gene&cmd=Retrieve&dopt=full_report&list_uids=399665) | family with sequence similarity 102 member A |
| [Details](https://mirdb.org/cgi-bin/target_detail.cgi?targetID=2854817) | 271 | 92 | hsa-miR-20a-5p | [TGM2](http://www.ncbi.nlm.nih.gov/entrez/query.fcgi?db=gene&cmd=Retrieve&dopt=full_report&list_uids=7052) | transglutaminase 2 |
| [Details](https://mirdb.org/cgi-bin/target_detail.cgi?targetID=2854925) | 272 | 92 | hsa-miR-20a-5p | [LASP1](http://www.ncbi.nlm.nih.gov/entrez/query.fcgi?db=gene&cmd=Retrieve&dopt=full_report&list_uids=3927) | LIM and SH3 protein 1 |
| [Details](https://mirdb.org/cgi-bin/target_detail.cgi?targetID=2854926) | 273 | 92 | hsa-miR-20a-5p | [OCRL](http://www.ncbi.nlm.nih.gov/entrez/query.fcgi?db=gene&cmd=Retrieve&dopt=full_report&list_uids=4952) | OCRL, inositol polyphosphate-5-phosphatase |
| [Details](https://mirdb.org/cgi-bin/target_detail.cgi?targetID=2854927) | 274 | 92 | hsa-miR-20a-5p | [SUCO](http://www.ncbi.nlm.nih.gov/entrez/query.fcgi?db=gene&cmd=Retrieve&dopt=full_report&list_uids=51430) | SUN domain containing ossification factor |
| [Details](https://mirdb.org/cgi-bin/target_detail.cgi?targetID=2854931) | 275 | 92 | hsa-miR-20a-5p | [TNKS2](http://www.ncbi.nlm.nih.gov/entrez/query.fcgi?db=gene&cmd=Retrieve&dopt=full_report&list_uids=80351) | tankyrase 2 |
| [Details](https://mirdb.org/cgi-bin/target_detail.cgi?targetID=2854945) | 276 | 92 | hsa-miR-20a-5p | [CENPQ](http://www.ncbi.nlm.nih.gov/entrez/query.fcgi?db=gene&cmd=Retrieve&dopt=full_report&list_uids=55166) | centromere protein Q |
| [Details](https://mirdb.org/cgi-bin/target_detail.cgi?targetID=2854980) | 277 | 92 | hsa-miR-20a-5p | [ACSL4](http://www.ncbi.nlm.nih.gov/entrez/query.fcgi?db=gene&cmd=Retrieve&dopt=full_report&list_uids=2182) | acyl-CoA synthetase long chain family member 4 |
| [Details](https://mirdb.org/cgi-bin/target_detail.cgi?targetID=2855009) | 278 | 92 | hsa-miR-20a-5p | [RAB11FIP1](http://www.ncbi.nlm.nih.gov/entrez/query.fcgi?db=gene&cmd=Retrieve&dopt=full_report&list_uids=80223) | RAB11 family interacting protein 1 |
| [Details](https://mirdb.org/cgi-bin/target_detail.cgi?targetID=2855052) | 279 | 92 | hsa-miR-20a-5p | [SESN3](http://www.ncbi.nlm.nih.gov/entrez/query.fcgi?db=gene&cmd=Retrieve&dopt=full_report&list_uids=143686) | sestrin 3 |
| [Details](https://mirdb.org/cgi-bin/target_detail.cgi?targetID=2855109) | 280 | 92 | hsa-miR-20a-5p | [SRCIN1](http://www.ncbi.nlm.nih.gov/entrez/query.fcgi?db=gene&cmd=Retrieve&dopt=full_report&list_uids=80725) | SRC kinase signaling inhibitor 1 |
| [Details](https://mirdb.org/cgi-bin/target_detail.cgi?targetID=2855186) | 281 | 92 | hsa-miR-20a-5p | [RGMB](http://www.ncbi.nlm.nih.gov/entrez/query.fcgi?db=gene&cmd=Retrieve&dopt=full_report&list_uids=285704) | repulsive guidance molecule BMP co-receptor b |
| [Details](https://mirdb.org/cgi-bin/target_detail.cgi?targetID=2855216) | 282 | 92 | hsa-miR-20a-5p | [HECTD2](http://www.ncbi.nlm.nih.gov/entrez/query.fcgi?db=gene&cmd=Retrieve&dopt=full_report&list_uids=143279) | HECT domain E3 ubiquitin protein ligase 2 |
| [Details](https://mirdb.org/cgi-bin/target_detail.cgi?targetID=2855245) | 283 | 92 | hsa-miR-20a-5p | [SSH2](http://www.ncbi.nlm.nih.gov/entrez/query.fcgi?db=gene&cmd=Retrieve&dopt=full_report&list_uids=85464) | slingshot protein phosphatase 2 |
| [Details](https://mirdb.org/cgi-bin/target_detail.cgi?targetID=2855267) | 284 | 92 | hsa-miR-20a-5p | [UNC80](http://www.ncbi.nlm.nih.gov/entrez/query.fcgi?db=gene&cmd=Retrieve&dopt=full_report&list_uids=285175) | unc-80 homolog, NALCN channel complex subunit |
| [Details](https://mirdb.org/cgi-bin/target_detail.cgi?targetID=2855277) | 285 | 92 | hsa-miR-20a-5p | [CEP120](http://www.ncbi.nlm.nih.gov/entrez/query.fcgi?db=gene&cmd=Retrieve&dopt=full_report&list_uids=153241) | centrosomal protein 120 |
| [Details](https://mirdb.org/cgi-bin/target_detail.cgi?targetID=2855333) | 286 | 92 | hsa-miR-20a-5p | [LRP8](http://www.ncbi.nlm.nih.gov/entrez/query.fcgi?db=gene&cmd=Retrieve&dopt=full_report&list_uids=7804) | LDL receptor related protein 8 |
| [Details](https://mirdb.org/cgi-bin/target_detail.cgi?targetID=2855334) | 287 | 92 | hsa-miR-20a-5p | [LRCH1](http://www.ncbi.nlm.nih.gov/entrez/query.fcgi?db=gene&cmd=Retrieve&dopt=full_report&list_uids=23143) | leucine rich repeats and calponin homology domain containing 1 |
| [Details](https://mirdb.org/cgi-bin/target_detail.cgi?targetID=2855337) | 288 | 92 | hsa-miR-20a-5p | [SIKE1](http://www.ncbi.nlm.nih.gov/entrez/query.fcgi?db=gene&cmd=Retrieve&dopt=full_report&list_uids=80143) | suppressor of IKBKE 1 |
| [Details](https://mirdb.org/cgi-bin/target_detail.cgi?targetID=2855373) | 289 | 92 | hsa-miR-20a-5p | [C14orf28](http://www.ncbi.nlm.nih.gov/entrez/query.fcgi?db=gene&cmd=Retrieve&dopt=full_report&list_uids=122525) | chromosome 14 open reading frame 28 |
| [Details](https://mirdb.org/cgi-bin/target_detail.cgi?targetID=2855399) | 290 | 92 | hsa-miR-20a-5p | [CTSK](http://www.ncbi.nlm.nih.gov/entrez/query.fcgi?db=gene&cmd=Retrieve&dopt=full_report&list_uids=1513) | cathepsin K |
| [Details](https://mirdb.org/cgi-bin/target_detail.cgi?targetID=2855426) | 291 | 92 | hsa-miR-20a-5p | [AGFG1](http://www.ncbi.nlm.nih.gov/entrez/query.fcgi?db=gene&cmd=Retrieve&dopt=full_report&list_uids=3267) | ArfGAP with FG repeats 1 |
| [Details](https://mirdb.org/cgi-bin/target_detail.cgi?targetID=2854093) | 292 | 91 | hsa-miR-20a-5p | [RAPH1](http://www.ncbi.nlm.nih.gov/entrez/query.fcgi?db=gene&cmd=Retrieve&dopt=full_report&list_uids=65059) | Ras association (RalGDS/AF-6) and pleckstrin homology domains 1 |
| [Details](https://mirdb.org/cgi-bin/target_detail.cgi?targetID=2854109) | 293 | 91 | hsa-miR-20a-5p | [DERL2](http://www.ncbi.nlm.nih.gov/entrez/query.fcgi?db=gene&cmd=Retrieve&dopt=full_report&list_uids=51009) | derlin 2 |
| [Details](https://mirdb.org/cgi-bin/target_detail.cgi?targetID=2854144) | 294 | 91 | hsa-miR-20a-5p | [KLF11](http://www.ncbi.nlm.nih.gov/entrez/query.fcgi?db=gene&cmd=Retrieve&dopt=full_report&list_uids=8462) | Kruppel like factor 11 |
| [Details](https://mirdb.org/cgi-bin/target_detail.cgi?targetID=2854173) | 295 | 91 | hsa-miR-20a-5p | [PCDHA8](http://www.ncbi.nlm.nih.gov/entrez/query.fcgi?db=gene&cmd=Retrieve&dopt=full_report&list_uids=56140) | protocadherin alpha 8 |
| [Details](https://mirdb.org/cgi-bin/target_detail.cgi?targetID=2854174) | 296 | 91 | hsa-miR-20a-5p | [PCDHAC1](http://www.ncbi.nlm.nih.gov/entrez/query.fcgi?db=gene&cmd=Retrieve&dopt=full_report&list_uids=56135) | protocadherin alpha subfamily C, 1 |
| [Details](https://mirdb.org/cgi-bin/target_detail.cgi?targetID=2854209) | 297 | 91 | hsa-miR-20a-5p | [GNB5](http://www.ncbi.nlm.nih.gov/entrez/query.fcgi?db=gene&cmd=Retrieve&dopt=full_report&list_uids=10681) | G protein subunit beta 5 |
| [Details](https://mirdb.org/cgi-bin/target_detail.cgi?targetID=2854233) | 298 | 91 | hsa-miR-20a-5p | [CCDC71L](http://www.ncbi.nlm.nih.gov/entrez/query.fcgi?db=gene&cmd=Retrieve&dopt=full_report&list_uids=168455) | coiled-coil domain containing 71 like |
| [Details](https://mirdb.org/cgi-bin/target_detail.cgi?targetID=2854275) | 299 | 91 | hsa-miR-20a-5p | [RB1CC1](http://www.ncbi.nlm.nih.gov/entrez/query.fcgi?db=gene&cmd=Retrieve&dopt=full_report&list_uids=9821) | RB1 inducible coiled-coil 1 |
| [Details](https://mirdb.org/cgi-bin/target_detail.cgi?targetID=2854292) | 300 | 91 | hsa-miR-20a-5p | [ARHGAP1](http://www.ncbi.nlm.nih.gov/entrez/query.fcgi?db=gene&cmd=Retrieve&dopt=full_report&list_uids=392) | Rho GTPase activating protein 1 |
| [Details](https://mirdb.org/cgi-bin/target_detail.cgi?targetID=2854318) | 301 | 91 | hsa-miR-20a-5p | [TET1](http://www.ncbi.nlm.nih.gov/entrez/query.fcgi?db=gene&cmd=Retrieve&dopt=full_report&list_uids=80312) | tet methylcytosine dioxygenase 1 |
| [Details](https://mirdb.org/cgi-bin/target_detail.cgi?targetID=2854379) | 302 | 91 | hsa-miR-20a-5p | [PCDHAC2](http://www.ncbi.nlm.nih.gov/entrez/query.fcgi?db=gene&cmd=Retrieve&dopt=full_report&list_uids=56134) | protocadherin alpha subfamily C, 2 |
| [Details](https://mirdb.org/cgi-bin/target_detail.cgi?targetID=2854386) | 303 | 91 | hsa-miR-20a-5p | [PCDHA13](http://www.ncbi.nlm.nih.gov/entrez/query.fcgi?db=gene&cmd=Retrieve&dopt=full_report&list_uids=56136) | protocadherin alpha 13 |
| [Details](https://mirdb.org/cgi-bin/target_detail.cgi?targetID=2854416) | 304 | 91 | hsa-miR-20a-5p | [KIAA1191](http://www.ncbi.nlm.nih.gov/entrez/query.fcgi?db=gene&cmd=Retrieve&dopt=full_report&list_uids=57179) | KIAA1191 |
| [Details](https://mirdb.org/cgi-bin/target_detail.cgi?targetID=2854473) | 305 | 91 | hsa-miR-20a-5p | [ZFAND4](http://www.ncbi.nlm.nih.gov/entrez/query.fcgi?db=gene&cmd=Retrieve&dopt=full_report&list_uids=93550) | zinc finger AN1-type containing 4 |
| [Details](https://mirdb.org/cgi-bin/target_detail.cgi?targetID=2854509) | 306 | 91 | hsa-miR-20a-5p | [ANKFY1](http://www.ncbi.nlm.nih.gov/entrez/query.fcgi?db=gene&cmd=Retrieve&dopt=full_report&list_uids=51479) | ankyrin repeat and FYVE domain containing 1 |
| [Details](https://mirdb.org/cgi-bin/target_detail.cgi?targetID=2854514) | 307 | 91 | hsa-miR-20a-5p | [P2RX4](http://www.ncbi.nlm.nih.gov/entrez/query.fcgi?db=gene&cmd=Retrieve&dopt=full_report&list_uids=5025) | purinergic receptor P2X 4 |
| [Details](https://mirdb.org/cgi-bin/target_detail.cgi?targetID=2854555) | 308 | 91 | hsa-miR-20a-5p | [ERC1](http://www.ncbi.nlm.nih.gov/entrez/query.fcgi?db=gene&cmd=Retrieve&dopt=full_report&list_uids=23085) | ELKS/RAB6-interacting/CAST family member 1 |
| [Details](https://mirdb.org/cgi-bin/target_detail.cgi?targetID=2854591) | 309 | 91 | hsa-miR-20a-5p | [PCDHA10](http://www.ncbi.nlm.nih.gov/entrez/query.fcgi?db=gene&cmd=Retrieve&dopt=full_report&list_uids=56139) | protocadherin alpha 10 |
| [Details](https://mirdb.org/cgi-bin/target_detail.cgi?targetID=2854592) | 310 | 91 | hsa-miR-20a-5p | [C7orf43](http://www.ncbi.nlm.nih.gov/entrez/query.fcgi?db=gene&cmd=Retrieve&dopt=full_report&list_uids=55262) | chromosome 7 open reading frame 43 |
| [Details](https://mirdb.org/cgi-bin/target_detail.cgi?targetID=2854608) | 311 | 91 | hsa-miR-20a-5p | [PCDHA5](http://www.ncbi.nlm.nih.gov/entrez/query.fcgi?db=gene&cmd=Retrieve&dopt=full_report&list_uids=56143) | protocadherin alpha 5 |
| [Details](https://mirdb.org/cgi-bin/target_detail.cgi?targetID=2854656) | 312 | 91 | hsa-miR-20a-5p | [FNDC3B](http://www.ncbi.nlm.nih.gov/entrez/query.fcgi?db=gene&cmd=Retrieve&dopt=full_report&list_uids=64778) | fibronectin type III domain containing 3B |
| [Details](https://mirdb.org/cgi-bin/target_detail.cgi?targetID=2854710) | 313 | 91 | hsa-miR-20a-5p | [PCDHA6](http://www.ncbi.nlm.nih.gov/entrez/query.fcgi?db=gene&cmd=Retrieve&dopt=full_report&list_uids=56142) | protocadherin alpha 6 |
| [Details](https://mirdb.org/cgi-bin/target_detail.cgi?targetID=2854718) | 314 | 91 | hsa-miR-20a-5p | [NBEA](http://www.ncbi.nlm.nih.gov/entrez/query.fcgi?db=gene&cmd=Retrieve&dopt=full_report&list_uids=26960) | neurobeachin |
| [Details](https://mirdb.org/cgi-bin/target_detail.cgi?targetID=2854755) | 315 | 91 | hsa-miR-20a-5p | [PCDHA11](http://www.ncbi.nlm.nih.gov/entrez/query.fcgi?db=gene&cmd=Retrieve&dopt=full_report&list_uids=56138) | protocadherin alpha 11 |
| [Details](https://mirdb.org/cgi-bin/target_detail.cgi?targetID=2854759) | 316 | 91 | hsa-miR-20a-5p | [TRIM3](http://www.ncbi.nlm.nih.gov/entrez/query.fcgi?db=gene&cmd=Retrieve&dopt=full_report&list_uids=10612) | tripartite motif containing 3 |
| [Details](https://mirdb.org/cgi-bin/target_detail.cgi?targetID=2854806) | 317 | 91 | hsa-miR-20a-5p | [PCDHA3](http://www.ncbi.nlm.nih.gov/entrez/query.fcgi?db=gene&cmd=Retrieve&dopt=full_report&list_uids=56145) | protocadherin alpha 3 |
| [Details](https://mirdb.org/cgi-bin/target_detail.cgi?targetID=2854824) | 318 | 91 | hsa-miR-20a-5p | [DAB2](http://www.ncbi.nlm.nih.gov/entrez/query.fcgi?db=gene&cmd=Retrieve&dopt=full_report&list_uids=1601) | DAB2, clathrin adaptor protein |
| [Details](https://mirdb.org/cgi-bin/target_detail.cgi?targetID=2854828) | 319 | 91 | hsa-miR-20a-5p | [USP28](http://www.ncbi.nlm.nih.gov/entrez/query.fcgi?db=gene&cmd=Retrieve&dopt=full_report&list_uids=57646) | ubiquitin specific peptidase 28 |
| [Details](https://mirdb.org/cgi-bin/target_detail.cgi?targetID=2854855) | 320 | 91 | hsa-miR-20a-5p | [ZBTB33](http://www.ncbi.nlm.nih.gov/entrez/query.fcgi?db=gene&cmd=Retrieve&dopt=full_report&list_uids=10009) | zinc finger and BTB domain containing 33 |
| [Details](https://mirdb.org/cgi-bin/target_detail.cgi?targetID=2854928) | 321 | 91 | hsa-miR-20a-5p | [APCDD1](http://www.ncbi.nlm.nih.gov/entrez/query.fcgi?db=gene&cmd=Retrieve&dopt=full_report&list_uids=147495) | APC down-regulated 1 |
| [Details](https://mirdb.org/cgi-bin/target_detail.cgi?targetID=2855032) | 322 | 91 | hsa-miR-20a-5p | [RPS6KA4](http://www.ncbi.nlm.nih.gov/entrez/query.fcgi?db=gene&cmd=Retrieve&dopt=full_report&list_uids=8986) | ribosomal protein S6 kinase A4 |
| [Details](https://mirdb.org/cgi-bin/target_detail.cgi?targetID=2855063) | 323 | 91 | hsa-miR-20a-5p | [SLC16A9](http://www.ncbi.nlm.nih.gov/entrez/query.fcgi?db=gene&cmd=Retrieve&dopt=full_report&list_uids=220963) | solute carrier family 16 member 9 |
| [Details](https://mirdb.org/cgi-bin/target_detail.cgi?targetID=2855066) | 324 | 91 | hsa-miR-20a-5p | [CC2D1A](http://www.ncbi.nlm.nih.gov/entrez/query.fcgi?db=gene&cmd=Retrieve&dopt=full_report&list_uids=54862) | coiled-coil and C2 domain containing 1A |
| [Details](https://mirdb.org/cgi-bin/target_detail.cgi?targetID=2855091) | 325 | 91 | hsa-miR-20a-5p | [WDFY3](http://www.ncbi.nlm.nih.gov/entrez/query.fcgi?db=gene&cmd=Retrieve&dopt=full_report&list_uids=23001) | WD repeat and FYVE domain containing 3 |
| [Details](https://mirdb.org/cgi-bin/target_detail.cgi?targetID=2855094) | 326 | 91 | hsa-miR-20a-5p | [FEM1C](http://www.ncbi.nlm.nih.gov/entrez/query.fcgi?db=gene&cmd=Retrieve&dopt=full_report&list_uids=56929) | fem-1 homolog C |
| [Details](https://mirdb.org/cgi-bin/target_detail.cgi?targetID=2855108) | 327 | 91 | hsa-miR-20a-5p | [PCDHA4](http://www.ncbi.nlm.nih.gov/entrez/query.fcgi?db=gene&cmd=Retrieve&dopt=full_report&list_uids=56144) | protocadherin alpha 4 |
| [Details](https://mirdb.org/cgi-bin/target_detail.cgi?targetID=2855141) | 328 | 91 | hsa-miR-20a-5p | [TIAM1](http://www.ncbi.nlm.nih.gov/entrez/query.fcgi?db=gene&cmd=Retrieve&dopt=full_report&list_uids=7074) | T cell lymphoma invasion and metastasis 1 |
| [Details](https://mirdb.org/cgi-bin/target_detail.cgi?targetID=2855156) | 329 | 91 | hsa-miR-20a-5p | [LYST](http://www.ncbi.nlm.nih.gov/entrez/query.fcgi?db=gene&cmd=Retrieve&dopt=full_report&list_uids=1130) | lysosomal trafficking regulator |
| [Details](https://mirdb.org/cgi-bin/target_detail.cgi?targetID=2855161) | 330 | 91 | hsa-miR-20a-5p | [RAB8B](http://www.ncbi.nlm.nih.gov/entrez/query.fcgi?db=gene&cmd=Retrieve&dopt=full_report&list_uids=51762) | RAB8B, member RAS oncogene family |
| [Details](https://mirdb.org/cgi-bin/target_detail.cgi?targetID=2855170) | 331 | 91 | hsa-miR-20a-5p | [IL6ST](http://www.ncbi.nlm.nih.gov/entrez/query.fcgi?db=gene&cmd=Retrieve&dopt=full_report&list_uids=3572) | interleukin 6 signal transducer |
| [Details](https://mirdb.org/cgi-bin/target_detail.cgi?targetID=2855172) | 332 | 91 | hsa-miR-20a-5p | [PCDHA1](http://www.ncbi.nlm.nih.gov/entrez/query.fcgi?db=gene&cmd=Retrieve&dopt=full_report&list_uids=56147) | protocadherin alpha 1 |
| [Details](https://mirdb.org/cgi-bin/target_detail.cgi?targetID=2855200) | 333 | 91 | hsa-miR-20a-5p | [STK38](http://www.ncbi.nlm.nih.gov/entrez/query.fcgi?db=gene&cmd=Retrieve&dopt=full_report&list_uids=11329) | serine/threonine kinase 38 |
| [Details](https://mirdb.org/cgi-bin/target_detail.cgi?targetID=2855215) | 334 | 91 | hsa-miR-20a-5p | [LDLR](http://www.ncbi.nlm.nih.gov/entrez/query.fcgi?db=gene&cmd=Retrieve&dopt=full_report&list_uids=3949) | low density lipoprotein receptor |
| [Details](https://mirdb.org/cgi-bin/target_detail.cgi?targetID=2855233) | 335 | 91 | hsa-miR-20a-5p | [CERCAM](http://www.ncbi.nlm.nih.gov/entrez/query.fcgi?db=gene&cmd=Retrieve&dopt=full_report&list_uids=51148) | cerebral endothelial cell adhesion molecule |
| [Details](https://mirdb.org/cgi-bin/target_detail.cgi?targetID=2855234) | 336 | 91 | hsa-miR-20a-5p | [THRA](http://www.ncbi.nlm.nih.gov/entrez/query.fcgi?db=gene&cmd=Retrieve&dopt=full_report&list_uids=7067) | thyroid hormone receptor alpha |
| [Details](https://mirdb.org/cgi-bin/target_detail.cgi?targetID=2855242) | 337 | 91 | hsa-miR-20a-5p | [PRR15](http://www.ncbi.nlm.nih.gov/entrez/query.fcgi?db=gene&cmd=Retrieve&dopt=full_report&list_uids=222171) | proline rich 15 |
| [Details](https://mirdb.org/cgi-bin/target_detail.cgi?targetID=2855249) | 338 | 91 | hsa-miR-20a-5p | [LRRC55](http://www.ncbi.nlm.nih.gov/entrez/query.fcgi?db=gene&cmd=Retrieve&dopt=full_report&list_uids=219527) | leucine rich repeat containing 55 |
| [Details](https://mirdb.org/cgi-bin/target_detail.cgi?targetID=2855260) | 339 | 91 | hsa-miR-20a-5p | [SMOC1](http://www.ncbi.nlm.nih.gov/entrez/query.fcgi?db=gene&cmd=Retrieve&dopt=full_report&list_uids=64093) | SPARC related modular calcium binding 1 |
| [Details](https://mirdb.org/cgi-bin/target_detail.cgi?targetID=2855293) | 340 | 91 | hsa-miR-20a-5p | [PCDHA7](http://www.ncbi.nlm.nih.gov/entrez/query.fcgi?db=gene&cmd=Retrieve&dopt=full_report&list_uids=56141) | protocadherin alpha 7 |
| [Details](https://mirdb.org/cgi-bin/target_detail.cgi?targetID=2855314) | 341 | 91 | hsa-miR-20a-5p | [PCDHA2](http://www.ncbi.nlm.nih.gov/entrez/query.fcgi?db=gene&cmd=Retrieve&dopt=full_report&list_uids=56146) | protocadherin alpha 2 |
| [Details](https://mirdb.org/cgi-bin/target_detail.cgi?targetID=2855381) | 342 | 91 | hsa-miR-20a-5p | [MAP10](http://www.ncbi.nlm.nih.gov/entrez/query.fcgi?db=gene&cmd=Retrieve&dopt=full_report&list_uids=54627) | microtubule associated protein 10 |
| [Details](https://mirdb.org/cgi-bin/target_detail.cgi?targetID=2855385) | 343 | 91 | hsa-miR-20a-5p | [PCDHA12](http://www.ncbi.nlm.nih.gov/entrez/query.fcgi?db=gene&cmd=Retrieve&dopt=full_report&list_uids=56137) | protocadherin alpha 12 |
| [Details](https://mirdb.org/cgi-bin/target_detail.cgi?targetID=2855437) | 344 | 91 | hsa-miR-20a-5p | [UBE3C](http://www.ncbi.nlm.nih.gov/entrez/query.fcgi?db=gene&cmd=Retrieve&dopt=full_report&list_uids=9690) | ubiquitin protein ligase E3C |
| [Details](https://mirdb.org/cgi-bin/target_detail.cgi?targetID=2854131) | 345 | 90 | hsa-miR-20a-5p | [SLC33A1](http://www.ncbi.nlm.nih.gov/entrez/query.fcgi?db=gene&cmd=Retrieve&dopt=full_report&list_uids=9197) | solute carrier family 33 member 1 |
| [Details](https://mirdb.org/cgi-bin/target_detail.cgi?targetID=2854137) | 346 | 90 | hsa-miR-20a-5p | [ENTPD4](http://www.ncbi.nlm.nih.gov/entrez/query.fcgi?db=gene&cmd=Retrieve&dopt=full_report&list_uids=9583) | ectonucleoside triphosphate diphosphohydrolase 4 |
| [Details](https://mirdb.org/cgi-bin/target_detail.cgi?targetID=2854159) | 347 | 90 | hsa-miR-20a-5p | [S1PR1](http://www.ncbi.nlm.nih.gov/entrez/query.fcgi?db=gene&cmd=Retrieve&dopt=full_report&list_uids=1901) | sphingosine-1-phosphate receptor 1 |
| [Details](https://mirdb.org/cgi-bin/target_detail.cgi?targetID=2854262) | 348 | 90 | hsa-miR-20a-5p | [GOLGA1](http://www.ncbi.nlm.nih.gov/entrez/query.fcgi?db=gene&cmd=Retrieve&dopt=full_report&list_uids=2800) | golgin A1 |
| [Details](https://mirdb.org/cgi-bin/target_detail.cgi?targetID=2854285) | 349 | 90 | hsa-miR-20a-5p | [BICC1](http://www.ncbi.nlm.nih.gov/entrez/query.fcgi?db=gene&cmd=Retrieve&dopt=full_report&list_uids=80114) | BicC family RNA binding protein 1 |
| [Details](https://mirdb.org/cgi-bin/target_detail.cgi?targetID=2854288) | 350 | 90 | hsa-miR-20a-5p | [ARHGEF10](http://www.ncbi.nlm.nih.gov/entrez/query.fcgi?db=gene&cmd=Retrieve&dopt=full_report&list_uids=9639) | Rho guanine nucleotide exchange factor 10 |
| [Details](https://mirdb.org/cgi-bin/target_detail.cgi?targetID=2854368) | 351 | 90 | hsa-miR-20a-5p | [KIF23](http://www.ncbi.nlm.nih.gov/entrez/query.fcgi?db=gene&cmd=Retrieve&dopt=full_report&list_uids=9493) | kinesin family member 23 |
| [Details](https://mirdb.org/cgi-bin/target_detail.cgi?targetID=2854374) | 352 | 90 | hsa-miR-20a-5p | [SLC4A8](http://www.ncbi.nlm.nih.gov/entrez/query.fcgi?db=gene&cmd=Retrieve&dopt=full_report&list_uids=9498) | solute carrier family 4 member 8 |
| [Details](https://mirdb.org/cgi-bin/target_detail.cgi?targetID=2854415) | 353 | 90 | hsa-miR-20a-5p | [CSRNP3](http://www.ncbi.nlm.nih.gov/entrez/query.fcgi?db=gene&cmd=Retrieve&dopt=full_report&list_uids=80034) | cysteine and serine rich nuclear protein 3 |
| [Details](https://mirdb.org/cgi-bin/target_detail.cgi?targetID=2854443) | 354 | 90 | hsa-miR-20a-5p | [CRY2](http://www.ncbi.nlm.nih.gov/entrez/query.fcgi?db=gene&cmd=Retrieve&dopt=full_report&list_uids=1408) | cryptochrome circadian regulator 2 |
| [Details](https://mirdb.org/cgi-bin/target_detail.cgi?targetID=2854460) | 355 | 90 | hsa-miR-20a-5p | [PXK](http://www.ncbi.nlm.nih.gov/entrez/query.fcgi?db=gene&cmd=Retrieve&dopt=full_report&list_uids=54899) | PX domain containing serine/threonine kinase like |
| [Details](https://mirdb.org/cgi-bin/target_detail.cgi?targetID=2854542) | 356 | 90 | hsa-miR-20a-5p | [ATP12A](http://www.ncbi.nlm.nih.gov/entrez/query.fcgi?db=gene&cmd=Retrieve&dopt=full_report&list_uids=479) | ATPase H+/K+ transporting non-gastric alpha2 subunit |
| [Details](https://mirdb.org/cgi-bin/target_detail.cgi?targetID=2854599) | 357 | 90 | hsa-miR-20a-5p | [MAPK1](http://www.ncbi.nlm.nih.gov/entrez/query.fcgi?db=gene&cmd=Retrieve&dopt=full_report&list_uids=5594) | mitogen-activated protein kinase 1 |
| [Details](https://mirdb.org/cgi-bin/target_detail.cgi?targetID=2854610) | 358 | 90 | hsa-miR-20a-5p | [CHP2](http://www.ncbi.nlm.nih.gov/entrez/query.fcgi?db=gene&cmd=Retrieve&dopt=full_report&list_uids=63928) | calcineurin like EF-hand protein 2 |
| [Details](https://mirdb.org/cgi-bin/target_detail.cgi?targetID=2854612) | 359 | 90 | hsa-miR-20a-5p | [MTMR3](http://www.ncbi.nlm.nih.gov/entrez/query.fcgi?db=gene&cmd=Retrieve&dopt=full_report&list_uids=8897) | myotubularin related protein 3 |
| [Details](https://mirdb.org/cgi-bin/target_detail.cgi?targetID=2854649) | 360 | 90 | hsa-miR-20a-5p | [CXCL6](http://www.ncbi.nlm.nih.gov/entrez/query.fcgi?db=gene&cmd=Retrieve&dopt=full_report&list_uids=6372) | C-X-C motif chemokine ligand 6 |
| [Details](https://mirdb.org/cgi-bin/target_detail.cgi?targetID=2854706) | 361 | 90 | hsa-miR-20a-5p | [SLC16A6](http://www.ncbi.nlm.nih.gov/entrez/query.fcgi?db=gene&cmd=Retrieve&dopt=full_report&list_uids=9120) | solute carrier family 16 member 6 |
| [Details](https://mirdb.org/cgi-bin/target_detail.cgi?targetID=2854724) | 362 | 90 | hsa-miR-20a-5p | [CREB1](http://www.ncbi.nlm.nih.gov/entrez/query.fcgi?db=gene&cmd=Retrieve&dopt=full_report&list_uids=1385) | cAMP responsive element binding protein 1 |
| [Details](https://mirdb.org/cgi-bin/target_detail.cgi?targetID=2854745) | 363 | 90 | hsa-miR-20a-5p | [ARHGEF18](http://www.ncbi.nlm.nih.gov/entrez/query.fcgi?db=gene&cmd=Retrieve&dopt=full_report&list_uids=23370) | Rho/Rac guanine nucleotide exchange factor 18 |
| [Details](https://mirdb.org/cgi-bin/target_detail.cgi?targetID=2854767) | 364 | 90 | hsa-miR-20a-5p | [NACC2](http://www.ncbi.nlm.nih.gov/entrez/query.fcgi?db=gene&cmd=Retrieve&dopt=full_report&list_uids=138151) | NACC family member 2 |
| [Details](https://mirdb.org/cgi-bin/target_detail.cgi?targetID=2854836) | 365 | 90 | hsa-miR-20a-5p | [ORMDL3](http://www.ncbi.nlm.nih.gov/entrez/query.fcgi?db=gene&cmd=Retrieve&dopt=full_report&list_uids=94103) | ORMDL sphingolipid biosynthesis regulator 3 |
| [Details](https://mirdb.org/cgi-bin/target_detail.cgi?targetID=2854906) | 366 | 90 | hsa-miR-20a-5p | [GPATCH2](http://www.ncbi.nlm.nih.gov/entrez/query.fcgi?db=gene&cmd=Retrieve&dopt=full_report&list_uids=55105) | G-patch domain containing 2 |
| [Details](https://mirdb.org/cgi-bin/target_detail.cgi?targetID=2854911) | 367 | 90 | hsa-miR-20a-5p | [RGMA](http://www.ncbi.nlm.nih.gov/entrez/query.fcgi?db=gene&cmd=Retrieve&dopt=full_report&list_uids=56963) | repulsive guidance molecule BMP co-receptor a |
| [Details](https://mirdb.org/cgi-bin/target_detail.cgi?targetID=2854969) | 368 | 90 | hsa-miR-20a-5p | [SQSTM1](http://www.ncbi.nlm.nih.gov/entrez/query.fcgi?db=gene&cmd=Retrieve&dopt=full_report&list_uids=8878) | sequestosome 1 |
| [Details](https://mirdb.org/cgi-bin/target_detail.cgi?targetID=2854989) | 369 | 90 | hsa-miR-20a-5p | [WDR37](http://www.ncbi.nlm.nih.gov/entrez/query.fcgi?db=gene&cmd=Retrieve&dopt=full_report&list_uids=22884) | WD repeat domain 37 |
| [Details](https://mirdb.org/cgi-bin/target_detail.cgi?targetID=2855003) | 370 | 90 | hsa-miR-20a-5p | [AFG1L](http://www.ncbi.nlm.nih.gov/entrez/query.fcgi?db=gene&cmd=Retrieve&dopt=full_report&list_uids=246269) | AFG1 like ATPase |
| [Details](https://mirdb.org/cgi-bin/target_detail.cgi?targetID=2855111) | 371 | 90 | hsa-miR-20a-5p | [AMER2](http://www.ncbi.nlm.nih.gov/entrez/query.fcgi?db=gene&cmd=Retrieve&dopt=full_report&list_uids=219287) | APC membrane recruitment protein 2 |
| [Details](https://mirdb.org/cgi-bin/target_detail.cgi?targetID=2855119) | 372 | 90 | hsa-miR-20a-5p | [FAM210A](http://www.ncbi.nlm.nih.gov/entrez/query.fcgi?db=gene&cmd=Retrieve&dopt=full_report&list_uids=125228) | family with sequence similarity 210 member A |
| [Details](https://mirdb.org/cgi-bin/target_detail.cgi?targetID=2855133) | 373 | 90 | hsa-miR-20a-5p | [NAA30](http://www.ncbi.nlm.nih.gov/entrez/query.fcgi?db=gene&cmd=Retrieve&dopt=full_report&list_uids=122830) | N(alpha)-acetyltransferase 30, NatC catalytic subunit |
| [Details](https://mirdb.org/cgi-bin/target_detail.cgi?targetID=2855179) | 374 | 90 | hsa-miR-20a-5p | [KPNA2](http://www.ncbi.nlm.nih.gov/entrez/query.fcgi?db=gene&cmd=Retrieve&dopt=full_report&list_uids=3838) | karyopherin subunit alpha 2 |
| [Details](https://mirdb.org/cgi-bin/target_detail.cgi?targetID=2855258) | 375 | 90 | hsa-miR-20a-5p | [PLXDC2](http://www.ncbi.nlm.nih.gov/entrez/query.fcgi?db=gene&cmd=Retrieve&dopt=full_report&list_uids=84898) | plexin domain containing 2 |
| [Details](https://mirdb.org/cgi-bin/target_detail.cgi?targetID=2855269) | 376 | 90 | hsa-miR-20a-5p | [MEX3D](http://www.ncbi.nlm.nih.gov/entrez/query.fcgi?db=gene&cmd=Retrieve&dopt=full_report&list_uids=399664) | mex-3 RNA binding family member D |
| [Details](https://mirdb.org/cgi-bin/target_detail.cgi?targetID=2855274) | 377 | 90 | hsa-miR-20a-5p | [KLHL15](http://www.ncbi.nlm.nih.gov/entrez/query.fcgi?db=gene&cmd=Retrieve&dopt=full_report&list_uids=80311) | kelch like family member 15 |
| [Details](https://mirdb.org/cgi-bin/target_detail.cgi?targetID=2855282) | 378 | 90 | hsa-miR-20a-5p | [ARHGEF11](http://www.ncbi.nlm.nih.gov/entrez/query.fcgi?db=gene&cmd=Retrieve&dopt=full_report&list_uids=9826) | Rho guanine nucleotide exchange factor 11 |
| [Details](https://mirdb.org/cgi-bin/target_detail.cgi?targetID=2855356) | 379 | 90 | hsa-miR-20a-5p | [SSX2IP](http://www.ncbi.nlm.nih.gov/entrez/query.fcgi?db=gene&cmd=Retrieve&dopt=full_report&list_uids=117178) | SSX family member 2 interacting protein |
| [Details](https://mirdb.org/cgi-bin/target_detail.cgi?targetID=2855380) | 380 | 90 | hsa-miR-20a-5p | [FBXO31](http://www.ncbi.nlm.nih.gov/entrez/query.fcgi?db=gene&cmd=Retrieve&dopt=full_report&list_uids=79791) | F-box protein 31 |
| [Details](https://mirdb.org/cgi-bin/target_detail.cgi?targetID=2855421) | 381 | 90 | hsa-miR-20a-5p | [ST6GALNAC6](http://www.ncbi.nlm.nih.gov/entrez/query.fcgi?db=gene&cmd=Retrieve&dopt=full_report&list_uids=30815) | ST6 N-acetylgalactosaminide alpha-2,6-sialyltransferase 6 |
| [Details](https://mirdb.org/cgi-bin/target_detail.cgi?targetID=2855428) | 382 | 90 | hsa-miR-20a-5p | [DPYSL2](http://www.ncbi.nlm.nih.gov/entrez/query.fcgi?db=gene&cmd=Retrieve&dopt=full_report&list_uids=1808) | dihydropyrimidinase like 2 |
| [Details](https://mirdb.org/cgi-bin/target_detail.cgi?targetID=2855440) | 383 | 90 | hsa-miR-20a-5p | [OXR1](http://www.ncbi.nlm.nih.gov/entrez/query.fcgi?db=gene&cmd=Retrieve&dopt=full_report&list_uids=55074) | oxidation resistance 1 |
| [Details](https://mirdb.org/cgi-bin/target_detail.cgi?targetID=2855447) | 384 | 90 | hsa-miR-20a-5p | [OSTM1](http://www.ncbi.nlm.nih.gov/entrez/query.fcgi?db=gene&cmd=Retrieve&dopt=full_report&list_uids=28962) | osteoclastogenesis associated transmembrane protein 1 |
| [Details](https://mirdb.org/cgi-bin/target_detail.cgi?targetID=2854090) | 385 | 89 | hsa-miR-20a-5p | [MAP3K9](http://www.ncbi.nlm.nih.gov/entrez/query.fcgi?db=gene&cmd=Retrieve&dopt=full_report&list_uids=4293) | mitogen-activated protein kinase kinase kinase 9 |
| [Details](https://mirdb.org/cgi-bin/target_detail.cgi?targetID=2854133) | 386 | 89 | hsa-miR-20a-5p | [FAM13A](http://www.ncbi.nlm.nih.gov/entrez/query.fcgi?db=gene&cmd=Retrieve&dopt=full_report&list_uids=10144) | family with sequence similarity 13 member A |
| [Details](https://mirdb.org/cgi-bin/target_detail.cgi?targetID=2854258) | 387 | 89 | hsa-miR-20a-5p | [SERF1B](http://www.ncbi.nlm.nih.gov/entrez/query.fcgi?db=gene&cmd=Retrieve&dopt=full_report&list_uids=728492) | small EDRK-rich factor 1B |
| [Details](https://mirdb.org/cgi-bin/target_detail.cgi?targetID=2854282) | 388 | 89 | hsa-miR-20a-5p | [APP](http://www.ncbi.nlm.nih.gov/entrez/query.fcgi?db=gene&cmd=Retrieve&dopt=full_report&list_uids=351) | amyloid beta precursor protein |
| [Details](https://mirdb.org/cgi-bin/target_detail.cgi?targetID=2854310) | 389 | 89 | hsa-miR-20a-5p | [ABL2](http://www.ncbi.nlm.nih.gov/entrez/query.fcgi?db=gene&cmd=Retrieve&dopt=full_report&list_uids=27) | ABL proto-oncogene 2, non-receptor tyrosine kinase |
| [Details](https://mirdb.org/cgi-bin/target_detail.cgi?targetID=2854317) | 390 | 89 | hsa-miR-20a-5p | [MFAP3L](http://www.ncbi.nlm.nih.gov/entrez/query.fcgi?db=gene&cmd=Retrieve&dopt=full_report&list_uids=9848) | microfibril associated protein 3 like |
| [Details](https://mirdb.org/cgi-bin/target_detail.cgi?targetID=2854373) | 391 | 89 | hsa-miR-20a-5p | [ROCK2](http://www.ncbi.nlm.nih.gov/entrez/query.fcgi?db=gene&cmd=Retrieve&dopt=full_report&list_uids=9475) | Rho associated coiled-coil containing protein kinase 2 |
| [Details](https://mirdb.org/cgi-bin/target_detail.cgi?targetID=2854378) | 392 | 89 | hsa-miR-20a-5p | [IRF1](http://www.ncbi.nlm.nih.gov/entrez/query.fcgi?db=gene&cmd=Retrieve&dopt=full_report&list_uids=3659) | interferon regulatory factor 1 |
| [Details](https://mirdb.org/cgi-bin/target_detail.cgi?targetID=2854387) | 393 | 89 | hsa-miR-20a-5p | [DCBLD2](http://www.ncbi.nlm.nih.gov/entrez/query.fcgi?db=gene&cmd=Retrieve&dopt=full_report&list_uids=131566) | discoidin, CUB and LCCL domain containing 2 |
| [Details](https://mirdb.org/cgi-bin/target_detail.cgi?targetID=2854399) | 394 | 89 | hsa-miR-20a-5p | [FBXO21](http://www.ncbi.nlm.nih.gov/entrez/query.fcgi?db=gene&cmd=Retrieve&dopt=full_report&list_uids=23014) | F-box protein 21 |
| [Details](https://mirdb.org/cgi-bin/target_detail.cgi?targetID=2854455) | 395 | 89 | hsa-miR-20a-5p | [OLFM3](http://www.ncbi.nlm.nih.gov/entrez/query.fcgi?db=gene&cmd=Retrieve&dopt=full_report&list_uids=118427) | olfactomedin 3 |
| [Details](https://mirdb.org/cgi-bin/target_detail.cgi?targetID=2854526) | 396 | 89 | hsa-miR-20a-5p | [ZBTB18](http://www.ncbi.nlm.nih.gov/entrez/query.fcgi?db=gene&cmd=Retrieve&dopt=full_report&list_uids=10472) | zinc finger and BTB domain containing 18 |
| [Details](https://mirdb.org/cgi-bin/target_detail.cgi?targetID=2854652) | 397 | 89 | hsa-miR-20a-5p | [HTR2A](http://www.ncbi.nlm.nih.gov/entrez/query.fcgi?db=gene&cmd=Retrieve&dopt=full_report&list_uids=3356) | 5-hydroxytryptamine receptor 2A |
| [Details](https://mirdb.org/cgi-bin/target_detail.cgi?targetID=2854668) | 398 | 89 | hsa-miR-20a-5p | [NIPA1](http://www.ncbi.nlm.nih.gov/entrez/query.fcgi?db=gene&cmd=Retrieve&dopt=full_report&list_uids=123606) | NIPA magnesium transporter 1 |
| [Details](https://mirdb.org/cgi-bin/target_detail.cgi?targetID=2854671) | 399 | 89 | hsa-miR-20a-5p | [LHX6](http://www.ncbi.nlm.nih.gov/entrez/query.fcgi?db=gene&cmd=Retrieve&dopt=full_report&list_uids=26468) | LIM homeobox 6 |
| [Details](https://mirdb.org/cgi-bin/target_detail.cgi?targetID=2854680) | 400 | 89 | hsa-miR-20a-5p | [DNAL1](http://www.ncbi.nlm.nih.gov/entrez/query.fcgi?db=gene&cmd=Retrieve&dopt=full_report&list_uids=83544) | dynein axonemal light chain 1 |
| [Details](https://mirdb.org/cgi-bin/target_detail.cgi?targetID=2854703) | 401 | 89 | hsa-miR-20a-5p | [CHD5](http://www.ncbi.nlm.nih.gov/entrez/query.fcgi?db=gene&cmd=Retrieve&dopt=full_report&list_uids=26038) | chromodomain helicase DNA binding protein 5 |
| [Details](https://mirdb.org/cgi-bin/target_detail.cgi?targetID=2854792) | 402 | 89 | hsa-miR-20a-5p | [TANC1](http://www.ncbi.nlm.nih.gov/entrez/query.fcgi?db=gene&cmd=Retrieve&dopt=full_report&list_uids=85461) | tetratricopeptide repeat, ankyrin repeat and coiled-coil containing 1 |
| [Details](https://mirdb.org/cgi-bin/target_detail.cgi?targetID=2854799) | 403 | 89 | hsa-miR-20a-5p | [BTN3A1](http://www.ncbi.nlm.nih.gov/entrez/query.fcgi?db=gene&cmd=Retrieve&dopt=full_report&list_uids=11119) | butyrophilin subfamily 3 member A1 |
| [Details](https://mirdb.org/cgi-bin/target_detail.cgi?targetID=2854811) | 404 | 89 | hsa-miR-20a-5p | [BTBD7](http://www.ncbi.nlm.nih.gov/entrez/query.fcgi?db=gene&cmd=Retrieve&dopt=full_report&list_uids=55727) | BTB domain containing 7 |
| [Details](https://mirdb.org/cgi-bin/target_detail.cgi?targetID=2854820) | 405 | 89 | hsa-miR-20a-5p | [CRYBG3](http://www.ncbi.nlm.nih.gov/entrez/query.fcgi?db=gene&cmd=Retrieve&dopt=full_report&list_uids=131544) | crystallin beta-gamma domain containing 3 |
| [Details](https://mirdb.org/cgi-bin/target_detail.cgi?targetID=2854892) | 406 | 89 | hsa-miR-20a-5p | [WNK3](http://www.ncbi.nlm.nih.gov/entrez/query.fcgi?db=gene&cmd=Retrieve&dopt=full_report&list_uids=65267) | WNK lysine deficient protein kinase 3 |
| [Details](https://mirdb.org/cgi-bin/target_detail.cgi?targetID=2854922) | 407 | 89 | hsa-miR-20a-5p | [IL1RAP](http://www.ncbi.nlm.nih.gov/entrez/query.fcgi?db=gene&cmd=Retrieve&dopt=full_report&list_uids=3556) | interleukin 1 receptor accessory protein |
| [Details](https://mirdb.org/cgi-bin/target_detail.cgi?targetID=2854963) | 408 | 89 | hsa-miR-20a-5p | [AGFG2](http://www.ncbi.nlm.nih.gov/entrez/query.fcgi?db=gene&cmd=Retrieve&dopt=full_report&list_uids=3268) | ArfGAP with FG repeats 2 |
| [Details](https://mirdb.org/cgi-bin/target_detail.cgi?targetID=2855042) | 409 | 89 | hsa-miR-20a-5p | [TNFAIP1](http://www.ncbi.nlm.nih.gov/entrez/query.fcgi?db=gene&cmd=Retrieve&dopt=full_report&list_uids=7126) | TNF alpha induced protein 1 |
| [Details](https://mirdb.org/cgi-bin/target_detail.cgi?targetID=2855056) | 410 | 89 | hsa-miR-20a-5p | [SMOC2](http://www.ncbi.nlm.nih.gov/entrez/query.fcgi?db=gene&cmd=Retrieve&dopt=full_report&list_uids=64094) | SPARC related modular calcium binding 2 |
| [Details](https://mirdb.org/cgi-bin/target_detail.cgi?targetID=2855100) | 411 | 89 | hsa-miR-20a-5p | [SERF1A](http://www.ncbi.nlm.nih.gov/entrez/query.fcgi?db=gene&cmd=Retrieve&dopt=full_report&list_uids=8293) | small EDRK-rich factor 1A |
| [Details](https://mirdb.org/cgi-bin/target_detail.cgi?targetID=2855122) | 412 | 89 | hsa-miR-20a-5p | [RLIM](http://www.ncbi.nlm.nih.gov/entrez/query.fcgi?db=gene&cmd=Retrieve&dopt=full_report&list_uids=51132) | ring finger protein, LIM domain interacting |
| [Details](https://mirdb.org/cgi-bin/target_detail.cgi?targetID=2855149) | 413 | 89 | hsa-miR-20a-5p | [SRGAP1](http://www.ncbi.nlm.nih.gov/entrez/query.fcgi?db=gene&cmd=Retrieve&dopt=full_report&list_uids=57522) | SLIT-ROBO Rho GTPase activating protein 1 |
| [Details](https://mirdb.org/cgi-bin/target_detail.cgi?targetID=2855165) | 414 | 89 | hsa-miR-20a-5p | [OSR1](http://www.ncbi.nlm.nih.gov/entrez/query.fcgi?db=gene&cmd=Retrieve&dopt=full_report&list_uids=130497) | odd-skipped related transcription factor 1 |
| [Details](https://mirdb.org/cgi-bin/target_detail.cgi?targetID=2855195) | 415 | 89 | hsa-miR-20a-5p | [TNKS1BP1](http://www.ncbi.nlm.nih.gov/entrez/query.fcgi?db=gene&cmd=Retrieve&dopt=full_report&list_uids=85456) | tankyrase 1 binding protein 1 |
| [Details](https://mirdb.org/cgi-bin/target_detail.cgi?targetID=2855202) | 416 | 89 | hsa-miR-20a-5p | [SLC22A23](http://www.ncbi.nlm.nih.gov/entrez/query.fcgi?db=gene&cmd=Retrieve&dopt=full_report&list_uids=63027) | solute carrier family 22 member 23 |
| [Details](https://mirdb.org/cgi-bin/target_detail.cgi?targetID=2855228) | 417 | 89 | hsa-miR-20a-5p | [GOSR1](http://www.ncbi.nlm.nih.gov/entrez/query.fcgi?db=gene&cmd=Retrieve&dopt=full_report&list_uids=9527) | golgi SNAP receptor complex member 1 |
| [Details](https://mirdb.org/cgi-bin/target_detail.cgi?targetID=2855230) | 418 | 89 | hsa-miR-20a-5p | [USP32](http://www.ncbi.nlm.nih.gov/entrez/query.fcgi?db=gene&cmd=Retrieve&dopt=full_report&list_uids=84669) | ubiquitin specific peptidase 32 |
| [Details](https://mirdb.org/cgi-bin/target_detail.cgi?targetID=2855248) | 419 | 89 | hsa-miR-20a-5p | [PBX3](http://www.ncbi.nlm.nih.gov/entrez/query.fcgi?db=gene&cmd=Retrieve&dopt=full_report&list_uids=5090) | PBX homeobox 3 |
| [Details](https://mirdb.org/cgi-bin/target_detail.cgi?targetID=2855255) | 420 | 89 | hsa-miR-20a-5p | [TPRG1L](http://www.ncbi.nlm.nih.gov/entrez/query.fcgi?db=gene&cmd=Retrieve&dopt=full_report&list_uids=127262) | tumor protein p63 regulated 1 like |
| [Details](https://mirdb.org/cgi-bin/target_detail.cgi?targetID=2855299) | 421 | 89 | hsa-miR-20a-5p | [MCL1](http://www.ncbi.nlm.nih.gov/entrez/query.fcgi?db=gene&cmd=Retrieve&dopt=full_report&list_uids=4170) | MCL1, BCL2 family apoptosis regulator |
| [Details](https://mirdb.org/cgi-bin/target_detail.cgi?targetID=2855442) | 422 | 89 | hsa-miR-20a-5p | [PITPNA](http://www.ncbi.nlm.nih.gov/entrez/query.fcgi?db=gene&cmd=Retrieve&dopt=full_report&list_uids=5306) | phosphatidylinositol transfer protein alpha |
| [Details](https://mirdb.org/cgi-bin/target_detail.cgi?targetID=2855443) | 423 | 89 | hsa-miR-20a-5p | [PLAGL2](http://www.ncbi.nlm.nih.gov/entrez/query.fcgi?db=gene&cmd=Retrieve&dopt=full_report&list_uids=5326) | PLAG1 like zinc finger 2 |
| [Details](https://mirdb.org/cgi-bin/target_detail.cgi?targetID=2855452) | 424 | 89 | hsa-miR-20a-5p | [RSRP1](http://www.ncbi.nlm.nih.gov/entrez/query.fcgi?db=gene&cmd=Retrieve&dopt=full_report&list_uids=57035) | arginine and serine rich protein 1 |
| [Details](https://mirdb.org/cgi-bin/target_detail.cgi?targetID=2854091) | 425 | 88 | hsa-miR-20a-5p | [PANX2](http://www.ncbi.nlm.nih.gov/entrez/query.fcgi?db=gene&cmd=Retrieve&dopt=full_report&list_uids=56666) | pannexin 2 |
| [Details](https://mirdb.org/cgi-bin/target_detail.cgi?targetID=2854105) | 426 | 88 | hsa-miR-20a-5p | [SNX8](http://www.ncbi.nlm.nih.gov/entrez/query.fcgi?db=gene&cmd=Retrieve&dopt=full_report&list_uids=29886) | sorting nexin 8 |
| [Details](https://mirdb.org/cgi-bin/target_detail.cgi?targetID=2854135) | 427 | 88 | hsa-miR-20a-5p | [FAM19A1](http://www.ncbi.nlm.nih.gov/entrez/query.fcgi?db=gene&cmd=Retrieve&dopt=full_report&list_uids=407738) | family with sequence similarity 19 member A1, C-C motif chemokine like |
| [Details](https://mirdb.org/cgi-bin/target_detail.cgi?targetID=2854140) | 428 | 88 | hsa-miR-20a-5p | [PGBD5](http://www.ncbi.nlm.nih.gov/entrez/query.fcgi?db=gene&cmd=Retrieve&dopt=full_report&list_uids=79605) | piggyBac transposable element derived 5 |
| [Details](https://mirdb.org/cgi-bin/target_detail.cgi?targetID=2854180) | 429 | 88 | hsa-miR-20a-5p | [FOXK2](http://www.ncbi.nlm.nih.gov/entrez/query.fcgi?db=gene&cmd=Retrieve&dopt=full_report&list_uids=3607) | forkhead box K2 |
| [Details](https://mirdb.org/cgi-bin/target_detail.cgi?targetID=2854238) | 430 | 88 | hsa-miR-20a-5p | [CTSA](http://www.ncbi.nlm.nih.gov/entrez/query.fcgi?db=gene&cmd=Retrieve&dopt=full_report&list_uids=5476) | cathepsin A |
| [Details](https://mirdb.org/cgi-bin/target_detail.cgi?targetID=2854269) | 431 | 88 | hsa-miR-20a-5p | [TFAM](http://www.ncbi.nlm.nih.gov/entrez/query.fcgi?db=gene&cmd=Retrieve&dopt=full_report&list_uids=7019) | transcription factor A, mitochondrial |
| [Details](https://mirdb.org/cgi-bin/target_detail.cgi?targetID=2854302) | 432 | 88 | hsa-miR-20a-5p | [CYBRD1](http://www.ncbi.nlm.nih.gov/entrez/query.fcgi?db=gene&cmd=Retrieve&dopt=full_report&list_uids=79901) | cytochrome b reductase 1 |
| [Details](https://mirdb.org/cgi-bin/target_detail.cgi?targetID=2854303) | 433 | 88 | hsa-miR-20a-5p | [CORO2B](http://www.ncbi.nlm.nih.gov/entrez/query.fcgi?db=gene&cmd=Retrieve&dopt=full_report&list_uids=10391) | coronin 2B |
| [Details](https://mirdb.org/cgi-bin/target_detail.cgi?targetID=2854305) | 434 | 88 | hsa-miR-20a-5p | [UNKL](http://www.ncbi.nlm.nih.gov/entrez/query.fcgi?db=gene&cmd=Retrieve&dopt=full_report&list_uids=64718) | unk like zinc finger |
| [Details](https://mirdb.org/cgi-bin/target_detail.cgi?targetID=2854383) | 435 | 88 | hsa-miR-20a-5p | [CAPN15](http://www.ncbi.nlm.nih.gov/entrez/query.fcgi?db=gene&cmd=Retrieve&dopt=full_report&list_uids=6650) | calpain 15 |
| [Details](https://mirdb.org/cgi-bin/target_detail.cgi?targetID=2854437) | 436 | 88 | hsa-miR-20a-5p | [MFN2](http://www.ncbi.nlm.nih.gov/entrez/query.fcgi?db=gene&cmd=Retrieve&dopt=full_report&list_uids=9927) | mitofusin 2 |
| [Details](https://mirdb.org/cgi-bin/target_detail.cgi?targetID=2854483) | 437 | 88 | hsa-miR-20a-5p | [TMEM265](http://www.ncbi.nlm.nih.gov/entrez/query.fcgi?db=gene&cmd=Retrieve&dopt=full_report&list_uids=100862671) | transmembrane protein 265 |
| [Details](https://mirdb.org/cgi-bin/target_detail.cgi?targetID=2854585) | 438 | 88 | hsa-miR-20a-5p | [GUCY1A1](http://www.ncbi.nlm.nih.gov/entrez/query.fcgi?db=gene&cmd=Retrieve&dopt=full_report&list_uids=2982) | guanylate cyclase 1 soluble subunit alpha 1 |
| [Details](https://mirdb.org/cgi-bin/target_detail.cgi?targetID=2854586) | 439 | 88 | hsa-miR-20a-5p | [FNBP1L](http://www.ncbi.nlm.nih.gov/entrez/query.fcgi?db=gene&cmd=Retrieve&dopt=full_report&list_uids=54874) | formin binding protein 1 like |
| [Details](https://mirdb.org/cgi-bin/target_detail.cgi?targetID=2854640) | 440 | 88 | hsa-miR-20a-5p | [ZNF264](http://www.ncbi.nlm.nih.gov/entrez/query.fcgi?db=gene&cmd=Retrieve&dopt=full_report&list_uids=9422) | zinc finger protein 264 |
| [Details](https://mirdb.org/cgi-bin/target_detail.cgi?targetID=2854654) | 441 | 88 | hsa-miR-20a-5p | [MOSMO](http://www.ncbi.nlm.nih.gov/entrez/query.fcgi?db=gene&cmd=Retrieve&dopt=full_report&list_uids=730094) | modulator of smoothened |
| [Details](https://mirdb.org/cgi-bin/target_detail.cgi?targetID=2854685) | 442 | 88 | hsa-miR-20a-5p | [FAT4](http://www.ncbi.nlm.nih.gov/entrez/query.fcgi?db=gene&cmd=Retrieve&dopt=full_report&list_uids=79633) | FAT atypical cadherin 4 |
| [Details](https://mirdb.org/cgi-bin/target_detail.cgi?targetID=2854691) | 443 | 88 | hsa-miR-20a-5p | [SMAD5](http://www.ncbi.nlm.nih.gov/entrez/query.fcgi?db=gene&cmd=Retrieve&dopt=full_report&list_uids=4090) | SMAD family member 5 |
| [Details](https://mirdb.org/cgi-bin/target_detail.cgi?targetID=2854808) | 444 | 88 | hsa-miR-20a-5p | [PIK3R1](http://www.ncbi.nlm.nih.gov/entrez/query.fcgi?db=gene&cmd=Retrieve&dopt=full_report&list_uids=5295) | phosphoinositide-3-kinase regulatory subunit 1 |
| [Details](https://mirdb.org/cgi-bin/target_detail.cgi?targetID=2854875) | 445 | 88 | hsa-miR-20a-5p | [SFMBT1](http://www.ncbi.nlm.nih.gov/entrez/query.fcgi?db=gene&cmd=Retrieve&dopt=full_report&list_uids=51460) | Scm like with four mbt domains 1 |
| [Details](https://mirdb.org/cgi-bin/target_detail.cgi?targetID=2854886) | 446 | 88 | hsa-miR-20a-5p | [DOCK4](http://www.ncbi.nlm.nih.gov/entrez/query.fcgi?db=gene&cmd=Retrieve&dopt=full_report&list_uids=9732) | dedicator of cytokinesis 4 |
| [Details](https://mirdb.org/cgi-bin/target_detail.cgi?targetID=2854887) | 447 | 88 | hsa-miR-20a-5p | [CMKLR1](http://www.ncbi.nlm.nih.gov/entrez/query.fcgi?db=gene&cmd=Retrieve&dopt=full_report&list_uids=1240) | chemerin chemokine-like receptor 1 |
| [Details](https://mirdb.org/cgi-bin/target_detail.cgi?targetID=2855118) | 448 | 88 | hsa-miR-20a-5p | [ABCG4](http://www.ncbi.nlm.nih.gov/entrez/query.fcgi?db=gene&cmd=Retrieve&dopt=full_report&list_uids=64137) | ATP binding cassette subfamily G member 4 |
| [Details](https://mirdb.org/cgi-bin/target_detail.cgi?targetID=2855146) | 449 | 88 | hsa-miR-20a-5p | [RHOC](http://www.ncbi.nlm.nih.gov/entrez/query.fcgi?db=gene&cmd=Retrieve&dopt=full_report&list_uids=389) | ras homolog family member C |
| [Details](https://mirdb.org/cgi-bin/target_detail.cgi?targetID=2855222) | 450 | 88 | hsa-miR-20a-5p | [NUP35](http://www.ncbi.nlm.nih.gov/entrez/query.fcgi?db=gene&cmd=Retrieve&dopt=full_report&list_uids=129401) | nucleoporin 35 |
| [Details](https://mirdb.org/cgi-bin/target_detail.cgi?targetID=2855232) | 451 | 88 | hsa-miR-20a-5p | [REPS2](http://www.ncbi.nlm.nih.gov/entrez/query.fcgi?db=gene&cmd=Retrieve&dopt=full_report&list_uids=9185) | RALBP1 associated Eps domain containing 2 |
| [Details](https://mirdb.org/cgi-bin/target_detail.cgi?targetID=2855297) | 452 | 88 | hsa-miR-20a-5p | [ZNF148](http://www.ncbi.nlm.nih.gov/entrez/query.fcgi?db=gene&cmd=Retrieve&dopt=full_report&list_uids=7707) | zinc finger protein 148 |
| [Details](https://mirdb.org/cgi-bin/target_detail.cgi?targetID=2855307) | 453 | 88 | hsa-miR-20a-5p | [FBXL3](http://www.ncbi.nlm.nih.gov/entrez/query.fcgi?db=gene&cmd=Retrieve&dopt=full_report&list_uids=26224) | F-box and leucine rich repeat protein 3 |
| [Details](https://mirdb.org/cgi-bin/target_detail.cgi?targetID=2854099) | 454 | 87 | hsa-miR-20a-5p | [PDLIM5](http://www.ncbi.nlm.nih.gov/entrez/query.fcgi?db=gene&cmd=Retrieve&dopt=full_report&list_uids=10611) | PDZ and LIM domain 5 |
| [Details](https://mirdb.org/cgi-bin/target_detail.cgi?targetID=2854220) | 455 | 87 | hsa-miR-20a-5p | [SRPK2](http://www.ncbi.nlm.nih.gov/entrez/query.fcgi?db=gene&cmd=Retrieve&dopt=full_report&list_uids=6733) | SRSF protein kinase 2 |
| [Details](https://mirdb.org/cgi-bin/target_detail.cgi?targetID=2854230) | 456 | 87 | hsa-miR-20a-5p | [AKAP11](http://www.ncbi.nlm.nih.gov/entrez/query.fcgi?db=gene&cmd=Retrieve&dopt=full_report&list_uids=11215) | A-kinase anchoring protein 11 |
| [Details](https://mirdb.org/cgi-bin/target_detail.cgi?targetID=2854255) | 457 | 87 | hsa-miR-20a-5p | [STAT3](http://www.ncbi.nlm.nih.gov/entrez/query.fcgi?db=gene&cmd=Retrieve&dopt=full_report&list_uids=6774) | signal transducer and activator of transcription 3 |
| [Details](https://mirdb.org/cgi-bin/target_detail.cgi?targetID=2854348) | 458 | 87 | hsa-miR-20a-5p | [ZNF202](http://www.ncbi.nlm.nih.gov/entrez/query.fcgi?db=gene&cmd=Retrieve&dopt=full_report&list_uids=7753) | zinc finger protein 202 |
| [Details](https://mirdb.org/cgi-bin/target_detail.cgi?targetID=2854384) | 459 | 87 | hsa-miR-20a-5p | [NR2C1](http://www.ncbi.nlm.nih.gov/entrez/query.fcgi?db=gene&cmd=Retrieve&dopt=full_report&list_uids=7181) | nuclear receptor subfamily 2 group C member 1 |
| [Details](https://mirdb.org/cgi-bin/target_detail.cgi?targetID=2854450) | 460 | 87 | hsa-miR-20a-5p | [ZDHHC1](http://www.ncbi.nlm.nih.gov/entrez/query.fcgi?db=gene&cmd=Retrieve&dopt=full_report&list_uids=29800) | zinc finger DHHC-type containing 1 |
| [Details](https://mirdb.org/cgi-bin/target_detail.cgi?targetID=2854463) | 461 | 87 | hsa-miR-20a-5p | [PHIP](http://www.ncbi.nlm.nih.gov/entrez/query.fcgi?db=gene&cmd=Retrieve&dopt=full_report&list_uids=55023) | pleckstrin homology domain interacting protein |
| [Details](https://mirdb.org/cgi-bin/target_detail.cgi?targetID=2854544) | 462 | 87 | hsa-miR-20a-5p | [HIF1A](http://www.ncbi.nlm.nih.gov/entrez/query.fcgi?db=gene&cmd=Retrieve&dopt=full_report&list_uids=3091) | hypoxia inducible factor 1 subunit alpha |
| [Details](https://mirdb.org/cgi-bin/target_detail.cgi?targetID=2854633) | 463 | 87 | hsa-miR-20a-5p | [ANKRD13C](http://www.ncbi.nlm.nih.gov/entrez/query.fcgi?db=gene&cmd=Retrieve&dopt=full_report&list_uids=81573) | ankyrin repeat domain 13C |
| [Details](https://mirdb.org/cgi-bin/target_detail.cgi?targetID=2854701) | 464 | 87 | hsa-miR-20a-5p | [WDFY2](http://www.ncbi.nlm.nih.gov/entrez/query.fcgi?db=gene&cmd=Retrieve&dopt=full_report&list_uids=115825) | WD repeat and FYVE domain containing 2 |
| [Details](https://mirdb.org/cgi-bin/target_detail.cgi?targetID=2854716) | 465 | 87 | hsa-miR-20a-5p | [CD274](http://www.ncbi.nlm.nih.gov/entrez/query.fcgi?db=gene&cmd=Retrieve&dopt=full_report&list_uids=29126) | CD274 molecule |
| [Details](https://mirdb.org/cgi-bin/target_detail.cgi?targetID=2854717) | 466 | 87 | hsa-miR-20a-5p | [PDGFRA](http://www.ncbi.nlm.nih.gov/entrez/query.fcgi?db=gene&cmd=Retrieve&dopt=full_report&list_uids=5156) | platelet derived growth factor receptor alpha |
| [Details](https://mirdb.org/cgi-bin/target_detail.cgi?targetID=2854850) | 467 | 87 | hsa-miR-20a-5p | [RETREG3](http://www.ncbi.nlm.nih.gov/entrez/query.fcgi?db=gene&cmd=Retrieve&dopt=full_report&list_uids=162427) | reticulophagy regulator family member 3 |
| [Details](https://mirdb.org/cgi-bin/target_detail.cgi?targetID=2855023) | 468 | 87 | hsa-miR-20a-5p | [MIDN](http://www.ncbi.nlm.nih.gov/entrez/query.fcgi?db=gene&cmd=Retrieve&dopt=full_report&list_uids=90007) | midnolin |
| [Details](https://mirdb.org/cgi-bin/target_detail.cgi?targetID=2855160) | 469 | 87 | hsa-miR-20a-5p | [MMP24](http://www.ncbi.nlm.nih.gov/entrez/query.fcgi?db=gene&cmd=Retrieve&dopt=full_report&list_uids=10893) | matrix metallopeptidase 24 |
| [Details](https://mirdb.org/cgi-bin/target_detail.cgi?targetID=2855191) | 470 | 87 | hsa-miR-20a-5p | [SH3PXD2A](http://www.ncbi.nlm.nih.gov/entrez/query.fcgi?db=gene&cmd=Retrieve&dopt=full_report&list_uids=9644) | SH3 and PX domains 2A |
| [Details](https://mirdb.org/cgi-bin/target_detail.cgi?targetID=2855194) | 471 | 87 | hsa-miR-20a-5p | [BAHD1](http://www.ncbi.nlm.nih.gov/entrez/query.fcgi?db=gene&cmd=Retrieve&dopt=full_report&list_uids=22893) | bromo adjacent homology domain containing 1 |
| [Details](https://mirdb.org/cgi-bin/target_detail.cgi?targetID=2855203) | 472 | 87 | hsa-miR-20a-5p | [ST3GAL1](http://www.ncbi.nlm.nih.gov/entrez/query.fcgi?db=gene&cmd=Retrieve&dopt=full_report&list_uids=6482) | ST3 beta-galactoside alpha-2,3-sialyltransferase 1 |
| [Details](https://mirdb.org/cgi-bin/target_detail.cgi?targetID=2855218) | 473 | 87 | hsa-miR-20a-5p | [ZBTB9](http://www.ncbi.nlm.nih.gov/entrez/query.fcgi?db=gene&cmd=Retrieve&dopt=full_report&list_uids=221504) | zinc finger and BTB domain containing 9 |
| [Details](https://mirdb.org/cgi-bin/target_detail.cgi?targetID=2855290) | 474 | 87 | hsa-miR-20a-5p | [SLMAP](http://www.ncbi.nlm.nih.gov/entrez/query.fcgi?db=gene&cmd=Retrieve&dopt=full_report&list_uids=7871) | sarcolemma associated protein |
| [Details](https://mirdb.org/cgi-bin/target_detail.cgi?targetID=2855351) | 475 | 87 | hsa-miR-20a-5p | [FAM199X](http://www.ncbi.nlm.nih.gov/entrez/query.fcgi?db=gene&cmd=Retrieve&dopt=full_report&list_uids=139231) | family with sequence similarity 199, X-linked |
| [Details](https://mirdb.org/cgi-bin/target_detail.cgi?targetID=2855384) | 476 | 87 | hsa-miR-20a-5p | [OTUD4](http://www.ncbi.nlm.nih.gov/entrez/query.fcgi?db=gene&cmd=Retrieve&dopt=full_report&list_uids=54726) | OTU deubiquitinase 4 |
| [Details](https://mirdb.org/cgi-bin/target_detail.cgi?targetID=2855398) | 477 | 87 | hsa-miR-20a-5p | [ANKRD50](http://www.ncbi.nlm.nih.gov/entrez/query.fcgi?db=gene&cmd=Retrieve&dopt=full_report&list_uids=57182) | ankyrin repeat domain 50 |
| [Details](https://mirdb.org/cgi-bin/target_detail.cgi?targetID=2855411) | 478 | 87 | hsa-miR-20a-5p | [CNRIP1](http://www.ncbi.nlm.nih.gov/entrez/query.fcgi?db=gene&cmd=Retrieve&dopt=full_report&list_uids=25927) | cannabinoid receptor interacting protein 1 |
| [Details](https://mirdb.org/cgi-bin/target_detail.cgi?targetID=2855417) | 479 | 87 | hsa-miR-20a-5p | [ZNF236](http://www.ncbi.nlm.nih.gov/entrez/query.fcgi?db=gene&cmd=Retrieve&dopt=full_report&list_uids=7776) | zinc finger protein 236 |
| [Details](https://mirdb.org/cgi-bin/target_detail.cgi?targetID=2855453) | 480 | 87 | hsa-miR-20a-5p | [PPP1R21](http://www.ncbi.nlm.nih.gov/entrez/query.fcgi?db=gene&cmd=Retrieve&dopt=full_report&list_uids=129285) | protein phosphatase 1 regulatory subunit 21 |
| [Details](https://mirdb.org/cgi-bin/target_detail.cgi?targetID=2855455) | 481 | 87 | hsa-miR-20a-5p | [ZFPM2](http://www.ncbi.nlm.nih.gov/entrez/query.fcgi?db=gene&cmd=Retrieve&dopt=full_report&list_uids=23414) | zinc finger protein, FOG family member 2 |
| [Details](https://mirdb.org/cgi-bin/target_detail.cgi?targetID=2854102) | 482 | 86 | hsa-miR-20a-5p | [PKD1](http://www.ncbi.nlm.nih.gov/entrez/query.fcgi?db=gene&cmd=Retrieve&dopt=full_report&list_uids=5310) | polycystin 1, transient receptor potential channel interacting |
| [Details](https://mirdb.org/cgi-bin/target_detail.cgi?targetID=2854107) | 483 | 86 | hsa-miR-20a-5p | [MAGI3](http://www.ncbi.nlm.nih.gov/entrez/query.fcgi?db=gene&cmd=Retrieve&dopt=full_report&list_uids=260425) | membrane associated guanylate kinase, WW and PDZ domain containing 3 |
| [Details](https://mirdb.org/cgi-bin/target_detail.cgi?targetID=2854111) | 484 | 86 | hsa-miR-20a-5p | [WFS1](http://www.ncbi.nlm.nih.gov/entrez/query.fcgi?db=gene&cmd=Retrieve&dopt=full_report&list_uids=7466) | wolframin ER transmembrane glycoprotein |
| [Details](https://mirdb.org/cgi-bin/target_detail.cgi?targetID=2854112) | 485 | 86 | hsa-miR-20a-5p | [CEP170](http://www.ncbi.nlm.nih.gov/entrez/query.fcgi?db=gene&cmd=Retrieve&dopt=full_report&list_uids=9859) | centrosomal protein 170 |
| [Details](https://mirdb.org/cgi-bin/target_detail.cgi?targetID=2854118) | 486 | 86 | hsa-miR-20a-5p | [ATG2B](http://www.ncbi.nlm.nih.gov/entrez/query.fcgi?db=gene&cmd=Retrieve&dopt=full_report&list_uids=55102) | autophagy related 2B |
| [Details](https://mirdb.org/cgi-bin/target_detail.cgi?targetID=2854122) | 487 | 86 | hsa-miR-20a-5p | [RAPGEFL1](http://www.ncbi.nlm.nih.gov/entrez/query.fcgi?db=gene&cmd=Retrieve&dopt=full_report&list_uids=51195) | Rap guanine nucleotide exchange factor like 1 |
| [Details](https://mirdb.org/cgi-bin/target_detail.cgi?targetID=2854169) | 488 | 86 | hsa-miR-20a-5p | [TMX3](http://www.ncbi.nlm.nih.gov/entrez/query.fcgi?db=gene&cmd=Retrieve&dopt=full_report&list_uids=54495) | thioredoxin related transmembrane protein 3 |
| [Details](https://mirdb.org/cgi-bin/target_detail.cgi?targetID=2854202) | 489 | 86 | hsa-miR-20a-5p | [SERP1](http://www.ncbi.nlm.nih.gov/entrez/query.fcgi?db=gene&cmd=Retrieve&dopt=full_report&list_uids=27230) | stress associated endoplasmic reticulum protein 1 |
| [Details](https://mirdb.org/cgi-bin/target_detail.cgi?targetID=2854204) | 490 | 86 | hsa-miR-20a-5p | [ZNF2](http://www.ncbi.nlm.nih.gov/entrez/query.fcgi?db=gene&cmd=Retrieve&dopt=full_report&list_uids=7549) | zinc finger protein 2 |
| [Details](https://mirdb.org/cgi-bin/target_detail.cgi?targetID=2854237) | 491 | 86 | hsa-miR-20a-5p | [DCUN1D1](http://www.ncbi.nlm.nih.gov/entrez/query.fcgi?db=gene&cmd=Retrieve&dopt=full_report&list_uids=54165) | defective in cullin neddylation 1 domain containing 1 |
| [Details](https://mirdb.org/cgi-bin/target_detail.cgi?targetID=2854328) | 492 | 86 | hsa-miR-20a-5p | [REV3L](http://www.ncbi.nlm.nih.gov/entrez/query.fcgi?db=gene&cmd=Retrieve&dopt=full_report&list_uids=5980) | REV3 like, DNA directed polymerase zeta catalytic subunit |
| [Details](https://mirdb.org/cgi-bin/target_detail.cgi?targetID=2854356) | 493 | 86 | hsa-miR-20a-5p | [PLXNA1](http://www.ncbi.nlm.nih.gov/entrez/query.fcgi?db=gene&cmd=Retrieve&dopt=full_report&list_uids=5361) | plexin A1 |
| [Details](https://mirdb.org/cgi-bin/target_detail.cgi?targetID=2854408) | 494 | 86 | hsa-miR-20a-5p | [SOX4](http://www.ncbi.nlm.nih.gov/entrez/query.fcgi?db=gene&cmd=Retrieve&dopt=full_report&list_uids=6659) | SRY-box 4 |
| [Details](https://mirdb.org/cgi-bin/target_detail.cgi?targetID=2854409) | 495 | 86 | hsa-miR-20a-5p | [ABHD5](http://www.ncbi.nlm.nih.gov/entrez/query.fcgi?db=gene&cmd=Retrieve&dopt=full_report&list_uids=51099) | abhydrolase domain containing 5 |
| [Details](https://mirdb.org/cgi-bin/target_detail.cgi?targetID=2854426) | 496 | 86 | hsa-miR-20a-5p | [ABI1](http://www.ncbi.nlm.nih.gov/entrez/query.fcgi?db=gene&cmd=Retrieve&dopt=full_report&list_uids=10006) | abl interactor 1 |
| [Details](https://mirdb.org/cgi-bin/target_detail.cgi?targetID=2854429) | 497 | 86 | hsa-miR-20a-5p | [RAB10](http://www.ncbi.nlm.nih.gov/entrez/query.fcgi?db=gene&cmd=Retrieve&dopt=full_report&list_uids=10890) | RAB10, member RAS oncogene family |
| [Details](https://mirdb.org/cgi-bin/target_detail.cgi?targetID=2854452) | 498 | 86 | hsa-miR-20a-5p | [MYLIP](http://www.ncbi.nlm.nih.gov/entrez/query.fcgi?db=gene&cmd=Retrieve&dopt=full_report&list_uids=29116) | myosin regulatory light chain interacting protein |
| [Details](https://mirdb.org/cgi-bin/target_detail.cgi?targetID=2854468) | 499 | 86 | hsa-miR-20a-5p | [CDC37L1](http://www.ncbi.nlm.nih.gov/entrez/query.fcgi?db=gene&cmd=Retrieve&dopt=full_report&list_uids=55664) | cell division cycle 37 like 1 |
| [Details](https://mirdb.org/cgi-bin/target_detail.cgi?targetID=2854547) | 500 | 86 | hsa-miR-20a-5p | [ZSCAN20](http://www.ncbi.nlm.nih.gov/entrez/query.fcgi?db=gene&cmd=Retrieve&dopt=full_report&list_uids=7579) | zinc finger and SCAN domain containing 20 |
| [Details](https://mirdb.org/cgi-bin/target_detail.cgi?targetID=2854641) | 501 | 86 | hsa-miR-20a-5p | [CREB5](http://www.ncbi.nlm.nih.gov/entrez/query.fcgi?db=gene&cmd=Retrieve&dopt=full_report&list_uids=9586) | cAMP responsive element binding protein 5 |
| [Details](https://mirdb.org/cgi-bin/target_detail.cgi?targetID=2854650) | 502 | 86 | hsa-miR-20a-5p | [TSG101](http://www.ncbi.nlm.nih.gov/entrez/query.fcgi?db=gene&cmd=Retrieve&dopt=full_report&list_uids=7251) | tumor susceptibility 101 |
| [Details](https://mirdb.org/cgi-bin/target_detail.cgi?targetID=2854681) | 503 | 86 | hsa-miR-20a-5p | [DLGAP1](http://www.ncbi.nlm.nih.gov/entrez/query.fcgi?db=gene&cmd=Retrieve&dopt=full_report&list_uids=9229) | DLG associated protein 1 |
| [Details](https://mirdb.org/cgi-bin/target_detail.cgi?targetID=2854760) | 504 | 86 | hsa-miR-20a-5p | [RRAS2](http://www.ncbi.nlm.nih.gov/entrez/query.fcgi?db=gene&cmd=Retrieve&dopt=full_report&list_uids=22800) | RAS related 2 |
| [Details](https://mirdb.org/cgi-bin/target_detail.cgi?targetID=2854814) | 505 | 86 | hsa-miR-20a-5p | [TMEM167A](http://www.ncbi.nlm.nih.gov/entrez/query.fcgi?db=gene&cmd=Retrieve&dopt=full_report&list_uids=153339) | transmembrane protein 167A |
| [Details](https://mirdb.org/cgi-bin/target_detail.cgi?targetID=2854844) | 506 | 86 | hsa-miR-20a-5p | [MINK1](http://www.ncbi.nlm.nih.gov/entrez/query.fcgi?db=gene&cmd=Retrieve&dopt=full_report&list_uids=50488) | misshapen like kinase 1 |
| [Details](https://mirdb.org/cgi-bin/target_detail.cgi?targetID=2854848) | 507 | 86 | hsa-miR-20a-5p | [LYPD6](http://www.ncbi.nlm.nih.gov/entrez/query.fcgi?db=gene&cmd=Retrieve&dopt=full_report&list_uids=130574) | LY6/PLAUR domain containing 6 |
| [Details](https://mirdb.org/cgi-bin/target_detail.cgi?targetID=2854863) | 508 | 86 | hsa-miR-20a-5p | [SNX16](http://www.ncbi.nlm.nih.gov/entrez/query.fcgi?db=gene&cmd=Retrieve&dopt=full_report&list_uids=64089) | sorting nexin 16 |
| [Details](https://mirdb.org/cgi-bin/target_detail.cgi?targetID=2854871) | 509 | 86 | hsa-miR-20a-5p | [BHLHE41](http://www.ncbi.nlm.nih.gov/entrez/query.fcgi?db=gene&cmd=Retrieve&dopt=full_report&list_uids=79365) | basic helix-loop-helix family member e41 |
| [Details](https://mirdb.org/cgi-bin/target_detail.cgi?targetID=2854883) | 510 | 86 | hsa-miR-20a-5p | [CMPK1](http://www.ncbi.nlm.nih.gov/entrez/query.fcgi?db=gene&cmd=Retrieve&dopt=full_report&list_uids=51727) | cytidine/uridine monophosphate kinase 1 |
| [Details](https://mirdb.org/cgi-bin/target_detail.cgi?targetID=2854884) | 511 | 86 | hsa-miR-20a-5p | [RBM12B](http://www.ncbi.nlm.nih.gov/entrez/query.fcgi?db=gene&cmd=Retrieve&dopt=full_report&list_uids=389677) | RNA binding motif protein 12B |
| [Details](https://mirdb.org/cgi-bin/target_detail.cgi?targetID=2854935) | 512 | 86 | hsa-miR-20a-5p | [NTNG1](http://www.ncbi.nlm.nih.gov/entrez/query.fcgi?db=gene&cmd=Retrieve&dopt=full_report&list_uids=22854) | netrin G1 |
| [Details](https://mirdb.org/cgi-bin/target_detail.cgi?targetID=2854966) | 513 | 86 | hsa-miR-20a-5p | [NANOS1](http://www.ncbi.nlm.nih.gov/entrez/query.fcgi?db=gene&cmd=Retrieve&dopt=full_report&list_uids=340719) | nanos C2HC-type zinc finger 1 |
| [Details](https://mirdb.org/cgi-bin/target_detail.cgi?targetID=2854993) | 514 | 86 | hsa-miR-20a-5p | [RBBP7](http://www.ncbi.nlm.nih.gov/entrez/query.fcgi?db=gene&cmd=Retrieve&dopt=full_report&list_uids=5931) | RB binding protein 7, chromatin remodeling factor |
| [Details](https://mirdb.org/cgi-bin/target_detail.cgi?targetID=2855030) | 515 | 86 | hsa-miR-20a-5p | [ZBTB21](http://www.ncbi.nlm.nih.gov/entrez/query.fcgi?db=gene&cmd=Retrieve&dopt=full_report&list_uids=49854) | zinc finger and BTB domain containing 21 |
| [Details](https://mirdb.org/cgi-bin/target_detail.cgi?targetID=2855035) | 516 | 86 | hsa-miR-20a-5p | [LMO3](http://www.ncbi.nlm.nih.gov/entrez/query.fcgi?db=gene&cmd=Retrieve&dopt=full_report&list_uids=55885) | LIM domain only 3 |
| [Details](https://mirdb.org/cgi-bin/target_detail.cgi?targetID=2855037) | 517 | 86 | hsa-miR-20a-5p | [AKAP13](http://www.ncbi.nlm.nih.gov/entrez/query.fcgi?db=gene&cmd=Retrieve&dopt=full_report&list_uids=11214) | A-kinase anchoring protein 13 |
| [Details](https://mirdb.org/cgi-bin/target_detail.cgi?targetID=2855057) | 518 | 86 | hsa-miR-20a-5p | [HLF](http://www.ncbi.nlm.nih.gov/entrez/query.fcgi?db=gene&cmd=Retrieve&dopt=full_report&list_uids=3131) | HLF, PAR bZIP transcription factor |
| [Details](https://mirdb.org/cgi-bin/target_detail.cgi?targetID=2855078) | 519 | 86 | hsa-miR-20a-5p | [CNOT7](http://www.ncbi.nlm.nih.gov/entrez/query.fcgi?db=gene&cmd=Retrieve&dopt=full_report&list_uids=29883) | CCR4-NOT transcription complex subunit 7 |
| [Details](https://mirdb.org/cgi-bin/target_detail.cgi?targetID=2855128) | 520 | 86 | hsa-miR-20a-5p | [RETREG2](http://www.ncbi.nlm.nih.gov/entrez/query.fcgi?db=gene&cmd=Retrieve&dopt=full_report&list_uids=79137) | reticulophagy regulator family member 2 |
| [Details](https://mirdb.org/cgi-bin/target_detail.cgi?targetID=2855142) | 521 | 86 | hsa-miR-20a-5p | [AHNAK](http://www.ncbi.nlm.nih.gov/entrez/query.fcgi?db=gene&cmd=Retrieve&dopt=full_report&list_uids=79026) | AHNAK nucleoprotein |
| [Details](https://mirdb.org/cgi-bin/target_detail.cgi?targetID=2855148) | 522 | 86 | hsa-miR-20a-5p | [MARCH8](http://www.ncbi.nlm.nih.gov/entrez/query.fcgi?db=gene&cmd=Retrieve&dopt=full_report&list_uids=220972) | membrane associated ring-CH-type finger 8 |
| [Details](https://mirdb.org/cgi-bin/target_detail.cgi?targetID=2855167) | 523 | 86 | hsa-miR-20a-5p | [PAPOLA](http://www.ncbi.nlm.nih.gov/entrez/query.fcgi?db=gene&cmd=Retrieve&dopt=full_report&list_uids=10914) | poly(A) polymerase alpha |
| [Details](https://mirdb.org/cgi-bin/target_detail.cgi?targetID=2855268) | 524 | 86 | hsa-miR-20a-5p | [STK11](http://www.ncbi.nlm.nih.gov/entrez/query.fcgi?db=gene&cmd=Retrieve&dopt=full_report&list_uids=6794) | serine/threonine kinase 11 |
| [Details](https://mirdb.org/cgi-bin/target_detail.cgi?targetID=2855317) | 525 | 86 | hsa-miR-20a-5p | [EPS15L1](http://www.ncbi.nlm.nih.gov/entrez/query.fcgi?db=gene&cmd=Retrieve&dopt=full_report&list_uids=58513) | epidermal growth factor receptor pathway substrate 15 like 1 |
| [Details](https://mirdb.org/cgi-bin/target_detail.cgi?targetID=2855328) | 526 | 86 | hsa-miR-20a-5p | [PXYLP1](http://www.ncbi.nlm.nih.gov/entrez/query.fcgi?db=gene&cmd=Retrieve&dopt=full_report&list_uids=92370) | 2-phosphoxylose phosphatase 1 |
| [Details](https://mirdb.org/cgi-bin/target_detail.cgi?targetID=2855376) | 527 | 86 | hsa-miR-20a-5p | [MAP3K8](http://www.ncbi.nlm.nih.gov/entrez/query.fcgi?db=gene&cmd=Retrieve&dopt=full_report&list_uids=1326) | mitogen-activated protein kinase kinase kinase 8 |
| [Details](https://mirdb.org/cgi-bin/target_detail.cgi?targetID=2855390) | 528 | 86 | hsa-miR-20a-5p | [BAMBI](http://www.ncbi.nlm.nih.gov/entrez/query.fcgi?db=gene&cmd=Retrieve&dopt=full_report&list_uids=25805) | BMP and activin membrane bound inhibitor |
| [Details](https://mirdb.org/cgi-bin/target_detail.cgi?targetID=2855460) | 529 | 86 | hsa-miR-20a-5p | [SCAMP5](http://www.ncbi.nlm.nih.gov/entrez/query.fcgi?db=gene&cmd=Retrieve&dopt=full_report&list_uids=192683) | secretory carrier membrane protein 5 |
| [Details](https://mirdb.org/cgi-bin/target_detail.cgi?targetID=2854086) | 530 | 85 | hsa-miR-20a-5p | [PAPOLB](http://www.ncbi.nlm.nih.gov/entrez/query.fcgi?db=gene&cmd=Retrieve&dopt=full_report&list_uids=56903) | poly(A) polymerase beta |
| [Details](https://mirdb.org/cgi-bin/target_detail.cgi?targetID=2854116) | 531 | 85 | hsa-miR-20a-5p | [PLAG1](http://www.ncbi.nlm.nih.gov/entrez/query.fcgi?db=gene&cmd=Retrieve&dopt=full_report&list_uids=5324) | PLAG1 zinc finger |
| [Details](https://mirdb.org/cgi-bin/target_detail.cgi?targetID=2854134) | 532 | 85 | hsa-miR-20a-5p | [PSG3](http://www.ncbi.nlm.nih.gov/entrez/query.fcgi?db=gene&cmd=Retrieve&dopt=full_report&list_uids=5671) | pregnancy specific beta-1-glycoprotein 3 |
| [Details](https://mirdb.org/cgi-bin/target_detail.cgi?targetID=2854189) | 533 | 85 | hsa-miR-20a-5p | [NPLOC4](http://www.ncbi.nlm.nih.gov/entrez/query.fcgi?db=gene&cmd=Retrieve&dopt=full_report&list_uids=55666) | NPL4 homolog, ubiquitin recognition factor |
| [Details](https://mirdb.org/cgi-bin/target_detail.cgi?targetID=2854195) | 534 | 85 | hsa-miR-20a-5p | [TRDN](http://www.ncbi.nlm.nih.gov/entrez/query.fcgi?db=gene&cmd=Retrieve&dopt=full_report&list_uids=10345) | triadin |
| [Details](https://mirdb.org/cgi-bin/target_detail.cgi?targetID=2854216) | 535 | 85 | hsa-miR-20a-5p | [SLC30A7](http://www.ncbi.nlm.nih.gov/entrez/query.fcgi?db=gene&cmd=Retrieve&dopt=full_report&list_uids=148867) | solute carrier family 30 member 7 |
| [Details](https://mirdb.org/cgi-bin/target_detail.cgi?targetID=2854272) | 536 | 85 | hsa-miR-20a-5p | [TSPAN9](http://www.ncbi.nlm.nih.gov/entrez/query.fcgi?db=gene&cmd=Retrieve&dopt=full_report&list_uids=10867) | tetraspanin 9 |
| [Details](https://mirdb.org/cgi-bin/target_detail.cgi?targetID=2854289) | 537 | 85 | hsa-miR-20a-5p | [RNF6](http://www.ncbi.nlm.nih.gov/entrez/query.fcgi?db=gene&cmd=Retrieve&dopt=full_report&list_uids=6049) | ring finger protein 6 |
| [Details](https://mirdb.org/cgi-bin/target_detail.cgi?targetID=2854320) | 538 | 85 | hsa-miR-20a-5p | [SEPT2](http://www.ncbi.nlm.nih.gov/entrez/query.fcgi?db=gene&cmd=Retrieve&dopt=full_report&list_uids=4735) | septin 2 |
| [Details](https://mirdb.org/cgi-bin/target_detail.cgi?targetID=2854385) | 539 | 85 | hsa-miR-20a-5p | [LRPAP1](http://www.ncbi.nlm.nih.gov/entrez/query.fcgi?db=gene&cmd=Retrieve&dopt=full_report&list_uids=4043) | LDL receptor related protein associated protein 1 |
| [Details](https://mirdb.org/cgi-bin/target_detail.cgi?targetID=2854498) | 540 | 85 | hsa-miR-20a-5p | [UXS1](http://www.ncbi.nlm.nih.gov/entrez/query.fcgi?db=gene&cmd=Retrieve&dopt=full_report&list_uids=80146) | UDP-glucuronate decarboxylase 1 |
| [Details](https://mirdb.org/cgi-bin/target_detail.cgi?targetID=2854511) | 541 | 85 | hsa-miR-20a-5p | [UNK](http://www.ncbi.nlm.nih.gov/entrez/query.fcgi?db=gene&cmd=Retrieve&dopt=full_report&list_uids=85451) | unk zinc finger |
| [Details](https://mirdb.org/cgi-bin/target_detail.cgi?targetID=2854529) | 542 | 85 | hsa-miR-20a-5p | [PTPDC1](http://www.ncbi.nlm.nih.gov/entrez/query.fcgi?db=gene&cmd=Retrieve&dopt=full_report&list_uids=138639) | protein tyrosine phosphatase domain containing 1 |
| [Details](https://mirdb.org/cgi-bin/target_detail.cgi?targetID=2854595) | 543 | 85 | hsa-miR-20a-5p | [IKZF4](http://www.ncbi.nlm.nih.gov/entrez/query.fcgi?db=gene&cmd=Retrieve&dopt=full_report&list_uids=64375) | IKAROS family zinc finger 4 |
| [Details](https://mirdb.org/cgi-bin/target_detail.cgi?targetID=2854721) | 544 | 85 | hsa-miR-20a-5p | [RCCD1](http://www.ncbi.nlm.nih.gov/entrez/query.fcgi?db=gene&cmd=Retrieve&dopt=full_report&list_uids=91433) | RCC1 domain containing 1 |
| [Details](https://mirdb.org/cgi-bin/target_detail.cgi?targetID=2854726) | 545 | 85 | hsa-miR-20a-5p | [ARAP2](http://www.ncbi.nlm.nih.gov/entrez/query.fcgi?db=gene&cmd=Retrieve&dopt=full_report&list_uids=116984) | ArfGAP with RhoGAP domain, ankyrin repeat and PH domain 2 |
| [Details](https://mirdb.org/cgi-bin/target_detail.cgi?targetID=2854786) | 546 | 85 | hsa-miR-20a-5p | [MCHR2](http://www.ncbi.nlm.nih.gov/entrez/query.fcgi?db=gene&cmd=Retrieve&dopt=full_report&list_uids=84539) | melanin concentrating hormone receptor 2 |
| [Details](https://mirdb.org/cgi-bin/target_detail.cgi?targetID=2854789) | 547 | 85 | hsa-miR-20a-5p | [MAPK4](http://www.ncbi.nlm.nih.gov/entrez/query.fcgi?db=gene&cmd=Retrieve&dopt=full_report&list_uids=5596) | mitogen-activated protein kinase 4 |
| [Details](https://mirdb.org/cgi-bin/target_detail.cgi?targetID=2854842) | 548 | 85 | hsa-miR-20a-5p | [FLT1](http://www.ncbi.nlm.nih.gov/entrez/query.fcgi?db=gene&cmd=Retrieve&dopt=full_report&list_uids=2321) | fms related tyrosine kinase 1 |
| [Details](https://mirdb.org/cgi-bin/target_detail.cgi?targetID=2854878) | 549 | 85 | hsa-miR-20a-5p | [TMEM100](http://www.ncbi.nlm.nih.gov/entrez/query.fcgi?db=gene&cmd=Retrieve&dopt=full_report&list_uids=55273) | transmembrane protein 100 |
| [Details](https://mirdb.org/cgi-bin/target_detail.cgi?targetID=2854953) | 550 | 85 | hsa-miR-20a-5p | [SYTL4](http://www.ncbi.nlm.nih.gov/entrez/query.fcgi?db=gene&cmd=Retrieve&dopt=full_report&list_uids=94121) | synaptotagmin like 4 |
| [Details](https://mirdb.org/cgi-bin/target_detail.cgi?targetID=2854957) | 551 | 85 | hsa-miR-20a-5p | [PSD](http://www.ncbi.nlm.nih.gov/entrez/query.fcgi?db=gene&cmd=Retrieve&dopt=full_report&list_uids=5662) | pleckstrin and Sec7 domain containing |
| [Details](https://mirdb.org/cgi-bin/target_detail.cgi?targetID=2854976) | 552 | 85 | hsa-miR-20a-5p | [CNOT6](http://www.ncbi.nlm.nih.gov/entrez/query.fcgi?db=gene&cmd=Retrieve&dopt=full_report&list_uids=57472) | CCR4-NOT transcription complex subunit 6 |
| [Details](https://mirdb.org/cgi-bin/target_detail.cgi?targetID=2855006) | 553 | 85 | hsa-miR-20a-5p | [KIF26B](http://www.ncbi.nlm.nih.gov/entrez/query.fcgi?db=gene&cmd=Retrieve&dopt=full_report&list_uids=55083) | kinesin family member 26B |
| [Details](https://mirdb.org/cgi-bin/target_detail.cgi?targetID=2855070) | 554 | 85 | hsa-miR-20a-5p | [PPP3R1](http://www.ncbi.nlm.nih.gov/entrez/query.fcgi?db=gene&cmd=Retrieve&dopt=full_report&list_uids=5534) | protein phosphatase 3 regulatory subunit B, alpha |
| [Details](https://mirdb.org/cgi-bin/target_detail.cgi?targetID=2855116) | 555 | 85 | hsa-miR-20a-5p | [SLC49A4](http://www.ncbi.nlm.nih.gov/entrez/query.fcgi?db=gene&cmd=Retrieve&dopt=full_report&list_uids=84925) | solute carrier family 49 member 4 |
| [Details](https://mirdb.org/cgi-bin/target_detail.cgi?targetID=2855164) | 556 | 85 | hsa-miR-20a-5p | [USP24](http://www.ncbi.nlm.nih.gov/entrez/query.fcgi?db=gene&cmd=Retrieve&dopt=full_report&list_uids=23358) | ubiquitin specific peptidase 24 |
| [Details](https://mirdb.org/cgi-bin/target_detail.cgi?targetID=2855295) | 557 | 85 | hsa-miR-20a-5p | [TP73](http://www.ncbi.nlm.nih.gov/entrez/query.fcgi?db=gene&cmd=Retrieve&dopt=full_report&list_uids=7161) | tumor protein p73 |
| [Details](https://mirdb.org/cgi-bin/target_detail.cgi?targetID=2855329) | 558 | 85 | hsa-miR-20a-5p | [PTGDR](http://www.ncbi.nlm.nih.gov/entrez/query.fcgi?db=gene&cmd=Retrieve&dopt=full_report&list_uids=5729) | prostaglandin D2 receptor |
| [Details](https://mirdb.org/cgi-bin/target_detail.cgi?targetID=2855339) | 559 | 85 | hsa-miR-20a-5p | [FAM13C](http://www.ncbi.nlm.nih.gov/entrez/query.fcgi?db=gene&cmd=Retrieve&dopt=full_report&list_uids=220965) | family with sequence similarity 13 member C |
| [Details](https://mirdb.org/cgi-bin/target_detail.cgi?targetID=2855422) | 560 | 85 | hsa-miR-20a-5p | [DDIAS](http://www.ncbi.nlm.nih.gov/entrez/query.fcgi?db=gene&cmd=Retrieve&dopt=full_report&list_uids=220042) | DNA damage induced apoptosis suppressor |
| [Details](https://mirdb.org/cgi-bin/target_detail.cgi?targetID=2854089) | 561 | 84 | hsa-miR-20a-5p | [ZNF25](http://www.ncbi.nlm.nih.gov/entrez/query.fcgi?db=gene&cmd=Retrieve&dopt=full_report&list_uids=219749) | zinc finger protein 25 |
| [Details](https://mirdb.org/cgi-bin/target_detail.cgi?targetID=2854127) | 562 | 84 | hsa-miR-20a-5p | [SH3BP5](http://www.ncbi.nlm.nih.gov/entrez/query.fcgi?db=gene&cmd=Retrieve&dopt=full_report&list_uids=9467) | SH3 domain binding protein 5 |
| [Details](https://mirdb.org/cgi-bin/target_detail.cgi?targetID=2854229) | 563 | 84 | hsa-miR-20a-5p | [GABPB1](http://www.ncbi.nlm.nih.gov/entrez/query.fcgi?db=gene&cmd=Retrieve&dopt=full_report&list_uids=2553) | GA binding protein transcription factor subunit beta 1 |
| [Details](https://mirdb.org/cgi-bin/target_detail.cgi?targetID=2854300) | 564 | 84 | hsa-miR-20a-5p | [OSBPL5](http://www.ncbi.nlm.nih.gov/entrez/query.fcgi?db=gene&cmd=Retrieve&dopt=full_report&list_uids=114879) | oxysterol binding protein like 5 |
| [Details](https://mirdb.org/cgi-bin/target_detail.cgi?targetID=2854325) | 565 | 84 | hsa-miR-20a-5p | [RNH1](http://www.ncbi.nlm.nih.gov/entrez/query.fcgi?db=gene&cmd=Retrieve&dopt=full_report&list_uids=6050) | ribonuclease/angiogenin inhibitor 1 |
| [Details](https://mirdb.org/cgi-bin/target_detail.cgi?targetID=2854505) | 566 | 84 | hsa-miR-20a-5p | [FASTK](http://www.ncbi.nlm.nih.gov/entrez/query.fcgi?db=gene&cmd=Retrieve&dopt=full_report&list_uids=10922) | Fas activated serine/threonine kinase |
| [Details](https://mirdb.org/cgi-bin/target_detail.cgi?targetID=2854533) | 567 | 84 | hsa-miR-20a-5p | [ZBTB8A](http://www.ncbi.nlm.nih.gov/entrez/query.fcgi?db=gene&cmd=Retrieve&dopt=full_report&list_uids=653121) | zinc finger and BTB domain containing 8A |
| [Details](https://mirdb.org/cgi-bin/target_detail.cgi?targetID=2854546) | 568 | 84 | hsa-miR-20a-5p | [ZNF597](http://www.ncbi.nlm.nih.gov/entrez/query.fcgi?db=gene&cmd=Retrieve&dopt=full_report&list_uids=146434) | zinc finger protein 597 |
| [Details](https://mirdb.org/cgi-bin/target_detail.cgi?targetID=2854551) | 569 | 84 | hsa-miR-20a-5p | [SOCS6](http://www.ncbi.nlm.nih.gov/entrez/query.fcgi?db=gene&cmd=Retrieve&dopt=full_report&list_uids=9306) | suppressor of cytokine signaling 6 |
| [Details](https://mirdb.org/cgi-bin/target_detail.cgi?targetID=2854620) | 570 | 84 | hsa-miR-20a-5p | [RAB12](http://www.ncbi.nlm.nih.gov/entrez/query.fcgi?db=gene&cmd=Retrieve&dopt=full_report&list_uids=201475) | RAB12, member RAS oncogene family |
| [Details](https://mirdb.org/cgi-bin/target_detail.cgi?targetID=2854687) | 571 | 84 | hsa-miR-20a-5p | [GRAMD1A](http://www.ncbi.nlm.nih.gov/entrez/query.fcgi?db=gene&cmd=Retrieve&dopt=full_report&list_uids=57655) | GRAM domain containing 1A |
| [Details](https://mirdb.org/cgi-bin/target_detail.cgi?targetID=2854708) | 572 | 84 | hsa-miR-20a-5p | [TBC1D8B](http://www.ncbi.nlm.nih.gov/entrez/query.fcgi?db=gene&cmd=Retrieve&dopt=full_report&list_uids=54885) | TBC1 domain family member 8B |
| [Details](https://mirdb.org/cgi-bin/target_detail.cgi?targetID=2854770) | 573 | 84 | hsa-miR-20a-5p | [MAP3K20](http://www.ncbi.nlm.nih.gov/entrez/query.fcgi?db=gene&cmd=Retrieve&dopt=full_report&list_uids=51776) | mitogen-activated protein kinase kinase kinase 20 |
| [Details](https://mirdb.org/cgi-bin/target_detail.cgi?targetID=2854843) | 574 | 84 | hsa-miR-20a-5p | [TENM1](http://www.ncbi.nlm.nih.gov/entrez/query.fcgi?db=gene&cmd=Retrieve&dopt=full_report&list_uids=10178) | teneurin transmembrane protein 1 |
| [Details](https://mirdb.org/cgi-bin/target_detail.cgi?targetID=2854915) | 575 | 84 | hsa-miR-20a-5p | [PTPN21](http://www.ncbi.nlm.nih.gov/entrez/query.fcgi?db=gene&cmd=Retrieve&dopt=full_report&list_uids=11099) | protein tyrosine phosphatase, non-receptor type 21 |
| [Details](https://mirdb.org/cgi-bin/target_detail.cgi?targetID=2854936) | 576 | 84 | hsa-miR-20a-5p | [BICD2](http://www.ncbi.nlm.nih.gov/entrez/query.fcgi?db=gene&cmd=Retrieve&dopt=full_report&list_uids=23299) | BICD cargo adaptor 2 |
| [Details](https://mirdb.org/cgi-bin/target_detail.cgi?targetID=2854991) | 577 | 84 | hsa-miR-20a-5p | [MCF2L](http://www.ncbi.nlm.nih.gov/entrez/query.fcgi?db=gene&cmd=Retrieve&dopt=full_report&list_uids=23263) | MCF.2 cell line derived transforming sequence like |
| [Details](https://mirdb.org/cgi-bin/target_detail.cgi?targetID=2855048) | 578 | 84 | hsa-miR-20a-5p | [DUSP8](http://www.ncbi.nlm.nih.gov/entrez/query.fcgi?db=gene&cmd=Retrieve&dopt=full_report&list_uids=1850) | dual specificity phosphatase 8 |
| [Details](https://mirdb.org/cgi-bin/target_detail.cgi?targetID=2855083) | 579 | 84 | hsa-miR-20a-5p | [ARHGEF3](http://www.ncbi.nlm.nih.gov/entrez/query.fcgi?db=gene&cmd=Retrieve&dopt=full_report&list_uids=50650) | Rho guanine nucleotide exchange factor 3 |
| [Details](https://mirdb.org/cgi-bin/target_detail.cgi?targetID=2855152) | 580 | 84 | hsa-miR-20a-5p | [ST6GALNAC3](http://www.ncbi.nlm.nih.gov/entrez/query.fcgi?db=gene&cmd=Retrieve&dopt=full_report&list_uids=256435) | ST6 N-acetylgalactosaminide alpha-2,6-sialyltransferase 3 |
| [Details](https://mirdb.org/cgi-bin/target_detail.cgi?targetID=2855225) | 581 | 84 | hsa-miR-20a-5p | [PRR16](http://www.ncbi.nlm.nih.gov/entrez/query.fcgi?db=gene&cmd=Retrieve&dopt=full_report&list_uids=51334) | proline rich 16 |
| [Details](https://mirdb.org/cgi-bin/target_detail.cgi?targetID=2855243) | 582 | 84 | hsa-miR-20a-5p | [LCOR](http://www.ncbi.nlm.nih.gov/entrez/query.fcgi?db=gene&cmd=Retrieve&dopt=full_report&list_uids=84458) | ligand dependent nuclear receptor corepressor |
| [Details](https://mirdb.org/cgi-bin/target_detail.cgi?targetID=2855273) | 583 | 84 | hsa-miR-20a-5p | [SLITRK2](http://www.ncbi.nlm.nih.gov/entrez/query.fcgi?db=gene&cmd=Retrieve&dopt=full_report&list_uids=84631) | SLIT and NTRK like family member 2 |
| [Details](https://mirdb.org/cgi-bin/target_detail.cgi?targetID=2855279) | 584 | 84 | hsa-miR-20a-5p | [NEUROG1](http://www.ncbi.nlm.nih.gov/entrez/query.fcgi?db=gene&cmd=Retrieve&dopt=full_report&list_uids=4762) | neurogenin 1 |
| [Details](https://mirdb.org/cgi-bin/target_detail.cgi?targetID=2855320) | 585 | 84 | hsa-miR-20a-5p | [C6orf120](http://www.ncbi.nlm.nih.gov/entrez/query.fcgi?db=gene&cmd=Retrieve&dopt=full_report&list_uids=387263) | chromosome 6 open reading frame 120 |
| [Details](https://mirdb.org/cgi-bin/target_detail.cgi?targetID=2855386) | 586 | 84 | hsa-miR-20a-5p | [NHLRC3](http://www.ncbi.nlm.nih.gov/entrez/query.fcgi?db=gene&cmd=Retrieve&dopt=full_report&list_uids=387921) | NHL repeat containing 3 |
| [Details](https://mirdb.org/cgi-bin/target_detail.cgi?targetID=2855393) | 587 | 84 | hsa-miR-20a-5p | [TAGAP](http://www.ncbi.nlm.nih.gov/entrez/query.fcgi?db=gene&cmd=Retrieve&dopt=full_report&list_uids=117289) | T cell activation RhoGTPase activating protein |
| [Details](https://mirdb.org/cgi-bin/target_detail.cgi?targetID=2855445) | 588 | 84 | hsa-miR-20a-5p | [MAP7](http://www.ncbi.nlm.nih.gov/entrez/query.fcgi?db=gene&cmd=Retrieve&dopt=full_report&list_uids=9053) | microtubule associated protein 7 |
| [Details](https://mirdb.org/cgi-bin/target_detail.cgi?targetID=2855454) | 589 | 84 | hsa-miR-20a-5p | [C3orf70](http://www.ncbi.nlm.nih.gov/entrez/query.fcgi?db=gene&cmd=Retrieve&dopt=full_report&list_uids=285382) | chromosome 3 open reading frame 70 |
| [Details](https://mirdb.org/cgi-bin/target_detail.cgi?targetID=2854092) | 590 | 83 | hsa-miR-20a-5p | [SCN2B](http://www.ncbi.nlm.nih.gov/entrez/query.fcgi?db=gene&cmd=Retrieve&dopt=full_report&list_uids=6327) | sodium voltage-gated channel beta subunit 2 |
| [Details](https://mirdb.org/cgi-bin/target_detail.cgi?targetID=2854095) | 591 | 83 | hsa-miR-20a-5p | [TRIM37](http://www.ncbi.nlm.nih.gov/entrez/query.fcgi?db=gene&cmd=Retrieve&dopt=full_report&list_uids=4591) | tripartite motif containing 37 |
| [Details](https://mirdb.org/cgi-bin/target_detail.cgi?targetID=2854152) | 592 | 83 | hsa-miR-20a-5p | [CBLN4](http://www.ncbi.nlm.nih.gov/entrez/query.fcgi?db=gene&cmd=Retrieve&dopt=full_report&list_uids=140689) | cerebellin 4 precursor |
| [Details](https://mirdb.org/cgi-bin/target_detail.cgi?targetID=2854193) | 593 | 83 | hsa-miR-20a-5p | [SAMD8](http://www.ncbi.nlm.nih.gov/entrez/query.fcgi?db=gene&cmd=Retrieve&dopt=full_report&list_uids=142891) | sterile alpha motif domain containing 8 |
| [Details](https://mirdb.org/cgi-bin/target_detail.cgi?targetID=2854210) | 594 | 83 | hsa-miR-20a-5p | [ARHGEF28](http://www.ncbi.nlm.nih.gov/entrez/query.fcgi?db=gene&cmd=Retrieve&dopt=full_report&list_uids=64283) | Rho guanine nucleotide exchange factor 28 |
| [Details](https://mirdb.org/cgi-bin/target_detail.cgi?targetID=2854215) | 595 | 83 | hsa-miR-20a-5p | [DDX5](http://www.ncbi.nlm.nih.gov/entrez/query.fcgi?db=gene&cmd=Retrieve&dopt=full_report&list_uids=1655) | DEAD-box helicase 5 |
| [Details](https://mirdb.org/cgi-bin/target_detail.cgi?targetID=2854433) | 596 | 83 | hsa-miR-20a-5p | [MTF1](http://www.ncbi.nlm.nih.gov/entrez/query.fcgi?db=gene&cmd=Retrieve&dopt=full_report&list_uids=4520) | metal regulatory transcription factor 1 |
| [Details](https://mirdb.org/cgi-bin/target_detail.cgi?targetID=2854438) | 597 | 83 | hsa-miR-20a-5p | [NFIC](http://www.ncbi.nlm.nih.gov/entrez/query.fcgi?db=gene&cmd=Retrieve&dopt=full_report&list_uids=4782) | nuclear factor I C |
| [Details](https://mirdb.org/cgi-bin/target_detail.cgi?targetID=2854465) | 598 | 83 | hsa-miR-20a-5p | [PFKFB3](http://www.ncbi.nlm.nih.gov/entrez/query.fcgi?db=gene&cmd=Retrieve&dopt=full_report&list_uids=5209) | 6-phosphofructo-2-kinase/fructose-2,6-biphosphatase 3 |
| [Details](https://mirdb.org/cgi-bin/target_detail.cgi?targetID=2854493) | 599 | 83 | hsa-miR-20a-5p | [ABHD2](http://www.ncbi.nlm.nih.gov/entrez/query.fcgi?db=gene&cmd=Retrieve&dopt=full_report&list_uids=11057) | abhydrolase domain containing 2 |
| [Details](https://mirdb.org/cgi-bin/target_detail.cgi?targetID=2854508) | 600 | 83 | hsa-miR-20a-5p | [SMAD4](http://www.ncbi.nlm.nih.gov/entrez/query.fcgi?db=gene&cmd=Retrieve&dopt=full_report&list_uids=4089) | SMAD family member 4 |
| [Details](https://mirdb.org/cgi-bin/target_detail.cgi?targetID=2854517) | 601 | 83 | hsa-miR-20a-5p | [USP6](http://www.ncbi.nlm.nih.gov/entrez/query.fcgi?db=gene&cmd=Retrieve&dopt=full_report&list_uids=9098) | ubiquitin specific peptidase 6 |
| [Details](https://mirdb.org/cgi-bin/target_detail.cgi?targetID=2854577) | 602 | 83 | hsa-miR-20a-5p | [ZBTB47](http://www.ncbi.nlm.nih.gov/entrez/query.fcgi?db=gene&cmd=Retrieve&dopt=full_report&list_uids=92999) | zinc finger and BTB domain containing 47 |
| [Details](https://mirdb.org/cgi-bin/target_detail.cgi?targetID=2854647) | 603 | 83 | hsa-miR-20a-5p | [KATNAL1](http://www.ncbi.nlm.nih.gov/entrez/query.fcgi?db=gene&cmd=Retrieve&dopt=full_report&list_uids=84056) | katanin catalytic subunit A1 like 1 |
| [Details](https://mirdb.org/cgi-bin/target_detail.cgi?targetID=2854694) | 604 | 83 | hsa-miR-20a-5p | [ZHX2](http://www.ncbi.nlm.nih.gov/entrez/query.fcgi?db=gene&cmd=Retrieve&dopt=full_report&list_uids=22882) | zinc fingers and homeoboxes 2 |
| [Details](https://mirdb.org/cgi-bin/target_detail.cgi?targetID=2854722) | 605 | 83 | hsa-miR-20a-5p | [MYNN](http://www.ncbi.nlm.nih.gov/entrez/query.fcgi?db=gene&cmd=Retrieve&dopt=full_report&list_uids=55892) | myoneurin |
| [Details](https://mirdb.org/cgi-bin/target_detail.cgi?targetID=2854800) | 606 | 83 | hsa-miR-20a-5p | [RNASEH2B](http://www.ncbi.nlm.nih.gov/entrez/query.fcgi?db=gene&cmd=Retrieve&dopt=full_report&list_uids=79621) | ribonuclease H2 subunit B |
| [Details](https://mirdb.org/cgi-bin/target_detail.cgi?targetID=2854847) | 607 | 83 | hsa-miR-20a-5p | [DNAJB9](http://www.ncbi.nlm.nih.gov/entrez/query.fcgi?db=gene&cmd=Retrieve&dopt=full_report&list_uids=4189) | DnaJ heat shock protein family (Hsp40) member B9 |
| [Details](https://mirdb.org/cgi-bin/target_detail.cgi?targetID=2854885) | 608 | 83 | hsa-miR-20a-5p | [UBE2D1](http://www.ncbi.nlm.nih.gov/entrez/query.fcgi?db=gene&cmd=Retrieve&dopt=full_report&list_uids=7321) | ubiquitin conjugating enzyme E2 D1 |
| [Details](https://mirdb.org/cgi-bin/target_detail.cgi?targetID=2854897) | 609 | 83 | hsa-miR-20a-5p | [WWP2](http://www.ncbi.nlm.nih.gov/entrez/query.fcgi?db=gene&cmd=Retrieve&dopt=full_report&list_uids=11060) | WW domain containing E3 ubiquitin protein ligase 2 |
| [Details](https://mirdb.org/cgi-bin/target_detail.cgi?targetID=2854903) | 610 | 83 | hsa-miR-20a-5p | [C2orf69](http://www.ncbi.nlm.nih.gov/entrez/query.fcgi?db=gene&cmd=Retrieve&dopt=full_report&list_uids=205327) | chromosome 2 open reading frame 69 |
| [Details](https://mirdb.org/cgi-bin/target_detail.cgi?targetID=2854956) | 611 | 83 | hsa-miR-20a-5p | [TMEM25](http://www.ncbi.nlm.nih.gov/entrez/query.fcgi?db=gene&cmd=Retrieve&dopt=full_report&list_uids=84866) | transmembrane protein 25 |
| [Details](https://mirdb.org/cgi-bin/target_detail.cgi?targetID=2855045) | 612 | 83 | hsa-miR-20a-5p | [POLR3G](http://www.ncbi.nlm.nih.gov/entrez/query.fcgi?db=gene&cmd=Retrieve&dopt=full_report&list_uids=10622) | RNA polymerase III subunit G |
| [Details](https://mirdb.org/cgi-bin/target_detail.cgi?targetID=2855120) | 613 | 83 | hsa-miR-20a-5p | [PARD6B](http://www.ncbi.nlm.nih.gov/entrez/query.fcgi?db=gene&cmd=Retrieve&dopt=full_report&list_uids=84612) | par-6 family cell polarity regulator beta |
| [Details](https://mirdb.org/cgi-bin/target_detail.cgi?targetID=2855199) | 614 | 83 | hsa-miR-20a-5p | [RBL1](http://www.ncbi.nlm.nih.gov/entrez/query.fcgi?db=gene&cmd=Retrieve&dopt=full_report&list_uids=5933) | RB transcriptional corepressor like 1 |
| [Details](https://mirdb.org/cgi-bin/target_detail.cgi?targetID=2855223) | 615 | 83 | hsa-miR-20a-5p | [BCL2L11](http://www.ncbi.nlm.nih.gov/entrez/query.fcgi?db=gene&cmd=Retrieve&dopt=full_report&list_uids=10018) | BCL2 like 11 |
| [Details](https://mirdb.org/cgi-bin/target_detail.cgi?targetID=2855259) | 616 | 83 | hsa-miR-20a-5p | [SCAMP2](http://www.ncbi.nlm.nih.gov/entrez/query.fcgi?db=gene&cmd=Retrieve&dopt=full_report&list_uids=10066) | secretory carrier membrane protein 2 |
| [Details](https://mirdb.org/cgi-bin/target_detail.cgi?targetID=2855357) | 617 | 83 | hsa-miR-20a-5p | [KANSL1L](http://www.ncbi.nlm.nih.gov/entrez/query.fcgi?db=gene&cmd=Retrieve&dopt=full_report&list_uids=151050) | KAT8 regulatory NSL complex subunit 1 like |
| [Details](https://mirdb.org/cgi-bin/target_detail.cgi?targetID=2855415) | 618 | 83 | hsa-miR-20a-5p | [CDC23](http://www.ncbi.nlm.nih.gov/entrez/query.fcgi?db=gene&cmd=Retrieve&dopt=full_report&list_uids=8697) | cell division cycle 23 |
| [Details](https://mirdb.org/cgi-bin/target_detail.cgi?targetID=2855450) | 619 | 83 | hsa-miR-20a-5p | [VCPKMT](http://www.ncbi.nlm.nih.gov/entrez/query.fcgi?db=gene&cmd=Retrieve&dopt=full_report&list_uids=79609) | valosin containing protein lysine methyltransferase |
| [Details](https://mirdb.org/cgi-bin/target_detail.cgi?targetID=2854276) | 620 | 82 | hsa-miR-20a-5p | [EIF4A2](http://www.ncbi.nlm.nih.gov/entrez/query.fcgi?db=gene&cmd=Retrieve&dopt=full_report&list_uids=1974) | eukaryotic translation initiation factor 4A2 |
| [Details](https://mirdb.org/cgi-bin/target_detail.cgi?targetID=2854375) | 621 | 82 | hsa-miR-20a-5p | [PLCB1](http://www.ncbi.nlm.nih.gov/entrez/query.fcgi?db=gene&cmd=Retrieve&dopt=full_report&list_uids=23236) | phospholipase C beta 1 |
| [Details](https://mirdb.org/cgi-bin/target_detail.cgi?targetID=2854394) | 622 | 82 | hsa-miR-20a-5p | [CNOT6L](http://www.ncbi.nlm.nih.gov/entrez/query.fcgi?db=gene&cmd=Retrieve&dopt=full_report&list_uids=246175) | CCR4-NOT transcription complex subunit 6 like |
| [Details](https://mirdb.org/cgi-bin/target_detail.cgi?targetID=2854478) | 623 | 82 | hsa-miR-20a-5p | [CDKN1A](http://www.ncbi.nlm.nih.gov/entrez/query.fcgi?db=gene&cmd=Retrieve&dopt=full_report&list_uids=1026) | cyclin dependent kinase inhibitor 1A |
| [Details](https://mirdb.org/cgi-bin/target_detail.cgi?targetID=2854541) | 624 | 82 | hsa-miR-20a-5p | [NFIB](http://www.ncbi.nlm.nih.gov/entrez/query.fcgi?db=gene&cmd=Retrieve&dopt=full_report&list_uids=4781) | nuclear factor I B |
| [Details](https://mirdb.org/cgi-bin/target_detail.cgi?targetID=2854556) | 625 | 82 | hsa-miR-20a-5p | [ZDHHC9](http://www.ncbi.nlm.nih.gov/entrez/query.fcgi?db=gene&cmd=Retrieve&dopt=full_report&list_uids=51114) | zinc finger DHHC-type containing 9 |
| [Details](https://mirdb.org/cgi-bin/target_detail.cgi?targetID=2854566) | 626 | 82 | hsa-miR-20a-5p | [GABBR2](http://www.ncbi.nlm.nih.gov/entrez/query.fcgi?db=gene&cmd=Retrieve&dopt=full_report&list_uids=9568) | gamma-aminobutyric acid type B receptor subunit 2 |
| [Details](https://mirdb.org/cgi-bin/target_detail.cgi?targetID=2854707) | 627 | 82 | hsa-miR-20a-5p | [SORL1](http://www.ncbi.nlm.nih.gov/entrez/query.fcgi?db=gene&cmd=Retrieve&dopt=full_report&list_uids=6653) | sortilin related receptor 1 |
| [Details](https://mirdb.org/cgi-bin/target_detail.cgi?targetID=2854769) | 628 | 82 | hsa-miR-20a-5p | [PRCP](http://www.ncbi.nlm.nih.gov/entrez/query.fcgi?db=gene&cmd=Retrieve&dopt=full_report&list_uids=5547) | prolylcarboxypeptidase |
| [Details](https://mirdb.org/cgi-bin/target_detail.cgi?targetID=2854788) | 629 | 82 | hsa-miR-20a-5p | [PAFAH1B1](http://www.ncbi.nlm.nih.gov/entrez/query.fcgi?db=gene&cmd=Retrieve&dopt=full_report&list_uids=5048) | platelet activating factor acetylhydrolase 1b regulatory subunit 1 |
| [Details](https://mirdb.org/cgi-bin/target_detail.cgi?targetID=2854829) | 630 | 82 | hsa-miR-20a-5p | [PRR14L](http://www.ncbi.nlm.nih.gov/entrez/query.fcgi?db=gene&cmd=Retrieve&dopt=full_report&list_uids=253143) | proline rich 14 like |
| [Details](https://mirdb.org/cgi-bin/target_detail.cgi?targetID=2854833) | 631 | 82 | hsa-miR-20a-5p | [PAG1](http://www.ncbi.nlm.nih.gov/entrez/query.fcgi?db=gene&cmd=Retrieve&dopt=full_report&list_uids=55824) | phosphoprotein membrane anchor with glycosphingolipid microdomains 1 |
| [Details](https://mirdb.org/cgi-bin/target_detail.cgi?targetID=2854872) | 632 | 82 | hsa-miR-20a-5p | [MMP2](http://www.ncbi.nlm.nih.gov/entrez/query.fcgi?db=gene&cmd=Retrieve&dopt=full_report&list_uids=4313) | matrix metallopeptidase 2 |
| [Details](https://mirdb.org/cgi-bin/target_detail.cgi?targetID=2854879) | 633 | 82 | hsa-miR-20a-5p | [KCNJ10](http://www.ncbi.nlm.nih.gov/entrez/query.fcgi?db=gene&cmd=Retrieve&dopt=full_report&list_uids=3766) | potassium voltage-gated channel subfamily J member 10 |
| [Details](https://mirdb.org/cgi-bin/target_detail.cgi?targetID=2855007) | 634 | 82 | hsa-miR-20a-5p | [TBC1D17](http://www.ncbi.nlm.nih.gov/entrez/query.fcgi?db=gene&cmd=Retrieve&dopt=full_report&list_uids=79735) | TBC1 domain family member 17 |
| [Details](https://mirdb.org/cgi-bin/target_detail.cgi?targetID=2855021) | 635 | 82 | hsa-miR-20a-5p | [EREG](http://www.ncbi.nlm.nih.gov/entrez/query.fcgi?db=gene&cmd=Retrieve&dopt=full_report&list_uids=2069) | epiregulin |
| [Details](https://mirdb.org/cgi-bin/target_detail.cgi?targetID=2855029) | 636 | 82 | hsa-miR-20a-5p | [ZADH2](http://www.ncbi.nlm.nih.gov/entrez/query.fcgi?db=gene&cmd=Retrieve&dopt=full_report&list_uids=284273) | zinc binding alcohol dehydrogenase domain containing 2 |
| [Details](https://mirdb.org/cgi-bin/target_detail.cgi?targetID=2855176) | 637 | 82 | hsa-miR-20a-5p | [KLF12](http://www.ncbi.nlm.nih.gov/entrez/query.fcgi?db=gene&cmd=Retrieve&dopt=full_report&list_uids=11278) | Kruppel like factor 12 |
| [Details](https://mirdb.org/cgi-bin/target_detail.cgi?targetID=2855182) | 638 | 82 | hsa-miR-20a-5p | [NEUROG2](http://www.ncbi.nlm.nih.gov/entrez/query.fcgi?db=gene&cmd=Retrieve&dopt=full_report&list_uids=63973) | neurogenin 2 |
| [Details](https://mirdb.org/cgi-bin/target_detail.cgi?targetID=2855286) | 639 | 82 | hsa-miR-20a-5p | [SPTY2D1](http://www.ncbi.nlm.nih.gov/entrez/query.fcgi?db=gene&cmd=Retrieve&dopt=full_report&list_uids=144108) | SPT2 chromatin protein domain containing 1 |
| [Details](https://mirdb.org/cgi-bin/target_detail.cgi?targetID=2855310) | 640 | 82 | hsa-miR-20a-5p | [GNS](http://www.ncbi.nlm.nih.gov/entrez/query.fcgi?db=gene&cmd=Retrieve&dopt=full_report&list_uids=2799) | glucosamine (N-acetyl)-6-sulfatase |
| [Details](https://mirdb.org/cgi-bin/target_detail.cgi?targetID=2855319) | 641 | 82 | hsa-miR-20a-5p | [PLS1](http://www.ncbi.nlm.nih.gov/entrez/query.fcgi?db=gene&cmd=Retrieve&dopt=full_report&list_uids=5357) | plastin 1 |
| [Details](https://mirdb.org/cgi-bin/target_detail.cgi?targetID=2855342) | 642 | 82 | hsa-miR-20a-5p | [ZNF280B](http://www.ncbi.nlm.nih.gov/entrez/query.fcgi?db=gene&cmd=Retrieve&dopt=full_report&list_uids=140883) | zinc finger protein 280B |
| [Details](https://mirdb.org/cgi-bin/target_detail.cgi?targetID=2855358) | 643 | 82 | hsa-miR-20a-5p | [VASP](http://www.ncbi.nlm.nih.gov/entrez/query.fcgi?db=gene&cmd=Retrieve&dopt=full_report&list_uids=7408) | vasodilator stimulated phosphoprotein |
| [Details](https://mirdb.org/cgi-bin/target_detail.cgi?targetID=2854146) | 644 | 81 | hsa-miR-20a-5p | [RASA2](http://www.ncbi.nlm.nih.gov/entrez/query.fcgi?db=gene&cmd=Retrieve&dopt=full_report&list_uids=5922) | RAS p21 protein activator 2 |
| [Details](https://mirdb.org/cgi-bin/target_detail.cgi?targetID=2854161) | 645 | 81 | hsa-miR-20a-5p | [ABCD2](http://www.ncbi.nlm.nih.gov/entrez/query.fcgi?db=gene&cmd=Retrieve&dopt=full_report&list_uids=225) | ATP binding cassette subfamily D member 2 |
| [Details](https://mirdb.org/cgi-bin/target_detail.cgi?targetID=2854196) | 646 | 81 | hsa-miR-20a-5p | [MSR1](http://www.ncbi.nlm.nih.gov/entrez/query.fcgi?db=gene&cmd=Retrieve&dopt=full_report&list_uids=4481) | macrophage scavenger receptor 1 |
| [Details](https://mirdb.org/cgi-bin/target_detail.cgi?targetID=2854604) | 647 | 81 | hsa-miR-20a-5p | [SOWAHC](http://www.ncbi.nlm.nih.gov/entrez/query.fcgi?db=gene&cmd=Retrieve&dopt=full_report&list_uids=65124) | sosondowah ankyrin repeat domain family member C |
| [Details](https://mirdb.org/cgi-bin/target_detail.cgi?targetID=2854672) | 648 | 81 | hsa-miR-20a-5p | [SCN2A](http://www.ncbi.nlm.nih.gov/entrez/query.fcgi?db=gene&cmd=Retrieve&dopt=full_report&list_uids=6326) | sodium voltage-gated channel alpha subunit 2 |
| [Details](https://mirdb.org/cgi-bin/target_detail.cgi?targetID=2854679) | 649 | 81 | hsa-miR-20a-5p | [KLF9](http://www.ncbi.nlm.nih.gov/entrez/query.fcgi?db=gene&cmd=Retrieve&dopt=full_report&list_uids=687) | Kruppel like factor 9 |
| [Details](https://mirdb.org/cgi-bin/target_detail.cgi?targetID=2854763) | 650 | 81 | hsa-miR-20a-5p | [LZIC](http://www.ncbi.nlm.nih.gov/entrez/query.fcgi?db=gene&cmd=Retrieve&dopt=full_report&list_uids=84328) | leucine zipper and CTNNBIP1 domain containing |
| [Details](https://mirdb.org/cgi-bin/target_detail.cgi?targetID=2854785) | 651 | 81 | hsa-miR-20a-5p | [SLC4A4](http://www.ncbi.nlm.nih.gov/entrez/query.fcgi?db=gene&cmd=Retrieve&dopt=full_report&list_uids=8671) | solute carrier family 4 member 4 |
| [Details](https://mirdb.org/cgi-bin/target_detail.cgi?targetID=2854830) | 652 | 81 | hsa-miR-20a-5p | [PHLPP2](http://www.ncbi.nlm.nih.gov/entrez/query.fcgi?db=gene&cmd=Retrieve&dopt=full_report&list_uids=23035) | PH domain and leucine rich repeat protein phosphatase 2 |
| [Details](https://mirdb.org/cgi-bin/target_detail.cgi?targetID=2854929) | 653 | 81 | hsa-miR-20a-5p | [RPS6KA6](http://www.ncbi.nlm.nih.gov/entrez/query.fcgi?db=gene&cmd=Retrieve&dopt=full_report&list_uids=27330) | ribosomal protein S6 kinase A6 |
| [Details](https://mirdb.org/cgi-bin/target_detail.cgi?targetID=2855079) | 654 | 81 | hsa-miR-20a-5p | [TAOK1](http://www.ncbi.nlm.nih.gov/entrez/query.fcgi?db=gene&cmd=Retrieve&dopt=full_report&list_uids=57551) | TAO kinase 1 |
| [Details](https://mirdb.org/cgi-bin/target_detail.cgi?targetID=2855158) | 655 | 81 | hsa-miR-20a-5p | [RAB30](http://www.ncbi.nlm.nih.gov/entrez/query.fcgi?db=gene&cmd=Retrieve&dopt=full_report&list_uids=27314) | RAB30, member RAS oncogene family |
| [Details](https://mirdb.org/cgi-bin/target_detail.cgi?targetID=2855197) | 656 | 81 | hsa-miR-20a-5p | [NDEL1](http://www.ncbi.nlm.nih.gov/entrez/query.fcgi?db=gene&cmd=Retrieve&dopt=full_report&list_uids=81565) | nudE neurodevelopment protein 1 like 1 |
| [Details](https://mirdb.org/cgi-bin/target_detail.cgi?targetID=2855287) | 657 | 81 | hsa-miR-20a-5p | [NPAS3](http://www.ncbi.nlm.nih.gov/entrez/query.fcgi?db=gene&cmd=Retrieve&dopt=full_report&list_uids=64067) | neuronal PAS domain protein 3 |
| [Details](https://mirdb.org/cgi-bin/target_detail.cgi?targetID=2855305) | 658 | 81 | hsa-miR-20a-5p | [GNPDA2](http://www.ncbi.nlm.nih.gov/entrez/query.fcgi?db=gene&cmd=Retrieve&dopt=full_report&list_uids=132789) | glucosamine-6-phosphate deaminase 2 |
| [Details](https://mirdb.org/cgi-bin/target_detail.cgi?targetID=2855378) | 659 | 81 | hsa-miR-20a-5p | [CPEB3](http://www.ncbi.nlm.nih.gov/entrez/query.fcgi?db=gene&cmd=Retrieve&dopt=full_report&list_uids=22849) | cytoplasmic polyadenylation element binding protein 3 |
| [Details](https://mirdb.org/cgi-bin/target_detail.cgi?targetID=2855433) | 660 | 81 | hsa-miR-20a-5p | [TMEM138](http://www.ncbi.nlm.nih.gov/entrez/query.fcgi?db=gene&cmd=Retrieve&dopt=full_report&list_uids=51524) | transmembrane protein 138 |
| [Details](https://mirdb.org/cgi-bin/target_detail.cgi?targetID=2854128) | 661 | 80 | hsa-miR-20a-5p | [FAM3C](http://www.ncbi.nlm.nih.gov/entrez/query.fcgi?db=gene&cmd=Retrieve&dopt=full_report&list_uids=10447) | family with sequence similarity 3 member C |
| [Details](https://mirdb.org/cgi-bin/target_detail.cgi?targetID=2854164) | 662 | 80 | hsa-miR-20a-5p | [RNF217](http://www.ncbi.nlm.nih.gov/entrez/query.fcgi?db=gene&cmd=Retrieve&dopt=full_report&list_uids=154214) | ring finger protein 217 |
| [Details](https://mirdb.org/cgi-bin/target_detail.cgi?targetID=2854170) | 663 | 80 | hsa-miR-20a-5p | [STYX](http://www.ncbi.nlm.nih.gov/entrez/query.fcgi?db=gene&cmd=Retrieve&dopt=full_report&list_uids=6815) | serine/threonine/tyrosine interacting protein |
| [Details](https://mirdb.org/cgi-bin/target_detail.cgi?targetID=2854208) | 664 | 80 | hsa-miR-20a-5p | [ATXN7L1](http://www.ncbi.nlm.nih.gov/entrez/query.fcgi?db=gene&cmd=Retrieve&dopt=full_report&list_uids=222255) | ataxin 7 like 1 |
| [Details](https://mirdb.org/cgi-bin/target_detail.cgi?targetID=2854249) | 665 | 80 | hsa-miR-20a-5p | [KPNA3](http://www.ncbi.nlm.nih.gov/entrez/query.fcgi?db=gene&cmd=Retrieve&dopt=full_report&list_uids=3839) | karyopherin subunit alpha 3 |
| [Details](https://mirdb.org/cgi-bin/target_detail.cgi?targetID=2854336) | 666 | 80 | hsa-miR-20a-5p | [BTG2](http://www.ncbi.nlm.nih.gov/entrez/query.fcgi?db=gene&cmd=Retrieve&dopt=full_report&list_uids=7832) | BTG anti-proliferation factor 2 |
| [Details](https://mirdb.org/cgi-bin/target_detail.cgi?targetID=2854346) | 667 | 80 | hsa-miR-20a-5p | [SS18L1](http://www.ncbi.nlm.nih.gov/entrez/query.fcgi?db=gene&cmd=Retrieve&dopt=full_report&list_uids=26039) | SS18L1, nBAF chromatin remodeling complex subunit |
| [Details](https://mirdb.org/cgi-bin/target_detail.cgi?targetID=2854388) | 668 | 80 | hsa-miR-20a-5p | [JAK1](http://www.ncbi.nlm.nih.gov/entrez/query.fcgi?db=gene&cmd=Retrieve&dopt=full_report&list_uids=3716) | Janus kinase 1 |
| [Details](https://mirdb.org/cgi-bin/target_detail.cgi?targetID=2854422) | 669 | 80 | hsa-miR-20a-5p | [KMT5B](http://www.ncbi.nlm.nih.gov/entrez/query.fcgi?db=gene&cmd=Retrieve&dopt=full_report&list_uids=51111) | lysine methyltransferase 5B |
| [Details](https://mirdb.org/cgi-bin/target_detail.cgi?targetID=2854454) | 670 | 80 | hsa-miR-20a-5p | [ATG14](http://www.ncbi.nlm.nih.gov/entrez/query.fcgi?db=gene&cmd=Retrieve&dopt=full_report&list_uids=22863) | autophagy related 14 |
| [Details](https://mirdb.org/cgi-bin/target_detail.cgi?targetID=2854501) | 671 | 80 | hsa-miR-20a-5p | [CCSAP](http://www.ncbi.nlm.nih.gov/entrez/query.fcgi?db=gene&cmd=Retrieve&dopt=full_report&list_uids=126731) | centriole, cilia and spindle associated protein |
| [Details](https://mirdb.org/cgi-bin/target_detail.cgi?targetID=2854559) | 672 | 80 | hsa-miR-20a-5p | [SLC41A1](http://www.ncbi.nlm.nih.gov/entrez/query.fcgi?db=gene&cmd=Retrieve&dopt=full_report&list_uids=254428) | solute carrier family 41 member 1 |
| [Details](https://mirdb.org/cgi-bin/target_detail.cgi?targetID=2854625) | 673 | 80 | hsa-miR-20a-5p | [CRK](http://www.ncbi.nlm.nih.gov/entrez/query.fcgi?db=gene&cmd=Retrieve&dopt=full_report&list_uids=1398) | CRK proto-oncogene, adaptor protein |
| [Details](https://mirdb.org/cgi-bin/target_detail.cgi?targetID=2854682) | 674 | 80 | hsa-miR-20a-5p | [PPARA](http://www.ncbi.nlm.nih.gov/entrez/query.fcgi?db=gene&cmd=Retrieve&dopt=full_report&list_uids=5465) | peroxisome proliferator activated receptor alpha |
| [Details](https://mirdb.org/cgi-bin/target_detail.cgi?targetID=2854689) | 675 | 80 | hsa-miR-20a-5p | [MDM4](http://www.ncbi.nlm.nih.gov/entrez/query.fcgi?db=gene&cmd=Retrieve&dopt=full_report&list_uids=4194) | MDM4, p53 regulator |
| [Details](https://mirdb.org/cgi-bin/target_detail.cgi?targetID=2854749) | 676 | 80 | hsa-miR-20a-5p | [TUSC2](http://www.ncbi.nlm.nih.gov/entrez/query.fcgi?db=gene&cmd=Retrieve&dopt=full_report&list_uids=11334) | tumor suppressor 2, mitochondrial calcium regulator |
| [Details](https://mirdb.org/cgi-bin/target_detail.cgi?targetID=2854782) | 677 | 80 | hsa-miR-20a-5p | [SLC45A4](http://www.ncbi.nlm.nih.gov/entrez/query.fcgi?db=gene&cmd=Retrieve&dopt=full_report&list_uids=57210) | solute carrier family 45 member 4 |
| [Details](https://mirdb.org/cgi-bin/target_detail.cgi?targetID=2854837) | 678 | 80 | hsa-miR-20a-5p | [ATP1A2](http://www.ncbi.nlm.nih.gov/entrez/query.fcgi?db=gene&cmd=Retrieve&dopt=full_report&list_uids=477) | ATPase Na+/K+ transporting subunit alpha 2 |
| [Details](https://mirdb.org/cgi-bin/target_detail.cgi?targetID=2854923) | 679 | 80 | hsa-miR-20a-5p | [ITCH](http://www.ncbi.nlm.nih.gov/entrez/query.fcgi?db=gene&cmd=Retrieve&dopt=full_report&list_uids=83737) | itchy E3 ubiquitin protein ligase |
| [Details](https://mirdb.org/cgi-bin/target_detail.cgi?targetID=2854944) | 680 | 80 | hsa-miR-20a-5p | [MTURN](http://www.ncbi.nlm.nih.gov/entrez/query.fcgi?db=gene&cmd=Retrieve&dopt=full_report&list_uids=222166) | maturin, neural progenitor differentiation regulator homolog |
| [Details](https://mirdb.org/cgi-bin/target_detail.cgi?targetID=2854997) | 681 | 80 | hsa-miR-20a-5p | [AKT3](http://www.ncbi.nlm.nih.gov/entrez/query.fcgi?db=gene&cmd=Retrieve&dopt=full_report&list_uids=10000) | AKT serine/threonine kinase 3 |
| [Details](https://mirdb.org/cgi-bin/target_detail.cgi?targetID=2855067) | 682 | 80 | hsa-miR-20a-5p | [FAS](http://www.ncbi.nlm.nih.gov/entrez/query.fcgi?db=gene&cmd=Retrieve&dopt=full_report&list_uids=355) | Fas cell surface death receptor |
| [Details](https://mirdb.org/cgi-bin/target_detail.cgi?targetID=2855104) | 683 | 80 | hsa-miR-20a-5p | [FGL2](http://www.ncbi.nlm.nih.gov/entrez/query.fcgi?db=gene&cmd=Retrieve&dopt=full_report&list_uids=10875) | fibrinogen like 2 |
| [Details](https://mirdb.org/cgi-bin/target_detail.cgi?targetID=2855174) | 684 | 80 | hsa-miR-20a-5p | [KRT10](http://www.ncbi.nlm.nih.gov/entrez/query.fcgi?db=gene&cmd=Retrieve&dopt=full_report&list_uids=3858) | keratin 10 |
| [Details](https://mirdb.org/cgi-bin/target_detail.cgi?targetID=2855302) | 685 | 80 | hsa-miR-20a-5p | [KIAA1522](http://www.ncbi.nlm.nih.gov/entrez/query.fcgi?db=gene&cmd=Retrieve&dopt=full_report&list_uids=57648) | KIAA1522 |
| [Details](https://mirdb.org/cgi-bin/target_detail.cgi?targetID=2854123) | 686 | 79 | hsa-miR-20a-5p | [ST8SIA2](http://www.ncbi.nlm.nih.gov/entrez/query.fcgi?db=gene&cmd=Retrieve&dopt=full_report&list_uids=8128) | ST8 alpha-N-acetyl-neuraminide alpha-2,8-sialyltransferase 2 |
| [Details](https://mirdb.org/cgi-bin/target_detail.cgi?targetID=2854141) | 687 | 79 | hsa-miR-20a-5p | [HOOK3](http://www.ncbi.nlm.nih.gov/entrez/query.fcgi?db=gene&cmd=Retrieve&dopt=full_report&list_uids=84376) | hook microtubule tethering protein 3 |
| [Details](https://mirdb.org/cgi-bin/target_detail.cgi?targetID=2854157) | 688 | 79 | hsa-miR-20a-5p | [ESR1](http://www.ncbi.nlm.nih.gov/entrez/query.fcgi?db=gene&cmd=Retrieve&dopt=full_report&list_uids=2099) | estrogen receptor 1 |
| [Details](https://mirdb.org/cgi-bin/target_detail.cgi?targetID=2854165) | 689 | 79 | hsa-miR-20a-5p | [TGFB1I1](http://www.ncbi.nlm.nih.gov/entrez/query.fcgi?db=gene&cmd=Retrieve&dopt=full_report&list_uids=7041) | transforming growth factor beta 1 induced transcript 1 |
| [Details](https://mirdb.org/cgi-bin/target_detail.cgi?targetID=2854187) | 690 | 79 | hsa-miR-20a-5p | [SPRY4](http://www.ncbi.nlm.nih.gov/entrez/query.fcgi?db=gene&cmd=Retrieve&dopt=full_report&list_uids=81848) | sprouty RTK signaling antagonist 4 |
| [Details](https://mirdb.org/cgi-bin/target_detail.cgi?targetID=2854197) | 691 | 79 | hsa-miR-20a-5p | [TWF1](http://www.ncbi.nlm.nih.gov/entrez/query.fcgi?db=gene&cmd=Retrieve&dopt=full_report&list_uids=5756) | twinfilin actin binding protein 1 |
| [Details](https://mirdb.org/cgi-bin/target_detail.cgi?targetID=2854211) | 692 | 79 | hsa-miR-20a-5p | [SH3GLB1](http://www.ncbi.nlm.nih.gov/entrez/query.fcgi?db=gene&cmd=Retrieve&dopt=full_report&list_uids=51100) | SH3 domain containing GRB2 like, endophilin B1 |
| [Details](https://mirdb.org/cgi-bin/target_detail.cgi?targetID=2854246) | 693 | 79 | hsa-miR-20a-5p | [ATXN1](http://www.ncbi.nlm.nih.gov/entrez/query.fcgi?db=gene&cmd=Retrieve&dopt=full_report&list_uids=6310) | ataxin 1 |
| [Details](https://mirdb.org/cgi-bin/target_detail.cgi?targetID=2854267) | 694 | 79 | hsa-miR-20a-5p | [CAMK2N2](http://www.ncbi.nlm.nih.gov/entrez/query.fcgi?db=gene&cmd=Retrieve&dopt=full_report&list_uids=94032) | calcium/calmodulin dependent protein kinase II inhibitor 2 |
| [Details](https://mirdb.org/cgi-bin/target_detail.cgi?targetID=2854279) | 695 | 79 | hsa-miR-20a-5p | [EGLN1](http://www.ncbi.nlm.nih.gov/entrez/query.fcgi?db=gene&cmd=Retrieve&dopt=full_report&list_uids=54583) | egl-9 family hypoxia inducible factor 1 |
| [Details](https://mirdb.org/cgi-bin/target_detail.cgi?targetID=2854598) | 696 | 79 | hsa-miR-20a-5p | [CXCL14](http://www.ncbi.nlm.nih.gov/entrez/query.fcgi?db=gene&cmd=Retrieve&dopt=full_report&list_uids=9547) | C-X-C motif chemokine ligand 14 |
| [Details](https://mirdb.org/cgi-bin/target_detail.cgi?targetID=2854752) | 697 | 79 | hsa-miR-20a-5p | [UTP14C](http://www.ncbi.nlm.nih.gov/entrez/query.fcgi?db=gene&cmd=Retrieve&dopt=full_report&list_uids=9724) | UTP14C, small subunit processome component |
| [Details](https://mirdb.org/cgi-bin/target_detail.cgi?targetID=2854825) | 698 | 79 | hsa-miR-20a-5p | [APBB2](http://www.ncbi.nlm.nih.gov/entrez/query.fcgi?db=gene&cmd=Retrieve&dopt=full_report&list_uids=323) | amyloid beta precursor protein binding family B member 2 |
| [Details](https://mirdb.org/cgi-bin/target_detail.cgi?targetID=2854835) | 699 | 79 | hsa-miR-20a-5p | [ZNF362](http://www.ncbi.nlm.nih.gov/entrez/query.fcgi?db=gene&cmd=Retrieve&dopt=full_report&list_uids=149076) | zinc finger protein 362 |
| [Details](https://mirdb.org/cgi-bin/target_detail.cgi?targetID=2854867) | 700 | 79 | hsa-miR-20a-5p | [C16orf70](http://www.ncbi.nlm.nih.gov/entrez/query.fcgi?db=gene&cmd=Retrieve&dopt=full_report&list_uids=80262) | chromosome 16 open reading frame 70 |
| [Details](https://mirdb.org/cgi-bin/target_detail.cgi?targetID=2855011) | 701 | 79 | hsa-miR-20a-5p | [UBE2B](http://www.ncbi.nlm.nih.gov/entrez/query.fcgi?db=gene&cmd=Retrieve&dopt=full_report&list_uids=7320) | ubiquitin conjugating enzyme E2 B |
| [Details](https://mirdb.org/cgi-bin/target_detail.cgi?targetID=2855046) | 702 | 79 | hsa-miR-20a-5p | [SGTB](http://www.ncbi.nlm.nih.gov/entrez/query.fcgi?db=gene&cmd=Retrieve&dopt=full_report&list_uids=54557) | small glutamine rich tetratricopeptide repeat containing beta |
| [Details](https://mirdb.org/cgi-bin/target_detail.cgi?targetID=2855190) | 703 | 79 | hsa-miR-20a-5p | [COX7A2L](http://www.ncbi.nlm.nih.gov/entrez/query.fcgi?db=gene&cmd=Retrieve&dopt=full_report&list_uids=9167) | cytochrome c oxidase subunit 7A2 like |
| [Details](https://mirdb.org/cgi-bin/target_detail.cgi?targetID=2855246) | 704 | 79 | hsa-miR-20a-5p | [MYO1D](http://www.ncbi.nlm.nih.gov/entrez/query.fcgi?db=gene&cmd=Retrieve&dopt=full_report&list_uids=4642) | myosin ID |
| [Details](https://mirdb.org/cgi-bin/target_detail.cgi?targetID=2855252) | 705 | 79 | hsa-miR-20a-5p | [FEM1B](http://www.ncbi.nlm.nih.gov/entrez/query.fcgi?db=gene&cmd=Retrieve&dopt=full_report&list_uids=10116) | fem-1 homolog B |
| [Details](https://mirdb.org/cgi-bin/target_detail.cgi?targetID=2854139) | 706 | 78 | hsa-miR-20a-5p | [TBC1D15](http://www.ncbi.nlm.nih.gov/entrez/query.fcgi?db=gene&cmd=Retrieve&dopt=full_report&list_uids=64786) | TBC1 domain family member 15 |
| [Details](https://mirdb.org/cgi-bin/target_detail.cgi?targetID=2854268) | 707 | 78 | hsa-miR-20a-5p | [LONP2](http://www.ncbi.nlm.nih.gov/entrez/query.fcgi?db=gene&cmd=Retrieve&dopt=full_report&list_uids=83752) | lon peptidase 2, peroxisomal |
| [Details](https://mirdb.org/cgi-bin/target_detail.cgi?targetID=2854284) | 708 | 78 | hsa-miR-20a-5p | [ERI1](http://www.ncbi.nlm.nih.gov/entrez/query.fcgi?db=gene&cmd=Retrieve&dopt=full_report&list_uids=90459) | exoribonuclease 1 |
| [Details](https://mirdb.org/cgi-bin/target_detail.cgi?targetID=2854291) | 709 | 78 | hsa-miR-20a-5p | [TMBIM6](http://www.ncbi.nlm.nih.gov/entrez/query.fcgi?db=gene&cmd=Retrieve&dopt=full_report&list_uids=7009) | transmembrane BAX inhibitor motif containing 6 |
| [Details](https://mirdb.org/cgi-bin/target_detail.cgi?targetID=2854354) | 710 | 78 | hsa-miR-20a-5p | [FAM117A](http://www.ncbi.nlm.nih.gov/entrez/query.fcgi?db=gene&cmd=Retrieve&dopt=full_report&list_uids=81558) | family with sequence similarity 117 member A |
| [Details](https://mirdb.org/cgi-bin/target_detail.cgi?targetID=2854382) | 711 | 78 | hsa-miR-20a-5p | [KDM2A](http://www.ncbi.nlm.nih.gov/entrez/query.fcgi?db=gene&cmd=Retrieve&dopt=full_report&list_uids=22992) | lysine demethylase 2A |
| [Details](https://mirdb.org/cgi-bin/target_detail.cgi?targetID=2854396) | 712 | 78 | hsa-miR-20a-5p | [CLEC12B](http://www.ncbi.nlm.nih.gov/entrez/query.fcgi?db=gene&cmd=Retrieve&dopt=full_report&list_uids=387837) | C-type lectin domain family 12 member B |
| [Details](https://mirdb.org/cgi-bin/target_detail.cgi?targetID=2854410) | 713 | 78 | hsa-miR-20a-5p | [PAF1](http://www.ncbi.nlm.nih.gov/entrez/query.fcgi?db=gene&cmd=Retrieve&dopt=full_report&list_uids=54623) | PAF1 homolog, Paf1/RNA polymerase II complex component |
| [Details](https://mirdb.org/cgi-bin/target_detail.cgi?targetID=2854421) | 714 | 78 | hsa-miR-20a-5p | [GBF1](http://www.ncbi.nlm.nih.gov/entrez/query.fcgi?db=gene&cmd=Retrieve&dopt=full_report&list_uids=8729) | golgi brefeldin A resistant guanine nucleotide exchange factor 1 |
| [Details](https://mirdb.org/cgi-bin/target_detail.cgi?targetID=2854445) | 715 | 78 | hsa-miR-20a-5p | [RBSN](http://www.ncbi.nlm.nih.gov/entrez/query.fcgi?db=gene&cmd=Retrieve&dopt=full_report&list_uids=64145) | rabenosyn, RAB effector |
| [Details](https://mirdb.org/cgi-bin/target_detail.cgi?targetID=2854474) | 716 | 78 | hsa-miR-20a-5p | [SMIM14](http://www.ncbi.nlm.nih.gov/entrez/query.fcgi?db=gene&cmd=Retrieve&dopt=full_report&list_uids=201895) | small integral membrane protein 14 |
| [Details](https://mirdb.org/cgi-bin/target_detail.cgi?targetID=2854602) | 717 | 78 | hsa-miR-20a-5p | [CIT](http://www.ncbi.nlm.nih.gov/entrez/query.fcgi?db=gene&cmd=Retrieve&dopt=full_report&list_uids=11113) | citron rho-interacting serine/threonine kinase |
| [Details](https://mirdb.org/cgi-bin/target_detail.cgi?targetID=2854622) | 718 | 78 | hsa-miR-20a-5p | [TMEM267](http://www.ncbi.nlm.nih.gov/entrez/query.fcgi?db=gene&cmd=Retrieve&dopt=full_report&list_uids=64417) | transmembrane protein 267 |
| [Details](https://mirdb.org/cgi-bin/target_detail.cgi?targetID=2854632) | 719 | 78 | hsa-miR-20a-5p | [EEA1](http://www.ncbi.nlm.nih.gov/entrez/query.fcgi?db=gene&cmd=Retrieve&dopt=full_report&list_uids=8411) | early endosome antigen 1 |
| [Details](https://mirdb.org/cgi-bin/target_detail.cgi?targetID=2854734) | 720 | 78 | hsa-miR-20a-5p | [PTGFRN](http://www.ncbi.nlm.nih.gov/entrez/query.fcgi?db=gene&cmd=Retrieve&dopt=full_report&list_uids=5738) | prostaglandin F2 receptor inhibitor |
| [Details](https://mirdb.org/cgi-bin/target_detail.cgi?targetID=2854737) | 721 | 78 | hsa-miR-20a-5p | [CHAF1A](http://www.ncbi.nlm.nih.gov/entrez/query.fcgi?db=gene&cmd=Retrieve&dopt=full_report&list_uids=10036) | chromatin assembly factor 1 subunit A |
| [Details](https://mirdb.org/cgi-bin/target_detail.cgi?targetID=2854754) | 722 | 78 | hsa-miR-20a-5p | [DOK6](http://www.ncbi.nlm.nih.gov/entrez/query.fcgi?db=gene&cmd=Retrieve&dopt=full_report&list_uids=220164) | docking protein 6 |
| [Details](https://mirdb.org/cgi-bin/target_detail.cgi?targetID=2854764) | 723 | 78 | hsa-miR-20a-5p | [RNF13](http://www.ncbi.nlm.nih.gov/entrez/query.fcgi?db=gene&cmd=Retrieve&dopt=full_report&list_uids=11342) | ring finger protein 13 |
| [Details](https://mirdb.org/cgi-bin/target_detail.cgi?targetID=2855012) | 724 | 78 | hsa-miR-20a-5p | [PAFAH1B2](http://www.ncbi.nlm.nih.gov/entrez/query.fcgi?db=gene&cmd=Retrieve&dopt=full_report&list_uids=5049) | platelet activating factor acetylhydrolase 1b catalytic subunit 2 |
| [Details](https://mirdb.org/cgi-bin/target_detail.cgi?targetID=2855025) | 725 | 78 | hsa-miR-20a-5p | [AKAP5](http://www.ncbi.nlm.nih.gov/entrez/query.fcgi?db=gene&cmd=Retrieve&dopt=full_report&list_uids=9495) | A-kinase anchoring protein 5 |
| [Details](https://mirdb.org/cgi-bin/target_detail.cgi?targetID=2855038) | 726 | 78 | hsa-miR-20a-5p | [PLEKHO2](http://www.ncbi.nlm.nih.gov/entrez/query.fcgi?db=gene&cmd=Retrieve&dopt=full_report&list_uids=80301) | pleckstrin homology domain containing O2 |
| [Details](https://mirdb.org/cgi-bin/target_detail.cgi?targetID=2855136) | 727 | 78 | hsa-miR-20a-5p | [DSG4](http://www.ncbi.nlm.nih.gov/entrez/query.fcgi?db=gene&cmd=Retrieve&dopt=full_report&list_uids=147409) | desmoglein 4 |
| [Details](https://mirdb.org/cgi-bin/target_detail.cgi?targetID=2855173) | 728 | 78 | hsa-miR-20a-5p | [CELSR2](http://www.ncbi.nlm.nih.gov/entrez/query.fcgi?db=gene&cmd=Retrieve&dopt=full_report&list_uids=1952) | cadherin EGF LAG seven-pass G-type receptor 2 |
| [Details](https://mirdb.org/cgi-bin/target_detail.cgi?targetID=2855193) | 729 | 78 | hsa-miR-20a-5p | [ZFYVE21](http://www.ncbi.nlm.nih.gov/entrez/query.fcgi?db=gene&cmd=Retrieve&dopt=full_report&list_uids=79038) | zinc finger FYVE-type containing 21 |
| [Details](https://mirdb.org/cgi-bin/target_detail.cgi?targetID=2855208) | 730 | 78 | hsa-miR-20a-5p | [M6PR](http://www.ncbi.nlm.nih.gov/entrez/query.fcgi?db=gene&cmd=Retrieve&dopt=full_report&list_uids=4074) | mannose-6-phosphate receptor, cation dependent |
| [Details](https://mirdb.org/cgi-bin/target_detail.cgi?targetID=2855261) | 731 | 78 | hsa-miR-20a-5p | [LGI2](http://www.ncbi.nlm.nih.gov/entrez/query.fcgi?db=gene&cmd=Retrieve&dopt=full_report&list_uids=55203) | leucine rich repeat LGI family member 2 |
| [Details](https://mirdb.org/cgi-bin/target_detail.cgi?targetID=2855263) | 732 | 78 | hsa-miR-20a-5p | [LAMP3](http://www.ncbi.nlm.nih.gov/entrez/query.fcgi?db=gene&cmd=Retrieve&dopt=full_report&list_uids=27074) | lysosomal associated membrane protein 3 |
| [Details](https://mirdb.org/cgi-bin/target_detail.cgi?targetID=2855346) | 733 | 78 | hsa-miR-20a-5p | [NEURL1B](http://www.ncbi.nlm.nih.gov/entrez/query.fcgi?db=gene&cmd=Retrieve&dopt=full_report&list_uids=54492) | neuralized E3 ubiquitin protein ligase 1B |
| [Details](https://mirdb.org/cgi-bin/target_detail.cgi?targetID=2855360) | 734 | 78 | hsa-miR-20a-5p | [AMIGO2](http://www.ncbi.nlm.nih.gov/entrez/query.fcgi?db=gene&cmd=Retrieve&dopt=full_report&list_uids=347902) | adhesion molecule with Ig like domain 2 |
| [Details](https://mirdb.org/cgi-bin/target_detail.cgi?targetID=2855413) | 735 | 78 | hsa-miR-20a-5p | [MYO5B](http://www.ncbi.nlm.nih.gov/entrez/query.fcgi?db=gene&cmd=Retrieve&dopt=full_report&list_uids=4645) | myosin VB |
| [Details](https://mirdb.org/cgi-bin/target_detail.cgi?targetID=2855451) | 736 | 78 | hsa-miR-20a-5p | [UTP23](http://www.ncbi.nlm.nih.gov/entrez/query.fcgi?db=gene&cmd=Retrieve&dopt=full_report&list_uids=84294) | UTP23, small subunit processome component |
| [Details](https://mirdb.org/cgi-bin/target_detail.cgi?targetID=2854125) | 737 | 77 | hsa-miR-20a-5p | [ACBD5](http://www.ncbi.nlm.nih.gov/entrez/query.fcgi?db=gene&cmd=Retrieve&dopt=full_report&list_uids=91452) | acyl-CoA binding domain containing 5 |
| [Details](https://mirdb.org/cgi-bin/target_detail.cgi?targetID=2854329) | 738 | 77 | hsa-miR-20a-5p | [SPATA1](http://www.ncbi.nlm.nih.gov/entrez/query.fcgi?db=gene&cmd=Retrieve&dopt=full_report&list_uids=100505741) | spermatogenesis associated 1 |
| [Details](https://mirdb.org/cgi-bin/target_detail.cgi?targetID=2854352) | 739 | 77 | hsa-miR-20a-5p | [ADAT2](http://www.ncbi.nlm.nih.gov/entrez/query.fcgi?db=gene&cmd=Retrieve&dopt=full_report&list_uids=134637) | adenosine deaminase, tRNA specific 2 |
| [Details](https://mirdb.org/cgi-bin/target_detail.cgi?targetID=2854393) | 740 | 77 | hsa-miR-20a-5p | [TMCC3](http://www.ncbi.nlm.nih.gov/entrez/query.fcgi?db=gene&cmd=Retrieve&dopt=full_report&list_uids=57458) | transmembrane and coiled-coil domain family 3 |
| [Details](https://mirdb.org/cgi-bin/target_detail.cgi?targetID=2854539) | 741 | 77 | hsa-miR-20a-5p | [C9orf40](http://www.ncbi.nlm.nih.gov/entrez/query.fcgi?db=gene&cmd=Retrieve&dopt=full_report&list_uids=55071) | chromosome 9 open reading frame 40 |
| [Details](https://mirdb.org/cgi-bin/target_detail.cgi?targetID=2854627) | 742 | 77 | hsa-miR-20a-5p | [E2F2](http://www.ncbi.nlm.nih.gov/entrez/query.fcgi?db=gene&cmd=Retrieve&dopt=full_report&list_uids=1870) | E2F transcription factor 2 |
| [Details](https://mirdb.org/cgi-bin/target_detail.cgi?targetID=2854642) | 743 | 77 | hsa-miR-20a-5p | [PHTF2](http://www.ncbi.nlm.nih.gov/entrez/query.fcgi?db=gene&cmd=Retrieve&dopt=full_report&list_uids=57157) | putative homeodomain transcription factor 2 |
| [Details](https://mirdb.org/cgi-bin/target_detail.cgi?targetID=2854659) | 744 | 77 | hsa-miR-20a-5p | [RB1](http://www.ncbi.nlm.nih.gov/entrez/query.fcgi?db=gene&cmd=Retrieve&dopt=full_report&list_uids=5925) | RB transcriptional corepressor 1 |
| [Details](https://mirdb.org/cgi-bin/target_detail.cgi?targetID=2854660) | 745 | 77 | hsa-miR-20a-5p | [C3orf38](http://www.ncbi.nlm.nih.gov/entrez/query.fcgi?db=gene&cmd=Retrieve&dopt=full_report&list_uids=285237) | chromosome 3 open reading frame 38 |
| [Details](https://mirdb.org/cgi-bin/target_detail.cgi?targetID=2854773) | 746 | 77 | hsa-miR-20a-5p | [TENT5C](http://www.ncbi.nlm.nih.gov/entrez/query.fcgi?db=gene&cmd=Retrieve&dopt=full_report&list_uids=54855) | terminal nucleotidyltransferase 5C |
| [Details](https://mirdb.org/cgi-bin/target_detail.cgi?targetID=2854849) | 747 | 77 | hsa-miR-20a-5p | [UBE2J1](http://www.ncbi.nlm.nih.gov/entrez/query.fcgi?db=gene&cmd=Retrieve&dopt=full_report&list_uids=51465) | ubiquitin conjugating enzyme E2 J1 |
| [Details](https://mirdb.org/cgi-bin/target_detail.cgi?targetID=2854933) | 748 | 77 | hsa-miR-20a-5p | [ZNF805](http://www.ncbi.nlm.nih.gov/entrez/query.fcgi?db=gene&cmd=Retrieve&dopt=full_report&list_uids=390980) | zinc finger protein 805 |
| [Details](https://mirdb.org/cgi-bin/target_detail.cgi?targetID=2854947) | 749 | 77 | hsa-miR-20a-5p | [CRIM1](http://www.ncbi.nlm.nih.gov/entrez/query.fcgi?db=gene&cmd=Retrieve&dopt=full_report&list_uids=51232) | cysteine rich transmembrane BMP regulator 1 |
| [Details](https://mirdb.org/cgi-bin/target_detail.cgi?targetID=2854978) | 750 | 77 | hsa-miR-20a-5p | [PTH](http://www.ncbi.nlm.nih.gov/entrez/query.fcgi?db=gene&cmd=Retrieve&dopt=full_report&list_uids=5741) | parathyroid hormone |
| [Details](https://mirdb.org/cgi-bin/target_detail.cgi?targetID=2854979) | 751 | 77 | hsa-miR-20a-5p | [MAPK8](http://www.ncbi.nlm.nih.gov/entrez/query.fcgi?db=gene&cmd=Retrieve&dopt=full_report&list_uids=5599) | mitogen-activated protein kinase 8 |
| [Details](https://mirdb.org/cgi-bin/target_detail.cgi?targetID=2855377) | 752 | 77 | hsa-miR-20a-5p | [SUMF1](http://www.ncbi.nlm.nih.gov/entrez/query.fcgi?db=gene&cmd=Retrieve&dopt=full_report&list_uids=285362) | sulfatase modifying factor 1 |
| [Details](https://mirdb.org/cgi-bin/target_detail.cgi?targetID=2855406) | 753 | 77 | hsa-miR-20a-5p | [MAP3K5](http://www.ncbi.nlm.nih.gov/entrez/query.fcgi?db=gene&cmd=Retrieve&dopt=full_report&list_uids=4217) | mitogen-activated protein kinase kinase kinase 5 |
| [Details](https://mirdb.org/cgi-bin/target_detail.cgi?targetID=2855458) | 754 | 77 | hsa-miR-20a-5p | [TADA2B](http://www.ncbi.nlm.nih.gov/entrez/query.fcgi?db=gene&cmd=Retrieve&dopt=full_report&list_uids=93624) | transcriptional adaptor 2B |
| [Details](https://mirdb.org/cgi-bin/target_detail.cgi?targetID=2854114) | 755 | 76 | hsa-miR-20a-5p | [HMGA2](http://www.ncbi.nlm.nih.gov/entrez/query.fcgi?db=gene&cmd=Retrieve&dopt=full_report&list_uids=8091) | high mobility group AT-hook 2 |
| [Details](https://mirdb.org/cgi-bin/target_detail.cgi?targetID=2854225) | 756 | 76 | hsa-miR-20a-5p | [CRIPT](http://www.ncbi.nlm.nih.gov/entrez/query.fcgi?db=gene&cmd=Retrieve&dopt=full_report&list_uids=9419) | CXXC repeat containing interactor of PDZ3 domain |
| [Details](https://mirdb.org/cgi-bin/target_detail.cgi?targetID=2854232) | 757 | 76 | hsa-miR-20a-5p | [CREBRF](http://www.ncbi.nlm.nih.gov/entrez/query.fcgi?db=gene&cmd=Retrieve&dopt=full_report&list_uids=153222) | CREB3 regulatory factor |
| [Details](https://mirdb.org/cgi-bin/target_detail.cgi?targetID=2854351) | 758 | 76 | hsa-miR-20a-5p | [TMEM50B](http://www.ncbi.nlm.nih.gov/entrez/query.fcgi?db=gene&cmd=Retrieve&dopt=full_report&list_uids=757) | transmembrane protein 50B |
| [Details](https://mirdb.org/cgi-bin/target_detail.cgi?targetID=2854447) | 759 | 76 | hsa-miR-20a-5p | [PDRG1](http://www.ncbi.nlm.nih.gov/entrez/query.fcgi?db=gene&cmd=Retrieve&dopt=full_report&list_uids=81572) | p53 and DNA damage regulated 1 |
| [Details](https://mirdb.org/cgi-bin/target_detail.cgi?targetID=2854459) | 760 | 76 | hsa-miR-20a-5p | [NMUR2](http://www.ncbi.nlm.nih.gov/entrez/query.fcgi?db=gene&cmd=Retrieve&dopt=full_report&list_uids=56923) | neuromedin U receptor 2 |
| [Details](https://mirdb.org/cgi-bin/target_detail.cgi?targetID=2854467) | 761 | 76 | hsa-miR-20a-5p | [GIGYF1](http://www.ncbi.nlm.nih.gov/entrez/query.fcgi?db=gene&cmd=Retrieve&dopt=full_report&list_uids=64599) | GRB10 interacting GYF protein 1 |
| [Details](https://mirdb.org/cgi-bin/target_detail.cgi?targetID=2854482) | 762 | 76 | hsa-miR-20a-5p | [COL4A3](http://www.ncbi.nlm.nih.gov/entrez/query.fcgi?db=gene&cmd=Retrieve&dopt=full_report&list_uids=1285) | collagen type IV alpha 3 chain |
| [Details](https://mirdb.org/cgi-bin/target_detail.cgi?targetID=2854553) | 763 | 76 | hsa-miR-20a-5p | [MARCH6](http://www.ncbi.nlm.nih.gov/entrez/query.fcgi?db=gene&cmd=Retrieve&dopt=full_report&list_uids=10299) | membrane associated ring-CH-type finger 6 |
| [Details](https://mirdb.org/cgi-bin/target_detail.cgi?targetID=2854605) | 764 | 76 | hsa-miR-20a-5p | [CCSER2](http://www.ncbi.nlm.nih.gov/entrez/query.fcgi?db=gene&cmd=Retrieve&dopt=full_report&list_uids=54462) | coiled-coil serine rich protein 2 |
| [Details](https://mirdb.org/cgi-bin/target_detail.cgi?targetID=2854658) | 765 | 76 | hsa-miR-20a-5p | [STX6](http://www.ncbi.nlm.nih.gov/entrez/query.fcgi?db=gene&cmd=Retrieve&dopt=full_report&list_uids=10228) | syntaxin 6 |
| [Details](https://mirdb.org/cgi-bin/target_detail.cgi?targetID=2854699) | 766 | 76 | hsa-miR-20a-5p | [IPO9](http://www.ncbi.nlm.nih.gov/entrez/query.fcgi?db=gene&cmd=Retrieve&dopt=full_report&list_uids=55705) | importin 9 |
| [Details](https://mirdb.org/cgi-bin/target_detail.cgi?targetID=2854787) | 767 | 76 | hsa-miR-20a-5p | [SHTN1](http://www.ncbi.nlm.nih.gov/entrez/query.fcgi?db=gene&cmd=Retrieve&dopt=full_report&list_uids=57698) | shootin 1 |
| [Details](https://mirdb.org/cgi-bin/target_detail.cgi?targetID=2854910) | 768 | 76 | hsa-miR-20a-5p | [PURA](http://www.ncbi.nlm.nih.gov/entrez/query.fcgi?db=gene&cmd=Retrieve&dopt=full_report&list_uids=5813) | purine rich element binding protein A |
| [Details](https://mirdb.org/cgi-bin/target_detail.cgi?targetID=2854918) | 769 | 76 | hsa-miR-20a-5p | [JAZF1](http://www.ncbi.nlm.nih.gov/entrez/query.fcgi?db=gene&cmd=Retrieve&dopt=full_report&list_uids=221895) | JAZF zinc finger 1 |
| [Details](https://mirdb.org/cgi-bin/target_detail.cgi?targetID=2854943) | 770 | 76 | hsa-miR-20a-5p | [KLHL20](http://www.ncbi.nlm.nih.gov/entrez/query.fcgi?db=gene&cmd=Retrieve&dopt=full_report&list_uids=27252) | kelch like family member 20 |
| [Details](https://mirdb.org/cgi-bin/target_detail.cgi?targetID=2854959) | 771 | 76 | hsa-miR-20a-5p | [RNF11](http://www.ncbi.nlm.nih.gov/entrez/query.fcgi?db=gene&cmd=Retrieve&dopt=full_report&list_uids=26994) | ring finger protein 11 |
| [Details](https://mirdb.org/cgi-bin/target_detail.cgi?targetID=2854975) | 772 | 76 | hsa-miR-20a-5p | [GGCX](http://www.ncbi.nlm.nih.gov/entrez/query.fcgi?db=gene&cmd=Retrieve&dopt=full_report&list_uids=2677) | gamma-glutamyl carboxylase |
| [Details](https://mirdb.org/cgi-bin/target_detail.cgi?targetID=2855068) | 773 | 76 | hsa-miR-20a-5p | [PGP](http://www.ncbi.nlm.nih.gov/entrez/query.fcgi?db=gene&cmd=Retrieve&dopt=full_report&list_uids=283871) | phosphoglycolate phosphatase |
| [Details](https://mirdb.org/cgi-bin/target_detail.cgi?targetID=2855072) | 774 | 76 | hsa-miR-20a-5p | [NRBP1](http://www.ncbi.nlm.nih.gov/entrez/query.fcgi?db=gene&cmd=Retrieve&dopt=full_report&list_uids=29959) | nuclear receptor binding protein 1 |
| [Details](https://mirdb.org/cgi-bin/target_detail.cgi?targetID=2855084) | 775 | 76 | hsa-miR-20a-5p | [ARL4C](http://www.ncbi.nlm.nih.gov/entrez/query.fcgi?db=gene&cmd=Retrieve&dopt=full_report&list_uids=10123) | ADP ribosylation factor like GTPase 4C |
| [Details](https://mirdb.org/cgi-bin/target_detail.cgi?targetID=2855103) | 776 | 76 | hsa-miR-20a-5p | [DMTF1](http://www.ncbi.nlm.nih.gov/entrez/query.fcgi?db=gene&cmd=Retrieve&dopt=full_report&list_uids=9988) | cyclin D binding myb like transcription factor 1 |
| [Details](https://mirdb.org/cgi-bin/target_detail.cgi?targetID=2855137) | 777 | 76 | hsa-miR-20a-5p | [CNTNAP3](http://www.ncbi.nlm.nih.gov/entrez/query.fcgi?db=gene&cmd=Retrieve&dopt=full_report&list_uids=79937) | contactin associated protein like 3 |
| [Details](https://mirdb.org/cgi-bin/target_detail.cgi?targetID=2855143) | 778 | 76 | hsa-miR-20a-5p | [BMT2](http://www.ncbi.nlm.nih.gov/entrez/query.fcgi?db=gene&cmd=Retrieve&dopt=full_report&list_uids=154743) | base methyltransferase of 25S rRNA 2 homolog |
| [Details](https://mirdb.org/cgi-bin/target_detail.cgi?targetID=2855180) | 779 | 76 | hsa-miR-20a-5p | [TTC39C](http://www.ncbi.nlm.nih.gov/entrez/query.fcgi?db=gene&cmd=Retrieve&dopt=full_report&list_uids=125488) | tetratricopeptide repeat domain 39C |
| [Details](https://mirdb.org/cgi-bin/target_detail.cgi?targetID=2855213) | 780 | 76 | hsa-miR-20a-5p | [PCMTD1](http://www.ncbi.nlm.nih.gov/entrez/query.fcgi?db=gene&cmd=Retrieve&dopt=full_report&list_uids=115294) | protein-L-isoaspartate (D-aspartate) O-methyltransferase domain containing 1 |
| [Details](https://mirdb.org/cgi-bin/target_detail.cgi?targetID=2855238) | 781 | 76 | hsa-miR-20a-5p | [PREX1](http://www.ncbi.nlm.nih.gov/entrez/query.fcgi?db=gene&cmd=Retrieve&dopt=full_report&list_uids=57580) | phosphatidylinositol-3,4,5-trisphosphate dependent Rac exchange factor 1 |
| [Details](https://mirdb.org/cgi-bin/target_detail.cgi?targetID=2855280) | 782 | 76 | hsa-miR-20a-5p | [PON2](http://www.ncbi.nlm.nih.gov/entrez/query.fcgi?db=gene&cmd=Retrieve&dopt=full_report&list_uids=5445) | paraoxonase 2 |
| [Details](https://mirdb.org/cgi-bin/target_detail.cgi?targetID=2855296) | 783 | 76 | hsa-miR-20a-5p | [WEE1](http://www.ncbi.nlm.nih.gov/entrez/query.fcgi?db=gene&cmd=Retrieve&dopt=full_report&list_uids=7465) | WEE1 G2 checkpoint kinase |
| [Details](https://mirdb.org/cgi-bin/target_detail.cgi?targetID=2855338) | 784 | 76 | hsa-miR-20a-5p | [B2M](http://www.ncbi.nlm.nih.gov/entrez/query.fcgi?db=gene&cmd=Retrieve&dopt=full_report&list_uids=567) | beta-2-microglobulin |
| [Details](https://mirdb.org/cgi-bin/target_detail.cgi?targetID=2855359) | 785 | 76 | hsa-miR-20a-5p | [PPP4R3B](http://www.ncbi.nlm.nih.gov/entrez/query.fcgi?db=gene&cmd=Retrieve&dopt=full_report&list_uids=57223) | protein phosphatase 4 regulatory subunit 3B |
| [Details](https://mirdb.org/cgi-bin/target_detail.cgi?targetID=2855401) | 786 | 76 | hsa-miR-20a-5p | [FRMD4B](http://www.ncbi.nlm.nih.gov/entrez/query.fcgi?db=gene&cmd=Retrieve&dopt=full_report&list_uids=23150) | FERM domain containing 4B |
| [Details](https://mirdb.org/cgi-bin/target_detail.cgi?targetID=2855404) | 787 | 76 | hsa-miR-20a-5p | [PPP6R2](http://www.ncbi.nlm.nih.gov/entrez/query.fcgi?db=gene&cmd=Retrieve&dopt=full_report&list_uids=9701) | protein phosphatase 6 regulatory subunit 2 |
| [Details](https://mirdb.org/cgi-bin/target_detail.cgi?targetID=2855425) | 788 | 76 | hsa-miR-20a-5p | [RABEP1](http://www.ncbi.nlm.nih.gov/entrez/query.fcgi?db=gene&cmd=Retrieve&dopt=full_report&list_uids=9135) | rabaptin, RAB GTPase binding effector protein 1 |
| [Details](https://mirdb.org/cgi-bin/target_detail.cgi?targetID=2855432) | 789 | 76 | hsa-miR-20a-5p | [SLC16A7](http://www.ncbi.nlm.nih.gov/entrez/query.fcgi?db=gene&cmd=Retrieve&dopt=full_report&list_uids=9194) | solute carrier family 16 member 7 |
| [Details](https://mirdb.org/cgi-bin/target_detail.cgi?targetID=2854148) | 790 | 75 | hsa-miR-20a-5p | [SH2D5](http://www.ncbi.nlm.nih.gov/entrez/query.fcgi?db=gene&cmd=Retrieve&dopt=full_report&list_uids=400745) | SH2 domain containing 5 |
| [Details](https://mirdb.org/cgi-bin/target_detail.cgi?targetID=2854191) | 791 | 75 | hsa-miR-20a-5p | [RAPGEF4](http://www.ncbi.nlm.nih.gov/entrez/query.fcgi?db=gene&cmd=Retrieve&dopt=full_report&list_uids=11069) | Rap guanine nucleotide exchange factor 4 |
| [Details](https://mirdb.org/cgi-bin/target_detail.cgi?targetID=2854203) | 792 | 75 | hsa-miR-20a-5p | [NDNF](http://www.ncbi.nlm.nih.gov/entrez/query.fcgi?db=gene&cmd=Retrieve&dopt=full_report&list_uids=79625) | neuron derived neurotrophic factor |
| [Details](https://mirdb.org/cgi-bin/target_detail.cgi?targetID=2854357) | 793 | 75 | hsa-miR-20a-5p | [COMMD6](http://www.ncbi.nlm.nih.gov/entrez/query.fcgi?db=gene&cmd=Retrieve&dopt=full_report&list_uids=170622) | COMM domain containing 6 |
| [Details](https://mirdb.org/cgi-bin/target_detail.cgi?targetID=2854360) | 794 | 75 | hsa-miR-20a-5p | [SKIL](http://www.ncbi.nlm.nih.gov/entrez/query.fcgi?db=gene&cmd=Retrieve&dopt=full_report&list_uids=6498) | SKI like proto-oncogene |
| [Details](https://mirdb.org/cgi-bin/target_detail.cgi?targetID=2854364) | 795 | 75 | hsa-miR-20a-5p | [MAPRE1](http://www.ncbi.nlm.nih.gov/entrez/query.fcgi?db=gene&cmd=Retrieve&dopt=full_report&list_uids=22919) | microtubule associated protein RP/EB family member 1 |
| [Details](https://mirdb.org/cgi-bin/target_detail.cgi?targetID=2854402) | 796 | 75 | hsa-miR-20a-5p | [KIF5A](http://www.ncbi.nlm.nih.gov/entrez/query.fcgi?db=gene&cmd=Retrieve&dopt=full_report&list_uids=3798) | kinesin family member 5A |
| [Details](https://mirdb.org/cgi-bin/target_detail.cgi?targetID=2854461) | 797 | 75 | hsa-miR-20a-5p | [RASGEF1A](http://www.ncbi.nlm.nih.gov/entrez/query.fcgi?db=gene&cmd=Retrieve&dopt=full_report&list_uids=221002) | RasGEF domain family member 1A |
| [Details](https://mirdb.org/cgi-bin/target_detail.cgi?targetID=2854469) | 798 | 75 | hsa-miR-20a-5p | [RNF38](http://www.ncbi.nlm.nih.gov/entrez/query.fcgi?db=gene&cmd=Retrieve&dopt=full_report&list_uids=152006) | ring finger protein 38 |
| [Details](https://mirdb.org/cgi-bin/target_detail.cgi?targetID=2854489) | 799 | 75 | hsa-miR-20a-5p | [ACIN1](http://www.ncbi.nlm.nih.gov/entrez/query.fcgi?db=gene&cmd=Retrieve&dopt=full_report&list_uids=22985) | apoptotic chromatin condensation inducer 1 |
| [Details](https://mirdb.org/cgi-bin/target_detail.cgi?targetID=2854519) | 800 | 75 | hsa-miR-20a-5p | [SNTG1](http://www.ncbi.nlm.nih.gov/entrez/query.fcgi?db=gene&cmd=Retrieve&dopt=full_report&list_uids=54212) | syntrophin gamma 1 |
| [Details](https://mirdb.org/cgi-bin/target_detail.cgi?targetID=2854528) | 801 | 75 | hsa-miR-20a-5p | [KLF10](http://www.ncbi.nlm.nih.gov/entrez/query.fcgi?db=gene&cmd=Retrieve&dopt=full_report&list_uids=7071) | Kruppel like factor 10 |
| [Details](https://mirdb.org/cgi-bin/target_detail.cgi?targetID=2854574) | 802 | 75 | hsa-miR-20a-5p | [STARD8](http://www.ncbi.nlm.nih.gov/entrez/query.fcgi?db=gene&cmd=Retrieve&dopt=full_report&list_uids=9754) | StAR related lipid transfer domain containing 8 |
| [Details](https://mirdb.org/cgi-bin/target_detail.cgi?targetID=2854630) | 803 | 75 | hsa-miR-20a-5p | [MAP3K12](http://www.ncbi.nlm.nih.gov/entrez/query.fcgi?db=gene&cmd=Retrieve&dopt=full_report&list_uids=7786) | mitogen-activated protein kinase kinase kinase 12 |
| [Details](https://mirdb.org/cgi-bin/target_detail.cgi?targetID=2854635) | 804 | 75 | hsa-miR-20a-5p | [SHOC2](http://www.ncbi.nlm.nih.gov/entrez/query.fcgi?db=gene&cmd=Retrieve&dopt=full_report&list_uids=8036) | SHOC2, leucine rich repeat scaffold protein |
| [Details](https://mirdb.org/cgi-bin/target_detail.cgi?targetID=2854645) | 805 | 75 | hsa-miR-20a-5p | [TXLNA](http://www.ncbi.nlm.nih.gov/entrez/query.fcgi?db=gene&cmd=Retrieve&dopt=full_report&list_uids=200081) | taxilin alpha |
| [Details](https://mirdb.org/cgi-bin/target_detail.cgi?targetID=2854713) | 806 | 75 | hsa-miR-20a-5p | [NR4A2](http://www.ncbi.nlm.nih.gov/entrez/query.fcgi?db=gene&cmd=Retrieve&dopt=full_report&list_uids=4929) | nuclear receptor subfamily 4 group A member 2 |
| [Details](https://mirdb.org/cgi-bin/target_detail.cgi?targetID=2854795) | 807 | 75 | hsa-miR-20a-5p | [ALX4](http://www.ncbi.nlm.nih.gov/entrez/query.fcgi?db=gene&cmd=Retrieve&dopt=full_report&list_uids=60529) | ALX homeobox 4 |
| [Details](https://mirdb.org/cgi-bin/target_detail.cgi?targetID=2854796) | 808 | 75 | hsa-miR-20a-5p | [ITPKB](http://www.ncbi.nlm.nih.gov/entrez/query.fcgi?db=gene&cmd=Retrieve&dopt=full_report&list_uids=3707) | inositol-trisphosphate 3-kinase B |
| [Details](https://mirdb.org/cgi-bin/target_detail.cgi?targetID=2855145) | 809 | 75 | hsa-miR-20a-5p | [RNASEL](http://www.ncbi.nlm.nih.gov/entrez/query.fcgi?db=gene&cmd=Retrieve&dopt=full_report&list_uids=6041) | ribonuclease L |
| [Details](https://mirdb.org/cgi-bin/target_detail.cgi?targetID=2855181) | 810 | 75 | hsa-miR-20a-5p | [TASOR](http://www.ncbi.nlm.nih.gov/entrez/query.fcgi?db=gene&cmd=Retrieve&dopt=full_report&list_uids=23272) | transcription activation suppressor |
| [Details](https://mirdb.org/cgi-bin/target_detail.cgi?targetID=2855185) | 811 | 75 | hsa-miR-20a-5p | [IL25](http://www.ncbi.nlm.nih.gov/entrez/query.fcgi?db=gene&cmd=Retrieve&dopt=full_report&list_uids=64806) | interleukin 25 |
| [Details](https://mirdb.org/cgi-bin/target_detail.cgi?targetID=2855231) | 812 | 75 | hsa-miR-20a-5p | [SYT17](http://www.ncbi.nlm.nih.gov/entrez/query.fcgi?db=gene&cmd=Retrieve&dopt=full_report&list_uids=51760) | synaptotagmin 17 |
| [Details](https://mirdb.org/cgi-bin/target_detail.cgi?targetID=2855321) | 813 | 75 | hsa-miR-20a-5p | [LRP1B](http://www.ncbi.nlm.nih.gov/entrez/query.fcgi?db=gene&cmd=Retrieve&dopt=full_report&list_uids=53353) | LDL receptor related protein 1B |
| [Details](https://mirdb.org/cgi-bin/target_detail.cgi?targetID=2855344) | 814 | 75 | hsa-miR-20a-5p | [CLEC4D](http://www.ncbi.nlm.nih.gov/entrez/query.fcgi?db=gene&cmd=Retrieve&dopt=full_report&list_uids=338339) | C-type lectin domain family 4 member D |
| [Details](https://mirdb.org/cgi-bin/target_detail.cgi?targetID=2855424) | 815 | 75 | hsa-miR-20a-5p | [ADGRD1](http://www.ncbi.nlm.nih.gov/entrez/query.fcgi?db=gene&cmd=Retrieve&dopt=full_report&list_uids=283383) | adhesion G protein-coupled receptor D1 |
| [Details](https://mirdb.org/cgi-bin/target_detail.cgi?targetID=2854115) | 816 | 74 | hsa-miR-20a-5p | [ZNF445](http://www.ncbi.nlm.nih.gov/entrez/query.fcgi?db=gene&cmd=Retrieve&dopt=full_report&list_uids=353274) | zinc finger protein 445 |
| [Details](https://mirdb.org/cgi-bin/target_detail.cgi?targetID=2854239) | 817 | 74 | hsa-miR-20a-5p | [CLEC12A](http://www.ncbi.nlm.nih.gov/entrez/query.fcgi?db=gene&cmd=Retrieve&dopt=full_report&list_uids=160364) | C-type lectin domain family 12 member A |
| [Details](https://mirdb.org/cgi-bin/target_detail.cgi?targetID=2854333) | 818 | 74 | hsa-miR-20a-5p | [RNF2](http://www.ncbi.nlm.nih.gov/entrez/query.fcgi?db=gene&cmd=Retrieve&dopt=full_report&list_uids=6045) | ring finger protein 2 |
| [Details](https://mirdb.org/cgi-bin/target_detail.cgi?targetID=2854340) | 819 | 74 | hsa-miR-20a-5p | [MAP3K13](http://www.ncbi.nlm.nih.gov/entrez/query.fcgi?db=gene&cmd=Retrieve&dopt=full_report&list_uids=9175) | mitogen-activated protein kinase kinase kinase 13 |
| [Details](https://mirdb.org/cgi-bin/target_detail.cgi?targetID=2854425) | 820 | 74 | hsa-miR-20a-5p | [F2R](http://www.ncbi.nlm.nih.gov/entrez/query.fcgi?db=gene&cmd=Retrieve&dopt=full_report&list_uids=2149) | coagulation factor II thrombin receptor |
| [Details](https://mirdb.org/cgi-bin/target_detail.cgi?targetID=2854436) | 821 | 74 | hsa-miR-20a-5p | [FAM53A](http://www.ncbi.nlm.nih.gov/entrez/query.fcgi?db=gene&cmd=Retrieve&dopt=full_report&list_uids=152877) | family with sequence similarity 53 member A |
| [Details](https://mirdb.org/cgi-bin/target_detail.cgi?targetID=2854527) | 822 | 74 | hsa-miR-20a-5p | [LIF](http://www.ncbi.nlm.nih.gov/entrez/query.fcgi?db=gene&cmd=Retrieve&dopt=full_report&list_uids=3976) | LIF, interleukin 6 family cytokine |
| [Details](https://mirdb.org/cgi-bin/target_detail.cgi?targetID=2854695) | 823 | 74 | hsa-miR-20a-5p | [DLEC1](http://www.ncbi.nlm.nih.gov/entrez/query.fcgi?db=gene&cmd=Retrieve&dopt=full_report&list_uids=9940) | DLEC1, cilia and flagella associated protein |
| [Details](https://mirdb.org/cgi-bin/target_detail.cgi?targetID=2854698) | 824 | 74 | hsa-miR-20a-5p | [KCNE4](http://www.ncbi.nlm.nih.gov/entrez/query.fcgi?db=gene&cmd=Retrieve&dopt=full_report&list_uids=23704) | potassium voltage-gated channel subfamily E regulatory subunit 4 |
| [Details](https://mirdb.org/cgi-bin/target_detail.cgi?targetID=2854743) | 825 | 74 | hsa-miR-20a-5p | [COA5](http://www.ncbi.nlm.nih.gov/entrez/query.fcgi?db=gene&cmd=Retrieve&dopt=full_report&list_uids=493753) | cytochrome c oxidase assembly factor 5 |
| [Details](https://mirdb.org/cgi-bin/target_detail.cgi?targetID=2854940) | 826 | 74 | hsa-miR-20a-5p | [AP3D1](http://www.ncbi.nlm.nih.gov/entrez/query.fcgi?db=gene&cmd=Retrieve&dopt=full_report&list_uids=8943) | adaptor related protein complex 3 subunit delta 1 |
| [Details](https://mirdb.org/cgi-bin/target_detail.cgi?targetID=2855076) | 827 | 74 | hsa-miR-20a-5p | [RPS6KA1](http://www.ncbi.nlm.nih.gov/entrez/query.fcgi?db=gene&cmd=Retrieve&dopt=full_report&list_uids=6195) | ribosomal protein S6 kinase A1 |
| [Details](https://mirdb.org/cgi-bin/target_detail.cgi?targetID=2855096) | 828 | 74 | hsa-miR-20a-5p | [BECN1](http://www.ncbi.nlm.nih.gov/entrez/query.fcgi?db=gene&cmd=Retrieve&dopt=full_report&list_uids=8678) | beclin 1 |
| [Details](https://mirdb.org/cgi-bin/target_detail.cgi?targetID=2855201) | 829 | 74 | hsa-miR-20a-5p | [ATG7](http://www.ncbi.nlm.nih.gov/entrez/query.fcgi?db=gene&cmd=Retrieve&dopt=full_report&list_uids=10533) | autophagy related 7 |
| [Details](https://mirdb.org/cgi-bin/target_detail.cgi?targetID=2855209) | 830 | 74 | hsa-miR-20a-5p | [PKN2](http://www.ncbi.nlm.nih.gov/entrez/query.fcgi?db=gene&cmd=Retrieve&dopt=full_report&list_uids=5586) | protein kinase N2 |
| [Details](https://mirdb.org/cgi-bin/target_detail.cgi?targetID=2855210) | 831 | 74 | hsa-miR-20a-5p | [COX8C](http://www.ncbi.nlm.nih.gov/entrez/query.fcgi?db=gene&cmd=Retrieve&dopt=full_report&list_uids=341947) | cytochrome c oxidase subunit 8C |
| [Details](https://mirdb.org/cgi-bin/target_detail.cgi?targetID=2855264) | 832 | 74 | hsa-miR-20a-5p | [SESN2](http://www.ncbi.nlm.nih.gov/entrez/query.fcgi?db=gene&cmd=Retrieve&dopt=full_report&list_uids=83667) | sestrin 2 |
| [Details](https://mirdb.org/cgi-bin/target_detail.cgi?targetID=2855311) | 833 | 74 | hsa-miR-20a-5p | [MSTO1](http://www.ncbi.nlm.nih.gov/entrez/query.fcgi?db=gene&cmd=Retrieve&dopt=full_report&list_uids=55154) | misato mitochondrial distribution and morphology regulator 1 |
| [Details](https://mirdb.org/cgi-bin/target_detail.cgi?targetID=2855313) | 834 | 74 | hsa-miR-20a-5p | [MAPK9](http://www.ncbi.nlm.nih.gov/entrez/query.fcgi?db=gene&cmd=Retrieve&dopt=full_report&list_uids=5601) | mitogen-activated protein kinase 9 |
| [Details](https://mirdb.org/cgi-bin/target_detail.cgi?targetID=2855388) | 835 | 74 | hsa-miR-20a-5p | [GPC6](http://www.ncbi.nlm.nih.gov/entrez/query.fcgi?db=gene&cmd=Retrieve&dopt=full_report&list_uids=10082) | glypican 6 |
| [Details](https://mirdb.org/cgi-bin/target_detail.cgi?targetID=2855410) | 836 | 74 | hsa-miR-20a-5p | [PRRX1](http://www.ncbi.nlm.nih.gov/entrez/query.fcgi?db=gene&cmd=Retrieve&dopt=full_report&list_uids=5396) | paired related homeobox 1 |
| [Details](https://mirdb.org/cgi-bin/target_detail.cgi?targetID=2855431) | 837 | 74 | hsa-miR-20a-5p | [SLC29A2](http://www.ncbi.nlm.nih.gov/entrez/query.fcgi?db=gene&cmd=Retrieve&dopt=full_report&list_uids=3177) | solute carrier family 29 member 2 |
| [Details](https://mirdb.org/cgi-bin/target_detail.cgi?targetID=2854149) | 838 | 73 | hsa-miR-20a-5p | [PFN2](http://www.ncbi.nlm.nih.gov/entrez/query.fcgi?db=gene&cmd=Retrieve&dopt=full_report&list_uids=5217) | profilin 2 |
| [Details](https://mirdb.org/cgi-bin/target_detail.cgi?targetID=2854264) | 839 | 73 | hsa-miR-20a-5p | [CNTNAP3B](http://www.ncbi.nlm.nih.gov/entrez/query.fcgi?db=gene&cmd=Retrieve&dopt=full_report&list_uids=728577) | contactin associated protein like 3B |
| [Details](https://mirdb.org/cgi-bin/target_detail.cgi?targetID=2854304) | 840 | 73 | hsa-miR-20a-5p | [TSHZ3](http://www.ncbi.nlm.nih.gov/entrez/query.fcgi?db=gene&cmd=Retrieve&dopt=full_report&list_uids=57616) | teashirt zinc finger homeobox 3 |
| [Details](https://mirdb.org/cgi-bin/target_detail.cgi?targetID=2854327) | 841 | 73 | hsa-miR-20a-5p | [EIF4G2](http://www.ncbi.nlm.nih.gov/entrez/query.fcgi?db=gene&cmd=Retrieve&dopt=full_report&list_uids=1982) | eukaryotic translation initiation factor 4 gamma 2 |
| [Details](https://mirdb.org/cgi-bin/target_detail.cgi?targetID=2854444) | 842 | 73 | hsa-miR-20a-5p | [GINS4](http://www.ncbi.nlm.nih.gov/entrez/query.fcgi?db=gene&cmd=Retrieve&dopt=full_report&list_uids=84296) | GINS complex subunit 4 |
| [Details](https://mirdb.org/cgi-bin/target_detail.cgi?targetID=2854464) | 843 | 73 | hsa-miR-20a-5p | [E2F3](http://www.ncbi.nlm.nih.gov/entrez/query.fcgi?db=gene&cmd=Retrieve&dopt=full_report&list_uids=1871) | E2F transcription factor 3 |
| [Details](https://mirdb.org/cgi-bin/target_detail.cgi?targetID=2854477) | 844 | 73 | hsa-miR-20a-5p | [NR4A3](http://www.ncbi.nlm.nih.gov/entrez/query.fcgi?db=gene&cmd=Retrieve&dopt=full_report&list_uids=8013) | nuclear receptor subfamily 4 group A member 3 |
| [Details](https://mirdb.org/cgi-bin/target_detail.cgi?targetID=2854534) | 845 | 73 | hsa-miR-20a-5p | [LHX8](http://www.ncbi.nlm.nih.gov/entrez/query.fcgi?db=gene&cmd=Retrieve&dopt=full_report&list_uids=431707) | LIM homeobox 8 |
| [Details](https://mirdb.org/cgi-bin/target_detail.cgi?targetID=2854597) | 846 | 73 | hsa-miR-20a-5p | [GID4](http://www.ncbi.nlm.nih.gov/entrez/query.fcgi?db=gene&cmd=Retrieve&dopt=full_report&list_uids=79018) | GID complex subunit 4 homolog |
| [Details](https://mirdb.org/cgi-bin/target_detail.cgi?targetID=2854678) | 847 | 73 | hsa-miR-20a-5p | [ZBTB6](http://www.ncbi.nlm.nih.gov/entrez/query.fcgi?db=gene&cmd=Retrieve&dopt=full_report&list_uids=10773) | zinc finger and BTB domain containing 6 |
| [Details](https://mirdb.org/cgi-bin/target_detail.cgi?targetID=2854688) | 848 | 73 | hsa-miR-20a-5p | [FAM129B](http://www.ncbi.nlm.nih.gov/entrez/query.fcgi?db=gene&cmd=Retrieve&dopt=full_report&list_uids=64855) | family with sequence similarity 129 member B |
| [Details](https://mirdb.org/cgi-bin/target_detail.cgi?targetID=2854700) | 849 | 73 | hsa-miR-20a-5p | [TP53INP1](http://www.ncbi.nlm.nih.gov/entrez/query.fcgi?db=gene&cmd=Retrieve&dopt=full_report&list_uids=94241) | tumor protein p53 inducible nuclear protein 1 |
| [Details](https://mirdb.org/cgi-bin/target_detail.cgi?targetID=2854715) | 850 | 73 | hsa-miR-20a-5p | [HYDIN](http://www.ncbi.nlm.nih.gov/entrez/query.fcgi?db=gene&cmd=Retrieve&dopt=full_report&list_uids=54768) | HYDIN, axonemal central pair apparatus protein |
| [Details](https://mirdb.org/cgi-bin/target_detail.cgi?targetID=2854731) | 851 | 73 | hsa-miR-20a-5p | [SLC12A3](http://www.ncbi.nlm.nih.gov/entrez/query.fcgi?db=gene&cmd=Retrieve&dopt=full_report&list_uids=6559) | solute carrier family 12 member 3 |
| [Details](https://mirdb.org/cgi-bin/target_detail.cgi?targetID=2854934) | 852 | 73 | hsa-miR-20a-5p | [ARSJ](http://www.ncbi.nlm.nih.gov/entrez/query.fcgi?db=gene&cmd=Retrieve&dopt=full_report&list_uids=79642) | arylsulfatase family member J |
| [Details](https://mirdb.org/cgi-bin/target_detail.cgi?targetID=2854950) | 853 | 73 | hsa-miR-20a-5p | [ZC3H7B](http://www.ncbi.nlm.nih.gov/entrez/query.fcgi?db=gene&cmd=Retrieve&dopt=full_report&list_uids=23264) | zinc finger CCCH-type containing 7B |
| [Details](https://mirdb.org/cgi-bin/target_detail.cgi?targetID=2854964) | 854 | 73 | hsa-miR-20a-5p | [PDPR](http://www.ncbi.nlm.nih.gov/entrez/query.fcgi?db=gene&cmd=Retrieve&dopt=full_report&list_uids=55066) | pyruvate dehydrogenase phosphatase regulatory subunit |
| [Details](https://mirdb.org/cgi-bin/target_detail.cgi?targetID=2854965) | 855 | 73 | hsa-miR-20a-5p | [MUC17](http://www.ncbi.nlm.nih.gov/entrez/query.fcgi?db=gene&cmd=Retrieve&dopt=full_report&list_uids=140453) | mucin 17, cell surface associated |
| [Details](https://mirdb.org/cgi-bin/target_detail.cgi?targetID=2855077) | 856 | 73 | hsa-miR-20a-5p | [EGR3](http://www.ncbi.nlm.nih.gov/entrez/query.fcgi?db=gene&cmd=Retrieve&dopt=full_report&list_uids=1960) | early growth response 3 |
| [Details](https://mirdb.org/cgi-bin/target_detail.cgi?targetID=2855387) | 857 | 73 | hsa-miR-20a-5p | [PRICKLE3](http://www.ncbi.nlm.nih.gov/entrez/query.fcgi?db=gene&cmd=Retrieve&dopt=full_report&list_uids=4007) | prickle planar cell polarity protein 3 |
| [Details](https://mirdb.org/cgi-bin/target_detail.cgi?targetID=2854084) | 858 | 72 | hsa-miR-20a-5p | [NSL1](http://www.ncbi.nlm.nih.gov/entrez/query.fcgi?db=gene&cmd=Retrieve&dopt=full_report&list_uids=25936) | NSL1, MIS12 kinetochore complex component |
| [Details](https://mirdb.org/cgi-bin/target_detail.cgi?targetID=2854103) | 859 | 72 | hsa-miR-20a-5p | [HMGB3](http://www.ncbi.nlm.nih.gov/entrez/query.fcgi?db=gene&cmd=Retrieve&dopt=full_report&list_uids=3149) | high mobility group box 3 |
| [Details](https://mirdb.org/cgi-bin/target_detail.cgi?targetID=2854145) | 860 | 72 | hsa-miR-20a-5p | [CACUL1](http://www.ncbi.nlm.nih.gov/entrez/query.fcgi?db=gene&cmd=Retrieve&dopt=full_report&list_uids=143384) | CDK2 associated cullin domain 1 |
| [Details](https://mirdb.org/cgi-bin/target_detail.cgi?targetID=2854163) | 861 | 72 | hsa-miR-20a-5p | [PTPRJ](http://www.ncbi.nlm.nih.gov/entrez/query.fcgi?db=gene&cmd=Retrieve&dopt=full_report&list_uids=5795) | protein tyrosine phosphatase, receptor type J |
| [Details](https://mirdb.org/cgi-bin/target_detail.cgi?targetID=2854178) | 862 | 72 | hsa-miR-20a-5p | [CDH24](http://www.ncbi.nlm.nih.gov/entrez/query.fcgi?db=gene&cmd=Retrieve&dopt=full_report&list_uids=64403) | cadherin 24 |
| [Details](https://mirdb.org/cgi-bin/target_detail.cgi?targetID=2854200) | 863 | 72 | hsa-miR-20a-5p | [GON4L](http://www.ncbi.nlm.nih.gov/entrez/query.fcgi?db=gene&cmd=Retrieve&dopt=full_report&list_uids=54856) | gon-4 like |
| [Details](https://mirdb.org/cgi-bin/target_detail.cgi?targetID=2854321) | 864 | 72 | hsa-miR-20a-5p | [BMP2](http://www.ncbi.nlm.nih.gov/entrez/query.fcgi?db=gene&cmd=Retrieve&dopt=full_report&list_uids=650) | bone morphogenetic protein 2 |
| [Details](https://mirdb.org/cgi-bin/target_detail.cgi?targetID=2854345) | 865 | 72 | hsa-miR-20a-5p | [TSC22D2](http://www.ncbi.nlm.nih.gov/entrez/query.fcgi?db=gene&cmd=Retrieve&dopt=full_report&list_uids=9819) | TSC22 domain family member 2 |
| [Details](https://mirdb.org/cgi-bin/target_detail.cgi?targetID=2854390) | 866 | 72 | hsa-miR-20a-5p | [POLQ](http://www.ncbi.nlm.nih.gov/entrez/query.fcgi?db=gene&cmd=Retrieve&dopt=full_report&list_uids=10721) | DNA polymerase theta |
| [Details](https://mirdb.org/cgi-bin/target_detail.cgi?targetID=2854451) | 867 | 72 | hsa-miR-20a-5p | [SIK1](http://www.ncbi.nlm.nih.gov/entrez/query.fcgi?db=gene&cmd=Retrieve&dopt=full_report&list_uids=150094) | salt inducible kinase 1 |
| [Details](https://mirdb.org/cgi-bin/target_detail.cgi?targetID=2854513) | 868 | 72 | hsa-miR-20a-5p | [NRP2](http://www.ncbi.nlm.nih.gov/entrez/query.fcgi?db=gene&cmd=Retrieve&dopt=full_report&list_uids=8828) | neuropilin 2 |
| [Details](https://mirdb.org/cgi-bin/target_detail.cgi?targetID=2854575) | 869 | 72 | hsa-miR-20a-5p | [CEP128](http://www.ncbi.nlm.nih.gov/entrez/query.fcgi?db=gene&cmd=Retrieve&dopt=full_report&list_uids=145508) | centrosomal protein 128 |
| [Details](https://mirdb.org/cgi-bin/target_detail.cgi?targetID=2854723) | 870 | 72 | hsa-miR-20a-5p | [MAT2B](http://www.ncbi.nlm.nih.gov/entrez/query.fcgi?db=gene&cmd=Retrieve&dopt=full_report&list_uids=27430) | methionine adenosyltransferase 2B |
| [Details](https://mirdb.org/cgi-bin/target_detail.cgi?targetID=2854727) | 871 | 72 | hsa-miR-20a-5p | [CAAP1](http://www.ncbi.nlm.nih.gov/entrez/query.fcgi?db=gene&cmd=Retrieve&dopt=full_report&list_uids=79886) | caspase activity and apoptosis inhibitor 1 |
| [Details](https://mirdb.org/cgi-bin/target_detail.cgi?targetID=2854823) | 872 | 72 | hsa-miR-20a-5p | [ITFG1](http://www.ncbi.nlm.nih.gov/entrez/query.fcgi?db=gene&cmd=Retrieve&dopt=full_report&list_uids=81533) | integrin alpha FG-GAP repeat containing 1 |
| [Details](https://mirdb.org/cgi-bin/target_detail.cgi?targetID=2854937) | 873 | 72 | hsa-miR-20a-5p | [ARHGAP35](http://www.ncbi.nlm.nih.gov/entrez/query.fcgi?db=gene&cmd=Retrieve&dopt=full_report&list_uids=2909) | Rho GTPase activating protein 35 |
| [Details](https://mirdb.org/cgi-bin/target_detail.cgi?targetID=2854951) | 874 | 72 | hsa-miR-20a-5p | [FGFR1OP](http://www.ncbi.nlm.nih.gov/entrez/query.fcgi?db=gene&cmd=Retrieve&dopt=full_report&list_uids=11116) | FGFR1 oncogene partner |
| [Details](https://mirdb.org/cgi-bin/target_detail.cgi?targetID=2855043) | 875 | 72 | hsa-miR-20a-5p | [IFIT5](http://www.ncbi.nlm.nih.gov/entrez/query.fcgi?db=gene&cmd=Retrieve&dopt=full_report&list_uids=24138) | interferon induced protein with tetratricopeptide repeats 5 |
| [Details](https://mirdb.org/cgi-bin/target_detail.cgi?targetID=2855054) | 876 | 72 | hsa-miR-20a-5p | [DPYD](http://www.ncbi.nlm.nih.gov/entrez/query.fcgi?db=gene&cmd=Retrieve&dopt=full_report&list_uids=1806) | dihydropyrimidine dehydrogenase |
| [Details](https://mirdb.org/cgi-bin/target_detail.cgi?targetID=2855114) | 877 | 72 | hsa-miR-20a-5p | [HP1BP3](http://www.ncbi.nlm.nih.gov/entrez/query.fcgi?db=gene&cmd=Retrieve&dopt=full_report&list_uids=50809) | heterochromatin protein 1 binding protein 3 |
| [Details](https://mirdb.org/cgi-bin/target_detail.cgi?targetID=2855153) | 878 | 72 | hsa-miR-20a-5p | [SFXN5](http://www.ncbi.nlm.nih.gov/entrez/query.fcgi?db=gene&cmd=Retrieve&dopt=full_report&list_uids=94097) | sideroflexin 5 |
| [Details](https://mirdb.org/cgi-bin/target_detail.cgi?targetID=2855237) | 879 | 72 | hsa-miR-20a-5p | [SHANK2](http://www.ncbi.nlm.nih.gov/entrez/query.fcgi?db=gene&cmd=Retrieve&dopt=full_report&list_uids=22941) | SH3 and multiple ankyrin repeat domains 2 |
| [Details](https://mirdb.org/cgi-bin/target_detail.cgi?targetID=2855262) | 880 | 72 | hsa-miR-20a-5p | [CERS2](http://www.ncbi.nlm.nih.gov/entrez/query.fcgi?db=gene&cmd=Retrieve&dopt=full_report&list_uids=29956) | ceramide synthase 2 |
| [Details](https://mirdb.org/cgi-bin/target_detail.cgi?targetID=2855434) | 881 | 72 | hsa-miR-20a-5p | [TRAF4](http://www.ncbi.nlm.nih.gov/entrez/query.fcgi?db=gene&cmd=Retrieve&dopt=full_report&list_uids=9618) | TNF receptor associated factor 4 |
| [Details](https://mirdb.org/cgi-bin/target_detail.cgi?targetID=2854081) | 882 | 71 | hsa-miR-20a-5p | [HMBOX1](http://www.ncbi.nlm.nih.gov/entrez/query.fcgi?db=gene&cmd=Retrieve&dopt=full_report&list_uids=79618) | homeobox containing 1 |
| [Details](https://mirdb.org/cgi-bin/target_detail.cgi?targetID=2854188) | 883 | 71 | hsa-miR-20a-5p | [SLC31A2](http://www.ncbi.nlm.nih.gov/entrez/query.fcgi?db=gene&cmd=Retrieve&dopt=full_report&list_uids=1318) | solute carrier family 31 member 2 |
| [Details](https://mirdb.org/cgi-bin/target_detail.cgi?targetID=2854218) | 884 | 71 | hsa-miR-20a-5p | [TMEM131L](http://www.ncbi.nlm.nih.gov/entrez/query.fcgi?db=gene&cmd=Retrieve&dopt=full_report&list_uids=23240) | transmembrane 131 like |
| [Details](https://mirdb.org/cgi-bin/target_detail.cgi?targetID=2854247) | 885 | 71 | hsa-miR-20a-5p | [GLO1](http://www.ncbi.nlm.nih.gov/entrez/query.fcgi?db=gene&cmd=Retrieve&dopt=full_report&list_uids=2739) | glyoxalase I |
| [Details](https://mirdb.org/cgi-bin/target_detail.cgi?targetID=2854261) | 886 | 71 | hsa-miR-20a-5p | [CADM2](http://www.ncbi.nlm.nih.gov/entrez/query.fcgi?db=gene&cmd=Retrieve&dopt=full_report&list_uids=253559) | cell adhesion molecule 2 |
| [Details](https://mirdb.org/cgi-bin/target_detail.cgi?targetID=2854371) | 887 | 71 | hsa-miR-20a-5p | [MPDZ](http://www.ncbi.nlm.nih.gov/entrez/query.fcgi?db=gene&cmd=Retrieve&dopt=full_report&list_uids=8777) | multiple PDZ domain crumbs cell polarity complex component |
| [Details](https://mirdb.org/cgi-bin/target_detail.cgi?targetID=2854490) | 888 | 71 | hsa-miR-20a-5p | [MYO5C](http://www.ncbi.nlm.nih.gov/entrez/query.fcgi?db=gene&cmd=Retrieve&dopt=full_report&list_uids=55930) | myosin VC |
| [Details](https://mirdb.org/cgi-bin/target_detail.cgi?targetID=2854521) | 889 | 71 | hsa-miR-20a-5p | [LUZP1](http://www.ncbi.nlm.nih.gov/entrez/query.fcgi?db=gene&cmd=Retrieve&dopt=full_report&list_uids=7798) | leucine zipper protein 1 |
| [Details](https://mirdb.org/cgi-bin/target_detail.cgi?targetID=2854550) | 890 | 71 | hsa-miR-20a-5p | [KLHL36](http://www.ncbi.nlm.nih.gov/entrez/query.fcgi?db=gene&cmd=Retrieve&dopt=full_report&list_uids=79786) | kelch like family member 36 |
| [Details](https://mirdb.org/cgi-bin/target_detail.cgi?targetID=2854614) | 891 | 71 | hsa-miR-20a-5p | [CSGALNACT1](http://www.ncbi.nlm.nih.gov/entrez/query.fcgi?db=gene&cmd=Retrieve&dopt=full_report&list_uids=55790) | chondroitin sulfate N-acetylgalactosaminyltransferase 1 |
| [Details](https://mirdb.org/cgi-bin/target_detail.cgi?targetID=2854626) | 892 | 71 | hsa-miR-20a-5p | [RELL1](http://www.ncbi.nlm.nih.gov/entrez/query.fcgi?db=gene&cmd=Retrieve&dopt=full_report&list_uids=768211) | RELT like 1 |
| [Details](https://mirdb.org/cgi-bin/target_detail.cgi?targetID=2854740) | 893 | 71 | hsa-miR-20a-5p | [YPEL2](http://www.ncbi.nlm.nih.gov/entrez/query.fcgi?db=gene&cmd=Retrieve&dopt=full_report&list_uids=388403) | yippee like 2 |
| [Details](https://mirdb.org/cgi-bin/target_detail.cgi?targetID=2854742) | 894 | 71 | hsa-miR-20a-5p | [SIK1B](http://www.ncbi.nlm.nih.gov/entrez/query.fcgi?db=gene&cmd=Retrieve&dopt=full_report&list_uids=102724428) | salt inducible kinase 1B (putative) |
| [Details](https://mirdb.org/cgi-bin/target_detail.cgi?targetID=2854779) | 895 | 71 | hsa-miR-20a-5p | [TCEAL7](http://www.ncbi.nlm.nih.gov/entrez/query.fcgi?db=gene&cmd=Retrieve&dopt=full_report&list_uids=56849) | transcription elongation factor A like 7 |
| [Details](https://mirdb.org/cgi-bin/target_detail.cgi?targetID=2854821) | 896 | 71 | hsa-miR-20a-5p | [TCF7L1](http://www.ncbi.nlm.nih.gov/entrez/query.fcgi?db=gene&cmd=Retrieve&dopt=full_report&list_uids=83439) | transcription factor 7 like 1 |
| [Details](https://mirdb.org/cgi-bin/target_detail.cgi?targetID=2854868) | 897 | 71 | hsa-miR-20a-5p | [SASH1](http://www.ncbi.nlm.nih.gov/entrez/query.fcgi?db=gene&cmd=Retrieve&dopt=full_report&list_uids=23328) | SAM and SH3 domain containing 1 |
| [Details](https://mirdb.org/cgi-bin/target_detail.cgi?targetID=2854920) | 898 | 71 | hsa-miR-20a-5p | [RPF2](http://www.ncbi.nlm.nih.gov/entrez/query.fcgi?db=gene&cmd=Retrieve&dopt=full_report&list_uids=84154) | ribosome production factor 2 homolog |
| [Details](https://mirdb.org/cgi-bin/target_detail.cgi?targetID=2854941) | 899 | 71 | hsa-miR-20a-5p | [SOGA1](http://www.ncbi.nlm.nih.gov/entrez/query.fcgi?db=gene&cmd=Retrieve&dopt=full_report&list_uids=140710) | suppressor of glucose, autophagy associated 1 |
| [Details](https://mirdb.org/cgi-bin/target_detail.cgi?targetID=2855082) | 900 | 71 | hsa-miR-20a-5p | [SRPK1](http://www.ncbi.nlm.nih.gov/entrez/query.fcgi?db=gene&cmd=Retrieve&dopt=full_report&list_uids=6732) | SRSF protein kinase 1 |
| [Details](https://mirdb.org/cgi-bin/target_detail.cgi?targetID=2855171) | 901 | 71 | hsa-miR-20a-5p | [FMNL3](http://www.ncbi.nlm.nih.gov/entrez/query.fcgi?db=gene&cmd=Retrieve&dopt=full_report&list_uids=91010) | formin like 3 |
| [Details](https://mirdb.org/cgi-bin/target_detail.cgi?targetID=2855204) | 902 | 71 | hsa-miR-20a-5p | [EPGN](http://www.ncbi.nlm.nih.gov/entrez/query.fcgi?db=gene&cmd=Retrieve&dopt=full_report&list_uids=255324) | epithelial mitogen |
| [Details](https://mirdb.org/cgi-bin/target_detail.cgi?targetID=2855247) | 903 | 71 | hsa-miR-20a-5p | [PLSCR4](http://www.ncbi.nlm.nih.gov/entrez/query.fcgi?db=gene&cmd=Retrieve&dopt=full_report&list_uids=57088) | phospholipid scramblase 4 |
| [Details](https://mirdb.org/cgi-bin/target_detail.cgi?targetID=2855256) | 904 | 71 | hsa-miR-20a-5p | [GBP3](http://www.ncbi.nlm.nih.gov/entrez/query.fcgi?db=gene&cmd=Retrieve&dopt=full_report&list_uids=2635) | guanylate binding protein 3 |
| [Details](https://mirdb.org/cgi-bin/target_detail.cgi?targetID=2854257) | 905 | 70 | hsa-miR-20a-5p | [PEAK1](http://www.ncbi.nlm.nih.gov/entrez/query.fcgi?db=gene&cmd=Retrieve&dopt=full_report&list_uids=79834) | pseudopodium enriched atypical kinase 1 |
| [Details](https://mirdb.org/cgi-bin/target_detail.cgi?targetID=2854335) | 906 | 70 | hsa-miR-20a-5p | [SLC11A1](http://www.ncbi.nlm.nih.gov/entrez/query.fcgi?db=gene&cmd=Retrieve&dopt=full_report&list_uids=6556) | solute carrier family 11 member 1 |
| [Details](https://mirdb.org/cgi-bin/target_detail.cgi?targetID=2854339) | 907 | 70 | hsa-miR-20a-5p | [RRP15](http://www.ncbi.nlm.nih.gov/entrez/query.fcgi?db=gene&cmd=Retrieve&dopt=full_report&list_uids=51018) | ribosomal RNA processing 15 homolog |
| [Details](https://mirdb.org/cgi-bin/target_detail.cgi?targetID=2854362) | 908 | 70 | hsa-miR-20a-5p | [ZNF217](http://www.ncbi.nlm.nih.gov/entrez/query.fcgi?db=gene&cmd=Retrieve&dopt=full_report&list_uids=7764) | zinc finger protein 217 |
| [Details](https://mirdb.org/cgi-bin/target_detail.cgi?targetID=2854401) | 909 | 70 | hsa-miR-20a-5p | [MVK](http://www.ncbi.nlm.nih.gov/entrez/query.fcgi?db=gene&cmd=Retrieve&dopt=full_report&list_uids=4598) | mevalonate kinase |
| [Details](https://mirdb.org/cgi-bin/target_detail.cgi?targetID=2854417) | 910 | 70 | hsa-miR-20a-5p | [BCR](http://www.ncbi.nlm.nih.gov/entrez/query.fcgi?db=gene&cmd=Retrieve&dopt=full_report&list_uids=613) | BCR, RhoGEF and GTPase activating protein |
| [Details](https://mirdb.org/cgi-bin/target_detail.cgi?targetID=2854435) | 911 | 70 | hsa-miR-20a-5p | [RAB3B](http://www.ncbi.nlm.nih.gov/entrez/query.fcgi?db=gene&cmd=Retrieve&dopt=full_report&list_uids=5865) | RAB3B, member RAS oncogene family |
| [Details](https://mirdb.org/cgi-bin/target_detail.cgi?targetID=2854475) | 912 | 70 | hsa-miR-20a-5p | [TTPAL](http://www.ncbi.nlm.nih.gov/entrez/query.fcgi?db=gene&cmd=Retrieve&dopt=full_report&list_uids=79183) | alpha tocopherol transfer protein like |
| [Details](https://mirdb.org/cgi-bin/target_detail.cgi?targetID=2854684) | 913 | 70 | hsa-miR-20a-5p | [KPNA4](http://www.ncbi.nlm.nih.gov/entrez/query.fcgi?db=gene&cmd=Retrieve&dopt=full_report&list_uids=3840) | karyopherin subunit alpha 4 |
| [Details](https://mirdb.org/cgi-bin/target_detail.cgi?targetID=2854714) | 914 | 70 | hsa-miR-20a-5p | [SLC2A4](http://www.ncbi.nlm.nih.gov/entrez/query.fcgi?db=gene&cmd=Retrieve&dopt=full_report&list_uids=6517) | solute carrier family 2 member 4 |
| [Details](https://mirdb.org/cgi-bin/target_detail.cgi?targetID=2854790) | 915 | 70 | hsa-miR-20a-5p | [DCUN1D3](http://www.ncbi.nlm.nih.gov/entrez/query.fcgi?db=gene&cmd=Retrieve&dopt=full_report&list_uids=123879) | defective in cullin neddylation 1 domain containing 3 |
| [Details](https://mirdb.org/cgi-bin/target_detail.cgi?targetID=2854794) | 916 | 70 | hsa-miR-20a-5p | [VPS53](http://www.ncbi.nlm.nih.gov/entrez/query.fcgi?db=gene&cmd=Retrieve&dopt=full_report&list_uids=55275) | VPS53, GARP complex subunit |
| [Details](https://mirdb.org/cgi-bin/target_detail.cgi?targetID=2854834) | 917 | 70 | hsa-miR-20a-5p | [NR2E3](http://www.ncbi.nlm.nih.gov/entrez/query.fcgi?db=gene&cmd=Retrieve&dopt=full_report&list_uids=10002) | nuclear receptor subfamily 2 group E member 3 |
| [Details](https://mirdb.org/cgi-bin/target_detail.cgi?targetID=2854890) | 918 | 70 | hsa-miR-20a-5p | [CERS6](http://www.ncbi.nlm.nih.gov/entrez/query.fcgi?db=gene&cmd=Retrieve&dopt=full_report&list_uids=253782) | ceramide synthase 6 |
| [Details](https://mirdb.org/cgi-bin/target_detail.cgi?targetID=2854913) | 919 | 70 | hsa-miR-20a-5p | [CYB561D1](http://www.ncbi.nlm.nih.gov/entrez/query.fcgi?db=gene&cmd=Retrieve&dopt=full_report&list_uids=284613) | cytochrome b561 family member D1 |
| [Details](https://mirdb.org/cgi-bin/target_detail.cgi?targetID=2854958) | 920 | 70 | hsa-miR-20a-5p | [KIRREL1](http://www.ncbi.nlm.nih.gov/entrez/query.fcgi?db=gene&cmd=Retrieve&dopt=full_report&list_uids=55243) | kirre like nephrin family adhesion molecule 1 |
| [Details](https://mirdb.org/cgi-bin/target_detail.cgi?targetID=2855022) | 921 | 70 | hsa-miR-20a-5p | [NTRK2](http://www.ncbi.nlm.nih.gov/entrez/query.fcgi?db=gene&cmd=Retrieve&dopt=full_report&list_uids=4915) | neurotrophic receptor tyrosine kinase 2 |
| [Details](https://mirdb.org/cgi-bin/target_detail.cgi?targetID=2855036) | 922 | 70 | hsa-miR-20a-5p | [TIMP2](http://www.ncbi.nlm.nih.gov/entrez/query.fcgi?db=gene&cmd=Retrieve&dopt=full_report&list_uids=7077) | TIMP metallopeptidase inhibitor 2 |
| [Details](https://mirdb.org/cgi-bin/target_detail.cgi?targetID=2855115) | 923 | 70 | hsa-miR-20a-5p | [BUD23](http://www.ncbi.nlm.nih.gov/entrez/query.fcgi?db=gene&cmd=Retrieve&dopt=full_report&list_uids=114049) | BUD23, rRNA methyltransferase and ribosome maturation factor |
| [Details](https://mirdb.org/cgi-bin/target_detail.cgi?targetID=2855240) | 924 | 70 | hsa-miR-20a-5p | [RILPL1](http://www.ncbi.nlm.nih.gov/entrez/query.fcgi?db=gene&cmd=Retrieve&dopt=full_report&list_uids=353116) | Rab interacting lysosomal protein like 1 |
| [Details](https://mirdb.org/cgi-bin/target_detail.cgi?targetID=2855368) | 925 | 70 | hsa-miR-20a-5p | [CSDE1](http://www.ncbi.nlm.nih.gov/entrez/query.fcgi?db=gene&cmd=Retrieve&dopt=full_report&list_uids=7812) | cold shock domain containing E1 |
| [Details](https://mirdb.org/cgi-bin/target_detail.cgi?targetID=2855457) | 926 | 70 | hsa-miR-20a-5p | [MICOS10-NBL1](http://www.ncbi.nlm.nih.gov/entrez/query.fcgi?db=gene&cmd=Retrieve&dopt=full_report&list_uids=100532736) | MICOS10-NBL1 readthrough |
| [Details](https://mirdb.org/cgi-bin/target_detail.cgi?targetID=2854096) | 927 | 69 | hsa-miR-20a-5p | [TYW5](http://www.ncbi.nlm.nih.gov/entrez/query.fcgi?db=gene&cmd=Retrieve&dopt=full_report&list_uids=129450) | tRNA-yW synthesizing protein 5 |
| [Details](https://mirdb.org/cgi-bin/target_detail.cgi?targetID=2854207) | 928 | 69 | hsa-miR-20a-5p | [SLC25A36](http://www.ncbi.nlm.nih.gov/entrez/query.fcgi?db=gene&cmd=Retrieve&dopt=full_report&list_uids=55186) | solute carrier family 25 member 36 |
| [Details](https://mirdb.org/cgi-bin/target_detail.cgi?targetID=2854314) | 929 | 69 | hsa-miR-20a-5p | [CHD9](http://www.ncbi.nlm.nih.gov/entrez/query.fcgi?db=gene&cmd=Retrieve&dopt=full_report&list_uids=80205) | chromodomain helicase DNA binding protein 9 |
| [Details](https://mirdb.org/cgi-bin/target_detail.cgi?targetID=2854337) | 930 | 69 | hsa-miR-20a-5p | [YWHAQ](http://www.ncbi.nlm.nih.gov/entrez/query.fcgi?db=gene&cmd=Retrieve&dopt=full_report&list_uids=10971) | tyrosine 3-monooxygenase/tryptophan 5-monooxygenase activation protein theta |
| [Details](https://mirdb.org/cgi-bin/target_detail.cgi?targetID=2854395) | 931 | 69 | hsa-miR-20a-5p | [FURIN](http://www.ncbi.nlm.nih.gov/entrez/query.fcgi?db=gene&cmd=Retrieve&dopt=full_report&list_uids=5045) | furin, paired basic amino acid cleaving enzyme |
| [Details](https://mirdb.org/cgi-bin/target_detail.cgi?targetID=2854488) | 932 | 69 | hsa-miR-20a-5p | [SMAD7](http://www.ncbi.nlm.nih.gov/entrez/query.fcgi?db=gene&cmd=Retrieve&dopt=full_report&list_uids=4092) | SMAD family member 7 |
| [Details](https://mirdb.org/cgi-bin/target_detail.cgi?targetID=2854494) | 933 | 69 | hsa-miR-20a-5p | [TBC1D2](http://www.ncbi.nlm.nih.gov/entrez/query.fcgi?db=gene&cmd=Retrieve&dopt=full_report&list_uids=55357) | TBC1 domain family member 2 |
| [Details](https://mirdb.org/cgi-bin/target_detail.cgi?targetID=2854624) | 934 | 69 | hsa-miR-20a-5p | [KCNQ2](http://www.ncbi.nlm.nih.gov/entrez/query.fcgi?db=gene&cmd=Retrieve&dopt=full_report&list_uids=3785) | potassium voltage-gated channel subfamily Q member 2 |
| [Details](https://mirdb.org/cgi-bin/target_detail.cgi?targetID=2854639) | 935 | 69 | hsa-miR-20a-5p | [COIL](http://www.ncbi.nlm.nih.gov/entrez/query.fcgi?db=gene&cmd=Retrieve&dopt=full_report&list_uids=8161) | coilin |
| [Details](https://mirdb.org/cgi-bin/target_detail.cgi?targetID=2854702) | 936 | 69 | hsa-miR-20a-5p | [CARNMT1](http://www.ncbi.nlm.nih.gov/entrez/query.fcgi?db=gene&cmd=Retrieve&dopt=full_report&list_uids=138199) | carnosine N-methyltransferase 1 |
| [Details](https://mirdb.org/cgi-bin/target_detail.cgi?targetID=2854838) | 937 | 69 | hsa-miR-20a-5p | [SOBP](http://www.ncbi.nlm.nih.gov/entrez/query.fcgi?db=gene&cmd=Retrieve&dopt=full_report&list_uids=55084) | sine oculis binding protein homolog |
| [Details](https://mirdb.org/cgi-bin/target_detail.cgi?targetID=2854858) | 938 | 69 | hsa-miR-20a-5p | [INO80](http://www.ncbi.nlm.nih.gov/entrez/query.fcgi?db=gene&cmd=Retrieve&dopt=full_report&list_uids=54617) | INO80 complex subunit |
| [Details](https://mirdb.org/cgi-bin/target_detail.cgi?targetID=2854889) | 939 | 69 | hsa-miR-20a-5p | [HDAC4](http://www.ncbi.nlm.nih.gov/entrez/query.fcgi?db=gene&cmd=Retrieve&dopt=full_report&list_uids=9759) | histone deacetylase 4 |
| [Details](https://mirdb.org/cgi-bin/target_detail.cgi?targetID=2854905) | 940 | 69 | hsa-miR-20a-5p | [SNX21](http://www.ncbi.nlm.nih.gov/entrez/query.fcgi?db=gene&cmd=Retrieve&dopt=full_report&list_uids=90203) | sorting nexin family member 21 |
| [Details](https://mirdb.org/cgi-bin/target_detail.cgi?targetID=2854983) | 941 | 69 | hsa-miR-20a-5p | [CCL1](http://www.ncbi.nlm.nih.gov/entrez/query.fcgi?db=gene&cmd=Retrieve&dopt=full_report&list_uids=6346) | C-C motif chemokine ligand 1 |
| [Details](https://mirdb.org/cgi-bin/target_detail.cgi?targetID=2854990) | 942 | 69 | hsa-miR-20a-5p | [GMCL1](http://www.ncbi.nlm.nih.gov/entrez/query.fcgi?db=gene&cmd=Retrieve&dopt=full_report&list_uids=64395) | germ cell-less, spermatogenesis associated 1 |
| [Details](https://mirdb.org/cgi-bin/target_detail.cgi?targetID=2855005) | 943 | 69 | hsa-miR-20a-5p | [LHFPL2](http://www.ncbi.nlm.nih.gov/entrez/query.fcgi?db=gene&cmd=Retrieve&dopt=full_report&list_uids=10184) | LHFPL tetraspan subfamily member 2 |
| [Details](https://mirdb.org/cgi-bin/target_detail.cgi?targetID=2855014) | 944 | 69 | hsa-miR-20a-5p | [SUSD1](http://www.ncbi.nlm.nih.gov/entrez/query.fcgi?db=gene&cmd=Retrieve&dopt=full_report&list_uids=64420) | sushi domain containing 1 |
| [Details](https://mirdb.org/cgi-bin/target_detail.cgi?targetID=2855090) | 945 | 69 | hsa-miR-20a-5p | [THBS2](http://www.ncbi.nlm.nih.gov/entrez/query.fcgi?db=gene&cmd=Retrieve&dopt=full_report&list_uids=7058) | thrombospondin 2 |
| [Details](https://mirdb.org/cgi-bin/target_detail.cgi?targetID=2855125) | 946 | 69 | hsa-miR-20a-5p | [GCA](http://www.ncbi.nlm.nih.gov/entrez/query.fcgi?db=gene&cmd=Retrieve&dopt=full_report&list_uids=25801) | grancalcin |
| [Details](https://mirdb.org/cgi-bin/target_detail.cgi?targetID=2855151) | 947 | 69 | hsa-miR-20a-5p | [LRRC20](http://www.ncbi.nlm.nih.gov/entrez/query.fcgi?db=gene&cmd=Retrieve&dopt=full_report&list_uids=55222) | leucine rich repeat containing 20 |
| [Details](https://mirdb.org/cgi-bin/target_detail.cgi?targetID=2855166) | 948 | 69 | hsa-miR-20a-5p | [SERPINB8](http://www.ncbi.nlm.nih.gov/entrez/query.fcgi?db=gene&cmd=Retrieve&dopt=full_report&list_uids=5271) | serpin family B member 8 |
| [Details](https://mirdb.org/cgi-bin/target_detail.cgi?targetID=2855403) | 949 | 69 | hsa-miR-20a-5p | [ISM1](http://www.ncbi.nlm.nih.gov/entrez/query.fcgi?db=gene&cmd=Retrieve&dopt=full_report&list_uids=140862) | isthmin 1 |
| [Details](https://mirdb.org/cgi-bin/target_detail.cgi?targetID=2854172) | 950 | 68 | hsa-miR-20a-5p | [EFCAB1](http://www.ncbi.nlm.nih.gov/entrez/query.fcgi?db=gene&cmd=Retrieve&dopt=full_report&list_uids=79645) | EF-hand calcium binding domain 1 |
| [Details](https://mirdb.org/cgi-bin/target_detail.cgi?targetID=2854222) | 951 | 68 | hsa-miR-20a-5p | [CDCA7](http://www.ncbi.nlm.nih.gov/entrez/query.fcgi?db=gene&cmd=Retrieve&dopt=full_report&list_uids=83879) | cell division cycle associated 7 |
| [Details](https://mirdb.org/cgi-bin/target_detail.cgi?targetID=2854253) | 952 | 68 | hsa-miR-20a-5p | [STK11IP](http://www.ncbi.nlm.nih.gov/entrez/query.fcgi?db=gene&cmd=Retrieve&dopt=full_report&list_uids=114790) | serine/threonine kinase 11 interacting protein |
| [Details](https://mirdb.org/cgi-bin/target_detail.cgi?targetID=2854420) | 953 | 68 | hsa-miR-20a-5p | [PUDP](http://www.ncbi.nlm.nih.gov/entrez/query.fcgi?db=gene&cmd=Retrieve&dopt=full_report&list_uids=8226) | pseudouridine 5'-phosphatase |
| [Details](https://mirdb.org/cgi-bin/target_detail.cgi?targetID=2854583) | 954 | 68 | hsa-miR-20a-5p | [RIMBP2](http://www.ncbi.nlm.nih.gov/entrez/query.fcgi?db=gene&cmd=Retrieve&dopt=full_report&list_uids=23504) | RIMS binding protein 2 |
| [Details](https://mirdb.org/cgi-bin/target_detail.cgi?targetID=2854751) | 955 | 68 | hsa-miR-20a-5p | [C2CD4A](http://www.ncbi.nlm.nih.gov/entrez/query.fcgi?db=gene&cmd=Retrieve&dopt=full_report&list_uids=145741) | C2 calcium dependent domain containing 4A |
| [Details](https://mirdb.org/cgi-bin/target_detail.cgi?targetID=2854780) | 956 | 68 | hsa-miR-20a-5p | [SMAD1](http://www.ncbi.nlm.nih.gov/entrez/query.fcgi?db=gene&cmd=Retrieve&dopt=full_report&list_uids=4086) | SMAD family member 1 |
| [Details](https://mirdb.org/cgi-bin/target_detail.cgi?targetID=2854802) | 957 | 68 | hsa-miR-20a-5p | [GTPBP10](http://www.ncbi.nlm.nih.gov/entrez/query.fcgi?db=gene&cmd=Retrieve&dopt=full_report&list_uids=85865) | GTP binding protein 10 |
| [Details](https://mirdb.org/cgi-bin/target_detail.cgi?targetID=2854882) | 958 | 68 | hsa-miR-20a-5p | [COL4A1](http://www.ncbi.nlm.nih.gov/entrez/query.fcgi?db=gene&cmd=Retrieve&dopt=full_report&list_uids=1282) | collagen type IV alpha 1 chain |
| [Details](https://mirdb.org/cgi-bin/target_detail.cgi?targetID=2854896) | 959 | 68 | hsa-miR-20a-5p | [SFR1](http://www.ncbi.nlm.nih.gov/entrez/query.fcgi?db=gene&cmd=Retrieve&dopt=full_report&list_uids=119392) | SWI5 dependent homologous recombination repair protein 1 |
| [Details](https://mirdb.org/cgi-bin/target_detail.cgi?targetID=2855059) | 960 | 68 | hsa-miR-20a-5p | [NEK9](http://www.ncbi.nlm.nih.gov/entrez/query.fcgi?db=gene&cmd=Retrieve&dopt=full_report&list_uids=91754) | NIMA related kinase 9 |
| [Details](https://mirdb.org/cgi-bin/target_detail.cgi?targetID=2855093) | 961 | 68 | hsa-miR-20a-5p | [SRGAP3](http://www.ncbi.nlm.nih.gov/entrez/query.fcgi?db=gene&cmd=Retrieve&dopt=full_report&list_uids=9901) | SLIT-ROBO Rho GTPase activating protein 3 |
| [Details](https://mirdb.org/cgi-bin/target_detail.cgi?targetID=2855162) | 962 | 68 | hsa-miR-20a-5p | [RSBN1](http://www.ncbi.nlm.nih.gov/entrez/query.fcgi?db=gene&cmd=Retrieve&dopt=full_report&list_uids=54665) | round spermatid basic protein 1 |
| [Details](https://mirdb.org/cgi-bin/target_detail.cgi?targetID=2855219) | 963 | 68 | hsa-miR-20a-5p | [CBFB](http://www.ncbi.nlm.nih.gov/entrez/query.fcgi?db=gene&cmd=Retrieve&dopt=full_report&list_uids=865) | core-binding factor subunit beta |
| [Details](https://mirdb.org/cgi-bin/target_detail.cgi?targetID=2855361) | 964 | 68 | hsa-miR-20a-5p | [CYP26B1](http://www.ncbi.nlm.nih.gov/entrez/query.fcgi?db=gene&cmd=Retrieve&dopt=full_report&list_uids=56603) | cytochrome P450 family 26 subfamily B member 1 |
| [Details](https://mirdb.org/cgi-bin/target_detail.cgi?targetID=2855402) | 965 | 68 | hsa-miR-20a-5p | [DGKH](http://www.ncbi.nlm.nih.gov/entrez/query.fcgi?db=gene&cmd=Retrieve&dopt=full_report&list_uids=160851) | diacylglycerol kinase eta |
| [Details](https://mirdb.org/cgi-bin/target_detail.cgi?targetID=2855448) | 966 | 68 | hsa-miR-20a-5p | [GOLPH3](http://www.ncbi.nlm.nih.gov/entrez/query.fcgi?db=gene&cmd=Retrieve&dopt=full_report&list_uids=64083) | golgi phosphoprotein 3 |
| [Details](https://mirdb.org/cgi-bin/target_detail.cgi?targetID=2854104) | 967 | 67 | hsa-miR-20a-5p | [KIAA1147](http://www.ncbi.nlm.nih.gov/entrez/query.fcgi?db=gene&cmd=Retrieve&dopt=full_report&list_uids=57189) | KIAA1147 |
| [Details](https://mirdb.org/cgi-bin/target_detail.cgi?targetID=2854297) | 968 | 67 | hsa-miR-20a-5p | [RASSF2](http://www.ncbi.nlm.nih.gov/entrez/query.fcgi?db=gene&cmd=Retrieve&dopt=full_report&list_uids=9770) | Ras association domain family member 2 |
| [Details](https://mirdb.org/cgi-bin/target_detail.cgi?targetID=2854309) | 969 | 67 | hsa-miR-20a-5p | [GIT2](http://www.ncbi.nlm.nih.gov/entrez/query.fcgi?db=gene&cmd=Retrieve&dopt=full_report&list_uids=9815) | GIT ArfGAP 2 |
| [Details](https://mirdb.org/cgi-bin/target_detail.cgi?targetID=2854315) | 970 | 67 | hsa-miR-20a-5p | [PRDM10](http://www.ncbi.nlm.nih.gov/entrez/query.fcgi?db=gene&cmd=Retrieve&dopt=full_report&list_uids=56980) | PR/SET domain 10 |
| [Details](https://mirdb.org/cgi-bin/target_detail.cgi?targetID=2854334) | 971 | 67 | hsa-miR-20a-5p | [TMTC1](http://www.ncbi.nlm.nih.gov/entrez/query.fcgi?db=gene&cmd=Retrieve&dopt=full_report&list_uids=83857) | transmembrane and tetratricopeptide repeat containing 1 |
| [Details](https://mirdb.org/cgi-bin/target_detail.cgi?targetID=2854372) | 972 | 67 | hsa-miR-20a-5p | [IGF2BP1](http://www.ncbi.nlm.nih.gov/entrez/query.fcgi?db=gene&cmd=Retrieve&dopt=full_report&list_uids=10642) | insulin like growth factor 2 mRNA binding protein 1 |
| [Details](https://mirdb.org/cgi-bin/target_detail.cgi?targetID=2854397) | 973 | 67 | hsa-miR-20a-5p | [MMAA](http://www.ncbi.nlm.nih.gov/entrez/query.fcgi?db=gene&cmd=Retrieve&dopt=full_report&list_uids=166785) | metabolism of cobalamin associated A |
| [Details](https://mirdb.org/cgi-bin/target_detail.cgi?targetID=2854457) | 974 | 67 | hsa-miR-20a-5p | [RAB11FIP4](http://www.ncbi.nlm.nih.gov/entrez/query.fcgi?db=gene&cmd=Retrieve&dopt=full_report&list_uids=84440) | RAB11 family interacting protein 4 |
| [Details](https://mirdb.org/cgi-bin/target_detail.cgi?targetID=2854570) | 975 | 67 | hsa-miR-20a-5p | [ADAMTS5](http://www.ncbi.nlm.nih.gov/entrez/query.fcgi?db=gene&cmd=Retrieve&dopt=full_report&list_uids=11096) | ADAM metallopeptidase with thrombospondin type 1 motif 5 |
| [Details](https://mirdb.org/cgi-bin/target_detail.cgi?targetID=2854634) | 976 | 67 | hsa-miR-20a-5p | [CYBB](http://www.ncbi.nlm.nih.gov/entrez/query.fcgi?db=gene&cmd=Retrieve&dopt=full_report&list_uids=1536) | cytochrome b-245 beta chain |
| [Details](https://mirdb.org/cgi-bin/target_detail.cgi?targetID=2854661) | 977 | 67 | hsa-miR-20a-5p | [TIGAR](http://www.ncbi.nlm.nih.gov/entrez/query.fcgi?db=gene&cmd=Retrieve&dopt=full_report&list_uids=57103) | TP53 induced glycolysis regulatory phosphatase |
| [Details](https://mirdb.org/cgi-bin/target_detail.cgi?targetID=2854696) | 978 | 67 | hsa-miR-20a-5p | [ELOA](http://www.ncbi.nlm.nih.gov/entrez/query.fcgi?db=gene&cmd=Retrieve&dopt=full_report&list_uids=6924) | elongin A |
| [Details](https://mirdb.org/cgi-bin/target_detail.cgi?targetID=2854736) | 979 | 67 | hsa-miR-20a-5p | [XIRP2](http://www.ncbi.nlm.nih.gov/entrez/query.fcgi?db=gene&cmd=Retrieve&dopt=full_report&list_uids=129446) | xin actin binding repeat containing 2 |
| [Details](https://mirdb.org/cgi-bin/target_detail.cgi?targetID=2854860) | 980 | 67 | hsa-miR-20a-5p | [LPIN1](http://www.ncbi.nlm.nih.gov/entrez/query.fcgi?db=gene&cmd=Retrieve&dopt=full_report&list_uids=23175) | lipin 1 |
| [Details](https://mirdb.org/cgi-bin/target_detail.cgi?targetID=2854895) | 981 | 67 | hsa-miR-20a-5p | [COX7A2](http://www.ncbi.nlm.nih.gov/entrez/query.fcgi?db=gene&cmd=Retrieve&dopt=full_report&list_uids=1347) | cytochrome c oxidase subunit 7A2 |
| [Details](https://mirdb.org/cgi-bin/target_detail.cgi?targetID=2854907) | 982 | 67 | hsa-miR-20a-5p | [MCC](http://www.ncbi.nlm.nih.gov/entrez/query.fcgi?db=gene&cmd=Retrieve&dopt=full_report&list_uids=4163) | MCC, WNT signaling pathway regulator |
| [Details](https://mirdb.org/cgi-bin/target_detail.cgi?targetID=2854962) | 983 | 67 | hsa-miR-20a-5p | [DNM1L](http://www.ncbi.nlm.nih.gov/entrez/query.fcgi?db=gene&cmd=Retrieve&dopt=full_report&list_uids=10059) | dynamin 1 like |
| [Details](https://mirdb.org/cgi-bin/target_detail.cgi?targetID=2855013) | 984 | 67 | hsa-miR-20a-5p | [SINHCAF](http://www.ncbi.nlm.nih.gov/entrez/query.fcgi?db=gene&cmd=Retrieve&dopt=full_report&list_uids=58516) | SIN3-HDAC complex associated factor |
| [Details](https://mirdb.org/cgi-bin/target_detail.cgi?targetID=2855250) | 985 | 67 | hsa-miR-20a-5p | [MINDY2](http://www.ncbi.nlm.nih.gov/entrez/query.fcgi?db=gene&cmd=Retrieve&dopt=full_report&list_uids=54629) | MINDY lysine 48 deubiquitinase 2 |
| [Details](https://mirdb.org/cgi-bin/target_detail.cgi?targetID=2855271) | 986 | 67 | hsa-miR-20a-5p | [ZRANB1](http://www.ncbi.nlm.nih.gov/entrez/query.fcgi?db=gene&cmd=Retrieve&dopt=full_report&list_uids=54764) | zinc finger RANBP2-type containing 1 |
| [Details](https://mirdb.org/cgi-bin/target_detail.cgi?targetID=2855335) | 987 | 67 | hsa-miR-20a-5p | [TNRC6B](http://www.ncbi.nlm.nih.gov/entrez/query.fcgi?db=gene&cmd=Retrieve&dopt=full_report&list_uids=23112) | trinucleotide repeat containing 6B |
| [Details](https://mirdb.org/cgi-bin/target_detail.cgi?targetID=2855414) | 988 | 67 | hsa-miR-20a-5p | [MKLN1](http://www.ncbi.nlm.nih.gov/entrez/query.fcgi?db=gene&cmd=Retrieve&dopt=full_report&list_uids=4289) | muskelin 1 |
| [Details](https://mirdb.org/cgi-bin/target_detail.cgi?targetID=2854153) | 989 | 66 | hsa-miR-20a-5p | [CCSER1](http://www.ncbi.nlm.nih.gov/entrez/query.fcgi?db=gene&cmd=Retrieve&dopt=full_report&list_uids=401145) | coiled-coil serine rich protein 1 |
| [Details](https://mirdb.org/cgi-bin/target_detail.cgi?targetID=2854244) | 990 | 66 | hsa-miR-20a-5p | [PTPRT](http://www.ncbi.nlm.nih.gov/entrez/query.fcgi?db=gene&cmd=Retrieve&dopt=full_report&list_uids=11122) | protein tyrosine phosphatase, receptor type T |
| [Details](https://mirdb.org/cgi-bin/target_detail.cgi?targetID=2854347) | 991 | 66 | hsa-miR-20a-5p | [KCNJ8](http://www.ncbi.nlm.nih.gov/entrez/query.fcgi?db=gene&cmd=Retrieve&dopt=full_report&list_uids=3764) | potassium voltage-gated channel subfamily J member 8 |
| [Details](https://mirdb.org/cgi-bin/target_detail.cgi?targetID=2854430) | 992 | 66 | hsa-miR-20a-5p | [GABRA1](http://www.ncbi.nlm.nih.gov/entrez/query.fcgi?db=gene&cmd=Retrieve&dopt=full_report&list_uids=2554) | gamma-aminobutyric acid type A receptor alpha1 subunit |
| [Details](https://mirdb.org/cgi-bin/target_detail.cgi?targetID=2854476) | 993 | 66 | hsa-miR-20a-5p | [AK4](http://www.ncbi.nlm.nih.gov/entrez/query.fcgi?db=gene&cmd=Retrieve&dopt=full_report&list_uids=205) | adenylate kinase 4 |
| [Details](https://mirdb.org/cgi-bin/target_detail.cgi?targetID=2854535) | 994 | 66 | hsa-miR-20a-5p | [OGA](http://www.ncbi.nlm.nih.gov/entrez/query.fcgi?db=gene&cmd=Retrieve&dopt=full_report&list_uids=10724) | O-GlcNAcase |
| [Details](https://mirdb.org/cgi-bin/target_detail.cgi?targetID=2854552) | 995 | 66 | hsa-miR-20a-5p | [EPHB4](http://www.ncbi.nlm.nih.gov/entrez/query.fcgi?db=gene&cmd=Retrieve&dopt=full_report&list_uids=2050) | EPH receptor B4 |
| [Details](https://mirdb.org/cgi-bin/target_detail.cgi?targetID=2854646) | 996 | 66 | hsa-miR-20a-5p | [PBXIP1](http://www.ncbi.nlm.nih.gov/entrez/query.fcgi?db=gene&cmd=Retrieve&dopt=full_report&list_uids=57326) | PBX homeobox interacting protein 1 |
| [Details](https://mirdb.org/cgi-bin/target_detail.cgi?targetID=2854666) | 997 | 66 | hsa-miR-20a-5p | [PPP1R12B](http://www.ncbi.nlm.nih.gov/entrez/query.fcgi?db=gene&cmd=Retrieve&dopt=full_report&list_uids=4660) | protein phosphatase 1 regulatory subunit 12B |
| [Details](https://mirdb.org/cgi-bin/target_detail.cgi?targetID=2854690) | 998 | 66 | hsa-miR-20a-5p | [YTHDF3](http://www.ncbi.nlm.nih.gov/entrez/query.fcgi?db=gene&cmd=Retrieve&dopt=full_report&list_uids=253943) | YTH N6-methyladenosine RNA binding protein 3 |
| [Details](https://mirdb.org/cgi-bin/target_detail.cgi?targetID=2854704) | 999 | 66 | hsa-miR-20a-5p | [PRRG4](http://www.ncbi.nlm.nih.gov/entrez/query.fcgi?db=gene&cmd=Retrieve&dopt=full_report&list_uids=79056) | proline rich and Gla domain 4 |
| [Details](https://mirdb.org/cgi-bin/target_detail.cgi?targetID=2854728) | 1000 | 66 | hsa-miR-20a-5p | [NFE2L2](http://www.ncbi.nlm.nih.gov/entrez/query.fcgi?db=gene&cmd=Retrieve&dopt=full_report&list_uids=4780) | nuclear factor, erythroid 2 like 2 |
| [Details](https://mirdb.org/cgi-bin/target_detail.cgi?targetID=2854771) | 1001 | 66 | hsa-miR-20a-5p | [SYT16](http://www.ncbi.nlm.nih.gov/entrez/query.fcgi?db=gene&cmd=Retrieve&dopt=full_report&list_uids=83851) | synaptotagmin 16 |
| [Details](https://mirdb.org/cgi-bin/target_detail.cgi?targetID=2854819) | 1002 | 66 | hsa-miR-20a-5p | [VEGFA](http://www.ncbi.nlm.nih.gov/entrez/query.fcgi?db=gene&cmd=Retrieve&dopt=full_report&list_uids=7422) | vascular endothelial growth factor A |
| [Details](https://mirdb.org/cgi-bin/target_detail.cgi?targetID=2854840) | 1003 | 66 | hsa-miR-20a-5p | [COQ2](http://www.ncbi.nlm.nih.gov/entrez/query.fcgi?db=gene&cmd=Retrieve&dopt=full_report&list_uids=27235) | coenzyme Q2, polyprenyltransferase |
| [Details](https://mirdb.org/cgi-bin/target_detail.cgi?targetID=2854845) | 1004 | 66 | hsa-miR-20a-5p | [SLC6A9](http://www.ncbi.nlm.nih.gov/entrez/query.fcgi?db=gene&cmd=Retrieve&dopt=full_report&list_uids=6536) | solute carrier family 6 member 9 |
| [Details](https://mirdb.org/cgi-bin/target_detail.cgi?targetID=2854861) | 1005 | 66 | hsa-miR-20a-5p | [MYF5](http://www.ncbi.nlm.nih.gov/entrez/query.fcgi?db=gene&cmd=Retrieve&dopt=full_report&list_uids=4617) | myogenic factor 5 |
| [Details](https://mirdb.org/cgi-bin/target_detail.cgi?targetID=2854977) | 1006 | 66 | hsa-miR-20a-5p | [WDR33](http://www.ncbi.nlm.nih.gov/entrez/query.fcgi?db=gene&cmd=Retrieve&dopt=full_report&list_uids=55339) | WD repeat domain 33 |
| [Details](https://mirdb.org/cgi-bin/target_detail.cgi?targetID=2854996) | 1007 | 66 | hsa-miR-20a-5p | [PXDN](http://www.ncbi.nlm.nih.gov/entrez/query.fcgi?db=gene&cmd=Retrieve&dopt=full_report&list_uids=7837) | peroxidasin |
| [Details](https://mirdb.org/cgi-bin/target_detail.cgi?targetID=2855008) | 1008 | 66 | hsa-miR-20a-5p | [METAP1](http://www.ncbi.nlm.nih.gov/entrez/query.fcgi?db=gene&cmd=Retrieve&dopt=full_report&list_uids=23173) | methionyl aminopeptidase 1 |
| [Details](https://mirdb.org/cgi-bin/target_detail.cgi?targetID=2855047) | 1009 | 66 | hsa-miR-20a-5p | [NETO2](http://www.ncbi.nlm.nih.gov/entrez/query.fcgi?db=gene&cmd=Retrieve&dopt=full_report&list_uids=81831) | neuropilin and tolloid like 2 |
| [Details](https://mirdb.org/cgi-bin/target_detail.cgi?targetID=2855298) | 1010 | 66 | hsa-miR-20a-5p | [MRPL43](http://www.ncbi.nlm.nih.gov/entrez/query.fcgi?db=gene&cmd=Retrieve&dopt=full_report&list_uids=84545) | mitochondrial ribosomal protein L43 |
| [Details](https://mirdb.org/cgi-bin/target_detail.cgi?targetID=2855324) | 1011 | 66 | hsa-miR-20a-5p | [ASXL3](http://www.ncbi.nlm.nih.gov/entrez/query.fcgi?db=gene&cmd=Retrieve&dopt=full_report&list_uids=80816) | ASXL transcriptional regulator 3 |
| [Details](https://mirdb.org/cgi-bin/target_detail.cgi?targetID=2855405) | 1012 | 66 | hsa-miR-20a-5p | [PPP6R3](http://www.ncbi.nlm.nih.gov/entrez/query.fcgi?db=gene&cmd=Retrieve&dopt=full_report&list_uids=55291) | protein phosphatase 6 regulatory subunit 3 |
| [Details](https://mirdb.org/cgi-bin/target_detail.cgi?targetID=2854087) | 1013 | 65 | hsa-miR-20a-5p | [ZNF532](http://www.ncbi.nlm.nih.gov/entrez/query.fcgi?db=gene&cmd=Retrieve&dopt=full_report&list_uids=55205) | zinc finger protein 532 |
| [Details](https://mirdb.org/cgi-bin/target_detail.cgi?targetID=2854120) | 1014 | 65 | hsa-miR-20a-5p | [TNFSF11](http://www.ncbi.nlm.nih.gov/entrez/query.fcgi?db=gene&cmd=Retrieve&dopt=full_report&list_uids=8600) | TNF superfamily member 11 |
| [Details](https://mirdb.org/cgi-bin/target_detail.cgi?targetID=2854136) | 1015 | 65 | hsa-miR-20a-5p | [AMPD3](http://www.ncbi.nlm.nih.gov/entrez/query.fcgi?db=gene&cmd=Retrieve&dopt=full_report&list_uids=272) | adenosine monophosphate deaminase 3 |
| [Details](https://mirdb.org/cgi-bin/target_detail.cgi?targetID=2854201) | 1016 | 65 | hsa-miR-20a-5p | [PKMYT1](http://www.ncbi.nlm.nih.gov/entrez/query.fcgi?db=gene&cmd=Retrieve&dopt=full_report&list_uids=9088) | protein kinase, membrane associated tyrosine/threonine 1 |
| [Details](https://mirdb.org/cgi-bin/target_detail.cgi?targetID=2854270) | 1017 | 65 | hsa-miR-20a-5p | [WNK1](http://www.ncbi.nlm.nih.gov/entrez/query.fcgi?db=gene&cmd=Retrieve&dopt=full_report&list_uids=65125) | WNK lysine deficient protein kinase 1 |
| [Details](https://mirdb.org/cgi-bin/target_detail.cgi?targetID=2854301) | 1018 | 65 | hsa-miR-20a-5p | [GPAM](http://www.ncbi.nlm.nih.gov/entrez/query.fcgi?db=gene&cmd=Retrieve&dopt=full_report&list_uids=57678) | glycerol-3-phosphate acyltransferase, mitochondrial |
| [Details](https://mirdb.org/cgi-bin/target_detail.cgi?targetID=2854330) | 1019 | 65 | hsa-miR-20a-5p | [PANK3](http://www.ncbi.nlm.nih.gov/entrez/query.fcgi?db=gene&cmd=Retrieve&dopt=full_report&list_uids=79646) | pantothenate kinase 3 |
| [Details](https://mirdb.org/cgi-bin/target_detail.cgi?targetID=2854367) | 1020 | 65 | hsa-miR-20a-5p | [SLC25A27](http://www.ncbi.nlm.nih.gov/entrez/query.fcgi?db=gene&cmd=Retrieve&dopt=full_report&list_uids=9481) | solute carrier family 25 member 27 |
| [Details](https://mirdb.org/cgi-bin/target_detail.cgi?targetID=2854412) | 1021 | 65 | hsa-miR-20a-5p | [BBIP1](http://www.ncbi.nlm.nih.gov/entrez/query.fcgi?db=gene&cmd=Retrieve&dopt=full_report&list_uids=92482) | BBSome interacting protein 1 |
| [Details](https://mirdb.org/cgi-bin/target_detail.cgi?targetID=2854480) | 1022 | 65 | hsa-miR-20a-5p | [ARL1](http://www.ncbi.nlm.nih.gov/entrez/query.fcgi?db=gene&cmd=Retrieve&dopt=full_report&list_uids=400) | ADP ribosylation factor like GTPase 1 |
| [Details](https://mirdb.org/cgi-bin/target_detail.cgi?targetID=2854524) | 1023 | 65 | hsa-miR-20a-5p | [DMRTA2](http://www.ncbi.nlm.nih.gov/entrez/query.fcgi?db=gene&cmd=Retrieve&dopt=full_report&list_uids=63950) | DMRT like family A2 |
| [Details](https://mirdb.org/cgi-bin/target_detail.cgi?targetID=2854554) | 1024 | 65 | hsa-miR-20a-5p | [RAD17](http://www.ncbi.nlm.nih.gov/entrez/query.fcgi?db=gene&cmd=Retrieve&dopt=full_report&list_uids=5884) | RAD17 checkpoint clamp loader component |
| [Details](https://mirdb.org/cgi-bin/target_detail.cgi?targetID=2854601) | 1025 | 65 | hsa-miR-20a-5p | [NOL4](http://www.ncbi.nlm.nih.gov/entrez/query.fcgi?db=gene&cmd=Retrieve&dopt=full_report&list_uids=8715) | nucleolar protein 4 |
| [Details](https://mirdb.org/cgi-bin/target_detail.cgi?targetID=2854673) | 1026 | 65 | hsa-miR-20a-5p | [HESX1](http://www.ncbi.nlm.nih.gov/entrez/query.fcgi?db=gene&cmd=Retrieve&dopt=full_report&list_uids=8820) | HESX homeobox 1 |
| [Details](https://mirdb.org/cgi-bin/target_detail.cgi?targetID=2854757) | 1027 | 65 | hsa-miR-20a-5p | [REL](http://www.ncbi.nlm.nih.gov/entrez/query.fcgi?db=gene&cmd=Retrieve&dopt=full_report&list_uids=5966) | REL proto-oncogene, NF-kB subunit |
| [Details](https://mirdb.org/cgi-bin/target_detail.cgi?targetID=2854853) | 1028 | 65 | hsa-miR-20a-5p | [TBX3](http://www.ncbi.nlm.nih.gov/entrez/query.fcgi?db=gene&cmd=Retrieve&dopt=full_report&list_uids=6926) | T-box 3 |
| [Details](https://mirdb.org/cgi-bin/target_detail.cgi?targetID=2854888) | 1029 | 65 | hsa-miR-20a-5p | [CAMK2D](http://www.ncbi.nlm.nih.gov/entrez/query.fcgi?db=gene&cmd=Retrieve&dopt=full_report&list_uids=817) | calcium/calmodulin dependent protein kinase II delta |
| [Details](https://mirdb.org/cgi-bin/target_detail.cgi?targetID=2854948) | 1030 | 65 | hsa-miR-20a-5p | [ARHGAP24](http://www.ncbi.nlm.nih.gov/entrez/query.fcgi?db=gene&cmd=Retrieve&dopt=full_report&list_uids=83478) | Rho GTPase activating protein 24 |
| [Details](https://mirdb.org/cgi-bin/target_detail.cgi?targetID=2854954) | 1031 | 65 | hsa-miR-20a-5p | [COA1](http://www.ncbi.nlm.nih.gov/entrez/query.fcgi?db=gene&cmd=Retrieve&dopt=full_report&list_uids=55744) | cytochrome c oxidase assembly factor 1 homolog |
| [Details](https://mirdb.org/cgi-bin/target_detail.cgi?targetID=2855040) | 1032 | 65 | hsa-miR-20a-5p | [TRAPPC10](http://www.ncbi.nlm.nih.gov/entrez/query.fcgi?db=gene&cmd=Retrieve&dopt=full_report&list_uids=7109) | trafficking protein particle complex 10 |
| [Details](https://mirdb.org/cgi-bin/target_detail.cgi?targetID=2855102) | 1033 | 65 | hsa-miR-20a-5p | [GALNT10](http://www.ncbi.nlm.nih.gov/entrez/query.fcgi?db=gene&cmd=Retrieve&dopt=full_report&list_uids=55568) | polypeptide N-acetylgalactosaminyltransferase 10 |
| [Details](https://mirdb.org/cgi-bin/target_detail.cgi?targetID=2855131) | 1034 | 65 | hsa-miR-20a-5p | [IPMK](http://www.ncbi.nlm.nih.gov/entrez/query.fcgi?db=gene&cmd=Retrieve&dopt=full_report&list_uids=253430) | inositol polyphosphate multikinase |
| [Details](https://mirdb.org/cgi-bin/target_detail.cgi?targetID=2855285) | 1035 | 65 | hsa-miR-20a-5p | [PKNOX1](http://www.ncbi.nlm.nih.gov/entrez/query.fcgi?db=gene&cmd=Retrieve&dopt=full_report&list_uids=5316) | PBX/knotted 1 homeobox 1 |
| [Details](https://mirdb.org/cgi-bin/target_detail.cgi?targetID=2855315) | 1036 | 65 | hsa-miR-20a-5p | [CDC25A](http://www.ncbi.nlm.nih.gov/entrez/query.fcgi?db=gene&cmd=Retrieve&dopt=full_report&list_uids=993) | cell division cycle 25A |
| [Details](https://mirdb.org/cgi-bin/target_detail.cgi?targetID=2855365) | 1037 | 65 | hsa-miR-20a-5p | [PPP2R2A](http://www.ncbi.nlm.nih.gov/entrez/query.fcgi?db=gene&cmd=Retrieve&dopt=full_report&list_uids=5520) | protein phosphatase 2 regulatory subunit Balpha |
| [Details](https://mirdb.org/cgi-bin/target_detail.cgi?targetID=2855395) | 1038 | 65 | hsa-miR-20a-5p | [TMEM9B](http://www.ncbi.nlm.nih.gov/entrez/query.fcgi?db=gene&cmd=Retrieve&dopt=full_report&list_uids=56674) | TMEM9 domain family member B |
| [Details](https://mirdb.org/cgi-bin/target_detail.cgi?targetID=2854212) | 1039 | 64 | hsa-miR-20a-5p | [DCTN6](http://www.ncbi.nlm.nih.gov/entrez/query.fcgi?db=gene&cmd=Retrieve&dopt=full_report&list_uids=10671) | dynactin subunit 6 |
| [Details](https://mirdb.org/cgi-bin/target_detail.cgi?targetID=2854221) | 1040 | 64 | hsa-miR-20a-5p | [KMT2C](http://www.ncbi.nlm.nih.gov/entrez/query.fcgi?db=gene&cmd=Retrieve&dopt=full_report&list_uids=58508) | lysine methyltransferase 2C |
| [Details](https://mirdb.org/cgi-bin/target_detail.cgi?targetID=2854259) | 1041 | 64 | hsa-miR-20a-5p | [WDCP](http://www.ncbi.nlm.nih.gov/entrez/query.fcgi?db=gene&cmd=Retrieve&dopt=full_report&list_uids=80304) | WD repeat and coiled coil containing |
| [Details](https://mirdb.org/cgi-bin/target_detail.cgi?targetID=2854271) | 1042 | 64 | hsa-miR-20a-5p | [KIAA1671](http://www.ncbi.nlm.nih.gov/entrez/query.fcgi?db=gene&cmd=Retrieve&dopt=full_report&list_uids=85379) | KIAA1671 |
| [Details](https://mirdb.org/cgi-bin/target_detail.cgi?targetID=2854448) | 1043 | 64 | hsa-miR-20a-5p | [SZT2](http://www.ncbi.nlm.nih.gov/entrez/query.fcgi?db=gene&cmd=Retrieve&dopt=full_report&list_uids=23334) | SZT2, KICSTOR complex subunit |
| [Details](https://mirdb.org/cgi-bin/target_detail.cgi?targetID=2854558) | 1044 | 64 | hsa-miR-20a-5p | [MID1](http://www.ncbi.nlm.nih.gov/entrez/query.fcgi?db=gene&cmd=Retrieve&dopt=full_report&list_uids=4281) | midline 1 |
| [Details](https://mirdb.org/cgi-bin/target_detail.cgi?targetID=2854653) | 1045 | 64 | hsa-miR-20a-5p | [DPY19L3](http://www.ncbi.nlm.nih.gov/entrez/query.fcgi?db=gene&cmd=Retrieve&dopt=full_report&list_uids=147991) | dpy-19 like C-mannosyltransferase 3 |
| [Details](https://mirdb.org/cgi-bin/target_detail.cgi?targetID=2854665) | 1046 | 64 | hsa-miR-20a-5p | [NFATC2IP](http://www.ncbi.nlm.nih.gov/entrez/query.fcgi?db=gene&cmd=Retrieve&dopt=full_report&list_uids=84901) | nuclear factor of activated T cells 2 interacting protein |
| [Details](https://mirdb.org/cgi-bin/target_detail.cgi?targetID=2854747) | 1047 | 64 | hsa-miR-20a-5p | [IL17RD](http://www.ncbi.nlm.nih.gov/entrez/query.fcgi?db=gene&cmd=Retrieve&dopt=full_report&list_uids=54756) | interleukin 17 receptor D |
| [Details](https://mirdb.org/cgi-bin/target_detail.cgi?targetID=2854822) | 1048 | 64 | hsa-miR-20a-5p | [FBXO28](http://www.ncbi.nlm.nih.gov/entrez/query.fcgi?db=gene&cmd=Retrieve&dopt=full_report&list_uids=23219) | F-box protein 28 |
| [Details](https://mirdb.org/cgi-bin/target_detail.cgi?targetID=2854856) | 1049 | 64 | hsa-miR-20a-5p | [PKIA](http://www.ncbi.nlm.nih.gov/entrez/query.fcgi?db=gene&cmd=Retrieve&dopt=full_report&list_uids=5569) | cAMP-dependent protein kinase inhibitor alpha |
| [Details](https://mirdb.org/cgi-bin/target_detail.cgi?targetID=2854894) | 1050 | 64 | hsa-miR-20a-5p | [MECP2](http://www.ncbi.nlm.nih.gov/entrez/query.fcgi?db=gene&cmd=Retrieve&dopt=full_report&list_uids=4204) | methyl-CpG binding protein 2 |
| [Details](https://mirdb.org/cgi-bin/target_detail.cgi?targetID=2854992) | 1051 | 64 | hsa-miR-20a-5p | [MAP3K1](http://www.ncbi.nlm.nih.gov/entrez/query.fcgi?db=gene&cmd=Retrieve&dopt=full_report&list_uids=4214) | mitogen-activated protein kinase kinase kinase 1 |
| [Details](https://mirdb.org/cgi-bin/target_detail.cgi?targetID=2855026) | 1052 | 64 | hsa-miR-20a-5p | [UGCG](http://www.ncbi.nlm.nih.gov/entrez/query.fcgi?db=gene&cmd=Retrieve&dopt=full_report&list_uids=7357) | UDP-glucose ceramide glucosyltransferase |
| [Details](https://mirdb.org/cgi-bin/target_detail.cgi?targetID=2855028) | 1053 | 64 | hsa-miR-20a-5p | [HCN4](http://www.ncbi.nlm.nih.gov/entrez/query.fcgi?db=gene&cmd=Retrieve&dopt=full_report&list_uids=10021) | hyperpolarization activated cyclic nucleotide gated potassium channel 4 |
| [Details](https://mirdb.org/cgi-bin/target_detail.cgi?targetID=2855064) | 1054 | 64 | hsa-miR-20a-5p | [EPB41L5](http://www.ncbi.nlm.nih.gov/entrez/query.fcgi?db=gene&cmd=Retrieve&dopt=full_report&list_uids=57669) | erythrocyte membrane protein band 4.1 like 5 |
| [Details](https://mirdb.org/cgi-bin/target_detail.cgi?targetID=2855073) | 1055 | 64 | hsa-miR-20a-5p | [CABLES1](http://www.ncbi.nlm.nih.gov/entrez/query.fcgi?db=gene&cmd=Retrieve&dopt=full_report&list_uids=91768) | Cdk5 and Abl enzyme substrate 1 |
| [Details](https://mirdb.org/cgi-bin/target_detail.cgi?targetID=2855327) | 1056 | 64 | hsa-miR-20a-5p | [FAXC](http://www.ncbi.nlm.nih.gov/entrez/query.fcgi?db=gene&cmd=Retrieve&dopt=full_report&list_uids=84553) | failed axon connections homolog |
| [Details](https://mirdb.org/cgi-bin/target_detail.cgi?targetID=2855374) | 1057 | 64 | hsa-miR-20a-5p | [NRG3](http://www.ncbi.nlm.nih.gov/entrez/query.fcgi?db=gene&cmd=Retrieve&dopt=full_report&list_uids=10718) | neuregulin 3 |
| [Details](https://mirdb.org/cgi-bin/target_detail.cgi?targetID=2854097) | 1058 | 63 | hsa-miR-20a-5p | [MED17](http://www.ncbi.nlm.nih.gov/entrez/query.fcgi?db=gene&cmd=Retrieve&dopt=full_report&list_uids=9440) | mediator complex subunit 17 |
| [Details](https://mirdb.org/cgi-bin/target_detail.cgi?targetID=2854098) | 1059 | 63 | hsa-miR-20a-5p | [GDF11](http://www.ncbi.nlm.nih.gov/entrez/query.fcgi?db=gene&cmd=Retrieve&dopt=full_report&list_uids=10220) | growth differentiation factor 11 |
| [Details](https://mirdb.org/cgi-bin/target_detail.cgi?targetID=2854168) | 1060 | 63 | hsa-miR-20a-5p | [MCTP2](http://www.ncbi.nlm.nih.gov/entrez/query.fcgi?db=gene&cmd=Retrieve&dopt=full_report&list_uids=55784) | multiple C2 and transmembrane domain containing 2 |
| [Details](https://mirdb.org/cgi-bin/target_detail.cgi?targetID=2854171) | 1061 | 63 | hsa-miR-20a-5p | [ZDHHC21](http://www.ncbi.nlm.nih.gov/entrez/query.fcgi?db=gene&cmd=Retrieve&dopt=full_report&list_uids=340481) | zinc finger DHHC-type containing 21 |
| [Details](https://mirdb.org/cgi-bin/target_detail.cgi?targetID=2854184) | 1062 | 63 | hsa-miR-20a-5p | [CDC5L](http://www.ncbi.nlm.nih.gov/entrez/query.fcgi?db=gene&cmd=Retrieve&dopt=full_report&list_uids=988) | cell division cycle 5 like |
| [Details](https://mirdb.org/cgi-bin/target_detail.cgi?targetID=2854359) | 1063 | 63 | hsa-miR-20a-5p | [TNS1](http://www.ncbi.nlm.nih.gov/entrez/query.fcgi?db=gene&cmd=Retrieve&dopt=full_report&list_uids=7145) | tensin 1 |
| [Details](https://mirdb.org/cgi-bin/target_detail.cgi?targetID=2854427) | 1064 | 63 | hsa-miR-20a-5p | [FER](http://www.ncbi.nlm.nih.gov/entrez/query.fcgi?db=gene&cmd=Retrieve&dopt=full_report&list_uids=2241) | FER tyrosine kinase |
| [Details](https://mirdb.org/cgi-bin/target_detail.cgi?targetID=2854434) | 1065 | 63 | hsa-miR-20a-5p | [DHRS12](http://www.ncbi.nlm.nih.gov/entrez/query.fcgi?db=gene&cmd=Retrieve&dopt=full_report&list_uids=79758) | dehydrogenase/reductase 12 |
| [Details](https://mirdb.org/cgi-bin/target_detail.cgi?targetID=2854486) | 1066 | 63 | hsa-miR-20a-5p | [SLC24A4](http://www.ncbi.nlm.nih.gov/entrez/query.fcgi?db=gene&cmd=Retrieve&dopt=full_report&list_uids=123041) | solute carrier family 24 member 4 |
| [Details](https://mirdb.org/cgi-bin/target_detail.cgi?targetID=2854607) | 1067 | 63 | hsa-miR-20a-5p | [CARD8](http://www.ncbi.nlm.nih.gov/entrez/query.fcgi?db=gene&cmd=Retrieve&dopt=full_report&list_uids=22900) | caspase recruitment domain family member 8 |
| [Details](https://mirdb.org/cgi-bin/target_detail.cgi?targetID=2854674) | 1068 | 63 | hsa-miR-20a-5p | [FIGNL1](http://www.ncbi.nlm.nih.gov/entrez/query.fcgi?db=gene&cmd=Retrieve&dopt=full_report&list_uids=63979) | fidgetin like 1 |
| [Details](https://mirdb.org/cgi-bin/target_detail.cgi?targetID=2854974) | 1069 | 63 | hsa-miR-20a-5p | [FIBIN](http://www.ncbi.nlm.nih.gov/entrez/query.fcgi?db=gene&cmd=Retrieve&dopt=full_report&list_uids=387758) | fin bud initiation factor homolog |
| [Details](https://mirdb.org/cgi-bin/target_detail.cgi?targetID=2854988) | 1070 | 63 | hsa-miR-20a-5p | [EDA2R](http://www.ncbi.nlm.nih.gov/entrez/query.fcgi?db=gene&cmd=Retrieve&dopt=full_report&list_uids=60401) | ectodysplasin A2 receptor |
| [Details](https://mirdb.org/cgi-bin/target_detail.cgi?targetID=2855196) | 1071 | 63 | hsa-miR-20a-5p | [FBXL22](http://www.ncbi.nlm.nih.gov/entrez/query.fcgi?db=gene&cmd=Retrieve&dopt=full_report&list_uids=283807) | F-box and leucine rich repeat protein 22 |
| [Details](https://mirdb.org/cgi-bin/target_detail.cgi?targetID=2855224) | 1072 | 63 | hsa-miR-20a-5p | [CCND2](http://www.ncbi.nlm.nih.gov/entrez/query.fcgi?db=gene&cmd=Retrieve&dopt=full_report&list_uids=894) | cyclin D2 |
| [Details](https://mirdb.org/cgi-bin/target_detail.cgi?targetID=2855257) | 1073 | 63 | hsa-miR-20a-5p | [ABCA10](http://www.ncbi.nlm.nih.gov/entrez/query.fcgi?db=gene&cmd=Retrieve&dopt=full_report&list_uids=10349) | ATP binding cassette subfamily A member 10 |
| [Details](https://mirdb.org/cgi-bin/target_detail.cgi?targetID=2855276) | 1074 | 63 | hsa-miR-20a-5p | [NR2C2AP](http://www.ncbi.nlm.nih.gov/entrez/query.fcgi?db=gene&cmd=Retrieve&dopt=full_report&list_uids=126382) | nuclear receptor 2C2 associated protein |
| [Details](https://mirdb.org/cgi-bin/target_detail.cgi?targetID=2855288) | 1075 | 63 | hsa-miR-20a-5p | [RND3](http://www.ncbi.nlm.nih.gov/entrez/query.fcgi?db=gene&cmd=Retrieve&dopt=full_report&list_uids=390) | Rho family GTPase 3 |
| [Details](https://mirdb.org/cgi-bin/target_detail.cgi?targetID=2855331) | 1076 | 63 | hsa-miR-20a-5p | [RBMS1](http://www.ncbi.nlm.nih.gov/entrez/query.fcgi?db=gene&cmd=Retrieve&dopt=full_report&list_uids=5937) | RNA binding motif single stranded interacting protein 1 |
| [Details](https://mirdb.org/cgi-bin/target_detail.cgi?targetID=2855366) | 1077 | 63 | hsa-miR-20a-5p | [ACTR1A](http://www.ncbi.nlm.nih.gov/entrez/query.fcgi?db=gene&cmd=Retrieve&dopt=full_report&list_uids=10121) | ARP1 actin related protein 1 homolog A |
| [Details](https://mirdb.org/cgi-bin/target_detail.cgi?targetID=2855394) | 1078 | 63 | hsa-miR-20a-5p | [FRMD4A](http://www.ncbi.nlm.nih.gov/entrez/query.fcgi?db=gene&cmd=Retrieve&dopt=full_report&list_uids=55691) | FERM domain containing 4A |
| [Details](https://mirdb.org/cgi-bin/target_detail.cgi?targetID=2855439) | 1079 | 63 | hsa-miR-20a-5p | [RANBP6](http://www.ncbi.nlm.nih.gov/entrez/query.fcgi?db=gene&cmd=Retrieve&dopt=full_report&list_uids=26953) | RAN binding protein 6 |
| [Details](https://mirdb.org/cgi-bin/target_detail.cgi?targetID=2854110) | 1080 | 62 | hsa-miR-20a-5p | [SLC35F3](http://www.ncbi.nlm.nih.gov/entrez/query.fcgi?db=gene&cmd=Retrieve&dopt=full_report&list_uids=148641) | solute carrier family 35 member F3 |
| [Details](https://mirdb.org/cgi-bin/target_detail.cgi?targetID=2854158) | 1081 | 62 | hsa-miR-20a-5p | [FGF5](http://www.ncbi.nlm.nih.gov/entrez/query.fcgi?db=gene&cmd=Retrieve&dopt=full_report&list_uids=2250) | fibroblast growth factor 5 |
| [Details](https://mirdb.org/cgi-bin/target_detail.cgi?targetID=2854293) | 1082 | 62 | hsa-miR-20a-5p | [WNT9B](http://www.ncbi.nlm.nih.gov/entrez/query.fcgi?db=gene&cmd=Retrieve&dopt=full_report&list_uids=7484) | Wnt family member 9B |
| [Details](https://mirdb.org/cgi-bin/target_detail.cgi?targetID=2854403) | 1083 | 62 | hsa-miR-20a-5p | [IKZF2](http://www.ncbi.nlm.nih.gov/entrez/query.fcgi?db=gene&cmd=Retrieve&dopt=full_report&list_uids=22807) | IKAROS family zinc finger 2 |
| [Details](https://mirdb.org/cgi-bin/target_detail.cgi?targetID=2854423) | 1084 | 62 | hsa-miR-20a-5p | [CAMTA2](http://www.ncbi.nlm.nih.gov/entrez/query.fcgi?db=gene&cmd=Retrieve&dopt=full_report&list_uids=23125) | calmodulin binding transcription activator 2 |
| [Details](https://mirdb.org/cgi-bin/target_detail.cgi?targetID=2854458) | 1085 | 62 | hsa-miR-20a-5p | [DAZAP2](http://www.ncbi.nlm.nih.gov/entrez/query.fcgi?db=gene&cmd=Retrieve&dopt=full_report&list_uids=9802) | DAZ associated protein 2 |
| [Details](https://mirdb.org/cgi-bin/target_detail.cgi?targetID=2854487) | 1086 | 62 | hsa-miR-20a-5p | [SDC2](http://www.ncbi.nlm.nih.gov/entrez/query.fcgi?db=gene&cmd=Retrieve&dopt=full_report&list_uids=6383) | syndecan 2 |
| [Details](https://mirdb.org/cgi-bin/target_detail.cgi?targetID=2854515) | 1087 | 62 | hsa-miR-20a-5p | [YTHDC1](http://www.ncbi.nlm.nih.gov/entrez/query.fcgi?db=gene&cmd=Retrieve&dopt=full_report&list_uids=91746) | YTH domain containing 1 |
| [Details](https://mirdb.org/cgi-bin/target_detail.cgi?targetID=2854567) | 1088 | 62 | hsa-miR-20a-5p | [YES1](http://www.ncbi.nlm.nih.gov/entrez/query.fcgi?db=gene&cmd=Retrieve&dopt=full_report&list_uids=7525) | YES proto-oncogene 1, Src family tyrosine kinase |
| [Details](https://mirdb.org/cgi-bin/target_detail.cgi?targetID=2854619) | 1089 | 62 | hsa-miR-20a-5p | [PCBP2](http://www.ncbi.nlm.nih.gov/entrez/query.fcgi?db=gene&cmd=Retrieve&dopt=full_report&list_uids=5094) | poly(rC) binding protein 2 |
| [Details](https://mirdb.org/cgi-bin/target_detail.cgi?targetID=2854637) | 1090 | 62 | hsa-miR-20a-5p | [CHIC1](http://www.ncbi.nlm.nih.gov/entrez/query.fcgi?db=gene&cmd=Retrieve&dopt=full_report&list_uids=53344) | cysteine rich hydrophobic domain 1 |
| [Details](https://mirdb.org/cgi-bin/target_detail.cgi?targetID=2854676) | 1091 | 62 | hsa-miR-20a-5p | [TMEM123](http://www.ncbi.nlm.nih.gov/entrez/query.fcgi?db=gene&cmd=Retrieve&dopt=full_report&list_uids=114908) | transmembrane protein 123 |
| [Details](https://mirdb.org/cgi-bin/target_detail.cgi?targetID=2854693) | 1092 | 62 | hsa-miR-20a-5p | [UBR5](http://www.ncbi.nlm.nih.gov/entrez/query.fcgi?db=gene&cmd=Retrieve&dopt=full_report&list_uids=51366) | ubiquitin protein ligase E3 component n-recognin 5 |
| [Details](https://mirdb.org/cgi-bin/target_detail.cgi?targetID=2854772) | 1093 | 62 | hsa-miR-20a-5p | [HECA](http://www.ncbi.nlm.nih.gov/entrez/query.fcgi?db=gene&cmd=Retrieve&dopt=full_report&list_uids=51696) | hdc homolog, cell cycle regulator |
| [Details](https://mirdb.org/cgi-bin/target_detail.cgi?targetID=2854783) | 1094 | 62 | hsa-miR-20a-5p | [EIF4H](http://www.ncbi.nlm.nih.gov/entrez/query.fcgi?db=gene&cmd=Retrieve&dopt=full_report&list_uids=7458) | eukaryotic translation initiation factor 4H |
| [Details](https://mirdb.org/cgi-bin/target_detail.cgi?targetID=2854818) | 1095 | 62 | hsa-miR-20a-5p | [NFE2L1](http://www.ncbi.nlm.nih.gov/entrez/query.fcgi?db=gene&cmd=Retrieve&dopt=full_report&list_uids=4779) | nuclear factor, erythroid 2 like 1 |
| [Details](https://mirdb.org/cgi-bin/target_detail.cgi?targetID=2854973) | 1096 | 62 | hsa-miR-20a-5p | [UHRF1BP1](http://www.ncbi.nlm.nih.gov/entrez/query.fcgi?db=gene&cmd=Retrieve&dopt=full_report&list_uids=54887) | UHRF1 binding protein 1 |
| [Details](https://mirdb.org/cgi-bin/target_detail.cgi?targetID=2854987) | 1097 | 62 | hsa-miR-20a-5p | [TNRC6C](http://www.ncbi.nlm.nih.gov/entrez/query.fcgi?db=gene&cmd=Retrieve&dopt=full_report&list_uids=57690) | trinucleotide repeat containing 6C |
| [Details](https://mirdb.org/cgi-bin/target_detail.cgi?targetID=2855086) | 1098 | 62 | hsa-miR-20a-5p | [LIAS](http://www.ncbi.nlm.nih.gov/entrez/query.fcgi?db=gene&cmd=Retrieve&dopt=full_report&list_uids=11019) | lipoic acid synthetase |
| [Details](https://mirdb.org/cgi-bin/target_detail.cgi?targetID=2855087) | 1099 | 62 | hsa-miR-20a-5p | [POGK](http://www.ncbi.nlm.nih.gov/entrez/query.fcgi?db=gene&cmd=Retrieve&dopt=full_report&list_uids=57645) | pogo transposable element derived with KRAB domain |
| [Details](https://mirdb.org/cgi-bin/target_detail.cgi?targetID=2855214) | 1100 | 62 | hsa-miR-20a-5p | [DGKE](http://www.ncbi.nlm.nih.gov/entrez/query.fcgi?db=gene&cmd=Retrieve&dopt=full_report&list_uids=8526) | diacylglycerol kinase epsilon |
| [Details](https://mirdb.org/cgi-bin/target_detail.cgi?targetID=2855446) | 1101 | 62 | hsa-miR-20a-5p | [FNBP4](http://www.ncbi.nlm.nih.gov/entrez/query.fcgi?db=gene&cmd=Retrieve&dopt=full_report&list_uids=23360) | formin binding protein 4 |
| [Details](https://mirdb.org/cgi-bin/target_detail.cgi?targetID=2854205) | 1102 | 61 | hsa-miR-20a-5p | [VWA8](http://www.ncbi.nlm.nih.gov/entrez/query.fcgi?db=gene&cmd=Retrieve&dopt=full_report&list_uids=23078) | von Willebrand factor A domain containing 8 |
| [Details](https://mirdb.org/cgi-bin/target_detail.cgi?targetID=2854214) | 1103 | 61 | hsa-miR-20a-5p | [ACADSB](http://www.ncbi.nlm.nih.gov/entrez/query.fcgi?db=gene&cmd=Retrieve&dopt=full_report&list_uids=36) | acyl-CoA dehydrogenase short/branched chain |
| [Details](https://mirdb.org/cgi-bin/target_detail.cgi?targetID=2854332) | 1104 | 61 | hsa-miR-20a-5p | [CCDC68](http://www.ncbi.nlm.nih.gov/entrez/query.fcgi?db=gene&cmd=Retrieve&dopt=full_report&list_uids=80323) | coiled-coil domain containing 68 |
| [Details](https://mirdb.org/cgi-bin/target_detail.cgi?targetID=2854381) | 1105 | 61 | hsa-miR-20a-5p | [MRGPRX3](http://www.ncbi.nlm.nih.gov/entrez/query.fcgi?db=gene&cmd=Retrieve&dopt=full_report&list_uids=117195) | MAS related GPR family member X3 |
| [Details](https://mirdb.org/cgi-bin/target_detail.cgi?targetID=2854441) | 1106 | 61 | hsa-miR-20a-5p | [NPM1](http://www.ncbi.nlm.nih.gov/entrez/query.fcgi?db=gene&cmd=Retrieve&dopt=full_report&list_uids=4869) | nucleophosmin 1 |
| [Details](https://mirdb.org/cgi-bin/target_detail.cgi?targetID=2854497) | 1107 | 61 | hsa-miR-20a-5p | [SART1](http://www.ncbi.nlm.nih.gov/entrez/query.fcgi?db=gene&cmd=Retrieve&dopt=full_report&list_uids=9092) | spliceosome associated factor 1, recruiter of U4/U6.U5 tri-snRNP |
| [Details](https://mirdb.org/cgi-bin/target_detail.cgi?targetID=2854516) | 1108 | 61 | hsa-miR-20a-5p | [LAMP5](http://www.ncbi.nlm.nih.gov/entrez/query.fcgi?db=gene&cmd=Retrieve&dopt=full_report&list_uids=24141) | lysosomal associated membrane protein family member 5 |
| [Details](https://mirdb.org/cgi-bin/target_detail.cgi?targetID=2854520) | 1109 | 61 | hsa-miR-20a-5p | [ADGRL3](http://www.ncbi.nlm.nih.gov/entrez/query.fcgi?db=gene&cmd=Retrieve&dopt=full_report&list_uids=23284) | adhesion G protein-coupled receptor L3 |
| [Details](https://mirdb.org/cgi-bin/target_detail.cgi?targetID=2854531) | 1110 | 61 | hsa-miR-20a-5p | [TMEM242](http://www.ncbi.nlm.nih.gov/entrez/query.fcgi?db=gene&cmd=Retrieve&dopt=full_report&list_uids=729515) | transmembrane protein 242 |
| [Details](https://mirdb.org/cgi-bin/target_detail.cgi?targetID=2854561) | 1111 | 61 | hsa-miR-20a-5p | [FAM8A1](http://www.ncbi.nlm.nih.gov/entrez/query.fcgi?db=gene&cmd=Retrieve&dopt=full_report&list_uids=51439) | family with sequence similarity 8 member A1 |
| [Details](https://mirdb.org/cgi-bin/target_detail.cgi?targetID=2854603) | 1112 | 61 | hsa-miR-20a-5p | [TNFSF13B](http://www.ncbi.nlm.nih.gov/entrez/query.fcgi?db=gene&cmd=Retrieve&dopt=full_report&list_uids=10673) | TNF superfamily member 13b |
| [Details](https://mirdb.org/cgi-bin/target_detail.cgi?targetID=2854719) | 1113 | 61 | hsa-miR-20a-5p | [PNPLA4](http://www.ncbi.nlm.nih.gov/entrez/query.fcgi?db=gene&cmd=Retrieve&dopt=full_report&list_uids=8228) | patatin like phospholipase domain containing 4 |
| [Details](https://mirdb.org/cgi-bin/target_detail.cgi?targetID=2854870) | 1114 | 61 | hsa-miR-20a-5p | [FGD1](http://www.ncbi.nlm.nih.gov/entrez/query.fcgi?db=gene&cmd=Retrieve&dopt=full_report&list_uids=2245) | FYVE, RhoGEF and PH domain containing 1 |
| [Details](https://mirdb.org/cgi-bin/target_detail.cgi?targetID=2854893) | 1115 | 61 | hsa-miR-20a-5p | [YPEL4](http://www.ncbi.nlm.nih.gov/entrez/query.fcgi?db=gene&cmd=Retrieve&dopt=full_report&list_uids=219539) | yippee like 4 |
| [Details](https://mirdb.org/cgi-bin/target_detail.cgi?targetID=2854916) | 1116 | 61 | hsa-miR-20a-5p | [F2RL3](http://www.ncbi.nlm.nih.gov/entrez/query.fcgi?db=gene&cmd=Retrieve&dopt=full_report&list_uids=9002) | F2R like thrombin or trypsin receptor 3 |
| [Details](https://mirdb.org/cgi-bin/target_detail.cgi?targetID=2854924) | 1117 | 61 | hsa-miR-20a-5p | [MMP3](http://www.ncbi.nlm.nih.gov/entrez/query.fcgi?db=gene&cmd=Retrieve&dopt=full_report&list_uids=4314) | matrix metallopeptidase 3 |
| [Details](https://mirdb.org/cgi-bin/target_detail.cgi?targetID=2854971) | 1118 | 61 | hsa-miR-20a-5p | [INHBA](http://www.ncbi.nlm.nih.gov/entrez/query.fcgi?db=gene&cmd=Retrieve&dopt=full_report&list_uids=3624) | inhibin subunit beta A |
| [Details](https://mirdb.org/cgi-bin/target_detail.cgi?targetID=2855060) | 1119 | 61 | hsa-miR-20a-5p | [TANC2](http://www.ncbi.nlm.nih.gov/entrez/query.fcgi?db=gene&cmd=Retrieve&dopt=full_report&list_uids=26115) | tetratricopeptide repeat, ankyrin repeat and coiled-coil containing 2 |
| [Details](https://mirdb.org/cgi-bin/target_detail.cgi?targetID=2855107) | 1120 | 61 | hsa-miR-20a-5p | [BMP8B](http://www.ncbi.nlm.nih.gov/entrez/query.fcgi?db=gene&cmd=Retrieve&dopt=full_report&list_uids=656) | bone morphogenetic protein 8b |
| [Details](https://mirdb.org/cgi-bin/target_detail.cgi?targetID=2855187) | 1121 | 61 | hsa-miR-20a-5p | [SETD2](http://www.ncbi.nlm.nih.gov/entrez/query.fcgi?db=gene&cmd=Retrieve&dopt=full_report&list_uids=29072) | SET domain containing 2, histone lysine methyltransferase |
| [Details](https://mirdb.org/cgi-bin/target_detail.cgi?targetID=2855192) | 1122 | 61 | hsa-miR-20a-5p | [CAPS2](http://www.ncbi.nlm.nih.gov/entrez/query.fcgi?db=gene&cmd=Retrieve&dopt=full_report&list_uids=84698) | calcyphosine 2 |
| [Details](https://mirdb.org/cgi-bin/target_detail.cgi?targetID=2855236) | 1123 | 61 | hsa-miR-20a-5p | [FOXJ2](http://www.ncbi.nlm.nih.gov/entrez/query.fcgi?db=gene&cmd=Retrieve&dopt=full_report&list_uids=55810) | forkhead box J2 |
| [Details](https://mirdb.org/cgi-bin/target_detail.cgi?targetID=2855275) | 1124 | 61 | hsa-miR-20a-5p | [TBL1X](http://www.ncbi.nlm.nih.gov/entrez/query.fcgi?db=gene&cmd=Retrieve&dopt=full_report&list_uids=6907) | transducin beta like 1 X-linked |
| [Details](https://mirdb.org/cgi-bin/target_detail.cgi?targetID=2855353) | 1125 | 61 | hsa-miR-20a-5p | [NUFIP2](http://www.ncbi.nlm.nih.gov/entrez/query.fcgi?db=gene&cmd=Retrieve&dopt=full_report&list_uids=57532) | nuclear FMR1 interacting protein 2 |
| [Details](https://mirdb.org/cgi-bin/target_detail.cgi?targetID=2855369) | 1126 | 61 | hsa-miR-20a-5p | [WDR36](http://www.ncbi.nlm.nih.gov/entrez/query.fcgi?db=gene&cmd=Retrieve&dopt=full_report&list_uids=134430) | WD repeat domain 36 |
| [Details](https://mirdb.org/cgi-bin/target_detail.cgi?targetID=2855420) | 1127 | 61 | hsa-miR-20a-5p | [JRKL](http://www.ncbi.nlm.nih.gov/entrez/query.fcgi?db=gene&cmd=Retrieve&dopt=full_report&list_uids=8690) | JRK like |
| [Details](https://mirdb.org/cgi-bin/target_detail.cgi?targetID=2854190) | 1128 | 60 | hsa-miR-20a-5p | [SLC36A1](http://www.ncbi.nlm.nih.gov/entrez/query.fcgi?db=gene&cmd=Retrieve&dopt=full_report&list_uids=206358) | solute carrier family 36 member 1 |
| [Details](https://mirdb.org/cgi-bin/target_detail.cgi?targetID=2854306) | 1129 | 60 | hsa-miR-20a-5p | [FAM227A](http://www.ncbi.nlm.nih.gov/entrez/query.fcgi?db=gene&cmd=Retrieve&dopt=full_report&list_uids=646851) | family with sequence similarity 227 member A |
| [Details](https://mirdb.org/cgi-bin/target_detail.cgi?targetID=2854462) | 1130 | 60 | hsa-miR-20a-5p | [ARSE](http://www.ncbi.nlm.nih.gov/entrez/query.fcgi?db=gene&cmd=Retrieve&dopt=full_report&list_uids=415) | arylsulfatase E |
| [Details](https://mirdb.org/cgi-bin/target_detail.cgi?targetID=2854495) | 1131 | 60 | hsa-miR-20a-5p | [FBXO11](http://www.ncbi.nlm.nih.gov/entrez/query.fcgi?db=gene&cmd=Retrieve&dopt=full_report&list_uids=80204) | F-box protein 11 |
| [Details](https://mirdb.org/cgi-bin/target_detail.cgi?targetID=2854540) | 1132 | 60 | hsa-miR-20a-5p | [FXR1](http://www.ncbi.nlm.nih.gov/entrez/query.fcgi?db=gene&cmd=Retrieve&dopt=full_report&list_uids=8087) | FMR1 autosomal homolog 1 |
| [Details](https://mirdb.org/cgi-bin/target_detail.cgi?targetID=2854581) | 1133 | 60 | hsa-miR-20a-5p | [PDZD11](http://www.ncbi.nlm.nih.gov/entrez/query.fcgi?db=gene&cmd=Retrieve&dopt=full_report&list_uids=51248) | PDZ domain containing 11 |
| [Details](https://mirdb.org/cgi-bin/target_detail.cgi?targetID=2854590) | 1134 | 60 | hsa-miR-20a-5p | [MXD1](http://www.ncbi.nlm.nih.gov/entrez/query.fcgi?db=gene&cmd=Retrieve&dopt=full_report&list_uids=4084) | MAX dimerization protein 1 |
| [Details](https://mirdb.org/cgi-bin/target_detail.cgi?targetID=2854616) | 1135 | 60 | hsa-miR-20a-5p | [FNIP2](http://www.ncbi.nlm.nih.gov/entrez/query.fcgi?db=gene&cmd=Retrieve&dopt=full_report&list_uids=57600) | folliculin interacting protein 2 |
| [Details](https://mirdb.org/cgi-bin/target_detail.cgi?targetID=2854631) | 1136 | 60 | hsa-miR-20a-5p | [VPS13C](http://www.ncbi.nlm.nih.gov/entrez/query.fcgi?db=gene&cmd=Retrieve&dopt=full_report&list_uids=54832) | vacuolar protein sorting 13 homolog C |
| [Details](https://mirdb.org/cgi-bin/target_detail.cgi?targetID=2854677) | 1137 | 60 | hsa-miR-20a-5p | [RXFP1](http://www.ncbi.nlm.nih.gov/entrez/query.fcgi?db=gene&cmd=Retrieve&dopt=full_report&list_uids=59350) | relaxin family peptide receptor 1 |
| [Details](https://mirdb.org/cgi-bin/target_detail.cgi?targetID=2854692) | 1138 | 60 | hsa-miR-20a-5p | [IKBIP](http://www.ncbi.nlm.nih.gov/entrez/query.fcgi?db=gene&cmd=Retrieve&dopt=full_report&list_uids=121457) | IKBKB interacting protein |
| [Details](https://mirdb.org/cgi-bin/target_detail.cgi?targetID=2854730) | 1139 | 60 | hsa-miR-20a-5p | [ZNF107](http://www.ncbi.nlm.nih.gov/entrez/query.fcgi?db=gene&cmd=Retrieve&dopt=full_report&list_uids=51427) | zinc finger protein 107 |
| [Details](https://mirdb.org/cgi-bin/target_detail.cgi?targetID=2854746) | 1140 | 60 | hsa-miR-20a-5p | [RAX](http://www.ncbi.nlm.nih.gov/entrez/query.fcgi?db=gene&cmd=Retrieve&dopt=full_report&list_uids=30062) | retina and anterior neural fold homeobox |
| [Details](https://mirdb.org/cgi-bin/target_detail.cgi?targetID=2854748) | 1141 | 60 | hsa-miR-20a-5p | [BTN3A2](http://www.ncbi.nlm.nih.gov/entrez/query.fcgi?db=gene&cmd=Retrieve&dopt=full_report&list_uids=11118) | butyrophilin subfamily 3 member A2 |
| [Details](https://mirdb.org/cgi-bin/target_detail.cgi?targetID=2854804) | 1142 | 60 | hsa-miR-20a-5p | [PTGS1](http://www.ncbi.nlm.nih.gov/entrez/query.fcgi?db=gene&cmd=Retrieve&dopt=full_report&list_uids=5742) | prostaglandin-endoperoxide synthase 1 |
| [Details](https://mirdb.org/cgi-bin/target_detail.cgi?targetID=2854805) | 1143 | 60 | hsa-miR-20a-5p | [C15orf41](http://www.ncbi.nlm.nih.gov/entrez/query.fcgi?db=gene&cmd=Retrieve&dopt=full_report&list_uids=84529) | chromosome 15 open reading frame 41 |
| [Details](https://mirdb.org/cgi-bin/target_detail.cgi?targetID=2855039) | 1144 | 60 | hsa-miR-20a-5p | [CDC40](http://www.ncbi.nlm.nih.gov/entrez/query.fcgi?db=gene&cmd=Retrieve&dopt=full_report&list_uids=51362) | cell division cycle 40 |
| [Details](https://mirdb.org/cgi-bin/target_detail.cgi?targetID=2855134) | 1145 | 60 | hsa-miR-20a-5p | [AP1G1](http://www.ncbi.nlm.nih.gov/entrez/query.fcgi?db=gene&cmd=Retrieve&dopt=full_report&list_uids=164) | adaptor related protein complex 1 subunit gamma 1 |
| [Details](https://mirdb.org/cgi-bin/target_detail.cgi?targetID=2855154) | 1146 | 60 | hsa-miR-20a-5p | [CBX5](http://www.ncbi.nlm.nih.gov/entrez/query.fcgi?db=gene&cmd=Retrieve&dopt=full_report&list_uids=23468) | chromobox 5 |
| [Details](https://mirdb.org/cgi-bin/target_detail.cgi?targetID=2855169) | 1147 | 60 | hsa-miR-20a-5p | [SRSF2](http://www.ncbi.nlm.nih.gov/entrez/query.fcgi?db=gene&cmd=Retrieve&dopt=full_report&list_uids=6427) | serine and arginine rich splicing factor 2 |
| [Details](https://mirdb.org/cgi-bin/target_detail.cgi?targetID=2855175) | 1148 | 60 | hsa-miR-20a-5p | [SEMA5A](http://www.ncbi.nlm.nih.gov/entrez/query.fcgi?db=gene&cmd=Retrieve&dopt=full_report&list_uids=9037) | semaphorin 5A |
| [Details](https://mirdb.org/cgi-bin/target_detail.cgi?targetID=2855283) | 1149 | 60 | hsa-miR-20a-5p | [SLITRK4](http://www.ncbi.nlm.nih.gov/entrez/query.fcgi?db=gene&cmd=Retrieve&dopt=full_report&list_uids=139065) | SLIT and NTRK like family member 4 |
| [Details](https://mirdb.org/cgi-bin/target_detail.cgi?targetID=2855355) | 1150 | 60 | hsa-miR-20a-5p | [AJUBA](http://www.ncbi.nlm.nih.gov/entrez/query.fcgi?db=gene&cmd=Retrieve&dopt=full_report&list_uids=84962) | ajuba LIM protein |
| [Details](https://mirdb.org/cgi-bin/target_detail.cgi?targetID=2855364) | 1151 | 60 | hsa-miR-20a-5p | [RGS4](http://www.ncbi.nlm.nih.gov/entrez/query.fcgi?db=gene&cmd=Retrieve&dopt=full_report&list_uids=5999) | regulator of G protein signaling 4 |
| [Details](https://mirdb.org/cgi-bin/target_detail.cgi?targetID=2855367) | 1152 | 60 | hsa-miR-20a-5p | [TNC](http://www.ncbi.nlm.nih.gov/entrez/query.fcgi?db=gene&cmd=Retrieve&dopt=full_report&list_uids=3371) | tenascin C |
| [Details](https://mirdb.org/cgi-bin/target_detail.cgi?targetID=2855407) | 1153 | 60 | hsa-miR-20a-5p | [AIFM2](http://www.ncbi.nlm.nih.gov/entrez/query.fcgi?db=gene&cmd=Retrieve&dopt=full_report&list_uids=84883) | apoptosis inducing factor, mitochondria associated 2 |
| [Details](https://mirdb.org/cgi-bin/target_detail.cgi?targetID=2855449) | 1154 | 60 | hsa-miR-20a-5p | [CYP2U1](http://www.ncbi.nlm.nih.gov/entrez/query.fcgi?db=gene&cmd=Retrieve&dopt=full_report&list_uids=113612) | cytochrome P450 family 2 subfamily U member 1 |
| [Details](https://mirdb.org/cgi-bin/target_detail.cgi?targetID=2854250) | 1155 | 59 | hsa-miR-20a-5p | [FKBP5](http://www.ncbi.nlm.nih.gov/entrez/query.fcgi?db=gene&cmd=Retrieve&dopt=full_report&list_uids=2289) | FKBP prolyl isomerase 5 |
| [Details](https://mirdb.org/cgi-bin/target_detail.cgi?targetID=2854326) | 1156 | 59 | hsa-miR-20a-5p | [KBTBD8](http://www.ncbi.nlm.nih.gov/entrez/query.fcgi?db=gene&cmd=Retrieve&dopt=full_report&list_uids=84541) | kelch repeat and BTB domain containing 8 |
| [Details](https://mirdb.org/cgi-bin/target_detail.cgi?targetID=2854350) | 1157 | 59 | hsa-miR-20a-5p | [FNDC3A](http://www.ncbi.nlm.nih.gov/entrez/query.fcgi?db=gene&cmd=Retrieve&dopt=full_report&list_uids=22862) | fibronectin type III domain containing 3A |
| [Details](https://mirdb.org/cgi-bin/target_detail.cgi?targetID=2854456) | 1158 | 59 | hsa-miR-20a-5p | [DEDD](http://www.ncbi.nlm.nih.gov/entrez/query.fcgi?db=gene&cmd=Retrieve&dopt=full_report&list_uids=9191) | death effector domain containing |
| [Details](https://mirdb.org/cgi-bin/target_detail.cgi?targetID=2854485) | 1159 | 59 | hsa-miR-20a-5p | [NAV2](http://www.ncbi.nlm.nih.gov/entrez/query.fcgi?db=gene&cmd=Retrieve&dopt=full_report&list_uids=89797) | neuron navigator 2 |
| [Details](https://mirdb.org/cgi-bin/target_detail.cgi?targetID=2854618) | 1160 | 59 | hsa-miR-20a-5p | [STC1](http://www.ncbi.nlm.nih.gov/entrez/query.fcgi?db=gene&cmd=Retrieve&dopt=full_report&list_uids=6781) | stanniocalcin 1 |
| [Details](https://mirdb.org/cgi-bin/target_detail.cgi?targetID=2854776) | 1161 | 59 | hsa-miR-20a-5p | [MIER2](http://www.ncbi.nlm.nih.gov/entrez/query.fcgi?db=gene&cmd=Retrieve&dopt=full_report&list_uids=54531) | MIER family member 2 |
| [Details](https://mirdb.org/cgi-bin/target_detail.cgi?targetID=2854912) | 1162 | 59 | hsa-miR-20a-5p | [FAF2](http://www.ncbi.nlm.nih.gov/entrez/query.fcgi?db=gene&cmd=Retrieve&dopt=full_report&list_uids=23197) | Fas associated factor family member 2 |
| [Details](https://mirdb.org/cgi-bin/target_detail.cgi?targetID=2854917) | 1163 | 59 | hsa-miR-20a-5p | [MRPL19](http://www.ncbi.nlm.nih.gov/entrez/query.fcgi?db=gene&cmd=Retrieve&dopt=full_report&list_uids=9801) | mitochondrial ribosomal protein L19 |
| [Details](https://mirdb.org/cgi-bin/target_detail.cgi?targetID=2854942) | 1164 | 59 | hsa-miR-20a-5p | [PIP4K2A](http://www.ncbi.nlm.nih.gov/entrez/query.fcgi?db=gene&cmd=Retrieve&dopt=full_report&list_uids=5305) | phosphatidylinositol-5-phosphate 4-kinase type 2 alpha |
| [Details](https://mirdb.org/cgi-bin/target_detail.cgi?targetID=2855020) | 1165 | 59 | hsa-miR-20a-5p | [CBX1](http://www.ncbi.nlm.nih.gov/entrez/query.fcgi?db=gene&cmd=Retrieve&dopt=full_report&list_uids=10951) | chromobox 1 |
| [Details](https://mirdb.org/cgi-bin/target_detail.cgi?targetID=2855049) | 1166 | 59 | hsa-miR-20a-5p | [ZDHHC8](http://www.ncbi.nlm.nih.gov/entrez/query.fcgi?db=gene&cmd=Retrieve&dopt=full_report&list_uids=29801) | zinc finger DHHC-type containing 8 |
| [Details](https://mirdb.org/cgi-bin/target_detail.cgi?targetID=2855088) | 1167 | 59 | hsa-miR-20a-5p | [DIP2A](http://www.ncbi.nlm.nih.gov/entrez/query.fcgi?db=gene&cmd=Retrieve&dopt=full_report&list_uids=23181) | disco interacting protein 2 homolog A |
| [Details](https://mirdb.org/cgi-bin/target_detail.cgi?targetID=2855095) | 1168 | 59 | hsa-miR-20a-5p | [FANCD2](http://www.ncbi.nlm.nih.gov/entrez/query.fcgi?db=gene&cmd=Retrieve&dopt=full_report&list_uids=2177) | FA complementation group D2 |
| [Details](https://mirdb.org/cgi-bin/target_detail.cgi?targetID=2855178) | 1169 | 59 | hsa-miR-20a-5p | [C3orf35](http://www.ncbi.nlm.nih.gov/entrez/query.fcgi?db=gene&cmd=Retrieve&dopt=full_report&list_uids=339883) | chromosome 3 open reading frame 35 |
| [Details](https://mirdb.org/cgi-bin/target_detail.cgi?targetID=2855221) | 1170 | 59 | hsa-miR-20a-5p | [SULF1](http://www.ncbi.nlm.nih.gov/entrez/query.fcgi?db=gene&cmd=Retrieve&dopt=full_report&list_uids=23213) | sulfatase 1 |
| [Details](https://mirdb.org/cgi-bin/target_detail.cgi?targetID=2855309) | 1171 | 59 | hsa-miR-20a-5p | [GK5](http://www.ncbi.nlm.nih.gov/entrez/query.fcgi?db=gene&cmd=Retrieve&dopt=full_report&list_uids=256356) | glycerol kinase 5 |
| [Details](https://mirdb.org/cgi-bin/target_detail.cgi?targetID=2855323) | 1172 | 59 | hsa-miR-20a-5p | [TBCEL](http://www.ncbi.nlm.nih.gov/entrez/query.fcgi?db=gene&cmd=Retrieve&dopt=full_report&list_uids=219899) | tubulin folding cofactor E like |
| [Details](https://mirdb.org/cgi-bin/target_detail.cgi?targetID=2855349) | 1173 | 59 | hsa-miR-20a-5p | [FOXA1](http://www.ncbi.nlm.nih.gov/entrez/query.fcgi?db=gene&cmd=Retrieve&dopt=full_report&list_uids=3169) | forkhead box A1 |
| [Details](https://mirdb.org/cgi-bin/target_detail.cgi?targetID=2855419) | 1174 | 59 | hsa-miR-20a-5p | [MXI1](http://www.ncbi.nlm.nih.gov/entrez/query.fcgi?db=gene&cmd=Retrieve&dopt=full_report&list_uids=4601) | MAX interactor 1, dimerization protein |
| [Details](https://mirdb.org/cgi-bin/target_detail.cgi?targetID=2854121) | 1175 | 58 | hsa-miR-20a-5p | [NAA50](http://www.ncbi.nlm.nih.gov/entrez/query.fcgi?db=gene&cmd=Retrieve&dopt=full_report&list_uids=80218) | N(alpha)-acetyltransferase 50, NatE catalytic subunit |
| [Details](https://mirdb.org/cgi-bin/target_detail.cgi?targetID=2854150) | 1176 | 58 | hsa-miR-20a-5p | [MYCN](http://www.ncbi.nlm.nih.gov/entrez/query.fcgi?db=gene&cmd=Retrieve&dopt=full_report&list_uids=4613) | MYCN proto-oncogene, bHLH transcription factor |
| [Details](https://mirdb.org/cgi-bin/target_detail.cgi?targetID=2854154) | 1177 | 58 | hsa-miR-20a-5p | [PDK3](http://www.ncbi.nlm.nih.gov/entrez/query.fcgi?db=gene&cmd=Retrieve&dopt=full_report&list_uids=5165) | pyruvate dehydrogenase kinase 3 |
| [Details](https://mirdb.org/cgi-bin/target_detail.cgi?targetID=2854185) | 1178 | 58 | hsa-miR-20a-5p | [AVL9](http://www.ncbi.nlm.nih.gov/entrez/query.fcgi?db=gene&cmd=Retrieve&dopt=full_report&list_uids=23080) | AVL9 cell migration associated |
| [Details](https://mirdb.org/cgi-bin/target_detail.cgi?targetID=2854312) | 1179 | 58 | hsa-miR-20a-5p | [COL19A1](http://www.ncbi.nlm.nih.gov/entrez/query.fcgi?db=gene&cmd=Retrieve&dopt=full_report&list_uids=1310) | collagen type XIX alpha 1 chain |
| [Details](https://mirdb.org/cgi-bin/target_detail.cgi?targetID=2854376) | 1180 | 58 | hsa-miR-20a-5p | [AFF4](http://www.ncbi.nlm.nih.gov/entrez/query.fcgi?db=gene&cmd=Retrieve&dopt=full_report&list_uids=27125) | AF4/FMR2 family member 4 |
| [Details](https://mirdb.org/cgi-bin/target_detail.cgi?targetID=2854406) | 1181 | 58 | hsa-miR-20a-5p | [TRIM8](http://www.ncbi.nlm.nih.gov/entrez/query.fcgi?db=gene&cmd=Retrieve&dopt=full_report&list_uids=81603) | tripartite motif containing 8 |
| [Details](https://mirdb.org/cgi-bin/target_detail.cgi?targetID=2854500) | 1182 | 58 | hsa-miR-20a-5p | [FGF4](http://www.ncbi.nlm.nih.gov/entrez/query.fcgi?db=gene&cmd=Retrieve&dopt=full_report&list_uids=2249) | fibroblast growth factor 4 |
| [Details](https://mirdb.org/cgi-bin/target_detail.cgi?targetID=2854565) | 1183 | 58 | hsa-miR-20a-5p | [PRND](http://www.ncbi.nlm.nih.gov/entrez/query.fcgi?db=gene&cmd=Retrieve&dopt=full_report&list_uids=23627) | prion like protein doppel |
| [Details](https://mirdb.org/cgi-bin/target_detail.cgi?targetID=2854623) | 1184 | 58 | hsa-miR-20a-5p | [CD36](http://www.ncbi.nlm.nih.gov/entrez/query.fcgi?db=gene&cmd=Retrieve&dopt=full_report&list_uids=948) | CD36 molecule |
| [Details](https://mirdb.org/cgi-bin/target_detail.cgi?targetID=2854643) | 1185 | 58 | hsa-miR-20a-5p | [C21orf58](http://www.ncbi.nlm.nih.gov/entrez/query.fcgi?db=gene&cmd=Retrieve&dopt=full_report&list_uids=54058) | chromosome 21 open reading frame 58 |
| [Details](https://mirdb.org/cgi-bin/target_detail.cgi?targetID=2854675) | 1186 | 58 | hsa-miR-20a-5p | [CPOX](http://www.ncbi.nlm.nih.gov/entrez/query.fcgi?db=gene&cmd=Retrieve&dopt=full_report&list_uids=1371) | coproporphyrinogen oxidase |
| [Details](https://mirdb.org/cgi-bin/target_detail.cgi?targetID=2854705) | 1187 | 58 | hsa-miR-20a-5p | [NRSN1](http://www.ncbi.nlm.nih.gov/entrez/query.fcgi?db=gene&cmd=Retrieve&dopt=full_report&list_uids=140767) | neurensin 1 |
| [Details](https://mirdb.org/cgi-bin/target_detail.cgi?targetID=2854774) | 1188 | 58 | hsa-miR-20a-5p | [SKI](http://www.ncbi.nlm.nih.gov/entrez/query.fcgi?db=gene&cmd=Retrieve&dopt=full_report&list_uids=6497) | SKI proto-oncogene |
| [Details](https://mirdb.org/cgi-bin/target_detail.cgi?targetID=2854781) | 1189 | 58 | hsa-miR-20a-5p | [EIF4E2](http://www.ncbi.nlm.nih.gov/entrez/query.fcgi?db=gene&cmd=Retrieve&dopt=full_report&list_uids=9470) | eukaryotic translation initiation factor 4E family member 2 |
| [Details](https://mirdb.org/cgi-bin/target_detail.cgi?targetID=2854955) | 1190 | 58 | hsa-miR-20a-5p | [PHF1](http://www.ncbi.nlm.nih.gov/entrez/query.fcgi?db=gene&cmd=Retrieve&dopt=full_report&list_uids=5252) | PHD finger protein 1 |
| [Details](https://mirdb.org/cgi-bin/target_detail.cgi?targetID=2854985) | 1191 | 58 | hsa-miR-20a-5p | [SLC5A3](http://www.ncbi.nlm.nih.gov/entrez/query.fcgi?db=gene&cmd=Retrieve&dopt=full_report&list_uids=6526) | solute carrier family 5 member 3 |
| [Details](https://mirdb.org/cgi-bin/target_detail.cgi?targetID=2855002) | 1192 | 58 | hsa-miR-20a-5p | [SLCO1C1](http://www.ncbi.nlm.nih.gov/entrez/query.fcgi?db=gene&cmd=Retrieve&dopt=full_report&list_uids=53919) | solute carrier organic anion transporter family member 1C1 |
| [Details](https://mirdb.org/cgi-bin/target_detail.cgi?targetID=2855053) | 1193 | 58 | hsa-miR-20a-5p | [FAM168A](http://www.ncbi.nlm.nih.gov/entrez/query.fcgi?db=gene&cmd=Retrieve&dopt=full_report&list_uids=23201) | family with sequence similarity 168 member A |
| [Details](https://mirdb.org/cgi-bin/target_detail.cgi?targetID=2855130) | 1194 | 58 | hsa-miR-20a-5p | [PPP1R1C](http://www.ncbi.nlm.nih.gov/entrez/query.fcgi?db=gene&cmd=Retrieve&dopt=full_report&list_uids=151242) | protein phosphatase 1 regulatory inhibitor subunit 1C |
| [Details](https://mirdb.org/cgi-bin/target_detail.cgi?targetID=2855157) | 1195 | 58 | hsa-miR-20a-5p | [GPR26](http://www.ncbi.nlm.nih.gov/entrez/query.fcgi?db=gene&cmd=Retrieve&dopt=full_report&list_uids=2849) | G protein-coupled receptor 26 |
| [Details](https://mirdb.org/cgi-bin/target_detail.cgi?targetID=2855206) | 1196 | 58 | hsa-miR-20a-5p | [ZIM3](http://www.ncbi.nlm.nih.gov/entrez/query.fcgi?db=gene&cmd=Retrieve&dopt=full_report&list_uids=114026) | zinc finger imprinted 3 |
| [Details](https://mirdb.org/cgi-bin/target_detail.cgi?targetID=2855211) | 1197 | 58 | hsa-miR-20a-5p | [TLE4](http://www.ncbi.nlm.nih.gov/entrez/query.fcgi?db=gene&cmd=Retrieve&dopt=full_report&list_uids=7091) | TLE family member 4, transcriptional corepressor |
| [Details](https://mirdb.org/cgi-bin/target_detail.cgi?targetID=2855241) | 1198 | 58 | hsa-miR-20a-5p | [AREL1](http://www.ncbi.nlm.nih.gov/entrez/query.fcgi?db=gene&cmd=Retrieve&dopt=full_report&list_uids=9870) | apoptosis resistant E3 ubiquitin protein ligase 1 |
| [Details](https://mirdb.org/cgi-bin/target_detail.cgi?targetID=2855253) | 1199 | 58 | hsa-miR-20a-5p | [ERBB3](http://www.ncbi.nlm.nih.gov/entrez/query.fcgi?db=gene&cmd=Retrieve&dopt=full_report&list_uids=2065) | erb-b2 receptor tyrosine kinase 3 |
| [Details](https://mirdb.org/cgi-bin/target_detail.cgi?targetID=2855391) | 1200 | 58 | hsa-miR-20a-5p | [ENTPD7](http://www.ncbi.nlm.nih.gov/entrez/query.fcgi?db=gene&cmd=Retrieve&dopt=full_report&list_uids=57089) | ectonucleoside triphosphate diphosphohydrolase 7 |
| [Details](https://mirdb.org/cgi-bin/target_detail.cgi?targetID=2855416) | 1201 | 58 | hsa-miR-20a-5p | [VEZF1](http://www.ncbi.nlm.nih.gov/entrez/query.fcgi?db=gene&cmd=Retrieve&dopt=full_report&list_uids=7716) | vascular endothelial zinc finger 1 |
| [Details](https://mirdb.org/cgi-bin/target_detail.cgi?targetID=2854341) | 1202 | 57 | hsa-miR-20a-5p | [SIRPA](http://www.ncbi.nlm.nih.gov/entrez/query.fcgi?db=gene&cmd=Retrieve&dopt=full_report&list_uids=140885) | signal regulatory protein alpha |
| [Details](https://mirdb.org/cgi-bin/target_detail.cgi?targetID=2854358) | 1203 | 57 | hsa-miR-20a-5p | [RCAN3](http://www.ncbi.nlm.nih.gov/entrez/query.fcgi?db=gene&cmd=Retrieve&dopt=full_report&list_uids=11123) | RCAN family member 3 |
| [Details](https://mirdb.org/cgi-bin/target_detail.cgi?targetID=2854446) | 1204 | 57 | hsa-miR-20a-5p | [SLC35E2A](http://www.ncbi.nlm.nih.gov/entrez/query.fcgi?db=gene&cmd=Retrieve&dopt=full_report&list_uids=9906) | solute carrier family 35 member E2A |
| [Details](https://mirdb.org/cgi-bin/target_detail.cgi?targetID=2854563) | 1205 | 57 | hsa-miR-20a-5p | [ZFP28](http://www.ncbi.nlm.nih.gov/entrez/query.fcgi?db=gene&cmd=Retrieve&dopt=full_report&list_uids=140612) | ZFP28 zinc finger protein |
| [Details](https://mirdb.org/cgi-bin/target_detail.cgi?targetID=2854584) | 1206 | 57 | hsa-miR-20a-5p | [TMUB2](http://www.ncbi.nlm.nih.gov/entrez/query.fcgi?db=gene&cmd=Retrieve&dopt=full_report&list_uids=79089) | transmembrane and ubiquitin like domain containing 2 |
| [Details](https://mirdb.org/cgi-bin/target_detail.cgi?targetID=2854609) | 1207 | 57 | hsa-miR-20a-5p | [RBM41](http://www.ncbi.nlm.nih.gov/entrez/query.fcgi?db=gene&cmd=Retrieve&dopt=full_report&list_uids=55285) | RNA binding motif protein 41 |
| [Details](https://mirdb.org/cgi-bin/target_detail.cgi?targetID=2854738) | 1208 | 57 | hsa-miR-20a-5p | [TMEM132B](http://www.ncbi.nlm.nih.gov/entrez/query.fcgi?db=gene&cmd=Retrieve&dopt=full_report&list_uids=114795) | transmembrane protein 132B |
| [Details](https://mirdb.org/cgi-bin/target_detail.cgi?targetID=2854798) | 1209 | 57 | hsa-miR-20a-5p | [XRRA1](http://www.ncbi.nlm.nih.gov/entrez/query.fcgi?db=gene&cmd=Retrieve&dopt=full_report&list_uids=143570) | X-ray radiation resistance associated 1 |
| [Details](https://mirdb.org/cgi-bin/target_detail.cgi?targetID=2854865) | 1210 | 57 | hsa-miR-20a-5p | [AGA](http://www.ncbi.nlm.nih.gov/entrez/query.fcgi?db=gene&cmd=Retrieve&dopt=full_report&list_uids=175) | aspartylglucosaminidase |
| [Details](https://mirdb.org/cgi-bin/target_detail.cgi?targetID=2854968) | 1211 | 57 | hsa-miR-20a-5p | [LTV1](http://www.ncbi.nlm.nih.gov/entrez/query.fcgi?db=gene&cmd=Retrieve&dopt=full_report&list_uids=84946) | LTV1 ribosome biogenesis factor |
| [Details](https://mirdb.org/cgi-bin/target_detail.cgi?targetID=2855015) | 1212 | 57 | hsa-miR-20a-5p | [UBASH3B](http://www.ncbi.nlm.nih.gov/entrez/query.fcgi?db=gene&cmd=Retrieve&dopt=full_report&list_uids=84959) | ubiquitin associated and SH3 domain containing B |
| [Details](https://mirdb.org/cgi-bin/target_detail.cgi?targetID=2855031) | 1213 | 57 | hsa-miR-20a-5p | [UBR1](http://www.ncbi.nlm.nih.gov/entrez/query.fcgi?db=gene&cmd=Retrieve&dopt=full_report&list_uids=197131) | ubiquitin protein ligase E3 component n-recognin 1 |
| [Details](https://mirdb.org/cgi-bin/target_detail.cgi?targetID=2855033) | 1214 | 57 | hsa-miR-20a-5p | [HIBCH](http://www.ncbi.nlm.nih.gov/entrez/query.fcgi?db=gene&cmd=Retrieve&dopt=full_report&list_uids=26275) | 3-hydroxyisobutyryl-CoA hydrolase |
| [Details](https://mirdb.org/cgi-bin/target_detail.cgi?targetID=2855051) | 1215 | 57 | hsa-miR-20a-5p | [EYS](http://www.ncbi.nlm.nih.gov/entrez/query.fcgi?db=gene&cmd=Retrieve&dopt=full_report&list_uids=346007) | eyes shut homolog |
| [Details](https://mirdb.org/cgi-bin/target_detail.cgi?targetID=2855092) | 1216 | 57 | hsa-miR-20a-5p | [PBLD](http://www.ncbi.nlm.nih.gov/entrez/query.fcgi?db=gene&cmd=Retrieve&dopt=full_report&list_uids=64081) | phenazine biosynthesis like protein domain containing |
| [Details](https://mirdb.org/cgi-bin/target_detail.cgi?targetID=2855121) | 1217 | 57 | hsa-miR-20a-5p | [SPACA4](http://www.ncbi.nlm.nih.gov/entrez/query.fcgi?db=gene&cmd=Retrieve&dopt=full_report&list_uids=171169) | sperm acrosome associated 4 |
| [Details](https://mirdb.org/cgi-bin/target_detail.cgi?targetID=2855198) | 1218 | 57 | hsa-miR-20a-5p | [ZNF780B](http://www.ncbi.nlm.nih.gov/entrez/query.fcgi?db=gene&cmd=Retrieve&dopt=full_report&list_uids=163131) | zinc finger protein 780B |
| [Details](https://mirdb.org/cgi-bin/target_detail.cgi?targetID=2855207) | 1219 | 57 | hsa-miR-20a-5p | [VPS26A](http://www.ncbi.nlm.nih.gov/entrez/query.fcgi?db=gene&cmd=Retrieve&dopt=full_report&list_uids=9559) | VPS26, retromer complex component A |
| [Details](https://mirdb.org/cgi-bin/target_detail.cgi?targetID=2855227) | 1220 | 57 | hsa-miR-20a-5p | [RARB](http://www.ncbi.nlm.nih.gov/entrez/query.fcgi?db=gene&cmd=Retrieve&dopt=full_report&list_uids=5915) | retinoic acid receptor beta |
| [Details](https://mirdb.org/cgi-bin/target_detail.cgi?targetID=2855340) | 1221 | 57 | hsa-miR-20a-5p | [TMED5](http://www.ncbi.nlm.nih.gov/entrez/query.fcgi?db=gene&cmd=Retrieve&dopt=full_report&list_uids=50999) | transmembrane p24 trafficking protein 5 |
| [Details](https://mirdb.org/cgi-bin/target_detail.cgi?targetID=2855352) | 1222 | 57 | hsa-miR-20a-5p | [MBD5](http://www.ncbi.nlm.nih.gov/entrez/query.fcgi?db=gene&cmd=Retrieve&dopt=full_report&list_uids=55777) | methyl-CpG binding domain protein 5 |
| [Details](https://mirdb.org/cgi-bin/target_detail.cgi?targetID=2855372) | 1223 | 57 | hsa-miR-20a-5p | [CYP20A1](http://www.ncbi.nlm.nih.gov/entrez/query.fcgi?db=gene&cmd=Retrieve&dopt=full_report&list_uids=57404) | cytochrome P450 family 20 subfamily A member 1 |
| [Details](https://mirdb.org/cgi-bin/target_detail.cgi?targetID=2855436) | 1224 | 57 | hsa-miR-20a-5p | [EAF1](http://www.ncbi.nlm.nih.gov/entrez/query.fcgi?db=gene&cmd=Retrieve&dopt=full_report&list_uids=85403) | ELL associated factor 1 |
| [Details](https://mirdb.org/cgi-bin/target_detail.cgi?targetID=2854101) | 1225 | 56 | hsa-miR-20a-5p | [MARVELD3](http://www.ncbi.nlm.nih.gov/entrez/query.fcgi?db=gene&cmd=Retrieve&dopt=full_report&list_uids=91862) | MARVEL domain containing 3 |
| [Details](https://mirdb.org/cgi-bin/target_detail.cgi?targetID=2854182) | 1226 | 56 | hsa-miR-20a-5p | [DPH6](http://www.ncbi.nlm.nih.gov/entrez/query.fcgi?db=gene&cmd=Retrieve&dopt=full_report&list_uids=89978) | diphthamine biosynthesis 6 |
| [Details](https://mirdb.org/cgi-bin/target_detail.cgi?targetID=2854186) | 1227 | 56 | hsa-miR-20a-5p | [KIF13A](http://www.ncbi.nlm.nih.gov/entrez/query.fcgi?db=gene&cmd=Retrieve&dopt=full_report&list_uids=63971) | kinesin family member 13A |
| [Details](https://mirdb.org/cgi-bin/target_detail.cgi?targetID=2854219) | 1228 | 56 | hsa-miR-20a-5p | [PDGFRB](http://www.ncbi.nlm.nih.gov/entrez/query.fcgi?db=gene&cmd=Retrieve&dopt=full_report&list_uids=5159) | platelet derived growth factor receptor beta |
| [Details](https://mirdb.org/cgi-bin/target_detail.cgi?targetID=2854256) | 1229 | 56 | hsa-miR-20a-5p | [RCOR1](http://www.ncbi.nlm.nih.gov/entrez/query.fcgi?db=gene&cmd=Retrieve&dopt=full_report&list_uids=23186) | REST corepressor 1 |
| [Details](https://mirdb.org/cgi-bin/target_detail.cgi?targetID=2854273) | 1230 | 56 | hsa-miR-20a-5p | [SPSB4](http://www.ncbi.nlm.nih.gov/entrez/query.fcgi?db=gene&cmd=Retrieve&dopt=full_report&list_uids=92369) | splA/ryanodine receptor domain and SOCS box containing 4 |
| [Details](https://mirdb.org/cgi-bin/target_detail.cgi?targetID=2854392) | 1231 | 56 | hsa-miR-20a-5p | [CEP126](http://www.ncbi.nlm.nih.gov/entrez/query.fcgi?db=gene&cmd=Retrieve&dopt=full_report&list_uids=57562) | centrosomal protein 126 |
| [Details](https://mirdb.org/cgi-bin/target_detail.cgi?targetID=2854404) | 1232 | 56 | hsa-miR-20a-5p | [C18orf32](http://www.ncbi.nlm.nih.gov/entrez/query.fcgi?db=gene&cmd=Retrieve&dopt=full_report&list_uids=497661) | chromosome 18 open reading frame 32 |
| [Details](https://mirdb.org/cgi-bin/target_detail.cgi?targetID=2854481) | 1233 | 56 | hsa-miR-20a-5p | [BOD1L1](http://www.ncbi.nlm.nih.gov/entrez/query.fcgi?db=gene&cmd=Retrieve&dopt=full_report&list_uids=259282) | biorientation of chromosomes in cell division 1 like 1 |
| [Details](https://mirdb.org/cgi-bin/target_detail.cgi?targetID=2854496) | 1234 | 56 | hsa-miR-20a-5p | [SLC39A6](http://www.ncbi.nlm.nih.gov/entrez/query.fcgi?db=gene&cmd=Retrieve&dopt=full_report&list_uids=25800) | solute carrier family 39 member 6 |
| [Details](https://mirdb.org/cgi-bin/target_detail.cgi?targetID=2854507) | 1235 | 56 | hsa-miR-20a-5p | [RD3](http://www.ncbi.nlm.nih.gov/entrez/query.fcgi?db=gene&cmd=Retrieve&dopt=full_report&list_uids=343035) | retinal degeneration 3, GUCY2D regulator |
| [Details](https://mirdb.org/cgi-bin/target_detail.cgi?targetID=2854582) | 1236 | 56 | hsa-miR-20a-5p | [PDS5A](http://www.ncbi.nlm.nih.gov/entrez/query.fcgi?db=gene&cmd=Retrieve&dopt=full_report&list_uids=23244) | PDS5 cohesin associated factor A |
| [Details](https://mirdb.org/cgi-bin/target_detail.cgi?targetID=2854617) | 1237 | 56 | hsa-miR-20a-5p | [FXN](http://www.ncbi.nlm.nih.gov/entrez/query.fcgi?db=gene&cmd=Retrieve&dopt=full_report&list_uids=2395) | frataxin |
| [Details](https://mirdb.org/cgi-bin/target_detail.cgi?targetID=2854777) | 1238 | 56 | hsa-miR-20a-5p | [INTS6](http://www.ncbi.nlm.nih.gov/entrez/query.fcgi?db=gene&cmd=Retrieve&dopt=full_report&list_uids=26512) | integrator complex subunit 6 |
| [Details](https://mirdb.org/cgi-bin/target_detail.cgi?targetID=2854864) | 1239 | 56 | hsa-miR-20a-5p | [PPM1A](http://www.ncbi.nlm.nih.gov/entrez/query.fcgi?db=gene&cmd=Retrieve&dopt=full_report&list_uids=5494) | protein phosphatase, Mg2+/Mn2+ dependent 1A |
| [Details](https://mirdb.org/cgi-bin/target_detail.cgi?targetID=2854981) | 1240 | 56 | hsa-miR-20a-5p | [SCN3A](http://www.ncbi.nlm.nih.gov/entrez/query.fcgi?db=gene&cmd=Retrieve&dopt=full_report&list_uids=6328) | sodium voltage-gated channel alpha subunit 3 |
| [Details](https://mirdb.org/cgi-bin/target_detail.cgi?targetID=2855000) | 1241 | 56 | hsa-miR-20a-5p | [IPO7](http://www.ncbi.nlm.nih.gov/entrez/query.fcgi?db=gene&cmd=Retrieve&dopt=full_report&list_uids=10527) | importin 7 |
| [Details](https://mirdb.org/cgi-bin/target_detail.cgi?targetID=2855024) | 1242 | 56 | hsa-miR-20a-5p | [COL4A2](http://www.ncbi.nlm.nih.gov/entrez/query.fcgi?db=gene&cmd=Retrieve&dopt=full_report&list_uids=1284) | collagen type IV alpha 2 chain |
| [Details](https://mirdb.org/cgi-bin/target_detail.cgi?targetID=2855027) | 1243 | 56 | hsa-miR-20a-5p | [RNF24](http://www.ncbi.nlm.nih.gov/entrez/query.fcgi?db=gene&cmd=Retrieve&dopt=full_report&list_uids=11237) | ring finger protein 24 |
| [Details](https://mirdb.org/cgi-bin/target_detail.cgi?targetID=2855050) | 1244 | 56 | hsa-miR-20a-5p | [DLC1](http://www.ncbi.nlm.nih.gov/entrez/query.fcgi?db=gene&cmd=Retrieve&dopt=full_report&list_uids=10395) | DLC1 Rho GTPase activating protein |
| [Details](https://mirdb.org/cgi-bin/target_detail.cgi?targetID=2855265) | 1245 | 56 | hsa-miR-20a-5p | [DDX46](http://www.ncbi.nlm.nih.gov/entrez/query.fcgi?db=gene&cmd=Retrieve&dopt=full_report&list_uids=9879) | DEAD-box helicase 46 |
| [Details](https://mirdb.org/cgi-bin/target_detail.cgi?targetID=2855389) | 1246 | 56 | hsa-miR-20a-5p | [AHRR](http://www.ncbi.nlm.nih.gov/entrez/query.fcgi?db=gene&cmd=Retrieve&dopt=full_report&list_uids=57491) | aryl-hydrocarbon receptor repressor |
| [Details](https://mirdb.org/cgi-bin/target_detail.cgi?targetID=2855400) | 1247 | 56 | hsa-miR-20a-5p | [CNN1](http://www.ncbi.nlm.nih.gov/entrez/query.fcgi?db=gene&cmd=Retrieve&dopt=full_report&list_uids=1264) | calponin 1 |
| [Details](https://mirdb.org/cgi-bin/target_detail.cgi?targetID=2855418) | 1248 | 56 | hsa-miR-20a-5p | [FOXL2](http://www.ncbi.nlm.nih.gov/entrez/query.fcgi?db=gene&cmd=Retrieve&dopt=full_report&list_uids=668) | forkhead box L2 |
| [Details](https://mirdb.org/cgi-bin/target_detail.cgi?targetID=2854117) | 1249 | 55 | hsa-miR-20a-5p | [PSD3](http://www.ncbi.nlm.nih.gov/entrez/query.fcgi?db=gene&cmd=Retrieve&dopt=full_report&list_uids=23362) | pleckstrin and Sec7 domain containing 3 |
| [Details](https://mirdb.org/cgi-bin/target_detail.cgi?targetID=2854132) | 1250 | 55 | hsa-miR-20a-5p | [ENSA](http://www.ncbi.nlm.nih.gov/entrez/query.fcgi?db=gene&cmd=Retrieve&dopt=full_report&list_uids=2029) | endosulfine alpha |
| [Details](https://mirdb.org/cgi-bin/target_detail.cgi?targetID=2854147) | 1251 | 55 | hsa-miR-20a-5p | [C1orf52](http://www.ncbi.nlm.nih.gov/entrez/query.fcgi?db=gene&cmd=Retrieve&dopt=full_report&list_uids=148423) | chromosome 1 open reading frame 52 |
| [Details](https://mirdb.org/cgi-bin/target_detail.cgi?targetID=2854162) | 1252 | 55 | hsa-miR-20a-5p | [PRR27](http://www.ncbi.nlm.nih.gov/entrez/query.fcgi?db=gene&cmd=Retrieve&dopt=full_report&list_uids=401137) | proline rich 27 |
| [Details](https://mirdb.org/cgi-bin/target_detail.cgi?targetID=2854192) | 1253 | 55 | hsa-miR-20a-5p | [KLK7](http://www.ncbi.nlm.nih.gov/entrez/query.fcgi?db=gene&cmd=Retrieve&dopt=full_report&list_uids=5650) | kallikrein related peptidase 7 |
| [Details](https://mirdb.org/cgi-bin/target_detail.cgi?targetID=2854241) | 1254 | 55 | hsa-miR-20a-5p | [PAX6](http://www.ncbi.nlm.nih.gov/entrez/query.fcgi?db=gene&cmd=Retrieve&dopt=full_report&list_uids=5080) | paired box 6 |
| [Details](https://mirdb.org/cgi-bin/target_detail.cgi?targetID=2854243) | 1255 | 55 | hsa-miR-20a-5p | [KIF14](http://www.ncbi.nlm.nih.gov/entrez/query.fcgi?db=gene&cmd=Retrieve&dopt=full_report&list_uids=9928) | kinesin family member 14 |
| [Details](https://mirdb.org/cgi-bin/target_detail.cgi?targetID=2854316) | 1256 | 55 | hsa-miR-20a-5p | [HARS](http://www.ncbi.nlm.nih.gov/entrez/query.fcgi?db=gene&cmd=Retrieve&dopt=full_report&list_uids=3035) | histidyl-tRNA synthetase |
| [Details](https://mirdb.org/cgi-bin/target_detail.cgi?targetID=2854319) | 1257 | 55 | hsa-miR-20a-5p | [GNB4](http://www.ncbi.nlm.nih.gov/entrez/query.fcgi?db=gene&cmd=Retrieve&dopt=full_report&list_uids=59345) | G protein subunit beta 4 |
| [Details](https://mirdb.org/cgi-bin/target_detail.cgi?targetID=2854424) | 1258 | 55 | hsa-miR-20a-5p | [RUBCN](http://www.ncbi.nlm.nih.gov/entrez/query.fcgi?db=gene&cmd=Retrieve&dopt=full_report&list_uids=9711) | rubicon autophagy regulator |
| [Details](https://mirdb.org/cgi-bin/target_detail.cgi?targetID=2854510) | 1259 | 55 | hsa-miR-20a-5p | [ASB1](http://www.ncbi.nlm.nih.gov/entrez/query.fcgi?db=gene&cmd=Retrieve&dopt=full_report&list_uids=51665) | ankyrin repeat and SOCS box containing 1 |
| [Details](https://mirdb.org/cgi-bin/target_detail.cgi?targetID=2854564) | 1260 | 55 | hsa-miR-20a-5p | [SMAD6](http://www.ncbi.nlm.nih.gov/entrez/query.fcgi?db=gene&cmd=Retrieve&dopt=full_report&list_uids=4091) | SMAD family member 6 |
| [Details](https://mirdb.org/cgi-bin/target_detail.cgi?targetID=2854600) | 1261 | 55 | hsa-miR-20a-5p | [TMCC1](http://www.ncbi.nlm.nih.gov/entrez/query.fcgi?db=gene&cmd=Retrieve&dopt=full_report&list_uids=23023) | transmembrane and coiled-coil domain family 1 |
| [Details](https://mirdb.org/cgi-bin/target_detail.cgi?targetID=2854662) | 1262 | 55 | hsa-miR-20a-5p | [TCF4](http://www.ncbi.nlm.nih.gov/entrez/query.fcgi?db=gene&cmd=Retrieve&dopt=full_report&list_uids=6925) | transcription factor 4 |
| [Details](https://mirdb.org/cgi-bin/target_detail.cgi?targetID=2854670) | 1263 | 55 | hsa-miR-20a-5p | [SIRT5](http://www.ncbi.nlm.nih.gov/entrez/query.fcgi?db=gene&cmd=Retrieve&dopt=full_report&list_uids=23408) | sirtuin 5 |
| [Details](https://mirdb.org/cgi-bin/target_detail.cgi?targetID=2854729) | 1264 | 55 | hsa-miR-20a-5p | [EXPH5](http://www.ncbi.nlm.nih.gov/entrez/query.fcgi?db=gene&cmd=Retrieve&dopt=full_report&list_uids=23086) | exophilin 5 |
| [Details](https://mirdb.org/cgi-bin/target_detail.cgi?targetID=2854758) | 1265 | 55 | hsa-miR-20a-5p | [F2RL2](http://www.ncbi.nlm.nih.gov/entrez/query.fcgi?db=gene&cmd=Retrieve&dopt=full_report&list_uids=2151) | coagulation factor II thrombin receptor like 2 |
| [Details](https://mirdb.org/cgi-bin/target_detail.cgi?targetID=2854793) | 1266 | 55 | hsa-miR-20a-5p | [SNX12](http://www.ncbi.nlm.nih.gov/entrez/query.fcgi?db=gene&cmd=Retrieve&dopt=full_report&list_uids=29934) | sorting nexin 12 |
| [Details](https://mirdb.org/cgi-bin/target_detail.cgi?targetID=2854816) | 1267 | 55 | hsa-miR-20a-5p | [RPS6KA2](http://www.ncbi.nlm.nih.gov/entrez/query.fcgi?db=gene&cmd=Retrieve&dopt=full_report&list_uids=6196) | ribosomal protein S6 kinase A2 |
| [Details](https://mirdb.org/cgi-bin/target_detail.cgi?targetID=2854841) | 1268 | 55 | hsa-miR-20a-5p | [VDAC1](http://www.ncbi.nlm.nih.gov/entrez/query.fcgi?db=gene&cmd=Retrieve&dopt=full_report&list_uids=7416) | voltage dependent anion channel 1 |
| [Details](https://mirdb.org/cgi-bin/target_detail.cgi?targetID=2854899) | 1269 | 55 | hsa-miR-20a-5p | [ABT1](http://www.ncbi.nlm.nih.gov/entrez/query.fcgi?db=gene&cmd=Retrieve&dopt=full_report&list_uids=29777) | activator of basal transcription 1 |
| [Details](https://mirdb.org/cgi-bin/target_detail.cgi?targetID=2855062) | 1270 | 55 | hsa-miR-20a-5p | [ENTPD5](http://www.ncbi.nlm.nih.gov/entrez/query.fcgi?db=gene&cmd=Retrieve&dopt=full_report&list_uids=957) | ectonucleoside triphosphate diphosphohydrolase 5 |
| [Details](https://mirdb.org/cgi-bin/target_detail.cgi?targetID=2855069) | 1271 | 55 | hsa-miR-20a-5p | [ADAMTSL5](http://www.ncbi.nlm.nih.gov/entrez/query.fcgi?db=gene&cmd=Retrieve&dopt=full_report&list_uids=339366) | ADAMTS like 5 |
| [Details](https://mirdb.org/cgi-bin/target_detail.cgi?targetID=2855075) | 1272 | 55 | hsa-miR-20a-5p | [PTPRO](http://www.ncbi.nlm.nih.gov/entrez/query.fcgi?db=gene&cmd=Retrieve&dopt=full_report&list_uids=5800) | protein tyrosine phosphatase, receptor type O |
| [Details](https://mirdb.org/cgi-bin/target_detail.cgi?targetID=2855284) | 1273 | 55 | hsa-miR-20a-5p | [RPGR](http://www.ncbi.nlm.nih.gov/entrez/query.fcgi?db=gene&cmd=Retrieve&dopt=full_report&list_uids=6103) | retinitis pigmentosa GTPase regulator |
| [Details](https://mirdb.org/cgi-bin/target_detail.cgi?targetID=2855304) | 1274 | 55 | hsa-miR-20a-5p | [ATE1](http://www.ncbi.nlm.nih.gov/entrez/query.fcgi?db=gene&cmd=Retrieve&dopt=full_report&list_uids=11101) | arginyltransferase 1 |
| [Details](https://mirdb.org/cgi-bin/target_detail.cgi?targetID=2855325) | 1275 | 55 | hsa-miR-20a-5p | [MAVS](http://www.ncbi.nlm.nih.gov/entrez/query.fcgi?db=gene&cmd=Retrieve&dopt=full_report&list_uids=57506) | mitochondrial antiviral signaling protein |
| [Details](https://mirdb.org/cgi-bin/target_detail.cgi?targetID=2855332) | 1276 | 55 | hsa-miR-20a-5p | [RPL17-C18orf32](http://www.ncbi.nlm.nih.gov/entrez/query.fcgi?db=gene&cmd=Retrieve&dopt=full_report&list_uids=100526842) | RPL17-C18orf32 readthrough |
| [Details](https://mirdb.org/cgi-bin/target_detail.cgi?targetID=2855435) | 1277 | 55 | hsa-miR-20a-5p | [MBTPS1](http://www.ncbi.nlm.nih.gov/entrez/query.fcgi?db=gene&cmd=Retrieve&dopt=full_report&list_uids=8720) | membrane bound transcription factor peptidase, site 1 |
| [Details](https://mirdb.org/cgi-bin/target_detail.cgi?targetID=2854160) | 1278 | 54 | hsa-miR-20a-5p | [SEC22C](http://www.ncbi.nlm.nih.gov/entrez/query.fcgi?db=gene&cmd=Retrieve&dopt=full_report&list_uids=9117) | SEC22 homolog C, vesicle trafficking protein |
| [Details](https://mirdb.org/cgi-bin/target_detail.cgi?targetID=2854236) | 1279 | 54 | hsa-miR-20a-5p | [RSAD2](http://www.ncbi.nlm.nih.gov/entrez/query.fcgi?db=gene&cmd=Retrieve&dopt=full_report&list_uids=91543) | radical S-adenosyl methionine domain containing 2 |
| [Details](https://mirdb.org/cgi-bin/target_detail.cgi?targetID=2854248) | 1280 | 54 | hsa-miR-20a-5p | [TOLLIP](http://www.ncbi.nlm.nih.gov/entrez/query.fcgi?db=gene&cmd=Retrieve&dopt=full_report&list_uids=54472) | toll interacting protein |
| [Details](https://mirdb.org/cgi-bin/target_detail.cgi?targetID=2854274) | 1281 | 54 | hsa-miR-20a-5p | [C16orf72](http://www.ncbi.nlm.nih.gov/entrez/query.fcgi?db=gene&cmd=Retrieve&dopt=full_report&list_uids=29035) | chromosome 16 open reading frame 72 |
| [Details](https://mirdb.org/cgi-bin/target_detail.cgi?targetID=2854363) | 1282 | 54 | hsa-miR-20a-5p | [SPART](http://www.ncbi.nlm.nih.gov/entrez/query.fcgi?db=gene&cmd=Retrieve&dopt=full_report&list_uids=23111) | spartin |
| [Details](https://mirdb.org/cgi-bin/target_detail.cgi?targetID=2854411) | 1283 | 54 | hsa-miR-20a-5p | [MRTFB](http://www.ncbi.nlm.nih.gov/entrez/query.fcgi?db=gene&cmd=Retrieve&dopt=full_report&list_uids=57496) | myocardin related transcription factor B |
| [Details](https://mirdb.org/cgi-bin/target_detail.cgi?targetID=2854479) | 1284 | 54 | hsa-miR-20a-5p | [ELAVL2](http://www.ncbi.nlm.nih.gov/entrez/query.fcgi?db=gene&cmd=Retrieve&dopt=full_report&list_uids=1993) | ELAV like RNA binding protein 2 |
| [Details](https://mirdb.org/cgi-bin/target_detail.cgi?targetID=2854502) | 1285 | 54 | hsa-miR-20a-5p | [DSTYK](http://www.ncbi.nlm.nih.gov/entrez/query.fcgi?db=gene&cmd=Retrieve&dopt=full_report&list_uids=25778) | dual serine/threonine and tyrosine protein kinase |
| [Details](https://mirdb.org/cgi-bin/target_detail.cgi?targetID=2854543) | 1286 | 54 | hsa-miR-20a-5p | [C6orf141](http://www.ncbi.nlm.nih.gov/entrez/query.fcgi?db=gene&cmd=Retrieve&dopt=full_report&list_uids=135398) | chromosome 6 open reading frame 141 |
| [Details](https://mirdb.org/cgi-bin/target_detail.cgi?targetID=2854572) | 1287 | 54 | hsa-miR-20a-5p | [MAST3](http://www.ncbi.nlm.nih.gov/entrez/query.fcgi?db=gene&cmd=Retrieve&dopt=full_report&list_uids=23031) | microtubule associated serine/threonine kinase 3 |
| [Details](https://mirdb.org/cgi-bin/target_detail.cgi?targetID=2854593) | 1288 | 54 | hsa-miR-20a-5p | [GJA1](http://www.ncbi.nlm.nih.gov/entrez/query.fcgi?db=gene&cmd=Retrieve&dopt=full_report&list_uids=2697) | gap junction protein alpha 1 |
| [Details](https://mirdb.org/cgi-bin/target_detail.cgi?targetID=2854615) | 1289 | 54 | hsa-miR-20a-5p | [BCL2L15](http://www.ncbi.nlm.nih.gov/entrez/query.fcgi?db=gene&cmd=Retrieve&dopt=full_report&list_uids=440603) | BCL2 like 15 |
| [Details](https://mirdb.org/cgi-bin/target_detail.cgi?targetID=2854644) | 1290 | 54 | hsa-miR-20a-5p | [OLIG1](http://www.ncbi.nlm.nih.gov/entrez/query.fcgi?db=gene&cmd=Retrieve&dopt=full_report&list_uids=116448) | oligodendrocyte transcription factor 1 |
| [Details](https://mirdb.org/cgi-bin/target_detail.cgi?targetID=2854683) | 1291 | 54 | hsa-miR-20a-5p | [PPARD](http://www.ncbi.nlm.nih.gov/entrez/query.fcgi?db=gene&cmd=Retrieve&dopt=full_report&list_uids=5467) | peroxisome proliferator activated receptor delta |
| [Details](https://mirdb.org/cgi-bin/target_detail.cgi?targetID=2854832) | 1292 | 54 | hsa-miR-20a-5p | [NTRK3](http://www.ncbi.nlm.nih.gov/entrez/query.fcgi?db=gene&cmd=Retrieve&dopt=full_report&list_uids=4916) | neurotrophic receptor tyrosine kinase 3 |
| [Details](https://mirdb.org/cgi-bin/target_detail.cgi?targetID=2854854) | 1293 | 54 | hsa-miR-20a-5p | [SH3TC2](http://www.ncbi.nlm.nih.gov/entrez/query.fcgi?db=gene&cmd=Retrieve&dopt=full_report&list_uids=79628) | SH3 domain and tetratricopeptide repeats 2 |
| [Details](https://mirdb.org/cgi-bin/target_detail.cgi?targetID=2854982) | 1294 | 54 | hsa-miR-20a-5p | [MCM3](http://www.ncbi.nlm.nih.gov/entrez/query.fcgi?db=gene&cmd=Retrieve&dopt=full_report&list_uids=4172) | minichromosome maintenance complex component 3 |
| [Details](https://mirdb.org/cgi-bin/target_detail.cgi?targetID=2855117) | 1295 | 54 | hsa-miR-20a-5p | [UBE2W](http://www.ncbi.nlm.nih.gov/entrez/query.fcgi?db=gene&cmd=Retrieve&dopt=full_report&list_uids=55284) | ubiquitin conjugating enzyme E2 W |
| [Details](https://mirdb.org/cgi-bin/target_detail.cgi?targetID=2855147) | 1296 | 54 | hsa-miR-20a-5p | [BTBD9](http://www.ncbi.nlm.nih.gov/entrez/query.fcgi?db=gene&cmd=Retrieve&dopt=full_report&list_uids=114781) | BTB domain containing 9 |
| [Details](https://mirdb.org/cgi-bin/target_detail.cgi?targetID=2855235) | 1297 | 54 | hsa-miR-20a-5p | [GKAP1](http://www.ncbi.nlm.nih.gov/entrez/query.fcgi?db=gene&cmd=Retrieve&dopt=full_report&list_uids=80318) | G kinase anchoring protein 1 |
| [Details](https://mirdb.org/cgi-bin/target_detail.cgi?targetID=2855343) | 1298 | 54 | hsa-miR-20a-5p | [IQSEC1](http://www.ncbi.nlm.nih.gov/entrez/query.fcgi?db=gene&cmd=Retrieve&dopt=full_report&list_uids=9922) | IQ motif and Sec7 domain 1 |
| [Details](https://mirdb.org/cgi-bin/target_detail.cgi?targetID=2855371) | 1299 | 54 | hsa-miR-20a-5p | [FGF12](http://www.ncbi.nlm.nih.gov/entrez/query.fcgi?db=gene&cmd=Retrieve&dopt=full_report&list_uids=2257) | fibroblast growth factor 12 |
| [Details](https://mirdb.org/cgi-bin/target_detail.cgi?targetID=2854106) | 1300 | 53 | hsa-miR-20a-5p | [MICU3](http://www.ncbi.nlm.nih.gov/entrez/query.fcgi?db=gene&cmd=Retrieve&dopt=full_report&list_uids=286097) | mitochondrial calcium uptake family member 3 |
| [Details](https://mirdb.org/cgi-bin/target_detail.cgi?targetID=2854199) | 1301 | 53 | hsa-miR-20a-5p | [KRT23](http://www.ncbi.nlm.nih.gov/entrez/query.fcgi?db=gene&cmd=Retrieve&dopt=full_report&list_uids=25984) | keratin 23 |
| [Details](https://mirdb.org/cgi-bin/target_detail.cgi?targetID=2854217) | 1302 | 53 | hsa-miR-20a-5p | [ZFYVE16](http://www.ncbi.nlm.nih.gov/entrez/query.fcgi?db=gene&cmd=Retrieve&dopt=full_report&list_uids=9765) | zinc finger FYVE-type containing 16 |
| [Details](https://mirdb.org/cgi-bin/target_detail.cgi?targetID=2854266) | 1303 | 53 | hsa-miR-20a-5p | [PLEKHM3](http://www.ncbi.nlm.nih.gov/entrez/query.fcgi?db=gene&cmd=Retrieve&dopt=full_report&list_uids=389072) | pleckstrin homology domain containing M3 |
| [Details](https://mirdb.org/cgi-bin/target_detail.cgi?targetID=2854353) | 1304 | 53 | hsa-miR-20a-5p | [C11orf58](http://www.ncbi.nlm.nih.gov/entrez/query.fcgi?db=gene&cmd=Retrieve&dopt=full_report&list_uids=10944) | chromosome 11 open reading frame 58 |
| [Details](https://mirdb.org/cgi-bin/target_detail.cgi?targetID=2854613) | 1305 | 53 | hsa-miR-20a-5p | [MFSD9](http://www.ncbi.nlm.nih.gov/entrez/query.fcgi?db=gene&cmd=Retrieve&dopt=full_report&list_uids=84804) | major facilitator superfamily domain containing 9 |
| [Details](https://mirdb.org/cgi-bin/target_detail.cgi?targetID=2854636) | 1306 | 53 | hsa-miR-20a-5p | [GARS](http://www.ncbi.nlm.nih.gov/entrez/query.fcgi?db=gene&cmd=Retrieve&dopt=full_report&list_uids=2617) | glycyl-tRNA synthetase |
| [Details](https://mirdb.org/cgi-bin/target_detail.cgi?targetID=2854686) | 1307 | 53 | hsa-miR-20a-5p | [PAX8](http://www.ncbi.nlm.nih.gov/entrez/query.fcgi?db=gene&cmd=Retrieve&dopt=full_report&list_uids=7849) | paired box 8 |
| [Details](https://mirdb.org/cgi-bin/target_detail.cgi?targetID=2854881) | 1308 | 53 | hsa-miR-20a-5p | [RMDN3](http://www.ncbi.nlm.nih.gov/entrez/query.fcgi?db=gene&cmd=Retrieve&dopt=full_report&list_uids=55177) | regulator of microtubule dynamics 3 |
| [Details](https://mirdb.org/cgi-bin/target_detail.cgi?targetID=2854930) | 1309 | 53 | hsa-miR-20a-5p | [UBASH3A](http://www.ncbi.nlm.nih.gov/entrez/query.fcgi?db=gene&cmd=Retrieve&dopt=full_report&list_uids=53347) | ubiquitin associated and SH3 domain containing A |
| [Details](https://mirdb.org/cgi-bin/target_detail.cgi?targetID=2854932) | 1310 | 53 | hsa-miR-20a-5p | [MTA3](http://www.ncbi.nlm.nih.gov/entrez/query.fcgi?db=gene&cmd=Retrieve&dopt=full_report&list_uids=57504) | metastasis associated 1 family member 3 |
| [Details](https://mirdb.org/cgi-bin/target_detail.cgi?targetID=2854946) | 1311 | 53 | hsa-miR-20a-5p | [IPCEF1](http://www.ncbi.nlm.nih.gov/entrez/query.fcgi?db=gene&cmd=Retrieve&dopt=full_report&list_uids=26034) | interaction protein for cytohesin exchange factors 1 |
| [Details](https://mirdb.org/cgi-bin/target_detail.cgi?targetID=2854961) | 1312 | 53 | hsa-miR-20a-5p | [DCUN1D5](http://www.ncbi.nlm.nih.gov/entrez/query.fcgi?db=gene&cmd=Retrieve&dopt=full_report&list_uids=84259) | defective in cullin neddylation 1 domain containing 5 |
| [Details](https://mirdb.org/cgi-bin/target_detail.cgi?targetID=2854970) | 1313 | 53 | hsa-miR-20a-5p | [C9orf72](http://www.ncbi.nlm.nih.gov/entrez/query.fcgi?db=gene&cmd=Retrieve&dopt=full_report&list_uids=203228) | chromosome 9 open reading frame 72 |
| [Details](https://mirdb.org/cgi-bin/target_detail.cgi?targetID=2854986) | 1314 | 53 | hsa-miR-20a-5p | [TAOK2](http://www.ncbi.nlm.nih.gov/entrez/query.fcgi?db=gene&cmd=Retrieve&dopt=full_report&list_uids=9344) | TAO kinase 2 |
| [Details](https://mirdb.org/cgi-bin/target_detail.cgi?targetID=2855097) | 1315 | 53 | hsa-miR-20a-5p | [CACNB4](http://www.ncbi.nlm.nih.gov/entrez/query.fcgi?db=gene&cmd=Retrieve&dopt=full_report&list_uids=785) | calcium voltage-gated channel auxiliary subunit beta 4 |
| [Details](https://mirdb.org/cgi-bin/target_detail.cgi?targetID=2855124) | 1316 | 53 | hsa-miR-20a-5p | [MAP3K3](http://www.ncbi.nlm.nih.gov/entrez/query.fcgi?db=gene&cmd=Retrieve&dopt=full_report&list_uids=4215) | mitogen-activated protein kinase kinase kinase 3 |
| [Details](https://mirdb.org/cgi-bin/target_detail.cgi?targetID=2855127) | 1317 | 53 | hsa-miR-20a-5p | [TES](http://www.ncbi.nlm.nih.gov/entrez/query.fcgi?db=gene&cmd=Retrieve&dopt=full_report&list_uids=26136) | testin LIM domain protein |
| [Details](https://mirdb.org/cgi-bin/target_detail.cgi?targetID=2855139) | 1318 | 53 | hsa-miR-20a-5p | [SNX9](http://www.ncbi.nlm.nih.gov/entrez/query.fcgi?db=gene&cmd=Retrieve&dopt=full_report&list_uids=51429) | sorting nexin 9 |
| [Details](https://mirdb.org/cgi-bin/target_detail.cgi?targetID=2855159) | 1319 | 53 | hsa-miR-20a-5p | [SLC25A43](http://www.ncbi.nlm.nih.gov/entrez/query.fcgi?db=gene&cmd=Retrieve&dopt=full_report&list_uids=203427) | solute carrier family 25 member 43 |
| [Details](https://mirdb.org/cgi-bin/target_detail.cgi?targetID=2855184) | 1320 | 53 | hsa-miR-20a-5p | [CCP110](http://www.ncbi.nlm.nih.gov/entrez/query.fcgi?db=gene&cmd=Retrieve&dopt=full_report&list_uids=9738) | centriolar coiled-coil protein 110 |
| [Details](https://mirdb.org/cgi-bin/target_detail.cgi?targetID=2855330) | 1321 | 53 | hsa-miR-20a-5p | [PLEKHG1](http://www.ncbi.nlm.nih.gov/entrez/query.fcgi?db=gene&cmd=Retrieve&dopt=full_report&list_uids=57480) | pleckstrin homology and RhoGEF domain containing G1 |
| [Details](https://mirdb.org/cgi-bin/target_detail.cgi?targetID=2855444) | 1322 | 53 | hsa-miR-20a-5p | [SP8](http://www.ncbi.nlm.nih.gov/entrez/query.fcgi?db=gene&cmd=Retrieve&dopt=full_report&list_uids=221833) | Sp8 transcription factor |
| [Details](https://mirdb.org/cgi-bin/target_detail.cgi?targetID=2854176) | 1323 | 52 | hsa-miR-20a-5p | [UCP3](http://www.ncbi.nlm.nih.gov/entrez/query.fcgi?db=gene&cmd=Retrieve&dopt=full_report&list_uids=7352) | uncoupling protein 3 |
| [Details](https://mirdb.org/cgi-bin/target_detail.cgi?targetID=2854181) | 1324 | 52 | hsa-miR-20a-5p | [NBEAL1](http://www.ncbi.nlm.nih.gov/entrez/query.fcgi?db=gene&cmd=Retrieve&dopt=full_report&list_uids=65065) | neurobeachin like 1 |
| [Details](https://mirdb.org/cgi-bin/target_detail.cgi?targetID=2854198) | 1325 | 52 | hsa-miR-20a-5p | [PRR11](http://www.ncbi.nlm.nih.gov/entrez/query.fcgi?db=gene&cmd=Retrieve&dopt=full_report&list_uids=55771) | proline rich 11 |
| [Details](https://mirdb.org/cgi-bin/target_detail.cgi?targetID=2854245) | 1326 | 52 | hsa-miR-20a-5p | [RFK](http://www.ncbi.nlm.nih.gov/entrez/query.fcgi?db=gene&cmd=Retrieve&dopt=full_report&list_uids=55312) | riboflavin kinase |
| [Details](https://mirdb.org/cgi-bin/target_detail.cgi?targetID=2854265) | 1327 | 52 | hsa-miR-20a-5p | [SPECC1](http://www.ncbi.nlm.nih.gov/entrez/query.fcgi?db=gene&cmd=Retrieve&dopt=full_report&list_uids=92521) | sperm antigen with calponin homology and coiled-coil domains 1 |
| [Details](https://mirdb.org/cgi-bin/target_detail.cgi?targetID=2854278) | 1328 | 52 | hsa-miR-20a-5p | [ZNF502](http://www.ncbi.nlm.nih.gov/entrez/query.fcgi?db=gene&cmd=Retrieve&dopt=full_report&list_uids=91392) | zinc finger protein 502 |
| [Details](https://mirdb.org/cgi-bin/target_detail.cgi?targetID=2854290) | 1329 | 52 | hsa-miR-20a-5p | [IPO5](http://www.ncbi.nlm.nih.gov/entrez/query.fcgi?db=gene&cmd=Retrieve&dopt=full_report&list_uids=3843) | importin 5 |
| [Details](https://mirdb.org/cgi-bin/target_detail.cgi?targetID=2854295) | 1330 | 52 | hsa-miR-20a-5p | [RASL12](http://www.ncbi.nlm.nih.gov/entrez/query.fcgi?db=gene&cmd=Retrieve&dopt=full_report&list_uids=51285) | RAS like family 12 |
| [Details](https://mirdb.org/cgi-bin/target_detail.cgi?targetID=2854324) | 1331 | 52 | hsa-miR-20a-5p | [AFF1](http://www.ncbi.nlm.nih.gov/entrez/query.fcgi?db=gene&cmd=Retrieve&dopt=full_report&list_uids=4299) | AF4/FMR2 family member 1 |
| [Details](https://mirdb.org/cgi-bin/target_detail.cgi?targetID=2854343) | 1332 | 52 | hsa-miR-20a-5p | [ZNF561](http://www.ncbi.nlm.nih.gov/entrez/query.fcgi?db=gene&cmd=Retrieve&dopt=full_report&list_uids=93134) | zinc finger protein 561 |
| [Details](https://mirdb.org/cgi-bin/target_detail.cgi?targetID=2854578) | 1333 | 52 | hsa-miR-20a-5p | [CTDSPL2](http://www.ncbi.nlm.nih.gov/entrez/query.fcgi?db=gene&cmd=Retrieve&dopt=full_report&list_uids=51496) | CTD small phosphatase like 2 |
| [Details](https://mirdb.org/cgi-bin/target_detail.cgi?targetID=2854720) | 1334 | 52 | hsa-miR-20a-5p | [SYT10](http://www.ncbi.nlm.nih.gov/entrez/query.fcgi?db=gene&cmd=Retrieve&dopt=full_report&list_uids=341359) | synaptotagmin 10 |
| [Details](https://mirdb.org/cgi-bin/target_detail.cgi?targetID=2854810) | 1335 | 52 | hsa-miR-20a-5p | [MICA](http://www.ncbi.nlm.nih.gov/entrez/query.fcgi?db=gene&cmd=Retrieve&dopt=full_report&list_uids=100507436) | MHC class I polypeptide-related sequence A |
| [Details](https://mirdb.org/cgi-bin/target_detail.cgi?targetID=2854813) | 1336 | 52 | hsa-miR-20a-5p | [NENF](http://www.ncbi.nlm.nih.gov/entrez/query.fcgi?db=gene&cmd=Retrieve&dopt=full_report&list_uids=29937) | neudesin neurotrophic factor |
| [Details](https://mirdb.org/cgi-bin/target_detail.cgi?targetID=2854874) | 1337 | 52 | hsa-miR-20a-5p | [PHF6](http://www.ncbi.nlm.nih.gov/entrez/query.fcgi?db=gene&cmd=Retrieve&dopt=full_report&list_uids=84295) | PHD finger protein 6 |
| [Details](https://mirdb.org/cgi-bin/target_detail.cgi?targetID=2854900) | 1338 | 52 | hsa-miR-20a-5p | [PDGFC](http://www.ncbi.nlm.nih.gov/entrez/query.fcgi?db=gene&cmd=Retrieve&dopt=full_report&list_uids=56034) | platelet derived growth factor C |
| [Details](https://mirdb.org/cgi-bin/target_detail.cgi?targetID=2854938) | 1339 | 52 | hsa-miR-20a-5p | [CAMK2N1](http://www.ncbi.nlm.nih.gov/entrez/query.fcgi?db=gene&cmd=Retrieve&dopt=full_report&list_uids=55450) | calcium/calmodulin dependent protein kinase II inhibitor 1 |
| [Details](https://mirdb.org/cgi-bin/target_detail.cgi?targetID=2854972) | 1340 | 52 | hsa-miR-20a-5p | [MYO10](http://www.ncbi.nlm.nih.gov/entrez/query.fcgi?db=gene&cmd=Retrieve&dopt=full_report&list_uids=4651) | myosin X |
| [Details](https://mirdb.org/cgi-bin/target_detail.cgi?targetID=2855001) | 1341 | 52 | hsa-miR-20a-5p | [MS4A14](http://www.ncbi.nlm.nih.gov/entrez/query.fcgi?db=gene&cmd=Retrieve&dopt=full_report&list_uids=84689) | membrane spanning 4-domains A14 |
| [Details](https://mirdb.org/cgi-bin/target_detail.cgi?targetID=2855019) | 1342 | 52 | hsa-miR-20a-5p | [PLPPR5](http://www.ncbi.nlm.nih.gov/entrez/query.fcgi?db=gene&cmd=Retrieve&dopt=full_report&list_uids=163404) | phospholipid phosphatase related 5 |
| [Details](https://mirdb.org/cgi-bin/target_detail.cgi?targetID=2855041) | 1343 | 52 | hsa-miR-20a-5p | [PPP2R3A](http://www.ncbi.nlm.nih.gov/entrez/query.fcgi?db=gene&cmd=Retrieve&dopt=full_report&list_uids=5523) | protein phosphatase 2 regulatory subunit B''alpha |
| [Details](https://mirdb.org/cgi-bin/target_detail.cgi?targetID=2855044) | 1344 | 52 | hsa-miR-20a-5p | [LITAF](http://www.ncbi.nlm.nih.gov/entrez/query.fcgi?db=gene&cmd=Retrieve&dopt=full_report&list_uids=9516) | lipopolysaccharide induced TNF factor |
| [Details](https://mirdb.org/cgi-bin/target_detail.cgi?targetID=2855065) | 1345 | 52 | hsa-miR-20a-5p | [SIK3](http://www.ncbi.nlm.nih.gov/entrez/query.fcgi?db=gene&cmd=Retrieve&dopt=full_report&list_uids=23387) | SIK family kinase 3 |
| [Details](https://mirdb.org/cgi-bin/target_detail.cgi?targetID=2855089) | 1346 | 52 | hsa-miR-20a-5p | [ATP8B1](http://www.ncbi.nlm.nih.gov/entrez/query.fcgi?db=gene&cmd=Retrieve&dopt=full_report&list_uids=5205) | ATPase phospholipid transporting 8B1 |
| [Details](https://mirdb.org/cgi-bin/target_detail.cgi?targetID=2855212) | 1347 | 52 | hsa-miR-20a-5p | [AP4S1](http://www.ncbi.nlm.nih.gov/entrez/query.fcgi?db=gene&cmd=Retrieve&dopt=full_report&list_uids=11154) | adaptor related protein complex 4 subunit sigma 1 |
| [Details](https://mirdb.org/cgi-bin/target_detail.cgi?targetID=2855229) | 1348 | 52 | hsa-miR-20a-5p | [ZDHHC20](http://www.ncbi.nlm.nih.gov/entrez/query.fcgi?db=gene&cmd=Retrieve&dopt=full_report&list_uids=253832) | zinc finger DHHC-type containing 20 |
| [Details](https://mirdb.org/cgi-bin/target_detail.cgi?targetID=2855375) | 1349 | 52 | hsa-miR-20a-5p | [USP53](http://www.ncbi.nlm.nih.gov/entrez/query.fcgi?db=gene&cmd=Retrieve&dopt=full_report&list_uids=54532) | ubiquitin specific peptidase 53 |
| [Details](https://mirdb.org/cgi-bin/target_detail.cgi?targetID=2854129) | 1350 | 51 | hsa-miR-20a-5p | [PLEKHM1](http://www.ncbi.nlm.nih.gov/entrez/query.fcgi?db=gene&cmd=Retrieve&dopt=full_report&list_uids=9842) | pleckstrin homology and RUN domain containing M1 |
| [Details](https://mirdb.org/cgi-bin/target_detail.cgi?targetID=2854130) | 1351 | 51 | hsa-miR-20a-5p | [AEN](http://www.ncbi.nlm.nih.gov/entrez/query.fcgi?db=gene&cmd=Retrieve&dopt=full_report&list_uids=64782) | apoptosis enhancing nuclease |
| [Details](https://mirdb.org/cgi-bin/target_detail.cgi?targetID=2854194) | 1352 | 51 | hsa-miR-20a-5p | [NBL1](http://www.ncbi.nlm.nih.gov/entrez/query.fcgi?db=gene&cmd=Retrieve&dopt=full_report&list_uids=4681) | NBL1, DAN family BMP antagonist |
| [Details](https://mirdb.org/cgi-bin/target_detail.cgi?targetID=2854287) | 1353 | 51 | hsa-miR-20a-5p | [RNF145](http://www.ncbi.nlm.nih.gov/entrez/query.fcgi?db=gene&cmd=Retrieve&dopt=full_report&list_uids=153830) | ring finger protein 145 |
| [Details](https://mirdb.org/cgi-bin/target_detail.cgi?targetID=2854449) | 1354 | 51 | hsa-miR-20a-5p | [PDCD4](http://www.ncbi.nlm.nih.gov/entrez/query.fcgi?db=gene&cmd=Retrieve&dopt=full_report&list_uids=27250) | programmed cell death 4 |
| [Details](https://mirdb.org/cgi-bin/target_detail.cgi?targetID=2854557) | 1355 | 51 | hsa-miR-20a-5p | [COBL](http://www.ncbi.nlm.nih.gov/entrez/query.fcgi?db=gene&cmd=Retrieve&dopt=full_report&list_uids=23242) | cordon-bleu WH2 repeat protein |
| [Details](https://mirdb.org/cgi-bin/target_detail.cgi?targetID=2854568) | 1356 | 51 | hsa-miR-20a-5p | [GRTP1](http://www.ncbi.nlm.nih.gov/entrez/query.fcgi?db=gene&cmd=Retrieve&dopt=full_report&list_uids=79774) | growth hormone regulated TBC protein 1 |
| [Details](https://mirdb.org/cgi-bin/target_detail.cgi?targetID=2854594) | 1357 | 51 | hsa-miR-20a-5p | [POU2F1](http://www.ncbi.nlm.nih.gov/entrez/query.fcgi?db=gene&cmd=Retrieve&dopt=full_report&list_uids=5451) | POU class 2 homeobox 1 |
| [Details](https://mirdb.org/cgi-bin/target_detail.cgi?targetID=2854712) | 1358 | 51 | hsa-miR-20a-5p | [UBAP1](http://www.ncbi.nlm.nih.gov/entrez/query.fcgi?db=gene&cmd=Retrieve&dopt=full_report&list_uids=51271) | ubiquitin associated protein 1 |
| [Details](https://mirdb.org/cgi-bin/target_detail.cgi?targetID=2854744) | 1359 | 51 | hsa-miR-20a-5p | [NCEH1](http://www.ncbi.nlm.nih.gov/entrez/query.fcgi?db=gene&cmd=Retrieve&dopt=full_report&list_uids=57552) | neutral cholesterol ester hydrolase 1 |
| [Details](https://mirdb.org/cgi-bin/target_detail.cgi?targetID=2854862) | 1360 | 51 | hsa-miR-20a-5p | [PCDHA9](http://www.ncbi.nlm.nih.gov/entrez/query.fcgi?db=gene&cmd=Retrieve&dopt=full_report&list_uids=9752) | protocadherin alpha 9 |
| [Details](https://mirdb.org/cgi-bin/target_detail.cgi?targetID=2854876) | 1361 | 51 | hsa-miR-20a-5p | [NCOR1](http://www.ncbi.nlm.nih.gov/entrez/query.fcgi?db=gene&cmd=Retrieve&dopt=full_report&list_uids=9611) | nuclear receptor corepressor 1 |
| [Details](https://mirdb.org/cgi-bin/target_detail.cgi?targetID=2854880) | 1362 | 51 | hsa-miR-20a-5p | [CYP39A1](http://www.ncbi.nlm.nih.gov/entrez/query.fcgi?db=gene&cmd=Retrieve&dopt=full_report&list_uids=51302) | cytochrome P450 family 39 subfamily A member 1 |
| [Details](https://mirdb.org/cgi-bin/target_detail.cgi?targetID=2854914) | 1363 | 51 | hsa-miR-20a-5p | [MTDH](http://www.ncbi.nlm.nih.gov/entrez/query.fcgi?db=gene&cmd=Retrieve&dopt=full_report&list_uids=92140) | metadherin |
| [Details](https://mirdb.org/cgi-bin/target_detail.cgi?targetID=2855126) | 1364 | 51 | hsa-miR-20a-5p | [TPM3](http://www.ncbi.nlm.nih.gov/entrez/query.fcgi?db=gene&cmd=Retrieve&dopt=full_report&list_uids=7170) | tropomyosin 3 |
| [Details](https://mirdb.org/cgi-bin/target_detail.cgi?targetID=2855129) | 1365 | 51 | hsa-miR-20a-5p | [NXF1](http://www.ncbi.nlm.nih.gov/entrez/query.fcgi?db=gene&cmd=Retrieve&dopt=full_report&list_uids=10482) | nuclear RNA export factor 1 |
| [Details](https://mirdb.org/cgi-bin/target_detail.cgi?targetID=2855354) | 1366 | 51 | hsa-miR-20a-5p | [TSKU](http://www.ncbi.nlm.nih.gov/entrez/query.fcgi?db=gene&cmd=Retrieve&dopt=full_report&list_uids=25987) | tsukushi, small leucine rich proteoglycan |
| [Details](https://mirdb.org/cgi-bin/target_detail.cgi?targetID=2854083) | 1367 | 50 | hsa-miR-20a-5p | [MOG](http://www.ncbi.nlm.nih.gov/entrez/query.fcgi?db=gene&cmd=Retrieve&dopt=full_report&list_uids=4340) | myelin oligodendrocyte glycoprotein |
| [Details](https://mirdb.org/cgi-bin/target_detail.cgi?targetID=2854151) | 1368 | 50 | hsa-miR-20a-5p | [NTN1](http://www.ncbi.nlm.nih.gov/entrez/query.fcgi?db=gene&cmd=Retrieve&dopt=full_report&list_uids=9423) | netrin 1 |
| [Details](https://mirdb.org/cgi-bin/target_detail.cgi?targetID=2854175) | 1369 | 50 | hsa-miR-20a-5p | [BLOC1S6](http://www.ncbi.nlm.nih.gov/entrez/query.fcgi?db=gene&cmd=Retrieve&dopt=full_report&list_uids=26258) | biogenesis of lysosomal organelles complex 1 subunit 6 |
| [Details](https://mirdb.org/cgi-bin/target_detail.cgi?targetID=2854177) | 1370 | 50 | hsa-miR-20a-5p | [NOTCH2NLA](http://www.ncbi.nlm.nih.gov/entrez/query.fcgi?db=gene&cmd=Retrieve&dopt=full_report&list_uids=388677) | notch 2 N-terminal like A |
| [Details](https://mirdb.org/cgi-bin/target_detail.cgi?targetID=2854228) | 1371 | 50 | hsa-miR-20a-5p | [SCD](http://www.ncbi.nlm.nih.gov/entrez/query.fcgi?db=gene&cmd=Retrieve&dopt=full_report&list_uids=6319) | stearoyl-CoA desaturase |
| [Details](https://mirdb.org/cgi-bin/target_detail.cgi?targetID=2854231) | 1372 | 50 | hsa-miR-20a-5p | [MYO19](http://www.ncbi.nlm.nih.gov/entrez/query.fcgi?db=gene&cmd=Retrieve&dopt=full_report&list_uids=80179) | myosin XIX |
| [Details](https://mirdb.org/cgi-bin/target_detail.cgi?targetID=2854263) | 1373 | 50 | hsa-miR-20a-5p | [KLF3](http://www.ncbi.nlm.nih.gov/entrez/query.fcgi?db=gene&cmd=Retrieve&dopt=full_report&list_uids=51274) | Kruppel like factor 3 |
| [Details](https://mirdb.org/cgi-bin/target_detail.cgi?targetID=2854453) | 1374 | 50 | hsa-miR-20a-5p | [ZNF385A](http://www.ncbi.nlm.nih.gov/entrez/query.fcgi?db=gene&cmd=Retrieve&dopt=full_report&list_uids=25946) | zinc finger protein 385A |
| [Details](https://mirdb.org/cgi-bin/target_detail.cgi?targetID=2854589) | 1375 | 50 | hsa-miR-20a-5p | [CYSLTR2](http://www.ncbi.nlm.nih.gov/entrez/query.fcgi?db=gene&cmd=Retrieve&dopt=full_report&list_uids=57105) | cysteinyl leukotriene receptor 2 |
| [Details](https://mirdb.org/cgi-bin/target_detail.cgi?targetID=2854733) | 1376 | 50 | hsa-miR-20a-5p | [CAV1](http://www.ncbi.nlm.nih.gov/entrez/query.fcgi?db=gene&cmd=Retrieve&dopt=full_report&list_uids=857) | caveolin 1 |
| [Details](https://mirdb.org/cgi-bin/target_detail.cgi?targetID=2854797) | 1377 | 50 | hsa-miR-20a-5p | [CKAP2](http://www.ncbi.nlm.nih.gov/entrez/query.fcgi?db=gene&cmd=Retrieve&dopt=full_report&list_uids=26586) | cytoskeleton associated protein 2 |
| [Details](https://mirdb.org/cgi-bin/target_detail.cgi?targetID=2855018) | 1378 | 50 | hsa-miR-20a-5p | [NT5E](http://www.ncbi.nlm.nih.gov/entrez/query.fcgi?db=gene&cmd=Retrieve&dopt=full_report&list_uids=4907) | 5'-nucleotidase ecto |
| [Details](https://mirdb.org/cgi-bin/target_detail.cgi?targetID=2855085) | 1379 | 50 | hsa-miR-20a-5p | [TCTEX1D1](http://www.ncbi.nlm.nih.gov/entrez/query.fcgi?db=gene&cmd=Retrieve&dopt=full_report&list_uids=200132) | Tctex1 domain containing 1 |
| [Details](https://mirdb.org/cgi-bin/target_detail.cgi?targetID=2855205) | 1380 | 50 | hsa-miR-20a-5p | [TTR](http://www.ncbi.nlm.nih.gov/entrez/query.fcgi?db=gene&cmd=Retrieve&dopt=full_report&list_uids=7276) | transthyretin |
| [Details](https://mirdb.org/cgi-bin/target_detail.cgi?targetID=2855291) | 1381 | 50 | hsa-miR-20a-5p | [XIAP](http://www.ncbi.nlm.nih.gov/entrez/query.fcgi?db=gene&cmd=Retrieve&dopt=full_report&list_uids=331) | X-linked inhibitor of apoptosis |
