# Supplementary material for: Integrated analysis of miR-15a-5p, miR-20a-5p, and miR-33b-3p identifies EGR2-associated biomarkers in multiple myeloma
Source: BMC Cancer. 2026 Feb 12;26:298. doi: 10.1186/s12885-026-15610-5 (PMC12930744; doi:10.1186/s12885-026-15610-5)
Supplement: Supplementary file 3 — Supplementary Material 3. [file 12885_2026_15610_MOESM3_ESM.docx]

**Supplementary table (3):** Predicted targets for hsa-miR-33b-3p.

| **Target Detail** | **Target Rank** | **Target Score** | **miRNA Name** | **Gene Symbol** | **Gene Description** |
| --- | --- | --- | --- | --- | --- |
| [Details](https://mirdb.org/cgi-bin/target_detail.cgi?targetID=2492576) | 1 | 98 | hsa-miR-33b-3p | [PAFAH1B2](http://www.ncbi.nlm.nih.gov/entrez/query.fcgi?db=gene&cmd=Retrieve&dopt=full_report&list_uids=5049) | platelet activating factor acetylhydrolase 1b catalytic subunit 2 |
| [Details](https://mirdb.org/cgi-bin/target_detail.cgi?targetID=2492496) | 2 | 97 | hsa-miR-33b-3p | [CLOCK](http://www.ncbi.nlm.nih.gov/entrez/query.fcgi?db=gene&cmd=Retrieve&dopt=full_report&list_uids=9575) | clock circadian regulator |
| [Details](https://mirdb.org/cgi-bin/target_detail.cgi?targetID=2492517) | 3 | 97 | hsa-miR-33b-3p | [FAT2](http://www.ncbi.nlm.nih.gov/entrez/query.fcgi?db=gene&cmd=Retrieve&dopt=full_report&list_uids=2196) | FAT atypical cadherin 2 |
| [Details](https://mirdb.org/cgi-bin/target_detail.cgi?targetID=2492394) | 4 | 96 | hsa-miR-33b-3p | [FEZF1](http://www.ncbi.nlm.nih.gov/entrez/query.fcgi?db=gene&cmd=Retrieve&dopt=full_report&list_uids=389549) | FEZ family zinc finger 1 |
| [Details](https://mirdb.org/cgi-bin/target_detail.cgi?targetID=2492491) | 5 | 94 | hsa-miR-33b-3p | [IGSF10](http://www.ncbi.nlm.nih.gov/entrez/query.fcgi?db=gene&cmd=Retrieve&dopt=full_report&list_uids=285313) | immunoglobulin superfamily member 10 |
| [Details](https://mirdb.org/cgi-bin/target_detail.cgi?targetID=2492512) | 6 | 94 | hsa-miR-33b-3p | [NR4A2](http://www.ncbi.nlm.nih.gov/entrez/query.fcgi?db=gene&cmd=Retrieve&dopt=full_report&list_uids=4929) | nuclear receptor subfamily 4 group A member 2 |
| [Details](https://mirdb.org/cgi-bin/target_detail.cgi?targetID=2492397) | 7 | 93 | hsa-miR-33b-3p | [EPHA7](http://www.ncbi.nlm.nih.gov/entrez/query.fcgi?db=gene&cmd=Retrieve&dopt=full_report&list_uids=2045) | EPH receptor A7 |
| [Details](https://mirdb.org/cgi-bin/target_detail.cgi?targetID=2492432) | 8 | 93 | hsa-miR-33b-3p | [NPAS2](http://www.ncbi.nlm.nih.gov/entrez/query.fcgi?db=gene&cmd=Retrieve&dopt=full_report&list_uids=4862) | neuronal PAS domain protein 2 |
| [Details](https://mirdb.org/cgi-bin/target_detail.cgi?targetID=2492454) | 9 | 93 | hsa-miR-33b-3p | [AGO1](http://www.ncbi.nlm.nih.gov/entrez/query.fcgi?db=gene&cmd=Retrieve&dopt=full_report&list_uids=26523) | argonaute RISC catalytic component 1 |
| [Details](https://mirdb.org/cgi-bin/target_detail.cgi?targetID=2492543) | 10 | 92 | hsa-miR-33b-3p | [YPEL4](http://www.ncbi.nlm.nih.gov/entrez/query.fcgi?db=gene&cmd=Retrieve&dopt=full_report&list_uids=219539) | yippee like 4 |
| [Details](https://mirdb.org/cgi-bin/target_detail.cgi?targetID=2492549) | 11 | 92 | hsa-miR-33b-3p | [TOB1](http://www.ncbi.nlm.nih.gov/entrez/query.fcgi?db=gene&cmd=Retrieve&dopt=full_report&list_uids=10140) | transducer of ERBB2, 1 |
| [Details](https://mirdb.org/cgi-bin/target_detail.cgi?targetID=2492571) | 12 | 92 | hsa-miR-33b-3p | [MAPK6](http://www.ncbi.nlm.nih.gov/entrez/query.fcgi?db=gene&cmd=Retrieve&dopt=full_report&list_uids=5597) | mitogen-activated protein kinase 6 |
| [Details](https://mirdb.org/cgi-bin/target_detail.cgi?targetID=2492588) | 13 | 92 | hsa-miR-33b-3p | [BCL11B](http://www.ncbi.nlm.nih.gov/entrez/query.fcgi?db=gene&cmd=Retrieve&dopt=full_report&list_uids=64919) | BCL11B, BAF complex component |
| [Details](https://mirdb.org/cgi-bin/target_detail.cgi?targetID=2492640) | 14 | 92 | hsa-miR-33b-3p | [ZNF280B](http://www.ncbi.nlm.nih.gov/entrez/query.fcgi?db=gene&cmd=Retrieve&dopt=full_report&list_uids=140883) | zinc finger protein 280B |
| [Details](https://mirdb.org/cgi-bin/target_detail.cgi?targetID=2492650) | 15 | 92 | hsa-miR-33b-3p | [PRICKLE3](http://www.ncbi.nlm.nih.gov/entrez/query.fcgi?db=gene&cmd=Retrieve&dopt=full_report&list_uids=4007) | prickle planar cell polarity protein 3 |
| [Details](https://mirdb.org/cgi-bin/target_detail.cgi?targetID=2492522) | 16 | 91 | hsa-miR-33b-3p | [ZNF213](http://www.ncbi.nlm.nih.gov/entrez/query.fcgi?db=gene&cmd=Retrieve&dopt=full_report&list_uids=7760) | zinc finger protein 213 |
| [Details](https://mirdb.org/cgi-bin/target_detail.cgi?targetID=2492525) | 17 | 91 | hsa-miR-33b-3p | [PAFAH1B1](http://www.ncbi.nlm.nih.gov/entrez/query.fcgi?db=gene&cmd=Retrieve&dopt=full_report&list_uids=5048) | platelet activating factor acetylhydrolase 1b regulatory subunit 1 |
| [Details](https://mirdb.org/cgi-bin/target_detail.cgi?targetID=2492557) | 18 | 91 | hsa-miR-33b-3p | [RNF11](http://www.ncbi.nlm.nih.gov/entrez/query.fcgi?db=gene&cmd=Retrieve&dopt=full_report&list_uids=26994) | ring finger protein 11 |
| [Details](https://mirdb.org/cgi-bin/target_detail.cgi?targetID=2492569) | 19 | 91 | hsa-miR-33b-3p | [MCF2L](http://www.ncbi.nlm.nih.gov/entrez/query.fcgi?db=gene&cmd=Retrieve&dopt=full_report&list_uids=23263) | MCF.2 cell line derived transforming sequence like |
| [Details](https://mirdb.org/cgi-bin/target_detail.cgi?targetID=2492618) | 20 | 91 | hsa-miR-33b-3p | [GDAP1](http://www.ncbi.nlm.nih.gov/entrez/query.fcgi?db=gene&cmd=Retrieve&dopt=full_report&list_uids=54332) | ganglioside induced differentiation associated protein 1 |
| [Details](https://mirdb.org/cgi-bin/target_detail.cgi?targetID=2492480) | 21 | 90 | hsa-miR-33b-3p | [GABBR2](http://www.ncbi.nlm.nih.gov/entrez/query.fcgi?db=gene&cmd=Retrieve&dopt=full_report&list_uids=9568) | gamma-aminobutyric acid type B receptor subunit 2 |
| [Details](https://mirdb.org/cgi-bin/target_detail.cgi?targetID=2492439) | 22 | 89 | hsa-miR-33b-3p | [DLG2](http://www.ncbi.nlm.nih.gov/entrez/query.fcgi?db=gene&cmd=Retrieve&dopt=full_report&list_uids=1740) | discs large MAGUK scaffold protein 2 |
| [Details](https://mirdb.org/cgi-bin/target_detail.cgi?targetID=2492545) | 23 | 89 | hsa-miR-33b-3p | [CYB561D1](http://www.ncbi.nlm.nih.gov/entrez/query.fcgi?db=gene&cmd=Retrieve&dopt=full_report&list_uids=284613) | cytochrome b561 family member D1 |
| [Details](https://mirdb.org/cgi-bin/target_detail.cgi?targetID=2492621) | 24 | 89 | hsa-miR-33b-3p | [PTPRG](http://www.ncbi.nlm.nih.gov/entrez/query.fcgi?db=gene&cmd=Retrieve&dopt=full_report&list_uids=5793) | protein tyrosine phosphatase, receptor type G |
| [Details](https://mirdb.org/cgi-bin/target_detail.cgi?targetID=2492638) | 25 | 89 | hsa-miR-33b-3p | [LRP8](http://www.ncbi.nlm.nih.gov/entrez/query.fcgi?db=gene&cmd=Retrieve&dopt=full_report&list_uids=7804) | LDL receptor related protein 8 |
| [Details](https://mirdb.org/cgi-bin/target_detail.cgi?targetID=2492406) | 26 | 88 | hsa-miR-33b-3p | [ITPRID2](http://www.ncbi.nlm.nih.gov/entrez/query.fcgi?db=gene&cmd=Retrieve&dopt=full_report&list_uids=6744) | ITPR interacting domain containing 2 |
| [Details](https://mirdb.org/cgi-bin/target_detail.cgi?targetID=2492486) | 27 | 88 | hsa-miR-33b-3p | [SYT1](http://www.ncbi.nlm.nih.gov/entrez/query.fcgi?db=gene&cmd=Retrieve&dopt=full_report&list_uids=6857) | synaptotagmin 1 |
| [Details](https://mirdb.org/cgi-bin/target_detail.cgi?targetID=2492505) | 28 | 88 | hsa-miR-33b-3p | [URI1](http://www.ncbi.nlm.nih.gov/entrez/query.fcgi?db=gene&cmd=Retrieve&dopt=full_report&list_uids=8725) | URI1, prefoldin like chaperone |
| [Details](https://mirdb.org/cgi-bin/target_detail.cgi?targetID=2492554) | 29 | 88 | hsa-miR-33b-3p | [TBC1D3H](http://www.ncbi.nlm.nih.gov/entrez/query.fcgi?db=gene&cmd=Retrieve&dopt=full_report&list_uids=729877) | TBC1 domain family member 3H |
| [Details](https://mirdb.org/cgi-bin/target_detail.cgi?targetID=2492641) | 30 | 88 | hsa-miR-33b-3p | [PTPN4](http://www.ncbi.nlm.nih.gov/entrez/query.fcgi?db=gene&cmd=Retrieve&dopt=full_report&list_uids=5775) | protein tyrosine phosphatase, non-receptor type 4 |
| [Details](https://mirdb.org/cgi-bin/target_detail.cgi?targetID=2492497) | 31 | 87 | hsa-miR-33b-3p | [TRAK1](http://www.ncbi.nlm.nih.gov/entrez/query.fcgi?db=gene&cmd=Retrieve&dopt=full_report&list_uids=22906) | trafficking kinesin protein 1 |
| [Details](https://mirdb.org/cgi-bin/target_detail.cgi?targetID=2492542) | 32 | 87 | hsa-miR-33b-3p | [DOCK4](http://www.ncbi.nlm.nih.gov/entrez/query.fcgi?db=gene&cmd=Retrieve&dopt=full_report&list_uids=9732) | dedicator of cytokinesis 4 |
| [Details](https://mirdb.org/cgi-bin/target_detail.cgi?targetID=2492612) | 33 | 87 | hsa-miR-33b-3p | [DLL1](http://www.ncbi.nlm.nih.gov/entrez/query.fcgi?db=gene&cmd=Retrieve&dopt=full_report&list_uids=28514) | delta like canonical Notch ligand 1 |
| [Details](https://mirdb.org/cgi-bin/target_detail.cgi?targetID=2492617) | 34 | 87 | hsa-miR-33b-3p | [KCNJ13](http://www.ncbi.nlm.nih.gov/entrez/query.fcgi?db=gene&cmd=Retrieve&dopt=full_report&list_uids=3769) | potassium voltage-gated channel subfamily J member 13 |
| [Details](https://mirdb.org/cgi-bin/target_detail.cgi?targetID=2492415) | 35 | 86 | hsa-miR-33b-3p | [PLBD1](http://www.ncbi.nlm.nih.gov/entrez/query.fcgi?db=gene&cmd=Retrieve&dopt=full_report&list_uids=79887) | phospholipase B domain containing 1 |
| [Details](https://mirdb.org/cgi-bin/target_detail.cgi?targetID=2492459) | 36 | 86 | hsa-miR-33b-3p | [ATG14](http://www.ncbi.nlm.nih.gov/entrez/query.fcgi?db=gene&cmd=Retrieve&dopt=full_report&list_uids=22863) | autophagy related 14 |
| [Details](https://mirdb.org/cgi-bin/target_detail.cgi?targetID=2492492) | 37 | 86 | hsa-miR-33b-3p | [ZNRF3](http://www.ncbi.nlm.nih.gov/entrez/query.fcgi?db=gene&cmd=Retrieve&dopt=full_report&list_uids=84133) | zinc and ring finger 3 |
| [Details](https://mirdb.org/cgi-bin/target_detail.cgi?targetID=2492556) | 38 | 86 | hsa-miR-33b-3p | [AADAC](http://www.ncbi.nlm.nih.gov/entrez/query.fcgi?db=gene&cmd=Retrieve&dopt=full_report&list_uids=13) | arylacetamide deacetylase |
| [Details](https://mirdb.org/cgi-bin/target_detail.cgi?targetID=2492441) | 39 | 85 | hsa-miR-33b-3p | [TBC1D3](http://www.ncbi.nlm.nih.gov/entrez/query.fcgi?db=gene&cmd=Retrieve&dopt=full_report&list_uids=729873) | TBC1 domain family member 3 |
| [Details](https://mirdb.org/cgi-bin/target_detail.cgi?targetID=2492503) | 40 | 85 | hsa-miR-33b-3p | [TLN2](http://www.ncbi.nlm.nih.gov/entrez/query.fcgi?db=gene&cmd=Retrieve&dopt=full_report&list_uids=83660) | talin 2 |
| [Details](https://mirdb.org/cgi-bin/target_detail.cgi?targetID=2492531) | 41 | 85 | hsa-miR-33b-3p | [DPYSL5](http://www.ncbi.nlm.nih.gov/entrez/query.fcgi?db=gene&cmd=Retrieve&dopt=full_report&list_uids=56896) | dihydropyrimidinase like 5 |
| [Details](https://mirdb.org/cgi-bin/target_detail.cgi?targetID=2492548) | 42 | 85 | hsa-miR-33b-3p | [C5AR2](http://www.ncbi.nlm.nih.gov/entrez/query.fcgi?db=gene&cmd=Retrieve&dopt=full_report&list_uids=27202) | complement component 5a receptor 2 |
| [Details](https://mirdb.org/cgi-bin/target_detail.cgi?targetID=2492552) | 43 | 85 | hsa-miR-33b-3p | [BICD2](http://www.ncbi.nlm.nih.gov/entrez/query.fcgi?db=gene&cmd=Retrieve&dopt=full_report&list_uids=23299) | BICD cargo adaptor 2 |
| [Details](https://mirdb.org/cgi-bin/target_detail.cgi?targetID=2492570) | 44 | 85 | hsa-miR-33b-3p | [NR1D2](http://www.ncbi.nlm.nih.gov/entrez/query.fcgi?db=gene&cmd=Retrieve&dopt=full_report&list_uids=9975) | nuclear receptor subfamily 1 group D member 2 |
| [Details](https://mirdb.org/cgi-bin/target_detail.cgi?targetID=2492658) | 45 | 85 | hsa-miR-33b-3p | [PRAMEF17](http://www.ncbi.nlm.nih.gov/entrez/query.fcgi?db=gene&cmd=Retrieve&dopt=full_report&list_uids=391004) | PRAME family member 17 |
| [Details](https://mirdb.org/cgi-bin/target_detail.cgi?targetID=2492395) | 46 | 84 | hsa-miR-33b-3p | [PAPOLB](http://www.ncbi.nlm.nih.gov/entrez/query.fcgi?db=gene&cmd=Retrieve&dopt=full_report&list_uids=56903) | poly(A) polymerase beta |
| [Details](https://mirdb.org/cgi-bin/target_detail.cgi?targetID=2492410) | 47 | 84 | hsa-miR-33b-3p | [CACNA1D](http://www.ncbi.nlm.nih.gov/entrez/query.fcgi?db=gene&cmd=Retrieve&dopt=full_report&list_uids=776) | calcium voltage-gated channel subunit alpha1 D |
| [Details](https://mirdb.org/cgi-bin/target_detail.cgi?targetID=2492472) | 48 | 84 | hsa-miR-33b-3p | [ZNF513](http://www.ncbi.nlm.nih.gov/entrez/query.fcgi?db=gene&cmd=Retrieve&dopt=full_report&list_uids=130557) | zinc finger protein 513 |
| [Details](https://mirdb.org/cgi-bin/target_detail.cgi?targetID=2492562) | 49 | 84 | hsa-miR-33b-3p | [GGCX](http://www.ncbi.nlm.nih.gov/entrez/query.fcgi?db=gene&cmd=Retrieve&dopt=full_report&list_uids=2677) | gamma-glutamyl carboxylase |
| [Details](https://mirdb.org/cgi-bin/target_detail.cgi?targetID=2492563) | 50 | 84 | hsa-miR-33b-3p | [SETD3](http://www.ncbi.nlm.nih.gov/entrez/query.fcgi?db=gene&cmd=Retrieve&dopt=full_report&list_uids=84193) | SET domain containing 3, actin histidine methyltransferase |
| [Details](https://mirdb.org/cgi-bin/target_detail.cgi?targetID=2492602) | 51 | 84 | hsa-miR-33b-3p | [KIAA1549L](http://www.ncbi.nlm.nih.gov/entrez/query.fcgi?db=gene&cmd=Retrieve&dopt=full_report&list_uids=25758) | KIAA1549 like |
| [Details](https://mirdb.org/cgi-bin/target_detail.cgi?targetID=2492629) | 52 | 84 | hsa-miR-33b-3p | [BAZ1B](http://www.ncbi.nlm.nih.gov/entrez/query.fcgi?db=gene&cmd=Retrieve&dopt=full_report&list_uids=9031) | bromodomain adjacent to zinc finger domain 1B |
| [Details](https://mirdb.org/cgi-bin/target_detail.cgi?targetID=2492592) | 53 | 83 | hsa-miR-33b-3p | [ST6GAL2](http://www.ncbi.nlm.nih.gov/entrez/query.fcgi?db=gene&cmd=Retrieve&dopt=full_report&list_uids=84620) | ST6 beta-galactoside alpha-2,6-sialyltransferase 2 |
| [Details](https://mirdb.org/cgi-bin/target_detail.cgi?targetID=2492603) | 54 | 83 | hsa-miR-33b-3p | [PPP1R7](http://www.ncbi.nlm.nih.gov/entrez/query.fcgi?db=gene&cmd=Retrieve&dopt=full_report&list_uids=5510) | protein phosphatase 1 regulatory subunit 7 |
| [Details](https://mirdb.org/cgi-bin/target_detail.cgi?targetID=2492633) | 55 | 83 | hsa-miR-33b-3p | [ZSWIM6](http://www.ncbi.nlm.nih.gov/entrez/query.fcgi?db=gene&cmd=Retrieve&dopt=full_report&list_uids=57688) | zinc finger SWIM-type containing 6 |
| [Details](https://mirdb.org/cgi-bin/target_detail.cgi?targetID=2492462) | 56 | 82 | hsa-miR-33b-3p | [DAZAP2](http://www.ncbi.nlm.nih.gov/entrez/query.fcgi?db=gene&cmd=Retrieve&dopt=full_report&list_uids=9802) | DAZ associated protein 2 |
| [Details](https://mirdb.org/cgi-bin/target_detail.cgi?targetID=2492479) | 57 | 82 | hsa-miR-33b-3p | [CBLL1](http://www.ncbi.nlm.nih.gov/entrez/query.fcgi?db=gene&cmd=Retrieve&dopt=full_report&list_uids=79872) | Cbl proto-oncogene like 1 |
| [Details](https://mirdb.org/cgi-bin/target_detail.cgi?targetID=2492627) | 58 | 82 | hsa-miR-33b-3p | [C6orf222](http://www.ncbi.nlm.nih.gov/entrez/query.fcgi?db=gene&cmd=Retrieve&dopt=full_report&list_uids=389384) | chromosome 6 open reading frame 222 |
| [Details](https://mirdb.org/cgi-bin/target_detail.cgi?targetID=2492646) | 59 | 82 | hsa-miR-33b-3p | [TIGD2](http://www.ncbi.nlm.nih.gov/entrez/query.fcgi?db=gene&cmd=Retrieve&dopt=full_report&list_uids=166815) | tigger transposable element derived 2 |
| [Details](https://mirdb.org/cgi-bin/target_detail.cgi?targetID=2492402) | 60 | 81 | hsa-miR-33b-3p | [PFN2](http://www.ncbi.nlm.nih.gov/entrez/query.fcgi?db=gene&cmd=Retrieve&dopt=full_report&list_uids=5217) | profilin 2 |
| [Details](https://mirdb.org/cgi-bin/target_detail.cgi?targetID=2492526) | 61 | 81 | hsa-miR-33b-3p | [DCUN1D3](http://www.ncbi.nlm.nih.gov/entrez/query.fcgi?db=gene&cmd=Retrieve&dopt=full_report&list_uids=123879) | defective in cullin neddylation 1 domain containing 3 |
| [Details](https://mirdb.org/cgi-bin/target_detail.cgi?targetID=2492630) | 62 | 81 | hsa-miR-33b-3p | [TENM3](http://www.ncbi.nlm.nih.gov/entrez/query.fcgi?db=gene&cmd=Retrieve&dopt=full_report&list_uids=55714) | teneurin transmembrane protein 3 |
| [Details](https://mirdb.org/cgi-bin/target_detail.cgi?targetID=2492423) | 63 | 80 | hsa-miR-33b-3p | [KIF14](http://www.ncbi.nlm.nih.gov/entrez/query.fcgi?db=gene&cmd=Retrieve&dopt=full_report&list_uids=9928) | kinesin family member 14 |
| [Details](https://mirdb.org/cgi-bin/target_detail.cgi?targetID=2492449) | 64 | 80 | hsa-miR-33b-3p | [CSRNP3](http://www.ncbi.nlm.nih.gov/entrez/query.fcgi?db=gene&cmd=Retrieve&dopt=full_report&list_uids=80034) | cysteine and serine rich nuclear protein 3 |
| [Details](https://mirdb.org/cgi-bin/target_detail.cgi?targetID=2492470) | 65 | 80 | hsa-miR-33b-3p | [TBC1D2](http://www.ncbi.nlm.nih.gov/entrez/query.fcgi?db=gene&cmd=Retrieve&dopt=full_report&list_uids=55357) | TBC1 domain family member 2 |
| [Details](https://mirdb.org/cgi-bin/target_detail.cgi?targetID=2492544) | 66 | 80 | hsa-miR-33b-3p | [SFR1](http://www.ncbi.nlm.nih.gov/entrez/query.fcgi?db=gene&cmd=Retrieve&dopt=full_report&list_uids=119392) | SWI5 dependent homologous recombination repair protein 1 |
| [Details](https://mirdb.org/cgi-bin/target_detail.cgi?targetID=2492558) | 67 | 80 | hsa-miR-33b-3p | [MTRF1L](http://www.ncbi.nlm.nih.gov/entrez/query.fcgi?db=gene&cmd=Retrieve&dopt=full_report&list_uids=54516) | mitochondrial translational release factor 1 like |
| [Details](https://mirdb.org/cgi-bin/target_detail.cgi?targetID=2492575) | 68 | 80 | hsa-miR-33b-3p | [TBC1D17](http://www.ncbi.nlm.nih.gov/entrez/query.fcgi?db=gene&cmd=Retrieve&dopt=full_report&list_uids=79735) | TBC1 domain family member 17 |
| [Details](https://mirdb.org/cgi-bin/target_detail.cgi?targetID=2492535) | 69 | 79 | hsa-miR-33b-3p | [THBS1](http://www.ncbi.nlm.nih.gov/entrez/query.fcgi?db=gene&cmd=Retrieve&dopt=full_report&list_uids=7057) | thrombospondin 1 |
| [Details](https://mirdb.org/cgi-bin/target_detail.cgi?targetID=2492538) | 70 | 79 | hsa-miR-33b-3p | [PKD1L1](http://www.ncbi.nlm.nih.gov/entrez/query.fcgi?db=gene&cmd=Retrieve&dopt=full_report&list_uids=168507) | polycystin 1 like 1, transient receptor potential channel interacting |
| [Details](https://mirdb.org/cgi-bin/target_detail.cgi?targetID=2492555) | 71 | 79 | hsa-miR-33b-3p | [MYO5A](http://www.ncbi.nlm.nih.gov/entrez/query.fcgi?db=gene&cmd=Retrieve&dopt=full_report&list_uids=4644) | myosin VA |
| [Details](https://mirdb.org/cgi-bin/target_detail.cgi?targetID=2492594) | 72 | 79 | hsa-miR-33b-3p | [AKAP6](http://www.ncbi.nlm.nih.gov/entrez/query.fcgi?db=gene&cmd=Retrieve&dopt=full_report&list_uids=9472) | A-kinase anchoring protein 6 |
| [Details](https://mirdb.org/cgi-bin/target_detail.cgi?targetID=2492655) | 73 | 79 | hsa-miR-33b-3p | [ZFP3](http://www.ncbi.nlm.nih.gov/entrez/query.fcgi?db=gene&cmd=Retrieve&dopt=full_report&list_uids=124961) | ZFP3 zinc finger protein |
| [Details](https://mirdb.org/cgi-bin/target_detail.cgi?targetID=2492442) | 74 | 78 | hsa-miR-33b-3p | [NEDD4L](http://www.ncbi.nlm.nih.gov/entrez/query.fcgi?db=gene&cmd=Retrieve&dopt=full_report&list_uids=23327) | neural precursor cell expressed, developmentally down-regulated 4-like, E3 ubiquitin protein ligase |
| [Details](https://mirdb.org/cgi-bin/target_detail.cgi?targetID=2492469) | 75 | 78 | hsa-miR-33b-3p | [LMO2](http://www.ncbi.nlm.nih.gov/entrez/query.fcgi?db=gene&cmd=Retrieve&dopt=full_report&list_uids=4005) | LIM domain only 2 |
| [Details](https://mirdb.org/cgi-bin/target_detail.cgi?targetID=2492513) | 76 | 78 | hsa-miR-33b-3p | [CD274](http://www.ncbi.nlm.nih.gov/entrez/query.fcgi?db=gene&cmd=Retrieve&dopt=full_report&list_uids=29126) | CD274 molecule |
| [Details](https://mirdb.org/cgi-bin/target_detail.cgi?targetID=2492637) | 77 | 78 | hsa-miR-33b-3p | [FGFR1](http://www.ncbi.nlm.nih.gov/entrez/query.fcgi?db=gene&cmd=Retrieve&dopt=full_report&list_uids=2260) | fibroblast growth factor receptor 1 |
| [Details](https://mirdb.org/cgi-bin/target_detail.cgi?targetID=2492398) | 78 | 77 | hsa-miR-33b-3p | [WAC](http://www.ncbi.nlm.nih.gov/entrez/query.fcgi?db=gene&cmd=Retrieve&dopt=full_report&list_uids=51322) | WW domain containing adaptor with coiled-coil |
| [Details](https://mirdb.org/cgi-bin/target_detail.cgi?targetID=2492453) | 79 | 77 | hsa-miR-33b-3p | [USH2A](http://www.ncbi.nlm.nih.gov/entrez/query.fcgi?db=gene&cmd=Retrieve&dopt=full_report&list_uids=7399) | usherin |
| [Details](https://mirdb.org/cgi-bin/target_detail.cgi?targetID=2492482) | 80 | 77 | hsa-miR-33b-3p | [HLX](http://www.ncbi.nlm.nih.gov/entrez/query.fcgi?db=gene&cmd=Retrieve&dopt=full_report&list_uids=3142) | H2.0 like homeobox |
| [Details](https://mirdb.org/cgi-bin/target_detail.cgi?targetID=2492498) | 81 | 77 | hsa-miR-33b-3p | [MYO6](http://www.ncbi.nlm.nih.gov/entrez/query.fcgi?db=gene&cmd=Retrieve&dopt=full_report&list_uids=4646) | myosin VI |
| [Details](https://mirdb.org/cgi-bin/target_detail.cgi?targetID=2492506) | 82 | 77 | hsa-miR-33b-3p | [HSP90AA1](http://www.ncbi.nlm.nih.gov/entrez/query.fcgi?db=gene&cmd=Retrieve&dopt=full_report&list_uids=3320) | heat shock protein 90 alpha family class A member 1 |
| [Details](https://mirdb.org/cgi-bin/target_detail.cgi?targetID=2492606) | 83 | 77 | hsa-miR-33b-3p | [GAPT](http://www.ncbi.nlm.nih.gov/entrez/query.fcgi?db=gene&cmd=Retrieve&dopt=full_report&list_uids=202309) | GRB2 binding adaptor protein, transmembrane |
| [Details](https://mirdb.org/cgi-bin/target_detail.cgi?targetID=2492475) | 84 | 76 | hsa-miR-33b-3p | [BCLAF3](http://www.ncbi.nlm.nih.gov/entrez/query.fcgi?db=gene&cmd=Retrieve&dopt=full_report&list_uids=256643) | BCLAF1 and THRAP3 family member 3 |
| [Details](https://mirdb.org/cgi-bin/target_detail.cgi?targetID=2492495) | 85 | 76 | hsa-miR-33b-3p | [ZKSCAN1](http://www.ncbi.nlm.nih.gov/entrez/query.fcgi?db=gene&cmd=Retrieve&dopt=full_report&list_uids=7586) | zinc finger with KRAB and SCAN domains 1 |
| [Details](https://mirdb.org/cgi-bin/target_detail.cgi?targetID=2492654) | 86 | 76 | hsa-miR-33b-3p | [FRMD4B](http://www.ncbi.nlm.nih.gov/entrez/query.fcgi?db=gene&cmd=Retrieve&dopt=full_report&list_uids=23150) | FERM domain containing 4B |
| [Details](https://mirdb.org/cgi-bin/target_detail.cgi?targetID=2492412) | 87 | 75 | hsa-miR-33b-3p | [MSR1](http://www.ncbi.nlm.nih.gov/entrez/query.fcgi?db=gene&cmd=Retrieve&dopt=full_report&list_uids=4481) | macrophage scavenger receptor 1 |
| [Details](https://mirdb.org/cgi-bin/target_detail.cgi?targetID=2492436) | 88 | 75 | hsa-miR-33b-3p | [CMTR2](http://www.ncbi.nlm.nih.gov/entrez/query.fcgi?db=gene&cmd=Retrieve&dopt=full_report&list_uids=55783) | cap methyltransferase 2 |
| [Details](https://mirdb.org/cgi-bin/target_detail.cgi?targetID=2492572) | 89 | 75 | hsa-miR-33b-3p | [APOL6](http://www.ncbi.nlm.nih.gov/entrez/query.fcgi?db=gene&cmd=Retrieve&dopt=full_report&list_uids=80830) | apolipoprotein L6 |
| [Details](https://mirdb.org/cgi-bin/target_detail.cgi?targetID=2492573) | 90 | 75 | hsa-miR-33b-3p | [CHRM2](http://www.ncbi.nlm.nih.gov/entrez/query.fcgi?db=gene&cmd=Retrieve&dopt=full_report&list_uids=1129) | cholinergic receptor muscarinic 2 |
| [Details](https://mirdb.org/cgi-bin/target_detail.cgi?targetID=2492580) | 91 | 75 | hsa-miR-33b-3p | [PI4KB](http://www.ncbi.nlm.nih.gov/entrez/query.fcgi?db=gene&cmd=Retrieve&dopt=full_report&list_uids=5298) | phosphatidylinositol 4-kinase beta |
| [Details](https://mirdb.org/cgi-bin/target_detail.cgi?targetID=2492494) | 92 | 74 | hsa-miR-33b-3p | [FBN2](http://www.ncbi.nlm.nih.gov/entrez/query.fcgi?db=gene&cmd=Retrieve&dopt=full_report&list_uids=2201) | fibrillin 2 |
| [Details](https://mirdb.org/cgi-bin/target_detail.cgi?targetID=2492499) | 93 | 74 | hsa-miR-33b-3p | [STMN4](http://www.ncbi.nlm.nih.gov/entrez/query.fcgi?db=gene&cmd=Retrieve&dopt=full_report&list_uids=81551) | stathmin 4 |
| [Details](https://mirdb.org/cgi-bin/target_detail.cgi?targetID=2492518) | 94 | 74 | hsa-miR-33b-3p | [ZFYVE26](http://www.ncbi.nlm.nih.gov/entrez/query.fcgi?db=gene&cmd=Retrieve&dopt=full_report&list_uids=23503) | zinc finger FYVE-type containing 26 |
| [Details](https://mirdb.org/cgi-bin/target_detail.cgi?targetID=2492583) | 95 | 74 | hsa-miR-33b-3p | [MKRN1](http://www.ncbi.nlm.nih.gov/entrez/query.fcgi?db=gene&cmd=Retrieve&dopt=full_report&list_uids=23608) | makorin ring finger protein 1 |
| [Details](https://mirdb.org/cgi-bin/target_detail.cgi?targetID=2492591) | 96 | 74 | hsa-miR-33b-3p | [SSTR1](http://www.ncbi.nlm.nih.gov/entrez/query.fcgi?db=gene&cmd=Retrieve&dopt=full_report&list_uids=6751) | somatostatin receptor 1 |
| [Details](https://mirdb.org/cgi-bin/target_detail.cgi?targetID=2492668) | 97 | 74 | hsa-miR-33b-3p | [UBE3C](http://www.ncbi.nlm.nih.gov/entrez/query.fcgi?db=gene&cmd=Retrieve&dopt=full_report&list_uids=9690) | ubiquitin protein ligase E3C |
| [Details](https://mirdb.org/cgi-bin/target_detail.cgi?targetID=2492418) | 98 | 73 | hsa-miR-33b-3p | [BTBD3](http://www.ncbi.nlm.nih.gov/entrez/query.fcgi?db=gene&cmd=Retrieve&dopt=full_report&list_uids=22903) | BTB domain containing 3 |
| [Details](https://mirdb.org/cgi-bin/target_detail.cgi?targetID=2492446) | 99 | 73 | hsa-miR-33b-3p | [RCBTB2](http://www.ncbi.nlm.nih.gov/entrez/query.fcgi?db=gene&cmd=Retrieve&dopt=full_report&list_uids=1102) | RCC1 and BTB domain containing protein 2 |
| [Details](https://mirdb.org/cgi-bin/target_detail.cgi?targetID=2492448) | 100 | 72 | hsa-miR-33b-3p | [MRTFB](http://www.ncbi.nlm.nih.gov/entrez/query.fcgi?db=gene&cmd=Retrieve&dopt=full_report&list_uids=57496) | myocardin related transcription factor B |
| [Details](https://mirdb.org/cgi-bin/target_detail.cgi?targetID=2492607) | 101 | 72 | hsa-miR-33b-3p | [C8orf59](http://www.ncbi.nlm.nih.gov/entrez/query.fcgi?db=gene&cmd=Retrieve&dopt=full_report&list_uids=401466) | chromosome 8 open reading frame 59 |
| [Details](https://mirdb.org/cgi-bin/target_detail.cgi?targetID=2492424) | 102 | 71 | hsa-miR-33b-3p | [TLK2](http://www.ncbi.nlm.nih.gov/entrez/query.fcgi?db=gene&cmd=Retrieve&dopt=full_report&list_uids=11011) | tousled like kinase 2 |
| [Details](https://mirdb.org/cgi-bin/target_detail.cgi?targetID=2492455) | 103 | 71 | hsa-miR-33b-3p | [DNALI1](http://www.ncbi.nlm.nih.gov/entrez/query.fcgi?db=gene&cmd=Retrieve&dopt=full_report&list_uids=7802) | dynein axonemal light intermediate chain 1 |
| [Details](https://mirdb.org/cgi-bin/target_detail.cgi?targetID=2492536) | 104 | 71 | hsa-miR-33b-3p | [MAST4](http://www.ncbi.nlm.nih.gov/entrez/query.fcgi?db=gene&cmd=Retrieve&dopt=full_report&list_uids=375449) | microtubule associated serine/threonine kinase family member 4 |
| [Details](https://mirdb.org/cgi-bin/target_detail.cgi?targetID=2492567) | 105 | 71 | hsa-miR-33b-3p | [TTC28](http://www.ncbi.nlm.nih.gov/entrez/query.fcgi?db=gene&cmd=Retrieve&dopt=full_report&list_uids=23331) | tetratricopeptide repeat domain 28 |
| [Details](https://mirdb.org/cgi-bin/target_detail.cgi?targetID=2492651) | 106 | 71 | hsa-miR-33b-3p | [NDUFA10](http://www.ncbi.nlm.nih.gov/entrez/query.fcgi?db=gene&cmd=Retrieve&dopt=full_report&list_uids=4705) | NADH:ubiquinone oxidoreductase subunit A10 |
| [Details](https://mirdb.org/cgi-bin/target_detail.cgi?targetID=2492666) | 107 | 71 | hsa-miR-33b-3p | [RNF165](http://www.ncbi.nlm.nih.gov/entrez/query.fcgi?db=gene&cmd=Retrieve&dopt=full_report&list_uids=494470) | ring finger protein 165 |
| [Details](https://mirdb.org/cgi-bin/target_detail.cgi?targetID=2492667) | 108 | 71 | hsa-miR-33b-3p | [MEF2A](http://www.ncbi.nlm.nih.gov/entrez/query.fcgi?db=gene&cmd=Retrieve&dopt=full_report&list_uids=4205) | myocyte enhancer factor 2A |
| [Details](https://mirdb.org/cgi-bin/target_detail.cgi?targetID=2492417) | 109 | 70 | hsa-miR-33b-3p | [CDKL5](http://www.ncbi.nlm.nih.gov/entrez/query.fcgi?db=gene&cmd=Retrieve&dopt=full_report&list_uids=6792) | cyclin dependent kinase like 5 |
| [Details](https://mirdb.org/cgi-bin/target_detail.cgi?targetID=2492426) | 110 | 70 | hsa-miR-33b-3p | [BCL11A](http://www.ncbi.nlm.nih.gov/entrez/query.fcgi?db=gene&cmd=Retrieve&dopt=full_report&list_uids=53335) | BCL11A, BAF complex component |
| [Details](https://mirdb.org/cgi-bin/target_detail.cgi?targetID=2492474) | 111 | 70 | hsa-miR-33b-3p | [PIK3CA](http://www.ncbi.nlm.nih.gov/entrez/query.fcgi?db=gene&cmd=Retrieve&dopt=full_report&list_uids=5290) | phosphatidylinositol-4,5-bisphosphate 3-kinase catalytic subunit alpha |
| [Details](https://mirdb.org/cgi-bin/target_detail.cgi?targetID=2492564) | 112 | 70 | hsa-miR-33b-3p | [ATP2A2](http://www.ncbi.nlm.nih.gov/entrez/query.fcgi?db=gene&cmd=Retrieve&dopt=full_report&list_uids=488) | ATPase sarcoplasmic/endoplasmic reticulum Ca2+ transporting 2 |
| [Details](https://mirdb.org/cgi-bin/target_detail.cgi?targetID=2492596) | 113 | 70 | hsa-miR-33b-3p | [NAA30](http://www.ncbi.nlm.nih.gov/entrez/query.fcgi?db=gene&cmd=Retrieve&dopt=full_report&list_uids=122830) | N(alpha)-acetyltransferase 30, NatC catalytic subunit |
| [Details](https://mirdb.org/cgi-bin/target_detail.cgi?targetID=2492620) | 114 | 70 | hsa-miR-33b-3p | [CRYZL1](http://www.ncbi.nlm.nih.gov/entrez/query.fcgi?db=gene&cmd=Retrieve&dopt=full_report&list_uids=9946) | crystallin zeta like 1 |
| [Details](https://mirdb.org/cgi-bin/target_detail.cgi?targetID=2492642) | 115 | 70 | hsa-miR-33b-3p | [NUFIP2](http://www.ncbi.nlm.nih.gov/entrez/query.fcgi?db=gene&cmd=Retrieve&dopt=full_report&list_uids=57532) | nuclear FMR1 interacting protein 2 |
| [Details](https://mirdb.org/cgi-bin/target_detail.cgi?targetID=2492433) | 116 | 69 | hsa-miR-33b-3p | [PRKD3](http://www.ncbi.nlm.nih.gov/entrez/query.fcgi?db=gene&cmd=Retrieve&dopt=full_report&list_uids=23683) | protein kinase D3 |
| [Details](https://mirdb.org/cgi-bin/target_detail.cgi?targetID=2492457) | 117 | 69 | hsa-miR-33b-3p | [RUNX1](http://www.ncbi.nlm.nih.gov/entrez/query.fcgi?db=gene&cmd=Retrieve&dopt=full_report&list_uids=861) | runt related transcription factor 1 |
| [Details](https://mirdb.org/cgi-bin/target_detail.cgi?targetID=2492551) | 118 | 69 | hsa-miR-33b-3p | [ZNF787](http://www.ncbi.nlm.nih.gov/entrez/query.fcgi?db=gene&cmd=Retrieve&dopt=full_report&list_uids=126208) | zinc finger protein 787 |
| [Details](https://mirdb.org/cgi-bin/target_detail.cgi?targetID=2492577) | 119 | 69 | hsa-miR-33b-3p | [CHST9](http://www.ncbi.nlm.nih.gov/entrez/query.fcgi?db=gene&cmd=Retrieve&dopt=full_report&list_uids=83539) | carbohydrate sulfotransferase 9 |
| [Details](https://mirdb.org/cgi-bin/target_detail.cgi?targetID=2492586) | 120 | 69 | hsa-miR-33b-3p | [BCOR](http://www.ncbi.nlm.nih.gov/entrez/query.fcgi?db=gene&cmd=Retrieve&dopt=full_report&list_uids=54880) | BCL6 corepressor |
| [Details](https://mirdb.org/cgi-bin/target_detail.cgi?targetID=2492601) | 121 | 69 | hsa-miR-33b-3p | [TRAF3IP1](http://www.ncbi.nlm.nih.gov/entrez/query.fcgi?db=gene&cmd=Retrieve&dopt=full_report&list_uids=26146) | TRAF3 interacting protein 1 |
| [Details](https://mirdb.org/cgi-bin/target_detail.cgi?targetID=2492659) | 122 | 69 | hsa-miR-33b-3p | [COX11](http://www.ncbi.nlm.nih.gov/entrez/query.fcgi?db=gene&cmd=Retrieve&dopt=full_report&list_uids=1353) | cytochrome c oxidase copper chaperone COX11 |
| [Details](https://mirdb.org/cgi-bin/target_detail.cgi?targetID=2492665) | 123 | 69 | hsa-miR-33b-3p | [ADGRF1](http://www.ncbi.nlm.nih.gov/entrez/query.fcgi?db=gene&cmd=Retrieve&dopt=full_report&list_uids=266977) | adhesion G protein-coupled receptor F1 |
| [Details](https://mirdb.org/cgi-bin/target_detail.cgi?targetID=2492407) | 124 | 68 | hsa-miR-33b-3p | [TMX3](http://www.ncbi.nlm.nih.gov/entrez/query.fcgi?db=gene&cmd=Retrieve&dopt=full_report&list_uids=54495) | thioredoxin related transmembrane protein 3 |
| [Details](https://mirdb.org/cgi-bin/target_detail.cgi?targetID=2492440) | 125 | 68 | hsa-miR-33b-3p | [BEND4](http://www.ncbi.nlm.nih.gov/entrez/query.fcgi?db=gene&cmd=Retrieve&dopt=full_report&list_uids=389206) | BEN domain containing 4 |
| [Details](https://mirdb.org/cgi-bin/target_detail.cgi?targetID=2492444) | 126 | 68 | hsa-miR-33b-3p | [SPRYD7](http://www.ncbi.nlm.nih.gov/entrez/query.fcgi?db=gene&cmd=Retrieve&dopt=full_report&list_uids=57213) | SPRY domain containing 7 |
| [Details](https://mirdb.org/cgi-bin/target_detail.cgi?targetID=2492560) | 127 | 68 | hsa-miR-33b-3p | [NANOS1](http://www.ncbi.nlm.nih.gov/entrez/query.fcgi?db=gene&cmd=Retrieve&dopt=full_report&list_uids=340719) | nanos C2HC-type zinc finger 1 |
| [Details](https://mirdb.org/cgi-bin/target_detail.cgi?targetID=2492609) | 128 | 68 | hsa-miR-33b-3p | [TRIM55](http://www.ncbi.nlm.nih.gov/entrez/query.fcgi?db=gene&cmd=Retrieve&dopt=full_report&list_uids=84675) | tripartite motif containing 55 |
| [Details](https://mirdb.org/cgi-bin/target_detail.cgi?targetID=2492399) | 129 | 67 | hsa-miR-33b-3p | [GJC1](http://www.ncbi.nlm.nih.gov/entrez/query.fcgi?db=gene&cmd=Retrieve&dopt=full_report&list_uids=10052) | gap junction protein gamma 1 |
| [Details](https://mirdb.org/cgi-bin/target_detail.cgi?targetID=2492420) | 130 | 67 | hsa-miR-33b-3p | [GABPB1](http://www.ncbi.nlm.nih.gov/entrez/query.fcgi?db=gene&cmd=Retrieve&dopt=full_report&list_uids=2553) | GA binding protein transcription factor subunit beta 1 |
| [Details](https://mirdb.org/cgi-bin/target_detail.cgi?targetID=2492429) | 131 | 67 | hsa-miR-33b-3p | [FER1L6](http://www.ncbi.nlm.nih.gov/entrez/query.fcgi?db=gene&cmd=Retrieve&dopt=full_report&list_uids=654463) | fer-1 like family member 6 |
| [Details](https://mirdb.org/cgi-bin/target_detail.cgi?targetID=2492478) | 132 | 67 | hsa-miR-33b-3p | [PRKAR1A](http://www.ncbi.nlm.nih.gov/entrez/query.fcgi?db=gene&cmd=Retrieve&dopt=full_report&list_uids=5573) | protein kinase cAMP-dependent type I regulatory subunit alpha |
| [Details](https://mirdb.org/cgi-bin/target_detail.cgi?targetID=2492500) | 133 | 67 | hsa-miR-33b-3p | [MMP19](http://www.ncbi.nlm.nih.gov/entrez/query.fcgi?db=gene&cmd=Retrieve&dopt=full_report&list_uids=4327) | matrix metallopeptidase 19 |
| [Details](https://mirdb.org/cgi-bin/target_detail.cgi?targetID=2492515) | 134 | 67 | hsa-miR-33b-3p | [HIC1](http://www.ncbi.nlm.nih.gov/entrez/query.fcgi?db=gene&cmd=Retrieve&dopt=full_report&list_uids=3090) | HIC ZBTB transcriptional repressor 1 |
| [Details](https://mirdb.org/cgi-bin/target_detail.cgi?targetID=2492600) | 135 | 67 | hsa-miR-33b-3p | [NFIA](http://www.ncbi.nlm.nih.gov/entrez/query.fcgi?db=gene&cmd=Retrieve&dopt=full_report&list_uids=4774) | nuclear factor I A |
| [Details](https://mirdb.org/cgi-bin/target_detail.cgi?targetID=2492624) | 136 | 67 | hsa-miR-33b-3p | [GPR155](http://www.ncbi.nlm.nih.gov/entrez/query.fcgi?db=gene&cmd=Retrieve&dopt=full_report&list_uids=151556) | G protein-coupled receptor 155 |
| [Details](https://mirdb.org/cgi-bin/target_detail.cgi?targetID=2492435) | 137 | 66 | hsa-miR-33b-3p | [RNF43](http://www.ncbi.nlm.nih.gov/entrez/query.fcgi?db=gene&cmd=Retrieve&dopt=full_report&list_uids=54894) | ring finger protein 43 |
| [Details](https://mirdb.org/cgi-bin/target_detail.cgi?targetID=2492490) | 138 | 66 | hsa-miR-33b-3p | [DES](http://www.ncbi.nlm.nih.gov/entrez/query.fcgi?db=gene&cmd=Retrieve&dopt=full_report&list_uids=1674) | desmin |
| [Details](https://mirdb.org/cgi-bin/target_detail.cgi?targetID=2492553) | 139 | 66 | hsa-miR-33b-3p | [SAMD12](http://www.ncbi.nlm.nih.gov/entrez/query.fcgi?db=gene&cmd=Retrieve&dopt=full_report&list_uids=401474) | sterile alpha motif domain containing 12 |
| [Details](https://mirdb.org/cgi-bin/target_detail.cgi?targetID=2492584) | 140 | 66 | hsa-miR-33b-3p | [IFNA2](http://www.ncbi.nlm.nih.gov/entrez/query.fcgi?db=gene&cmd=Retrieve&dopt=full_report&list_uids=3440) | interferon alpha 2 |
| [Details](https://mirdb.org/cgi-bin/target_detail.cgi?targetID=2492605) | 141 | 66 | hsa-miR-33b-3p | [MARCH8](http://www.ncbi.nlm.nih.gov/entrez/query.fcgi?db=gene&cmd=Retrieve&dopt=full_report&list_uids=220972) | membrane associated ring-CH-type finger 8 |
| [Details](https://mirdb.org/cgi-bin/target_detail.cgi?targetID=2492652) | 142 | 66 | hsa-miR-33b-3p | [MRNIP](http://www.ncbi.nlm.nih.gov/entrez/query.fcgi?db=gene&cmd=Retrieve&dopt=full_report&list_uids=51149) | MRN complex interacting protein |
| [Details](https://mirdb.org/cgi-bin/target_detail.cgi?targetID=2492669) | 143 | 66 | hsa-miR-33b-3p | [MORN4](http://www.ncbi.nlm.nih.gov/entrez/query.fcgi?db=gene&cmd=Retrieve&dopt=full_report&list_uids=118812) | MORN repeat containing 4 |
| [Details](https://mirdb.org/cgi-bin/target_detail.cgi?targetID=2492401) | 144 | 65 | hsa-miR-33b-3p | [FAM13A](http://www.ncbi.nlm.nih.gov/entrez/query.fcgi?db=gene&cmd=Retrieve&dopt=full_report&list_uids=10144) | family with sequence similarity 13 member A |
| [Details](https://mirdb.org/cgi-bin/target_detail.cgi?targetID=2492408) | 145 | 65 | hsa-miR-33b-3p | [SMARCAD1](http://www.ncbi.nlm.nih.gov/entrez/query.fcgi?db=gene&cmd=Retrieve&dopt=full_report&list_uids=56916) | SWI/SNF-related, matrix-associated actin-dependent regulator of chromatin, subfamily a, containing DEAD/H box 1 |
| [Details](https://mirdb.org/cgi-bin/target_detail.cgi?targetID=2492466) | 146 | 65 | hsa-miR-33b-3p | [PTPRB](http://www.ncbi.nlm.nih.gov/entrez/query.fcgi?db=gene&cmd=Retrieve&dopt=full_report&list_uids=5787) | protein tyrosine phosphatase, receptor type B |
| [Details](https://mirdb.org/cgi-bin/target_detail.cgi?targetID=2492483) | 147 | 65 | hsa-miR-33b-3p | [ARIH1](http://www.ncbi.nlm.nih.gov/entrez/query.fcgi?db=gene&cmd=Retrieve&dopt=full_report&list_uids=25820) | ariadne RBR E3 ubiquitin protein ligase 1 |
| [Details](https://mirdb.org/cgi-bin/target_detail.cgi?targetID=2492510) | 148 | 65 | hsa-miR-33b-3p | [UBR5](http://www.ncbi.nlm.nih.gov/entrez/query.fcgi?db=gene&cmd=Retrieve&dopt=full_report&list_uids=51366) | ubiquitin protein ligase E3 component n-recognin 5 |
| [Details](https://mirdb.org/cgi-bin/target_detail.cgi?targetID=2492511) | 149 | 65 | hsa-miR-33b-3p | [ZFAND5](http://www.ncbi.nlm.nih.gov/entrez/query.fcgi?db=gene&cmd=Retrieve&dopt=full_report&list_uids=7763) | zinc finger AN1-type containing 5 |
| [Details](https://mirdb.org/cgi-bin/target_detail.cgi?targetID=2492541) | 150 | 65 | hsa-miR-33b-3p | [RBM12B](http://www.ncbi.nlm.nih.gov/entrez/query.fcgi?db=gene&cmd=Retrieve&dopt=full_report&list_uids=389677) | RNA binding motif protein 12B |
| [Details](https://mirdb.org/cgi-bin/target_detail.cgi?targetID=2492639) | 151 | 65 | hsa-miR-33b-3p | [UBN2](http://www.ncbi.nlm.nih.gov/entrez/query.fcgi?db=gene&cmd=Retrieve&dopt=full_report&list_uids=254048) | ubinuclein 2 |
| [Details](https://mirdb.org/cgi-bin/target_detail.cgi?targetID=2492546) | 152 | 64 | hsa-miR-33b-3p | [SV2B](http://www.ncbi.nlm.nih.gov/entrez/query.fcgi?db=gene&cmd=Retrieve&dopt=full_report&list_uids=9899) | synaptic vesicle glycoprotein 2B |
| [Details](https://mirdb.org/cgi-bin/target_detail.cgi?targetID=2492587) | 153 | 64 | hsa-miR-33b-3p | [TNFSF12](http://www.ncbi.nlm.nih.gov/entrez/query.fcgi?db=gene&cmd=Retrieve&dopt=full_report&list_uids=8742) | TNF superfamily member 12 |
| [Details](https://mirdb.org/cgi-bin/target_detail.cgi?targetID=2492422) | 154 | 63 | hsa-miR-33b-3p | [TCF12](http://www.ncbi.nlm.nih.gov/entrez/query.fcgi?db=gene&cmd=Retrieve&dopt=full_report&list_uids=6938) | transcription factor 12 |
| [Details](https://mirdb.org/cgi-bin/target_detail.cgi?targetID=2492460) | 155 | 63 | hsa-miR-33b-3p | [SHISA2](http://www.ncbi.nlm.nih.gov/entrez/query.fcgi?db=gene&cmd=Retrieve&dopt=full_report&list_uids=387914) | shisa family member 2 |
| [Details](https://mirdb.org/cgi-bin/target_detail.cgi?targetID=2492527) | 156 | 63 | hsa-miR-33b-3p | [RGS6](http://www.ncbi.nlm.nih.gov/entrez/query.fcgi?db=gene&cmd=Retrieve&dopt=full_report&list_uids=9628) | regulator of G protein signaling 6 |
| [Details](https://mirdb.org/cgi-bin/target_detail.cgi?targetID=2492529) | 157 | 63 | hsa-miR-33b-3p | [PHF8](http://www.ncbi.nlm.nih.gov/entrez/query.fcgi?db=gene&cmd=Retrieve&dopt=full_report&list_uids=23133) | PHD finger protein 8 |
| [Details](https://mirdb.org/cgi-bin/target_detail.cgi?targetID=2492534) | 158 | 63 | hsa-miR-33b-3p | [PTPN3](http://www.ncbi.nlm.nih.gov/entrez/query.fcgi?db=gene&cmd=Retrieve&dopt=full_report&list_uids=5774) | protein tyrosine phosphatase, non-receptor type 3 |
| [Details](https://mirdb.org/cgi-bin/target_detail.cgi?targetID=2492566) | 159 | 63 | hsa-miR-33b-3p | [UBR7](http://www.ncbi.nlm.nih.gov/entrez/query.fcgi?db=gene&cmd=Retrieve&dopt=full_report&list_uids=55148) | ubiquitin protein ligase E3 component n-recognin 7 (putative) |
| [Details](https://mirdb.org/cgi-bin/target_detail.cgi?targetID=2492595) | 160 | 63 | hsa-miR-33b-3p | [PRLR](http://www.ncbi.nlm.nih.gov/entrez/query.fcgi?db=gene&cmd=Retrieve&dopt=full_report&list_uids=5618) | prolactin receptor |
| [Details](https://mirdb.org/cgi-bin/target_detail.cgi?targetID=2492634) | 161 | 63 | hsa-miR-33b-3p | [ZBTB20](http://www.ncbi.nlm.nih.gov/entrez/query.fcgi?db=gene&cmd=Retrieve&dopt=full_report&list_uids=26137) | zinc finger and BTB domain containing 20 |
| [Details](https://mirdb.org/cgi-bin/target_detail.cgi?targetID=2492396) | 162 | 62 | hsa-miR-33b-3p | [GATAD2B](http://www.ncbi.nlm.nih.gov/entrez/query.fcgi?db=gene&cmd=Retrieve&dopt=full_report&list_uids=57459) | GATA zinc finger domain containing 2B |
| [Details](https://mirdb.org/cgi-bin/target_detail.cgi?targetID=2492411) | 163 | 62 | hsa-miR-33b-3p | [STS](http://www.ncbi.nlm.nih.gov/entrez/query.fcgi?db=gene&cmd=Retrieve&dopt=full_report&list_uids=412) | steroid sulfatase |
| [Details](https://mirdb.org/cgi-bin/target_detail.cgi?targetID=2492484) | 164 | 62 | hsa-miR-33b-3p | [ACBD3](http://www.ncbi.nlm.nih.gov/entrez/query.fcgi?db=gene&cmd=Retrieve&dopt=full_report&list_uids=64746) | acyl-CoA binding domain containing 3 |
| [Details](https://mirdb.org/cgi-bin/target_detail.cgi?targetID=2492487) | 165 | 62 | hsa-miR-33b-3p | [PBX1](http://www.ncbi.nlm.nih.gov/entrez/query.fcgi?db=gene&cmd=Retrieve&dopt=full_report&list_uids=5087) | PBX homeobox 1 |
| [Details](https://mirdb.org/cgi-bin/target_detail.cgi?targetID=2492539) | 166 | 62 | hsa-miR-33b-3p | [SRP68](http://www.ncbi.nlm.nih.gov/entrez/query.fcgi?db=gene&cmd=Retrieve&dopt=full_report&list_uids=6730) | signal recognition particle 68 |
| [Details](https://mirdb.org/cgi-bin/target_detail.cgi?targetID=2492611) | 167 | 62 | hsa-miR-33b-3p | [PHC1](http://www.ncbi.nlm.nih.gov/entrez/query.fcgi?db=gene&cmd=Retrieve&dopt=full_report&list_uids=1911) | polyhomeotic homolog 1 |
| [Details](https://mirdb.org/cgi-bin/target_detail.cgi?targetID=2492626) | 168 | 62 | hsa-miR-33b-3p | [CEMIP2](http://www.ncbi.nlm.nih.gov/entrez/query.fcgi?db=gene&cmd=Retrieve&dopt=full_report&list_uids=23670) | cell migration inducing hyaluronidase 2 |
| [Details](https://mirdb.org/cgi-bin/target_detail.cgi?targetID=2492660) | 169 | 62 | hsa-miR-33b-3p | [GRIA3](http://www.ncbi.nlm.nih.gov/entrez/query.fcgi?db=gene&cmd=Retrieve&dopt=full_report&list_uids=2892) | glutamate ionotropic receptor AMPA type subunit 3 |
| [Details](https://mirdb.org/cgi-bin/target_detail.cgi?targetID=2492414) | 170 | 61 | hsa-miR-33b-3p | [FBXL20](http://www.ncbi.nlm.nih.gov/entrez/query.fcgi?db=gene&cmd=Retrieve&dopt=full_report&list_uids=84961) | F-box and leucine rich repeat protein 20 |
| [Details](https://mirdb.org/cgi-bin/target_detail.cgi?targetID=2492428) | 171 | 61 | hsa-miR-33b-3p | [HHIP](http://www.ncbi.nlm.nih.gov/entrez/query.fcgi?db=gene&cmd=Retrieve&dopt=full_report&list_uids=64399) | hedgehog interacting protein |
| [Details](https://mirdb.org/cgi-bin/target_detail.cgi?targetID=2492501) | 172 | 61 | hsa-miR-33b-3p | [DCAF12](http://www.ncbi.nlm.nih.gov/entrez/query.fcgi?db=gene&cmd=Retrieve&dopt=full_report&list_uids=25853) | DDB1 and CUL4 associated factor 12 |
| [Details](https://mirdb.org/cgi-bin/target_detail.cgi?targetID=2492520) | 173 | 61 | hsa-miR-33b-3p | [ZFHX3](http://www.ncbi.nlm.nih.gov/entrez/query.fcgi?db=gene&cmd=Retrieve&dopt=full_report&list_uids=463) | zinc finger homeobox 3 |
| [Details](https://mirdb.org/cgi-bin/target_detail.cgi?targetID=2492574) | 174 | 61 | hsa-miR-33b-3p | [EGR4](http://www.ncbi.nlm.nih.gov/entrez/query.fcgi?db=gene&cmd=Retrieve&dopt=full_report&list_uids=1961) | early growth response 4 |
| [Details](https://mirdb.org/cgi-bin/target_detail.cgi?targetID=2492597) | 175 | 61 | hsa-miR-33b-3p | [ARHGAP26](http://www.ncbi.nlm.nih.gov/entrez/query.fcgi?db=gene&cmd=Retrieve&dopt=full_report&list_uids=23092) | Rho GTPase activating protein 26 |
| [Details](https://mirdb.org/cgi-bin/target_detail.cgi?targetID=2492610) | 176 | 61 | hsa-miR-33b-3p | [TMEM273](http://www.ncbi.nlm.nih.gov/entrez/query.fcgi?db=gene&cmd=Retrieve&dopt=full_report&list_uids=170371) | transmembrane protein 273 |
| [Details](https://mirdb.org/cgi-bin/target_detail.cgi?targetID=2492632) | 177 | 61 | hsa-miR-33b-3p | [VAPA](http://www.ncbi.nlm.nih.gov/entrez/query.fcgi?db=gene&cmd=Retrieve&dopt=full_report&list_uids=9218) | VAMP associated protein A |
| [Details](https://mirdb.org/cgi-bin/target_detail.cgi?targetID=2492673) | 178 | 61 | hsa-miR-33b-3p | [PFN4](http://www.ncbi.nlm.nih.gov/entrez/query.fcgi?db=gene&cmd=Retrieve&dopt=full_report&list_uids=375189) | profilin family member 4 |
| [Details](https://mirdb.org/cgi-bin/target_detail.cgi?targetID=2492438) | 179 | 60 | hsa-miR-33b-3p | [SS18L1](http://www.ncbi.nlm.nih.gov/entrez/query.fcgi?db=gene&cmd=Retrieve&dopt=full_report&list_uids=26039) | SS18L1, nBAF chromatin remodeling complex subunit |
| [Details](https://mirdb.org/cgi-bin/target_detail.cgi?targetID=2492463) | 180 | 60 | hsa-miR-33b-3p | [E2F3](http://www.ncbi.nlm.nih.gov/entrez/query.fcgi?db=gene&cmd=Retrieve&dopt=full_report&list_uids=1871) | E2F transcription factor 3 |
| [Details](https://mirdb.org/cgi-bin/target_detail.cgi?targetID=2492488) | 181 | 60 | hsa-miR-33b-3p | [REEP1](http://www.ncbi.nlm.nih.gov/entrez/query.fcgi?db=gene&cmd=Retrieve&dopt=full_report&list_uids=65055) | receptor accessory protein 1 |
| [Details](https://mirdb.org/cgi-bin/target_detail.cgi?targetID=2492589) | 182 | 60 | hsa-miR-33b-3p | [SCUBE3](http://www.ncbi.nlm.nih.gov/entrez/query.fcgi?db=gene&cmd=Retrieve&dopt=full_report&list_uids=222663) | signal peptide, CUB domain and EGF like domain containing 3 |
| [Details](https://mirdb.org/cgi-bin/target_detail.cgi?targetID=2492625) | 183 | 60 | hsa-miR-33b-3p | [KMT2E](http://www.ncbi.nlm.nih.gov/entrez/query.fcgi?db=gene&cmd=Retrieve&dopt=full_report&list_uids=55904) | lysine methyltransferase 2E |
| [Details](https://mirdb.org/cgi-bin/target_detail.cgi?targetID=2492643) | 184 | 60 | hsa-miR-33b-3p | [SOX11](http://www.ncbi.nlm.nih.gov/entrez/query.fcgi?db=gene&cmd=Retrieve&dopt=full_report&list_uids=6664) | SRY-box 11 |
| [Details](https://mirdb.org/cgi-bin/target_detail.cgi?targetID=2492648) | 185 | 60 | hsa-miR-33b-3p | [TRIM71](http://www.ncbi.nlm.nih.gov/entrez/query.fcgi?db=gene&cmd=Retrieve&dopt=full_report&list_uids=131405) | tripartite motif containing 71 |
| [Details](https://mirdb.org/cgi-bin/target_detail.cgi?targetID=2492493) | 186 | 59 | hsa-miR-33b-3p | [FAM13B](http://www.ncbi.nlm.nih.gov/entrez/query.fcgi?db=gene&cmd=Retrieve&dopt=full_report&list_uids=51306) | family with sequence similarity 13 member B |
| [Details](https://mirdb.org/cgi-bin/target_detail.cgi?targetID=2492514) | 187 | 59 | hsa-miR-33b-3p | [ZC3H12B](http://www.ncbi.nlm.nih.gov/entrez/query.fcgi?db=gene&cmd=Retrieve&dopt=full_report&list_uids=340554) | zinc finger CCCH-type containing 12B |
| [Details](https://mirdb.org/cgi-bin/target_detail.cgi?targetID=2492582) | 188 | 59 | hsa-miR-33b-3p | [SNN](http://www.ncbi.nlm.nih.gov/entrez/query.fcgi?db=gene&cmd=Retrieve&dopt=full_report&list_uids=8303) | stannin |
| [Details](https://mirdb.org/cgi-bin/target_detail.cgi?targetID=2492585) | 189 | 59 | hsa-miR-33b-3p | [DENND1B](http://www.ncbi.nlm.nih.gov/entrez/query.fcgi?db=gene&cmd=Retrieve&dopt=full_report&list_uids=163486) | DENN domain containing 1B |
| [Details](https://mirdb.org/cgi-bin/target_detail.cgi?targetID=2492613) | 190 | 59 | hsa-miR-33b-3p | [SLC44A1](http://www.ncbi.nlm.nih.gov/entrez/query.fcgi?db=gene&cmd=Retrieve&dopt=full_report&list_uids=23446) | solute carrier family 44 member 1 |
| [Details](https://mirdb.org/cgi-bin/target_detail.cgi?targetID=2492614) | 191 | 59 | hsa-miR-33b-3p | [FMNL3](http://www.ncbi.nlm.nih.gov/entrez/query.fcgi?db=gene&cmd=Retrieve&dopt=full_report&list_uids=91010) | formin like 3 |
| [Details](https://mirdb.org/cgi-bin/target_detail.cgi?targetID=2492615) | 192 | 59 | hsa-miR-33b-3p | [ARL4A](http://www.ncbi.nlm.nih.gov/entrez/query.fcgi?db=gene&cmd=Retrieve&dopt=full_report&list_uids=10124) | ADP ribosylation factor like GTPase 4A |
| [Details](https://mirdb.org/cgi-bin/target_detail.cgi?targetID=2492661) | 193 | 59 | hsa-miR-33b-3p | [ST6GALNAC6](http://www.ncbi.nlm.nih.gov/entrez/query.fcgi?db=gene&cmd=Retrieve&dopt=full_report&list_uids=30815) | ST6 N-acetylgalactosaminide alpha-2,6-sialyltransferase 6 |
| [Details](https://mirdb.org/cgi-bin/target_detail.cgi?targetID=2492670) | 194 | 59 | hsa-miR-33b-3p | [FOXF1](http://www.ncbi.nlm.nih.gov/entrez/query.fcgi?db=gene&cmd=Retrieve&dopt=full_report&list_uids=2294) | forkhead box F1 |
| [Details](https://mirdb.org/cgi-bin/target_detail.cgi?targetID=2492672) | 195 | 59 | hsa-miR-33b-3p | [GCLC](http://www.ncbi.nlm.nih.gov/entrez/query.fcgi?db=gene&cmd=Retrieve&dopt=full_report&list_uids=2729) | glutamate-cysteine ligase catalytic subunit |
| [Details](https://mirdb.org/cgi-bin/target_detail.cgi?targetID=2492403) | 196 | 58 | hsa-miR-33b-3p | [ADCY6](http://www.ncbi.nlm.nih.gov/entrez/query.fcgi?db=gene&cmd=Retrieve&dopt=full_report&list_uids=112) | adenylate cyclase 6 |
| [Details](https://mirdb.org/cgi-bin/target_detail.cgi?targetID=2492404) | 197 | 58 | hsa-miR-33b-3p | [NTN1](http://www.ncbi.nlm.nih.gov/entrez/query.fcgi?db=gene&cmd=Retrieve&dopt=full_report&list_uids=9423) | netrin 1 |
| [Details](https://mirdb.org/cgi-bin/target_detail.cgi?targetID=2492413) | 198 | 58 | hsa-miR-33b-3p | [ZNF2](http://www.ncbi.nlm.nih.gov/entrez/query.fcgi?db=gene&cmd=Retrieve&dopt=full_report&list_uids=7549) | zinc finger protein 2 |
| [Details](https://mirdb.org/cgi-bin/target_detail.cgi?targetID=2492430) | 199 | 58 | hsa-miR-33b-3p | [KIAA1671](http://www.ncbi.nlm.nih.gov/entrez/query.fcgi?db=gene&cmd=Retrieve&dopt=full_report&list_uids=85379) | KIAA1671 |
| [Details](https://mirdb.org/cgi-bin/target_detail.cgi?targetID=2492507) | 200 | 58 | hsa-miR-33b-3p | [BORA](http://www.ncbi.nlm.nih.gov/entrez/query.fcgi?db=gene&cmd=Retrieve&dopt=full_report&list_uids=79866) | bora, aurora kinase A activator |
| [Details](https://mirdb.org/cgi-bin/target_detail.cgi?targetID=2492616) | 201 | 58 | hsa-miR-33b-3p | [SLC48A1](http://www.ncbi.nlm.nih.gov/entrez/query.fcgi?db=gene&cmd=Retrieve&dopt=full_report&list_uids=55652) | solute carrier family 48 member 1 |
| [Details](https://mirdb.org/cgi-bin/target_detail.cgi?targetID=2492645) | 202 | 58 | hsa-miR-33b-3p | [SORBS2](http://www.ncbi.nlm.nih.gov/entrez/query.fcgi?db=gene&cmd=Retrieve&dopt=full_report&list_uids=8470) | sorbin and SH3 domain containing 2 |
| [Details](https://mirdb.org/cgi-bin/target_detail.cgi?targetID=2492465) | 203 | 57 | hsa-miR-33b-3p | [TRIM22](http://www.ncbi.nlm.nih.gov/entrez/query.fcgi?db=gene&cmd=Retrieve&dopt=full_report&list_uids=10346) | tripartite motif containing 22 |
| [Details](https://mirdb.org/cgi-bin/target_detail.cgi?targetID=2492477) | 204 | 57 | hsa-miR-33b-3p | [TTYH1](http://www.ncbi.nlm.nih.gov/entrez/query.fcgi?db=gene&cmd=Retrieve&dopt=full_report&list_uids=57348) | tweety family member 1 |
| [Details](https://mirdb.org/cgi-bin/target_detail.cgi?targetID=2492528) | 205 | 57 | hsa-miR-33b-3p | [JPH4](http://www.ncbi.nlm.nih.gov/entrez/query.fcgi?db=gene&cmd=Retrieve&dopt=full_report&list_uids=84502) | junctophilin 4 |
| [Details](https://mirdb.org/cgi-bin/target_detail.cgi?targetID=2492532) | 206 | 57 | hsa-miR-33b-3p | [ATP1A2](http://www.ncbi.nlm.nih.gov/entrez/query.fcgi?db=gene&cmd=Retrieve&dopt=full_report&list_uids=477) | ATPase Na+/K+ transporting subunit alpha 2 |
| [Details](https://mirdb.org/cgi-bin/target_detail.cgi?targetID=2492550) | 207 | 57 | hsa-miR-33b-3p | [CS](http://www.ncbi.nlm.nih.gov/entrez/query.fcgi?db=gene&cmd=Retrieve&dopt=full_report&list_uids=1431) | citrate synthase |
| [Details](https://mirdb.org/cgi-bin/target_detail.cgi?targetID=2492561) | 208 | 57 | hsa-miR-33b-3p | [INHBA](http://www.ncbi.nlm.nih.gov/entrez/query.fcgi?db=gene&cmd=Retrieve&dopt=full_report&list_uids=3624) | inhibin subunit beta A |
| [Details](https://mirdb.org/cgi-bin/target_detail.cgi?targetID=2492604) | 209 | 57 | hsa-miR-33b-3p | [SLC28A1](http://www.ncbi.nlm.nih.gov/entrez/query.fcgi?db=gene&cmd=Retrieve&dopt=full_report&list_uids=9154) | solute carrier family 28 member 1 |
| [Details](https://mirdb.org/cgi-bin/target_detail.cgi?targetID=2492623) | 210 | 57 | hsa-miR-33b-3p | [AREL1](http://www.ncbi.nlm.nih.gov/entrez/query.fcgi?db=gene&cmd=Retrieve&dopt=full_report&list_uids=9870) | apoptosis resistant E3 ubiquitin protein ligase 1 |
| [Details](https://mirdb.org/cgi-bin/target_detail.cgi?targetID=2492647) | 211 | 57 | hsa-miR-33b-3p | [C8orf48](http://www.ncbi.nlm.nih.gov/entrez/query.fcgi?db=gene&cmd=Retrieve&dopt=full_report&list_uids=157773) | chromosome 8 open reading frame 48 |
| [Details](https://mirdb.org/cgi-bin/target_detail.cgi?targetID=2492431) | 212 | 56 | hsa-miR-33b-3p | [ARHGAP20](http://www.ncbi.nlm.nih.gov/entrez/query.fcgi?db=gene&cmd=Retrieve&dopt=full_report&list_uids=57569) | Rho GTPase activating protein 20 |
| [Details](https://mirdb.org/cgi-bin/target_detail.cgi?targetID=2492451) | 213 | 56 | hsa-miR-33b-3p | [KCNK2](http://www.ncbi.nlm.nih.gov/entrez/query.fcgi?db=gene&cmd=Retrieve&dopt=full_report&list_uids=3776) | potassium two pore domain channel subfamily K member 2 |
| [Details](https://mirdb.org/cgi-bin/target_detail.cgi?targetID=2492467) | 214 | 56 | hsa-miR-33b-3p | [THBS3](http://www.ncbi.nlm.nih.gov/entrez/query.fcgi?db=gene&cmd=Retrieve&dopt=full_report&list_uids=7059) | thrombospondin 3 |
| [Details](https://mirdb.org/cgi-bin/target_detail.cgi?targetID=2492485) | 215 | 56 | hsa-miR-33b-3p | [STX5](http://www.ncbi.nlm.nih.gov/entrez/query.fcgi?db=gene&cmd=Retrieve&dopt=full_report&list_uids=6811) | syntaxin 5 |
| [Details](https://mirdb.org/cgi-bin/target_detail.cgi?targetID=2492509) | 216 | 56 | hsa-miR-33b-3p | [SUN1](http://www.ncbi.nlm.nih.gov/entrez/query.fcgi?db=gene&cmd=Retrieve&dopt=full_report&list_uids=23353) | Sad1 and UNC84 domain containing 1 |
| [Details](https://mirdb.org/cgi-bin/target_detail.cgi?targetID=2492519) | 217 | 56 | hsa-miR-33b-3p | [ZBTB10](http://www.ncbi.nlm.nih.gov/entrez/query.fcgi?db=gene&cmd=Retrieve&dopt=full_report&list_uids=65986) | zinc finger and BTB domain containing 10 |
| [Details](https://mirdb.org/cgi-bin/target_detail.cgi?targetID=2492521) | 218 | 56 | hsa-miR-33b-3p | [HAUS4](http://www.ncbi.nlm.nih.gov/entrez/query.fcgi?db=gene&cmd=Retrieve&dopt=full_report&list_uids=54930) | HAUS augmin like complex subunit 4 |
| [Details](https://mirdb.org/cgi-bin/target_detail.cgi?targetID=2492598) | 219 | 56 | hsa-miR-33b-3p | [DSG4](http://www.ncbi.nlm.nih.gov/entrez/query.fcgi?db=gene&cmd=Retrieve&dopt=full_report&list_uids=147409) | desmoglein 4 |
| [Details](https://mirdb.org/cgi-bin/target_detail.cgi?targetID=2492636) | 220 | 56 | hsa-miR-33b-3p | [IRF2BP2](http://www.ncbi.nlm.nih.gov/entrez/query.fcgi?db=gene&cmd=Retrieve&dopt=full_report&list_uids=359948) | interferon regulatory factor 2 binding protein 2 |
| [Details](https://mirdb.org/cgi-bin/target_detail.cgi?targetID=2492664) | 221 | 56 | hsa-miR-33b-3p | [STRIP2](http://www.ncbi.nlm.nih.gov/entrez/query.fcgi?db=gene&cmd=Retrieve&dopt=full_report&list_uids=57464) | striatin interacting protein 2 |
| [Details](https://mirdb.org/cgi-bin/target_detail.cgi?targetID=2492409) | 222 | 55 | hsa-miR-33b-3p | [ARHGEF12](http://www.ncbi.nlm.nih.gov/entrez/query.fcgi?db=gene&cmd=Retrieve&dopt=full_report&list_uids=23365) | Rho guanine nucleotide exchange factor 12 |
| [Details](https://mirdb.org/cgi-bin/target_detail.cgi?targetID=2492437) | 223 | 55 | hsa-miR-33b-3p | [PANK3](http://www.ncbi.nlm.nih.gov/entrez/query.fcgi?db=gene&cmd=Retrieve&dopt=full_report&list_uids=79646) | pantothenate kinase 3 |
| [Details](https://mirdb.org/cgi-bin/target_detail.cgi?targetID=2492443) | 224 | 55 | hsa-miR-33b-3p | [ROCK2](http://www.ncbi.nlm.nih.gov/entrez/query.fcgi?db=gene&cmd=Retrieve&dopt=full_report&list_uids=9475) | Rho associated coiled-coil containing protein kinase 2 |
| [Details](https://mirdb.org/cgi-bin/target_detail.cgi?targetID=2492452) | 225 | 55 | hsa-miR-33b-3p | [F2R](http://www.ncbi.nlm.nih.gov/entrez/query.fcgi?db=gene&cmd=Retrieve&dopt=full_report&list_uids=2149) | coagulation factor II thrombin receptor |
| [Details](https://mirdb.org/cgi-bin/target_detail.cgi?targetID=2492468) | 226 | 55 | hsa-miR-33b-3p | [SC5D](http://www.ncbi.nlm.nih.gov/entrez/query.fcgi?db=gene&cmd=Retrieve&dopt=full_report&list_uids=6309) | sterol-C5-desaturase |
| [Details](https://mirdb.org/cgi-bin/target_detail.cgi?targetID=2492473) | 227 | 55 | hsa-miR-33b-3p | [SAMD5](http://www.ncbi.nlm.nih.gov/entrez/query.fcgi?db=gene&cmd=Retrieve&dopt=full_report&list_uids=389432) | sterile alpha motif domain containing 5 |
| [Details](https://mirdb.org/cgi-bin/target_detail.cgi?targetID=2492476) | 228 | 55 | hsa-miR-33b-3p | [KIT](http://www.ncbi.nlm.nih.gov/entrez/query.fcgi?db=gene&cmd=Retrieve&dopt=full_report&list_uids=3815) | KIT proto-oncogene receptor tyrosine kinase |
| [Details](https://mirdb.org/cgi-bin/target_detail.cgi?targetID=2492508) | 229 | 55 | hsa-miR-33b-3p | [SERPINA1](http://www.ncbi.nlm.nih.gov/entrez/query.fcgi?db=gene&cmd=Retrieve&dopt=full_report&list_uids=5265) | serpin family A member 1 |
| [Details](https://mirdb.org/cgi-bin/target_detail.cgi?targetID=2492579) | 230 | 55 | hsa-miR-33b-3p | [RNF149](http://www.ncbi.nlm.nih.gov/entrez/query.fcgi?db=gene&cmd=Retrieve&dopt=full_report&list_uids=284996) | ring finger protein 149 |
| [Details](https://mirdb.org/cgi-bin/target_detail.cgi?targetID=2492631) | 231 | 55 | hsa-miR-33b-3p | [CTSV](http://www.ncbi.nlm.nih.gov/entrez/query.fcgi?db=gene&cmd=Retrieve&dopt=full_report&list_uids=1515) | cathepsin V |
| [Details](https://mirdb.org/cgi-bin/target_detail.cgi?targetID=2492421) | 232 | 54 | hsa-miR-33b-3p | [NAMPT](http://www.ncbi.nlm.nih.gov/entrez/query.fcgi?db=gene&cmd=Retrieve&dopt=full_report&list_uids=10135) | nicotinamide phosphoribosyltransferase |
| [Details](https://mirdb.org/cgi-bin/target_detail.cgi?targetID=2492425) | 233 | 54 | hsa-miR-33b-3p | [ATXN1](http://www.ncbi.nlm.nih.gov/entrez/query.fcgi?db=gene&cmd=Retrieve&dopt=full_report&list_uids=6310) | ataxin 1 |
| [Details](https://mirdb.org/cgi-bin/target_detail.cgi?targetID=2492434) | 234 | 54 | hsa-miR-33b-3p | [TMBIM6](http://www.ncbi.nlm.nih.gov/entrez/query.fcgi?db=gene&cmd=Retrieve&dopt=full_report&list_uids=7009) | transmembrane BAX inhibitor motif containing 6 |
| [Details](https://mirdb.org/cgi-bin/target_detail.cgi?targetID=2492445) | 235 | 54 | hsa-miR-33b-3p | [CHD7](http://www.ncbi.nlm.nih.gov/entrez/query.fcgi?db=gene&cmd=Retrieve&dopt=full_report&list_uids=55636) | chromodomain helicase DNA binding protein 7 |
| [Details](https://mirdb.org/cgi-bin/target_detail.cgi?targetID=2492447) | 236 | 54 | hsa-miR-33b-3p | [SOX4](http://www.ncbi.nlm.nih.gov/entrez/query.fcgi?db=gene&cmd=Retrieve&dopt=full_report&list_uids=6659) | SRY-box 4 |
| [Details](https://mirdb.org/cgi-bin/target_detail.cgi?targetID=2492450) | 237 | 54 | hsa-miR-33b-3p | [FRYL](http://www.ncbi.nlm.nih.gov/entrez/query.fcgi?db=gene&cmd=Retrieve&dopt=full_report&list_uids=285527) | FRY like transcription coactivator |
| [Details](https://mirdb.org/cgi-bin/target_detail.cgi?targetID=2492537) | 238 | 54 | hsa-miR-33b-3p | [MTR](http://www.ncbi.nlm.nih.gov/entrez/query.fcgi?db=gene&cmd=Retrieve&dopt=full_report&list_uids=4548) | 5-methyltetrahydrofolate-homocysteine methyltransferase |
| [Details](https://mirdb.org/cgi-bin/target_detail.cgi?targetID=2492540) | 239 | 54 | hsa-miR-33b-3p | [ZNF570](http://www.ncbi.nlm.nih.gov/entrez/query.fcgi?db=gene&cmd=Retrieve&dopt=full_report&list_uids=148268) | zinc finger protein 570 |
| [Details](https://mirdb.org/cgi-bin/target_detail.cgi?targetID=2492581) | 240 | 54 | hsa-miR-33b-3p | [SUZ12](http://www.ncbi.nlm.nih.gov/entrez/query.fcgi?db=gene&cmd=Retrieve&dopt=full_report&list_uids=23512) | SUZ12, polycomb repressive complex 2 subunit |
| [Details](https://mirdb.org/cgi-bin/target_detail.cgi?targetID=2492608) | 241 | 54 | hsa-miR-33b-3p | [ADCY1](http://www.ncbi.nlm.nih.gov/entrez/query.fcgi?db=gene&cmd=Retrieve&dopt=full_report&list_uids=107) | adenylate cyclase 1 |
| [Details](https://mirdb.org/cgi-bin/target_detail.cgi?targetID=2492635) | 242 | 54 | hsa-miR-33b-3p | [PLA2G12A](http://www.ncbi.nlm.nih.gov/entrez/query.fcgi?db=gene&cmd=Retrieve&dopt=full_report&list_uids=81579) | phospholipase A2 group XIIA |
| [Details](https://mirdb.org/cgi-bin/target_detail.cgi?targetID=2492656) | 243 | 54 | hsa-miR-33b-3p | [PLCB4](http://www.ncbi.nlm.nih.gov/entrez/query.fcgi?db=gene&cmd=Retrieve&dopt=full_report&list_uids=5332) | phospholipase C beta 4 |
| [Details](https://mirdb.org/cgi-bin/target_detail.cgi?targetID=2492400) | 244 | 53 | hsa-miR-33b-3p | [SCNM1](http://www.ncbi.nlm.nih.gov/entrez/query.fcgi?db=gene&cmd=Retrieve&dopt=full_report&list_uids=79005) | sodium channel modifier 1 |
| [Details](https://mirdb.org/cgi-bin/target_detail.cgi?targetID=2492405) | 245 | 53 | hsa-miR-33b-3p | [PLD1](http://www.ncbi.nlm.nih.gov/entrez/query.fcgi?db=gene&cmd=Retrieve&dopt=full_report&list_uids=5337) | phospholipase D1 |
| [Details](https://mirdb.org/cgi-bin/target_detail.cgi?targetID=2492427) | 246 | 53 | hsa-miR-33b-3p | [DCTN4](http://www.ncbi.nlm.nih.gov/entrez/query.fcgi?db=gene&cmd=Retrieve&dopt=full_report&list_uids=51164) | dynactin subunit 4 |
| [Details](https://mirdb.org/cgi-bin/target_detail.cgi?targetID=2492456) | 247 | 53 | hsa-miR-33b-3p | [NUTF2](http://www.ncbi.nlm.nih.gov/entrez/query.fcgi?db=gene&cmd=Retrieve&dopt=full_report&list_uids=10204) | nuclear transport factor 2 |
| [Details](https://mirdb.org/cgi-bin/target_detail.cgi?targetID=2492481) | 248 | 53 | hsa-miR-33b-3p | [KAT6A](http://www.ncbi.nlm.nih.gov/entrez/query.fcgi?db=gene&cmd=Retrieve&dopt=full_report&list_uids=7994) | lysine acetyltransferase 6A |
| [Details](https://mirdb.org/cgi-bin/target_detail.cgi?targetID=2492489) | 249 | 53 | hsa-miR-33b-3p | [TNFAIP8L2-SCNM1](http://www.ncbi.nlm.nih.gov/entrez/query.fcgi?db=gene&cmd=Retrieve&dopt=full_report&list_uids=100534012) | TNFAIP8L2-SCNM1 readthrough |
| [Details](https://mirdb.org/cgi-bin/target_detail.cgi?targetID=2492502) | 250 | 53 | hsa-miR-33b-3p | [TXLNA](http://www.ncbi.nlm.nih.gov/entrez/query.fcgi?db=gene&cmd=Retrieve&dopt=full_report&list_uids=200081) | taxilin alpha |
| [Details](https://mirdb.org/cgi-bin/target_detail.cgi?targetID=2492504) | 251 | 53 | hsa-miR-33b-3p | [MLLT3](http://www.ncbi.nlm.nih.gov/entrez/query.fcgi?db=gene&cmd=Retrieve&dopt=full_report&list_uids=4300) | MLLT3, super elongation complex subunit |
| [Details](https://mirdb.org/cgi-bin/target_detail.cgi?targetID=2492516) | 252 | 53 | hsa-miR-33b-3p | [ZNF257](http://www.ncbi.nlm.nih.gov/entrez/query.fcgi?db=gene&cmd=Retrieve&dopt=full_report&list_uids=113835) | zinc finger protein 257 |
| [Details](https://mirdb.org/cgi-bin/target_detail.cgi?targetID=2492523) | 253 | 53 | hsa-miR-33b-3p | [USP46](http://www.ncbi.nlm.nih.gov/entrez/query.fcgi?db=gene&cmd=Retrieve&dopt=full_report&list_uids=64854) | ubiquitin specific peptidase 46 |
| [Details](https://mirdb.org/cgi-bin/target_detail.cgi?targetID=2492533) | 254 | 53 | hsa-miR-33b-3p | [VDAC1](http://www.ncbi.nlm.nih.gov/entrez/query.fcgi?db=gene&cmd=Retrieve&dopt=full_report&list_uids=7416) | voltage dependent anion channel 1 |
| [Details](https://mirdb.org/cgi-bin/target_detail.cgi?targetID=2492559) | 255 | 53 | hsa-miR-33b-3p | [LATS1](http://www.ncbi.nlm.nih.gov/entrez/query.fcgi?db=gene&cmd=Retrieve&dopt=full_report&list_uids=9113) | large tumor suppressor kinase 1 |
| [Details](https://mirdb.org/cgi-bin/target_detail.cgi?targetID=2492653) | 256 | 53 | hsa-miR-33b-3p | [RHD](http://www.ncbi.nlm.nih.gov/entrez/query.fcgi?db=gene&cmd=Retrieve&dopt=full_report&list_uids=6007) | Rh blood group D antigen |
| [Details](https://mirdb.org/cgi-bin/target_detail.cgi?targetID=2492657) | 257 | 53 | hsa-miR-33b-3p | [RORA](http://www.ncbi.nlm.nih.gov/entrez/query.fcgi?db=gene&cmd=Retrieve&dopt=full_report&list_uids=6095) | RAR related orphan receptor A |
| [Details](https://mirdb.org/cgi-bin/target_detail.cgi?targetID=2492663) | 258 | 53 | hsa-miR-33b-3p | [MON2](http://www.ncbi.nlm.nih.gov/entrez/query.fcgi?db=gene&cmd=Retrieve&dopt=full_report&list_uids=23041) | MON2 homolog, regulator of endosome-to-Golgi trafficking |
| [Details](https://mirdb.org/cgi-bin/target_detail.cgi?targetID=2492671) | 259 | 53 | hsa-miR-33b-3p | [VEPH1](http://www.ncbi.nlm.nih.gov/entrez/query.fcgi?db=gene&cmd=Retrieve&dopt=full_report&list_uids=79674) | ventricular zone expressed PH domain containing 1 |
| [Details](https://mirdb.org/cgi-bin/target_detail.cgi?targetID=2492416) | 260 | 52 | hsa-miR-33b-3p | [SLC30A7](http://www.ncbi.nlm.nih.gov/entrez/query.fcgi?db=gene&cmd=Retrieve&dopt=full_report&list_uids=148867) | solute carrier family 30 member 7 |
| [Details](https://mirdb.org/cgi-bin/target_detail.cgi?targetID=2492461) | 261 | 52 | hsa-miR-33b-3p | [ANK3](http://www.ncbi.nlm.nih.gov/entrez/query.fcgi?db=gene&cmd=Retrieve&dopt=full_report&list_uids=288) | ankyrin 3 |
| [Details](https://mirdb.org/cgi-bin/target_detail.cgi?targetID=2492547) | 262 | 52 | hsa-miR-33b-3p | [FAT3](http://www.ncbi.nlm.nih.gov/entrez/query.fcgi?db=gene&cmd=Retrieve&dopt=full_report&list_uids=120114) | FAT atypical cadherin 3 |
| [Details](https://mirdb.org/cgi-bin/target_detail.cgi?targetID=2492565) | 263 | 52 | hsa-miR-33b-3p | [STAMBP](http://www.ncbi.nlm.nih.gov/entrez/query.fcgi?db=gene&cmd=Retrieve&dopt=full_report&list_uids=10617) | STAM binding protein |
| [Details](https://mirdb.org/cgi-bin/target_detail.cgi?targetID=2492628) | 264 | 52 | hsa-miR-33b-3p | [DCLK1](http://www.ncbi.nlm.nih.gov/entrez/query.fcgi?db=gene&cmd=Retrieve&dopt=full_report&list_uids=9201) | doublecortin like kinase 1 |
| [Details](https://mirdb.org/cgi-bin/target_detail.cgi?targetID=2492644) | 265 | 52 | hsa-miR-33b-3p | [LDLRAD2](http://www.ncbi.nlm.nih.gov/entrez/query.fcgi?db=gene&cmd=Retrieve&dopt=full_report&list_uids=401944) | low density lipoprotein receptor class A domain containing 2 |
| [Details](https://mirdb.org/cgi-bin/target_detail.cgi?targetID=2492649) | 266 | 52 | hsa-miR-33b-3p | [TRIM35](http://www.ncbi.nlm.nih.gov/entrez/query.fcgi?db=gene&cmd=Retrieve&dopt=full_report&list_uids=23087) | tripartite motif containing 35 |
| [Details](https://mirdb.org/cgi-bin/target_detail.cgi?targetID=2492419) | 267 | 51 | hsa-miR-33b-3p | [U2SURP](http://www.ncbi.nlm.nih.gov/entrez/query.fcgi?db=gene&cmd=Retrieve&dopt=full_report&list_uids=23350) | U2 snRNP associated SURP domain containing |
| [Details](https://mirdb.org/cgi-bin/target_detail.cgi?targetID=2492464) | 268 | 51 | hsa-miR-33b-3p | [GIGYF1](http://www.ncbi.nlm.nih.gov/entrez/query.fcgi?db=gene&cmd=Retrieve&dopt=full_report&list_uids=64599) | GRB10 interacting GYF protein 1 |
| [Details](https://mirdb.org/cgi-bin/target_detail.cgi?targetID=2492590) | 269 | 51 | hsa-miR-33b-3p | [C11orf87](http://www.ncbi.nlm.nih.gov/entrez/query.fcgi?db=gene&cmd=Retrieve&dopt=full_report&list_uids=399947) | chromosome 11 open reading frame 87 |
| [Details](https://mirdb.org/cgi-bin/target_detail.cgi?targetID=2492662) | 270 | 51 | hsa-miR-33b-3p | [RTN4](http://www.ncbi.nlm.nih.gov/entrez/query.fcgi?db=gene&cmd=Retrieve&dopt=full_report&list_uids=57142) | reticulon 4 |
| [Details](https://mirdb.org/cgi-bin/target_detail.cgi?targetID=2492458) | 271 | 50 | hsa-miR-33b-3p | [ATP5MPL](http://www.ncbi.nlm.nih.gov/entrez/query.fcgi?db=gene&cmd=Retrieve&dopt=full_report&list_uids=9556) | ATP synthase membrane subunit 6.8PL |
| [Details](https://mirdb.org/cgi-bin/target_detail.cgi?targetID=2492471) | 272 | 50 | hsa-miR-33b-3p | [MBOAT2](http://www.ncbi.nlm.nih.gov/entrez/query.fcgi?db=gene&cmd=Retrieve&dopt=full_report&list_uids=129642) | membrane bound O-acyltransferase domain containing 2 |
| [Details](https://mirdb.org/cgi-bin/target_detail.cgi?targetID=2492524) | 273 | 50 | hsa-miR-33b-3p | [SRP9](http://www.ncbi.nlm.nih.gov/entrez/query.fcgi?db=gene&cmd=Retrieve&dopt=full_report&list_uids=6726) | signal recognition particle 9 |
| [Details](https://mirdb.org/cgi-bin/target_detail.cgi?targetID=2492530) | 274 | 50 | hsa-miR-33b-3p | [CISD3](http://www.ncbi.nlm.nih.gov/entrez/query.fcgi?db=gene&cmd=Retrieve&dopt=full_report&list_uids=284106) | CDGSH iron sulfur domain 3 |
| [Details](https://mirdb.org/cgi-bin/target_detail.cgi?targetID=2492568) | 275 | 50 | hsa-miR-33b-3p | [HOGA1](http://www.ncbi.nlm.nih.gov/entrez/query.fcgi?db=gene&cmd=Retrieve&dopt=full_report&list_uids=112817) | 4-hydroxy-2-oxoglutarate aldolase 1 |
| [Details](https://mirdb.org/cgi-bin/target_detail.cgi?targetID=2492578) | 276 | 50 | hsa-miR-33b-3p | [SESN3](http://www.ncbi.nlm.nih.gov/entrez/query.fcgi?db=gene&cmd=Retrieve&dopt=full_report&list_uids=143686) | sestrin 3 |
| [Details](https://mirdb.org/cgi-bin/target_detail.cgi?targetID=2492593) | 277 | 50 | hsa-miR-33b-3p | [RETREG2](http://www.ncbi.nlm.nih.gov/entrez/query.fcgi?db=gene&cmd=Retrieve&dopt=full_report&list_uids=79137) | reticulophagy regulator family member 2 |
| [Details](https://mirdb.org/cgi-bin/target_detail.cgi?targetID=2492599) | 278 | 50 | hsa-miR-33b-3p | [CARD10](http://www.ncbi.nlm.nih.gov/entrez/query.fcgi?db=gene&cmd=Retrieve&dopt=full_report&list_uids=29775) | caspase recruitment domain family member 10 |
| [Details](https://mirdb.org/cgi-bin/target_detail.cgi?targetID=2492619) | 279 | 50 | hsa-miR-33b-3p | [SPOCK2](http://www.ncbi.nlm.nih.gov/entrez/query.fcgi?db=gene&cmd=Retrieve&dopt=full_report&list_uids=9806) | SPARC (osteonectin), cwcv and kazal like domains proteoglycan 2 |
| [Details](https://mirdb.org/cgi-bin/target_detail.cgi?targetID=2492622) | 280 | 50 | hsa-miR-33b-3p | [TSPAN1](http://www.ncbi.nlm.nih.gov/entrez/query.fcgi?db=gene&cmd=Retrieve&dopt=full_report&list_uids=10103) | tetraspanin 1 |
